# Safety and durability of mRNA-1273–induced SARS-CoV-2 immune responses in adolescents: results from the phase 2/3 TeenCOVE trial

**DOI:** 10.1016/j.eclinm.2024.102720

**Published:** 2024-07-18

**Authors:** Amparo L. Figueroa, Kashif Ali, Gary Berman, Honghong Zhou, Weiping Deng, Wenqin Xu, Stephanie Lussier, Bethany Girard, Frank J. Dutko, Karen Slobod, Anne Yeakey, Frances Priddy, Jacqueline M. Miller, Rituparna Das

**Affiliations:** a Moderna, Inc., Cambridge, MA, USA; b Kool Kids Pediatrics, DM Clinical Research, Houston, TX, USA; c Clinical Research Institute, Minneapolis, MN, USA

**Keywords:** Durability, Safety, Long-term, COVID-19 vaccine, mRNA vaccine, mRNA-1273, Adolescents, Single-dose

## Abstract

**Background:**

Longitudinal changes in vaccination-induced immune response remain inadequately characterized in adolescents. We present long-term safety, immunogenicity, and COVID-19 incidence following a 2-dose mRNA-1273 100-μg primary series, and immunogenicity following a single dose of mRNA-1273 50 μg in vaccine-naïve adolescents.

**Methods:**

TeenCOVE (NCT04649151) Part 1 randomized adolescents (12–17 years) to 2-dose mRNA-1273 100 μg (n = 2490) or placebo (n = 1243) 28 days apart. Subsequently, placebo recipients (n = 91) could receive open-label mRNA-1273. Primary objectives included prespecified adverse events through 12 months; secondary objectives were COVID-19 incidence and neutralizing and spike-binding antibodies (nAbs/bAbs) against SARS-CoV-2 (ancestral/variants) through 12 months (study period: December 2020–January 2022). In Part 2, vaccine-naïve adolescents (n = 52) received up to 2 doses of mRNA-1273 50 μg; interim analysis included Day 28 (D28) nAbs post-injection 1 in SARS-CoV-2-baseline-positive participants (serologic/virologic evidence of prior infection).

**Findings:**

In SARS-CoV-2-baseline-negative adolescents (N = 369), mRNA-1273 induced robust nAb responses versus baseline (geometric mean concentration [GMC] = 11; 95% CI, 11–12) at D28 (1868 [1759–1985]), 6 months (625 [583–670]) and 12 months (550 [490–618]) post-injection 2. Similar bAb responses were observed to alpha/beta/delta/gamma variants; nAb/bAb responses were similar in SARS-CoV-2-baseline-positive adolescents. The 2-dose mRNA-1273 100-μg primary series was generally well-tolerated; one case of nonserious, moderate, probable acute myocarditis resolved by 8 days from symptom onset. A single dose of mRNA-1273 50 μg in SARS-CoV-2-baseline-positive adolescents induced higher D28 nAb GMCs against ancestral SARS-CoV-2 than 2-dose mRNA-1273 100 μg in young adults (geometric mean ratio = 4.322 [3.274-5.707]).

**Interpretation:**

The overall risk–benefit profile of mRNA-1273 remains favorable in adolescents, with durable 12-month immune responses against SARS-CoV-2 (ancestral/variants). A single mRNA-1273 50-μg injection in vaccine-naïve adolescents elicited robust immune responses against SARS-CoV-2.

**Funding:**

This project has been funded in whole or in part with federal funds by the 10.13039/100000016Department of Health and Human Services, United States; 10.13039/100021704Administration for Strategic Preparedness and Response, United States; 10.13039/100012399Biomedical Advanced Research and Development Authority, United States, under Contract No. 75A50120C00034. The findings and conclusions in this report are those of the authors and do not necessarily represent the views of the Department of Health and Human Services or its components.

**Research in context**

**Evidence before this study**

We searched PubMed for published clinical trials on long-term safety and durability of mRNA SARS-CoV-2 primary series vaccine-induced immune responses in adolescents and immunogenicity of a single-dose mRNA SARS-CoV-2 vaccine in previously unvaccinated adolescents with evidence of prior SARS-CoV-2 infection at baseline using the search terms “COVID-19 or SARS-CoV-2” and “mRNA vaccine” and “adolescent” and “clinical trial,” with no restrictions on date or language. Two clinical trials evaluated mRNA-1273 or BNT162b2 COVID-19 vaccines in adolescents and demonstrated short-term safety (within 1 month) and immunogenicity (after 1 month) following two-injections primary series vaccination. One study reported antibody response at 9 months following two injections of BNT162b2 primary series vaccination in adolescents.

**Added value of this study**

To the best of our knowledge, this is among the first studies to demonstrate the long-term safety and immunogenicity of mRNA SARS-CoV-2 vaccines up to 12 months following the completion of the primary series in adolescents aged 12–17 years. mRNA-1273 as a 2-dose 100-μg primary series, was generally well tolerated and the overall risk–benefit profile remains favorable in adolescents. Robust neutralizing antibody levels were observed through 12 months regardless of whether participants tested negative or positive for SARS-CoV-2 infection at baseline. Spike binding antibody responses were also observed against alpha, beta, delta, and gamma variants. Overall, the adverse event profile was typical for this age group, and no unexpected safety concerns were identified. There were no serious adverse events assessed as related to the vaccine, no deaths, and no cases of multisystem inflammatory syndrome. One nonserious adverse event of chest pain was adjudicated by the cardiac event adjudication committee as probable myocarditis. Symptoms resolved after 8 days and a subsequent follow-up cardiology evaluation, which included a physical examination, electrocardiogram, and echocardiogram, showed no abnormalities.

Additionally, we showed that a single 50-μg injection of mRNA-1273 in previously unvaccinated adolescents with evidence of prior SARS-CoV-2 infection induced robust neutralizing antibody responses by 28 days post-injection.

**Implications of all the available evidence**

Our long-term follow-up analysis demonstrated sustained mRNA-1273-induced immune response with durable neutralizing and spike binding antibody levels against SARS-CoV-2 for up to 12 months after the two-injection primary series vaccination. Our findings provide insights into the persistence of immune responses following COVID-19 vaccines, which can inform clinical recommendations on future vaccine doses in adolescents for continued protection against COVID-19. Importantly, the robust immunogenicity of a single 50-μg injection of mRNA-1273 in vaccine-naive SARS-CoV-2-baseline-positive adolescents highlights the benefits of hybrid immunity and provides supportive evidence for a simplified single-dose regimen for populations with high seroprevalence.

## Introduction

SARS-CoV-2 infection can cause severe COVID-19 in children, especially those with underlying medical conditions.^1^^,^^2^ Since the pandemic began, new highly transmissible variants such as omicron have emerged,^3^ with peak levels of COVID-19–related hospitalizations observed among children and adolescents during delta- and omicron-predominant periods.^4^^,^^5^ Notably, during these periods, monthly hospitalization rates were 6- to 10-times higher among unvaccinated versus fully vaccinated adolescents.^4^^,^^5^ mRNA-1273 (SPIKEVAX; Moderna, Inc.), administered as a 2-injection primary series, has an acceptable safety profile in adults and adolescents, with 93.2% and 93.3% efficacy, respectively, against ancestral SARS-CoV-2.^6^, ^7^, ^8^ Understanding the durability of vaccination-induced immune response is important for guiding future vaccination strategies and ensuring ongoing protection against SARS-CoV-2.^9^ However, longitudinal changes in immune response following vaccination in adolescents remain inadequately characterized.^10^, ^11^, ^12^

In the United States, most individuals have immunity to SARS-CoV-2 due to infection or vaccination.^13^^,^^14^ Several observational studies have shown that individuals who have been vaccinated against COVID-19 and had previous SARS-CoV-2 infection are likely to have better protection (termed as hybrid immunity) against the omicron variant, as well as against severe outcomes, including hospitalization, compared with primary series vaccination or infection alone.^15^, ^16^, ^17^, ^18^ Recently, the United States Food and Drug Administration (FDA) and Centers for Disease Control and Prevention (CDC) have simplified the COVID-19 vaccination schedule (2023–2024), recommending a single mRNA vaccine dose for individuals aged 5 years and older, regardless of prior vaccination history.^19^ Data are currently limited for the immunogenicity of a single-dose mRNA vaccine in adolescents with evidence of prior SARS-CoV-2 infection.

Here, we report long-term safety, immunogenicity, and COVID-19 incidence following a 2-dose primary series vaccination of mRNA-1273 100 μg in baseline-SARS-CoV-2-positive and -negative (status determined by serologic and/or virologic evidence of prior SARS-CoV-2 infection at baseline) adolescents aged 12–17 years in the phase 2/3 TeenCOVE trial. In addition, given the high SARS-CoV-2 seroprevalence in the community, we evaluated whether a single 50-μg dose of mRNA-1273 in previously unvaccinated baseline-SARS-CoV-2-positive adolescents, could elicit comparable immune responses to those observed with the 2-dose primary series in baseline-SARS-CoV-2-negative young adults in the COVE study where vaccine efficacy was previously demonstrated.

## Methods

### Study design

TeenCOVE (NCT04649151)^20^ is an ongoing phase 2/3 study that consists of multiple parts. Part 1A was a randomized, observer-blind, placebo-controlled study to evaluate the safety, reactogenicity, and effectiveness of two 100-μg doses of the mRNA-1273 SARS-CoV-2 vaccine in healthy adolescents aged 12–17 years (interim analysis previously reported^8^). Upon the Emergency Use Authorization of a non-study COVID-19 vaccine in adolescents aged ≥12 years in May 2021, the study transitioned to an open-label follow-up (Part 1B) in which participants who originally received placebo were offered mRNA-1273 (Supplementary Figure S1).

Part 2 of TeenCOVE is an open-label study that enrolled 52 previously unvaccinated, healthy US adolescents aged 12–17 years between April and August 2022 to receive a 50-μg primary series of mRNA-1273 (two doses given 28 days apart). Part 2 enrollment was discontinued in August 2022, when the updated variant-containing vaccines were authorized for use as a booster dose (Supplementary Figure S1).

### Study objectives

In Part 1, the primary objectives included inferred effectiveness based on immunogenicity at Day 28 post-injection 2, reactogenicity through 7 days after each injection, and safety through 1 year after two doses of mRNA-1273 100 μg administered 28 days apart. Secondary objectives were to evaluate the persistence of the immune response of two doses of mRNA-1273 100 μg as assessed by SARS-CoV-2 neutralizing antibodies (nAbs) and spike-specific binding antibodies (bAbs) through 1 year after Dose 2 and to evaluate the incidence of symptomatic COVID-19 infection after vaccination with mRNA-1273. Interim analyses (with median duration of follow-up of 53 days from the second injection to the database lock) on immunogenicity at Day 28 post-injection 2, reactogenicity through 7 days and unsolicited adverse events (AEs) through 28 days after each injection, and COVID-19 incidence 14 days post-injection 2 have been published previously^8^ This manuscript reports the prespecified AEs through 1 year (primary objective), and antibody persistence and COVID-19 incidence at 1 year (secondary objectives) following Dose 2 of the mRNA-1273 primary series.

In Part 2, the primary objectives were to evaluate the safety, reactogenicity, and immunogenicity of a two-dose primary series of mRNA-1273 50 μg (doses given 28 days apart). An interim analysis was conducted to describe nAb levels 28 days after 1 dose of mRNA-1273 in vaccine-naïve SARS-CoV-2-positive participants at baseline (determined by positive serology or reverse transcription-polymerase chain reaction [RT-PCR] at pre-injection 1), and to compare them to nAb levels in young adults (18–25 years; baseline SARS-CoV-2-negative) 28 days after the second 100-μg injection of mRNA-1273 primary series (Day 57) in the phase 3 COVE (NCT04470427) study where efficacy was demonstrated.

### Participants

US adolescents (aged 12–17 years) who were considered in good general health by the study investigators were eligible for enrollment. Baseline SARS-CoV-2 status was determined by using virologic and serologic evidence of SARS-CoV-2 infection on or before Day 1, prior to administration of Dose 1 of vaccine. A nasopharyngeal or nasal swab for RT-PCR testing and a blood sample for antibodies against nonvaccine SARS-CoV-2 nucleocapsid protein (by Roche Elecsys) were used for the assessment of SARS-CoV-2 infection.

**Fig. 1:**
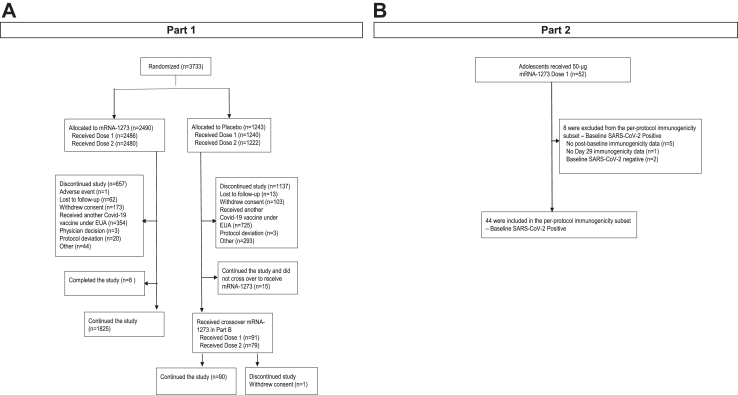
**Trial profile**. EUA, Emergency Use Authorization. Part 1 (A) was a randomized, observer-blind, placebo-controlled study to evaluate the safety, reactogenicity, and effectiveness of two 100-μg doses of mRNA-1273 in healthy adolescents aged 12–17 years; upon the Emergency Use Authorization of a non-study COVID-19 vaccine in adolescents aged ≥12 years, the study transitioned to an open-label follow-up in which participants who originally received placebo were offered the 2-dose primary series of mRNA-1273. Part 2 (B) is an open-label study that enrolled previously unvaccinated, healthy US adolescents aged 12–17 years to receive a 50-μg 2-dose primary series of mRNA-1273. Placebo participants in Part B may have received injection 2 or discontinued from the study after the data cutoff date of 31 January 2022, and therefore, not all participants who received injection 2 or discontinued study vaccine beyond the data cutoff are summarized in the figure.

In Part 1, 3733 participants were randomly assigned 2:1 to two injections of mRNA-1273 100 μg (n = 2490) or placebo (n = 1243), 28 days apart (Fig. 1; Supplementary Figure S2). After authorization of COVID-19 vaccines in adolescents, of the 1222 participants who received two injections of placebo, 91 (7.4%) crossed over and received at least 1 injection of mRNA-1273 (placebo-mRNA-1273 group). In total, 977 participants (80.0%) in the placebo group and 221 participants (8.9%) in the mRNA-1273 group discontinued from the study during Part 1A (most to seek the authorized non-study vaccine).

Overall, the long-term safety set for mRNA-1273 included 2478 participants who received two injections (Supplementary Figure S2), and the long-term immunogenicity set included 383 participants, regardless of their baseline SARS-CoV-2 status determined either by RT-PCR or nucleoside anti-immunoglobulin G antibody. The per-protocol immunogenicity set for long-term analysis included participants who received planned injections of study vaccination per schedule, had no major protocol deviations, complied with immunogenicity testing schedule, and were SARS-CoV-2 negative at baseline (n = 369).

At the time of enrollment discontinuation in August 2022, a total of 52 adolescents were enrolled in Part 2 and had received at least 1 dose of 50 μg of mRNA-1273.

### Immunogenicity assessments

In Part 1, blood samples for determining SARS-CoV-2-specific antibody responses were collected on Days 1, 57 (28 days after injection 2), 209 (6 months after injection 2), and 394 (1 year after injection 2). SARS-CoV-2 nAbs against SARS-CoV-2 (D614G) were measured using a validated pseudovirus neutralization assay (VAC62) with 50% inhibitory concentration as the primary endpoint. bAbs specific for the SARS-CoV-2 spike protein for the alpha, beta, delta, and gamma variants and ancestral SARS-CoV-2 (D614G) were measured with the use of a Meso-Scale Discovery assay (VAC113). Immunogenicity assays are described in the Supplementary Appendix. In Part 2, blood samples were collected 28 days after the first 50-μg injection of mRNA-1273 and immunogenicity was assessed by measuring nAb levels against the ancestral SARS-CoV-2.

### Safety assessments

The interim results of the blinded data from the mRNA-1273 and placebo group were previously reported.^8^ Upon the Emergency Use Authorization of a non-study COVID-19 vaccine in adolescents, unblinding and attrition of participants from the placebo group occurred; thus, 31 May 2021 was considered to be the end of the comparative blinded portion of the study.

Long-term safety analysis (Part 1A and Part 1B) consisted of cumulative long-term data (data cutoff date of 31 January 2022) for all participants from the time they received a dose of mRNA-1273 and included serious adverse events (SAEs), adverse events of special interest (AESIs), AEs leading to discontinuation, and medically attended adverse events (MAAEs) throughout the study.

An independent cardiac event adjudication committee (CEAC) reviewed suspected cases of myocarditis and pericarditis to determine if they met Centers for Disease Control and Prevention (CDC) criteria of “probable” or “confirmed” events (see Supplementary Appendix for details).^21^

### Long-term analysis of incidence rate of COVID-19

After transitioning to the open-label phase, COVID-19 cases were continued to be monitored. The long-term incidence rates (1 January 2021 through 31 January 2022) of symptomatic COVID-19 among mRNA-1273 recipients who remained on study until the data cut-off date of 31 January 2022 are presented. The diagnosis of COVID-19 was determined using the primary case definition followed in the phase 3 COVE trial, which required ≥2 systemic symptoms or ≥1 respiratory symptom AND a positive RT-PCR test (Supplementary Appendix).^6^

### Statistical analysis

Long-term analyses of safety, immunogenicity, and COVID-19 incidence endpoints were summarized descriptively by treatment without comparing the placebo-mRNA-1273 and mRNA-1273 groups. Incidence rates of COVID-19 infections were analyzed starting 14 days after the second injection of mRNA-1273 in Parts 1A and 1B in the per-protocol efficacy set (Supplementary Figure S2) with 95% confidence intervals (CIs) calculated using the exact method (Poisson distribution) and adjusted for person-time. Incidence rates were also analyzed by calendar month.

Immunogenicity analyses were assessed for both the immunogenicity subset (baseline SARS-CoV-2-positive and -negative participants as determined by serologic/virologic evidence of prior SARS-CoV-2 infection at baseline) and the per-protocol immunogenicity subset (baseline SARS-CoV-2 negative participants). In Part 1, nAb and bAb levels were summarized over time at specified time points (Days 1 [pre-injection], 57 [28 days post-injection 2], 209 [6 months post-injection 2], and 394 [1-year post-injection 2]). In Part 2, nAb GMC levels at Day 1 (pre-injection) and Day 29 (28 days post-injection 1) were summarized.

The GMCs (for nAbs) or geometric mean (GM, for bAbs) of SARS-CoV-2–specific antibodies with corresponding 95% CIs calculated based on the *t* distribution of the log-transformed values then back-transformed to the original scale for presentation were provided at each time point. The geometric mean fold rise (GMFR) of SARS-CoV-2–specific antibody levels from pre-injection (baseline) at Day 1 with corresponding 95% CIs calculated based on the *t* distribution of the log-transformed values then back-transformed to the original scale for presentation were provided at each post-baseline time point. Seroresponse was defined as an antibody value change from baseline (pre-injection 1) below the lower limit of quantitation (LLOQ) to ≥ 4 × LLOQ, or at least a 4-fold rise if baseline is ≥ LLOQ. Number and percentage of participants with seroresponse after vaccination (seroresonse rate [SRR]) were provided with two-sided 95% CIs (Clopper-Pearson method) at each postbaseline timepoint.

For Part 2, the geometric mean ratio defined as the ratio of the GMC value against the ancestral strain post-injection 1 of mRNA-1273 50 μg in the current study over the GMC value against the ancestral strain post-injection 2 of mRNA-1273 100 μg (Day 57) in young adult participants (18–25 years) of COVE study, was calculated. The difference between the adolescent SRR (post-injection 1 relative to pre-injection 1) in the current study and the young adult SRR (post-injection 2 relative to pre-injection 1) in the COVE study was also measured. Due to the smaller number of participants enrolled in Part 2 (N = 52) than the originally intended 362 participants, the initial plan for immunogenicity hypothesis testing (i.e., immunobridging to young adults from the COVE trial) was revised to a descriptive analysis.

### Ethics statement

The study was conducted according to the study protocol, applicable laws and regulatory requirements, and the ethical principles outlined by the Declaration of Helsinki and the Council for International Organizations of Medical Sciences International Ethical guidelines, all Good Clinical Practice guidelines of the International Council for Harmonisation, and all applicable regulatory requirements. The study protocol and any amendments were approved by the institutional review board prior to study initiation. All participants provided written informed consent.

### Role of the funding source

This project has been supported in whole or in part with federal funds by the Department of Health and Human Services; Administration for Strategic Preparedness and Response; Biomedical Advanced Research and Development Authority (BARDA), under contract 75A50120C00034. The findings and conclusions in this report are those of the authors and do not necessarily represent the views of the Department of Health and Human Services or its components. BARDA was involved in the study design. The trial sponsor, Moderna, was responsible for the overall trial design, site selection and monitoring, and data analysis. Investigators were responsible for data collection. Two medical writers funded by Moderna assisted in drafting the manuscript for submission.

## Results

### Trial population

In the long-term analysis, 2486 participants who originally received mRNA-1273 in Part 1A had a median follow-up of 312 days after the second injection. The 91 participants in Part 1B (placebo-mRNA-1273 group) had a median follow-up of 71 days after the second injection of mRNA-1273 (Fig. 1; Supplementary Figure S2).

Demographics and baseline characteristics in the long-term analysis safety set were comparable between the placebo-mRNA-1273 and mRNA-1273 groups (Table 1). The mean ages at screening in the mRNA-1273 and placebo-mRNA-1273 groups were 14.3 and 14.1 years, 51.6% [1283/2486] and 52.7% [48/91] were male; and 83.8% (2084/2486) and 90.1% (82/91) were White, respectively. However, 39.6% of the placebo-mRNA-1273 recipients (36/91) were positive for SARS-CoV-2 infection pre-injection 1 compared with 5.9% (147/2486) of the mRNA-1273 recipients (147/2486).

**Table 1:** Baseline demographics – Part 1, long-term analysis phase (safety set; immunogenicity subset; per-protocol immunogenicity subset).

Characteristics	Part 1, safety set^a^		Part 1, immunogenicity subset^b^	Part 1, per-protocol immunogenicity subset^c^
	Placebo-mRNA-1273 (n = 91)	Two injections mRNA-1273 100 μg (n = 2486)	Two injections mRNA-1273 100 μg (n = 383)	Two injections mRNA-1273 100 μg (n = 369)
Age at screening, years				
Mean (range)	14.1 (12–17)	14.3 (12–17)	14.0 (12–17)	14.0 (12–17)
Age category at screening, n (%)				
≥12 and < 16 years	70 (76.9)	1839 (74.0)	314 (82.0)	303 (82.1)
≥16 and < 18 years	21 (23.1)	647 (26.0)	69 (18.0)	66 (17.9)
Sex, n (%)				
Male	48 (52.7)	1283 (51.6)	187 (48.8)	179 (48.5)
Female	43 (47.3)	1203 (48.4)	196 (51.2)	190 (51.5)
Body mass index, median (range), kg/m^2^	20.86 (15.1–38.2)	21.13 (11.0–76.7)	20.74 (15.2–76.7)	20.82 (15.2–76.7)
Race, n (%)				
White	82 (90.1)	2084 (83.8)	329 (85.9)	317 (85.9)
Black	2 (2.2)	83 (3.3)	6 (1.6)	5 (1.4)
Asian	3 (3.3)	142 (5.7)	17 (4.4)	17 (4.6)
Multiracial	3 (3.3)	118 (4.7)	25 (6.5)	24 (6.5)
American Indian or Alaska Native	1 (1.1)	12 (0.5)	1 (0.3)	1 (0.3)
Native Hawaiian or Other Pacific Islander	0	3 (0.1)	0	0
Other, not reported, or Unknown	0	44 (1.8)	5 (1.3)	5 (1.4)
Ethnicity, n (%)				
Hispanic or Latinx	23 (25.3)	280 (11.3)	46 (12.0)	44 (11.9)
Not Hispanic or Latinx	68 (74.7)	2186 (87.9)	335 (87.5)	323 (87.5)
Not reported or Unknown	0	20 (0.8)	2 (0.5)	2 (0.5)
Pre-injection 1 RT-PCR result, n (%)				
Negative	83 (91.2)	2311 (93.0)	381 (99.5)	369 (100.0)
Positive	3 (3.3)	13 (0.5)	2 (0.5)	0
Missing	5 (5.5)	162 (6.5)	0	0
Pre-injection 1 anti-SARS-CoV-2 nucleocapsid assay result, n (%)				
Negative	53 (58.2)	2304 (92.7)	371 (96.9)	369 (100.0)
Positive	34 (37.4)	139 (5.6)	12 (3.1)	0
Missing	4 (4.4)	43 (1.7)	0	0
Pre-injection 1 SARS-CoV-2 infection status,^d^ n (%)				
Negative	51 (56.0)	2171 (87.3)	369 (96.3)	369 (100.0)
Positive	36 (39.6)	147 (5.9)	14 (3.7)	0
Missing	4 (4.4)	168 (6.8)	0	0
SARS-CoV-2 infection status at or before Day 394, n (%)				
Positive	NA	NA	76 (19.8)	64 (17.3)
Negative	NA	NA	307 (80.2)	305 (82.7)

NA = not applicable; RT-PCR = reverse transcription-polymerase chain reaction.

a The safety set includes all randomly assigned participants who received ≥1 dose of placebo or mRNA-1273.

b The immunogenicity subset is a subset of participants who received ≥1 dose of mRNA-1273 that was selected for immunogenicity testing.

c The per-protocol immunogenicity subset includes participants in the immunogenicity subset who received planned injections of study vaccination per schedule, complied with immunogenicity testing schedule, and have no major protocol deviations that impact key or critical data. Participants who were seropositive at baseline were excluded from the per-protocol immunogenicity subset.

d Pre-injection 1 SARS-CoV-2 status was positive if there was evidence of previous SARS-CoV-2 infection, defined as positive binding antibody against the SARS-CoV-2 nucleocapsid or positive RT-PCR assay prior to administration of mRNA-1273 injection 1; negative SARS-CoV-2 status was defined as negative binding antibody against the SARS-CoV-2 nucleocapsid and a negative RT-PCR assay prior to administration of mRNA-1273 injection 1. mRNA-1273 group received mRNA-1273 vaccine between December 2020 and February 2021; placebo-mRNA-1273 crossover participants received the mRNA-1273 primary series starting from October 2021.

In Part 2, 52 SARS-CoV-2-baseline-positive adolescent participants were enrolled and received up to 2 doses of mRNA-1273 50 μg. The baseline characteristics of adolescents in this study and young adults in the phase 3 COVE study are shown in Supplementary Table S1. The mean age was 14 years for adolescents in Study P203 and 22 years for young adults in Study P301. The proportions of male and female participants were comparable in the two studies.

### Immunogenicity

**Fig. 2:**
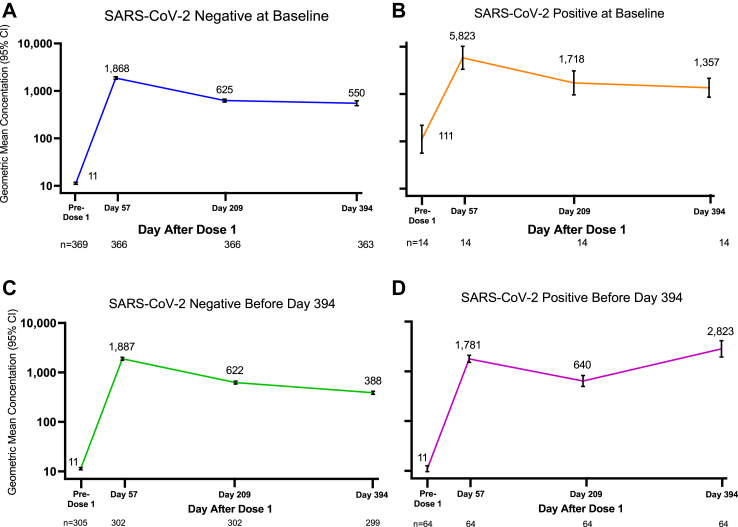
**Long-term analysis of pseudovirus neutralizing antibody values by baseline SARS-CoV-2 status and by SARS-CoV-2 infection status at or before Day 394**. CI, confidence interval; n, number of participants with non-missing data at the time point. Pseudovirus neutralizing antibody geometric mean concentrations in the VAC62 assay at Day 0 (pre-injection 1) and 57, 209, and 394 days after injection 1 are provided for participants in the per-protocol immunogenicity set negative for SARS-CoV-2 at baseline (A), those positive for SARS-CoV-2 at baseline (B), those negative for SARS-CoV-2 at baseline and negative for SARS-CoV-2 post-baseline at or before Day 394 (C), and those negative for SARS-CoV-2 at baseline and positive for SARS-CoV-2 post-baseline at or before Day 394 (D). 95% CIs were calculated based on the *t* distribution of the log-transformed values of the geometric mean concentrations and then back-transformed to the original scale for presentation as black brackets.

As previously reported, the blinded analysis demonstrated robust nAb titers in SARS-CoV-2 baseline-negative adolescents aged 12–17 years, which were noninferior to those in baseline-negative participants aged 18–25 years at 28 days post-injection 2 (Day 57).^8^ At 28 days post-injection 2, mRNA-1273 induced robust nAb levels in SARS-CoV-2 baseline-negative adolescents (N = 369; GMC = 1868; 95% CI, 1759–1985 vs baseline, GMC = 11; 95% CI, 11–12) (Table 2; Fig. 2A). The GMFR in nAb levels compared with pre-injection 1 was 166 (95% CI, 154–180). The SRR was 100% (95% CI, 99.0–100.0%) at Day 57. At 6 and 12 months post-injection 2, nAb responses had declined but remained markedly higher than baseline levels. The GMFR in nAbs at 6 months post-injection 2 (Day 209) compared to baseline (pre-injection 1) remained 55-fold higher, with a GMC of 625 (95% CI, 583–670). Relatively little additional decline was observed between 6 and 12 months after injection 2 (Day 394 GMC = 550; 95% CI, 490–618; 49-fold higher than the baseline GMC). The SRR was maintained at 100% (95% CI, 99.0–100.0%) at 6 and 12 months. nAb responses were also maintained in participants with no SARS-CoV-2 infection through Day 394 (GMC at Day 209 = 622; 95% CI, 582–665) and persisted through Day 394 (388; 95% CI, 362–415) (Supplementary Table S2; Fig. 2C).

**Table 2:** Long-term analysis of pseudovirus neutralizing antibody levels against ancestral SARS-CoV-2 (D614G) and seroresponse rates following two injections of mRNA-1273 100 μg in adolescents aged 12–17 years (Part 1).

Time point	All participants (N = 383)	SARS-CoV-2 baseline-negative (n = 369)	SARS-CoV-2 baseline-positive (n = 14)
Baseline (pre-injection 1), Day 1, n^a^	383	369	14
Geometric mean concentration (95% CI)^b^	12 (11–13)	11 (11–12)	111 (56–219)
Day 57, n	380	366	14
Geometric mean concentration (95% CI)	1948 (1826–2078)	1868 (1759–1985)	5823 (3323–10206)
Geometric mean fold rise (95% CI)^c^	159 (147–173)	166 (154–180)	53 (32–86)
Seroresponse rate, n/N1, % (95% CI)^d^	380/380, 100% (99.9–100.0%)	366/366, 100% (99.0–100.0%)	14/14, 100% (76.8–100.0%)
Day 209, n	380	366	14
Geometric mean concentration (95% CI)	649 (604–698)	625 (583–670)	1718 (960–3073)
Geometric mean fold rise (95% CI)	53 (48–58)	55 (51–60)	16 (9–26)
Seroresponse rate, n/N1, % (95% CI)	379/380, 99.7% (98.5–100.0%)	366/366, 100% (99.0–100.0%)	13/14, 92.9% (66.1–99.8%)
Day 394, n	377	363	14
Geometric mean concentration (95% CI)	569 (508–638)	550 (490–618)	1357 (857–2149)
Geometric mean fold rise (95% CI)	46 (41–53)	49 (43–56)	12 (7–20)
Seroresponse rate, n/N1, % (95% CI)	376/377, 99.7% (98.5–100.0%)	363/363, 100% (99.0–100.0%)	13/14, 92.9% (66.1–99.8%)

CI = confidence interval; LLOQ = lower limit of quantitation; N1 = number of participants with non-missing data at baseline (pre-injection 1, ie, Day 1), and the corresponding post-Day 1 time point; RT-PCR = reverse transcriptase-polymerase chain reaction; ULOQ = upper limit of quantitation.

Pre-injection 1 SARS-CoV-2 status was positive if there was evidence of previous SARS-CoV-2 infection, defined as positive binding antibody against the SARS-CoV-2 nucleocapsid or positive RT-PCR assay at Day 1; negative SARS-CoV-2 status was defined as negative binding antibody against the SARS-CoV-2 nucleocapsid and a negative RT-PCR assay at Day 1. Antibody values reported as below the LLOQ (10 for ancestral SARS-CoV-2 [D614G]) were replaced by 0.5 x LLOQ. Values reported as greater than the ULOQ (281,600 for Part 1) were replaced by the ULOQ if actual values were not available.

a Number of participants with non-missing data at the time point.

b Geometric mean concentrations of pseudovirus neutralizing antibodies to SARS-CoV-2 (D614G) were determined in the VAC62 assay. 95% CIs were calculated based on the *t*-distribution of the log-transformed values of the geometric mean concentrations and then back-transformed to the original scale for presentation.

c Geometric mean fold rises in geometric mean concentrations of neutralizing antibodies are relative to Day 1. 95% CIs were calculated based on the *t*-distribution of the log-transformed values of the geometric mean concentrations and then back-transformed to the original scale for presentation.

d Seroresponse rates are relative to pre-injection 1. Seroresponse is defined as a change from below the LLOQ to equal or higher than 4 x LLOQ, or at least a 4-fold rise if baseline is equal to or above the LLOQ. Percentages are based on N1. 95% CIs are calculated using the Clopper-Pearson method.

Robust nAb responses, higher than those in SARS CoV-2 baseline-negative participants, were observed in participants who were SARS-CoV-2 baseline-positive, with a GMC of 5823 (95% CI, 3323–10206) at 1-month post-injection 2, a GMC of 1718 (960–3073) at 6 months post-injection 2, and a GMC of 1357 (857–2149) at 12 months post-injection 2 (Table 2; Fig. 2B). nAb responses were 53-fold higher than baseline at 1 month (GMFR, 53; 32–86), 16-fold higher (9–26) at 6 months, and 12-fold higher (7–20) at 12 months post-injection 2. Persistent SRRs were also observed: 100% at 1 month (76.8%–100.0%), 92.9% at 6 months (66.1%–99.8%), and 92.9% at 12 months (66.1%–99.8%) post-injection 2. Similar results were observed for bAb levels to ancestral SARS-CoV-2 as well as to the alpha, beta, delta, and gamma variants (Supplementary Figure S3).

At baseline, the nAb GMC in previously unvaccinated SARS-CoV-2 baseline-positive participants in Part 2 was 361 (95% CI, 242–541), which was higher than that of SARS-CoV-2 baseline-negative young adults in the phase 3 COVE study (GMC = 11; 11–12) (Table 3). After 28 days following injection 1 of 50-μg mRNA-1273, the GMC levels in adolescent baseline-positive participants (GMC = 6053; 95% CI, 4496–8150) were 18-fold higher than baseline GMC levels. The GMC post-injection 1 was higher than that observed following the primary series in SARS-CoV-2 baseline-negative young adults in the COVE study (GMC = 1400; 95% CI, 1273–1541), with a geometric mean ratio of 4.3 (95% CI, 3.3–5.7). The SRRs were comparable between the P203 adolescents (97.5%; n = 39/40) and the P301 young adults (99.3%; n = 292/294), with an SRR difference of −1.8% (95% CI, −12.2% to 0.8%). The immune response after the administration of the second injection of mRNA-1273 50 μg was comparable to the response following the first injection of mRNA-1273 50 μg (GMC [95% CI] = 7614 [5818–9970] versus 6053 [4496–8150]).

**Table 3:** Comparison of pseudovirus neutralizing antibody levels against ancestral SARS-CoV-2 (D614G) and seroresponse rates following one injection of mRNA-1273 50 μg in adolescents aged 12–17 years and two injections of mRNA-1273 in young adults aged 18–25 years (Part 2).

One injection, mRNA-1273 50 μg Baseline SARS-CoV-2 positive P203 Part 2 adolescents (12–17 years) (n = 44)		Two injections, mRNA-1273 100 μg Baseline SARS-CoV-2 negative P301 young adults (18–25 years) (n = 296)	
Baseline (pre-injection 1), Day 1, n	43	Baseline (pre-injection 1), Day 1, n	295
GMC (95% CI)	361 (242, 541)	GMC (95% CI)	11 (11, 12)
Day 29, n	41	Day 57, n	294
GMC (95% CI)	6053 (4496, 8150)	GMC	1400 (1273, 1541)
GMFR, (95% CI)	18 (12, 27)	GMFR	127 (114, 141)
Seroresponse rate, n/total n, % (95% CI)	39/40, 97.5% (86.8–99.9%)	Seroresponse rate, n/total n, % (95% CI)	292/294, 99.3% (97.6–99.9%)
GMR (adolescents vs young adults), (95% CI)	4.3 (3.3–5.7)		
Seroresponse rate difference (adolescents vs young adults), % (95% CI)	−1.8 (−12.2 to 0.8)		

CI = confidence interval; GMC = geometric mean concentration; GMFR = geometric mean fold rise; GMR = geometric mean ratio; LLOQ = lower limit of quantitation; N1 = number of participants with non-missing data at baseline (pre-injection 1, ie, Day 1), and the corresponding post-Day 1 time point; RT-PCR = reverse transcriptase-polymerase chain reaction; ULOQ = upper limit of quantitation.

Baseline SARS-CoV-2 status was positive if there was evidence of previous SARS-CoV-2 infection, defined as positive binding antibody against the SARS-CoV-2 nucleocapsid or positive RT-PCR assay at Day 1; negative SARS-CoV-2 status was defined as negative binding antibody against the SARS-CoV-2 nucleocapsid and a negative RT-PCR assay at Day 1. Antibody values reported as below the LLOQ (10 for ancestral SARS-CoV-2 [D614G]) were replaced by 0.5 x LLOQ. Values reported as greater than the ULOQ (111,433 for Part 2) were replaced by the ULOQ if actual values were not available.

### Safety

The reactogenicity in the blinded Part 1A of the study as of 8 May 2021 was previously reported.^8^ In the long-term analysis from injection 1 through Day 394, there were no SAEs assessed as related to the vaccine by the investigator, no deaths, and no cases of multisystem inflammatory syndrome in children (Table 4). The AE profile was typical for this age group, and no new safety concerns were identified. The most commonly reported MAAE was COVID-19. The increased rates of COVID-19 likely reflect the omicron wave. The most commons AESIs were anosmia and/or ageusia; no AESIs were considered vaccine-related by the investigators. One nonserious AE of chest pain was adjudicated by the CEAC as probable myocarditis. Symptoms resolved after 8 days; a subsequent follow-up cardiology evaluation, which included a physical examination, electrocardiogram, and echocardiogram, showed no abnormalities (See Supplementary Appendix for more details).

**Table 4:** Unsolicited adverse events (long-term analysis safety set).

Unsolicited adverse event^a^	Regardless of relationship to study vaccination (total; N = 2577)^b^	Related to study vaccination (total; N = 2577)^b^
All	1427 (55.4)	391 (15.2)
Serious	22 (0.9)	0
Fatal	0	0
Medically attended	1014 (39.3)	29 (1.1)
Leading to study vaccine discontinuation	3 (0.1)	1 (<0.1)
Leading to study discontinuation	0	0
Severe	50 (1.9)	15 (0.6)
AESI		
MIS-C	0	0
Other	13 (0.5)	0

AESI = adverse event of special interest; MIS-C = multisystem inflammatory syndrome in children.

a A treatment-emergent adverse event is defined as any event not present before exposure to study vaccination or any event already present that worsens in intensity or frequency after exposure to study vaccination. The median follow-up was 71 days for the participants who initially received placebo and then crossed over to receive mRNA-1273, and 312 days for the participants who received mRNA-1273.

b Total participants include 2486 who received mRNA-1273 and 91 who initially received placebo and then crossed over to receive mRNA-1273.

### Incidence rate of COVID-19

The incidence rate of COVID-19 after administration of the primary series of mRNA-1273 in the per-protocol efficacy set was low (0.0–3.53 cases per 1000 person-months) from January to November 2021 (Supplementary Table S3). The incidence rates of COVID-19 increased in December 2021 (23.4 cases per 1000 person-months) and January 2022 (92.6 cases per 1000 person-months), corresponding with the initial omicron wave.

## Discussion

Previously, we demonstrated mRNA-1273 vaccine efficacy in the pivotal efficacy trial in adults^6^^,^^7^ and vaccine effectiveness in younger groups was inferred through immunobridging of adolescent and pediatric antibody responses to those in adults.^8^^,^^22^^,^^23^ In this study, we provide evidence of the durability of mRNA-1273 immune responses in adolescents aged 12–17 years for up to 12 months as shown by the robust nAb levels in participants who were negative for SARS-CoV-2 infection throughout the study. In addition, robust immune responses following the primary series of mRNA-1273 100 μg were observed in participants who were positive for SARS-CoV-2-infection at baseline. Importantly, a single 50-μg injection of mRNA-1273 in previously unvaccinated adolescents with evidence of prior SARS-CoV-2 infection induced robust nAb responses against ancestral SARS-CoV-2. Taken together, these data also highlight the benefits of hybrid immunity and demonstrate immunogenicity of a single 50-μg injection of mRNA-1273 (albeit derived from a limited participant cohort [n = 41]) in populations with high seroprevalence, thereby supporting the recent recommendation (2023–2024) by the US Food and Drug Administration for a single-dose regimen to simplify vaccination schedule for most individuals.^24^

This study is one of the first to demonstrate long-term immunogenicity up to 12 months following the completion of the mRNA-1273 primary series. The KidCOVE study (two injections of mRNA-1273 50 μg administered 28 days apart) also demonstrated the persistence of immune response up to 12 months in the pediatric age group (aged 6–11 years).^25^ In the phase 3 pivotal P301 COVE study in adults, the durability of vaccine-induced responses was demonstrated for up to 6 months after the second injection.^26^ Another mRNA-based vaccine has also demonstrated antibody responses up to 9 months following completion of a two-injection primary series vaccination.^10^, ^11^, ^12^ Our data also provide further evidence of the safety of mRNA-1273 in adolescents. One AE of chest pain was adjudicated as probable myocarditis; this event was mild and self-limited, with no evidence of sequelae, which is consistent with most cases of myocarditis post-vaccination. The safety profile of mRNA-1273 has been well-characterized in extensive post-marketing data analyses^27^, ^28^, ^29^ and in clinical studies in adults.^6^^,^^7^

While decreases in antibody levels were observed compared with Day 57 (1 month after the second injection) in our long-term analysis, the magnitude of the nAb response at 6 months was maintained up to 1 year from the second injection. Similar results were observed in bAb levels, including responses to the alpha, beta, delta, and gamma variants, which suggests the breadth of antibody responses against these viral variants. Indeed, our data showed low COVID-19 incidence rates from January 2021 through November 2021, during which time these variants were circulating. However, sharp increases in COVID-19 incidence rates were observed in December 2021 and January 2022, which coincides with the circulation of omicron variants. In a separate analysis in a small subset of adolescent participants who received a two-injection primary series of mRNA-1273, the nAb levels 28 days after the second injection were 11.8-fold lower for omicron BA.1 than against ancestral SARS-CoV-2 (D614G).^30^

The ongoing genetic evolution of SARS-CoV-2 variants^31^^,^^32^ highlights the need to develop vaccine formulations that provide broader immunity and further enhance clinical effectiveness against circulating variants. Our data provide further evidence for the overall benefits of mRNA-1273 vaccination, including the long-term safety and durability of antibody responses. Given that variant-containing vaccines include a more closely matched strain to the circulating variant, the benefit observed for the original vaccine is likely to extend to variant-containing vaccines as well. Continued monitoring of neutralization and vaccine effectiveness of mRNA variant-containing vaccine formulations against future emerging variants is needed in the future development of vaccination strategies against COVID-19.

This trial has several limitations. The large number of discontinuations in the placebo group prevented the determination of long-term vaccine efficacy. While the study showed an increase in bAb against alpha, beta, delta, and gamma variants, nAb levels against these variants were not determined in this study. However, previous studies have demonstrated a strong correlation between bAb and nAb levels,^33^, ^34^, ^35^ including those induced by mRNA-1273 vaccination.^36^ Furthermore, nAb levels against omicron variants were not determined in this study. However, mRNA-1273 primary series has been shown to elicit nAbs against omicron variant (although lower than against ancestral strain) at 1 month post-vaccination in adolescents and children.^30^ To our knowledge, there is currently no available data on the long-term durability of nAbs against omicron variant. A study evaluating an omicron-containing bivalent booster in vaccine-naïve adolescents is ongoing.

In conclusion, this long-term follow-up analysis shows that the overall risk–benefit profile of mRNA-1273 remains favorable in adolescents aged 12–17 years. The immunogenicity of mRNA-1273 demonstrated durable nAb and spike bAb levels for up to 1 year after the two-injection primary series against SARS-CoV-2 and durable spike bAb levels against several variants. In addition, a single 50-μg injection of mRNA-1273 in vaccine-naive baseline-positive adolescents was highly immunogenic, which supports a simplified single-dose regimen. The emergence of highly transmissible variants in adolescents warrants continued and uninterrupted access to COVID-19 vaccines in this population.

## Contributors

A Figueroa, J Miller, R Das, W Deng, K Slobod, H Zhou, and K Ali were involved in the study concept and design. Data collection was performed by A Figueroa, A Yeakey, J Miller, W Xu, B Girard, R Das, G Berman, W Deng, H Zhou, and K Ali. Analysis and interpretation of the data were undertaken by A Yeakey, K Slobod, A Figueroa, J Miller, W Xu, S Lussier, R Das, G Berman, F Dutko, W Deng, H Zhou, and K Ali. All authors contributed to the drafting and critical review of this manuscript and approved the final draft. A Figueroa, H Zhou, W Deng, W Xu, S Lussier, B Girard, F Dutko, K Slobod, A Yeakey, F Priddy, J Miller, and R Das have directly accessed and verified the underlying data reported in the manuscript. A Figueroa verifies that authors had full access to the data in the study and accept responsibility to submit for publication.

## Data sharing statement

As the trial is ongoing, access to patient-level data and supporting clinical documents with qualified external researchers may be available upon request and subject to review once the trial is complete.

## Declaration of interests

A Figueroa, H Zhou, W Deng, W Xu, S Lussier, B Girard, F Dutko, F Priddy, J Miller, and R Das are employees of and shareholders in Moderna, Inc. K Ali has nothing to disclose. G Berman received vaccine clinical research payments for work completed for Moderna, Inc. A Yeakey and K Slobod are consultants and were contracted by Moderna Inc. for this study.

## Appendix A Supplementary data

Supplementary Appendix Tables and Figures

mRNA-1273-P203-protocol amendment 6-final

Moderna mRNA-1273-P203 SAP v5.0_Part 1A

Moderna mRNA-1273-P203 SAP v5.0_Part 1B+1C+2+3
