# Supplementary material for: Safety and durability of mRNA-1273–induced SARS-CoV-2 immune responses in adolescents: results from the phase 2/3 TeenCOVE trial
Source: eClinicalMedicine. 2024 Jul 18;74:102720. doi: 10.1016/j.eclinm.2024.102720 (PMC11293523; doi:10.1016/j.eclinm.2024.102720)
Supplement: Supplementary Appendix Tables and Figures [file mmc1.docx]

**Supplementary appendix**

Supplement to: Figueroa AL, Ali K, Berman G, et al. Safety and durability of mRNA-1273–induced SARS-CoV-2 immune responses in adolescents: Results from the phase 2/3 TeenCOVE trial

**SUPPLEMENTARY APPENDIX**

Contents

[1 List of Investigators 3](#_Toc169128116)

[2 Supplementary Methods 4](#_Toc169128117)

[2.1 Safety assessments 4](#_Toc169128118)

[2.2 Immunogenicity assays 4](#_Toc169128119)

[2.3 Diagnosis of COVID-19 4](#_Toc169128120)

[2.4 Study eligibility criteria (Part 1B) 4](#_Toc169128121)

[2.5 Trial vaccine 5](#_Toc169128122)

[3 Supplementary Results 6](#_Toc169128123)

[3.1 Safety 6](#_Toc169128124)

[4 Supplementary Figures 7](#_Toc169128125)

[4.1 Supplementary Figure 1. Study design (Parts 1A, 1B, Part 2) 7](#_Toc169128126)

[4.2 Supplementary Figure 2. Part 1 analysis populations 8](#_Toc169128127)

[4.3 Supplementary Figure 3. Spike-binding antibody levels and seroresponse rates for variants and ancestral SARS-CoV-2 (long-term analysis) 9](#_Toc169128128)

[5 Supplementary Tables 10](#_Toc169128129)

[5.1 Supplementary Table 1. Baseline demographics – Part 2 10](#_Toc169128130)

[5.2 Supplementary Table 2. Pseudovirus neutralizing antibody levels and seroresponse rates for ancestral SARS-CoV-2 (D614G) by SARS-CoV-2 infection status at or before Day 394 (long-term analysis) 12](#_Toc169128131)

[5.3 Supplementary Table 3. Long-term analysis of incidence rates of COVID-19 (phase 3 COVE case definition) in the mRNA-1273 group (combined blinded and open-label phases) 14](#_Toc169128132)

# List of Investigators

| **Investigator** | **Institution** | **Location** |
| --- | --- | --- |
| Olutola Adetona | Tekton Research - Texas - Platinum | San Antonio, Texas |
| Adebayo Akinsola | Tekton Research - Georgia - Platinum | Chamblee, Georgia |
| Kashif Ali | DM Clinical Research - Kool Kids Pediatrics | Houston, Texas |
| Madhavi Ampajwala | ACRC Trials - Hunt | Plano, Texas |
| Gary Berman | Clinical Research Institute, Inc | Minneapolis, Minnesota |
| Gary Boone | Paradigm Clinical Research | La Mesa, California |
| Robert Buynak | Velocity Clinical Research - Valparaiso | Valparaiso, Indiana |
| Quito Carr | MedPharmics, LLC. - Albuquerque | Albuquerque, New Mexico |
| Laurence Chu | Benchmark Research - Austin | Austin, Texas |
| Robert Clifford | Coastal Pediatric Associates | Charleston, South Carolina |
| Salma Elfaki | Accel Research Sites - Nona Pediatric Center | Orlando, Florida |
| Kenneth Etokhana | Tekton Research - Texas - Platinum | San Antonio, Texas |
| Carlos Fierro | Johnson County Clin-Trials | Lenexa, Kansas |
| Carl Griffin | Lynn Health Science Institute | Oklahoma City, Oklahoma |
| Robert Jeanfreau | MedPharmics - Platinum | Metairie, Louisiana |
| Judith Kirstein | Velocity Clinical Research - Banning | West Jordan, Utah |
| Paul Matherne | MedPharmics - Platinum | Gulfport, Mississippi |
| Richard Ohnmacht | Velocity Clinical Research - Providence | Warwick, Rhode Island |
| Paul Pickrell | Tekton Research - Texas - Platinum | Austin, Texas |
| Richard Powell | Velocity Clinical Research - Cincinnati | Cincinnati, Ohio |
| Celia Reyes-Acuna | Crossroads Clinical Research (Victoria) | Corpus Christi, Texas |
| Barbara Rizzardi | Velocity Clinical Research - Salt Lake City - Jordan Valley | West Jordan, Utah |
| Katherine Ruiz-de-Luzuriaga | University of Massachusetts Medical School | Worcester, Massachusetts |
| Nathan Segall | Clinical Research Atlanta | Stockbridge, Georgia |
| Mark Turner | Velocity Clinical Research - Boise | Meridiani Idaho |
| Leonard Weiner | Child Healthcare Associates - East Syracuse | Syracuse, New York |

# Supplementary Methods

## Safety assessments

The study incorporated enhanced surveillance for possible unrecognized myocarditis/pericarditis cases. The script used in routine safety calls was modified to specifically query for symptoms associated with myocarditis/pericarditis. Further, the safety dataset was interrogated (i) for all AEs included in the narrow and broad cardiomyopathy Standardized MedDRA Queries and (ii) using an algorithm generated based on the Centers for Disease Control and Prevention working case definitions of acute myocarditis/pericarditis.^1^

Solicited adverse reactions and unsolicited adverse events within 28 days after any injection were not collected from placebo-mRNA-1273 participants vaccinated in Part 1B of the study unless they met the criteria for a medically attended adverse event, serious adverse event, adverse event leading to discontinuation, or adverse event of special interest. Unlike in the blinded portion of the study, study pause rules were not applied in the open-label portion; however, if any of the threshold criteria were met, the sponsor was required to inform the data and safety monitoring board. None of the pause rules were met during the blinded and open-label portions of the study.

## Immunogenicity assays

Blood samples for immunogenicity assessments were collected at Day 1 (pre-injection 1), and Days 57, 209, and 394 after the first injection of mRNA-1273. The SARS CoV-2 pseudovirus neutralization assay (VAC62) is a cell-based assay that is designed to determine the ability of anti-SARS CoV-2 spike protein (D614G) neutralizing antibodies to inhibit the infection of 293T-ACE2 cells by SARS CoV-2 spike reporter virus particles, which express green fluorescent protein (GFP). A given serum sample was preincubated with a known quantity of SARS CoV-2 spike GFP for 60 (± 5) minutes prior to infection of 293T-ACE2 cells. COVID-19 infection was enumerated 48 (± 4) hours following infection by counting the number of fluorescent green cells using the Cytation 5 cell imaging reader. Sample antibody concentrations are reported in AU/mL units. This assay was conducted by PPD Laboratories in Richmond, Virginia, USA.

Binding antibodies to the SARS-CoV-2 spike protein were measured in a multiplex electrochemiluminescence method (VAC113). The assay used spike proteins of ancestral SARS-CoV-2 (D614G) or SARS-CoV-2 variants (alpha [B.1.1.7; V1] with the following amino acid changes in the spike protein: ΔH69-V70, ΔY144Y, N501Y, A570D, D614G, P681H, T761I, S982A, and D1118H; beta [B.1.351; 501Y-V2] with the following amino acid changes in the spike protein: L18F, D80A, D215G, Δ242-244, R246I, K417N, E484K, N501Y, D614G, and A701V; delta [B.1.617.2] with the following amino acid changes in the spike protein: T19R, Δ157/158, L452R, T478K, D614G, P681R, D950N; and gamma [P.1; V3] with the following amino acid changes in the spike protein: L18F, T20N, P26S, D138Y, R190S, K417T, E484K, N501Y, D614G, H655Y, T1027I, and V1176F) in human serum. The assay is based on the Meso-Scale Discovery technology, which employs multi-spot microtiter plates fitted with a series of electrodes associated with the bottom of each well. Anti-SARS-CoV-2 binding antibodies present in the test sample bind to the plates and form an antibody-antigen complex. Subsequently, SULFO-TAG-labeled anti-immunoglobulin G total antibodies bind to the test sample antibodies. The resulting electrochemiluminescence is measured in relative light units using the Meso-Scale Discovery SECTOR S 600 Plate Reader. Sample antibody concentrations are reported in AU/mL units. This assay was conducted by PPD Laboratories in Richmond, Virginia, USA.

## Diagnosis of COVID-19

The diagnosis of COVID-19 was determined using the primary case definition followed in the phase 3 COVE trial which required ≥2 systemic symptoms or ≥1 respiratory symptom plus ≥1 nasopharyngeal swab, nasal swab, or saliva sample positive for SARS-CoV-2 by RT-PCR.

If COVID-19 symptoms reported lasted at least 48 hours (except for fever and/or respiratory symptoms), an illness visit was scheduled within 72 hours to collect a nasopharyngeal or nasal swab.

Nasal swabs or nasopharyngeal specimens collected from individuals with symptoms of COVID-19 were tested for SARS-CoV-2 infection using a validated SARS-CoV-2 specific RT-PCR.

## Study eligibility criteria (Part 1B)

1. Participants must have been previously enrolled in the mRNA-1273-P203 study.

2. Female participants of childbearing potential may be enrolled in the study if the participant had a negative pregnancy test on the day of the first injection (open-label Day 1) and on the day of the second injection (open-label Day 29).

## Trial vaccine

The mRNA-1273 vaccine is a lipid nanoparticle dispersion of an mRNA encoding the prefusion stabilized spike protein of SARS-CoV-2 (ancestral Wuhan-Hu-1 isolate). mRNA-1273 injection is provided as a sterile liquid for injection, white to off-white dispersion in appearance, at a concentration of 0⸱5 mg/mL. Participants were randomly assigned in a 2:1 ratio to receive two injections of either mRNA-1273 vaccine (each injection containing 100 μg, for a total injection of 200 μg) or placebo (saline), 28 days apart.

# Supplementary Results

## Safety

One adverse event of chest pain was adjudicated by the cardiac event adjudication committee (CEAC) as probable myocarditis. An adolescent male participant who received placebo and crossed over to receive mRNA-1273 experienced a nonserious, moderate event of chest pain and vomiting 1 day after the second injection. This participant had a history of gastroesophageal reflux disease. He was evaluated; an electrocardiogram (ECG) showed sinus tachycardia, ST-segment elevation with right axis deviation. Differential diagnosis included suspected mild myocarditis, costochondritis, or gastrointestinal process. The participant remained home with close follow-up. Treatment included hydration, ibuprofen, and a heating pad, with avoidance of physical activity. The chest pain resolved 8 days after symptom onset. The investigator arranged a follow-up assessment with a cardiologist as a precaution, although the participant returned to his normal physical activity. Five months from symptom onset, the participant received a follow-up cardiology assessment, which included a physical examination, ECG, and echocardiogram; results of the testing were normal. The CEAC adjudicated the event as probable acute myocarditis based on the proximity of symptoms to vaccination and a repeat ECG that differed from the initial ECG.

# Supplementary Figures

## Supplementary Figure 1. Study design (Parts 1A, 1B, Part 2)


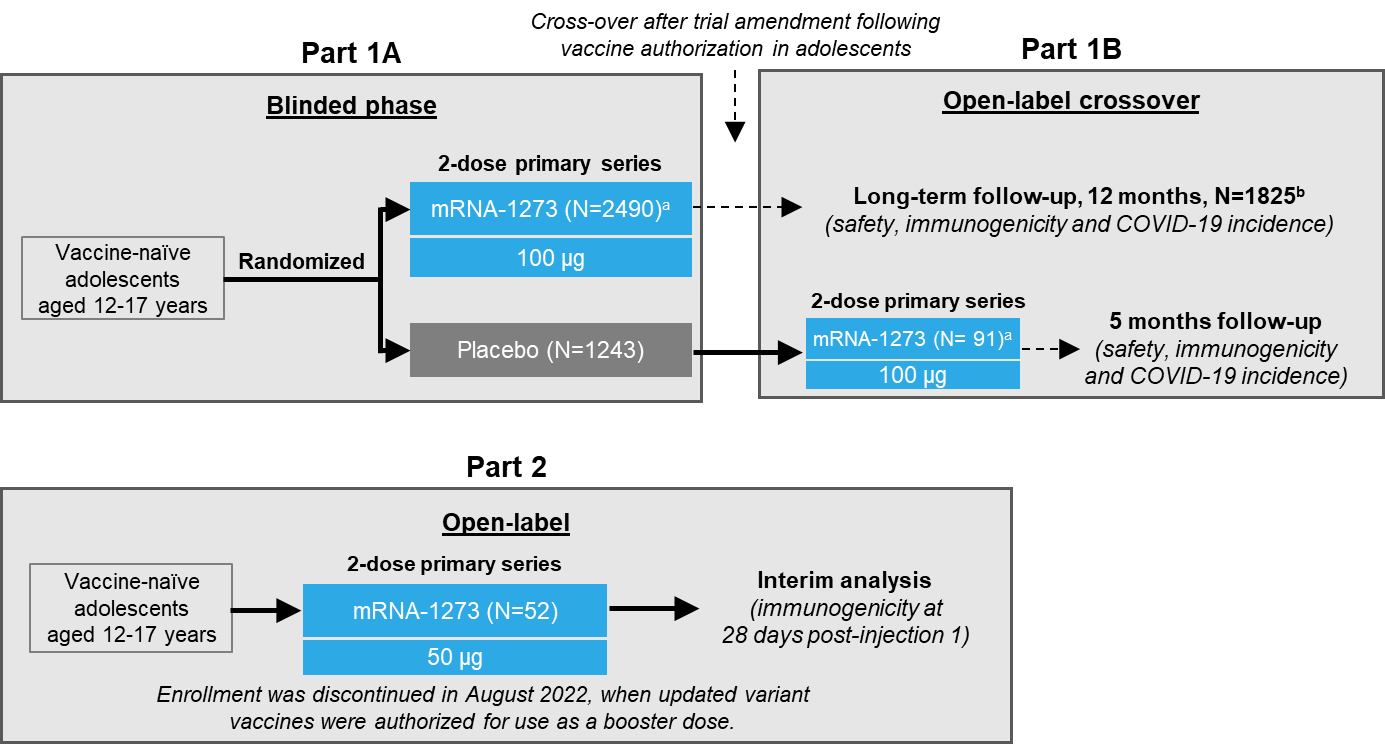


## Supplementary Figure 2. Part 1 analysis populations


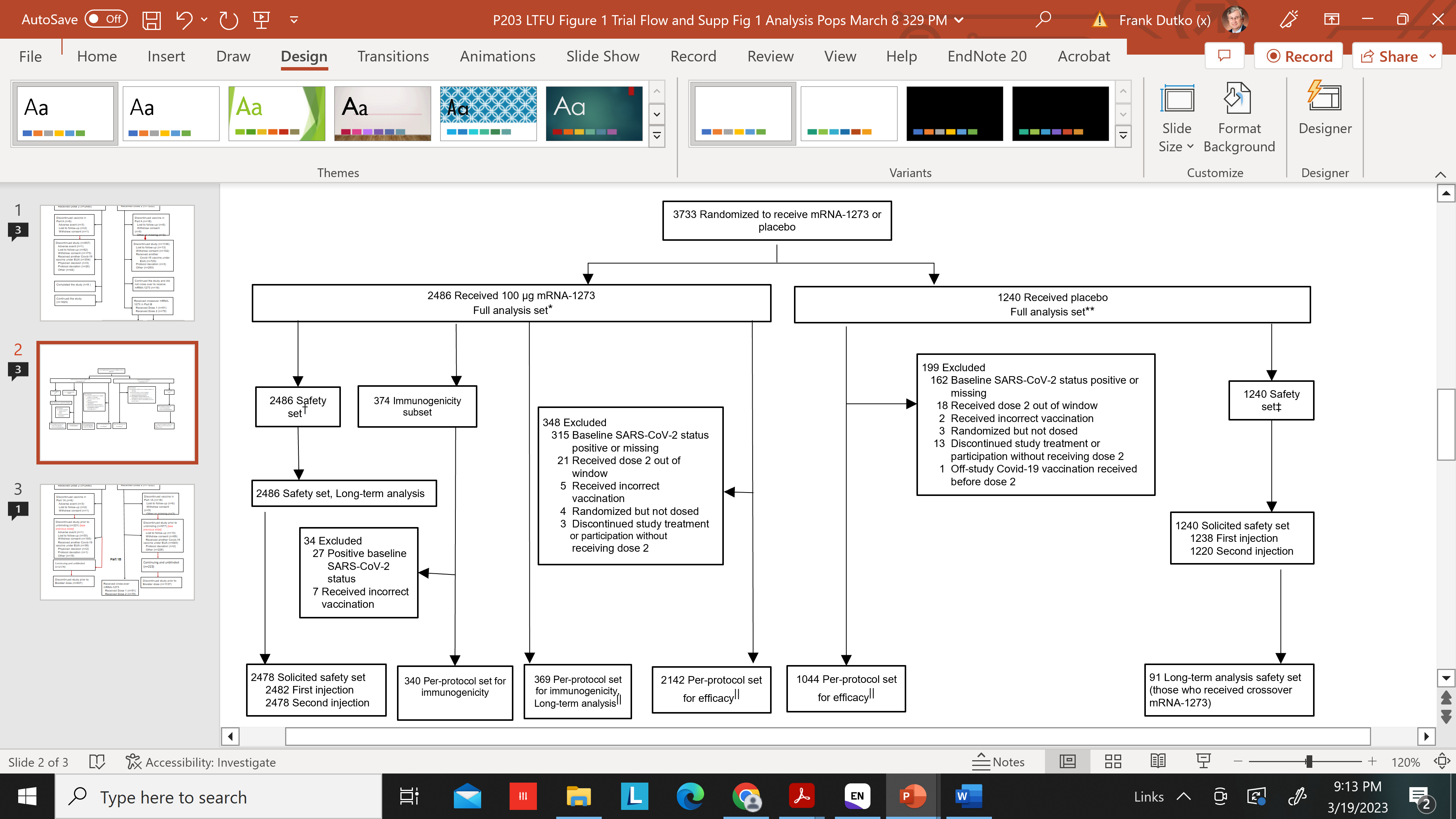


The full analysis set includes all enrolled and randomly assigned participants who received ≥1 injection of mRNA-1273 or placebo. The safety set includes all enrolled and randomly assigned participants who received any study injection. The solicited safety set consists of participants in the full analysis set who contributed any solicited adverse reaction data. The per-protocol efficacy set includes all participants in the full analysis set who received planned injections of study vaccination, had no immunologic or virologic evidence of prior COVID-19, and had no major protocol deviations that impacted key or critical efficacy data. The per-protocol immunogenicity subset includes participants in the full analysis set selected for the immunogenicity subset who received planned injections of study vaccination per schedule, complied with immunogenicity testing schedule, and have no major protocol deviations that impact key or critical data. Participants who were seropositive at baseline were excluded from the per-protocol immunogenicity subset.

*Four participants were randomly assigned to the mRNA-1273 group but did not receive mRNA-1273.

**Three participants were randomly assigned to the placebo group but did not receive the placebo.

## Supplementary Figure 3. Spike-binding antibody levels and seroresponse rates for variants and ancestral SARS-CoV-2 (long-term analysis)


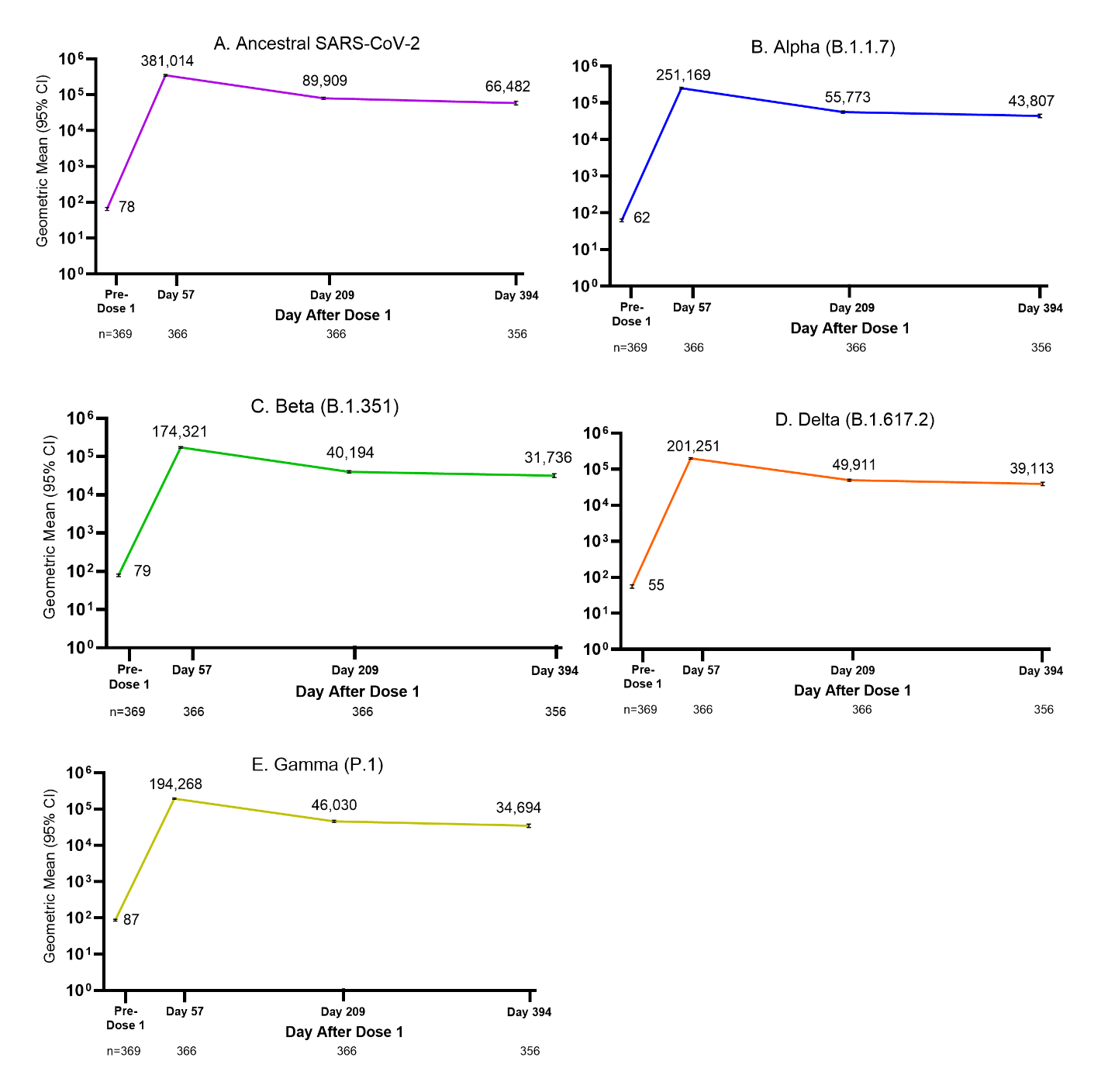


CI=confidence interval; IgG=immunoglobulin G; LLOQ=lower limit of quantitation; n=number of participants with non-missing data at Day 1 and the corresponding post-Day 1 time point; RT-PCR=reverse transcription polymerase chain reaction; ULOQ=upper limit of quantitation.

Geometric mean levels of spike-binding IgG antibodies for ancestral SARS-CoV-2 and variants were determined in the VAC113 assay for participants who were SARS-CoV-2 negative at baseline. 95% CIs were calculated based on the *t* distribution of the log-transformed values of the geometric mean levels and then back-transformed to the original scale for presentation as black brackets.

The SARS-CoV-2-negative at baseline group contains participants who were negative in the RT-PCR test and negative in the anti-SARS-CoV-2 nucleocapsid assay at baseline. Binding antibody values reported as below the LLOQ (69 for ancestral SARS-CoV-2; 52 for alpha; 111 for beta; 49 for delta; and 143 for gamma) were replaced by 0⸱5 x LLOQ. Values reported as greater than the ULOQ (14,400,000 for ancestral SARS-CoV-2; 8,800,000 for alpha; 5,000,000 for beta; 7,400,000 for delta; and 5,800,000 for gamma) were replaced by the ULOQ if the actual values were not available.

# Supplementary Tables

## Supplementary Table 1. Baseline demographics – Part 2

|  | **Part 2, per-protocol immunogenicity subset (baseline SARS-CoV-2 positive)*** | **Phase 3 COVE P301, per-protocol immunogenicity subset (baseline SARS-CoV-2 negative)^†^** |
| --- | --- | --- |
| **Characteristics** | **1 injection, mRNA-1273 50 µg**  **(N=44)** | **Two injections, mRNA-1273 100 µg**  **(N=296)** |
| Age at screening, years  Mean (range) | 14⸱0 (12-17) | 22⸱4 (18-25) |
| Age category at screening, n (%) |  |  |
| ≥12 and <16 years | 37 (84⸱1) | NA |
| ≥16 and <18 years | 7 (15⸱9) | NA |
| Sex, n (%) |  |  |
| Male | 20 (45⸱5) | 143 (48⸱3) |
| Female | 24 (54⸱5) | 153 (51⸱7) |
| Body mass index, median (range), kg/m^2^ | 21⸱62 (15⸱5-37⸱5) | 24⸱91 (16⸱7-48⸱7) |
| Race, n (%) |  |  |
| White | 22 (50⸱0) | 207 (69⸱9) |
| Black | 18 (40⸱9) | 29 (9⸱8) |
| Other, not reported, or unknown | 4 (9⸱1) | 60 (20⸱3) |
| Ethnicity, n (%) |  |  |
| Hispanic or Latinx | 12 (27⸱3) | 78 (26⸱4) |
| Not Hispanic or Latinx | 30 (68⸱2) | 216 (73⸱0) |
| Not reported or unknown | 2 (4⸱5) | 2 (0⸱7) |
| Pre-injection 1 RT-PCR result, n (%) |  |  |
| Negative | 43 (97⸱7) | 296 (100⸱0) |
| Positive | 1 (2⸱3) | 0 |
| Missing | 0 | 0 |
| Pre-injection 1 anti-SARS-CoV-2 nucleocapsid assay result, n (%) |  |  |
| Negative | 0 | 296 (100⸱0) |
| Positive | 44 (100⸱0) | 0 |
| Missing | 0 | 0 |
| Pre-injection 1 SARS-CoV-2 infection status,^‡^ n (%) |  |  |
| Negative | 0 | 296 (100⸱0) |
| Positive | 44 (100⸱0) | 0 |
| Missing | 0 | 0 |

NA=not applicable; RT-PCR=reverse transcription-polymerase chain reaction.

*The P203 Part 2 per-protocol immunogenicity subset (baseline SARS-CoV-2 positive) includes participants who were positive for SARS-CoV-2 at baseline, received ≥1 injection of mRNA-1273, complied with immunogenicity testing schedule, and have no major protocol deviations that impact key or critical data.

^†^The P301 per-protocol immunogenicity subset (baseline SARS-CoV-2 negative) includes randomly assigned participants who were negative for SARS-CoV-2 at baseline, received ≥1 injection of mRNA-1273, received planned injections of study vaccination per schedule, complied with immunogenicity testing schedule, and have no major protocol deviations that impact key or critical data.

^‡^Pre-injection 1 SARS-CoV-2 status was positive if there was evidence of previous SARS-CoV-2 infection, defined as positive binding antibody against the SARS-CoV-2 nucleocapsid or positive RT-PCR assay at Day 1; negative SARS-CoV-2 status was defined as negative binding antibody against the SARS-CoV-2 nucleocapsid and a negative RT-PCR assay at Day 1.

## Supplementary Table 2. Pseudovirus neutralizing antibody levels and seroresponse rates for ancestral SARS-CoV-2 (D614G) by SARS-CoV-2 infection status at or before Day 394 (long-term analysis)

|  | All participants  (N=369) | No prior infection at or before Day 394  (n=305) | Prior infection at or before Day 394  (n=64) |
| --- | --- | --- | --- |
| Day 1 (baseline), n* | 369 | 305 | 64 |
| Geometric mean concentration (95% CI)^†^ | 11 (11-12) | 11 (11-12) | 11 (10-13) |
| Day 57, n | 366 | 302 | 64 |
| Geometric mean concentration (95% CI) | 1868 (1759-1985) | 1887 (1768-2014) | 1781 (1515-2094) |
| Geometric mean fold rise (95% CI)^‡^ | 166 (154-180) | 167 (154-182) | 162 (130-201) |
| Seroresponse rate, n/N1, % (95% CI)^§^ | 366/366, 100% (99⸱0-100⸱0%) | 302/302, 100% (98⸱8-100⸱0%) | 64/64, 100% (94⸱4-100⸱0%) |
| Day 209, n | 366 | 302 | 64 |
| Geometric mean concentration (95% CI) | 625 (583-670) | 622 (582-665) | 640 (499-821) |
| Geometric mean fold rise (95% CI) | 55 (51-60) | 55 (50-60) | 58 (44-77) |
| Seroresponse rate, n/N1, % (95% CI) | 366/366, 100% (99⸱0-100⸱0%) | 302/302, 100% (98⸱8-100⸱0%) | 64/64, 100% (94⸱4-100⸱0%) |
| Day 394, n | 363 | 299 | 64 |
| Geometric mean concentration (95% CI) | 550 (490-618) | 388 (362-415) | 2823 (1940-4107) |
| Geometric mean fold rise (95% CI) | 49 (43-56) | 34 (31-37) | 256 (168-390) |
| Seroresponse rate, n/N1, % (95% CI) | 363/363, 100% (99⸱0-100⸱0%) | 299/299, 100% (98⸱8-100⸱0%) | 64/64, 100% (94⸱4-100⸱0%) |

CI=confidence interval; GMC=geometric mean concentration; GMFR=geometric mean fold rise; LLOQ=lower limit of quantitation; N1=number of participants with non-missing data at Day 1 and the corresponding post-Day 1 time point; RT-PCR= reverse transcription-polymerase chain reaction; SRR=seroresponse rate; ULOQ=upper limit of quantitation.

*Number of participants with non-missing data at the time point.

^†^GMCs of pseudovirus neutralizing antibodies against ancestral SARS-CoV-2 (D614G) were determined using the VAC62 assay. 95% CIs were calculated based on the *t* distribution of the log-transformed values of the GMCs and then back-transformed to the original scale for presentation.

^‡^GMFRs in GMCs of neutralizing antibodies are relative to Day 1. 95% CIs were calculated based on the *t* distribution of the log-transformed values of the GMCs and then back-transformed to the original scale for presentation.

^§^The seroresponse rates are relative to pre-injection 1. Seroresponse rate at a participant level is defined as a change from below the LLOQ to equal or above 4 x LLOQ, or at least a 4-fold rise if baseline is equal to or above the LLOQ. Percentages are based on N1. 95% CIs were calculated using the Clopper-Pearson method.

The no prior infection group contains participants who were negative in the RT-PCR test and negative in the anti-SARS-CoV-2 nucleocapsid assay at or before Day 394. The prior infection group contains participants who were positive in the RT-PCR test or positive in the anti-SARS-CoV-2 nucleocapsid assay at or before Day 394. Pseudovirus neutralizing antibody values reported as below the LLOQ (10 for ancestral SARS-CoV-2[D614G]) were replaced by 0⸱5 x LLOQ. Values reported as greater than the ULOQ (281,600) were replaced by the ULOQ if the actual values were not available.

## Supplementary Table 3. Long-term analysis of incidence rates of COVID-19 (phase 3 COVE case definition) in the mRNA-1273 group (combined blinded and open-label phases)

| Calendar month | Circulating SARS-CoV-2* | mRNA-1273 (n=2142) | | |
| --- | --- | --- | --- | --- |
|  |  | Cases/at risk  (%)^†^ | Person-months^‡^ | Incidence rate/1000 person-months  (95% CI)^§^ |
| Jan 2021 | Original | 0/10 | 1⸱8 | 0⸱000 (NE-2005⸱005) |
| Feb 2021 | Original | 0/167 | 64⸱3 | 0⸱000 (NE-57⸱344) |
| Mar 2021 | Original | 0/1149 | 614⸱1 | 0⸱000 (NE-6⸱007) |
| Apr 2021 | Original | 0/2116 | 1868⸱4 | 0⸱000 (NE-1⸱974) |
| May 2021 | Alpha variant | 0/2099 | 2131⸱6 | 0⸱000 (NE-1⸱731) |
| Jun 2021 | Alpha variant | 0/2085 | 2047⸱8 | 0⸱000 (NE-1⸱801) |
| Jul 2021 | Alpha variant | 2/2070 (<0⸱1) | 2103⸱8 | 0⸱951 (0⸱115-3⸱434) |
| Aug 2021 | Delta variant | 6/2061 (0⸱3) | 2091⸱8 | 2⸱868 (1⸱053-6⸱243) |
| Sep 2021 | Delta variant | 7/2038 (0⸱3) | 1984⸱5 | 3⸱527 (1⸱418-7⸱268) |
| Oct 2021 | Delta variant | 6/1990 (0⸱3) | 2004⸱8 | 2⸱993 (1⸱098-6⸱514) |
| Nov 2021 | Delta variant | 5/1949 (0⸱3) | 1903⸱0 | 2⸱627 (0⸱853-6⸱132) |
| Dec 2021 | Omicron variant | 44/1911 (2⸱3) | 1876⸱9 | 23⸱443 (17⸱034-31⸱472) |
| Jan 2022 | Omicron variant | 103/1703 (6⸱0) | 1112⸱1 | 92⸱616 (75⸱596-112⸱324) |
| Jan 2021 – Jan 2022 | Original, alpha, delta, and omicron | 173/2130 (8⸱1) | 19804⸱9 | 8⸱735 (7⸱482-10⸱138) |

CI=confidence interval.

Analysis was conducted using the per-protocol efficacy set.

*Based on Summary of Variant Surveillance, CDC Data Tracker, [https://COVID.cdc.gov/COVID-data-tracker/#variant-summary](https://covid.cdc.gov/covid-data-tracker/#variant-summary).

^†^Percentages based on number at risk.

^‡^Person-months for each time period is defined as the total months from the earlier date of the start of each time period or 14 days after second injection to the earliest date of the first occurrence of COVID-19, the end of each time period, study discontinuation, non-study COVID-19 vaccination, booster injection, or data cutoff. One month=30⸱4375 days.

^§^Incidence rate for each time period is defined as the number of participants with an event during the time period divided by the number of participants at risk during the time period and adjusted by person-months (total time at risk) in each treatment group. The 95% CIs were calculated using the exact method (Poisson distribution) and adjusted by person-months.

**References**

1. Centers for Disease Control and Prevention. Clinical Considerations: Myocarditis and pericarditis after receipt of COVID-19 vaccines among adolescents and young adults. 2023. <https://www.cdc.gov/vaccines/covid-19/clinical-considerations/myocarditis.html> (accessed May 30 2023).
