# Supplementary material for: Safety and durability of mRNA-1273–induced SARS-CoV-2 immune responses in adolescents: results from the phase 2/3 TeenCOVE trial
Source: eClinicalMedicine. 2024 Jul 18;74:102720. doi: 10.1016/j.eclinm.2024.102720 (PMC11293523; doi:10.1016/j.eclinm.2024.102720)
Supplement: mRNA-1273-P203-protocol amendment 6-final [file mmc2.pdf]

CLINICAL STUDY PROTOCOL

**Protocol Title:**	A Phase 2/3, Randomized, Observer-Blind, Placebo-Controlled Study to Evaluate the Safety, Reactogenicity, and Effectiveness of mRNA-1273 SARS-CoV-2 Vaccine in Healthy Adolescents 12 to <18 Years of Age
**Protocol Number:**	mRNA-1273-P203
**Sponsor Name:**	ModernaTX, Inc.
**Legal Registered Address:**	200 Technology Square<br>Cambridge, MA 02139
**Sponsor Contact and Medical Monitor:**	Amparo Figueroa, MD, MPH<br>ModernaTX, Inc.<br>200 Technology Square<br>Cambridge, MA 02139<br>Telephone: 1-617-335-3762<br>e-mail: Amparo.Figueroa@modernatx.com
**Regulatory Agency Identifier Number(s):**	IND: 019745
**Amendment Number:**	6
**Date of Amendment 6**	22 Jun 2023
**Date of Amendment 5**	11 Oct 2022
**Date of Amendment 4:**	25 Jan 2022
**Date of Amendment 3:**	04 Nov 2021
**Date of Amendment 2:**	27 Jul 2021
**Date of Amendment 1:**	23 Mar 2021
**Date of Original Protocol:**	04 Nov 2020

CONFIDENTIAL

All financial and nonfinancial support for this study will be provided by ModernaTX, Inc. The concepts and information contained in this document or generated during the study are considered proprietary and may not be disclosed in whole or in part without the expressed written consent of ModernaTX, Inc. The study will be conducted according to the *International Council for Harmonisation (ICH) of Technical Requirements for Registration of Pharmaceuticals for Human Use, E6(R2) Good Clinical Practice (GCP) Guidance.*

PROTOCOL APPROVAL – SPONSOR SIGNATORIES

**Study Title:**	A Phase 2/3, Randomized, Observer-Blind, Placebo-Controlled Study to Evaluate the Safety, Reactogenicity, and Effectiveness of mRNA-1273 SARS-CoV-2 Vaccine in Healthy Adolescents 12 to <18 Years of Age
**Protocol Number:**	mRNA-1273-P203
**Amendment Number:**	6
**Amendment 6 Date:**	22 Jun 2023

Protocol accepted and approved by:

See e-signature and date signed on the last page of the document.

Amparo Figueroa, MD, MPH<br>Director Clinical Development,<br>Infectious Diseases<br>ModernaTX, Inc.<br>200 Technology Square<br>Cambridge, MA 02139<br>Telephone: 1-617-335-3762	Date

DECLARATION OF INVESTIGATOR

I have read and understood all sections of the protocol entitled "A Phase 2/3, Randomized, Observer-Blind, Placebo-Controlled Study to Evaluate the Safety, Reactogenicity, and Effectiveness of mRNA-1273 SARS-CoV-2 Vaccine in Healthy Adolescents 12 to <18 Years of Age" and the most recent version of the investigator's brochure.

I agree to supervise all aspects of the protocol and to conduct the clinical investigation in accordance with the current protocol, the *International Council for Harmonisation (ICH) of Technical Requirements for Registration of Pharmaceuticals for Human Use, E6(R2) Good Clinical Practice (GCP) Guidance*, and all applicable government regulations. I will not make changes to the protocol before consulting with ModernaTX, Inc. or implement protocol changes without IRB approval except to eliminate an immediate risk to participants.

I agree to administer study treatment only to participants under my personal supervision or the supervision of a sub-investigator. I will not supply study treatment to any person not authorized to receive it. I also agree that persons debarred from conducting or working on clinical studies by any court or regulatory agency will not be allowed to conduct or work on studies for the Sponsor or a partnership in which the Sponsor is involved. I will immediately disclose it in writing to the Sponsor if any person who is involved in the study is debarred, or if any proceeding for debarment is pending, or, to the best of my knowledge, threatened.

I will not disclose confidential information contained in this document including participant information, to anyone other than the recipient study staffs and members of the IRB. I agree to ensure that this information will not be used for any purpose other than the evaluation or conduct of the clinical investigation without the prior written consent from ModernaTX, Inc. I will not disclose information regarding this clinical investigation or publish results of the investigation without authorization from ModernaTX, Inc.

The signature below provides the necessary assurance that this study will be conducted according to all stipulations of the protocol, including statements regarding confidentiality, and according to local legal and regulatory requirements, US federal regulations, and ICH E6(R2) GCP guidelines.

_______________________________________ ______________________

Signature of principal investigator Date

____

Printed name of principal investigator

PROTOCOL AMENDMENT SUMMARY OF CHANGES

DOCUMENT HISTORY	
**Document**	**Date**
Amendment 6	22 Jun 2023
Amendment 5	11 Oct 2022
Amendment 4	25 Jan 2022
Amendment 3	04 Nov 2021
Amendment 2	27 Jul 2021
Amendment 1	23 Mar 2021
Original Protocol	04 Nov 2020

Amendment 6, 22 Jun 2023: Current Amendment

This amendment is considered to be substantial based on the criteria set forth in Article 10(a) of Directive 2001/20/EC of the European Parliament and the Council of the European Union.

Main Rationale for the Amendment:

The main purpose of this amendment is to update the primary immunogenicity objective of Part 3, per regulatory feedback, to evaluate the effectiveness of a single 50 µg dose of mRNA-1273.222 in COVID-19 vaccine-naïve, baseline SARS-CoV-2 positive individuals 12 to 17 years of age, by comparing the immune response in these individuals at 1-month post single dose (50 µg mRNA-1273.222) to the 1-month post Dose 2 (100 µg mRNA-1273) immune response from baseline SARS-CoV-2 negative participants in Study P301 adults for whom clinical vaccine efficacy was demonstrated.

Summary of Major Changes in Protocol Amendment 6:

The Summary of Changes table provided below describes the major changes made in Protocol Amendment 6 relative to Protocol Amendment 5, including the sections modified and the corresponding rationales. The synopsis of Protocol Amendment 6 has been modified to correspond to changes in the body of the protocol. Formatting edits were introduced throughout the protocol to accommodate the recent update to the Sponsor’s style guide. Literature references were updated based on changes in cited content. Minor copy edits and administrative updates were made throughout the protocol to align with new content as well as for clarity, readability, and/or accuracy.

Section Number and Name	Description of Change	Brief Rationale
Section 2 Objectives and Endpoints; Section 3 Study Design; Section 3.2 Scientific Rationale for Study Design; Section 8.2.3 Statistical Hypothesis	Modifications to primary and (key) secondary immunogenicity objectives and endpoints, statistical hypothesis, power and sample size, and analysis in Part 3 of the study.	Updates to immunogenicity objectives and endpoints were made in response to regulatory feedback.

Section Number and Name	Description of Change	Brief Rationale
(Part 3); Section 8.3.3 Power and Sample Size (Part 3); Section 8.5.8 Part 3 Analysis; Section 8.6 Multiplicity Adjustment	Revised primary immunogenicity objective to include coprimary immunogenicity endpoints:<br>• To demonstrate superior GM value of Ab against Omicron BA.4/BA.5 after single 50 µg dose of mRNA-1273.222 in COVID-19 vaccine-naïve, baseline SARS-CoV-2 positive adolescents in Part 3 of study P203 compared to GM value of Ab against Omicron BA.4/BA.5 after 2 doses (100 µg each) of mRNA-1273 in baseline SARS-CoV-2 negative young adult participants 18 to 25 years of age in Study P301<br>• To demonstrate noninferior GM value of Ab against ancestral strain after single 50 µg dose of mRNA-1273.222 in COVID-19 vaccine-naïve, baseline SARS-CoV-2 positive adolescents in Part 3 of study P203 compared to GM value of Ab against ancestral strain after 2 doses (100 µg each) of mRNA-1273 in baseline SARS-CoV-2 negative young adult participants 18 to 25 years of age in Study P301<br>Key secondary endpoints were revised to secondary endpoints without hypothesis testing.	
Section 3.1.3 Part 3 Study Design; Section 8.3.3 Power and Sample Size (Part 3)	The target number of enrolled participants was also revised (from N of 500 to N of at least 300) according to the new sample size and power calculation (based on new information): With at least 300 adolescents enrolled, the study will have at least a 95% probability to observe at least 1 participant with an AE at a true AE rate of 1%. A sample size of approximately 168 participants is required to sufficiently power the hypothesis testing of new coprimary immunogenicity endpoints, resulting in approximately 240 enrolled participants required for immunogenicity primary analysis after considering the potential 30% exclusion rate for the PP Immunogenicity Subset-baseline SARS-CoV-2 positive (PPIS-POS), as participants may be baseline SARS-CoV-2	Update to sample size based revised Part 2 data based and made in response to regulatory feedback.

Section Number and Name	Description of Change	Brief Rationale
	negative or have missing immunogenicity results due to any reason or protocol deviations impacting critical data.	
Section 4.1.2 Exclusion Criteria	Updated Exclusion Criterion #12 as follows:<br>Any licensed vaccine within 28 days before the first dose of IP or plans for receipt of any licensed vaccine within 28 days before and/or after each dose of IP.	Participants can be enrolled if they have received or plan to receive a licensed vaccine outside of these clarified windows and in between doses.
Section 1.2.2 Clinical Studies	Added Study mRNA-1273-P206 in the list of ongoing trials being conducted to support SARS-CoV-2 variants.	Updated section with the latest information.
Section 1.2.2.3 Phase 2/3 Study mRNA-1273-P205; Section 3.2 Scientific Rationale for Study Design; Section 3.3 Justification for Dose, Control Product, and Choice of Study Population	Addition of interim results from Part 1C-1 and Part 2.	Updated section with the latest information.
Section 1.2.2 Clinical Studies, Section 3.3 Justification for Dose, Control Product, and Choice of Study Population	Added latest effectiveness data on mRNA-1273.222 vaccine from literature based on the mRNA-1273-P205 Study.	Updated section with the latest information.
Table 7 Analysis Sets	For the PP Immunogenicity Subset for Part 3, clarified the following points:<br>The PP Immunogenicity Subset for Part 3 with baseline-positive SARS-CoV-2 status will serve as the population for the primary and secondary analysis of immunogenicity data at Day 29 in Part 3. The PP Immunogenicity Subset (Part 3, at Day 209) will serve as the population for the primary and secondary analysis of immunogenicity data at Day 209 in Part 3.<br>Additionally, added the following note under Table 7:<br>Positive SARS-CoV-2 status at baseline is defined as a positive RT-PCR test for SARS-CoV-2, and/or a positive serology test based on bAb specific to SARS-CoV-2 nucleocapsid (as measured by Roche Elecsys Anti SARS-CoV-2 assay) on or	Clarifying the definition of the SARS-CoV-2 status at baseline.

Section Number and Name	Description of Change	Brief Rationale
	before Day 1. Negative status is defined as a negative RT-PCR test for SARS-CoV-2, and a negative serology test based on bAb specific to SARS-CoV-2 nucleocapsid (as measured by Roche Elecsys Anti SARS-CoV-2 assay) on or before Day 1.	

Abbreviations: AR = adverse reaction; bAb = binding antibody; CBER = Center for Biologics Evaluation and Research; D = day; EUA = Emergency Use Authorization; ICH = International Council for Harmonisation; IP = investigational product; MAAE = medically attended adverse event; mITT = modified intent-to-treat; nAb = neutralizing antibody; PP = per-protocol; SAE = serious adverse event; RT-PCR = reverse transcriptase polymerase chain reaction; SARS-CoV2 = Severe Acute Respiratory Syndrome coronavirus 2; SoA = Schedule of Assessments.

IRB and Regulatory Authority Approval

A copy of this amended protocol will be sent to the IRB and regulatory authority.

The changes described in this amended protocol require IRB approval prior to implementation. In addition, if the changes herein affect the informed consent, sites are required to update and submit a revised informed consent for approval that incorporates the changes described in this amended protocol.

PROTOCOL SYNOPSIS

Name of Sponsor/Company: ModernaTX, Inc.

Name of Investigational Product: mRNA-1273 for injection

Name of Active Ingredient: mRNA-1273

Protocol Title: A Phase 2/3, Randomized, Observer-Blind, Placebo-Controlled Study to Evaluate the Safety, Reactogenicity, and Effectiveness of mRNA-1273 SARS-CoV-2 Vaccine in Healthy Adolescents 12 to <18 Years of Age

Protocol Number: mRNA-1273-P203

Study Period (months): Up to 25 months

Phase of Development: Phase 2/3

Estimated date first participant enrolled: 30 Nov 2020

Estimated date last participant completed: 01 Aug 2024

Total Number of Sites: Approximately 35 to 64 study sites in the USA or out of USA countries

Part 1 Objectives and Endpoints

Objectives	Endpoints
**Primary Objectives**	**Primary Endpoints**
• To evaluate the safety and reactogenicity of 100 µg mRNA-1273 vaccine administered in 2 doses 28 days apart	• Solicited local and systemic ARs through 7 days after each injection<br>• Unsolicited AEs through 28 days after each injection<br>• MAAEs through the entire study period<br>• SAEs through the entire study period<br>• AESIs through the entire study period<br>• Vital sign measurements<br>• Physical examination findings
• To infer efficacy of mRNA-1273 (100 µg, 2 doses 28 days apart), serum Ab responses obtained 28 days after the second injection of mRNA-1273 (Day 57) will be either:<br>– Evaluated against an accepted Ab threshold of protection against	• The proportion of participants with a serum Ab level at Day 57 ≥ an Ab threshold of protection1<br>• The primary vaccine response as measured by GM value of serum Ab level and SRR from Study P203 vaccine recipients at Day 57 compared with those obtained from young adult recipients (18 to 25 years of age) at

COVID-19 (if established in Study P301)<br>– Compared in primary vaccine response as measured by GM values of serum Ab and SRR in Study P203 with those obtained from young adult recipients (18 to 25 years of age) of mRNA-1273 in the clinical endpoint efficacy trial (Study P301)	Day 57 in the clinical endpoint efficacy trial (Study P301)[2]<br>1. If an accepted serum Ab threshold of vaccine protection against COVID-19 is available, this analysis will form the basis to infer efficacy.<br>2. If a threshold is not available, efficacy will be inferred based on establishing noninferiority of adolescent (12 to < 18 years; this clinical study) to adult GM values of serum Ab and SRR obtained in Study P301 (GM value 12 to < 18 years / GM value 18 to 25 years).
**Secondary Objectives**	**Secondary Endpoints**
• To evaluate the persistence of the immune response of mRNA-1273 vaccine (100 µg) administered in 2 doses 28 days apart, as assessed by the level of SARS-CoV-2 S2P-specific bAb through 1 year after Dose 2	• The GM value of SARS-CoV-2 S2P-specific bAb on Day 1, Day 57 (1 month after Dose 2), Day 209 (6 months after Dose 2), and Day 394 (1 year after Dose 2)
• To evaluate the persistence of the immune response of mRNA-1273 vaccine (100 µg) administered in 2 doses 28 days apart, as assessed by the level of nAb through 1 year after Dose 2	• The GM values of SARS-CoV-2-specific nAb on Day 1, Day 57 (1 month after Dose 2), Day 209 (6 months after Dose 2), and Day 394 (1 year after Dose 2)
• To evaluate the effect of mRNA-1273 on the incidence of SARS-CoV-2 infection compared with the incidence among placebo recipients	• The incidence of SARS-CoV-2 infection (symptomatic or asymptomatic infection) counted starting 14 days after the second dose of IP<br>• SARS-CoV-2 infection will be defined in participants with negative SARS-CoV-2 at baseline:<br>– bAb level against SARS-CoV-2 nucleocapsid protein negative at Day 1 that becomes positive (as measured by Roche Elecsys) at Day 57 or later, OR<br>– Positive RT-PCR counted starting 14 days after the second dose of IP
• To evaluate the incidence of asymptomatic SARS-CoV-2 infection after vaccination with mRNA-1273 or placebo	• The incidence of asymptomatic SARS-CoV-2 infection measured by RT-PCR and/or bAb levels against SARS-CoV-2 nucleocapsid protein (by Roche Elecsys) counted starting 14 days after the second dose of IP in participants with negative SARS-CoV-2 at baseline
• To evaluate the incidence of COVID-19 after vaccination with mRNA-1273 or	• The incidence of the first occurrence of COVID-19 starting 14 days after the second

placebo. COVID-19 is defined as clinical symptoms consistent with SARS-CoV-2 infection AND positive RT-PCR for SARS-CoV-2	dose of IP, where COVID-19 is defined as symptomatic disease based on the following criteria:<br>– The participant must have experienced at least TWO of the following systemic symptoms: Fever (≥ 38ºC/≥ 100.4ºF), chills, myalgia, headache, sore throat, new olfactory and taste disorder(s), OR<br>– The participant must have experienced at least ONE of the following respiratory signs/symptoms: cough, shortness of breath or difficulty breathing, OR clinical or radiographical evidence of pneumonia; AND<br>– The participant must have at least 1 NP swab, nasal swab, or saliva sample (or respiratory sample, if hospitalized) positive for SARS-CoV-2 by RT-PCR
**Exploratory Objectives**	**Exploratory Endpoints**
• To evaluate the genetic and/or phenotypic relationships of isolated SARS-CoV-2 strains to the vaccine sequence	• Alignment of genetic sequence of viral isolates with that of the vaccine sequence and comparison of bAb and nAb titers against isolated strain relative to prototype vaccine strain
• To describe the ratio or profile of specific bAb relative to nAb in serum	• Relative amounts or profiles of S protein-specific bAb and specific nAb levels/titers in serum
• To characterize the clinical profile and immune responses of participants with COVID-19 or with SARS-CoV-2 infection	• Description of clinical severity and immune responses of participants who are identified as infected by SARS-CoV-2 (COVID-19)
• To evaluate the incidence of asymptomatic SARS-CoV-2 infection after vaccination with mRNA-1273 or placebo in participants with serologic evidence of infection at baseline	• GM value and GMFR of bAb levels against SARS-CoV-2 nucleocapsid protein (quantitative IgG) and % of participants with 2x, 3x, and 4x rise of bAb relative to baseline
**Exploratory Objectives for Part 1B:**	**Exploratory Endpoints for Part 1B:**
• To evaluate the safety of 2 doses of mRNA-1273	• MAAEs through the entire study period<br>• SAEs through the entire study period<br>• AESIs through the entire study period<br>• AEs leading to discontinuation from study participation through the last day of study participation

• To evaluate the incidence of SARS-CoV-2 infection or COVID-19 after vaccination with mRNA-1273	• The incidence of SARS-CoV-2 infection (symptomatic or asymptomatic infection) counted starting 14 days after the second dose of mRNA-1273<br>• To evaluate the incidence of asymptomatic SARS-CoV-2 infection after vaccination with mRNA-1273 measured by RT-PCR and/or bAb levels against SARS-CoV-2 nucleocapsid protein (by Roche Elecsys) counted starting 14 days after the second dose in participants with negative SARS-CoV-2 at baseline<br>• The incidence of the first occurrence of symptomatic COVID-19 starting 14 days after the second dose of mRNA-1273
**Objectives for Part 1C-1 – Homologous Booster Dose**	**Endpoints for Part 1C-1 – Homologous Booster Dose**
**Primary Objectives**	**Primary Endpoints**
• To evaluate the safety of the 50 µg BD of mRNA-1273	• Solicited local and systemic ARs through 7 days after BD<br>• Unsolicited AEs through 28 days after BD injection<br>• MAAEs through the entire study period<br>• SAEs through the entire study period<br>• AESIs through the entire study period<br>• AEs leading to discontinuation from study participation post BD through the last day of study participation
• To infer effectiveness of 50 µg of mRNA-1273 booster by establishing noninferiority of Ab response after the BD compared to the primary series of mRNA-1273. GM values of serum Ab and SRR of postbooster in Study P203 compared with primary series from young adult (18 to 25 years of age) recipients of mRNA-1273 in the clinical endpoint efficacy trial (Study P301)	Coprimary endpoint(s):<br>• GM value of postbooster (post Dose 3) Ab against ancestral strain in Study P203 as compared to post primary series (post Dose 2) against ancestral strain in the young adults in Study P301<br>• SRR of postbooster/Dose 3 from baseline (pre Dose 1) as compared to post Dose 2 from baseline (pre Dose1) against ancestral strain in the young adults in Study P301, using 4-fold rise definition<br>– Surresponses is defined as Ab value change from baseline (pre Dose 1) below the LLOQ to ≥4 × LLOQ, or at least a 4-fold rise if baseline is ≥LLOQ
**Key Secondary Objective**	**Key Secondary Endpoint**

• To evaluate immune response elicited by the 50 µg prototype booster of mRNA-1273 against variant(s) of interest	• GM value of postbooster (post Dose 3) Ab against circulating strain as compared to post primary series (post Dose 2) against circulating strain<br>• SRR of postbooster/Dose 3 from baseline (pre Dose 1) as compared to post Dose 2 from baseline (pre Dose 1) against circulating strain using 4-fold rise definition
**Exploratory Objectives**	**Exploratory Endpoints**
• To evaluate the persistence of the immune response of the BD of mRNA-1273 vaccine (50 µg) as assessed by the level of SARS-CoV-2 S2P specific bAb through 1 year after BD	• The GM values of SARS-CoV-2 S2P specific bAb on BD-Day 1, BD-Day 29 (1 month after BD), BD-Day 181 (6 months after BD), and BD-Day 361 (1 year after BD)
• To evaluate the persistence of the immune response of the BD of mRNA-1273 vaccine (50 µg) as assessed by the level of nAb through 1 year after BD	• The GM values of SARS-CoV-2-specific nAb on BD-Day 1, BD-Day 29 (1 month after BD), BD-Day 181 (6 months after BD), and BD-Day 361 (1 year after BD)
• To evaluate the incidence of SARS-CoV-2 infection or COVID-19 after vaccination with mRNA-1273	• The incidence of SARS-CoV-2 infection (symptomatic or asymptomatic infection) counted starting 14 days after BD of mRNA-1273<br>• To evaluate the incidence of asymptomatic SARS-CoV-2 infection after vaccination with mRNA-1273 measured by RT-PCR and/or bAb levels against SARS-CoV-2 nucleocapsid protein (by Roche Elecsys) counted starting 14 days after BD in participants with negative SARS-CoV-2 at baseline or prebooster<br>• The incidence of the first occurrence of symptomatic COVID-19 starting 14 days after BD of mRNA-1273
**Objectives for Part 1C-2 – Heterologous Booster Dose**	**Endpoints for Part 1C-2 – Heterologous Booster Dose**
**Primary Objectives**	**Primary Endpoints**
• To evaluate the safety of the 50 µg BD of mRNA-1273 in participants who received non-Moderna COVID-19 primary series vaccination	• Solicited local and systemic ARs through 7 days after BD<br>• Unsolicited AEs through 28 days after BD injection<br>• MAAEs through the entire study period<br>• SAEs through the entire study period<br>• AESIs through the entire study period

	• AEs leading to discontinuation from study participation post BD through the last day of study participation
• To evaluate immune response elicited by the 50 µg booster of mRNA-1273 in participants who received non-Moderna COVID-19 primary series vaccination	• GM value of postbooster Ab (at BD-Day 29) against ancestral strain
**Key Secondary Objective**	**Key Secondary Endpoint**
• To evaluate immune response elicited by the 50 µg prototype booster of mRNA-1273 against variant(s) of interest in participants who received non-Moderna COVID-19 primary series vaccination	• GM value of postbooster Ab (at BD-Day 29) against circulating strain
**Exploratory Objectives**	**Exploratory Endpoints**
• To evaluate the persistence of the immune response of the BD of mRNA-1273 vaccine (50 µg) as assessed by the level of SARS-CoV-2 S2P specific bAb through 1 year after BD in participants who received non-Moderna COVID-19 primary series vaccination	• The GM values of SARS-CoV-2 S2P specific bAb on BD-Day 1, BD-Day 29 (1 month after BD), BD-Day 181 (6 months after BD), and BD-Day 361 (1 year after BD)
• To evaluate the persistence of the immune response of the BD of mRNA-1273 vaccine (50 µg) as assessed by the level of nAb through 1 year after BD in participants who received non-Moderna COVID-19 primary series vaccination	• The GM values of SARS-CoV-2-specific nAb on BD-Day 1, BD-Day 29 (1 month after BD), BD-Day 181 (6 months after BD), and BD-Day 361 (1 year after BD)
• To evaluate the incidence of SARS-CoV-2 infection or COVID-19 after booster vaccination with mRNA-1273 in participants who received non-Moderna COVID-19 primary series vaccination	• The incidence of SARS-CoV-2 infection (symptomatic or asymptomatic infection) counted starting 14 days after BD of mRNA-1273<br>• To evaluate the incidence of asymptomatic SARS-CoV-2 infection after vaccination with mRNA-1273 measured by RT-PCR and/or bAb levels against SARS-CoV-2 nucleocapsid protein (by Roche Elecsys) counted starting 14 days after BD in participants with negative SARS-CoV-2 at baseline or prebooster<br>• The incidence of the first occurrence of symptomatic COVID-19 starting 14 days after BD of mRNA-1273

Abbreviations: Ab = antibody; AE = adverse event; AESI = adverse event of special interest; AR = adverse reaction; bAb = binding antibody; BD = booster dose; COVID-19 = coronavirus disease 2019; GM = geometric mean; GMFR = geometric mean fold rise; IgG = immunoglobulin G; IP = investigational product; LLOQ = lower limit of quantification; MAAE = medically attended adverse event; nAb = neutralizing antibody; NP = nasopharyngeal; RT-PCR = reverse transcriptase

polymerase chain reaction; S = spike; S2P = S protein; SAE = serious adverse event; SARS-CoV-2 = Severe Acute Respiratory Syndrome coronavirus 2; SRR = seroresponse rate.

Part 2 Objectives and Endpoints

Primary Objectives	Primary Endpoints
• To evaluate the safety and reactogenicity of the 50 µg mRNA-1273 vaccine administered in 2 doses 28 days apart	• Solicited local and systemic ARs through 7 days after each injection<br>• Unsolicited AEs through 28 days after each injection<br>• MAAEs through the entire study period<br>• SAEs through the entire study period<br>• AESIs through the entire study period<br>• AEs leading to discontinuation from study participation from Dose 1 through the last day of study participation
• To evaluate immune response elicited by the 50 µg mRNA-1273 vaccine administered in 2 doses 28 days apart	• GM value of post Dose 2 (Day 57) Ab against ancestral strain in Part 2 of Study P203<br>• SRR of post Dose 2 from baseline (pre Dose 1) in Part 2 of Study P203 against ancestral strain, using 4-fold rise definition<br>– Seroresponse is defined as Ab value change from baseline (pre Dose 1) below the LLOQ to ≥4 × LLOQ, or at least a 4-fold rise if baseline is ≥LLOQ
**Exploratory Objectives**	**Exploratory Endpoints**
• To evaluate the persistence of the immune response of 50 µg mRNA-1273 vaccine administered in 2 doses 28 days apart as assessed by the level of SARS-CoV-2 S2P specific bAb through 1 year after Dose 2	• The GM values of SARS-CoV-2 S2P specific bAb on Day 1, Day 57 (1 month after Dose 2), Day 209 (6 months from Dose 2), Day 394 (1 year from Dose 2)
• To evaluate the persistence of the immune response of the 50 µg mRNA-1273 vaccine as assessed by the level of nAb through 1 year after Dose 2	• The GM values of SARS-CoV-2-specific nAb on Day 1, Day 57 (1 month after Dose 2), Day 209 (6 months from Dose 2), Day 394 (1 year from Dose 2)
• To evaluate the incidence of SARS-CoV-2 infection or COVID-19 after vaccination with mRNA-1273	• The incidence of SARS-CoV-2 infection (symptomatic or asymptomatic infection) counted starting 14 days after Dose 2 of mRNA-1273<br>• To evaluate the incidence of asymptomatic SARS-CoV-2 infection after vaccination with mRNA-1273 measured by RT-PCR and/or bAb levels against SARS-CoV-2 nucleocapsid protein (by Roche Elecsys) counted starting 14 days after

	Dose 2 in participants with negative SARS-CoV-2 at baseline<br>• The incidence of the first occurrence of symptomatic COVID-19 starting 14 days after Dose 2 of mRNA-1273

Abbreviations: Ab = antibody; AE = adverse event; AESI = adverse event of special interest; AR = adverse reaction; bAb = binding antibody; COVID-19 = coronavirus disease 2019; GM = geometric mean; LLOQ = lower limit of quantification; MAAE = medically attended adverse event; nAb = neutralizing antibody; RT-PCR = reverse transcriptase polymerase chain reaction; S2P = S protein; SAE = serious adverse event; SARS-CoV-2 = Severe Acute Respiratory Syndrome coronavirus 2; SRR = seroresponse rate.

Part 3 Objectives and Endpoints

Primary Objectives	Primary Endpoints
• To evaluate the safety and reactogenicity of the 50 µg mRNA-1273.222 vaccine administered in 2 doses 6 months apart.	• Solicited local and systemic ARs through 7 days after each injection.<br>• Unsolicited AEs through 28 days after each injection.<br>• MAAEs through the entire study period.<br>• SAEs through the entire study period.<br>• AESIs through the entire study period.<br>• AEs leading to withdrawal from study participation from Dose 1 through the last day of study participation.<br>• AEs leading to discontinuation from dosing from Dose 1 through the last day of study participation.
• To infer effectiveness of the 50 µg mRNA-1273.222 vaccine based on immune response against SARS-CoV-2 VOC (Omicron BA.4/BA.5) and ancestral strain obtained 28 days post Dose 1 in the baseline SARS-CoV-2 positive population.	• GM value of post Dose 1 (Day 29) of mRNA-1273.222 Ab against SARS-CoV-2 VOC (Omicron BA.4/BA.5) in adolescents who are baseline SARS-CoV-2 positive in Part 3 of Study P203 compared to that of post primary series of mRNA-1273 (post Dose 2 [Day 57] of 100 µg) in young adults 18 to 25 years of age who are baseline SARS-CoV-2 negative in Study P301 (superiority testing).<br>• GM value of post Dose 1 (Day 29) of mRNA-1273.222 Ab against ancestral strain in adolescents who are baseline SARS-CoV-2 positive in Part 3 of Study P203 compared to that of post primary series of mRNA-1273 (post Dose 2 [Day 57] of 100 µg) in young adults 18 to 25 years of age who are baseline SARS-CoV-2 negative in Study P301 (noninferiority testing).
**Secondary Objectives**	**Secondary Endpoints**

• To evaluate immune response elicited by 50 µg mRNA-1273.222 vaccine administered in 2 doses 6 months apart based on immune responses against Omicron BA.4/BA.5 and ancestral strain obtained 28 days post Dose 1 or 28 days post Dose 2.	• SRR of post Dose 1 (Day 29) from baseline (pre Dose 1) against Omicron BA.4/BA.5 in adolescents who are baseline SARS-CoV-2 positive in Part 3 of Study P203 compared to that of post primary series of mRNA-1273 (post Dose 2 [Day 57] of 100 µg) in adults 18 to 25 years of age in Study P301.<br>• SRR of post Dose 1 (Day 29) from baseline (pre Dose 1) against ancestral strain in adolescents who are baseline SARS-CoV-2 positive in Part 3 of Study P203 compared to that of post primary series of mRNA-1273 (post Dose 2 [Day 57] of 100 µg) in adults 18 to 25 years of age in Study P301.<br>• GM value of post Dose 2 (Day 209) of mRNA-1273.222 Ab against Omicron BA.4/BA.5 in Part 3 of Study P203 compared to that of post primary series of mRNA-1273 (post Dose 2 [Day 57] of 100 µg) in adults 18 to 25 years of age in Study P301.<br>• GM value of post Dose 2 (Day 209) of mRNA-1273.222 Ab against ancestral strain in Part 3 of Study P203 compared to that of post primary series of mRNA-1273 (post Dose 2 [Day 57] of 100 µg) in adults 18 to 25 years of age in Study P301.<br>• SRR of post Dose 2 (Day 209) from baseline (pre Dose 1) against Omicron BA.4/BA.5 in Part 3 of Study P203 compared to that of post primary series of mRNA-1273 (post Dose 2 [Day 57] of 100 µg) in adults 18 to 25 years of age in Study P301.<br>• SRR of post Dose 2 (Day 209) from baseline (pre Dose 1) against ancestral strain in Part 3 of Study P203 compared to that of post primary series of mRNA-1273 (post Dose 2 [Day 57] of 100 µg) in adults 18 to 25 years of age in Study P301.<br><br>*Note: Seroresponse is defined as an Ab value change from baseline (pre Dose 1) below the LLOQ to ≥4 × LLOQ, or at least a 4-fold rise if baseline is ≥LLOQ*

• To evaluate immune response elicited by 50 µg mRNA-1273.222 vaccine administered in 2 doses 6 months apart against other variant(s) of interest obtained 28 days post Dose 1 or 28 days post Dose 2.	• GM value of post Dose 1 (Day 29) of mRNA-1273.222 Ab against other variant(s) of interest in Part 3 of Study P203 as compared to that of post primary series of mRNA-1273 (post Dose [Day 57] 2 of 100 µg) in adults 18 to 25 years of age in Study P301.<br>• GM value of post Dose 2 (Day 209) of mRNA-1273.222 Ab against other variant(s) of interest in Part 3 of Study P203 as compared to that of post primary series of mRNA-1273 (post Dose 2 [Day 57] of 100 µg) in adults 18 to 25 years of age in Study P301.
**Exploratory Objectives**	**Exploratory Endpoints**
• To evaluate the persistence of the immune response of 50 µg mRNA-1273.222 vaccine, as assessed by the level of SARS-CoV-2 S2P specific bAb through 6 months after Dose 2.	• The GM values of SARS-CoV-2 S2P specific bAb on Day 1, Day 29 (1 month after Dose 1), Day 85 (3 months after Dose 1), Day 181 (Dose 2), Day 209 (1 month after Dose 2) and Day 361 (6 months after Dose 2).
• To evaluate the persistence of the immune response of the 50 µg mRNA-1273.222 vaccine as assessed by the level of nAb through 6 months after Dose 2.	• The GM values of SARS-CoV-2-specific nAb on Day 1, Day 29 (1 month after Dose 1), Day 85 (3 months after Dose 1), Day 181 (Dose 2), and Day 209 (1 month after Dose 2), and Day 361 (6 months after Dose 2).
• To evaluate the incidence COVID-19 after vaccination with mRNA-1273.222.	• The incidence of the first occurrence of symptomatic COVID-19 starting 14 days after Dose 1 and Dose 2 of mRNA-1273.222.

Abbreviations: Ab = antibody; AE = adverse event; AESI = adverse event of special interest; AR = adverse reaction; bAb = binding antibody; COVID-19 = coronavirus disease 2019; GM = geometric mean; LLOQ = lower limit of quantification; MAAE = medically attended adverse event; nAb = neutralizing antibody; RT-PCR = reverse transcriptase polymerase chain reaction; S2P = S protein; SAE = serious adverse event; SARS-CoV-2 = Severe Acute Respiratory Syndrome coronavirus 2; SRR = seroresponse rate; VOC = variant of concern.

Part 1 Overall Study Design

This is a three-part, Phase 2/3, study: Part 1A, Part 1B, and Part 1C. The study will evaluate the safety, reactogenicity, and effectiveness of mRNA-1273 SARS-CoV-2 vaccine in healthy adolescents 12 to <18 years of age.

Participants in Part 1A, the Blinded Phase, will be randomly assigned to receive injections of either 100 µg mRNA-1273 vaccine or a placebo control in a 2:1 randomization ratio. Part 1B, the Open-label Observational Phase of this study, is designed to offer participants who received placebo in Part 1A of this study, and who meet Emergency Use Authorization (EUA) eligibility criteria, an option to receive mRNA-1273 in an open-label fashion. Part 1C-1, the Homologous Booster Dose Phase, is designed to offer participants in Part 1A and Part 1B who are at least 5 months from the last dose, the option to request a booster dose (BD) (50 µg) of mRNA-1273. Part 1C-2, the Heterologous Booster Phase, is

designed to provide a BD (50 µg) of mRNA-1273 to eligible participants who completed primary coronavirus disease 2019 [COVID-19] vaccination series with a non-Moderna vaccine under EUA and are at least 3 months from the last dose.

The goal of the study is to seek an indication for use of mRNA-1273 (100 µg intramuscular [IM], given as 2 injections, 28 days apart) in the 12 to < 18 years age group. The basis for demonstrating vaccine effectiveness is proposed to be met by serum antibody (Ab) response measured in this adolescent age group. The approach to inferring vaccine effectiveness will depend on whether an accepted serum Ab threshold conferring protection against COVID-19 has been established. If an Ab threshold of protection has been established, effectiveness will be inferred based on the proportion of adolescent study participants with serum Ab levels (on Day 57) that meet or exceed the Ab threshold. If an Ab threshold of protection has not been established, effectiveness will be inferred by demonstrating noninferiority of both the (i) geometric mean (GM) value of serum neutralizing antibody (nAb) and (ii) the seroresponse rate (SRR) from adolescent participants compared with those from young adults (18 to 25 years of age) enrolled in the ongoing clinical endpoint efficacy trial (Study P301).

Part 1A, the Blinded Phase - Randomized, Observer-Blind, Placebo-Controlled

This study in adolescents will monitor all participants for a total of 12 months following the second dose of vaccine or placebo. Safety assessments will include solicited ARs; 7 days after each injection), unsolicited AEs; 28 days after each injection, medically attended adverse events (MAAEs), serious adverse events (SAEs), and adverse events of special interest (AESIs) including multisystem inflammatory syndrome in children [MIS-C]) throughout the study period.

Blood samples will be collected from all participants at baseline (Day 1), Day 57 (28 days after Dose 2), Day 209 (6 months after Dose 2), and Day 394 for measurement of Severe Acute Respiratory Syndrome coronavirus 2 (SARS-CoV-2) specific binding antibody (bAb) and nAb responses. Blood samples will also be tested for the development of Ab directed against nonvaccine antigen (eg, Ab against the nucleocapsid protein), which will signify infection with SARS-CoV-2.

The incidence of SARS-CoV-2 infection among vaccine recipients and placebo recipients will be compared to assess the potential for mRNA-1273 to reduce the rate of infection in vaccine recipients.

Part 1A comprises 8 scheduled visits including a Screening Visit and 7 scheduled visits, of which Visit 2 and Visit 4 will be virtual/telephone visits and the other visits will be in-clinic visits. This study will be conducted in compliance with the protocol, Good Clinical Practice (GCP), and all applicable regulatory requirements.

Part 1B, the Open-label Observational Phase

Part 1B, the Open-label Observational Phase of the study, will be prompted by the authorization of a COVID-19 vaccine under an EUA for any persons under the age of 18 years. Participants will be transitioned to Part 1B of the study as their age group becomes EUA-eligible. This transition permits all ongoing study participants to eventually be informed of the availability and eligibility criteria of any COVID-19 vaccine made available under an EUA and the option to offer all ongoing study participants an opportunity to schedule a Participant Decision Visit to know their original treatment assignment (placebo vs. mRNA-1273 100 µg vaccine).

Part 1B provides the opportunity for study participants to be informed regarding the EUA, be unblinded to their original assignment (mRNA-1273 or placebo), and for those who previously received placebo to actively request to receive 2 doses of mRNA-1273 (100 µg) vaccine. EUA-eligible

study participants will receive a notification letter summarizing the basis for EUA of a COVID-19 vaccine to receive an EUA and will be asked to schedule a Participant Decision Clinic Visit.

At the Participant Decision Clinic Visit, EUA-eligible participants will:

- Be given the option to be unblinded as to their original group assignment (placebo vs. mRNA-1273 vaccine [100 µg]),
- Be counseled about the importance of continuing other public health measures to limit the spread of disease including social distancing, wearing a mask, and hand-washing,
- Sign a revised informed consent form (ICF) and assent, and
- Provide a nasopharyngeal (NP) or nasal swab for RT-PCR for SARS-CoV-2 and a blood sample for serology and immunogenicity.

Participants that decline unblinding will remain in Part 1A and follow the Part 1A schedule of assessment (SoA).

After the Participant Decision Clinic Visit, participants that consent to unblinding will follow the Part 1A SoA or Part 1B SoA as follows:

- Participants received placebo in Part 1A and consent to receiving 2 doses of mRNA-1273 in Part 1B: These participants will proceed to Part 1B and follow the Part 1B SoA until BD is offered.
- Participants received 2 doses of mRNA-1273 in Part 1A: Due to statistical considerations, these participants will be considered in the Open-label Observational Phase but will continue to follow the Part 1A SoA until BD is offered.

Part 1C-1, Homologous Booster Dose Phase

Part 1C-1 – Homologous Booster Dose Phase is designed to offer participants in Part 1A and Part 1B who are at least 5 months from the last dose, the option to request a BD (50 µg) of mRNA-1273.

If eligible, each study participant will receive a notification letter and will be asked to schedule a BD-1 Visit at their study site. Principal investigators should consider current local public health guidance for administration of COVID-19 vaccines under EUA and marketing authorization (if any) when determining the scheduling priority of participants.

At the BD-1 Visit, each participant will:

- Be encouraged to remain in the ongoing study,
- Sign a revised ICF that includes both updated safety information relevant to the ongoing study and a BD, and the option to receive a BD,
- Be given the option to receive a BD consisting of a 50 µg dose of mRNA-1273,
- Be counseled about the importance of continuing other public health measures to limit the spread of disease including social distancing, wearing a mask, and hand-washing.

At the BD-1 Visit, participants who request a BD and are eligible will have the following study site visits and complete scheduled activities (subject to investigational vaccine availability) according to the Part 1C-1 Supplemental SoA:

- BD-1 Visit: Participants will receive a single 50 µg dose of mRNA-1273,
- BD-1a Visit: All participants who chose to receive BD, Day 4, 3 days after BD on Day 1,
- BD-2 Visit: Day 29, 28 days after the BD on Day 1,
- BD-3 Visit: Day 181, 180 days after the BD on Day 1,
- BD-4 Visit: Day 361, 360 days after the BD on Day 1.

Participants that crossed over from placebo to mRNA-1273 and decline BD will continue with Part 1B SoA.

Participants that received mRNA-1273 in Part 1A and decline BD will continue with Part 1A SoA.

The investigator is responsible for conducting all assessments as specified in the Part 1C-1 Supplemental SoA, according to the schedule. As this Supplemental SoA is intended to occur in addition to the original SoAs being followed by all participants in Part 1A or Part 1B, there is a possibility for study visits to overlap (ie, Part 1C-1 BD-Day 1 and study visit in Part 1A or Part 1B that coincides with Part 1C-1 BD-Day 1). If visits overlap according to respective visit windows, a single visit may be done with the combined study procedures completed once. Any subject who consented to receive a BD will follow Part 1C-1 SoA. In case a study visit in Part 1A or Part 1B coincides with Part 1C-1 BD-Day 1, participant should follow Part 1C-1 SoA.

Part 1C-2, - Heterologous Booster Phase

Part 1C-2- Heterologous Booster Phase is designed to provide a BD (50 µg) of mRNA-1273 to eligible participants who completed primary COVID-19 vaccination series with a non-Moderna vaccine under EUA (ie. Pfizer) and are at least 3 months from the last dose.

Non-Moderna COVID-19 Vaccine Available Under Emergency Use Authorization

**COVID-19 Vaccine – Primary Series**	**Primary Series Number of Doses**
Pfizer-BioNTech	2 doses

Abbreviations: COVID-19 = coronavirus disease 2019.

Principal investigators should consider current local public health guidance for administration of COVID-19 vaccines under EUA and marketing authorization (if any) when determining the scheduling priority of participants.

At the BD-0/BD-1 Visit, each participant will:

- Sign an ICF,
- Be counseled about the importance of continuing other public health measures to limit the spread of disease including social distancing, wearing a mask, and hand-washing.

At the BD-1 Visit, participants will have the following study site visits and complete scheduled activities (subject to investigational vaccine availability) according to the Part 1C-2 Supplemental SoA:

- BD-1 Visit: Participants will receive a single 50 µg dose of mRNA-1273,
- BD-1a Visit: Day 4, 3 days after BD on Day 1,

- BD-2 Visit: Day 29, 28 days after the BD on Day 1,
- BD-3 Visit: Day 181, 180 days after the BD on Day 1,
- BD-4 Visit: Day 361, 360 days after the BD on Day 1.

The investigator is responsible for conducting all assessments as specified in the Part 1C-2 Supplemental SoA, according to the schedule.

Part 2 Study Design

Part 2 is an open-label design. The study will evaluate the safety, reactogenicity, and effectiveness of a 50 µg primary series of mRNA-1273 SARS-CoV-2 vaccine in healthy adolescents 12 to < 18 years of age.

This study in adolescents will monitor all participants for a total of 12 months following Dose 2 of vaccine. Safety assessments will include solicited ARs (7 days after each injection), unsolicited AEs (28 days after each injection), MAAEs, SAEs, and AESIs (including MIS-C) throughout the study period, and AEs leading to discontinuation from study participation.

Blood samples will be collected from all participants at baseline (Day 1), Day 29, and at specified visits presented in Part 2 SoA for measurement of SARS-CoV-2 specific bAb and nAb responses. Blood samples will also be tested for the development of Ab directed against nonvaccine antigen (eg, Ab against the nucleocapsid protein), which will signify infection with SARS-CoV-2. Blood sample will be collected 3 days after Dose 2 for future biomarker analysis (ie, inflammatory and/or cardiac biomarkers).

The incidence of SARS-CoV-2 infection after mRNA-1273 vaccination will be evaluated.

Part 2 comprises 11 scheduled visits including a Screening Visit and 10 scheduled visits, of which Visit 2, Visit 5, and Visit 7 will be virtual/telephone visits and the other visits will be in-clinic visits.

This study will be conducted in compliance with the protocol, GCP, and all applicable regulatory requirements.

Part 3 Study Design

Part 3 is an open-label, single-arm design. The study will evaluate the safety, reactogenicity, and effectiveness of a 2-dose 50 µg primary series of mRNA-1273.222 SARS-CoV-2 vaccine, administered 6 months apart, in approximately at least 300 healthy adolescents 12 to <18 years of age.

This study in adolescents will monitor all participants for 6 months following Dose 2 of vaccine. Safety assessments will include solicited ARs (7 days after each injection), unsolicited AEs (28 days after each injection), MAAEs, SAEs, and AESIs (including MIS-C) throughout the study period and AEs leading to discontinuation from dosing and/or withdrawal from study.

Blood samples will be collected from all participants at baseline (Day 1), Day 29 (28 days after Dose 1), Day 85 (3 months after Dose 1), Day 181 (Dose 2), Day 209 (1 month after Dose 2), and Day 361 (6 months after Dose 2) for measurement of SARS-CoV-2 specific bAb and nAb responses. Blood samples will also be tested for the development of Ab directed against nonvaccine antigen (eg, Ab against the nucleocapsid protein), which will signify infection with SARS-CoV-2. Blood samples will be collected 3 days after Dose 1 and 3 days after Dose 2 (Day 184), for future biomarker analysis (ie, inflammatory and/or cardiac biomarkers).

The incidence of SARS-CoV-2 infection after mRNA-1273.222 vaccination will be evaluated.

Safety Oversight:

Safety oversight will be under the direction of a Data Safety Monitoring Board (DSMB) composed of external independent consultants with relevant expertise.

An independent cardiac event adjudication committee (CEAC) of medically qualified personnel, including cardiologists, will review all suspected cases of myocarditis and pericarditis to determine if they meet CDC criteria of "probable" or "confirmed" events which are reported in ongoing interventional clinical trials per the CEAC charter. Any case that the CEAC assesses as representing probable or confirmed cases of myocarditis or pericarditis will be referred to the Sponsor, who will then determine if additional action is needed.

The CEAC will operate under the rules of an approved charter that will be written and reviewed at the organizational meeting of the CEAC. Details regarding the CEAC composition, responsibilities, procedures, and frequency of data review will be defined in its charter.

The contract research organization (CRO's) medical monitor, the Sponsor's medical monitor, and the individual study site investigators will monitor safety throughout the study.

Study Duration:

Part 1

Participants who received mRNA-1273 in Part 1A will be in the study approximately 25 months total if a BD is received in Part 1C-1, which includes 1 month for Screening (Day -28 to Day 1), up to 12 months for dosing (on Day 1, Day 29, and Day 209 for BD), and 12 months for follow-up.

Participants who received placebo in Part 1A will be in the study for approximately 25 months total if BD is received in Part 1C-1, which includes approximately 9 months in Part 1A, approximately 4 months of follow-up following their second dose of mRNA-1273 in Part 1B before entering Part 1C-1 or before BD is received; or approximately 12 months of follow up.

Participants that decline unblinding or decline to receive a BD will be in the study approximately 14 months total, which includes 1 month for Screening (Day -28 to Day 1), 1 month for dosing (on Day 1 and Day 29), and 12 months for follow-up.

Participants in Part 1C-2 will be in the study for approximately 12 months total, which includes 7 days for Screening (Day -7 to Day 1), 1 day of dosing (BD-Day 1), and 12 months of follow-up.

Part 2

Participants who received mRNA-1273 in Part 2 will be in the study approximately 13 months, which includes 1 week for Screening (Day -7 to Day 1), 1 month for dosing (Day 1 to Day 29), and 12 months of follow-up after Dose 2.

Part 3

Participants who receive mRNA-1273.222 in Part 3 will be in the study for approximately 12 months including 1 week for Screening (Day -7 to Day -1), 6 months for dosing (Day 1 to Day 181), and 6 months of follow-up after Dose 2.

Number of Participants:

Part 1A (Blinded Phase) – Approximately 3,000 participants will be enrolled.

Part 1C-2 (Heterologous Booster Phase) – Approximately 362 participants will be enrolled.

Part 2 – Approximately 362 participants will be enrolled.

Part 3 – Approximately at least 300 participants will be enrolled.

Study Eligibility Criteria (Part 1A, Part 2, and Part 3):

Inclusion Criteria:

Each participant must meet all of the following criteria at the Screening Visit (Day 0) or at Day 1, unless noted otherwise, to be enrolled in this study:

1. Male or female, 12 to < 18 years of age at the time of consent (Screening Visit, Day 0) who, in the opinion of the investigator, is in good general health based on review of medical history and Screening physical examination.
2. Investigator assessment that the participant, in the case of an emancipated minor, or parent(s)/legally accepted representative (LAR) understand and are willing and physically able to comply with protocol-mandated follow-up, including all procedures and provides written informed consent/assent.
3. Body mass index (BMI) at or above the third percentile according to WHO Child Growth Standards at the Screening Visit (Day 0).
4. Female participants of nonchildbearing potential may be enrolled in the study. Nonchildbearing potential is defined as premenarche or surgically sterile (history of bilateral tubal ligation, bilateral oophorectomy, hysterectomy).
5. Female participants of childbearing potential may be enrolled in the study if the participant fulfills all the following criteria:
   - Has a negative pregnancy test at Screening (Day 0), on the day of the first injection (Day 1), and on the day of the second injection (Day 29 in Part 1 A and Part 2, and Day 181 in Part 3).
   - Has practiced adequate contraception or has abstained from all activities that could result in pregnancy for at least 28 days prior to the first injection (Day 1).
6. Has agreed to continue adequate contraception or abstinence through 3 months following the second injection (Day 29 in Part 1 A and Part 2, and Day 181 in Part 3).

Adequate female contraception is defined as consistent and correct use of approved contraceptive method in accordance with the product label.

Exclusion Criteria:

Participants who meet any of the following criteria at the Screening Visit (Day 0) or at Day 1, unless noted otherwise, will be excluded from the study:

1. Has a known history of SARS-CoV-2 infection within 2 weeks prior to administration of investigational product (IP) or known close contact with anyone with laboratory-confirmed

SARS-CoV-2 infection of COVID-19 within 2 weeks prior to administration of IP (Part 2 participants only). For Part 3 participants, known history of SARS-CoV-2 infection within 90 days prior to administration of IP or known close contact with anyone with laboratory-confirmed SARS-CoV-2 infection or COVID-19 within 90 days prior to administration of IP.

2. Travel outside of the USA or home country (Part 2 and Part 3 only) in the 28 days prior to the Screening Visit (Day 0).
3. Pregnant or breastfeeding.
4. Is acutely ill or febrile 24 hours prior to or at the Screening Visit (Day 0). Fever is defined as a body temperature ≥ 38.0°C/≥ 100.4°F. Participants who meet this criterion may have visits rescheduled within the relevant study visit windows. Afebrile participants with minor illnesses can be enrolled at the discretion of the investigator.
5. Prior administration of an investigational CoV (eg, SARS-CoV-2, Middle East Respiratory Syndrome [MERS]-CoV) vaccine.
6. Current treatment with investigational agents for prophylaxis against COVID-19.
7. Has a medical, psychiatric, or occupational condition that may pose additional risk as a result of participation, or that could interfere with safety assessments or interpretation of results according to the investigator's judgment.
8. Current use of any inhaled substance (eg, tobacco or cannabis smoke, nicotine vapors).
9. History of chronic smoking (≥ 1 cigarette a day) within 1 year of the Screening Visit (Day 0).
10. History of illegal substance use or alcohol abuse within the past 2 years. This exclusion does not apply to historical cannabis use that was formerly illegal in the participant's state but is legal at the time of Screening.
11. History of a diagnosis or condition that, in the judgment of the investigator, may affect study endpoint assessment or compromise participant safety, specifically:
    - Congenital or acquired immunodeficiency, including human immunodeficiency virus (HIV) infection.
    - Suspected active hepatitis.
    - Has a bleeding disorder that is considered a contraindication to IM injection or phlebotomy.
    - Dermatologic conditions that could affect local solicited AR assessments.
    - History of anaphylaxis, urticaria, or other significant AR requiring medical intervention after receipt of a vaccine.
    - Diagnosis of malignancy within the previous 10 years (excluding nonmelanoma skin cancer).
    - Febrile seizures.
12. Receipt of:

- Any licensed vaccine within 28 days before the first dose of IP or plans for receipt of any licensed vaccine within 28 days before and/or after each dose of IP.
- Systemic immunosuppressants or immune-modifying drugs for > 14 days in total within 6 months prior to the day of enrollment (for corticosteroids, ≥ 20 mg/day prednisone equivalent). Topical tacrolimus is allowed if not used within 14 days prior to the day of enrollment. Participants may have visits rescheduled for enrollment if they no longer meet this criterion within the Screening Visit window. Inhaled, nasal, and topical steroids are allowed.
- Intravenous blood products (red cells, platelets, immunoglobulins) within 3 months prior to enrollment.

13. Has donated ≥450 mL of blood products within 28 days prior to the Screening Visit (Day 0) or plans to donate blood products during the study.
14. Participated in an interventional clinical study within 28 days prior to the Screening Visit (Day 0) or plans to do so while participating in this study.
15. Is an immediate family member or has a household contact who is an employee of the research center or otherwise involved with the conduct of the study.

Study Eligibility Criteria (Part 1B):

1. Participants must have been previously enrolled in the mRNA-1273-P203 study.
2. Female participants of childbearing potential may be enrolled in the study if the participant has a negative pregnancy test on the day of the first injection (OL-Day 1) and on the day of the second injection (OL-Day 29).

Study Eligibility Criteria (Part 1C-1- Homologous Booster Dose):

Inclusion Criteria:

1. Participants must have been previously enrolled in the mRNA-1273-P203 study, are actively participating in Part 1A or Part 1B, and are at least 5 months from the last dose.
2. Female participants of childbearing potential may be enrolled in the study if the participant has a negative pregnancy test on the day of the first injection (BD-Day 1).

Exclusion Criteria:

1. Pregnant or breastfeeding.
2. Is acutely ill or febrile 24 hours prior to or at the Screening Visit (Day 0). Fever is defined as a body temperature ≥38.0°C/≥100.4°F. Participants who meet this criterion may have visits rescheduled within the relevant study visit windows. Afebrile participants with minor illnesses can be enrolled at the discretion of the investigator.
3. Has a medical, psychiatric, or occupational condition that may pose additional risk as a result of participation, or that could interfere with safety assessments or interpretation of results according to the investigator's judgment.
4. History of a diagnosis or condition (after enrollment in Part 1A) that, in the judgment of the investigator, may affect study endpoint assessment or compromise participant safety:

- Suspected active hepatitis.
- Has a bleeding disorder that is considered a contraindication to IM injection or phlebotomy.
- Dermatologic conditions that could affect local solicited AR assessments.
- History of anaphylaxis, urticaria, or other significant AR requiring medical intervention after receipt of a vaccine.
- Diagnosis of malignancy (excluding nonmelanoma skin cancer).

5. Receipt of:
   - Any authorized or licensed vaccine within 28 days before the first dose of IP or plans for receipt of any licensed vaccine through 28 days following the last dose of IP (authorized/approved bivalent vaccine against SARS-CoV-2 as 4th BD is not exclusionary); OR
   - Any seasonal influenza vaccine within 14 days before the first dose of IP or plans for receipt of any seasonal influenza vaccine 14 days following the last dose of IP.
6. Participated in an interventional clinical study, other than mRNA-1273-P203 study, within 28 days prior to the Screening Visit (Day 0) or plans to do so while participating in this study.

Study Eligibility Criteria (Part 1C-2 – Heterologous Booster Dose):

Inclusion Criteria:

1. Male or female, 12 to < 18 years of age at the time of consent who, in the opinion of the investigator, is in good general health based on review of medical history and Screening physical examination AND has completed non-Moderna primary COVID-19 vaccination series under EUA (ie, Pfizer) at least 3 months from consent.
2. Investigator assessment that the participant, in the case of an emancipated minor, or parent(s)/LAR understand and are willing and physically able to comply with protocol-mandated follow-up, including all procedures and provides written informed consent/assent.
3. BMI at or above the third percentile according to WHO Child Growth Standards at the Screening Visit (BD-Day 0).
4. Female participants of nonchildbearing potential may be enrolled in the study. Nonchildbearing potential is defined as premenarche or surgically sterile (history of bilateral tubal ligation, bilateral oophorectomy, hysterectomy). Female participants of childbearing potential may be enrolled in the study if the participant has a negative pregnancy test on the day of the first injection (BD-Day 1) and has agreed to continue adequate contraception or abstinence through 3 months following BD (BD-Day 1).

Exclusion Criteria:

1. Has a known history of SARS-CoV-2 infection within 2 weeks prior to administration of IP or known close contact with anyone with laboratory-confirmed SARS-CoV-2 infection or COVID-19 within 2 weeks prior to administration of IP.

2. Pregnant or breastfeeding.
3. Is acutely ill or febrile 24 hours prior to or at the Screening Visit (BD-Day 0). Fever is defined as a body temperature ≥38.0°C/≥100.4°F. Participants who meet this criterion may have visits rescheduled within the relevant study visit windows. Afebrile participants with minor illnesses can be enrolled at the discretion of the investigator.
4. Has a medical, psychiatric, or occupational condition that may pose additional risk as a result of participation, or that could interfere with safety assessments or interpretation of results according to the investigator's judgment.
5. History of a diagnosis or condition that, in the judgment of the investigator, may affect study endpoint assessment or compromise participant safety:
   - Suspected active hepatitis
   - Has a bleeding disorder that is considered a contraindication to IM injection or phlebotomy
   - Dermatologic conditions that could affect local solicited AR assessments
   - History of anaphylaxis, urticaria, or other significant AR requiring medical intervention after receipt of a vaccine
   - Diagnosis of malignancy (excluding nonmelanoma skin cancer)
6. Receipt of:
   - Any authorized or licensed vaccine within 28 days before the first dose of IP (monovalent COVID-19 primary series vaccines are not exclusionary) or plans for receipt of any licensed vaccine through 28 days following the last dose of IP (monovalent COVID-19 primary series vaccines as well as authorized/approved bivalent vaccine against SARS-CoV-2 as 4th booster are not exclusionary) or any seasonal influenza vaccine within 14 days before the first dose of IP or plans for receipt of any seasonal influenza vaccine 14 days following the last dose of IP.
7. Participated in an interventional clinical study, other than mRNA-1273-P203 study, within 28 days prior to the Screening Visit (BD-Day 0) or plans to do so while participating in this study.

Study Treatment:

Investigational Product:

The IP (mRNA-1273 vaccine) is a lipid nanoparticle (LNP) dispersion of a mRNA encoding the prefusion stabilized spike (S) protein of SARS-CoV-2 formulated in LNPs composed of 4 lipids (1 proprietary and 3 commercially available): the proprietary ionizable lipid SM-102; cholesterol; 1,2-distearoyl-sn-glycero-3-phosphocholine (DSPC); and 1-monomethoxypolyethyleneglycol-2,3-dimyristylglycerol with PEG2000-DMG. mRNA-1273 injection is provided as a sterile liquid for injection, white to off-white dispersion in appearance, at a concentration of 0.5 mg/mL in 20 mM Tris buffer containing 87 mg/mL sucrose and 10.7 mM sodium acetate at pH 7.5 For Part 1A and Part 1B, each injection will have a volume of 0.5 mL and contain mRNA-1273 100 µg. For Part 1C-1, Part 1C-2, and Part 2, each injection will have a volume of 0.25 mL and contain mRNA-1273 50 µg.

The Sponsor initiated the development of modified COVID-19 vaccine leveraging the mRNA/LNP technology platform against SARS-CoV-2 variant, which incorporates key mutations present in Omicron subvariants BA.4/BA.5. The vaccine IP, mRNA-1273.222, contains 2 mRNAs: CX-024414 encoding for the S-2P of Wuhan-Hu-1 and CX-034476 encoding for the S-2P of Omicron subvariants BA.4/BA.5.

mRNA-1273.222 will be provided as a sterile solution for injection at a concentration of 0.1 mg/mL in 20 mM Tris buffer with sucrose, at pH 7.5. The solution will be kept in 2R USP Type I borosilicate glass vials with PLASCAP vial seal containing a 13 mm FluroTec-coated plug stopper with a 0.8 mL nominal fill volume. For Part 3, each injection preparation will have a volume of 0.5 mL and contain 50 µg mRNA-1273.222.

Mode of Administration:

Doses will be administered by IM injection into the deltoid muscle according to the procedures specified in the mRNA-1273-P203 Pharmacy Manual. Preferably, all doses should be administered into the nondominant arm.

Procedures and Assessments:

Safety Assessments:

Safety assessments will include monitoring and recording of the following for each participant:

- Solicited local and systemic ARs that occur during the 7 days following each injection (ie, the day of injection and 6 subsequent days) in Part 1A, Part 1C, Part 2, and Part 3. Solicited ARs will be recorded daily using electronic diaries (eDiaries)
- Unsolicited AEs observed or reported during the 28 days following each injection (ie, the day of injection and 27 subsequent days) in Part 1A, Part 1C, Part 2, and Part 3
- AEs leading to discontinuation from dosing and/or withdrawal from study participation from Day 1 through the last day of study participation (ie, Day 394 per Part 1A SoA, OL-Day 178 in Part 1B, BD-Day 361 in Part 1C, Day 394 in Part 2, and Day 361 in Part 3)
- MAAEs from first dose on Day 1 through the entire study period (ie, Day 394 per Part 1A SoA, OL-Day 178 in Part 1B, BD-Day 361 in Part 1C, Day 394 in Part 2, and Day 361 in Part 3)
- SAEs from first dose on Day 1 through the entire study period (ie, Day 394 per Part 1A SoA, OL-Day 178 in Part 1B, BD-Day 361 in Part 1C, Day 394 in Part 2, and Day 361 in Part 3)
- AESIs through the entire study period (ie, Day 394 per Part 1A SoA, OL-Day 178 in Part 1B, BD-Day 361 in Part 1C, Day 394 in Part 2, and Day 361 in Part 3)
- Vital sign measurements
- Physical examination findings

- Assessments for SARS-CoV-2 infection from Day 1 through study completion
- Details of all pregnancies in female participants will be collected after the start of study treatment and until the end of their participation in the study

Immunogenicity Assessments:

The following analytes will be measured in blood samples for immunogenicity assessments:

- Serum nAb level against SARS-CoV-2 as measured by pseudovirus and/or live virus neutralization assays
- Serum bAb levels against SARS-CoV-2 as measured by a multiplex serology assay specific to the SARS-CoV-2 S, N, RBD protein.

Serum collected from all participants will be tested for bAb against SARS-CoV-2 nucleocapsid protein at specified timepoints. In addition, serum samples from a selected subset of study participants who received mRNA-1273 or mRNA-1273.222 will be selected for testing of nAb and bAb against the SARS-CoV-2 S protein.

Exploratory Assessments and Biomarkers:

Exploratory assessments may include assessment of biomarkers for safety, reactogenicity, inflammatory and cardiac function. Serologic markers of disease severity, immune response to SARS-CoV-2, RT-PCR of NP or nasal swab or saliva samples, and genetic sequences of SARS-CoV-2 strains isolated from participants' samples may also be measured.

Efficacy Assessments:

Vaccine effectiveness for adolescents of ages of 12 to < 18 years will be inferred based on serum Ab responses obtained on Day 57 (28 days after the second injection of mRNA-1273) and Day 209 (28 days after the second injection of mRNA1273.222). Inference will be based on assessing the adolescent Ab responses against the following:

- *If available at the time of analysis*, adolescent Ab responses will be assessed against an accepted serum Ab threshold conferring protection against COVID-19.
- *If an accepted threshold of protection is not available*, adolescent Ab responses will be assessed by establishing noninferiority of the GM value and SRR of serum nAb from adolescent participants compared with those from young adults enrolled in the ongoing clinical endpoint efficacy trial (Study P301).

Statistical Methods:

Part 1

Hypothesis Testing (Part 1A)

If an accepted serum Ab threshold of protection against COVID-19 is established for the primary immunogenicity objective, the null hypothesis is that the percentage of participants on mRNA-1273 with serum Ab equal to or above the established threshold at Day 57 is ≤70% (ie, H_0: percentage of participants on mRNA-1273 ≤70% with serum Ab at Day 57 equal to or above the established threshold).

The study would be considered as meeting the immunogenicity objective if the 95% confidence interval (CI) of percentage of participants on mRNA-1273 rules out 70% (lower bound of the 95% CI >70%).

If an accepted serum Ab threshold of protection against COVID-19 is not available for the primary immunogenicity objective, the immunogenicity analysis of primary vaccine response will be performed using the noninferiority tests of the 2 null hypotheses based on the 2 coprimary endpoints, respectively.

Coprimary endpoint 1: Ab GM value at Day 57

H^1_0: immunogenicity response to mRNA-1273 as measured by Ab GM at Day 57 is inferior in adolescents (12 to < 18 years of age) compared with that in young adults (18 to 25 years of age) using mRNA-1273 Study P301 data.

The noninferiority in Ab GM in adolescents compared with that in young adults (18 to 25 years of age) is demonstrated by that the lower bound of the 95% CI of the GM ratio (GMR) rules out 0.667 (lower bound >0.667) using a noninferiority margin of 1.5. The GMR is the ratio of the GM value of adolescents on mRNA-1273 in this study, Study P203, at Day 57 compared with the GM value of young adults (18 to 25 years of age) on mRNA-1273 in Study P301.

Coprimary endpoint 2: Ab seroresponse rate at Day 57

A definition of seroresponse will be provided in the SAP based on forthcoming information about assay performance.

The null hypothesis:

H^2_0: immunogenicity response to mRNA-1273 as measured by seroresponse rate at Day 57 is inferior in adolescents (12 to <18 years of age) compared with that in young adults (18 to 25 years of age) using mRNA-1273 Study P301 data.

The noninferiority in seroresponse rate in adolescents compared with that in young adults (18 to 25 years of age) is demonstrated by that the lower bound of the 95% CI of the seroresponse rate difference rules out -10% (ie, lower bound >-10%) using the noninferiority margin of 10%. The seroresponse rate difference is defined as the rate in adolescents receiving mRNA-1273 minus the rate in young adults (18 to 25 years of age) receiving mRNA-1273 from Study P301.

The study would be considered as meeting the primary immunogenicity objective if noninferiority is demonstrated based on both coprimary endpoints.

Details regarding the assay to be used to assess noninferiority will be provided in the SAP.

Power and Sample Size

The sample size of this study is driven by safety. Approximately 3,000 participants will be randomly assigned in a 2:1 ratio to receive mRNA-1273 or placebo. With 2,000 participants exposed to mRNA-1273, the study has at least 90% probability to observe at least 1 participant with an AE at a true 0.25% AE rate.

Serum samples from all participants will be collected and banked, a subset of participants will be selected, and their samples will be processed for immunogenicity testing (the Immunogenicity Subset).

Approximately 362 participants who receive mRNA-1273 will be selected for the Immunogenicity Subset, with a target of 289 participants receiving mRNA-1273 in the per-protocol (PP)

Immunogenicity Subset (adjusting for approximately 20% of participants who may be excluded from the PP Immunogenicity Subset, as they may not have immunogenicity results due to any reason). The sample size of the Immunogenicity Subset may be updated with data from other mRNA-1273 studies or external data especially regarding a threshold of protection. In such a situation, the final sample size of the Immunogenicity Subset will be documented in the SAP.

For the primary immunogenicity objective, with approximately 289 participants in the PP Immunogenicity Subset, the study will have > 90% power to rule out 70% with a 2-sided 95% CI for the percentage of mRNA-1273 participants exceeding the acceptable threshold if the true rate of participants exceeding the acceptable threshold is 80%.

If an acceptable Ab threshold of protection against COVID-19 is not available at the time of analysis, for the primary immunogenicity objective, noninferiority tests of 2 null hypotheses based on 2 coprimary endpoints, respectively, will be performed. The sample size calculation for each of the 2 noninferiority tests was performed, and the larger sample size was chosen for the study.

- With approximately 289 participants in the PP Immunogenicity Subset in Study P203 and 289 participants in the PP Immunogenicity Subset in young adults (18 to 25 years of age) from Study P301, there will be 90% power to demonstrate noninferiority of the immune response as measured by Ab GM in adolescents in Study P203 at a 2-sided α of 0.05, compared with that in young adults (18 to 25 years of age) from Study P301 receiving mRNA-1273, assuming an underlying GMR value of 1 and a noninferiority margin of 1.5. The SD of the log-transformed levels is assumed to be 1.5.
- With approximately 289 participants in the PP Immunogenicity Subset in Study P203 and 289 participants in the PP Immunogenicity Subset in young adults (18 to 25 years of age) from Study P301, there will be at least 90% power to demonstrate noninferiority of the immune response as measured by seroresponse rate in adolescents in Study P203 at a 2-sided α of 0.05, compared with that in young adults of (18 to 25 years of age) from Study P301 receiving mRNA-1273, assuming true seroresponse rate of 85% in young adults (18 to 25 years of age) from Study P301, and a true seroresponse rate of 85% in adolescents in Study P203 (ie, true rate difference is 0 compared to young adults [18 to 25 years of age] from Study P301), and a noninferiority margin of 10%.

Safety Analyses

All safety analyses will be based on the Safety Set, except summaries of solicited ARs, which will be based on the Solicited Safety Set. All safety analyses will be provided by treatment group.

Safety and reactogenicity will be assessed by clinical review of all relevant parameters including solicited ARs (local and systemic events), unsolicited AEs, SAEs, MAAEs, AESI, AEs leading to withdrawal, vital sign measurements, and physical examination findings. The number and percentage of participants with any solicited local AR, with any solicited systemic AR, and with any solicited AR during the 7-day follow-up period after each injection (except Part 1B) will be summarized.

The number and percentage of participants with any solicited local AR, with any solicited systemic AR, and with any solicited AR during the 7-day follow-up period after each dose (except Part 1B) will be provided. A 2-sided 95% exact CI using the Clopper-Pearson method will also be provided for the percentage of participants with any solicited AR.

The number and percentage of participants with unsolicited AEs, SAEs, MAAEs, Grade 3 or higher ARs and AEs, and AEs leading to discontinuation from IP or withdrawal from the study will be summarized. Unsolicited AEs will be presented by the Medical Dictionary for Regulatory Activities (MedDRA) preferred term and system organ class.

The number of events of solicited ARs, unsolicited AEs/SAEs, and MAAEs will be reported in summary tables accordingly.

Immunogenicity Analyses

The SAP will describe the complete set of immunogenicity analyses, including the approach to sample participants into an Immunogenicity Subset for analysis of immunogenicity. The PP Immunogenicity Subset is the primary analysis set for immunogenicity unless otherwise specified. The primary immunogenicity objective of this study is to use the immunogenicity response to infer efficacy in adolescents (12 to < 18 years in this study). The primary immunogenicity analysis is performed in Part 1A.

If an accepted serum Ab threshold of protection against COVID-19 is available based on data from other mRNA-1273 studies or external data, the number and percentage of participants with Ab greater than or equal to the threshold at Day 57 will be provided with a 2-sided 95% CI using the Clopper-Pearson method. If the lower bound of the 95% CI on the mRNA-1273 group is >70%, the primary immunogenicity objective of this study will be considered to be met.

The percentage of participants with serum Ab greater than or equal to the threshold with 95% CI will be provided at each postbaseline timepoint. The CI will be calculated using the Clopper-Pearson method.

If an accepted serum Ab threshold of protection against COVID-19 is not established, the noninferiority of primary vaccine response as measured by Ab GM value and seroresponse rate in adolescents compared with those in young adults (18 to 25 years of age) receiving mRNA-1273 will be assessed. The study is considered as meeting the primary immunogenicity objective if the noninferiority of the immune response to mRNA-1273 as measured by both GM value and seroresponse rate at Day 57 is demonstrated in adolescents in this study at a 2-sided α of 0.05, compared with that in young adults (18 to 25 years of age) in Study P301 receiving mRNA-1273.

An analysis of covariance model will be carried out with Ab value at Day 57 as a dependent variable and a group variable (adolescents in Study P203 and young adults [18 to 25 years of age] in Study P301) as the fixed variable. The GM values of the adolescents at Day 57 will be estimated by the geometric least squares mean (GLSM) from the model. The GMR will be estimated by the ratio of GLSM from the model. A corresponding 2-sided 95% CI will be provided to assess the difference in immune response for the adolescents in Study P203 compared to the young adults (18 to 25 years of age) in Study P301 at Day 57. The noninferiority of immune response to mRNA-1273 as measured by GM value will be considered demonstrated if the lower bound of the 95% CI of the GMR is >0.667 based on the noninferiority margin of 1.5.

The number and percentage (rate) of participants achieving Ab seroresponse at Day 57 will be summarized. The difference of seroresponse rates between adolescents receiving mRNA-1273 in Study P203 and young adults (18 to 25 years of age) receiving mRNA-1273 in Study P301 will be calculated with 95% CI. The noninferiority in seroresponse rate of adolescents in Study P203 compared to young adults (18 to 25 years of age) in Study P301 will be considered demonstrated if the lower bound of the

95% CI of the seroresponse rate (SRR) difference is >-10%, based on the noninferiority margin of 10%.

In addition, the GM level of specific nAb and bAb with corresponding 95% CI will be provided at each timepoint. The 95% CIs will be calculated based on the t-distribution of the log transformed values then back transformed to the original scale. The GM fold-rise of nAb and bAb with corresponding 95% CI will be provided at each timepoint with Day 57 as the primary timepoint of interest. Descriptive summary statistics including median, minimum, and maximum will also be provided.

Efficacy Analyses

To evaluate the incidence of COVID-19 after vaccination with mRNA-1273 or placebo, the incidence rate will be provided by vaccination group, calculated as the number of cases divided by the total person-time. The incidence rate ratio of mRNA-1273 versus placebo will be provided with 95% CI computed using the exact method conditional upon the total number of cases adjusted by the total person-time.

For SARS-CoV-2 infection (serologically confirmed SARS-CoV-2 infection or COVID-19), regardless of symptomatology or severity, infection rate will be provided by vaccination group. The infection rate ratio of mRNA-1273 versus placebo may be provided with its 95% CI using the exact method conditional upon the total number of cases adjusted by the total person-time. The incidence rate of asymptomatic SARS-CoV-2 infection will also be provided.

The secondary efficacy analyses will be performed in the PP set, with sensitivity analyses in the full analysis set (FAS), modified intent-to-treat (mITT) Set, and mITT1 Set.

Long-Term Analysis (including Part 1B)

Long-term analysis will be performed including data collected in the Open-label Observational Phase (Part 1B), and prior to BD if a BD is received. The analysis will include participants who are randomized to mRNA-1273 in Part 1A and remained in the study with or without unblinding, and participants who are originally randomized to placebo and later crossed over to receive mRNA-1273 in Part 1B after unblinding. Long-term analysis of applicable safety, efficacy, and immunogenicity endpoints will be summarized descriptively by treatment cohort without treatment group comparison.

In the long-term safety analysis, unsolicited AEs will be summarized.

In the long-term immunogenicity analysis, nAb and bAb values will be summarized at specified timepoints.

In the long-term efficacy analysis, the incidence rates of COVID-19 and of SARS-CoV-2 infection cases will be counted starting 14 days after the second dose of IP for participants in treatment cohorts of mRNA-1273 and Placebo or starting 14 days after the second dose of mRNA-1273 for participants in the Placebo-mRNA-1273 cohort. Incidence rate with 95% CI adjusting for person-time will be provided. The incidence rate of asymptomatic SARS-CoV-2 infection will also be provided.

Hypothesis Testing (Part 1C-1 – Homologous Booster Phase)

The immunogenicity analysis of BD vaccine response against the ancestral strain will be performed using the noninferiority tests of the 2 null hypotheses based on the 2 coprimary endpoints, respectively.

Coprimary Endpoint 1: Ab GM value at BD-Day 29

Null hypothesis

H^1_0: immunogenicity response to mRNA-1273 BD as measured by Ab GM value at BD-Day 29 in adolescents (12-<18 years of age) in Study P203 Part 1C-1 is inferior compared with Ab GM value at Day 57 (28 days after Dose 2) in the primary series of mRNA-1273 in young adults (18 to 25 years of age) in Study P301.

The noninferiority in Ab GM at BD-Day 29 in Study P203 Part 1C-1 compared with Ab GM at Day 57 in the primary series in young adults (18 to 25 years of age) in Study P301 will be demonstrated by the GMR 95% CI lower bound >0.667 using a noninferiority margin of 1.5. The GMR is defined as the GM value of Ab at BD-D29 in Study P203 Part 1C-1 compared with Ab GM value at Day 57 (28 days after Dose 2) following the primary series of mRNA-1273 in Study P301.

<u>Coprimary Endpoint 2: Ab Seroresponse Rate (SRR) at BD-Day 29</u>

Null hypothesis

H^2_0: immunogenicity response to mRNA-1273 BD as measured by SRR at BD-Day 29 in adolescents (12-<18 years of age) in Study P203 Part 1C-1 is inferior compared with SRR at Day 57 (28 days after Dose 2) following the primary series of mRNA-1273 in young adults (18 to 25 years of age) in Study P301.

The noninferiority in SRR at BD-D29 compared with SRR at Day 57 (28 days after Dose 2) following the primary series of mRNA-1273 will be demonstrated by the SRR 95% CI lower bound >-10% using the noninferiority margin of 10%. The SRR difference is defined as the SRR at BD-Day 29 Study P203 Part 1C-1 minus the rate at Day 57 (28 days after Dose 2) following the primary series of mRNA-1273 in young adults in Study P301. Seroresponse is defined as Ab value change from baseline (pre Dose 1) below the lower limit of quantification (LLOQ) to ≥4 × LLOQ, or at least a 4-fold rise if baseline is ≥ LLOQ.

The primary immunogenicity objective in Part 1C-1 is met if the noninferiority is demonstrated based on both coprimary endpoints.

<u>Key Secondary Endpoint 1: GM value of Ab Against Circulating Strain at BD-Day 29</u>

Null hypothesis

H^1_0: immunogenicity response to mRNA-1273 BD as measured by GM value of Ab against circulating strain at BD-Day 29 in adolescents (12-<18 years of age) in Study P203 Part 1C-1 is inferior compared with GM value of Ab against circulating strain at Day 57 (28 days after Dose 2) in the primary series of mRNA-1273 in young adults (18 to 25 years of age) in Study P301.

The noninferiority in Ab GM value at BD-Day 29 in Study P203 Part 1C-1 compared with Ab GM value at Day 57 in the primary series in young adults (18 to 25 years of age) in Study P301 will be demonstrated by the GMR 95% CI lower bound >0.667 using a noninferiority margin of 1.5. The GMR is defined as the ratio of GM value of Ab at BD-Day 29 in Study P203 Part 1C-1 compared with Ab GM value at Day 57 (28 days after Dose 2) following the primary series of mRNA-1273 in Study P301.

<u>Key Secondary Endpoint 2: Seroresponse Rate (SRR) of Ab Against Circulating Strain at BD-Day 29</u>

Null hypothesis

H^2_0: immunogenicity response to mRNA-1273 BD as measured by SRR of Ab against circulating strain at BD-Day 29 in adolescents (12-<18 years of age) in Study P203 Part 1C-1 is inferior compared with SRR of Ab against circulating strain at Day 57 (28 days after Dose 2) following the primary series of mRNA-1273 in young adults (18 to 25 years of age) in Study P301.

The noninferiority in SRR at BD-D29 compared with SRR at Day 57 (28 days after Dose 2) following the primary series of mRNA-1273 will be demonstrated by the SRR 95% CI lower bound >-10% using the noninferiority margin of 10%. The SRR difference is defined as the SRR at BD-Day 29 Study P203 Part 1C-1 minus the rate at Day 57 (28 days after Dose 2) following the primary series of mRNA-1273 in young adults in Study P301. Seroresponse is defined as Ab value change from baseline (pre Dose 1) below the LLOQ to ≥4 × LLOQ, or at least a 4-fold rise if baseline is ≥ LLOQ.

The key secondary immunogenicity objective in Part 1C-1 is met if the noninferiority is demonstrated based on both key secondary endpoints.

Power and Sample Size (Part 1C-1 – Homologous Booster Phase)

All participants enrolled in Part 1A or Part 1B who meet the eligibility criteria for BD will be offered a BD of mRNA-1273 50 µg. With more than 1,000 participants expected to receive mRNA-1273 BD, the study Part 1C-1 has a 90% probability to observe at least 1 participant with an AE at a true AE rate of 0.25%.

Serum samples from all participants will be collected and banked, a subset of participants will be selected, and their samples will be processed for immunogenicity testing (the Immunogenicity Subset) at specified timepoints.

Approximately 400 participants who receive mRNA-1273 BD will be selected for the Immunogenicity Subset for Part 1C-1, with a target of 289 participants receiving mRNA-1273 BD in the PP Immunogenicity Subset with prebooster negative SARS-CoV-2 for Part 1C-1 (adjusting for approximately 25% of participants who may be excluded as they may have prebooster positive SARS-CoV-2, have no immunogenicity results due to any reason or have protocol deviations impacting critical data).

For the primary immunogenicity objective in Part 1C-1, noninferiority tests of 2 null hypotheses based on 2 coprimary endpoints, respectively, will be performed.

- With approximately 289 participants receiving mRNA-1273 BD in the PP Immunogenicity Subset with prebooster negative SARS-CoV-2 in Study P203 Part 1C-1 and 289 participants in the PP Immunogenicity Subset in young adults (18 to 25 years of age) in Study P301, there will be 90% power to demonstrate noninferiority of the immune response post BD as measured by Ab GM value in adolescents in Study P203 Part 1C-1 compared with Ab GM value in young adults (18 to 25 years of age) following primary series of mRNA-1273 in Study P301, at a 2-sided α of 0.05, assuming an underlying GMR value of 1 and a noninferiority margin of 1.5. The SD of the log-transformed levels is assumed to be 1.5.
- With approximately 289 participants receiving mRNA-1273 BD in the PP Immunogenicity Subset with prebooster negative SARS-CoV-2 in Study P203 Part 1C-1 and 289 participants in the PP Immunogenicity Subset in young adults (18 to 25 years of age) in Study P301, there will be at least 90% power to demonstrate noninferiority of the immune response post BD as measured by SRR in adolescents in Study P203 Part 1C-1 compared with SRR in young adults of (18 to 25 years of age) following primary series of

mRNA-1273 in Study P301, at a 2-sided α of 0.05, assuming true SRR of 90% in young adults (18 to 25 years of age) following primary series of mRNA-1273 in Study P301, and a true SRR of 90% post BD in adolescents in Study P203 Part 1C-1 (ie, true rate difference is 0 compared to young adults [18 to 25 years of age] from Study P301), and a noninferiority margin of 10%.

Homologous Booster Phase Analysis (Part 1C-1)

Booster Phase analysis will be performed in participants who receive a BD in Part 1C-1. Safety, immunogenicity endpoints, and incidence of COVID-19 and SARS-CoV-2 infection will be summarized descriptively, and by prebooster SARS-CoV-2 status if applicable.

In the Booster Phase safety analysis, the number and percentage of participants with unsolicited AEs, severe AEs, treatment-related AEs, SAEs, MAAEs, AESIs, and AEs leading to discontinuation from the study participation in Part 1C-1 will be summarized.

The number and percentage of participants with any solicited local AR, with any solicited systemic AR, and with any solicited AR during the 7-day follow-up period after the BD will be provided.

In the Part 1C-1 Booster Phase, for the coprimary endpoints (Ab GM value and SRR against the ancestral strain) and key secondary endpoints (Ab GM value and SRR against the circulating strain), the following analyses will be performed for the respective hypothesis testing:

- The GM titers with 95% CI will be summarized using t-distribution of the log transferred values and then back transformed to the original scale. The GMR with 95% CI to compare postbooster GM value at BD-Day 29 in adolescents in Study P203 with the primary series GM value at Day 57 (28 days after Dose 2) in young adults (18 to 25 years of age) in Study P301 will be computed based on the t-distribution of mean difference in the log transferred values and then back transformed to the original scale.
- The SRR with 95% CI (using Clopper-Pearson method) will be summarized. The SRR difference with 95% CI (using Miettinen-Nurminen score method) to compare postbooster SRR at BD-Day 29 in adolescents in Study P203 with the primary series SRR at Day 57 (28 days after Dose 2) in young adults in Study P301 will be computed. Seroresponse is defined as Ab value change from baseline (pre Dose 1) below the LLOQ to ≥4 × LLOQ, or at least a 4-fold rise if baseline is ≥ LLOQ.

In the Booster Phase immunogenicity analyses, the GM titers or levels for specific nAb and bAb, with corresponding 95% CI will be provided at BD-Day 1, BD-Day 29, BD-Day 181, and BD-Day 361 (Part 1C-1). Geometric mean fold rise (GMFR) relative to pre Dose 1, BD-Day 1 and prior Day 57 in the phase of primary 2-dose series of mRNA-1273 will be summarized with 95% CIs at BD-Day 29, BD-Day 181, and BD-Day 361 (Part 1C-1). The 95% CIs will be calculated based on the t-distribution of the log transformed values then back transformed to the original scale. Descriptive summary statistics including median, minimum, and maximum will also be provided. The immunogenicity descriptive summaries will be provided in the participants who have available nAb and bAb assay results for the specified timepoints.

In the Booster Phase, the incidence rate of the first occurrence of symptomatic COVID-19 starting 14 days after the BD will be summarized by prebooster SARS-CoV-2 status. The incidence rate of SARS-CoV-2 infection (symptomatic or asymptomatic infection) starting 14 days after the BD, and the incidence rate of asymptomatic SARS-CoV-2 infection starting 14 days after the BD will be

summarized. The incidence rates with 95% CI will be computed adjusting for person-years starting from BD-Day 1 in Part 1C-1.

Part 1C-2 Heterologous Booster Phase

Power and Sample Size

Approximately 362 participants who received non-Moderna COVID-19 vaccine as primary series vaccination at least 3 months prior were planned to be enrolled to receive mRNA-1273 50 µg as heterologous booster in Part 1C-2. However, the study population in Part 1C-2 will have less than the planned number of participants enrolled, as a result of slow enrollment and discontinuation of recruitment in Part 1C-2.

Heterologous Booster Phase Analysis (Part 1C-2)

Heterologous Booster Phase analysis will be performed in participants who receive mRNA-1273 50 µg in Part 1C-2. Safety, immunogenicity endpoints, and incidence of COVID-19 and SARS-CoV-2 infection will be summarized descriptively, and by prebooster SARS-CoV-2 status if applicable.

In the Heterologous Booster Phase safety analysis, the number and percentage of participants with unsolicited AEs, severe AEs, treatment-related AEs, SAEs, MAAEs, AESIs, and AEs leading to discontinuation from the study participation in Part 1-C2 will be summarized.

The number and percentage of participants with any solicited local AR, with any solicited systemic AR, and with any solicited AR during the 7-day follow-up period after the BD will be provided.

In the Heterologous Booster Phase immunogenicity analyses, the GM titers or levels for specific nAb and bAb (against the ancestral strain or against specific circulating variants of interest), with corresponding 95% CI will be provided at BD-Day 1, BD-Day 29, BD-Day 181, and BD-Day 361. GMFR relative to BD-Day 1 will be summarized with 95% CIs at BD-Day 29, and BD-Day 181, and BD-Day 361. The 95% CIs will be calculated based on the t-distribution of the log transformed values then back transformed to the original scale. Descriptive summary statistics including median, minimum, and maximum will also be provided for GM value and GMFR. The SRR and category of at least 2-fold rise relative to BD-Day 1 will be summarized with numbers and percentages of participants. The immunogenicity descriptive summaries will be provided in the PP Immunogenicity Subset who have available nAb and bAb assay results for the specified timepoints.

In the Heterologous Booster Phase, the incidence rate of the first occurrence of symptomatic COVID-19 starting 14 days after the BD will be summarized by prebooster SARS-CoV-2 status. The incidence rate of SARS-CoV-2 infection (symptomatic or asymptomatic infection) starting 14 days after the BD, and the incidence rate of asymptomatic SARS-CoV-2 infection starting 14 days after the BD will be summarized. The incidence rates with 95% CI will be computed adjusting for person-years starting from BD-Day 1 in Part 1C-2.

Part 2

There will be no hypothesis testing in Part 2, given that the enrollment in Part 2 was discontinued. All analyses for Part 2 will be descriptive based on available data.

Part 2 Power and Sample Size

Approximately 362 participants were planned to be enrolled to receive mRNA-1273 50 µg in the open-label Part 2. However, the study population in Part 2 will have less than the planned number of

participants receiving at least one dose of 50 µg mRNA-1273, with a small sample size as a result of slow enrollment and discontinuation of recruitment in Part 2.

Part 2 Analyses

Part 2 safety and immunogenicity endpoints will be summarized descriptively.

In the safety analysis, the number and percentage of participants with unsolicited AEs, severe AEs, treatment-related AEs, SAEs, MAAEs, AESIs, and AEs leading to discontinuation from the study participation in Part 2 will be summarized.

The number and percentage of participants with any solicited local AR, with any solicited systemic AR, and with any solicited AR during the 7-day follow-up period after each injection will be provided.

In the immunogenicity descriptive analyses, the GM value for specific Ab (against the ancestral strain or against specific circulating variants of concern) with corresponding 95% CI, and GMFR from baseline with 95% CI will be provided by timepoint. GM and GMFR 95% CIs will be calculated based on the t-distribution of the log transformed values then back transformed to the original scale. Descriptive summary statistics including median, minimum, and maximum will also be provided for GM and GMFR. The SRR and category of at least 2-fold rise from baseline by timepoint will be summarized with numbers and percentages of participants. The immunogenicity descriptive summaries will be provided in the PP Immunogenicity Subset who have available nAb and bAb assay results for the specified timepoints.

Part 3

Hypothesis testing for the endpoints that infer effectiveness of the 50 µg mRNA-1273.222 in adolescents will be performed on the null hypotheses for the 2 coprimary endpoints as follows:

Coprimary Endpoint 1: Post Dose 1 Ab GM value against Omicron BA.4/BA.5 at Day 29 (superiority testing)

The null hypothesis H_0: GM value of Ab against Omicron BA.4/BA.5 at Day 29 post Dose 1 of 50 µg mRNA-1273.222 in adolescents who are baseline SARS-CoV-2 positive is not superior to GM value at Day 57 post Dose 2 of 100 µg mRNA-1273 in young adults (18 to 25 years of age baseline SARS-CoV-2 negative) in Study P301.

The superiority in Ab GM value against Omicron BA.4/BA.5 in adolescents who are baseline SARS-CoV-2 positive compared with that in young adults (18 to 25 years of age baseline SARS-CoV-2 negative) is demonstrated by meeting the following success criterion:

- The lower bound of the 95% CI of the GMR >1.

The GMR is the ratio of the GM value against Omicron BA.4/BA.5 at Day 29 post Dose 1 of 50 µg mRNA-1273.222 in adolescents who are baseline SARS-CoV-2 positive in Study P203 Part 3 over the GM value against Omicron BA.4/BA.5 at Day 57 in young adults (18 to 25 years of age baseline SARS-CoV-2 negative) post Dose 2 of 100 µg mRNA-1273 in Study P301.

Coprimary Endpoint 2: Post Dose 1 Ab GM value against the ancestral strain at Day 29 (noninferiority testing)

The null hypothesis H_0: GM value of Ab against the ancestral strain at Day 29 post Dose 1 of 50 µg mRNA-1273.222 in adolescents who are baseline SARS-CoV-2 positive is inferior to GM value at

Day 57 post Dose 2 of 100 µg mRNA-1273 in young adults (18 to 25 years of age baseline SARS-CoV-2 negative) in Study P301.

The noninferiority in Ab GM value against the ancestral strain in adolescents who are baseline SARS-CoV-2 positive compared with that in baseline SARS-CoV-2 negative young adults (18 to 25 years of age) is demonstrated by meeting the following success criterion:

- The lower bound of the 95% CI of the GMR >0.667.

The GMR is the ratio of the GM value against the ancestral strain at Day 29 post Dose 1 of 50 µg mRNA-1273.222 in adolescents who are baseline SARS-CoV-2 positive in Study P203 Part 3 over the GM value against ancestral strain at Day 57 in baseline SARS-CoV-2 negative young adults (18 to 25 years of age) post Dose 2 of 100 µg mRNA-1273 in Study P301.

Part 3 Power and Sample Size

With at least 300 adolescents enrolled in Part 3 to receive 50 µg mRNA-1273.222 primary series, the study will have at least a 95% probability to observe at least 1 participant with an AE at a true AE rate of 1%.

Assuming a true serum Ab GMR of 1.6 for GM value against Omicron BA.4/BA.5 at Day 29 after Dose 1 of mRNA-1273.222 in baseline SARS-CoV-2 positive adolescents compared with GM value against Omicron BA.4/BA.5 at Day 57 after Dose 2 of mRNA-1273 primary series in young adults who are baseline SARS-CoV-2 negative in P301, with approximately 168 participants in the PP Immunogenicity Subset who are baseline SARS-CoV-2 positive in P203 Part 3 and 300 young adults in P301 who are baseline SARS-CoV-2 negative, there will be at least >90% power to demonstrate superiority of the serum Ab GM value against Omicron BA.4/BA.5 after Dose 1 of mRNA-1273.222 in adolescents in P203 to that after Dose 2 of mRNA-1273 in young adults in Study P301 at two-sided α of 0.05 using a superiority margin of 1.0. The standard deviation of the natural log transformed levels of Ab is assumed to be 1.5.

Assuming a true serum Ab GMR of 1.1 for GM value against ancestral strain at Day 29 after Dose 1 of mRNA-1273.222 in baseline SARS-CoV-2 positive adolescents compared with GM value against ancestral strain at Day 57 after Dose 2 of mRNA-1273 primary series in young adults who are baseline SARS-CoV-2 negative in P301, this sample size will also provide at least >90% power to demonstrate noninferiority of GM value of Ab against ancestral strain at Day 29 post Dose 1 of mRNA-1273.222 in adolescents in P203 to that after Dose 2 of mRNA-1273 in young adults in Study P301 at two-sided α of 0.05 using a noninferiority margin of 1.5. The standard deviation of the natural log transformed levels of Ab is assumed to be 1.5.

With approximately 30% of participants in Part 3 who may be excluded from the PP Immunogenicity Subset-baseline SARS-CoV-2 positive (PPIS-POS), as they may be baseline SARS-CoV-2 negative or have missing immunogenicity results due to any reason or protocol deviations impacting critical data, approximately 240 participants are required in Part 3 to provide 168 participants in the PP Immunogenicity Subset-baseline SARS-CoV-2 positive.

Part 3 Analyses

In the safety analysis, the number and percentage of participants with unsolicited AEs, severe AEs, treatment-related AEs, SAEs, MAAEs, AESIs, and AEs leading to discontinuation from the study participation in Part 3 will be summarized.

The number and percentage of participants with any solicited local AR, with any solicited systemic AR, and with any solicited AR during the 7-day follow-up period after each injection will be provided.

In the Part 3 immunogenicity analysis, for the coprimary endpoints (Ab GM value against Omicron BA.4/BA.5 and the ancestral strain at Day 29 after Dose 1 of 50 µg mRNA-1273.222 in baseline SARS-CoV-2 positive adolescents in Study P203 compared with those at Day 57 after Dose 2 of 100 µg mRNA-1273 in baseline SARS-CoV-2 negative adults 18 to 25 years of age in Study P301), the following analyses will be performed for the respective hypothesis testing:

An ANCOVA model will be carried out with Ab value at Day 29 in P203 and Ab value at Day 57 in P301 as a dependent variable and a group variable (baseline SARS-CoV-2 positive adolescents in Study P203 and baseline negative SARS-CoV-2 young adults in Study P301) as the fixed variable, where a covariate may be included in the model (to be specified in the SAP). The GM value at Day 29 in P203 and the GM value at Day 57 in P301, and respective GMR for P203 compared to P301 will be estimated from the model. A corresponding 2-sided 95% CI of GMR estimated from the ANCOVA model will be provided to assess the difference in immune response after Dose 1 (Day 29) for the adolescents in Study P203 compared to that after Dose 2 (Day 57) in the young adults (18 to 25 years of age) in Study P301.

The first coprimary endpoint, superiority of immune response to mRNA-1273.222 against Omicron BA.4/BA.5 after Dose 1 compared to mRNA-1273 against Omicron BA.4/BA.5 after Dose 2 as measured by GM value will be considered demonstrated if the respective GMR 95% CI lower bound is >1 based on a superiority margin of 1.

The second coprimary endpoint, noninferiority of immune response to mRNA-1273.222 against ancestral strain after Dose 1 compared to mRNA-1273 against the ancestral strain after Dose 2 as measured by GM value will be considered demonstrated if the respective GMR 95% CI lower bound is >0.667 based on the noninferiority margin of 1.5. The GM value and GMR with specified CIs will also be summarized using t-distribution of the log transferred values and then back transformed to the original scale, and by baseline SARS-CoV-2 status.

For secondary immunogenicity endpoints (Ab GM values after Dose 1 or Dose 2 in adolescents compared with those after Dose 2 in adults in P301), descriptive summaries will be provided in the participants who have available nAb and bAb assay results for the specified timepoints. Secondary endpoints evaluating SRR with 95% CIs (using Clopper-Pearson method) will be summarized by baseline SARS-CoV-2 status at Day 29 and Day 209 in adolescents in Study P203, and at Day 57 in young adults in Study P301. Seroresponse at subject level is defined as Ab value change from baseline (preDose 1) below the LLOQ to ≥4 × LLOQ, or at least a 4-fold rise if baseline is ≥LLOQ. The SRR and category of at least 2-fold rise from baseline by timepoint will be summarized with numbers and percentages of participants.

The GM values for specific nAb and bAb, with corresponding 95% CIs, and GMFR from baseline with 95% CIs will be summarized by timepoint. The 95% CIs will be calculated based on the t-distribution of the log transformed values then back transformed to the original scale. Descriptive summary statistics including median, minimum, and maximum will also be provided.

The incidence rate of the first occurrence of symptomatic COVID-19, 14 days after Dose 1, and 14 days after Dose 2, will be summarized. The incidence rates with 95% CIs will be computed adjusting for person-years.

Analysis Sets

The analysis sets are defined in the following table:

Analysis Set	Description
Randomization Set	All participants who are randomized, regardless of the participants' treatment status in the study.
FAS	All randomized participants who received at least 1 injection of IP.
Immunogenicity Subset	A subset of participants in the FAS will be selected for immunogenicity testing.
PP Immunogenicity Subset	A subset of participants in the FAS will be selected for immunogenicity testing. The PP Immunogenicity Subset includes participants selected for the Immunogenicity Subset who received planned doses of study vaccination per schedule, complied with immunogenicity testing schedule, and have no major protocol deviations that impact key or critical data. Participants who are seropositive at baseline will be excluded from the PP Immunogenicity Subset. The PP Immunogenicity Subset will be used for analyses of immunogenicity unless specified otherwise.
PP Set for Efficacy	All participants in the FAS who received planned doses of study vaccination, had no immunologic or virologic evidence of prior COVID-19, and have no major protocol deviations that impact key or critical efficacy data.
Safety Set	All randomized participants who receive at least 1 dose of IP. The Safety Set will be used for all analyses of safety except for the solicited ARs.
mITT1 Set	All participants in the mITT Set excluding those who received the wrong treatment (ie, at least 1 dose received is not as randomized or planned).
PP Immunogenicity Subset for BD phase	The PP Immunogenicity Subset for BD phase includes participants who received planned BD per schedule, complied with immunogenicity testing schedule, and have no major protocol deviations that impact key or critical data, with prebooster negative or positive SARS-CoV-2. The PP Immunogenicity Subset with prebooster negative SARS-CoV-2 will be used for the primary immunogenicity analysis.
PP Immunogenicity Subset for Part 3	The PP Immunogenicity Subset for Part 3 includes participants who received planned dose per schedule, complied with immunogenicity testing schedule, and have no major protocol deviations that impact key or critical data, with baseline negative or positive SARS-CoV-2. The PP Immunogenicity Subset for

	Part 3 with baseline-positive SARS-CoV-2 status will serve as the population for the primary and secondary analysis of immunogenicity data at Day 29 in Part 3.<br>The PP Immunogenicity Subset (Part 3, at Day 209) will serve as the population for the primary and secondary analysis of immunogenicity data at Day 209 in Part 3.

Abbreviations: AR = adverse reaction; bAb = binding antibody; COVID-19 = coronavirus disease 2019; FAS = full analysis set; IP = investigational product; mITT = modified intent-to-treat; PP = per-protocol; RT-PCR = reverse transcription polymerase chain reaction; SARS-CoV-2 = Severe Acute Respiratory Syndrome coronavirus 2.

Note: Positive SARS-CoV-2 status at baseline is defined as a positive RT-PCR test for SARS-CoV-2, and/or a positive serology test based on bAb specific to SARS-CoV-2 nucleocapsid (as measured by Roche Elecsys Anti-SARS-CoV-2 assay) on or before Day 1. Negative status is defined as a negative RT-PCR test for SARS-CoV-2, and a negative serology test based on bAb specific to SARS-CoV-2 nucleocapsid (as measured by Roche Elecsys Anti-SARS-CoV-2 assay) on or before Day 1.

Multiplicity Adjustment

A hierarchical sequential hypothesis testing (fixed-sequence) method will be used to adjust multiplicity to preserve the family-wise type I error rate ($\alpha = 0.05$). The hypothesis testing for the 2 coprimary endpoints (geometric mean titer [GMT] and SRR) for the primary series of mRNA-1273 in Part 1A was completed and statistically significant based on data snapshot dated 08 May 2021, and thus the α level of 0.05 can be passed to Part 1C-1 hypothesis testing. In Part 1C-1 Homologous Booster Phase, the hypothesis testing for the 2 coprimary endpoints (GMT and SRR against the ancestral strain) after BD of mRNA-1273 will be tested at α level of 0.05. The testing in Part 1A and 1C-1 will continue through the sequence only until an endpoint is not statistically significant (did not meet specified noninferiority success criteria), in which case the testing will stop. If the hypothesis testing for the 2 coprimary endpoints in Part 1C-1-Homologous Booster Phase is statistically significant (meeting the noninferiority success criteria of the coprimary endpoints), the α level of 0.05 will be passed to the hypothesis testing in Part 3.

For the key secondary objective in Part 1C-1 Homologous Booster Phase to evaluate immune response elicited by the 50 µg prototype booster of mRNA-1273 against variant(s) of interest, the key secondary endpoints will be analyzed independently, given that prototype vaccine mRNA-1273 does not contain variant specific sequences.

Part 3

Since the hypothesis testing for the coprimary endpoints in Part 1C-1 was statistically significant based on an interim analysis (IA) with a data cutoff dated 16 May 2022, the α level of 0.05 (two-sided) was passed to Part 3 hypothesis testing.

Study Analyses:

Interim Analyses

More than one IA may be performed.

- The IA of immunogenicity, safety, and efficacy will be performed after Day 57 immunogenicity data are available for the Immunogenicity Subset and at least 1,500 participants (1,000 participants receiving mRNA-1273) have completed Day 57

(1 month after Dose 2, Part 1A). This IA will be considered the primary analysis of immunogenicity for Part 1A.

- An IA of immunogenicity and safety may be performed after all or subset of participants who receive BD have completed BD-Day 29 after the BD in Part 1C-1.
- An IA of immunogenicity and safety may be performed after all or subset of participants who receive heterologous BD have completed BD-Day 29 after the BD in Part 1C-2.
- An IA of immunogenicity and safety may be performed after Day 57 immunogenicity data are available after all or a subset of participants in Part 2 have completed Day 57 (1 month after Dose 2, Part 2).
- An IA of immunogenicity and safety may be performed after all or a subset of participants who receive 50 µg mRNA-1273.222 dose have completed Day 29 (1 month after Dose 1) and an additional IA may be performed in all or a subset of participants who completed Day 209 (1 month after Dose 2) in Part 3.
- At the Sponsor’s discretion, a clinical study report (CSR) may be developed for an IA.

Final Analysis

The final analysis of all applicable endpoints will be performed after all participants have completed all planned study procedures. Results of this analysis will be presented in a final CSR, including individual listings.

TABLE OF CONTENTS

CLINICAL STUDY PROTOCOL 1
PROTOCOL APPROVAL – SPONSOR SIGNATORIES 2
DECLARATION OF INVESTIGATOR 3
PROTOCOL AMENDMENT SUMMARY OF CHANGES 4
PROTOCOL SYNOPSIS 8
TABLE OF CONTENTS 44
LIST OF TABLES 49
LIST OF FIGURES 49
LIST OF ABBREVIATIONS AND TERMS 50
1. INTRODUCTION 54
1.1. Study Rationale 54
1.2. Background and Overview 57
1.2.1. Nonclinical Studies 58
1.2.2. Clinical Studies 59
1.3. Benefit/Risk Assessment 67
1.3.1. Potential Benefits from Participation 67
1.3.2. Risks to Study Participants and Risk Mitigation 68
1.3.3. Overall Benefit/Risk Conclusion 69
2. OBJECTIVES AND ENDPOINTS 70
3. STUDY DESIGN 79
3.1. General Design 79
3.1.1. Part 1 Study Design 79
3.1.2. Part 2 Study Design 86
3.1.3. Part 3 Study Design 87
3.2. Scientific Rationale for Study Design 88
3.3. Justification for Dose, Control Product, and Choice of Study Population 90
3.4. End-of-Study Definition 92
4. STUDY POPULATION 93
4.1. Eligibility Criteria (Part 1A, Part 2, and Part 3) 93

4.1.1. Inclusion Criteria 93
4.1.2. Exclusion Criteria 93
4.2. Study Eligibility Criteria (Part 1B) 95
4.3. Study Eligibility Criteria (Part 1C-1 – Homologous Booster Phase) 95
4.4. Study Eligibility Criteria (Part 1C-2 – Heterologous Booster Dose): 96
4.5. Lifestyle Restrictions 97
4.6. Screen Failures (Part 1A: Blinded Phase, Part 2, and Part 3) 98
5. STUDY TREATMENT 99
5.1. Investigational Product Administered 99
5.2. Randomization (Part 1A Only) 99
5.3. Dosing and Management of mRNA-1273 Vaccine 99
5.3.1. Preparation of Study Vaccine for Injection 99
5.3.2. Administration of Study Vaccine 100
5.3.3. Study Vaccine Delivery and Receipt 100
5.3.4. Study Vaccine Packaging and Labeling 101
5.3.5. Study Vaccine Storage 101
5.3.6. Study Vaccine Accountability 101
5.3.7. Study Vaccine Handling and Disposal 102
5.4. Study Treatment Compliance 102
5.5. Prior and Concomitant Medications 102
5.5.1. Prior Medications and Therapies 102
5.5.2. Concomitant Medications and Therapies 102
5.5.3. Recording of Concomitant Medications and Concomitant Vaccinations 103
5.5.4. Concomitant Medications and Vaccines that May Lead to the Elimination of a Participant from Per-Protocol Analyses 103
5.6. Intervention After the End of the Study 104
6. DELAYING OR DISCONTINUING STUDY TREATMENT AND PARTICIPANT WITHDRAWAL FROM THE STUDY 105
6.1. Criteria for Delay of Vaccine Administration 105
6.1.1. Individual Participant Criteria for Delay of Study Vaccination 105
6.2. Discontinuing Study Vaccination 105
6.3. Participant Discontinuation/Withdrawal from the Study 106
6.4. Study Pause Rules 107

6.5. Lost to Follow-up 108
7. STUDY ASSESSMENTS AND PROCEDURES 109
7.1. Safety Assessments and Procedures 110
7.1.1. Use of Electronic Diaries 110
7.1.2. Safety Telephone Calls 112
7.1.3. Safety Laboratory Assessments 113
7.1.4. Vital Sign Measurements 113
7.1.5. Physical Examinations 113
7.1.6. Assessment for SARS-CoV-2 Infection 114
7.2. Immunogenicity Assessments 115
7.3. Exploratory Assessments and Biomarkers 116
7.4. Efficacy Assessments 116
7.4.1. Vaccine Effectiveness Assessments 116
7.4.2. Surveillance for COVID-19 Symptoms 117
7.4.3. Follow-up/Convalescent Period After Diagnosis with COVID-19 119
7.5. Safety Definitions and Related Procedures 120
7.5.1. Adverse Event 120
7.5.2. Serious Adverse Events 120
7.5.3. Solicited Adverse Reactions 121
7.5.4. Medically Attended Adverse Events 123
7.5.5. Adverse Events of Special Interest 124
7.5.6. Recording and Follow-up of Pregnancy 127
7.5.7. Eliciting and Documenting Adverse Events 127
7.5.8. Assessment of Intensity 128
7.5.9. Assessment of Causality 129
7.5.10. Reporting Adverse Events 129
7.5.11. Reporting SAEs 129
7.5.12. Time Period and Frequency for Collecting AE and SAE Information 130
7.5.13. Method of Detecting AEs and SAEs 131
7.5.14. Follow-up of AEs and SAEs 131
7.5.15. Regulatory Reporting Requirements for SAEs 131
7.6. Safety Monitoring 131
7.6.1. Data Safety Monitoring Board 132

7.6.2. Independent Cardiac Event Adjudication Committee 132
7.7. Treatment of Overdose 132
7.8. Pharmacokinetics 132
7.9. Pharmacodynamics 132
7.10. Biomarkers 133
7.11. Health Economics 133
8. STATISTICAL ANALYSIS PLAN 134
8.1. Blinding and Responsibility for Analyses 134
8.1.1. Breaking the Blind 135
8.2. Statistical Hypothesis 135
8.2.1. Part 1 135
8.2.2. Part 2 137
8.2.3. Part 3 138
8.3. Power and Sample Size 138
8.3.1. Part 1 138
8.3.2. Part 2 140
8.3.3. Part 3 141
8.4. Analysis Sets 141
8.5. Statistical Methods 143
8.5.1. Baseline Characteristics and Demographics 143
8.5.2. Safety Analyses 143
8.5.3. Immunogenicity Analyses in Part 1 144
8.5.4. Efficacy Analyses 145
8.5.5. Long-term Analysis (Including Part 1B) 145
8.5.6. Booster Phase Analysis 146
8.5.7. Part 2 Analysis 147
8.5.8. Part 3 Analysis 148
8.5.9. Exploratory Analyses 149
8.5.10. Subgroup Analyses 149
8.6. Multiplicity Adjustment 149
8.7. Study Analyses 150
8.7.1. Interim Analyses 150
8.7.2. Final Analysis 150

9. REFERENCES 151
10. SUPPORTING DOCUMENTATION AND OPERATIONAL CONSIDERATIONS 157
10.1. APPENDIX 1: Schedule of Assessments 158
10.2. APPENDIX 2: Study Governance Considerations 181
10.2.1. Regulatory and Ethical Considerations 181
10.2.2. Study Monitoring 181
10.2.3. Audits and Inspections 182
10.2.4. Financial Disclosure 183
10.2.5. Recruitment Procedures 183
10.2.6. Informed Consent/Assent Process 183
10.2.7. Protocol Amendments 184
10.2.8. Protocol Deviations 184
10.2.9. Data Protection 185
10.2.10. Sample Retention and Future Biomedical Research 185
10.2.11. Dissemination of Clinical Study Data 186
10.2.12. Data Quality Assurance and Quality Control 186
10.2.13. Data Collection and Management 187
10.2.14. Source Documents 187
10.2.15. Retention of Records 188
10.2.16. Study and Site Closure 188
10.2.17. Publication Policy 188
10.2.18. BMI Charts for Boys and Girls 189
10.3. APPENDIX 3: Contraceptive Guidance 191
10.4. APPENDIX 4: Adverse Event of Special Interest Terms 192
10.5. APPENDIX 5: Protocol Amendment History 194

LIST OF TABLES

Table 1: Part 1 Study Objectives and Endpoints 70

Table 2: Part 2 Objectives and Endpoints 75

Table 3: Part 3 Objectives and Endpoints 76

Table 4: Non-Moderna COVID-19 Vaccine Available Under Emergency Use Authorization 85

Table 5: Pause Rule Criteria, Events, and Thresholds 107

Table 6: Solicited Adverse Reactions and Grades 122

Table 7: Analysis Sets 141

Table 8: Analysis Strategy for Safety Parameters 143

Table 9: Treatment Cohorts for the Long-term Analysis 146

Table 10: Schedule of Assessments Part 1A, the Blinded Phase; Part 1B Open-label Observational Phase for Participants who Received mRNA-1273 in Part 1A 159

Table 11: Participant Decision Clinic Visit 163

Table 12: Schedule of Assessments Part 1B, Open-label Observational Phase for Participants Who Previously Received Placebo 164

Table 13: Schedule of Assessments Part 1C-1 – Homologous Booster Dose Phase 166

Table 14: Schedule of Assessments Part 1C-2 – Heterologous Booster Phase 169

Table 15: Schedule of Assessments Part 2 173

Table 16: Schedule of Assessments Part 3 177

Table 17: Medical Concepts that Qualify as Adverse Events of Special Interest 192

LIST OF FIGURES

Figure 1: Overall Design Schema 79

Figure 2: Study Schema (Part 1A, Blinded Phase) 81

Figure 3: Study Schema (Part 1B, Open-label Observational Phase) 83

Figure 4: Study Schema (Part 1C-1, Open-label Homologous Booster Dose Observational Phase) 84

Figure 5: Study Schema (Part 2) 87

Figure 6: Study Schema (Part 3) 88

Figure 7: Surveillance for COVID-19 Symptoms and the Corresponding Clinical Data Pathways 118

Figure 8: Schedule of Part 1A and Part 1B Participant Visits During the Part 1B Open-label Period 163

LIST OF ABBREVIATIONS AND TERMS

The following abbreviations and terms are used in this study protocol.

Abbreviation or Specialist Term	Definition
Ab	antibody
AE	adverse event
AESI	adverse events of special interest
ANCOVA	analysis of covariance
AR	adverse reaction
bAb	binding antibody
BD	booster dose
BLA	biologics license application
BMI	body mass index
CBER	Center for Biologics Evaluation and Research
CD	cluster of differentiation
CDC	Centers for Disease Control and Prevention
CEAC	cardiac event adjudication committee
CFR	Code of Federal Regulations
CI	confidence interval
CoV	coronavirus
CoV S	S protein SARS-CoV-2
COVID-19	coronavirus disease 2019
CRO	contract research organization
CRP	C-reactive protein
CSR	clinical study report
DMID	Division of Microbiology and Infectious Diseases
DSMB	Data Safety Monitoring Board
DSPC	1,2-distearoyl-sn-glycero-3-phosphocholine
ECG (or EKG)	electrocardiogram
ECMO	extracorporeal membrane oxygenation
eCRF	electronic case report form

Abbreviation or Specialist Term	Definition
EDC	electronic data capture
eDiary	electronic diary
EOS	end-of-study
ERD	enhanced respiratory disease
ESR	erythrocyte sedimentation rate
EUA	Emergency Use Authorization
FAS	full analysis set
FDA	Food and Drug Administration
FIO2	fraction of inspired oxygen
GCP	Good Clinical Practice
GLSM	geometric least squares mean
GM	geometric mean
GMFR	geometric mean fold rise
GMR	geometric mean ratio
GMT	geometric mean titer
HCP	healthcare practitioner
HIV	human immunodeficiency virus
IA	interim analysis
IB	investigator's brochure
ICF	informed consent form
ICH	International Council for Harmonisation
ICU	intensive care unit
IL-6	interleukin 6
IM	intramuscular(ly)
IP	investigational product
IRB	institutional review board
LAR	legally acceptable representative(s)
LDH	lactic acid dehydrogenase
LLOQ	lower limit of quantification

Abbreviation or Specialist Term	Definition
LNP	lipid nanoparticle
LTFU	lost to follow-up
MAAE	medically attended adverse event
MedDRA	Medical Dictionary for Regulatory Activities
MERS	Middle East Respiratory Syndrome
MIS-C	multisystem inflammatory syndrome in children
mITT	modified intent-to-treat
mRNA	messenger RNA
nAb	neutralizing antibody
NHP	nonhuman primate
NP	nasopharyngeal
PaO2	partial pressure of oxygen
PEG2000-DMG	1-monomethoxypolyethyleneglycol-2,3-dimyristylglycerol polyethylene glycol 2000
PP	per-protocol
PsVNA	pseudovirus neutralizing antibody
PsVNT	pseudovirus neutralization titer
QA	quality assurance
RT-PCR	reverse transcriptase polymerase chain reaction
S	spike
S2P	S protein
SAE	serious adverse event
SAP	statistical analysis plan
SARS	Severe Acute Respiratory Syndrome
SARS-CoV-2	Severe Acute Respiratory Syndrome coronavirus 2
SD	standard deviation
SM-102	heptadecane-9-yl 8-((2-hydroxyethyl)(6-oxo-6-(undecyloxy)hexyl)amino)octanoate
SMC	Safety Monitoring Committee

Abbreviation or Specialist Term	Definition
SoA	schedule of assessments
SpO2	oxygen saturation
SRR	seroresponse rate
TEAE	treatment-emergent adverse event
Th1	T helper cell 1
Th2	T helper cell 2
VE	vaccine efficacy
VOC	variant of concern
WHO	World Health Organization
WOCBP	woman of childbearing potential

1. INTRODUCTION

1.1. Study Rationale

Coronaviruses are a large family of viruses that cause illness ranging from the common cold to more severe diseases, such as MERS and SARS. Coronaviruses are zoonotic, meaning they are transmitted between animals and people. An outbreak of COVID-19 caused by SARS-CoV-2 began in Wuhan, Hubei Province, China in December 2019 and has spread throughout China and to over 215 other countries, territories, and areas including the USA (WHO 2020a). On 11 Mar 2020, the WHO officially declared COVID-19 a pandemic.

Following the outbreak of COVID-19, ModernaTX, Inc. (hereafter referred to as the Sponsor) applied its mRNA vaccine platform and coronavirus research experience to rapidly develop the mRNA-1273 vaccine against SARS-CoV-2. At the time of study initiation, evidence suggested that the burden of COVID-19 has begun to increase in younger age groups (>408000 COVID-19 cases and 88 deaths in children less than 18 years of age as of 21 Sep 2020), particularly as schools in the USA started to reopen for in person instruction (CDC 2022a). Adolescents also likely represented a segment of the population contributing toward sustained community transmission of SARS-CoV-2 as well as spread of SARS-CoV-2 within households given their mobility and less likelihood of compliance with mask wearing and social distancing (Fontanet et al 2020).

Since then, there have been approximately 6.2 million COVID-19 cases and 772 deaths as of 30 Aug 2022 in the US (CDC 2022b) in adolescents 12-17 years of age. Since Mar 2020, approximately 1 in 4 hospitalized children and adolescents (0 to 17 years old) with COVID-19 have required intensive care (CDC 2022c). While rates of severe disease have not increased since the start of the pandemic, the increase in absolute numbers of cases has added substantial burden, in terms of the increased number of emergency department and urgent care clinic encounters and hospitalizations in the adolescent population (Klein et al 2022; CDC 2022c).

In addition to the increased incidence and hospitalizations observed, COVID-19 disease in children and adolescents can include MIS-C, a unique complication in conjunction with severe disease (Vogel et al 2021). MIS-C shares some common features with Kawasaki disease, and is defined as fever, laboratory evidence of inflammation, and involvement of ≥2 organ systems in an individual <21 years of age with no plausible alternative diagnosis and evidence of current or recent SARS-CoV-2 infection. MIS-C is also associated with cardiac complications. Through 01 Aug 2022, 8798 cases of MIS-C have been reported in the US, with 71 MIS-C related deaths reported. The median age of patients with MIS-C was 9 years (range 5 to 13 years) (CDC 2022d).

Myocarditis has been well described as a complication of pediatric COVID-19. Children under 16 years of age with COVID-19 are at an almost 37-fold higher risk of myocarditis than the uninfected age- and gender-matched control population (Boehmer et al 2020). Of note, the frequency of myocarditis due to COVID-19 is higher than that of vaccine-associated myocarditis. Additionally, the clinical syndrome of COVID-19 related myocarditis is more severe and clinically meaningful than vaccine-associated myocarditis. Patients with vaccine-associated myocarditis generally have a milder clinical course than patients with classic

myocarditis or MIS-C myocarditis, with a lower likelihood of cardiac dysfunction at presentation and more rapid recovery (Patel et al 2022).

Long COVID, a sequela of COVID-19, affecting up to 20% of persons infected with SARS-CoV-2, has also been described in children, including in those with a mild infection. This syndrome is manifested by symptoms that can include fatigue, muscle and joint pain, insomnia, concentration difficulties, respiratory problems, persistence anosmia and ageusia, and cardiac palpitations that may occur within 6 months after infection (Buonsenso et al 2022; Buonsenso et al 2021; Dembinski et al 2021).

Variants of concern such as Delta and Omicron have also emerged contributing to the substantial burden in the adolescent population. After the onset of the Omicron wave, the demographics of hospitalized patients with COVID-19 shifted to younger age groups (Abdullah et al 2022; Goga et al 2021; United Kingdom Health Security Agency 2022). Furthermore, the peak number of hospitalizations during Omicron was nearly 4 times greater than that observed during the Delta variant wave (Delahoy et al 2021; Marks et al 2022a; Marks et al 2022b).

Taken together, a vaccine that prevents COVID-19 and its sequelae as well as SARS-CoV-2 transmission in adolescents would be a crucial public health tool to help curb the pandemic.

At the time the present study was initiated, no vaccine had been authorized or licensed to prevent SARS-CoV-2, and there was an urgent public health need to develop one, there being no proven therapy. The Sponsor initiated an accelerated development program for mRNA-1273 vaccine against SARS-CoV-2 infection (Section 1.2), and started a Phase 3 clinical study in the US involving administration of mRNA-1273 vaccine 100 µg as both an initial dose and a second dose 28 days later (Section 1.2.2). Success criteria for early efficacy were met at first IA based on 95 adjudicated cases with a VE of 94.5% (95% CI: 86.5%, 97.8%; one-sided p-value < 0.0001), and EUA was granted for mRNA-1273 in the adult population.

Data from the ongoing pivotal study mRNA-1273-P301 (Study P301) showed that mRNA-1273 continued to induce durable protection: clinical efficacy against COVID-19 was maintained at 93% through a median of approximately 5 months after the second dose.

The primary objective for the Blinded Phase 2/3 study, mRNA-1273-P203 (Study P203), is to evaluate the safety and reactogenicity of a single dose level (100 µg) of mRNA-1273 vaccine administered in 2 doses 28 days apart (Section 3.1) to an adolescent population. Specifically, the primary immunogenicity objective of the study is to infer effectiveness of mRNA-1273 (100 µg, 2 doses 28 days apart) 28 days after Dose 2 of mRNA-1273 (Day 57) by comparison of immune responses in adolescents (aged ≥ 12 through < 18 years) to the young adult (≥18 to ≤25 year of age) cohort in Study P301, where efficacy was demonstrated.

Interim data from the current Study P203 (based on a data snapshot of 08 May 2021) met the primary immunogenicity objective by demonstrating noninferiority of both the (i) GM value of serum nAb and (ii) the seroconversion rate from adolescent participants compared with those from young adults (aged 18 to 25 years) enrolled in the ongoing Phase 3 efficacy study (Study P301). These interim results supported administration of mRNA-1273 as two 100 µg doses 28 days apart in adolescents between ≥12 and 17 years of age. On 17 Jun 2022, the FDA granted EUA of the mRNA-1273 vaccine for adolescents 12 through 17 years of age. Global filings/authorizations down to 12 years of age have also occurred.

An additional objective of the current Study P203 is to evaluate safety and immunogenicity of a 50 µg of mRNA-1273 given as a 2 dose primary series as well as when given as a homologous and heterologous booster vaccine. The rationale for these objectives is as follows: In Part A of Study mRNA-1273-P201 (Study P201), the time course and magnitude of antibody (both bAb and nAb) responses to mRNA-1273 was similar between 100 µg and 50 µg dose levels (both showing strong immunogenicity) at each postbaseline timepoint (Days 29, 43, 57, and 209), although the 100 µg dose group had numerically greater responses. In addition, recent interim results of the ongoing Study P201 support administration of a BD of vaccine. Adult participants in Study P201 received 2 doses of either 50 µg or 100 µg of mRNA-1273 and were administered a 50 µg booster of mRNA-1273 6 to 8 months after the second dose. Participants in Study P201 who received the BD demonstrated enhanced immune responses to SARS-CoV-2 compared to preboost levels and met the noninferiority criteria stipulated in the FDA Guidance on EUA for Vaccines to Prevent COVID-19. Interim results from Study mRNA-1273-P205 (Study P205) also show that a 50 µg booster of mRNA-1273 increased nAb levels against Omicron approximately 37-fold compared to preboost levels. Available data also show that heterologous or mixed series of COVID-19 vaccine-induced high immune response in the adult population (Atmar et al 2021). Additionally, no new safety signals emerged upon administration of the BD in Study P201. Based on cumulative evidence, the benefit-risk profile of a BD of mRNA-1273 is favorable, particularly in light of increasing breakthrough disease with the emergence of the Delta and Omicron variants.

The Sponsor also aims to evaluate the safety and immunogenicity of a 50 µg of mRNA-1273.222 given as a 1 dose and 2 dose primary series in COVID-19 vaccine-naïve participants. Recent disease trends support the need for pediatric vaccination strategies that induce broader protection against VOCs (eg, Omicron variant) in addition to the ancestral SARS-CoV-2 strain. In addition, increasing prevalence of SARS-CoV-2 infection in the community (Clarke et al 2022), together with Study P205 data showing that Omicron containing vaccine (ie, mRNA-1273.214) elicits strong immunogenicity in SARS-CoV-2 positive individuals (Chalkias et al 2022b), as well as durability of immune response post BD of a bivalent Beta containing vaccine (mRNA-1273.211) up to 6 months with higher nAb responses in adults compared with mRNA-1273 against D614G, Beta, Omicron BA.1, and Delta variants (Chalkias et al 2022a), prompted the evaluation of the immunogenicity of 2 doses of the bivalent mRNA-1273.222 vaccine given 6 months apart which will also allow evaluation of immunogenicity post Dose 1. The Sponsor has developed mRNA-1273.222, a bivalent vaccine consisting of the mRNA that encodes for the prefusion stabilized spike glycoprotein of both the Wuhan-Hu-1 isolate of SARS-CoV-2 and the SARS-CoV-2 B.1.1.529, Omicron, variant. In November 2021, the SARS-CoV-2 Omicron variant was detected in South Africa and was found to contain potential antibody escape site mutations such as K417N, T478K, E484A, and N501Y. The ability of the Omicron variant to escape prior immunity led to an unprecedented rise in COVID-19 infections and related hospitalizations, in adults as well as children, between December 2021 and February 2022. Real world effectiveness studies in adults conducted during the Omicron wave found a lower efficacy of the prototype vaccine against this new variant (Ferdinands et al 2022; Tseng et al 2022). Moderna COVID-19 investigational variant-containing vaccine candidates are being evaluated as booster vaccines in Study P205 including the BA.1 Omicron containing vaccine, mRNA-1273.214 and the Omicron BA.4/BA.5 containing vaccine mRNA-1273.222. Results for the vaccine against Omicron BA.4/BA.5 are pending. These studies included adult participants,

who previously received a primary series of mRNA-1273 (100 µg) and some participants in Part G and Part H of Study P205 who also previously received a 3rd dose (50 µg) of mRNA-1273, received a 4th dose (second booster given at a 50 µg) of bivalent Omicron containing mRNA-1273.214 and mRNA-1273.222. For the mRNA-1273.214 booster recipients, local reactogenicity of the 4th dose was similar to 2nd dose of primary series and 3rd dose of original mRNA-1273. Systemic reactogenicity of the 4th dose was generally lower compared with 2nd dose of primary series and third dose of mRNA-1273. Overall safety profile of mRNA-1273.214 was similar with original mRNA-1273 as 4th dose (second booster). nAb titers for Omicron BA.1 were significantly higher following 4th dose (second booster) of bivalent mRNA-1273.214 vaccine than with mRNA-1273 among participants who had no prior SARS-CoV-2 infection. For these participants, success criteria were met for superiority of GMTs. In addition, a 4th dose of mRNA-1273.214 elicited significantly higher neutralizing titers than mRNA-1273 in adult participants who had prior SARS-CoV-2 infection. A 4th dose of mRNA-1273.214 also resulted in higher nAb titers against Omicron BA.4/BA.5 than mRNA-1273 in adults. It is anticipated that the immunogenicity and safety profile observed in the bivalent containing Omicron BA.1 mRN-1273.214 given as a 4th dose (second booster) will be comparable to the bivalent containing Omicron BA.4/BA.5 mRNA-1273.222 given as a 50 µg 1- or 2-dose primary series.

1.2. Background and Overview

The Sponsor's scalable mRNA/LNP technology platform allowed for a rapid response to the COVID-19 pandemic and was used to develop mRNA-1273, a novel LNP-encapsulated mRNA-based vaccine against SARS-CoV-2. The Sponsor's mRNA/LNP platform is based on the principle and observations that cells in vivo can take up mRNA, translate it, and then express protein viral antigen(s) on the cell surface. The delivered mRNA does not enter the cellular nucleus or interact with the genome, is nonreplicating, and is expressed transiently. Vaccine mRNA-1273 encodes for the full-length spike (S) protein SARS-CoV-2(CoV S). The CoV S protein mediates attachment and entry of the virus into host cells, by binding to the angiotensin converting enzyme 2 receptor followed by membrane fusion, making it a primary target for nAb that prevents infection (Johnson et al 2016; Wang et al 2015; Wang et al 2018; Chen et al 2017; Corti et al 2015; Yu et al 2015; Kim et al 2019; Widjaja et al 2019; Corbett et al 2020a; Ju et al 2020; Robbiani et al 2020).

The monovalent mRNA-1273 has been licensed as a 2-dose 100 µg primary series in adults and has been authorized under emergency use in adolescents 12 to 17 years of age. With the recent surge of a more divergent Omicron subvariant, the development of a bivalent mRNA-1273.222 vaccine is being accelerated to address the current SARS-CoV-2 outbreak (Hastie et al 2021).

In November 2021, the Omicron variant (B.1.1.529; BA.1) emerged as the most antigenically divergent variant to date that currently has more than 30 mutations in the S2P protein (Hastie et al 2021). In early 2022, new Omicron subvariants BA.4/BA.5 emerged which have their own unique and shared mutations with the BA.1. Leveraging the mRNA/LNP technology platform, the Sponsor initiated the development of modified COVID-19 vaccines against SARS-CoV-2 variants including the mRNA-1273.222 which contains 2 mRNAs: CX-024414 encoding for the S-2P of Wuhan-Hu-1 and CX-034476 encoding for the S-2P of Omicron subvariants BA.4/BA.5. (See IB V8 Addendum 2)

1.2.1. Nonclinical Studies

Nonclinical studies have demonstrated that CoV S proteins are immunogenic and S protein-based vaccines, including those based on mRNA delivery platforms, are protective in animals. Prior clinical studies of vaccines targeting related CoVs and other viruses have demonstrated that mRNA-based vaccines are safe and immunogenic. mRNA-1273 has shown preliminary evidence of protection against SARS-CoV-2 in studies in young mice (Corbett et al 2020a) and NHPs (Corbett et al 2020b).

Nonclinical studies in mice, hamsters, and NHPs assessing the monovalent mRNA-1273.529, bivalent mRNA-1273.214 (contains the ancestral SARS-CoV-2 and the Omicron variant BA.1 spike sequences), and bivalent mRNA-1273.222 (contains the ancestral SARS-CoV-2 and the Omicron variant BA.4/BA.5 spike sequences) vaccines are ongoing. These studies are designed to assess these vaccines as a primary series (2-dose) or as a booster (3rd or 4th dose) in animals previously vaccinated with mRNA-1273. Overall, interim data suggest that a primary series with a monovalent vaccine induces robust neutralization against the matched and closely related variants but does not induce significant neutralization against highly divergent SARS-CoV-2 lineages. A bivalent vaccine, however, induces broader neutralization across divergent lineages. In these studies, no significant differences were measured in CD4 or CD8 cytokine production in mice that received mRNA-1273, mRNA-1273.529, or mRNA-1273.214.

Mice were vaccinated with 2 doses of monovalent mRNA-1273, BA.1-matched monovalent mRNA-1273.529, or bivalent mRNA-1273.214, with 2 doses administered 3 weeks apart. At Day 36 (2 weeks after the second dose), robust serum nAb responses, determined via pseudovirus neutralization assay, were observed against Wuhan-1 D614G after a 2-dose primary series of 1 μg mRNA-1273, with slightly lower nAb titers observed against B.1.351 or B.1.617.2. Serum neutralizing activity, however, was significantly reduced against the BA.1 and BA.2 compared with Wuhan-1 D614G. Mice immunized with mRNA-1273.529 had high neutralization antibody titers against BA.1 and BA.2, but low levels of neutralization against Wuhan-1 D614G, B.1.351, and B.1.617.2. In mice vaccinated with the bivalent mRNA-1273.214, significant neutralization against both the prototype strain (Wuhan-1 D614G) and against BA.1 and BA.2 was measured, indicating that the bivalent vaccine affords broader neutralization across variants.

In addition, mRNA-1273.214 and mRNA-1273.222 were studied in mice previously vaccinated with primary series of mRNA-1273. Mice were boosted with original (mRNA-1273), BA.1 Omicron bivalent Omicron mRNA-1273.214, or Omicron BA.4/BA.5 bivalent mRNA-1273.222 approximately 31 weeks between primary series and booster. Data shows increased immunogenicity of bivalent mRNA-1273.214 and mRNA-1273.222 vaccines (7- and 3-fold increase, respectively in BA.1 neutralization titers) & (4.2- and 4.5-fold increase, respectively in Omicron BA.4/BA.5 neutralization titers) after BD in mice whereas limited immunogenicity was observed from the original mRNA-1273 vaccine. Bivalent vaccines also provide stronger protection from BA.5 infection in the lungs (Scheaffer et al 2022).

In support of the development of mRNA-1273 for prophylaxis against SARS-CoV-2 infection, nonclinical immunogenicity, biodistribution, and safety studies have been completed with similar mRNA-based vaccines formulated in LNPs containing SM-102 (heptadecane-9-yl 8-((2-hydroxyethyl)(6-oxo-6-(undecyloxy)hexyl)amino)octanoate), the novel proprietary lipid used in the mRNA-1273 LNP formulation.

A detailed review of nonclinical experience with mRNA-1273 vaccine is provided in the IB.

1.2.2. Clinical Studies

The clinical development of mRNA-1273 vaccines to support its use in the adult population consists of 5 ongoing clinical trials being conducted in the US:

- Two Phase 1, open-label, dose ranging studies (Study 20-0003; NCT04283461; Study 21-0002, NCT04785144) sponsored by the NIAID.
- Phase 2a, randomized, observer-blind, placebo-controlled, dose-confirmation study (Study mRNA-1273-P201; NCT04405076).
- Phase 3 randomized, stratified, observer-blind, placebo-controlled study (Study mRNA-1273-P301; NCT04470427) to evaluate the efficacy, safety, and immunogenicity of the vaccine.
- Phase 3b, open-label study (Study mRNA-1273-P304, NCT04860297) to evaluate the safety and immunogenicity of the vaccine in adult solid organ transplant recipients and healthy controls.

In addition, 2 ongoing trials are being conducted in the pediatric population:

- Phase 2/3 randomized, placebo-controlled safety and immunogenicity study of mRNA-1273 in children 12 to <18 years of age (Study mRNA-1273-P203 [current study]; NCT04649151).
- Phase 2/3, study to evaluate the safety and immunogenicity of mRNA-1273 in children 6 months to <12 years of age (mRNA-1273-P204; NCT04796896).

In addition, 4 ongoing trials are being conducted to support SARS-CoV-2 variants:

- Study mRNA-1273-P205 (NCT04927065) is an open-label, Phase 2/3 study with multiple parts to evaluate the safety and immunogenicity of boosters for SARS-CoV-2 variants.
- Study mRNA-1273-P206 (NCT05584202) is a Phase 2, Part 1 open-label, Part 2 randomized placebo-controlled study to evaluate the safety, reactogenicity and immunogenicity of the bivalent mRNA1273.214 vaccine for SARS-CoV-2 variants of concern in infants aged 12 weeks to <6 months.
- Study mRNA-1273-P305 (NCT05249829) is a Phase 2/3, randomized, observer-blind, active-controlled, multicenter study to evaluate the immunogenicity and safety of Omicron variant vaccines in comparison with mRNA-1273 (prototype) booster vaccine.
- Study mRNA-1273-P306 is a Phase 3 open-label study to evaluate the safety and immunogenicity of the mRNA1273.214 vaccine for SARS-CoV-2 variants of concern in participants aged 6 months to <6 years of age (Study mRNA-1273-P306; NCT05436834).

1.2.2.1. Phase 1 Study DMID20-0003

The safety and immunogenicity of mRNA-1273 were evaluated in Phase 1 (DMID 20-0003) and Phase 2 (P201) studies that were important to select and to confirm the dose of the vaccine used

in the pivotal Phase 3 study. In Study 20-0003 (Phase 1), 2 doses of 100 µg or higher generated the highest titers of nAb or bAb with an acceptable safety profile, and this observation was the basis for selecting the 100 µg dose for use in the pivotal Phase 3 study. Importantly, the Ab levels after 2 doses of mRNA-1273 exceeded those from a pool of convalescent sera. Neutralizing activity was observed for the 100 µg mRNA-1273 dose as of Day 36 and was higher than that of the convalescent sera control group, and the median titers remained in the same range as the median titer in the convalescent sera control group at Day 119 across the age strata. In this study, the majority of the solicited ARs were mild or moderate. A higher incidence of severe solicited ARs was observed with the 250 µg dose (in the 18–55-year age cohort) compared with the lower doses (25 µg, 50 µg, and 100 µg); thus, the mRNA-1273 250 µg dose was not evaluated in participants ≥56 years of age. One severe unsolicited AE related to mRNA-1273 and 1 severe clinically meaningful elevation in serum lipase related to mRNA-1273 were also observed with the 250 µg dose (in the 18–55-year age cohort). Additionally, in Study 20-0003, Th1-directed CD4+ T-cells were observed to be induced across age groups, with limited indication of a Th2-directed response, and similar responses were observed among all age groups for the 100 µg dose. The predominance of a Th1-directed T-cell profile helps mitigate concern of the risk of enhanced disease associated with Th2-driven pathophysiology.

1.2.2.2. Phase 2 Study mRNA-1273-P201

Study P201 is an ongoing Phase 2a, safety, reactogenicity, and immunogenicity study in healthy adults that provided confirmation of the immunogenicity of both the 100 µg and 50 µg doses. The study was designed as a randomized, observer-blind, placebo-controlled dose-confirmation study (Part A). Two mRNA-1273 dose levels, 50 µg and 100 µg, and placebo were evaluated in 2 age cohorts: Cohort 1 enrolled participants ≥18 to < 55 years old (300 participants), and Cohort 2 enrolled participants ≥ 55 years old (300 participants). A total of 600 participants received either mRNA-1273 or placebo according to a 1:1:1 randomization ratio, ie, within each age cohort, 100 participants each received mRNA-1273 50 µg, mRNA-1273 100 µg, or placebo.

An amendment to the Study 201 protocol adapted the study design to include open-label interventional phases (Part B and Part C). Part B allowed unblinding of participants and offered 2 injections of mRNA-1273 in an open-label manner, 28 days apart, to all participants who received placebo in Part A. Part B also offered a single BD of mRNA-1273 (50 µg) to participants who received 1 or 2 doses of mRNA-1273 (50 µg or 100 µg) in Part A. Part C was prompted by the need to proactively prepare for vaccination strategies that might induce broader protection, including against emerging variants of SARS-CoV-2 such as B.1.351. Part C enrolled participants from Study 301 who received 2 doses of mRNA-1273 100 µg at least 6 months prior. Part C participants received a single injection of mRNA-1273.351 (20 µg or 50 µg) or mRNA-1273.351 mixture (50 µg total – 25 µg of mRNA-1273 and 25 µg of mRNA-1273.351). mRNA-1273 demonstrated an acceptable safety profile in the participant population enrolled in this study at both dose levels and both age cohorts, as observed through 6 months after the second injection. Vaccination with mRNA-1273 in Study 201 resulted in robust immune responses to SARS-CoV-2 in participants 18 years and older at both dose levels, and persistence of immune response was observed up to 6 months after the second injection in Part A of the study. In Part A of Study 201, the time course and magnitude of antibody (both bAb and nAb) responses to mRNA-1273 was similar between 100 µg and 50 µg dose levels at each postbaseline timepoint (Days 29, 43, 57, and 209), although the 100 µg dose group had numerically greater

responses (and was the dose selected for the pivotal Phase 3 efficacy study). In Part B of Study 201, administration of a 50 µg BD of mRNA-1273 6 months or more after the primary series improved the immune responses to 1.7-fold the peak achieved after the primary vaccination series in the current mRNA-1273-P301 study, where efficacy of mRNA-1273 against COVID-19 was demonstrated. Safety data based on preliminary results for 50 µg BDs of mRNA-1273 (selected participants in Part B) or mRNA-1273.351 (Part C Cohort 1) are available. Percentages of participants with solicited local and systemic AEs were similar in the group who received mRNA-1273.351 as a BD compared to those who received mRNA-1273 vaccine as a BD. The majority of solicited local and systemic AEs were mild (Grade 1) or moderate (Grade 2). The frequency of any Grade 3 solicited local or systemic AE was 15% (3 of 20 participants) after the BD of mRNA-1273 and 10.5% (2 of 19 participants) after the BD of mRNA-1273.351. There were no Grade 4 solicited local or systemic AEs. The most common solicited local AE was injection site pain after injection in both groups (68.4% for the mRNA-1273.351 vaccine and 90.0% for the mRNA-1273 vaccine). The most common solicited systemic AEs after the BD of the mRNA-1273.351 vaccine were fatigue (36.8%), headache (36.8%), myalgia (31.6%), and arthralgia (21.1%). The most common solicited systemic AEs after the BD of the mRNA-1273 vaccine were fatigue (70.0%), headache (55.0%), arthralgia (50.0%), and myalgia (45.0%). Fever was reported after the BD of mRNA-1273 in 3 of 20 participants (15%) but not after the BD of mRNA-1273.351 (0 of 19 participants). There were no SAEs reported in this study.

1.2.2.3. Phase 2/3 Study mRNA-1273-P205

Part G of Study P205 will evaluate the immunogenicity, safety, and reactogenicity of the mRNA-1273.214 vaccine (contains the ancestral SARS-CoV-2 and the Omicron variant spike sequences) when administered as a single BD to adult participants who have previously received the mRNA-1273 primary series as well as a BD. The results of the mRNA-1273.214 vaccine will be compared to the immunogenicity induced after an mRNA-1273 BD, in individuals who also previously received the 2-dose primary series of mRNA-1273. Interim results are available. Part H of the study will evaluate the immunogenicity, safety, and reactogenicity of the mRNA-1273.222 vaccine (contains the ancestral SARS-CoV-2 and the Omicron variant BA.4/BA.5 spike sequences) when administered as a single BD to adult participants who have previously received the mRNA-1273 primary series as well as a BD. The results of the mRNA-1273.222 vaccine will be compared with the immunogenicity induced after an mRNA-1273 BD in participants who also previously received the 2-dose primary series of mRNA-1273. Results from this study are pending.

Immunogenicity Study (mRNA-1273-P205) – Day 29 Interim Results of Study mRNA-1273.214:

The bivalent booster vaccine, 50 µg mRNA-1273.214 contains equal amounts of the mRNA sequence for the S protein, 25 µg of the ancestral SARS-CoV-2 and 25 µg of the Omicron spike mRNA sequence. The immunogenicity of 50 µg mRNA-1273.214 was evaluated in Part G of the P205 Study when administered as a second BD in adults who previously received 2 doses of 100 µg mRNA-1273 as a primary series and a single BD of 50 µg mRNA-1273. The IA of Study P205 showed that all primary and key secondary immunogenicity objectives were met based on the prespecified testing sequence. The mRNA-1273.214 dose of 50 µg elicited a

superior nAb response against Omicron and a noninferior neutralizing response against the ancestral SARS-CoV-2 strain compared with the 50 µg mRNA-1273 28 days after the BD.

Specifically, in the primary analysis set, defined as participants without evidence of prior SARS-CoV-2 infection, the estimated Day 29 nAb GMTs (95% CI) against the ancestral SARS-CoV-2 (D614G) were 6422.3 (5990.1, 6885.7) and 5286.6 (4887.1, 5718.9) 28 days after the mRNA-1273.214 and mRNA-1273 BDs, respectively. The GMR (97.5% CI) was 1.22 (1.08, 1.37), meeting the prespecified criterion for noninferiority (lower bound of 97.5% CI >0.667). The Day 29 SRR (95% CI) against ancestral SARS-CoV-2 was 100% (98.9, 100 and 98.6, 100) 28 days after in the mRNA-1273.214 and mRNA-1273 groups, respectively, with an SRR difference of 0, meeting the noninferiority criterion (lower bound of CI >-10%). In participants who received the mRNA-1273.214, who were SARS-CoV-2 positive, baseline nAb GMT against the ancestral strain as well as 28 days post BD were higher than those who were SARS-CoV-2 negative (Chalkias et al 2022b).

The estimated Day 29 nAb GMTs against Omicron were 2479.9 (2264.5, 2715.8) and 1421.2 (1283.0, 1574.4) in the mRNA-1273.214 and mRNA-1273 groups, respectively. The GMR (97.5% CI) was 1.75 (1.49, 2.04), which met the prespecified superiority criterion (lower bound of CI >1). The Day 29 Omicron SRRs (95% CI) were 100% (98.9, 100) and 99.2% (97.2, 99.9) in the mRNA-1273.214 and mRNA-1273 groups, respectively. The SRR difference (97.5% CI) was 1.5% (-1.1, 4.0), meeting noninferiority criteria (lower bound of CI >-10%).

All immunogenicity endpoints were also met based on an analysis that included participants with and without evidence of prebooster SARS-CoV-2 infection.

Additionally, in the primary analysis set (PPSI – Neg, reflecting participants without prior SARS-CoV-2 infection), the observed GMTs (95% CI) against Omicron BA.4/BA5 GMTs (95% CI) prebooster were 115.6 (98.5, 135.6) and increased to 727.4 (632.9, 836.1) at 28 days after the BD for mRNA-1273.214 with a GMFR (95% CI) of 6.3 (5.7, 6.9). The GMTs (95% CIs) in the mRNA-1273.214 group were 139.7 (119.5, 163.3) prebooster and 492.1 (431.1, 561.9) 28 days after the BD, and the GMFR (95% CI) was 3.5 (3.2, 3.9). The GMR (95% CI) for the comparison of mRNA-1273.214 50 µg BD with the mRNA-1273 50 µg BD was 1.69 (1.51, 1.90) with the lower bound of the CI >1 (See IB V8 Addendum 2).

On the basis of these results, the mRNA-1273.214 (50 µg) elicited superior nAb responses against the Omicron subvariants BA.4, BA.5 compared with mRNA-1273 (50 µg) (nominal α of 0.05) and the BA.4, BA.5 nAb response was consistently higher in the mRNA-1273.214 group compared to the mRNA-1273 group in participants with and without prior SARS-CoV-2 infection.

Immunogenicity Study (mRNA-1273-P205) – Interim Results (mRNA-1273.222)

Participants received 50-µg of mRNA-1273 (n=376) or mRNA-1273.222 (n=511) as second booster doses. Omicron BA.4/BA.5 and ancestral SARS-CoV-2 D614G neutralizing antibody geometric mean titers (GMTs [95% confidence interval]) after mRNA-1273.222 (2324.6 [1921.2-2812.7] and 7322.4 [6386.2-8395.7]) were significantly higher than mRNA-1273 (488.5 [427.4-558.4] and 5651.4 (5055.7-6317.3) respectively, at day 29 post-boost in participants with no prior SARS-CoV-2-infection. A randomly selected subgroup (N=60) of participants in the mRNA-1273.222 group also exhibited cross-neutralization against the emerging omicron variants BQ.1.1 and XBB.1. No new safety concerns were identified with mRNA-1273.222.

Vaccine effectiveness was not assessed in this study; in an exploratory analysis 1.6% (8/511) of mRNA-1273.222 recipients had Covid-19 post-boost (Chalkias et al 2022c).

Safety:

The safety and reactogenicity of 50 µg mRNA-1273.214 was evaluated in the Part G of Study P205 when administered as a second BD in adults who previously received 2 doses of 100 µg mRNA-1273 as a primary series and a single BD of 50 µg mRNA-1273. The 50 µg mRNA-1273.214 second BD was administered a median of 136 days after a first BD of the 50 µg mRNA-1273 and had a median follow-up duration of 43 days (range 22 to 51 days). The 50 µg mRNA-1273.214 was well-tolerated and had a reactogenicity profile similar to that of the 50 µg mRNA-1273 when given as a second BD. The incidence of ARs after immunization with 50 µg mRNA-1273.214 was also similar or lower relative to that of a first BD of 50 µg mRNA-1273 and relative to the second dose of the 100 µg mRNA-1273 primary series. No new safety signals were identified.

The overall occurrence of solicited local ARs within 7 days following second BDs was 79.4% and 79.5% in the mRNA-1273.214 and mRNA-1273 groups, respectively. The most common solicited local AR was injection site pain for any Grade event in both groups (77.3% and 76.6% in the mRNA-1273.214 and mRNA-1273 group, respectively). The incidences of systemic ARs were 70.3% in the mRNA-1273.214 and 66.1% in the mRNA- 1273 groups, and the most frequent ARs for any Grade event were fatigue (54.9% and 51.4%), headache (43.9% and 41.1%), and myalgia (39.6% and 38.6%) in the mRNA-1273.214 and mRNA-1273 groups, respectively. The majority of solicited ARs were mild to moderate (Grades 1-2) for both groups. The incidence of Grade 3 events was 8.0% in both groups, with the most common being fatigue (3.4% and 3.1%) and myalgia (2.3% and 3.7%), for the mRNA-1273.214 and mRNA-1273 groups, respectively. No Grade 4 events occurred in either group (See IB V8 Addendum 2).

The unsolicited AEs were reported in 18.5% of participants in the mRNA-1273.214 group and 20.7% in the mRNA-1273 group regardless of the relationship to vaccination up to 28 days after the second BD. In the mRNA-1273.214 group, 2 (0.5%) participants each experienced 2 SAEs (prostate cancer and traumatic fracture) and 1 (0.3%) participant in the mRNA-1273 group reported an SAE of spinal osteoarthritis within 28 days of the BD; none were assessed by the investigator to be related to study vaccination. MAAEs were 9.8% in the mRNA-1273.214 and 13.8% in the mRNA-1273 groups up to 28 days after the second BD vaccination. There were no fatal events or AEs leading to study discontinuation that were related to vaccination as assessed by investigators. As of the data cutoff date, no deaths or AEs of special interest, including cases of myocarditis or pericarditis, occurred, and 1 additional SAE (Grade 3, nephrolithiasis) considered unrelated to study vaccination was reported in the mRNA-1273.214 group. Additional details of results of clinical studies are provided in the IB (IB V8 Addendum 2).

1.2.2.4. Phase 3 Study mRNA-1273-P301

Currently, a Phase 3 pivotal, randomized, placebo-controlled, observer-blind clinical Study P301 is being conducted in participants 18 years of age and older who are at an increased risk of COVID-19 disease. In addition, prespecified cohorts of participants who were either ≥65 years of age or 18 to <65 years of age with comorbid medical conditions were included. A total of 30351 participants were followed for a median of 92 days (range: 1-122) for the development of COVID-19 disease. Success criteria for early efficacy were met at first IA based on 95

adjudicated cases with a VE of 94.5% (95% CI: 86.5%, 97.8%; one-sided p-value <0.0001). Study P301 is expected to provide immunogenicity data where an Ab threshold of protection against COVID-19 will be estimated.

In Study P301, solicited ARs were reported more frequently among vaccine participants than placebo participants. The most frequently reported ARs after any dose in the vaccine group were pain at the injection site (92.0% any Grade; 6.1% Grade ≥3), fatigue (70% any Grade; 10.1% Grade ≥3), headache (64.7% any Grade; 5.7% Grade ≥3), myalgia (61.5% any Grade; 9.1% Grade ≥3) and chills (45.4% any Grade; 1.4% Grade ≥3). The majority of local and systemic ARs had a median duration of 1 to 3 days. Overall, there was a higher reported rate of some ARs in younger age groups: the incidence of axillary swelling/tenderness, fatigue, headache, myalgia, arthralgia, chills, nausea/vomiting, and fever was higher in adults aged 18 to <65 years than in those aged 65 years and above. Grade 3 solicited local ARs were more frequently reported after Dose 2 than after Dose 1. In the participants who received the vaccine, solicited systemic ARs were reported numerically more frequently by vaccine participants after Dose 2 than after Dose 1. Grade 3 systemic ARs (fatigue, myalgia, arthralgia, and headache) were reported more frequently after Dose 2 than after Dose 1. Unsolicited AEs and SAEs were reported at generally similar rates in participants who received mRNA-1273 and placebo from the first dose until the last observation. Unsolicited AEs that occurred in ≥1% of study participants who received mRNA-1273 and at a rate at least 1.5-fold higher rate than placebo, were lymphadenopathy-related events (1.1% of versus 0.6%). All of the lymphadenopathy events are similar to the axillary swelling/tenderness in the injected arm reported as solicited ARs. Hypersensitivity AEs were reported in 1.5% of vaccine recipients and 1.1% of placebo recipients. Hypersensitivity events in the vaccine group included injection site rash and injection site urticaria, which are likely related to vaccination. There have been no cases of severe hypersensitivity or anaphylactic reactions reported immediately after vaccination in the trial to date. There were 3 reports of Bell's palsy in the mRNA-1273 vaccine group (one of which was an SAE), which occurred 22, 28, and 32 days after vaccination, and one in the placebo group, which occurred 17 days after vaccination. The currently available information on Bell's palsy is insufficient to determine a causal relationship with the vaccine. There were 2 SAEs of facial swelling in vaccine recipients with a history of injection of dermatological fillers. The onset of swelling was reported 1 and 2 days, respectively, after vaccination and was likely related to vaccination. There was 1 SAE of intractable nausea and vomiting in a participant with prior history of severe headache and nausea requiring hospitalization. This event occurred 1 day after vaccination and was likely related to vaccination.

There were no other notable patterns or numerical imbalances between treatment groups for specific categories of AEs (including other neurologic, neuro-inflammatory, and thrombotic events) that would suggest a causal relationship to mRNA-1273.

The Sponsor submitted a data package to FDA on 30 November 2020 for consideration of EUA. FDA granted an EUA for mRNA-1273 for use in adults 18 years of age and older on 18 December 2020.

Efficacy and safety results based on the final analysis of Part A with a database lock of 04 May 2021 are provided in the IB. The results are generally comparable to the IA. On 25 Aug 2021, Moderna announced that it had completed the rolling submission process for its BLA to the FDA for the full licensure of the Moderna COVID-19 vaccine (mRNA-1273) for

active immunization to prevent COVID-19 in individuals 18 years of age and older. On 31 Jan 2022, mRNA-1273 was granted full licensure by the US FDA. mRNA-1273 has been showing durable efficacy of 93% through 6 months after the second dose.

Study mRNA-1273-P203 - Interim Results:

Interim data from the current Part 1A of Study P203 (based on a data snapshot of 08 May 2021) show that the primary immunogenicity objective was met by demonstrating noninferiority of both the (i) GM value of serum nAb and (ii) the seroconversion rate from adolescent participants compared with those from young adults (aged 18 to 25 years) enrolled in the ongoing Phase 3 efficacy study (Study P301). The GMR of adolescent (Study P203) to young adult (Study P301) nAb titers at Day 57 was 1.077 (95% CI: 0.939, 1.236), meeting the 1.5-fold noninferiority criterion (ie, lower bound of the 95% CI for GMR is >0.667). The difference in adolescent to young adult nAb SRRs at Day 57 was 0.2 (95% CI: -1.8, 2.4), meeting the 10% noninferiority criterion (lower bound of the 95% of the SRR difference is ≥-10%). In addition, results of VE against COVID-19, defined using either the "Study P301 case definition" or the "CDC case definition", and conducted starting 14 days after Dose 1 or Dose 2 were all similar to the 94.1% VE observed 14 days post Dose 2 in the pivotal Study P301. Overall, local reactogenicity was higher and systemic reactogenicity was lower to mRNA-1273 in adolescents compared with that observed in the adult mRNA-1273-P301 study. Both solicited local and systemic ARs were more commonly reported by participants in the mRNA-1273 group and systemic ARs generally increased after Dose 2. While the majority of these solicited ARs were Grade 1 or Grade 2, there was a higher frequency of Grade 3 or higher solicited ARs in the mRNA-1273 group than in the placebo group after Dose 1 and Dose 2. The majority of the solicited ARs in participants who received mRNA-1273 occurred within the first 1 to 2 days after any dose and generally persisted for a median of 1 to 3 days. The frequency of reported solicited ARs were generally similar and there were no notable differences in the reported rates of unsolicited AEs observed between participants aged ≥ 12 to < 16 years and participants aged ≥16 to <18 years.

Unsolicited treatment-emergent AEs (TEAEs) up to 28 days after any dose were more common in the mRNA-1273 group than in the placebo group. Imbalances in unsolicited TEAEs up to 28 days after any dose was primarily attributable to events related to reactogenicity. The incidence of MAAEs within 28 days of injection was generally similar between the mRNA-1273 and placebo groups. The incidence of unsolicited severe TEAEs and serious TEAEs in the 28 days after any dose was low overall and generally similar in participants who received mRNA-1273 and those who received placebo. Unsolicited TEAEs, including MAAEs, up to 28 days after any dose assessed by the investigator as related to study treatment were more frequently reported in the mRNA-1273 group than in the placebo group.

Data from interim data cut of 31 Jan 2022 showed consistent immunogenicity and safety results as the first interim results (08 May 2021). The evidence from the 08 May 2021 data cutoff infers effectiveness, suggesting that mRNA-1273 is effective in preventing COVID-19, and the known and potential benefits of mRNA-1273 when used to prevent COVID-19 outweigh the known and potential risks.

The updated data collected as of 31 Jan 2022 included: (i) VE of the blinded phase updated as of 31 May 2021; (ii) COVID-19 cases as of 31 Jan 2022; and (iii) safety data from all mRNA-1273 recipients for the blinded and open-label phases of the study.

Vaccine efficacy through 31 May 2021 was 100% (95% CI: 61.2%, not estimated [NE]) using the P301 case definition and 89.9% (95% CI: 51.0%, 98.9%) using the CDC case definition. The updated analysis confirms results provided in the EUA Amendment submitted on 09 Jun 2021 based on the 08 May 2021 data cutoff). Beyond 31 May 2021, the loss of placebo participants precluded meaningful analysis of VE. Long-term assessment of monthly incidence rates among all participants who received mRNA-1273 as randomized and remained on study up to 31 Jan 2022 (combined blinded and unblinded) shows low monthly incidence rates of COVID-19 until November 2021. Not unexpectedly, an increase in COVID-19 incidence rates was observed in December 2021 and January 2022, when the Omicron variant prevailed.

Neutralizing activity against the Omicron variant was evaluated in a small subset (N=20 each study) of serum samples randomly selected from adults (≥18 years) in the pivotal P301 study, and from adolescents (≥12 to <18 years) in Study P203 following a 2-dose regimen of 100 µg mRNA-1273 and from children (6 to <12 years) in Study mRNA-1273-P204 after 2 doses of 50 µg mRNA-1273. In adolescents, nAb measured against Omicron was present in all participants (100%) 4 weeks after Dose 2. The GMT against Omicron was 11.8-fold lower than that measured against D614G among adolescents. Compared with adults, adolescent GMTs were 1.5- and 3.8-fold higher for D614G and the Omicron variant, respectively (Girard et al 2022).

mRNA-1273 was found to be generally safe in clinical Study P203 with no new safety signals in either the 08 May 2021 initial EUA Amendment data cutoff or the longer-term safety cut of 31 Jan 2022. The overall safety profile observed in the adolescent study (Study P203) was generally consistent with the findings to date in the Phase 3 Study P301 in adults aged ≥18 years. In the long-term safety follow-up, SAEs were reported for 22/2577 participants (0.9%) overall. No SAEs were assessed by investigators as related to study vaccine and there were no fatal events. MAAEs were reported for 1002/2577 participants (38.9%) overall. MAAEs considered by the investigator to be related to mRNA-1273 were reported for 26 participants (1.0%). The incidence of MAAEs reflects the Omicron surge. Three participants in the mRNA-1273 group discontinued study vaccine because of a TEAE. AESIs were reported for 13 participants. In the open-label, crossover phase, there was 1 probable case of myocarditis (adjudicated by cardiac endpoint adjudication committee) observed in the Study P203 clinical program which occurred 1 day following second dose but did not require hospitalization and completely resolved by 8 days postvaccination with no sequelae at 5 months of follow-up (Study mRNA-1273-P203 data on file).

Interim results from Part 1C-1 as of data cutoff date of 15 Aug 2022 (DBL of 27 Oct 2022) demonstrate effectiveness of a mRNA-1273 BD (50 µg) in adolescents 12 to <18 years inferred based on comparison of adolescent nAb responses to those post-mRNA-1273 primary series in young adults in Study P301. Results echo the successful immunobridging of the primary series for this same age group. Prespecified NI immunogenicity criteria were met for adolescents (GMR point estimate 5.071 and lower bound of the 95% CI of 4.477; SRR difference point estimate of 0.7% and lower bound of the 95% CI of -0.8%) allowing effectiveness of a 50 µg BD of mRNA-1273 in adolescents 12 to <18 years to be inferred. There were no new safety findings observed in the mRNA-1273 Booster Phase of Study P203 and in general, the BD showed less reactogenicity than the primary series. Most solicited ARs were reported within 2 days, were Grade 1 or 2 and lasted a median of 2 to 3 days. Pain was the most frequently reported solicited local AR and headache and fatigue were the most frequently reported solicited systemic AR. There were no SAEs or AESIs reported in the 28 days after BD. There were no reported deaths

or cases of MIS-C, and even with the enhanced surveillance implemented in this study, no TEAE indicative of myocarditis or pericarditis was identified. Up to the time of data cutoff (15 Aug 2022), the unsolicited AE profile, including SAEs and AESIs, demonstrated events typically observed in an adolescent population during the COVID-19 pandemic (CSR Addendum 1).

Results from Part 2 data snapshot as of 01 May 2023, show robust nAb responses 28 days after 1 dose of 50 µg mRNA-1273 in baseline SARS-CoV-2 positive participants. After 28 days post dose 1 of 50 µg mRNA-1273, the GMC levels in the adolescent baseline-positive participants increased to 18-fold higher than the baseline GMC levels (GMC=6053.2 [95%CI: 4495.8, 8150.1]). Despite the higher baseline nAb responses observed in adolescent baseline-positive participants, the GMC post dose 1 of 50-µg mRNA-1273 was observed to be higher than those observed post primary series in baseline SARS-CoV-2 negative young adults in Study P301 (GMC=1400.4 [95% CI: 1272.7, 1541.0) with a GMR of 4.322 (95% CI: 3.274, 5.707). The seroresponse rates were comparable between the Study P203 adolescents (97.5%, n=39/40) and Study P301 young adults (99.3%, n=292/294), with SRR difference of -1.8% (95% CI: -12.2%, 0.8%). Administration of the second dose did not increase the neutralizing antibody response to the degree which was observed in baseline SARS-CoV2 negative participants (mRNA-1273 Part 2 Data Memo).

Global filings/approvals down to 12 years of age have occurred. On 17 Jun 2022, the US FDA granted EUA of the mRNA-1273 vaccine for adolescents 12 through 17 years of age. Worldwide approvals or authorizations have been issued for mRNA-1273 as 2-dose 100 µg primary series or as a 50 µg BD in this age group. Moderna variant-containing bivalent booster vaccines (mRNA-1273.214 and mRNA-1273.222) have also received approvals or authorizations globally in this age group. On 18 Apr 2023, the FDA amended the EUAs for the Moderna COVID-19 Vaccine (mRNA-1273) and Moderna COVID-19 Vaccine, Bivalent (mRNA-1273.222) to simplify the vaccination schedule for most individuals. In this recent authorization, individuals 12 years of age and older who have not yet received a bivalent COVID-19 vaccine can receive a single dose of Moderna COVID-19, Bivalent (50 µg total mRNA), including individuals who have never received any prior doses of COVID-19 vaccines. In the post-EUA period, anaphylaxis has been reported following mRNA-1273 administration. There have been very rare reports of myocarditis and pericarditis occurring after vaccination with COVID-19 mRNA vaccines. A detailed review of the clinical experience with LNPs containing SM-102 (mRNA vaccines and placebo) is provided in the IB.

1.3. Benefit/Risk Assessment

1.3.1. Potential Benefits from Participation

The following benefits may accrue to participants:

- The mRNA-1273 and mRNA-1273.222 vaccines may be effective against COVID-19 including disease caused by variants of concern.
- Participants will have a baseline (Day 1) evaluation for SARS-CoV-2 infection and ongoing surveillance for COVID-19 throughout the study.

- The study will contribute to the development of a vaccine against COVID-19 for adolescents.

1.3.2. Risks to Study Participants and Risk Mitigation

Immediate systemic allergic reactions (eg, anaphylaxis) can occur following any vaccination. These reactions are very rare and are estimated to occur once per 450,000 vaccinations for vaccines that do not contain allergens such as gelatin or egg protein (Zent et al 2002). As a precaution, all participants will remain under observation at the study site for at least 30 minutes after injection.

Vasovagal syncope (fainting) can occur before or after any vaccination, is usually triggered by the pain or anxiety caused by the injection and is not related to the substance injected. Therefore, it is important that standard precautions and procedures are followed to avoid injury from fainting.

IM injection with other mRNA vaccines manufactured by the Sponsor containing the SM-102 lipid formulation commonly results in a transient and self-limiting local inflammatory reaction. This typically includes pain, erythema (redness), or swelling (hardness) at the injection site, which are mostly mild to moderate in severity and usually occur within 24 hours of injection.

The majority of local and systemic solicited ARs observed after injection with mRNA-1273 at the 100 µg dose level have been mild to moderate in severity (Section 1.2.2). The most commonly reported systemic ARs were headache, myalgia, fatigue, chills, and fever. In the majority of cases, the reactions resolved spontaneously within several days.

There is a theoretical risk that active vaccination to prevent SARS-CoV-2 infection may cause a paradoxical increase in the risk of COVID-19. This possibility is based on the rare phenomenon of vaccine-associated disease enhancement, which was first seen in the 1960s with 2 vaccines made in the same way (formalin-inactivated whole virus) and designed to protect children against infection with respiratory syncytial virus (Chin et al 1969) or measles virus (Fulginiti et al 1967). Disease enhancement has also been proposed as a possible explanation for cases of more serious disease associated with dengue vaccination (Thomas and Yoon 2019; WHO 2019).

In order to address this theoretical risk, animal studies were performed in young and aged wild-type mice and rhesus macaques (NHPs). These studies were designed to capture immunogenicity endpoints that would be predictive of ERD and also to evaluate if, at protective or subprotective dose levels of mRNA-1273, evidence of disease enhancement would be observed after challenge of the animals with SARS-CoV-2. These nonclinical studies demonstrated that mRNA-1273 is safe and well-tolerated in different animal species, is immunogenic; drives a robust SARS-CoV-2-specific Ab, neutralization, and Th1-directed CD4 T-cell response; fully protects animals from challenge at dose levels as low as 1 µg/dose in mice and 30 µg/dose in NHPs; and does not lead to ERD at protective or subprotective dose levels (Corbett et al 2020a; Corbett et al 2020b). Clinical immunogenicity data from the DMID Phase 1 study of mRNA-1273 demonstrated high levels of nAbs and Th1-polarized CD4+ T-cell responses (Jackson et al 2020), consistent with the immunogenicity observed in these nonclinical studies. These data suggest that a paradoxical increase in the risk of disease, while not eliminated, is likely to be low.

In individuals 18 years of age and older, there have been very rare reports of myocarditis and pericarditis occurring after vaccination with Moderna COVID-19 vaccine. The majority of the cases have been reported in young males shortly after the second dose of the vaccine. These are typically mild cases and individuals tend to recover within a short time following standard treatment and rest (Gargano et al 2021). One probable case of myocarditis was reported in this study with features consistent with this typical mild, short-lived pattern (Study mRNA-1273-P203 data on file). Recent data from an observational study (Wong 2022) conducted in 4 large US administrative health plan claims databases covering more than 100 million persons, (OptumServe, HealthCore, Blue Health Intelligence, and CVS Health) observed no statistical difference in risk rates of myocarditis/pericarditis following any dose of 2 mRNA COVID-19 vaccines. Moreover, recently published data from a health care organization in Israel estimated the incidence of myocarditis due to COVID-19 infection as 11.0 cases per 100,000 persons (95% CI: 5.6–15.8), compared to the nearly five-fold lower incidence of myocarditis observed following the 2nd dose of mRNA-1273 (highest incidence rate) at 2.17 per 100,000 person-days (95% CI: 1.55-3.04) (Wong 2022). Other studies also show that myocarditis cases related to COVID-19 infection revealed a higher mortality rate and rate of severe complications compared to myocarditis cases following vaccination with a COVID-19 mRNA vaccine (Patel et al 2022). Moreover, infection with SARS-CoV-2 has more adverse events beyond myocarditis, including many carrying significant risk for death.

In adults, the AEs after receiving a third dose of mRNA-1273 are described in Section 1.2.2. In general, solicited local and systemic ARs after the BD (Study P201) were comparable to what had been observed with the primary series (Study P301). Preliminary report from a heterologous booster study in adults show reactogenicity and adverse events were similar across booster groups (Atmar et al 2021).

1.3.3. Overall Benefit/Risk Conclusion

All participants in Part 1 will receive a single dosage of 100 µg mRNA-1273 vaccine or placebo administered in 2 doses 28 days apart (Section 3.1). Participants in Part 1A who received mRNA-1273 will be offered an option to receive a third BD of 50 µg of mRNA-1273 at least 5 months from second dose in Part 1C-1. Participants in Part 1A who received placebo will be offered to receive a single dose of 100 µg mRNA-1273 vaccine administered in 2 doses 28 days apart in Part 1B and a third BD of 50 µg of mRNA-1273 at least 5 months from second dose in Part 1C-1. All participants in Part 1C-2 will receive a single BD of 50 µg of mRNA-1273. All participants in Part 2 will receive a single dosage of 50 µg mRNA-1273 vaccine administered in 2 doses 28 days apart. All participants in Part 3 will receive single dosage of 50 µg bivalent mRNA-1273.222 vaccine administered in 2 doses 6 months apart.

Safety will be monitored throughout the study (Section 7.6).

Considering the limited number of approved vaccines for COVID-19, the participants' risk of COVID-19 outside the study during a pandemic, and the nonclinical and clinical data to date, the Sponsor considers the potential benefits of participation to exceed the risks.

2. OBJECTIVES AND ENDPOINTS

The objectives which will be evaluated in this study and endpoints associated with each objective are provided in Table 1, Table 2, and Table 3.

Table 1: Part 1 Study Objectives and Endpoints

Objectives	Endpoints
**Primary Objectives**	**Primary Endpoints**
• To evaluate the safety and reactogenicity of 100 µg mRNA-1273 vaccine administered in 2 doses 28 days apart	• Solicited local and systemic ARs through 7 days after each injection<br>• Unsolicited AEs through 28 days after each injection<br>• MAAEs through the entire study period<br>• SAEs through the entire study period<br>• AESIs through the entire study period<br>• Vital sign measurements<br>• Physical examination findings
• To infer efficacy of mRNA-1273 (100 µg, 2 doses 28 days apart), serum Ab responses obtained 28 days after the second injection of mRNA-1273 (Day 57) will be either:<br>– Evaluated against an accepted Ab threshold of protection against COVID-19 (if established in Study P301)<br>– Compared in primary vaccine response as measured by GM values of serum Ab and SRR in Study P203 with those obtained from young adult recipients (18 to 25 years of age) of mRNA-1273 in the clinical endpoint efficacy trial (Study P301)	• The proportion of participants with a serum Ab level at Day 57 ≥ an Ab threshold of protection1<br>• The primary vaccine response as measured by GM value of serum Ab level and SRR from Study P203 vaccine recipients at Day 57 compared with those obtained from young adult recipients (18 to 25 years of age) at Day 57 in the clinical endpoint efficacy trial (Study P301)[2]<br>1. If an accepted serum Ab threshold of vaccine protection against COVID-19 is available, this analysis will form the basis to infer efficacy<br>2. If a threshold is not available, efficacy will be inferred based on establishing noninferiority of adolescent (12 to < 18 years; this clinical study) to adult GM values of serum Ab and SRR obtained in Study P301 (GM value 12 to < 18 years / GM value 18 to 25 years)
**Secondary Objectives**	**Secondary Endpoints**
• To evaluate the persistence of the immune response of mRNA-1273 vaccine (100 µg) administered in 2 doses 28 days apart, as assessed by the level of SARS-CoV-2 S2P-specific bAb through 1 year after Dose 2	• The GM value of SARS-CoV-2 S2P-specific bAb on Day 1, Day 57 (1 month after Dose 2), Day 209 (6 months after Dose 2), and Day 394 (1 year after Dose 2)
• To evaluate the persistence of the immune response of mRNA-1273 vaccine (100 µg) administered in 2 doses	• The GM values of SARS-CoV-2-specific nAb on Day 1, Day 57 (1 month after Dose 2), Day 209

Objectives	Endpoints
28 days apart, as assessed by the level of nAb through 1 year after Dose 2	(6 months after Dose 2), and Day 394 (1 year after Dose 2)
• To evaluate the effect of mRNA-1273 on the incidence of SARS-CoV-2 infection compared with the incidence among placebo recipients	• The incidence of SARS-CoV-2 infection (symptomatic or asymptomatic infection) counted starting 14 days after the second dose of IP<br>• SARS-CoV-2 infection will be defined in participants with negative SARS-CoV-2 at baseline:<br>– bAb level against SARS-CoV-2 nucleocapsid protein negative at Day 1 that becomes positive (as measured by Roche Elecsys) at Day 57 or later, OR<br>– Positive RT-PCR counted starting 14 days after the second dose of IP
• To evaluate the incidence of asymptomatic SARS-CoV-2 infection after vaccination with mRNA-1273 or placebo	• The incidence of asymptomatic SARS-CoV-2 infection measured by RT-PCR and/or bAb levels against SARS-CoV-2 nucleocapsid protein (by Roche Elecsys) counted starting 14 days after the second dose of IP in participants with negative SARS-CoV-2 at baseline
• To evaluate the incidence of COVID-19 after vaccination with mRNA-1273 or placebo. COVID-19 is defined as clinical symptoms consistent with SARS-CoV-2 infection AND positive RT-PCR for SARS-CoV-2	• The incidence of the first occurrence of COVID-19 starting 14 days after the second dose of IP, where COVID-19 is defined as symptomatic disease based on the following criteria:<br>– The participant must have experienced at least TWO of the following systemic symptoms: Fever (≥ 38ºC/≥ 100.4ºF), chills, myalgia, headache, sore throat, new olfactory and taste disorder(s), OR<br>– The participant must have experienced at least ONE of the following respiratory signs/symptoms: cough, shortness of breath or difficulty breathing, OR clinical or radiographical evidence of pneumonia; AND<br>– The participant must have at least 1 NP swab, nasal swab, or saliva sample (or respiratory sample, if hospitalized) positive for SARS-CoV-2 by RT-PCR
**Exploratory Objectives**	**Exploratory Endpoints**
• To evaluate the genetic and/or phenotypic relationships of isolated SARS-CoV-2 strains to the vaccine sequence	• Alignment of genetic sequence of viral isolates with that of the vaccine sequence and comparison of bAb and nAb titers against isolated strain relative to prototype vaccine strain

Objectives	Endpoints
• To describe the ratio or profile of specific bAb relative to nAb in serum	• Relative amounts or profiles of S protein-specific bAb and specific nAb levels/titers in serum
• To characterize the clinical profile and immune responses of participants with COVID-19 or with SARS-CoV-2 infection	• Description of clinical severity and immune responses of participants who are identified as infected by SARS-CoV-2 (COVID-19)
• To evaluate the incidence of asymptomatic SARS-CoV-2 infection after vaccination with mRNA-1273 or placebo in participants with serologic evidence of infection at baseline	• GM value and GMFR of bAb levels against SARS-CoV-2 nucleocapsid protein (quantitative IgG) and % of participants with 2x, 3x, and 4x rise of bAb relative to baseline
**Exploratory Objectives for Part 1B:**	**Exploratory Endpoints for Part 1B:**
• To evaluate the safety of 2 doses of mRNA-1273	• MAAEs through the entire study period<br>• SAEs through the entire study period<br>• AESIs through the entire study period<br>• AEs leading to discontinuation from study participation through the last day of study participation
• To evaluate the incidence of SARS-CoV-2 or COVID-19 after vaccination with mRNA-1273	• The incidence of SARS-CoV-2 infection (symptomatic or asymptomatic infection) counted starting 14 days after the second dose of mRNA-1273<br>• To evaluate the incidence of asymptomatic SARS-CoV-2 infection after vaccination with mRNA-1273 measured by RT-PCR and/or bAb levels against SARS-CoV-2 nucleocapsid protein (by Roche Elecsys) counted starting 14 days after the second dose in participants with negative SARS-CoV-2 at baseline<br>• The incidence of the first occurrence of symptomatic COVID-19 starting 14 days after the second dose of mRNA-1273
**Objectives for Part 1C-1 – Homologous Booster Dose**	**Endpoints for Part 1C-1 – Homologous Booster Dose**
**Primary Objectives**	**Primary Endpoints**
• To evaluate the safety of the 50 µg BD of mRNA-1273	• Solicited local and systemic ARs through 7 days after BD<br>• Unsolicited AEs through 28 days after BD injection<br>• MAAEs through the entire study period<br>• SAEs through the entire study period<br>• AESIs through the entire study period

Objectives	Endpoints
	• AEs leading to discontinuation from study participation post BD through the last day of study participation
• To infer effectiveness of 50 µg of mRNA-1273 booster by establishing noninferiority of Ab response after the BD compared to the primary series of mRNA-1273. GM values of serum Ab and SRR of postbooster in Study P203 compared with primary series from young adult (18 to 25 years of age) recipients of mRNA-1273 in the clinical endpoint efficacy trial (Study P301)	Coprimary endpoints:<br>• GM value of postbooster (post Dose 3) Ab against ancestral strain in Study P203 as compared to post primary series (post Dose 2) against ancestral strain in the young adults in Study P301<br>• SRR of postbooster/Dose 3 from baseline (pre Dose 1) as compared to post Dose 2 from baseline (pre Dose1) against ancestral strain in the young adults in Study P301, using 4-fold rise definition<br>– Seroresponse is defined as Ab value change from baseline (pre Dose 1) below the LLOQ to ≥4 × LLOQ, or at least a 4-fold rise if baseline is ≥LLOQ
**Key Secondary Objective**	**Key Secondary Endpoint**
• To evaluate immune response elicited by the 50 µg prototype booster of mRNA-1273 against variant(s) of interest	• GM value of postbooster (post Dose 3) Ab against circulating strain as compared to post primary series (post Dose 2) against circulating strain<br>• SRR of postbooster/Dose 3 from baseline (pre Dose 1) as compared to post Dose 2 from baseline (pre Dose1) against circulating strain using 4-fold rise definition
**Exploratory Objectives**	**Exploratory Endpoints**
• To evaluate the persistence of the immune response of the BD of mRNA-1273 vaccine (50 µg) as assessed by the level of SARS-CoV-2 S2P specific bAb through 1 year after BD	• The GM values of SARS-CoV-2 S2P specific bAb on BD-Day 1, BD-Day 29 (1 month after BD), BD-Day 181 (6 months after BD), and BD-Day 361 (1 year after BD)
• To evaluate the persistence of the immune response of the BD of mRNA-1273 vaccine (50 µg) as assessed by the level of nAb through 1 year after BD	• The GM values of SARS-CoV-2-specific nAb on BD-Day 1, BD-Day 29 (1 month after BD), BD-Day 181 (6 months after BD), and BD-Day 361 (1 year after BD)
• To evaluate the incidence of SARS-CoV-2 infection or COVID-19 after vaccination with mRNA-1273	• The incidence of SARS-CoV-2 infection (symptomatic or asymptomatic infection) counted starting 14 days after BD of mRNA-1273<br>• To evaluate the incidence of asymptomatic SARS-CoV-2 infection after vaccination with mRNA-1273 measured by RT-PCR and/or bAb levels against SARS-CoV-2 nucleocapsid protein (by Roche Elecsys) counted starting 14 days after

Objectives	Endpoints
	BD in participants with negative SARS-CoV-2 at baseline or prebooster<br>• The incidence of the first occurrence of symptomatic COVID-19 starting 14 days after BD of mRNA-1273
**Objectives for Part 1C-2 – Heterologous Booster Dose**	**Endpoints for Part 1C-2 – Heterologous Booster Dose**
**Primary Objectives**	**Primary Endpoints**
• To evaluate the safety of the 50 µg BD of mRNA-1273 in participants who received non-Moderna COVID-19 primary series vaccination	• Solicited local and systemic ARs through 7 days after BD<br>• Unsolicited AEs through 28 days after BD injection<br>• MAAEs through the entire study period<br>• SAEs through the entire study period<br>• AESIs through the entire study period<br>• AEs leading to discontinuation from study participation post BD through the last day of study participation
• To evaluate immune response elicited by the 50 µg booster of mRNA-1273 in participants who received non-Moderna COVID-19 primary series vaccination	• GM value of postbooster Ab (BD-Day 29) against ancestral strain
**Key Secondary Objective**	**Key Secondary Endpoint**
• To evaluate immune response elicited by the 50 µg prototype booster of mRNA-1273 against variant(s) of interest in participants who received non-Moderna COVID-19 primary series vaccination	• GM value of postbooster Ab (BD-Day 29) against circulating strain
**Exploratory Objectives**	**Exploratory Objectives**
• To evaluate the persistence of the immune response of the BD of mRNA-1273 vaccine (50 µg) as assessed by the level of SARS-CoV-2 S2P specific bAb through 1 year after BD in participants who received non-Moderna COVID-19 primary series vaccination	• The GM values of SARS-CoV-2 S2P specific bAb on BD-Day 1, BD-Day 29 (1 month after BD), BD-Day 181 (6 months after BD), and BD-Day 361 (1 year after BD)
• To evaluate the persistence of the immune response of the BD of mRNA-1273 vaccine (50 µg) as assessed by the level of nAb through 1 year after BD in participants who received non-Moderna COVID-19 primary series vaccination	• The GM values of SARS-CoV-2-specific nAb on BD-Day 1, BD-Day 29 (1 month after BD), BD-Day 181 (6 months after BD), and BD-Day-361 (1 year after BD)

Objectives	Endpoints
• To evaluate the incidence of SARS-CoV-2 infection or COVID-19 after booster vaccination with mRNA-1273 in participants who received non-Moderna COVID-19 primary series vaccination	• The incidence of SARS-CoV-2 infection (symptomatic or asymptomatic infection) counted starting 14 days after BD of mRNA-1273<br>• To evaluate the incidence of asymptomatic SARS-CoV-2 infection after vaccination with mRNA-1273 measured by RT-PCR and/or bAb levels against SARS-CoV-2 nucleocapsid protein (by Roche Elecsys) counted starting 14 days after BD in participants with negative SARS-CoV-2 at baseline or prebooster<br>• The incidence of the first occurrence of symptomatic COVID-19 starting 14 days after BD of mRNA-1273

Abbreviations: Ab = antibody; AE = adverse event; AESI = adverse event of special interest; AR = adverse reaction; bAb = binding antibody; BD = booster dose; COVID-19 = coronavirus disease 2019; GM = geometric mean; GMFR = geometric mean fold rise; IP = investigational product; LLOQ = lower limit of quantification; MAAE = medically attended adverse event; nAb = neutralizing antibody; NP = nasopharyngeal; RT-PCR = reverse transcriptase polymerase chain reaction; S = spike; S2P = S protein; SAE = serious adverse event; SARS-CoV-2 = Severe Acute Respiratory Syndrome coronavirus 2; SRR = seroresponse rate.

Table 2: Part 2 Objectives and Endpoints

Primary Objectives	Primary Endpoints
• To evaluate the safety and reactogenicity of the 50 µg mRNA-1273 vaccine administered in 2 doses 28 days apart.	• Solicited local and systemic ARs through 7 days after each injection<br>• Unsolicited AEs through 28 days after each injection<br>• MAAEs through the entire study period<br>• SAEs through the entire study period<br>• AESIs through the entire study period<br>• AEs leading to discontinuation from study participation from Dose 1 through the last day of study participation
• To evaluate immune response elicited by the 50 µg mRNA-1273 vaccine administered in 2 doses 28 days apart.	• GM value of post Dose 2 (Day 57) Ab against ancestral strain in Part 2 of Study P203<br>• SRR of post Dose 2 from baseline (pre Dose 1) in Part 2 of Study P203 against ancestral strain, using 4-fold rise definition<br>– Seroresponse is defined as Ab value change from baseline (pre Dose 1) below the LLOQ to ≥4 × LLOQ, or at least a 4-fold rise if baseline is ≥LLOQ

Exploratory Objectives	Exploratory Endpoints
• To evaluate the persistence of the immune response of 50 µg mRNA-1273 vaccine administered in 2 doses 28 days apart as assessed by the level of SARS-CoV-2 S2P specific bAb through 1 year after Dose 2	• The GM values of SARS-CoV-2 S2P specific bAb on Day 1, Day 57 (1 month after Dose 2), Day 209 (6 months from Dose 2), Day 394 (1 year from Dose 2)
• To evaluate the persistence of the immune response of the 50 µg mRNA-1273 vaccine as assessed by the level of nAb through 1 year after Dose 2	• The GM values of SARS-CoV-2-specific nAb on Day 1, Day 57 (1 month after Dose 2), Day 209 (6 months from Dose 2), Day 394 (1 year from Dose 2)
• To evaluate the incidence of SARS-CoV-2 infection or COVID-19 after vaccination with mRNA-1273.	• The incidence of SARS-CoV-2 infection (symptomatic or asymptomatic infection) counted starting 14 days after Dose 2 of mRNA-1273<br>• To evaluate the incidence of asymptomatic SARS-CoV-2 infection after vaccination with mRNA-1273 measured by RT-PCR and/or bAb levels against SARS-CoV-2 nucleocapsid protein (by Roche Elecsys) counted starting 14 days after Dose 2 in participants with negative SARS-CoV-2 at baseline<br>• The incidence of the first occurrence of symptomatic COVID-19 starting 14 days after Dose 2 of mRNA-1273

Abbreviations: Ab = antibody; AE = adverse event; AESI = adverse event of special interest; AR = adverse reaction; bAb = binding antibody; COVID-19 = coronavirus disease 2019; GM = geometric mean; GMFR = geometric mean fold rise; LLOQ = lower limit of quantification; MAAE = medically attended adverse event; nAb = neutralizing antibody; RT-PCR = reverse transcriptase polymerase chain reaction; S2P = S protein; SAE = serious adverse event; SARS-CoV-2 = Severe Acute Respiratory Syndrome coronavirus 2; SRR = seroresponse rate.

Table 3: Part 3 Objectives and Endpoints

Primary Objectives	Primary Endpoints
• To evaluate the safety and reactogenicity of the 50 µg mRNA-1273.222 vaccine administered in 2 doses 6 months apart.	• Solicited local and systemic ARs through 7 days after each injection.<br>• Unsolicited AEs through 28 days after each injection.<br>• MAAEs through the entire study period.<br>• SAEs through the entire study period.<br>• AESIs through the entire study period.<br>• AEs leading to withdrawal from study participation from Dose 1 through the last day of study participation.<br>• AEs leading to discontinuation from dosing from Dose 1 through the last day of study participation.

• To infer effectiveness of the 50 µg mRNA-1273.222 vaccine based on immune response against SARS-CoV-2 VOC (Omicron BA.4/BA.5) and ancestral strain obtained 28 days post Dose 1 in the baseline SARS-CoV-2 positive population.	• GM value of post Dose 1 (Day 29) of mRNA-1273.222 Ab against SARS-CoV-2 VOC (Omicron BA.4/BA.5) in adolescents who are baseline SARS-CoV-2 positive in Part 3 of Study P203 compared to that of post primary series of mRNA-1273 (post Dose 2 [Day 57] of 100 µg) in young adults 18 to 25 years of age who are baseline SARS-CoV-2 negative in Study P301 (superiority testing).<br>• GM value of post Dose 1 (Day 29) of mRNA-1273.222 Ab against ancestral strain in adolescents who are baseline SARS-CoV-2 positive in Part 3 of Study P203 compared to that of post primary series of mRNA-1273 (post Dose 2 [Day 57] of 100 µg) in young adults 18 to 25 years of age who are baseline SARS-CoV-2 negative in Study P301 (noninferiority testing).
**Secondary Objectives**	**Secondary Endpoints**
• To evaluate immune response elicited by 50 µg mRNA-1273.222 vaccine administered in 2 doses 6 months apart based on immune responses against Omicron BA.4/BA.5 and ancestral strain obtained 28 days post Dose 1 or 28 days post Dose 2.	• SRR of post Dose 1 (Day 29) from baseline (pre Dose 1) against Omicron BA.4/BA.5 in adolescents who are baseline SARS-CoV-2 positive in Part 3 of Study P203 compared to that of post primary series of mRNA-1273 (post Dose 2 [Day 57] of 100 µg) in adults 18 to 25 years of age in Study P301.<br>• SRR of post Dose 1 (Day 29) from baseline (pre Dose 1) against ancestral strain in adolescents who are baseline SARS-CoV-2 positive in Part 3 of Study P203 compared to that of post primary series of mRNA-1273 (post Dose 2 [Day 57] of 100 µg) in adults 18 to 25 years of age in Study P301.<br>• GM value of post Dose 2 (Day 209) of mRNA-1273.222 Ab against Omicron BA.4/BA.5 in Part 3 of Study P203 compared to that of post primary series of mRNA-1273 (post Dose 2 [Day 57] of 100 µg) in adults 18 to 25 years of age in Study P301.<br>• GM value of post Dose 2 (Day 209) of mRNA-1273.222 Ab against ancestral strain in Part 3 of Study P203 compared to that of post primary series of mRNA-1273 (post Dose 2 [Day 57] of 100 µg) in adults 18 to 25 years of age in Study P301.<br>• SRR of post Dose 2 (Day 209) from baseline (pre Dose 1) against Omicron BA.4/BA.5 in Part 3 of Study P203 compared to that of post primary series of mRNA-1273 (post Dose 2 [Day 57] of 100 µg) in adults 18 to 25 years of age in Study P301.<br>• SRR of post Dose 2 (Day 209) from baseline (pre Dose 1) against ancestral strain in Part 3 of Study P203 compared to that of post primary series of

	mRNA-1273 (post Dose 2 [Day 57] of 100 µg) in adults 18 to 25 years of age in Study P301.<br>*Note: Seroresponse is defined as an Ab value change from baseline (pre Dose 1) below the LLOQ to ≥4 × LLOQ, or at least a 4-fold rise if baseline is ≥ LLOQ.*
• To evaluate immune response elicited by 50 µg mRNA-1273.222 vaccine administered in 2 doses 6 months apart against other variant(s) of interest obtained 28 days post Dose 1 or 28 days post Dose 2.	• GM value of post Dose 1 (Day 29) of mRNA-1273.222 Ab against other variant(s) of interest in Part 3 of Study P203 as compared to that of post primary series of mRNA-1273 (post Dose 2 [Day 57] of 100 µg) in adults 18 to 25 years of age in Study P301.<br>• GM value of post Dose 2 (Day 209) of mRNA-1273.222 Ab against other variant(s) of interest in Part 3 of Study P203 as compared to that of post primary series of mRNA-1273 (post Dose [Day 57] 2 of 100 µg) in adults 18 to 25 years of age in Study P301.
**Exploratory Objectives**	**Exploratory Endpoints**
• To evaluate the persistence of the immune response of 50 µg mRNA-1273.222 vaccine, as assessed by the level of SARS-CoV-2 S2P specific bAb through 6 months after Dose 2.	• The GM values of SARS-CoV-2 S2P specific bAb on Day 1, Day 29 (1 month after Dose 1), Day 85 (3 months after Dose 1), Day 181 (Dose 2), Day 209 (1 month after Dose 2) and Day 361 (6 months after Dose 2).
• To evaluate the persistence of the immune response of the 50 µg mRNA-1273.222 vaccine as assessed by the level of nAb through 6 months after Dose 2.	• The GM values of SARS-CoV-2-specific nAb on Day 1, Day 29 (1 month after Dose 1), Day 85 (3 months after Dose 1), Day 181 (Dose 2), and Day 209 (1 month after Dose 2), and Day 361 (6 months after Dose 2).
• To evaluate the incidence COVID-19 after vaccination with mRNA-1273.222.	• The incidence of the first occurrence of symptomatic COVID-19 starting 14 days after Dose 1 and Dose 2 of mRNA-1273.222.

Abbreviations: Ab = antibody; AE = adverse event; AESI = adverse event of special interest; AR = adverse reaction; bAb = binding antibody; COVID-19 = coronavirus disease 2019; GM = geometric mean; LLOQ = lower limit of quantification; MAAE = medically attended adverse event; nAb = neutralizing antibody; RT-PCR = reverse transcriptase polymerase chain reaction; S2P = S protein; SAE = serious adverse event; SARS-CoV-2 = Severe Acute Respiratory Syndrome coronavirus 2; SRR = seroresponse rate; VOC = variant of concern.

3. STUDY DESIGN

3.1. General Design

This study is split into 2 parts and the overall design schematic can be seen in Figure 1.

Figure 1: Overall Design Schema

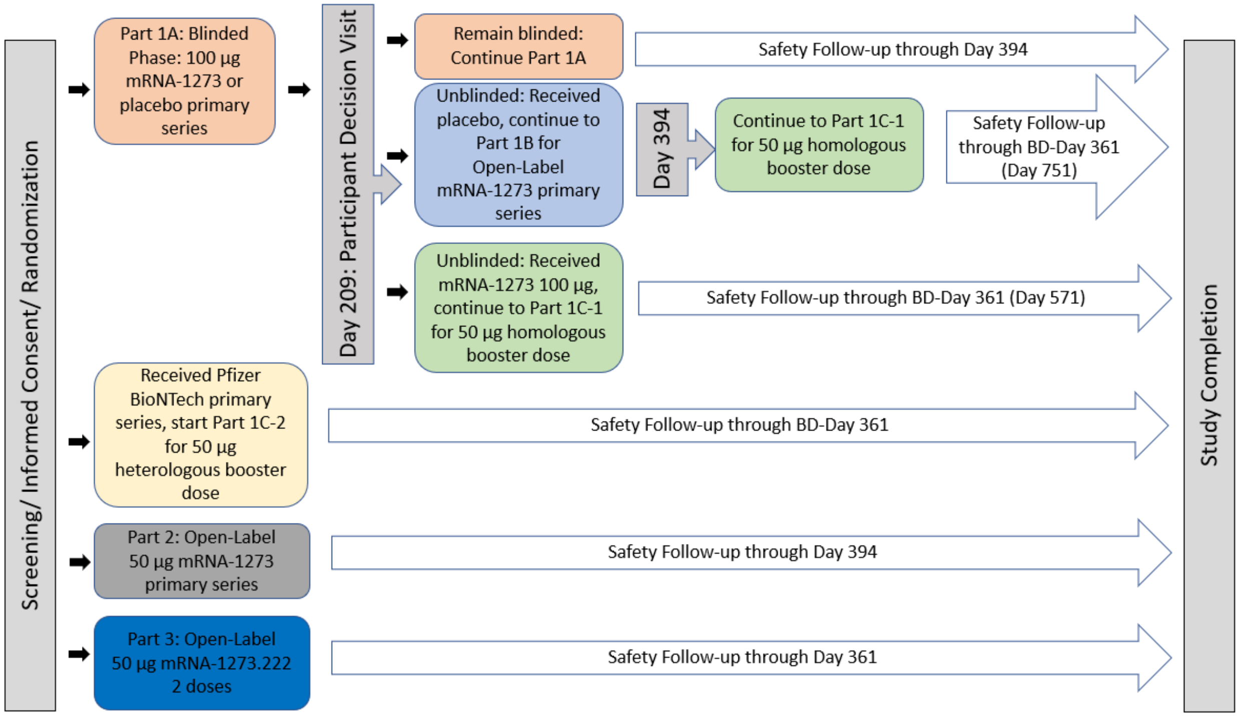

BD = booster dose.

3.1.1. Part 1 Study Design

This is a three-part, Phase 2/3 study: Part 1A, Part 1B, and Part 1C. Participants in Part 1A, the Blinded Phase, are blinded to their treatment assignment.

Part 1B, the Open-label Observational Phase of this study, is designed to offer participants who received placebo in Part 1A of this study and who meet the EUA eligibility criteria an option to receive mRNA-1273 in an open-label fashion (Figure 3). Participants who received mRNA-1273 (100 µg) in Part 1A of this study will proceed to Part 1B after they are unblinded and will continue to follow the Part 1A SoA (Table 10). Part 1C-1, the Homologous Booster Dose Phase, is designed to offer participants in Part 1A and Part 1B, and who are at least 5 months from the last dose, the option to request a BD of 50 µg mRNA-1273. Part 1C-2, the Heterologous Booster Phase, is designed to provide participants who completed non-Moderna primary COVID-19 vaccination series under EUA (ie, Pfizer) a 50 µg BD of mRNA-1273 at least 3 months from the last dose.

Participants who received mRNA-1273 in Part 1A will be in the study approximately 25 months if a BD is received in Part 1C-1, which includes 1 month for Screening (Day -28 to Day 1), up to 12 months for dosing (on Day 1, Day 29, and Day 209 for BD), and 12 months for follow-up.

Participants who received placebo in Part 1A will be in the study for approximately 25 months total if a BD is received in Part 1C-1, which includes approximately 9 months in Part 1A and approximately 4 months of follow-up following their second dose of mRNA-1273 in Part 1B before entering Part 1C-1 or before BD is received; or approximately 12 months of follow-up.

Participants that decline unblinding or decline to receive a BD will be in the study approximately 14 months total, which includes 1 month for Screening (Day -28 to Day 1), 1 month for dosing (on Day 1 and Day 29), and 12 months for follow-up.

Participants in Part 1C-2 will be in the study for approximately 12 months total, which includes 7 days for Screening (Day -7 to Day 1), 1 day of dosing (BD-Day 1), and 12 months of follow-up.

This study will be conducted in compliance with the protocol, GCP, and all applicable regulatory requirements.

3.1.1.1. Part 1A, the Blinded Phase

The blinded phase of this study is a randomized, observer-blind, placebo-controlled study intended to infer the effectiveness of mRNA-1273 in an adolescent population aged 12 to < 18 years. The study includes 2 arms: (i) 100 µg of mRNA-1273, and (ii) placebo. Approximately, 3000 participants between 12 to < 18 years of age will be randomly assigned in a 2:1 ratio to receive mRNA-1273 (n=2000) or placebo (n=1000).

The schematic of study arms and major study events for Part 1A is illustrated in Figure 2 and the SoA for Part 1A is located in Table 10.

The goal of the study is to seek an indication for use of mRNA-1273 (100 µg IM, given as 2 injections, 28 days apart) in the 12 to < 18 years age group. The basis for demonstrating vaccine effectiveness is proposed to be met by serum Ab response measured in this adolescent age group. The approach to inferring vaccine effectiveness will depend on whether an accepted serum Ab threshold conferring protection against COVID-19 has been established. If an Ab threshold of protection has been established, effectiveness will be inferred based on the proportion of adolescent study participants with serum Ab levels (on Day 57) that meet or exceed the Ab threshold. If an Ab threshold of protection has not been established, effectiveness will be inferred by demonstrating noninferiority of both (i) the GM value of serum nAb and (ii) the SRR from adolescent participants compared with those from young adults (18 to 25 years of age) enrolled in the ongoing clinical endpoint efficacy trial (Study P301). The statistical parameters to infer effectiveness are described in Section 2.

This study in adolescents will monitor all participants for a total of 12 months following the second dose of vaccine or placebo. Safety assessments will include solicited ARs (7 days after each injection), unsolicited AEs (28 days after each injection), MAAEs, SAEs, and AESIs (including MIS-C) throughout the study period.

Blood samples will be collected from all participants at baseline (Day 1), Day 57 (28 days after Dose 2), Day 209 (6 months after Dose 2), and Day 394 for measurement of SARS-CoV-2-specific bAb and nAb responses. Blood samples will also be tested for the development of Ab directed against nonvaccine antigen (eg, Ab against the nucleocapsid protein), which will signify infection with SARS-CoV-2. The incidence of SARS-CoV-2

infection among vaccine recipients and placebo recipients will be compared to assess the potential for mRNA-1273 to reduce the rate of infection in vaccine recipients.

Figure 2: Study Schema (Part 1A, Blinded Phase)

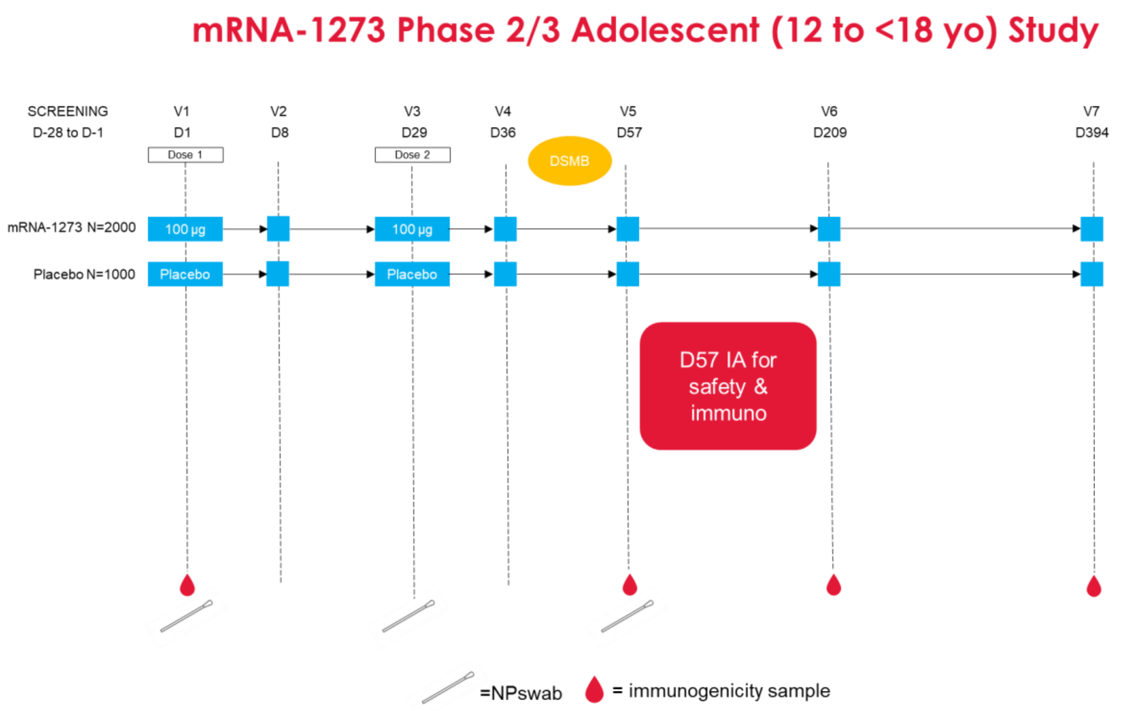

Abbreviation: D = day; DSMB = Data Safety Monitoring Board, IA = interim analysis, immune = immunogenicity, NP = nasopharyngeal or nasal swab, V = visit, yo = years old.

Part 1A, the Blinded Phase, comprises 8 scheduled visits including a Screening Visit and 7 scheduled visits, of which Visit 2 and Visit 4 will be virtual/telephone visits and the other visits will be in-clinic visits.

The study duration will be approximately 14 months, which includes 1 month for Screening (Day -28 to Day 1), 1 month for dosing (on Day 1 and Day 29), and, for participants who received mRNA-1273 in Part 1A, 12 months of follow-up after the second dose to monitor for safety, immunogenicity, and efficacy. Participants who received placebo in Part 1A will still be in the study for approximately 14 months total but will be followed for approximately 4 months following their second dose of mRNA-1273 in Part 1B before receiving a BD.

Note: Day 0 and Day 1 may be combined on the same day (Table 10).

After providing informed consent/assent, participants will undergo Screening assessments to determine study eligibility. Screening assessments (Table 10) must be completed after signing the ICF/assent form. The investigator will review study entry criteria to determine the participant eligibility during the Screening Period.

Eligible participants will enter the Treatment Period.

On Day 1, after the completion of the scheduled assessments (Table 10), participants will be administered a single IM dose of mRNA-1273 (100 µg) or placebo (procedures will be detailed in the mRNA-1273-P203 Pharmacy Manual). Participants will be closely monitored for safety

and will remain at the study site for observation for at least 30 minutes after dosing. On Day 29, the second dose of IP will be administered. Participants will be monitored for 12 months after the second dose of IP for safety and immunogenicity assessments.

To test for the presence of SARS-CoV-2 by RT-PCR, NP or nasal swab samples will be collected on each day of injection prior to dosing and on Day 57 (28 days post Dose 2), according to the SoA (Table 10).

During the course of the study, participants who meet prespecified disease criteria that suggest possible SARS-CoV-2 infection will be asked to contact the study site to arrange for a prompt, thorough, and careful assessment, including an NP or nasal swab sample to be tested for the presence of SARS-CoV-2 by RT-PCR. Confirmed, symptomatic cases of SARS-CoV-2 infection will be captured as MAAEs and reported in an expedited time frame to the Sponsor (Section 7.4.3).

All participants will be monitored for safety and reactogenicity and provide predose and postdose blood specimens for immunogenicity through 12 months after the second dose of mRNA-1273.

Participants will be instructed on the day of the first dose (Day 1) and reminded on the day of the second dose (Day 29) how to document and report solicited local or systemic ARs in a provided eDiary. Solicited ARs, unsolicited AEs, MAAEs, AEs leading to withdrawal, AESIs, and SAEs will be assessed as described in Section 7.1, according to the timepoints in the SoA (Table 10).

Blood sampling for immunogenicity testing is scheduled throughout the study: on the day of injection before the first dose and 1, 6, and 12 months after the second dose.

Participants may experience AEs that necessitate an unscheduled visit, including situations when the investigator asks a participant to return to the study clinic for an unscheduled visit following the report of an AE. Additional examinations may be conducted at these visits as necessary to ensure the safety and well-being of participants during the study. eCRF should be completed for each unscheduled visit.

3.1.1.2. Part 1B, the Open-label Observational Phase

Part 1B, the Open-label Observational Phase of the study, will be prompted by the authorization of a COVID-19 vaccine under an EUA for any persons under the age of 18 years. Participants will be transitioned to Part 1B of the study as their age group becomes EUA-eligible. This transition permits all ongoing study participants to eventually be informed of the availability and eligibility criteria of any COVID-19 vaccine made available under an EUA and the option to offer all ongoing study participants an opportunity to schedule a Participant Decision Visit to know their original treatment assignment (placebo vs. mRNA-1273 100 µg vaccine).

Part 1B provides the opportunity for study participants to be informed regarding the EUA, to be unblinded to their original assignment (mRNA-1273 or placebo), and, for those who previously received placebo, to actively request to receive 2 doses of mRNA-1273 (100 µg) vaccine.

Figure 3: Study Schema (Part 1B, Open-label Observational Phase)

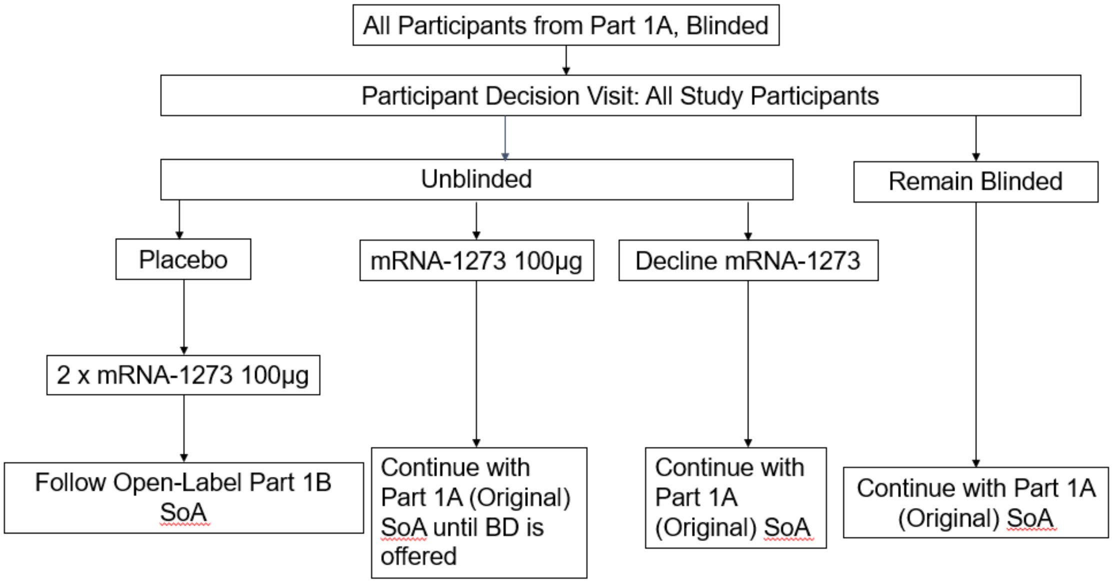

Abbreviation: BD = booster dose; SoA = Schedule of Assessments.

At the Participant Decision Clinic Visit (Table 11), EUA-eligible participants will:

- Be given the option to be unblinded as to their original group assignment (placebo vs. mRNA-1273 vaccine [100 µg]),
- Be counseled about the importance of continuing other public health measures to limit the spread of disease including social distancing, wearing a mask, and hand-washing,
- Sign a revised ICF and assent if not signed at a previous clinic visit, and
- Provide an NP or nasal swab for RT-PCR for SARS-CoV-2 and a blood sample for serology and immunogenicity.

Participants that decline unblinding will remain in Part 1A and follow the Part 1A SoA in Table 10.

After the Participant Decision Clinic Visit, participants that consent to unblinding will follow the Part 1A SoA (Table 10) or Part 1B SoA (Table 12) as follows:

- <u>Participants received placebo in Part 1A and consent to receiving 2 doses of mRNA-1273 in Part 1B</u>: These participants will proceed to Part 1B and follow the Part 1B SoA in Table 12 until BD is offered.
- <u>Participants received 2 doses of mRNA-1273 in Part 1A:</u> Due to statistical considerations, these participants will be considered in the Open-label Observational Phase but will continue to follow the Part 1A SoA in Table 10 until BD is offered.

3.1.1.3. Part 1C-1, Homologous Booster Phase

Part 1C-1 Homologous Booster Phase is designed to offer participants in Part 1A and Part 1B, who are at least 5 months from the last dose, the option to request a BD (50 µg) of mRNA-1273.

If eligible, each study participant will receive a notification letter and will be asked to schedule a BD-1 Visit at their study site. Principal investigators should consider current local public health guidance for administration of COVID-19 vaccines under EUA and marketing authorization (if any) when determining the scheduling priority of participants.

Figure 4: Study Schema (Part 1C-1, Open-label Homologous Booster Dose Observational Phase)

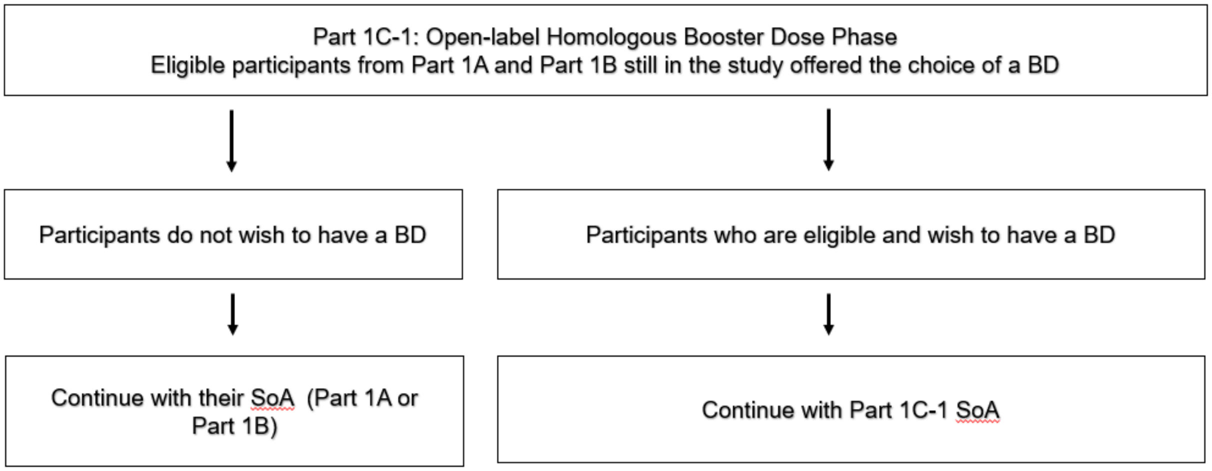

Abbreviations: BD = booster dose; D = day; SoA = Schedule of Assessments

At the BD-1 Visit, each participant will:

- Be encouraged to remain in the ongoing study,
- Sign a revised ICF that includes both updated safety information relevant to the ongoing study and a BD, and the option to receive a BD,
- Be given the option to receive a BD consisting of a 50 µg dose of mRNA-1273,
- Be counseled about the importance of continuing other public health measures to limit the spread of disease including social distancing, wearing a mask, and hand-washing.

At the BD-1 Visit, participants who request a BD and are eligible will have the following study site visits and complete scheduled activities (subject to investigational vaccine availability) according to the Part 1C-1 Supplemental SoA:

- BD-1 Visit: Participants will receive a single 50 µg dose of mRNA-1273,
- BD-1a Visit: All participants who chose to receive BD, Day 4, 3 days after BD on Day 1,
- BD-2 Visit: Day 29, 28 days after the BD on Day 1,
- BD-3 Visit: Day 181, 180 days after the BD on Day 1,

- BD-4 Visit: Day 361, 360 days after the BD on Day 1.

Participants that crossed over from placebo to mRNA-1273 and decline BD will continue with Part 1B SoA (Table 12).

Participants that received mRNA-1273 in Part 1A and decline BD will continue with in Part 1A SoA (Table 10).

The investigator is responsible for conducting all assessments as specified in the Part 1C-1 Supplemental SoA, according to the schedule. As this Supplemental SoA is intended to occur in addition to the original SoAs being followed by all participants in Part 1A or Part 1B, there is a possibility for study visits to overlap (ie, Part 1C-1 BD-Day 1 and study visit in Part 1A or Part 1B that coincides with Part 1C-1 BD-Day 1). If visits overlap according to respective visit windows, a single visit may be done with the combined study procedures completed once. Any participant who consented to receive a BD will follow Part 1C-1 SoA. In case a study visit in Part 1A or Part 1B coincides with Part 1C-1 BD-Day1, participants should follow Part 1C SoA.

Note: Participants enrolled in Part 1C-1 who have received the third (booster) dose in this study and would like to receive the bivalent COVID-19 vaccine, can receive it outside of Study P203. Participants who receive the updated bivalent COVID-19 vaccine may remain in the P203 study after receipt of the nonstudy vaccine. However, sites must report this as concomitant medication.

3.1.1.4. Part 1C-2, Heterologous Booster Phase

Part 1C-2 – Heterologous Booster Phase is designed to provide a BD (50 µg) of mRNA-1273 to eligible participants who completed primary COVID-19 vaccination series with a non-Moderna vaccine under EUA (ie, Pfizer, Table 4) and are at least 3 months from the last dose.

Table 4: Non-Moderna COVID-19 Vaccine Available Under Emergency Use Authorization

COVID-19 Vaccine – Primary Series	Primary Series Number of Doses
Pfizer-BioNTech	2 doses

Abbreviations: COVID-19 = coronavirus disease 2019.

Principal investigators should consider current local public health guidance for administration of COVID-19 vaccines under EUA and marketing authorization (if any) when determining the scheduling priority of participants.

At the BD-0/BD-1 Visit, each participant will:

- Sign an ICF,
- Be counseled about the importance of continuing other public health measures to limit the spread of disease including social distancing, wearing a mask, and hand-washing.

At the BD-1 Visit, participants will have the following study site visits and complete scheduled activities (subject to investigational vaccine availability) according to the Part 1C-2 Supplemental SoA (Table 14):

- BD-1 Visit: Participants will receive a single 50 µg dose of mRNA-1273,
- BD-1a Visit: Day 4, 3 days after BD on Day 1,

- BD-2 Visit: Day 29, 28 days after the BD on Day 1,
- BD-3 Visit: Day 181, 180 days after the BD on Day 1,
- BD-4 Visit: Day 361, 360 days after the BD on Day 1.

The investigator is responsible for conducting all assessments as specified in the Part 1C-2 Supplemental SoA, according to the schedule (Table 14).

Note: Due to recent emergence of a more divergent variant of concern (ie. Omicron), an amendment to the protocol was planned to replace the current investigative product (mRNA-1273) with a bivalent vaccine (mRNA-1273.222) for a more comprehensive protection. This amendment will aim to assess primary series of 50 µg bivalent mRNA-1273.222 in Part 3 of the study. As a result of this amendment, Part 1C-2 enrollment was discontinued effective 16 August 2022. Participants enrolled in Part 1C-2 who have received the third (booster) dose in this study and would like to receive the bivalent COVID-19 vaccine, can receive it outside of Study P203. Participants who receive the updated bivalent COVID-19 vaccine may remain in the P203 study after receipt of the nonstudy vaccine. However, sites must report this as concomitant medication.

3.1.2. Part 2 Study Design

Part 2 is an open-label design. The study will evaluate the safety, reactogenicity, and effectiveness of a 50 µg primary series of mRNA-1273 SARS-CoV-2 vaccine in healthy adolescents 12 to < 18 years of age.

The schematic of study arms and major study events for Part 2 is illustrated in Figure 5 and the SoA for Part 2 is located in Table 15.

This study in adolescents will monitor all participants for a total of 12 months following Dose 2 of vaccine. Safety assessments will include solicited ARs (7 days after each injection), unsolicited AEs (28 days after each injection), MAAEs, SAEs, and AESIs (including MIS-C) throughout the study period and AEs leading to discontinuation from dosing and/or withdrawal from study.

Blood samples will be collected from all participants at baseline (Day 1) and at specified visits presented in Part 2 SoA (Table 15) for measurement of SARS-CoV-2 specific binding and nAb responses. Blood samples will also be tested for the development of Ab directed against nonvaccine antigen (eg, Ab against the nucleocapsid protein), which will signify infection with SARS-CoV-2. Blood sample will be collected 3 days after Dose 2 for future biomarker analysis (ie, inflammatory and/or cardiac biomarkers).

The incidence of SARS-CoV-2 infection after mRNA-1273 vaccination will be evaluated.

Figure 5: Study Schema (Part 2)

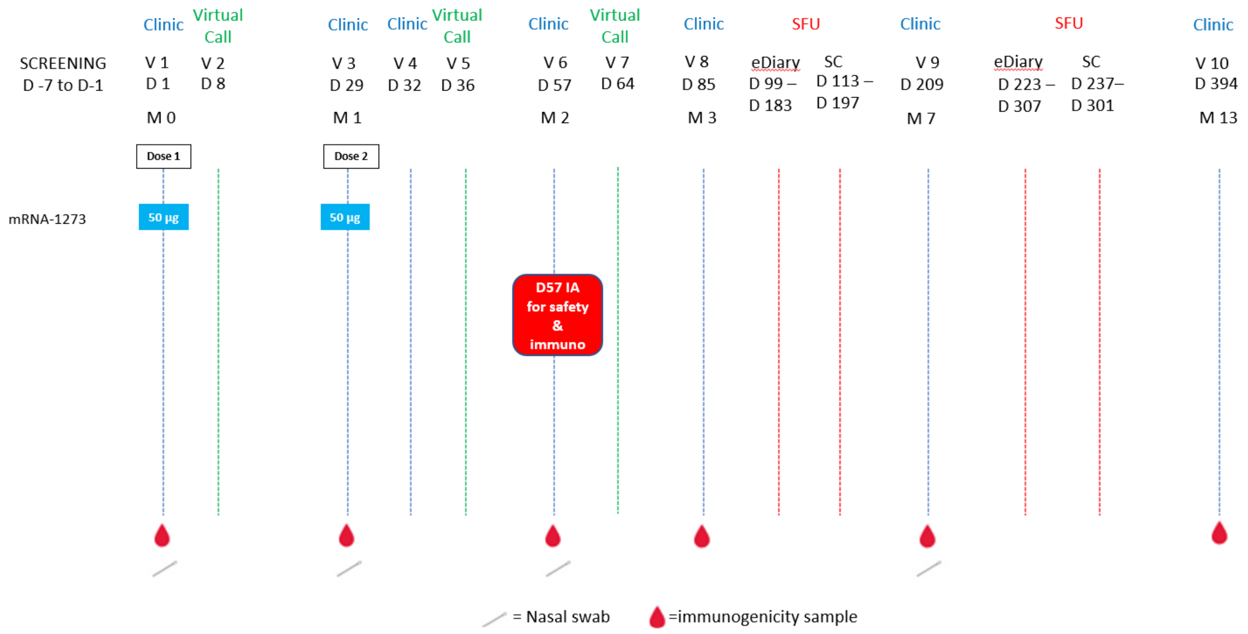

Abbreviation: D = day; IA = interim analysis; immuno = immunogenicity; SC = safety call; SFU = safety follow-up; V = visit.

Part 2 comprises 11 scheduled visits including a Screening Visit and 10 scheduled visits, of which Visit 2, Visit 5, and Visit 7 will be virtual/telephone visits and the other visits will be in-clinic visits. Participants who received mRNA-1273 in Part 2 will be in the study approximately 12 months, which includes 1 week for Screening (Day -7 to Day 1), 1 month for dosing (on Day 1, Day 29), and 12 months of follow-up after Dose 2.

This study will be conducted in compliance with the protocol, GCP, and all applicable regulatory requirements.

Note: Due to recent emergence of a more divergent variant of concern (ie. Omicron), an amendment to the protocol was planned to replace the current investigative product (mRNA-1273) with a bivalent vaccine (mRNA-1273.222) for a more comprehensive protection. This amendment will aim to assess primary series of 50 µg bivalent mRNA-1273.222 in Part 3 of the study. As a result of this amendment, Part 2 enrollment was discontinued effective 16 August 2022. Participants enrolled in Part 2 who have received the primary series or third (booster) dose in this study and would like to receive the bivalent COVID-19 vaccine, can receive it outside of Study P203. Participants who receive the updated bivalent COVID-19 vaccine may remain in the P203 study after receipt of the nonstudy vaccine. However, sites must report this as concomitant medication.

3.1.3. Part 3 Study Design

Part 3 is an open-label, single-arm design. The study will evaluate the safety, reactogenicity, and effectiveness of a 2-dose 50 µg primary series of mRNA-1273.222 SARS-CoV-2 vaccine, administered 6 months apart, in approximately at least 300 healthy adolescents 12 to <18 years of age.

The schematic of study arms and major study events for Part 3 is illustrated in Figure 6 and the SoA for Part 3 is described in Table 16.

This study in adolescents will monitor all participants 6 months following Dose 2 of vaccine. Safety assessments will include solicited ARs (7 days after each injection), unsolicited AEs (28 days after each injection), MAAEs, SAEs, and AESIs (including MIS-C) throughout the study period and AEs leading to discontinuation from dosing and/or withdrawal from study.

Blood samples will be collected from all participants at baseline (Day 1) and in visits specified in Part 3 SoA (Table 16) for measurement of SARS-CoV-2 specific bAB and nAb responses. Blood samples will also be tested for the development of Ab directed against nonvaccine antigen (eg, Ab against the nucleocapsid protein), which will signify infection with SARS-CoV-2. Blood samples will be collected 3 days after Dose 1 (Day 4) and 3 days after Dose 2 (Day 184), for future biomarker analysis (ie, inflammatory and/or cardiac biomarkers).

The incidence of SARS-CoV-2 infection after mRNA-1273.222 vaccination will be evaluated.

Participants who receive mRNA-1273.222 in Part 3 will be in the study for approximately12 months including 1 week for Screening (Day -7 to Day -1), 6 months for dosing (Day 1 to Day 181), and 6 months of follow-up after Dose 2.

Figure 6: Study Schema (Part 3)

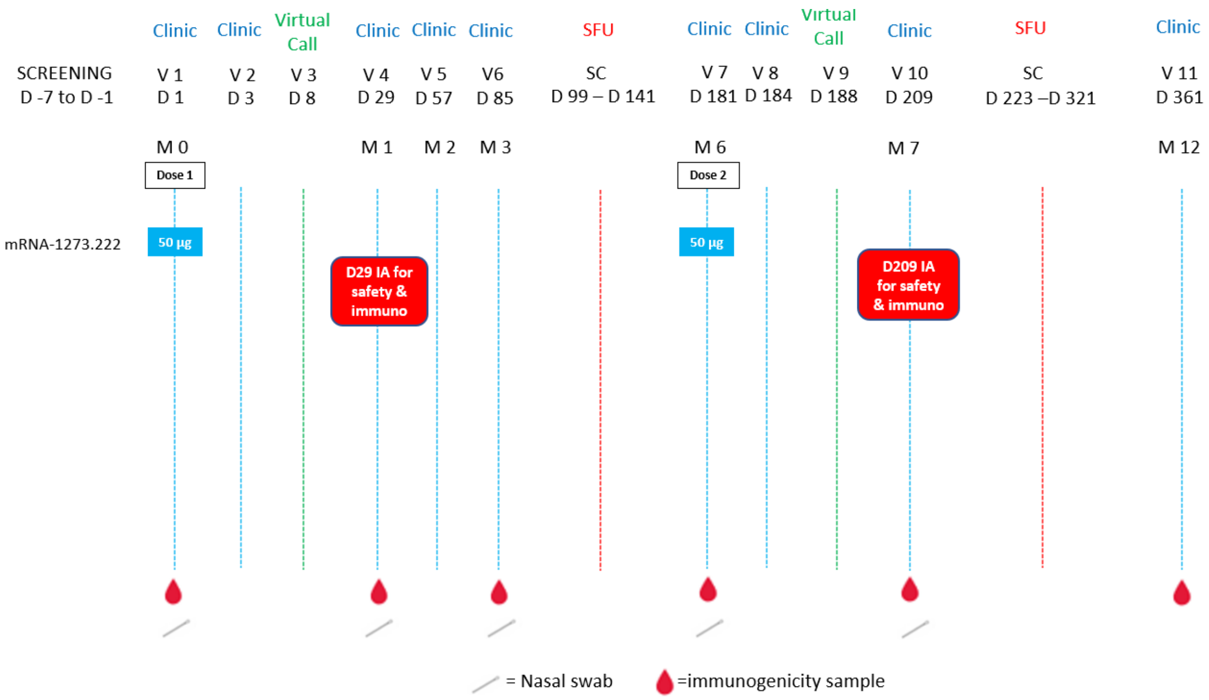

Abbreviation: D = day; IA = interim analysis; immuno = immunogenicity; M = month; SC = safety call; SFU = safety follow-up; V = visit.

3.2. Scientific Rationale for Study Design

The single age cohort in this Phase 2/3 study, 12 to < 18 years of age, was established to understand the tolerability and immunogenicity of 100 µg (Part 1A) and 50 µg (Part 1C-1, Part 1C-2, and Part 2) mRNA-1273, as well as 50 µg (Part 3) mRNA-1273.222 in an adolescent

population. The lower age boundary used in this study is consistent with the definition of adolescence provided by the American Academy of Pediatrics (Hardin et al 2017).

With SARS-CoV-2 expected to be circulating in the general population during the study, all participants will provide preinjection blood samples and postinjection blood samples for Ab analysis through 12 months after the last dose of IP during Part 1A, Part 2, and through 6 months after the last dose of IP in Part 3 of the study. In addition, participants will have NP or nasal swab samples collected, before the injections on Day 1, Day 29 or Day 181, on Day 57 or Day 209 and on Day 394 (Part 2). Furthermore, with any signs or symptoms or MAAE suggesting SARS-CoV-2 infection in a participant, an additional nasal or NP swab sample and a blood sample will be taken to confirm the diagnosis of SARS-CoV-2 via serology and RT-PCR. Additionally, clinical information will be carefully collected to evaluate the severity of the clinical case.

As it is possible that participants are naturally exposed to SARS-CoV-2 through community exposure, the NP or nasal swab samples collected before study injection and the serologic assays for Ab responses to nonvaccine antigen(s) may help discriminate between natural infection and vaccine-induced Ab responses, should such discrimination be needed.

Part 1B, is the Open-label Observational Phase. During Part 1B of the study, all ongoing study participants were permitted (a) to be informed of the availability and eligibility criteria of any COVID-19 vaccine made available under an EUA and (b) to schedule a study visit to know their original group assignment (placebo vs. mRNA-1273 vaccine). Part 1B also provided the opportunity for eligible study participants who previously received placebo to receive 2 doses of mRNA-1273 vaccine under open-label conditions.

Part 1C, the Booster Phase and Part 2, were prompted by the recent interim results of an ongoing Moderna Phase 2 study (mRNA-1273-P201). In Part A of Study mRNA-1273-P201, the time course and magnitude of antibody (both bAb and nAb) responses to mRNA-1273 was similar between 100 µg and 50 µg dose levels (both showing strong immunogenicity) at each postbaseline timepoint (Days 29, 43, 57, and 209), although the 100 µg dose group had numerically greater responses. In Part B of Study mRNA-1273-P201 in which participants who had received 2 doses of 50 µg or 100 µg of mRNA-1273 6-8 months prior were administered a 50 µg booster of mRNA-1273, participants demonstrated enhanced immune responses compared to preboost levels and met the noninferiority criteria stipulated in the FDA Guidance on EUA for Vaccines to Prevent COVID-19. In addition, interim results from Study mRNA-1273-P205 show that a 50 µg booster of mRNA-1273 increased nAb levels against Omicron approximately 37-fold compared to preboost levels. No new safety signals emerged upon administration of the BD in Study mRNA-1273-P201. Available data (DMID 21-0012) also show that heterologous or mixed series of COVID-19 vaccine-induced strong immune response in the adult population with persons who received an mRNA-based booster vaccination achieving a four-fold increase in their neutralization response more frequently than those who were boosted with Ad26.COV.S. (Atmar et al 2021).

Part 3 was prompted by preclinical data on bivalent vaccine (mRNA-1273.214 given as 2 dose primary series) showing broader neutralization across divergent lineages. Preclinical mice studies also show increased immunogenicity after BD of the BA.1 & BA.4/BA.5 Omicron Bivalent Vaccines (mRNA-1273.214 & mRNA-1273.222). Further, mRNA-1273.214 when given as 50 µg BD in Study P205 participants elicited superior nAb responses against the

Omicron subvariants BA.4, BA.5 compared with mRNA-1273 50 µg (nominal α of 0.05) and the BA.4, BA.5. nAb response was consistently higher in the mRNA-1273.214 group compared to the mRNA-1273 group in participants with and without prior SARS-CoV-2 infection (although higher GMT titers post BD were observed in those who were SARS-CoV-2 positive at pre-BD). Data also demonstrate antibody responses 180 days after the 50 µg mRNA-1273.211 BD were higher than those after a 50 µg mRNA-1273 BD (NCT04405076; Chalkias et al 2022a) against the ancestral SARS-CoV-2 and Beta, as well as Omicron BA.1 and Delta variants. In addition, the bivalent Omicron BA.4/BA.5-containing vaccine mRNA-1273.222 elicited superior neutralizing antibody responses against BA.4/BA.5 compared to mRNA-1273, with no safety concerns identified (Chalkias et al 2022c). Given the increasing prevalence of SARS-CoV-2 infection in the community (Clarke et al 2022), effectiveness of 50 µg mRNA-1273.222 post dose 1 will be evaluated as a primary objective under Part 3. Recent IA results from Study P203 Part 2 also support evaluation of immunogenicity of a single 50 µg-dose. Study P203 Part 2 interim results show higher immunogenicity post dose 1 of 50 µg mRNA-1273 in Study P203 unvaccinated baseline SARS-CoV-2 positive adolescent participants when compared to immunogenicity post primary series in unvaccinated baseline SARS-CoV-2 negative young adults in Study P301 (GMR of 4.322 [95% CI: 3.274, 5.707]).

Based on cumulative evidence, the benefit-risk profile of monovalent mRNA-1273 and bivalent mRNA-1273.222 vaccine is favorable, particularly in light of increasing breakthrough disease with the emergence of the Delta and Omicron variants.

3.3. Justification for Dose, Control Product, and Choice of Study Population

The 100 µg dose level is currently being investigated in a large Phase 3 efficacy study in adults 18 years of age and older; therefore, based on this and the results of the studies described in Section 1.2.2, the Sponsor intended to study a single dose level of 100 µg in Part 1A of this Phase 2/3 study in the adolescents age group of 12 to < 18 years of age.

At the time of study initiation, there were no licensed SARS-CoV-2 vaccines available, and so 0.9% sodium chloride was used as a placebo control for the safety and immunogenicity assessments. The mRNA-1273 vaccine and placebo injections look different, so administration was blinded in Part 1A of this study (Section 8.1).

Interim data from the current Part 1A Study P203 (based on a data snapshot of 08 May 2021) show that the primary immunogenicity objective was met by demonstrating noninferiority of both the (i) GM value of serum nAb and (ii) the seroconversion rate from adolescent participants compared with those from young adults (aged 18 to 25 years) enrolled in the ongoing Phase 3 efficacy study (Study P301).

Following potential EUA of a COVID-19 vaccine for persons under 18 years of age, a study amendment was designed to give EUA-eligible participants the opportunity to transition to Part 1B, the Open-label Observational Phase (Figure 3). Transitioning the study to Part 1B, open-label Observational Phase, permits (a) all ongoing study participants to be informed of the availability and eligibility criteria of any COVID-19 vaccine made available under an EUA and (b) the option to offer all ongoing study participants who request unblinding an opportunity to schedule a study visit to know their original group assignment (placebo vs. mRNA-1273 [100 µg

vaccine]). Part 1B, the Open-label Observational Phase, also provides the opportunity for study participants who previously received placebo to request to receive 2 doses of mRNA-1273 (100 µg) vaccine.

For Part 1C (provision for the option of a single 50 µg BD for eligible participants during the Open-Label Observational Phase) and for Part 2 (evaluation of 2 doses of 50 µg given 28 days apart), the selected 50 µg dose was based primarily on data from Study mRNA-1273-P201. For Part 1C-2, participants will be boosted at least 3 months post Dose 2. For Part 1C-1, participants are well beyond 3 months post Dose 2 and thus will not be changed to a BD at least 3 months post Dose 2. In Part A of Study mRNA-1273-P201, the time course and magnitude of antibody (both bAb and nAb) responses to mRNA-1273 was similar between 100 µg and 50 µg dose levels (both showing strong immunogenicity) at each postbaseline timepoint (Days 29, 43, 57, and 209), although the 100-µg dose group had numerically greater responses. In Part B of Study mRNA-1273-P201, administration of a 50 µg BD of mRNA-1273 6 months or more after the primary series, improved the immune responses to 1.7-fold the peak achieved after the primary vaccination series in the current mRNA-1273-P301 study, where efficacy of mRNA-1273 against COVID-19 was demonstrated. In addition, interim results from Study mRNA-1273-P205 show that 50 µg booster of mRNA-1273 increased nAb levels against Omicron approximately 37-fold compared to preboost levels.

Available data also show that heterologous or mixed series of COVID-19 vaccine-induced strong immune response in the adult population (Atmar et al 2021). In a recent DMID study (DMID 21-0012), interim data show Day 15 post-boost neutralization titers ranged from 676.1-901.8 IU50/ml for participants boosted with 100 µg mRNA-1273 compared to 31.2-382.2 IU50/ml for those boosted with Ad26.COV2.S, and 341.3-677.9 IU50/mL for those boosted with BNT162b2. In general, Day 15 titers post-boost were highest in mRNA-1273-primed participants, followed by BNT162b2 and Ad26.COV2.S, irrespective of the booster vaccine administered (Atmar et al 2021).

For Part 3, the rationale for assessment of 50 µg primary series of the bivalent Omicron containing vaccine mRNA-1273.222 is based on the preclinical data on bivalent vaccines given as primary series (mRNA-1273.214) and as BD (mRNA-1273.222) that show robust immunogenicity and broader neutralization across divergent lineages. Clinical data from mRNA-1273.214 bivalent vaccine (given as a 50 µg BD) also show consistent robust immunogenicity, regardless of SARS-CoV-2 infection status in participants (although higher GMT titers post BD were observed in those who were SARS-CoV-2 positive at pre-BD), with acceptable safety profile. Data also demonstrates antibody responses 180 days after the 50 µg mRNA-1273.211 BD were higher than those after a 50 µg mRNA-1273 BD (NCT04405076; Chalkias et al 2022a) against the ancestral SARS-CoV-2 and Beta, as well as Omicron BA.1 and Delta variants. In addition, the bivalent Omicron BA.4/BA.5-containing vaccine mRNA-1273.222 elicited superior neutralizing antibody responses against BA.4/BA.5 compared to mRNA-1273, with no safety concerns identified (Chalkias et al 2022c).

These data together with increasing prevalence of SARS-CoV-2 infection in the community (Clarke et al 2022) as well as recent interim results from Study P203 Part 2 showing robust immunogenicity after dose 1 of mRNA-1273 in unvaccinated baseline SARS-CoV-2 positive adolescent participants, prompted the evaluation of the immunogenicity of 2 doses of the

bivalent mRNA-1273.222 vaccine given 6 months apart. The 6-month interval will allow evaluation of the immunogenicity of Dose 1.

Taken together, these data strongly suggest that a monovalent 50 µg mRNA-1273 given as a 2 dose primary series, as a homologous and heterologous booster vaccine is anticipated to provide strong immunogenicity in the adolescent population. Similarly, these data also suggest that a bivalent 50 µg mRNA-1273.222, given as 1 dose or 2 doses, may provide strong immunogenicity in the adolescent population.

3.4. End-of-Study Definition

The EOS for the full study is defined as completion of the last visit of the last participant in the study or the last scheduled procedure, as presented in the SoAs (Table 10 [if remained blinded or declined to receive a BD in Part A], Table 13, Table 14, Table 15, and Table 16).

4. STUDY POPULATION

Participants will be enrolled at approximately 35 to 64 study sites in the USA or out of USA countries.

Prospective approval of protocol deviations to recruitment and enrollment criteria, also known as protocol waivers or exemptions, is not permitted.

4.1. Eligibility Criteria (Part 1A, Part 2, and Part 3)

4.1.1. Inclusion Criteria

Each participant must meet all of the following criteria at the Screening Visit (Day 0) or at Day 1, unless noted otherwise, to be enrolled in this study:

1. Male or female, 12 to < 18 years of age at the time of consent (Screening Visit, Day 0) who, in the opinion of the investigator, is in good general health based on review of medical history and Screening physical examination.
2. Investigator assessment that the participant, in the case of an emancipated minor, or parent(s)/LAR(s) understand and are willing and physically able to comply with protocol-mandated follow-up, including all procedures and provides written informed consent/assent.
3. BMI at or above the third percentile according to WHO Child Growth Standards at the Screening Visit (Day 0); see Section 10.2.18.
4. Female participants of nonchildbearing potential may be enrolled in the study. Nonchildbearing potential is defined as premenarche or surgically sterile (history of bilateral tubal ligation, bilateral oophorectomy, hysterectomy).
5. Female participants of childbearing potential may be enrolled in the study if the participant fulfills all the following criteria:
    - Has a negative pregnancy test at Screening (Day 0), on the day of the first injection (Day 1), and on the day of the second injection (Day 29 in Part 1A and Part 2, and Day 181 in Part 3).
    - Has practiced adequate contraception or has abstained from all activities that could result in pregnancy for at least 28 days prior to the first injection (Day 1).
6. Has agreed to continue adequate contraception or abstinence through 3 months following the second injection (Day 29 in Part 1 A and Part 2, and Day 181 in Part 3).

Adequate female contraception is defined as consistent and correct use of approved contraceptive method in accordance with the product label (Section 10.3).

4.1.2. Exclusion Criteria

Participants who meet any of the following criteria at the Screening Visit (Day 0) or at Day 1, unless noted otherwise, will be excluded from the study:

1. Has a known history of SARS-CoV-2 infection within 2 weeks prior to administration of IP or known close contact with anyone with laboratory-confirmed SARS-CoV-2 infection

or COVID-19 within 2 weeks prior to administration of IP (Part 2 participants only). For Part 3 participants, known history of SARS-CoV-2 infection within 90 days prior to administration of IP or known close contact with anyone with laboratory-confirmed SARS-CoV-2 infection or COVID-19 within 90 days prior to administration of IP.

2. Travel outside of the USA or home country (Part 2 and Part 3 only) in the 28 days prior to the Screening Visit (Day 0).
3. Pregnant or breastfeeding.
4. Is acutely ill or febrile 24 hours prior to or at the Screening Visit (Day 0). Fever is defined as a body temperature ≥ 38.0°C/≥ 100.4°F. Participants who meet this criterion may have visits rescheduled within the relevant study visit windows. Afebrile participants with minor illnesses can be enrolled at the discretion of the investigator.
5. Prior administration of an investigational CoV (eg, SARS-CoV-2, SARS-CoV, MERS-CoV) vaccine.
6. Current treatment with investigational agents for prophylaxis against COVID-19.
7. Has a medical, psychiatric, or occupational condition that may pose additional risk as a result of participation, or that could interfere with safety assessments or interpretation of results according to the investigator's judgment.
8. Current use of any inhaled substance (eg, tobacco or cannabis smoke, nicotine vapors).
9. History of chronic smoking (≥ 1 cigarette a day) within 1 year of the Screening Visit (Day 0).
10. History of illegal substance use or alcohol abuse within the past 2 years. This exclusion does not apply to historical cannabis use that was formerly illegal in the participant's state but is legal at the time of Screening.
11. History of a diagnosis or condition that, in the judgment of the investigator, may affect study endpoint assessment or compromise participant safety, specifically:
    - Congenital or acquired immunodeficiency, including HIV infection
    - Suspected active hepatitis
    - Has a bleeding disorder that is considered a contraindication to IM injection or phlebotomy
    - Dermatologic conditions that could affect local solicited AR assessments
    - History of anaphylaxis, urticaria, or other significant AR requiring medical intervention after receipt of a vaccine
    - Diagnosis of malignancy within the previous 10 years (excluding nonmelanoma skin cancer)
    - Febrile seizures

12. Receipt of:
    - Any licensed vaccine within 28 days before the first dose of IP or plans for receipt of any licensed vaccine within 28 days before and/or after each dose of IP.
    - Systemic immunosuppressants or immune-modifying drugs for > 14 days in total within 6 months prior to the day of enrollment (for corticosteroids, ≥ 20 mg/day prednisone equivalent). Topical tacrolimus is allowed if not used within 14 days prior to the day of enrollment. Participants may have visits rescheduled for enrollment if they no longer meet this criterion within the Screening Visit window. Inhaled, nasal, and topical steroids are allowed.
    - Intravenous blood products (red cells, platelets, immunoglobulins) within 3 months prior to enrollment.
13. Has donated ≥450 mL of blood products within 28 days prior to the Screening Visit (Day 0) or plans to donate blood products during the study.
14. Participated in an interventional clinical study within 28 days prior to the Screening Visit (Day 0) or plans to do so while participating in this study.
15. Is an immediate family member or has a household contact who is an employee of the research center or otherwise involved with the conduct of the study.

4.2. Study Eligibility Criteria (Part 1B)

1. Participants must have been previously enrolled in the mRNA-1273-P203 study.
2. Female participants of childbearing potential may be enrolled in the study if the participant has a negative pregnancy test on the day of the first injection (OL-Day 1) and on the day of the second injection (OL-Day 29).

4.3. Study Eligibility Criteria (Part 1C-1 – Homologous Booster Phase)

Inclusion Criteria:

1. Participants must have been previously enrolled in the mRNA-1273-P203 study, are actively participating in Part 1A or Part 1B and are least 5 months from the last dose.
2. Female participants of childbearing potential may be enrolled in the study if the participant has a negative pregnancy test on the day of the first injection (BD-Day 1).

Exclusion Criteria:

1. Pregnant or breastfeeding.
2. Is acutely ill or febrile 24 hours prior to or at the Screening Visit (Day 0). Fever is defined as a body temperature ≥38.0°C/≥100.4°F. Participants who meet this criterion may have visits rescheduled within the relevant study visit windows. Afebrile participants with minor illnesses can be enrolled at the discretion of the investigator.
3. Has a medical, psychiatric, or occupational condition that may pose additional risk as a result of participation, or that could interfere with safety assessments or interpretation of results according to the investigator’s judgment.

4. History of a diagnosis or condition (after enrollment in Part 1A) that, in the judgment of the investigator, may affect study endpoint assessment or compromise participant safety:
    - Suspected active hepatitis
    - Has a bleeding disorder that is considered a contraindication to IM injection or phlebotomy
    - Dermatologic conditions that could affect local solicited AR assessments
    - History of anaphylaxis, urticaria, or other significant AR requiring medical intervention after receipt of a vaccine
    - Diagnosis of malignancy (excluding nonmelanoma skin cancer)
5. Receipt of:
    - Any authorized or licensed vaccine within 28 days before the first dose of IP or plans for receipt of any licensed vaccine through 28 days following the last dose of IP (authorized/approved bivalent vaccine against SARS-CoV-2 as 4th BD is not exclusionary); OR
    - Any seasonal influenza vaccine within 14 days before the first dose of IP or plans for receipt of any seasonal influenza vaccine 14 days following the last dose of IP.
6. Participated in an interventional clinical study, other than mRNA-1273-P203, within 28 days prior to the Screening Visit (Day 0) or plans to do so while participating in this study.

4.4. Study Eligibility Criteria (Part 1C-2 – Heterologous Booster Dose):

Inclusion Criteria:

1. Male or female, 12 to < 18 years of age at the time of consent who, in the opinion of the investigator, is in good general health based on review of medical history and Screening physical examination AND has completed non-Moderna primary COVID-19 vaccination series under EUA (ie, Pfizer) at least 3 months from consent.
2. Investigator assessment that the participant, in the case of an emancipated minor, or parent(s)/LAR understand and are willing and physically able to comply with protocol-mandated follow-up, including all procedures and provides written informed consent/assent.
3. BMI at or above the third percentile according to WHO Child Growth Standards at the Screening Visit (BD-Day 0).
4. Female participants of nonchildbearing potential may be enrolled in the study. Nonchildbearing potential is defined as premenarche or surgically sterile (history of bilateral tubal ligation, bilateral oophorectomy, hysterectomy). Female participants of childbearing potential may be enrolled in the study if the participant has a negative pregnancy test on the day of the first injection (BD-Day 1) and has agreed to continue adequate contraception or abstinence through 3 months following BD (BD-Day 1).

Exclusion Criteria:

1. Has a known history of SARS-CoV-2 infection within 2 weeks prior to administration of IP or known close contact with anyone with laboratory-confirmed SARS-CoV-2 infection or COVID-19 within 2 weeks prior to administration of IP.
2. Pregnant or breastfeeding.
3. Is acutely ill or febrile 24 hours prior to or at the Screening Visit (BD-Day 0). Fever is defined as a body temperature ≥38.0°C/≥100.4°F. Participants who meet this criterion may have visits rescheduled within the relevant study visit windows. Afebrile participants with minor illnesses can be enrolled at the discretion of the investigator.
4. Has a medical, psychiatric, or occupational condition that may pose additional risk as a result of participation, or that could interfere with safety assessments or interpretation of results according to the investigator’s judgment.
5. History of a diagnosis or condition that, in the judgment of the investigator, may affect study endpoint assessment or compromise participant safety:
   - Suspected active hepatitis
   - Has a bleeding disorder that is considered a contraindication to IM injection or phlebotomy
   - Dermatologic conditions that could affect local solicited AR assessments
   - History of anaphylaxis, urticaria, or other significant AR requiring medical intervention after receipt of a vaccine
   - Diagnosis of malignancy (excluding nonmelanoma skin cancer)
6. Receipt of:
   - Any authorized or licensed vaccine within 28 days before the first dose of IP (monovalent COVID-19 primary series vaccines are not exclusionary) or plans for receipt of any licensed vaccine through 28 days following the last dose of IP (monovalent COVID-19 primary series vaccines as well as authorized/approved bivalent vaccine against SARS-CoV-2 as 4th booster are not exclusionary) or any seasonal influenza vaccine within 14 days before the first dose of IP or plans for receipt of any seasonal influenza vaccine 14 days following the last dose of IP.
7. Participated in an interventional clinical study, other than mRNA-1273-P203 study, within 28 days prior to the Screening Visit (BD-Day 0) or plans to do so while participating in this study.

4.5. Lifestyle Restrictions

Participants must not eat or drink anything hot or cold within 10 minutes before oral temperature is taken.

4.6. Screen Failures (Part 1A: Blinded Phase, Part 2, and Part 3)

Screen failures are defined as participants who consent to participate in the clinical study but are not subsequently randomly assigned to treatment. A minimum set of screen failure information is required to ensure transparent reporting of screen failures to meet the CONSORT publishing requirements and to respond to queries from regulatory authorities. Minimum information includes date of informed consent, demography, reason(s) for screen failure, eligibility criteria, and information on any SAE that may have occurred from Day 1 to the time of withdrawal.

5. STUDY TREATMENT

5.1. Investigational Product Administered

The term IP refers to mRNA-1273 (100 µg for Part 1A and Part 1B, and 50 µg for Part 1C, and Part 2), and 50 µg mRNA-1273.222 (Part 3) vaccine or placebo (0.9% sodium chloride) in this study.

The mRNA-1273 is an LNP dispersion of an mRNA encoding the prefusion stabilized S protein of SARS-CoV-2 formulated in LNPs composed of 4 lipids (1 proprietary and 3 commercially available): the proprietary ionizable lipid SM-102; cholesterol; 1,2-distearoyl-sn-glycero-3-phosphocholine (DSPC); and 1-monomethoxypolyethyleneglycol-2,3-dimyristylglycerol with polyethylene glycol of average molecular weight 2000 (1-monomethoxypolyethyleneglycol-2,3-dimyristylglycerol polyethylene glycol 2000 [PEG2000-DMG]).

mRNA-1273 injection is provided as a sterile liquid for injection, white to off-white dispersion in appearance, at a concentration of 0.5 mg/mL in 20 mM Tris buffer containing 87 mg/mL sucrose and 10.7 mM sodium acetate at pH 7.5.

The Sponsor initiated the development of modified COVID-19 vaccine leveraging the mRNA/LNP technology platform against SARS-CoV-2 variant, which incorporates key mutations present in Omicron subvariants BA.4/BA.5. The vaccine IP, mRNA-1273.222, contains 2 mRNAs: CX-024414 encoding for the S-2P of Wuhan-Hu-1 and CX-034476 encoding for the S-2P of Omicron subvariants BA.4/BA.5.

mRNA-1273.222 will be provided as a sterile solution for injection at a concentration of 0.1 mg/mL in 20 mM Tris buffer with sucrose, at pH 7.5. The solution will be kept in 2R USP Type I borosilicate glass vials with PLASCAP vial seal containing a 13 mm FluroTec-coated plug stopper with a 0.8 mL nominal fill volume. For Part 3, each injection preparation will have a volume of 0.5 mL and contain 50 µg mRNA-1273.222.

5.2. Randomization (Part 1A Only)

Random assignment of participants will use a centralized interactive response technology, in accordance with pregenerated randomization schedules.

5.3. Dosing and Management of mRNA-1273 Vaccine

5.3.1. Preparation of Study Vaccine for Injection

Each dose of IP will be prepared for each participant based on the assigned treatment, as detailed in the mRNA-1273-P203 Pharmacy Manual. For Part 1A and Part 1B, the volume of IP injected will be 0.5 mL consisting of either 100 µg dose of mRNA-1273 or placebo (normal saline), as detailed in the mRNA-1273-P203 Pharmacy Manual. For Part 1C and Part 2, each injection will have a volume of 0.25 mL and contain mRNA-1273 50 µg.

For Part 3, each dose of IP will be prepared for each participant as detailed in the mRNA-1273-P203 Pharmacy Manual. The volume of IP injected will be 0.5 mL and contain 50 µg mRNA-1273.222.

5.3.2. Administration of Study Vaccine

In the Blinded Phase, Part 1A of the study, each participant will receive 2 doses of IP by IM injection, 28 days apart (ie, Day 1 and Day 29) into the deltoid muscle, according to their assigned regimen and according to the procedures specified in the mRNA-1273-P203 Pharmacy Manual. Preferably, both doses should be administered into the nondominant arm.

In the open-label Part 1B of the study, mRNA-1273 vaccine will be administered as an IM injection into the deltoid muscle following the injection schedule for each group based on the product received in Part 1A. Participants who received placebo in Part 1A will receive 2 doses of mRNA-1273 (100 µg) on OL – Day 1 and OL-Day 29 of Part 1B (Table 12). Preferably, both doses should be administered into the nondominant arm.

For Part 1C, mRNA-1273 vaccine will be administered as an IM injection into the deltoid muscle. Preferably, the dose should be administered into the nondominant arm. Each injection will have a volume of 0.25 mL and contain mRNA-1273 50 µg.

For Part 2, each participant will receive 2 doses of 50 µg mRNA-1273 by IM injection, 28 days apart (ie, Day 1, Day 29) into the deltoid muscle, according to the procedures specified in the mRNA-1273-P203 Pharmacy Manual. Preferably, all doses should be administered into the nondominant arm.

For Part 3, each participant will receive 2 doses of 50 µg mRNA-1273.222 by IM injection, 6 months apart (ie, Day 1, Day 181) into the deltoid muscle, according to the procedures specified in the mRNA-1273-P203 Pharmacy Manual. Preferably, all doses should be administered into the nondominant arm.

At each visit when IP is administered, participants will be monitored for a minimum of 30 minutes after administration. Assessments will include vital sign measurements and monitoring for local or systemic reactions (Table 10, Table 12, Table 13, Table 14, and Table 15).

Eligibility for a subsequent dose of IP will be determined by following the criteria outlined in Section 6.

The study sites will be appropriately staffed with individuals with basic cardiopulmonary resuscitation training/certification. Either on-site resuscitation equipment and personnel or appropriate protocols for the rapid transport of participant to a resuscitation area or facility are required.

5.3.3. Study Vaccine Delivery and Receipt

The Sponsor or designee is responsible for the following:

- Supplying the IP
- Confirming the appropriate labeling of the IP, so that it complies with the legal requirements of the USA

The investigator is responsible for acknowledging the receipt of the IP by a designated staff member at the study site, including the following:

- Confirming that the IP was received in good condition

- Confirming that the temperature during shipment from the Sponsor to the investigator's designated storage location was appropriate
- Confirming that the Sponsor has authorized the IP for use
- Ensuring the appropriate dose level of IP is properly prepared using aseptic technique

Further description of the IP and instructions for the receipt, storage, preparation, administration, accountability, and destruction of the IP are described in the mRNA-1273-P203 Pharmacy Manual.

5.3.4. Study Vaccine Packaging and Labeling

The Sponsor will provide the investigator (via the study site pharmacy) with adequate quantities of IP. The sterile IP is packaged in 10R glass vials with a 5.0-mL fill volume (Part 1A, Part 1B, and Part 1C), a 10R glass vial with an 8.0 mL fill volume (Part 1C and Part 2 only), or a 10R glass vial with a 6.3 mL fill volume (Part 1C and Part 2 only). The IP will have all required labeling per regulations and will be supplied to the pharmacy in an unblinded manner. For Part 3, the IP will be packaged in a 2R glass vial.

The IP will be packaged and labeled in accordance with the standard operating procedures of the Sponsor or of its designee, CFR Title 21, ICH GCP guidelines, guidelines for Quality System Regulations, and applicable regulations.

5.3.5. Study Vaccine Storage

The IP must be stored as per the temperature conditions printed on the IP label in a secure area with limited access and protected from moisture and light until it is prepared for administration (Section 5.3.1). The refrigerator and/or freezer utilized for IP storage should have automated temperature recording and a 24-hour alert system in place that allows for rapid response in case of refrigerator malfunction. There must be an available backup refrigerator and freezer. The refrigerators and freezers must be connected to a backup generator(s). In addition, IP accountability study staff are required to keep a temperature log to establish a record of compliance with these storage conditions. The study site is responsible for reporting any IP that was not temperature controlled during shipment or during storage. Such IP will be retained for inspection by the monitor and disposed of according to approved methods.

5.3.6. Study Vaccine Accountability

It is the investigator's responsibility that the IP accountability study staff maintain accurate records in an IP accountability log of receipt of all IP, study site IP inventory, IP dispensing, IP injections, and return to the Sponsor or alternative disposition of used and unused IP vials.

A study site monitor will review the inventory and accountability log during study site visits and at the completion of the study. Additional details are found in the mRNA-1273-P203 Pharmacy Manual.

5.3.7. Study Vaccine Handling and Disposal

A study site monitor will reconcile the IP inventory during the conduct and at the end of the study for compliance. Once fully reconciled at the study site at the end of the study, the IP can be destroyed at the investigational site or at a Sponsor-selected third party, as appropriate.

Vaccine may be destroyed at the study site only if permitted by local regulations and authorized by the Sponsor. A certificate of destruction must be completed and sent to the Sponsor or designee.

5.4. Study Treatment Compliance

All doses of IP will be administered at the study site under direct observation of medically qualified study staff and appropriately recorded (date and time) in the eCRF. Qualified study site staff will confirm that the participant has received the entire dose of IP. If a participant does not receive IP or does not receive all of the planned dose, the reason for the missed dose will be recorded. Data will be reconciled with study site accountability records to assess compliance.

Participants who miss the second or third dose due to noncompliance with the visit schedule and not due to a safety pause will still be required to follow the original visit and testing schedule as described in the protocol and their regimen schedule. Unless consent is withdrawn, a participant who withdraws or is withheld from receiving the second dose will remain in the study and complete all safety and immunogenicity assessments required through the participant's last scheduled study visit.

The study site staff are responsible for ensuring that participants comply with the allowed study visit windows. If a participant misses a visit, every effort should be made to contact the participant and complete a visit within the defined visit window (Part 1A, Table 10; Part 1B, Table 12; Part 1C, Table 13 and Table 14; and Part 2, Table 15, and Part 3, (Table 16). If a participant does not complete a visit within the time window, that visit will be classified as a missed visit and the participant will continue with subsequent scheduled study visits. All safety requirements of the missed visit will be captured and included in the subsequent visit.

5.5. Prior and Concomitant Medications

5.5.1. Prior Medications and Therapies

Information about prior medications (including any prescription or over-the-counter medications, vaccines, or blood products) taken by the participant within the 28 days before providing informed consent/assent (or as designated in the inclusion/exclusion requirements) will be recorded in the participant's eCRF.

5.5.2. Concomitant Medications and Therapies

At each study visit, study site staff must question the participant and/or the participants' parent(s)/LAR(s) regarding any medications taken and vaccinations received by the participant and record the following information in the eCRF:

- All nonstudy vaccinations administered within the period starting 28 days before the first dose of IP.

- All concomitant medications and nonstudy vaccinations taken through 28 days after each dose of IP. Antipyretics and analgesics taken prophylactically (ie, taken in the absence of any symptoms in anticipation of an injection reaction) will be recorded as such.
- Any concomitant medications relevant to or for the treatment of an SAE or a MAAE.
- Participants will be asked in the eDiary if they have taken any antipyretic or analgesic to treat or prevent fever or pain within 7 days after each dose of IP, including the day of injection. Reported antipyretic or analgesic medications should be recorded in the source document by the study site staff during the postinjection study visits or via other participant interactions (eg, telephone calls).

5.5.3. Recording of Concomitant Medications and Concomitant Vaccinations

Study site staff must question the participant regarding any medications taken and vaccinations received by the participant and record the following information in the eCRF:

- All nonstudy vaccinations administered within the period starting 28 days before the first dose of IP.
- Seasonal influenza vaccine administered for the current influenza season (typically October through April in the Northern Hemisphere).
- All concomitant medications and nonstudy vaccinations taken through 28 days after each dose of IP. Antipyretics and analgesics taken prophylactically (ie, taken in the absence of any symptoms in anticipation of an injection reaction) will be recorded as such.
- Any concomitant medications used to prevent or treat COVID-19 or its symptoms.
- Any concomitant medications relevant to or for the treatment of an SAE or a MAAE.
- Participants will be asked in the eDiary if they have taken any antipyretic or analgesic to treat or prevent fever or pain within 7 days after each IP dose, including on the day of dosing. Reported antipyretic or analgesic medications should be recorded in the source document by the study site staff during the postinjection study visits or via other participant interactions (eg, phone calls).

Concomitant medications (including vaccinations) will be coded using the WHO Drug Dictionary. If a participant takes a prohibited drug therapy, the investigator and the CRO’s medical monitor will make a joint decision about continuing or withholding further injection of the participant based on the time the medication was administered, the drug’s pharmacology and pharmacokinetics, and whether use of the medication will compromise the participant’s safety or interpretation of the data. It is the investigator’s responsibility to ensure that details regarding the concomitant medications are adequately recorded in the eCRF.

5.5.4. Concomitant Medications and Vaccines that May Lead to the Elimination of a Participant from Per-Protocol Analyses

The use of the following concomitant medications and/or vaccines will not require withdrawal of the participant from the study (with the exception of planned or actual receipt of a nonstudy COVID-19 vaccine either under EUA or licensed for Part 1A and 1B only) but may determine a

participant's eligibility to receive a second dose or evaluability in the PP analysis (analysis sets are described in Section 8.4:

- Any investigational or nonregistered product (drug or vaccine) other than the IP used during the study period.
- Immunosuppressants or other immune-modifying drugs administered chronically (ie, more than 14 days in total) during the study period. For corticosteroids, this will mean that prednisone ≥20 mg/day or the equivalent is not permitted. Inhaled, nasal, and topical steroids are allowed.
- Long-acting immune-modifying drugs administered at any time during the study period (eg, infliximab).
- Immunoglobulins and/or any blood products administered during the study period.

5.6. Intervention After the End of the Study

Any SAE occurring after the end of the study and considered to be caused by the IP must be reported to the Sponsor.

6. DELAYING OR DISCONTINUING STUDY TREATMENT AND PARTICIPANT WITHDRAWAL FROM THE STUDY

6.1. Criteria for Delay of Vaccine Administration

6.1.1. Individual Participant Criteria for Delay of Study Vaccination

Body temperature (oral) must be measured on dosing visits before vaccine administration. The following events constitute criteria for delay of injection, and if either of these events occur at the time scheduled for dosing, the participant may receive the study injection at a later date within the time window specified in the relevant SoA (Table 10, Table 12, Table 13, Table 14, Table 15, Table 16), or the participant may be discontinued from dosing at the discretion of the investigator (Section 6.2):

- Acute moderate or severe infection with or without fever at the time of dosing
- Fever, defined as body temperature ≥38.0°C/≥100.4°F at the time of dosing

Participants with a minor illness without fever, as assessed by the investigator, can be vaccinated. Participants with a fever of ≥38.0°C/≥100.4°F will be contacted within the time window acceptable for participation and re-evaluated for eligibility. If the investigator determines that the participant's health on the day of dosing temporarily precludes injection, the visit should be rescheduled within the allowed interval for that visit if possible or at a time the participant is clinically stable according to the judgment of the investigator.

If a participant takes a prohibited drug therapy, an injection could be delayed within the visit window based on the joint decision of the investigator and the CRO's medical monitor (Section 5.5.3).

6.2. Discontinuing Study Vaccination

Participants can discontinue study injection (ie, refuse the second dose) for any reason, without prejudice to further treatment the participant may need to receive.

The investigator, in consultation with the Sponsor's medical monitor, may withhold a participant from further injection if the participant experiences any of the following:

- Becomes pregnant
- Withdrawal of consent (not related to COVID-19)
- Develops, during the course of the study, symptoms or conditions listed in the exclusion criteria (Section 4.1.2)
- Experiences an AE (other than reactogenicity) after injection that is considered by the investigator to be related to IP (Section 7.5.9) and is of Grade 3 (severe) or greater severity
- Experiences an AE or SAE that, in the judgment of the investigator, requires IP withdrawal due to its nature, severity, or required treatment, regardless of the causal relationship to vaccine
- Experiences an AESI

- Experiences a clinically significant change in vital sign measurements, or general condition that, in the judgment of the investigator, requires vaccine withdrawal
- Experiences anaphylaxis clearly related to IP
- Experiences generalized urticaria related to IP

The reason(s) for withdrawal from further injection will be recorded in the eCRF.

If a participant takes a prohibited drug therapy, the investigator could withhold the second or third dose based on a joint decision of the investigator and the CRO's medical monitor (Section 5.5.3).

Every reasonable attempt will be made to follow-up with participants for safety throughout the entire scheduled study period according to their regimen, even if the participant does not receive the second dose or misses one or more visits. Unless participants withdraw consent, they are expected to remain in the study and complete all scheduled visits and assessments.

6.3. Participant Discontinuation/Withdrawal from the Study

Participants who withdraw or are withdrawn from the study will not be replaced. A "withdrawal" from the study refers to a situation wherein a participant does not return for the final visit planned in the protocol. The statistical management of participant withdrawals is discussed in Section 8.

Participants can withdraw consent and withdraw from the study at any time, for any reason, without prejudice to further treatment the participant may need to receive. The investigator will request that the participant complete all study procedures pending at the time of withdrawal.

If participant desires to withdraw from the study because of an AE, the investigator will try to obtain agreement to follow-up with the participant until the event is considered resolved or stable and will then complete the EOS eCRF.

Information related to the withdrawal will be documented in the eCRF. The investigator will document whether the decision to withdraw a participant from the study was made by the participant or by the investigator, as well as which of the following possible reasons was responsible for withdrawal:

- AE (specify)
- SAE (specify)
- Death
- Lost to follow-up
- Physician decision (specify)
- Pregnancy
- Protocol deviation
- Study terminated by Sponsor
- Withdrawal of consent by participant (specify)

- Other (specify)

Participants who are withdrawn from the study because of AEs (including SAEs) must be clearly distinguished from participants who are withdrawn for other reasons. Investigators will follow-up with participants who are withdrawn from the study as result of an SAE or AE until resolution of the event.

A participant who withdraws from the study may request destruction of any samples taken and not tested, and the investigator must document this in the study site study records.

If the participant withdraws consent for disclosure of future information, the Sponsor may retain and continue to use any data collected before such a withdrawal of consent (Section 10.2.10).

The Sponsor will continue to retain and use all research data that have already been collected for the study evaluation, unless the participant has requested destruction of these samples. All biological samples that have already been collected may be retained and analyzed at a later date (or as permitted by local regulations).

6.4. Study Pause Rules

During Part 1A of the study, the investigators, study medical monitor, and Sponsor will monitor for events that could trigger a study pause. Study pause rule criteria, events, and thresholds are described in Table 5. Although these pause rules are not applicable during Part 1B or Part 1C, Part 2, or Part 3, participants will continue to be monitored for the events in Table 5, and the Sponsor will be notified if any of these events occur.

Table 5: Pause Rule Criteria, Events, and Thresholds

Pause Rule Criterion	Event	Participant Threshold for Triggering Study Pause
1	Any death due to SARS-CoV-2 infection	≥ 1
2	Any related SAE or related Grade 4 AE	≥ 1
3	Hospitalization due to SARS-CoV-2 infection	≥ 1
4[a]	Any Grade 3 or higher solicited local AR lasting ≥ 24 hours and occurring within 7-days of injection (Days 1-8)	≥30 participants out of the first 300 participants enrolled
5[a]	Any Grade 3 or higher solicited systemic AR lasting ≥ 24 hours and occurring within 7-days of injection (Days 1-8)	≥30 participants out of the first 300 participants enrolled
6[a]	Any ≥ Grade 3 or higher unsolicited AR that cannot be reasonably attributed to a cause other than vaccination	≥30 participants out of the first 300 participants enrolled

Abbreviations: AE = adverse event; AR = adverse reaction; ICU = intensive care unit; SAE = serious adverse event; SARS-CoV-2 = Severe Acute Respiratory Syndrome coronavirus 2.

a. Pause Rules 4, 5, and 6 apply only to the first 300 participants enrolled.

If any of the thresholds for a study pause is met during Part 1A, the Sponsor will immediately suspend further enrollment, pause study dosing, and notify all investigators. Such a suspension will remain in force until the threshold event(s) is (are) reviewed by the DSMB and a recommendation to continue is provided to the Sponsor.

The investigator or designee is responsible for reporting to the Sponsor, via the EDC system within 24 hours of observation, each event that potentially meets any pause rule criterion. The Sponsor will inform the DSMB of any event that potentially meets any pause rule criterion. The DSMB will review all available study data to adjudicate such events in accordance with the DSMB charter.

The Sponsor will notify the CBER within 48 hours in the event of a study pause. In the event of a study pause, all safety and immunogenicity assessments will continue PP. The window allowance for injection visits may be extended by an additional 7 days (ie, +14 days) for affected participants at the discretion of the Sponsor.

During Part 1B, Part 1C, Part 2, and Part 3 of the study, the Sponsor will continue to inform the DSMB of the occurrence of any of the events in Table 5. The DSMB will review all available relevant study data to adjudicate such events in accordance with the DSMB charter.

6.5. Lost to Follow-up

A participant will be considered LTFU if he or she repeatedly fails to return for scheduled visits without stating an intention to withdraw consent and is unable to be contacted by the study site. The following actions must be taken if a participant fails to return to the clinic for a required study visit:

- The study site staff must attempt to contact the participant and reschedule the missed visit as soon as possible, counsel the participant on the importance of maintaining the assigned visit schedule and ascertain whether the participant wishes to and/or should continue in the study.
- Before a participant is deemed LTFU, the investigator or designee must make every effort to regain contact with the participant (where possible, 3 telephone calls and, if necessary, a certified letter to the participant’s last known mailing address or local equivalent methods). These contact attempts (eg, dates of telephone calls and registered letters) should be documented in the participant’s medical record.
- A participant who continues to be unreachable or continues to be noncompliant with study visits or procedures will be considered to have withdrawn from the study.
- A participant should not be considered LTFU until due diligence has been completed.

7. STUDY ASSESSMENTS AND PROCEDURES

Before performing any study procedures, all potential participants and/or participants' parent/LAR will sign an ICF (as detailed in Section 10.2.6). Participants will undergo study procedures at the timepoints specified in the Part 1A SoA (Table 10), Part 1B SoA (Table 12), Part 1C (Table 13 and Table 14), Part 2 (Table 15), or Part 3 (Table 16).

After the participant proceeds to the Participant Decision Clinic Visit (Part 1A) of the study, participants who received mRNA-1273 in Part 1A will continue to follow the open-label Part 1A SoA (Table 10). Participants who received placebo in Part 1A will transition to the open-label Part 1B of the study (Figure 8) and will follow the Part 1B SoA (Table 12). Participants in Part 1A or Part 1B who transitioned to Part 1C-1, at least 5 months from the last dose, will follow open-label Part 1C-1 SoA (Table 13) until study completion. Participants who remained blinded will follow Part 1A SoA. Participants who do not receive a BD will continue with their SoA (Part 1A or Part 1B). Eligible participants enrolled in Part 1C-2 will follow Part 1C-2 SoA (Table 14). Eligible participants enrolled in Part 2 will follow Part 2 SoA (Table 15). Eligible participants enrolled in Part 3 will follow Part 3 SoA (Table 16).

A participant can also be seen for an unscheduled visit at any time during the study. An unscheduled visit may be prompted by reactogenicity issues, illness visit criteria for COVID-19, or new or ongoing AEs. The study site also has the discretion to make reminder telephone calls or send text messages to inform the participant about visits, review eDiary requirements, or follow-up on ongoing or outstanding issues.

In accordance with "FDA Guidance on Conduct of Clinical Trials of Medical Products during COVID-19 Public Health Emergency" (DHHS 2020), investigators may convert study site visits to home visits or telemedicine visits with the approval of the Sponsor. Such action should be taken to protect the safety and well-being of study participants and study site staff or to comply with state or municipal mandates.

General considerations for study assessments and procedures include the following:

- Protocol waivers or exemptions are not allowed. The study procedures and their timing must be followed as presented in Table 10, Table 12, Table 13, Table 14, Table 15, and Table 16. Adherence to the study design requirements is essential and required for study conduct.
- Immediate safety concerns should be discussed with the Sponsor immediately upon occurrence or awareness to determine if the participant should continue study treatment or participation in the study.
- All screening evaluations must be completed and reviewed to confirm that potential participants meet all eligibility criteria. The investigator will maintain a screening log to record details of all participants screened and to confirm eligibility or record reasons for screening failure, as applicable.
- Procedures conducted as part of the participant's routine clinical management (eg, blood count) and obtained before signing of the ICF may be utilized for screening or baseline assessments provided the procedures met the protocol-specified criteria and were performed within the time frame defined in the SoA.

7.1. Safety Assessments and Procedures

In Part 1A (blinded), Part 1B (open-label), Part 1C (open-label), Part 2 (open-label), and Part 3 (open-label) safety assessments will include monitoring and recording of the following for each participant, according to the SoA (Table 10, Table 12, Table 13, Table 14, Table 15, and Table 16):

- Solicited local and systemic ARs (Section 7.5.3) that occur during the 7 days following each injection (ie, the day of injection and 6 subsequent days) in Part 1A, Part 1C, Part 2, and Part 3. Solicited ARs will be recorded daily using eDiaries (Section 7.1.1).
- Unsolicited AEs observed or reported during the 28 days following each injection (ie, the day of injection and 27 subsequent days) in Part 1A, Part 1C, Part 2, and Part 3. Unsolicited AEs are defined in Section 7.5.1.
- AEs leading to discontinuation from dosing and/or withdrawal from study participation from Day 1 through the last day of study participation (ie, Day 394 per Part 1A SoA, OL-Day 178 in Part 1B, BD-Day 361 in Part 1C, Day 394 in Part 2, and Day 361 in Part 3).
- MAAEs from first dose on Day 1 through the entire study period (ie, Day 394 per Part 1A SoA, OL-Day 178 in Part 1B, BD-Day 361 in Part 1C, Day 394 in Part 2, and Day 361 in Part 3).
- SAEs from first dose on Day 1 through the entire study period (ie, Day 394 per Part 1A SoA, OL-Day 178 in Part 1B, BD-Day 361 in Part 1C, Day 394 in Part 2, and Day 361 in Part 3).
- AESIs through the entire study period (ie, Day 394 per Part 1A SoA, OL-Day 178 in Part 1B, BD-Day 361 in Part 1C, Day 394 in Part 2, and Day 361 in Part 3).
- Vital sign measurements (Section 7.1.4).
- Physical examination findings (Section 7.1.5).
- Assessments for SARS-CoV-2 infection from Day 1 through study completion (Section 7.1.6).
- Details of all pregnancies in female participants will be collected after the start of study treatment and until the end of their participation in the study (Section 7.5.6).

7.1.1. Use of Electronic Diaries

At the time of consent/assent, participants or their caregivers must confirm they will be willing to complete an eDiary using either an application downloaded to their smartphone or using a device that is provided at the time of enrollment. Before enrollment on Day 1, participants or their caregivers will be instructed to download the eDiary application or will be provided an eDiary device to record solicited ARs (Section 7.5.3) on Day 1. Participants who were originally randomized to placebo and who opt to receive mRNA-1273 will enter the Open-label Observational Phase. They will receive Dose 1 at Day 209 (OL-Day 1), return for their second dose at OL-Day 29, and return again at OL-Day 57. Participants who entered the open-label phase, may opt to receive a BD in Part 1C-1, and will follow Part 1C-1 SoA until study

completion. During the open-label period Part 1B, the eDiary will not be used. During the open-label Part 1C-1, Part 1C-2, Part 2, and Part 3, the eDiary will be used to record solicited ARs.

In Part 1A, the Blinded Phase, Part 2, and Part 3 at each injection visit, participants or their caregivers will be instructed (Day 1) or reminded (Day 29 for Part 1A and Part 2, and Day 181 for Part 3) on thermometer usage to measure body temperature, ruler usage to measure injection site erythema and swelling/induration (hardness), and self-assessment for localized axillary swelling or tenderness on the same side as the injection arm.

In Part 1A, the Blinded Phase, Part 2, and Part 3 at each injection visit, participants or their caregivers will record data into the eDiary starting approximately 30 minutes after injection under supervision of the study site staff to ensure successful entry of assessments. The study site staff will perform any retraining as necessary. Participants or their caregivers will continue to record data in the eDiary after they leave the study site, preferably in the evening and at the same time each day, on the day of injection and for 6 days following injection.

Participants or their caregivers will record the following data in the eDiary:

- Solicited local and systemic reactogenicity ARs, as defined in Section 7.5.3, that occur on the day of each vaccine administration and during the 7 days after vaccine administration (ie, the day of injection and 6 subsequent days). If a solicited local or systemic AR continues beyond Day 7 after vaccination, the participant will be prompted to capture details of the solicited local or systemic AR in the eDiary until it is resolves or the next IP injection occurs, whichever occurs first; capture of details of ARs in the eDiary should not exceed 28 days after each vaccination and not to exceed 28 days after each vaccination, whichever occurs first. ARs recorded in the eDiary beyond Day 7 should be reviewed by the study site staff either during the next scheduled telephone call or at the next study site visit.
- Daily oral body temperature measurement should be performed at approximately the same time each day using the thermometer provided by the study site. If body temperature is taken more than once in a given day, only the highest temperature reading should be recorded.
- Measurement, as applicable, for solicited local ARs (injection site erythema and swelling/induration); the size measurements will be performed using the ruler provided by the study site.
- Any medications taken to treat or prevent pain or fever on a day of injection or for the next 6 days.

The eDiary will be the only source documents allowed for solicited systemic or local ARs (including body temperature measurements). Participants or their caregivers will be instructed to complete eDiary entries daily. The participant or their caregiver will have a limited window on the following day to complete assessments for the previous day; quantitative temperature recordings and measurement of any injection site erythema or swelling/induration reported on the following day may be excluded from the analyses of solicited ARs.

Any new safety information reported during safety telephone calls or at study site visits (including a solicited reaction) that is not already captured in the eDiary will be described in the

source documents as a verbally reported event. An event reported in this manner must be described as a solicited event and entered on the solicited AR eCRF.

Study site staff will review eDiary data with participants at visits 7 days after each injection.

The eDiary will also be used every 4 weeks, starting at Day 71 through Day 183 and again starting at Day 223 through Day 363 for participants in Part 1A (Table 10), and on Day 99 through Day 183 and again on Day 223 through Day 307 in Part 2 (Table 15) to capture the occurrence of AEs, MAAEs, SAEs, AESI, or AEs leading to withdrawal. As specified in the applicable SoA (Table 10 and Table 15), the eDiary will prompt the participant to complete an eDiary questionnaire that collects the following data:

- Changes in health since last completing the questionnaire or since in contact with the study site.
- Known exposure to someone with known COVID-19 or SARS-CoV-2 infection.
- Any experience of symptoms of COVID-19.
- Any MAAEs or SAEs.

If an eDiary record results in identification of relevant safety events according to the study period, or of symptoms of COVID-19, a follow-up safety telephone call will be triggered.

Completion of eDiary questionnaires will alternate with safety telephone calls (Section 7.1.2) as the procedure for safety follow-up approximately every 4 weeks starting at Day 85 through Day 197 and again starting at Day 237 through Day 377 (Part 1A SoA, Table 10), every 4 weeks starting at Day 113 through Day 197 and again starting at Day 237 through Day 321 (Part 2, Table 15), and every 6 weeks starting Day 99 through Day 141 and again on Day 223 through Day 321 (Part 3, Table 16).

In Part 1C-1 and Part 1C-2, diary entries will be recorded by the participant at approximately 30 minutes after BD injection while at the study site with instruction provided by study staff. Study participants will continue to record entries in the eDiary each day after they leave the study site, preferably in the evening, on the day of injection and for 6 days following injection. If a solicited local or systemic AR continues beyond Day 7 after vaccination, the participant will be prompted to capture details of the solicited local or systemic AR in the eDiary until the AR resolves; capture of details of ARs in the eDiary should not exceed 28 days after vaccination. ARs recorded in eDiaries beyond Day 7 should be reviewed either via telephone call or at the following study visit. Review of eDiary will occur on BD-Day 8.

7.1.1.1. Ancillary Supplies for Participant Use

Study sites will distribute Sponsor-provided oral thermometers and rulers for use by participants in assessing body temperature and injection site reactions for recording solicited ARs in eDiaries. Based on availability, smartphone devices may be provided to those participants who do not have their own device to use for eDiary activities.

7.1.2. Safety Telephone Calls

A safety telephone call is a telephone call made to the participant by trained study site personnel. This call will follow a script, which will facilitate the collection of relevant safety information.

Safety telephone calls follow a schedule for each participant as indicated in the Part 1A SoA (Table 10), Part 1B (Table 12), Part 1C (Table 13 and Table 14), Part 2 (Table 15), and Part 3 (Table 16).

The participant will be interviewed according to the script about occurrence of AEs, MAAEs, SAEs, AESI, AEs leading to study withdrawal, concomitant medications associated with those events, and any nonstudy vaccinations (Section 7.5.7). In addition, study personnel will collect information on known participant exposure to someone with known COVID-19 or SARS-CoV-2 infection and on participant experience of COVID-19 symptoms. All safety information collected from the telephone contact must be documented in source documents as described by the participant and not documented on the script used for the safety telephone contact. As noted in Section 7.1.1, an unscheduled follow-up safety telephone call may be triggered if an eDiary record results in identification of a relevant safety event.

7.1.3. Safety Laboratory Assessments

No scheduled laboratory assessments for safety are planned. This is based on the absence of clinically significant abnormal laboratory findings in the Phase 1 and Phase 2 studies of mRNA-1273 in adults.

A point-of-care urine pregnancy test will be performed at the Screening Visit (Day 0) and before each vaccine dose. At any time, a pregnancy test either via blood or point-of-care urine can be performed, at the discretion of the investigator.

7.1.4. Vital Sign Measurements

Vital sign measurements will include systolic and diastolic blood pressure, heart rate, respiratory rate, and body temperature (preferred route is oral). The participant will be seated for at least 5 minutes before all measurements are taken. Vital signs will be measured at the timepoints indicated in the SoAs (Table 10, Table 12, Table 13, Table 14, Table 15, and Table 16). At dosing visits, vital sign measurements will be collected once before injection and at least 30 minutes post injection (before participants are discharged from the study site).

Febrile participants at dosing visits (fever is defined as a body temperature ≥38.0°C/≥100.4°F) may have visits rescheduled within the relevant study visit windows. Afebrile participants with minor illnesses may be injected at the discretion of the investigator.

When procedures overlap and are scheduled to occur at the same timepoint, the order of procedures should be vital sign measurements and then the blood collection.

7.1.5. Physical Examinations

A full physical examination, including height and weight, will be performed at scheduled timepoints as indicated in the Part 1A (Table 10), Part 1B (Table 12), and Part 1C (Table 13 and Table 14), Part 2 (Table 15), and Part 3 (Table 16) SoAs. The full examination will include assessment of skin, head, ears, eyes, nose, throat, neck, thyroid, lungs, heart, cardiovascular system, abdomen, lymph nodes, and musculoskeletal system/extremities. Any clinically significant finding identified during a study visit should be reported as a MAAE.

Symptom-directed physical examinations may be performed at other timepoints at the discretion of the investigator. On each injection day before injection and again 7 days after injection, the

arm receiving the injection should be examined and the associated lymph nodes should be evaluated. Any clinically significant finding identified during a study visit should be reported as a MAAE.

Body mass index will be calculated at the Screening Visit (Day 0) only.

7.1.6. Assessment for SARS-CoV-2 Infection

Study participants will have NP or nasal swab samples collected for SARS-CoV-2 testing at timepoints specified in the SoA (Part 1A, Table 10; Part 1B, Table 12; Part 1C, Table 13 and Table 14; Part 2, Table 15; and Part 3, Table 16).

For Part 1A, Part 1B, Part 1C, Part 2, and Part 3, a study illness visit or a consultation will be arranged within 72 hours or as soon as possible to collect an NP or nasal swab sample to ascertain the presence of SARS-CoV-2 via RT-PCR if a participant experiences any of the following:

- Signs or symptoms of SARS-CoV-2 infection as defined by the CDC (CDC 2020b).
- Exposure to an individual confirmed to be infected with SARS-CoV-2.
- MAAE suggesting a SARS-CoV-2 infection.

If the participant had known exposure to COVID-19 (eg, exposure to someone with confirmed COVID-19), it will be captured in the COVID-19 exposure form.

If scheduled, the study illness visit may collect additional clinical information, including assessments such as medical history, physical examination, blood sampling for clinical laboratory testing, and nasal, saliva, and/or NP swab sampling for viral PCR (including multiplex PCR for respiratory viruses including SARS-CoV-2) to evaluate the severity of the clinical case. Radiologic imaging studies may be conducted. All findings will be recorded in the eCRF.

If participants are confirmed to have SARS-CoV-2 infection, the investigator will notify the participant, and the participant's primary care physician, of the diagnosis. If the study participant does not have a primary care physician, the investigator will assist them to obtain one. The participant will also be instructed on infection prevention measures consistent with local public health guidance.

Any confirmed symptomatic SARS-CoV-2 infection occurring in participants will be captured as a MAAE along with relevant concomitant medications and details about severity, seriousness, and outcome. Additionally, a convalescent visit will be scheduled approximately 28 days (+7 days) after diagnosis (Section 7.4.3). At this visit, an NP or nasal swab sampling for viral PCR and a blood sample will be collected for potential immunologic assessment of SARS-CoV-2 infection.

During the ongoing Omicron-related surge in SARS-CoV-2 infections or if any variant of concern-related surge may occur the following alternative process may be used for assessment of SARS-CoV-2 infection:

If an illness visit is not possible due to COVID-19 related restrictions at site or in the wider community, a telehealth visit combined with a local diagnostic test, to be recorded under an unscheduled visit eCRF page, can substitute for a clinic visit.

If due to COVID-19 related restrictions in the community, the participant's LAR performed a home test rather than obtaining a test performed by a health care professional, the following process should apply:

- If the participant reports symptoms but home test was negative, the investigator may report symptoms as 'upper respiratory infection' or other appropriate AE and report the negative home test on the local diagnostic lab page for an unscheduled visit, and forego the illness visit.
- If the participant reports symptoms and the home test was positive, the investigator should encourage the family to seek confirmatory PCR-based testing at a local testing center or return to site for an illness visit.
- If a local confirmatory PCR-based test is obtained, the investigator may report the result under an unscheduled visit and forego the illness visit. A convalescent visit should be scheduled if feasible.
- If due to COVID-19 related restrictions at the site or in the community, neither a local test nor an illness visit is feasible, the investigator may report the result of the home test under the local diagnostic lab page as an unscheduled visit and forego the illness visit. A convalescent visit should be scheduled if feasible.

7.2. Immunogenicity Assessments

Blood samples for immunogenicity assessments will be collected at the timepoints indicated in the SoA (Part 1A, Table 10; Part 1B, Table 12; Part 1C, Table 13 and Table 14; Part 2, Table 15; and Part 3, Table 16): The following analytes will be measured:

- Serum nAb level against SARS-CoV-2 as measured by pseudovirus and/or live virus neutralization assays
  - Serum bAb levels against SARS-CoV-2 as measured by a multiplex serology assay specific to the SARS-CoV-2 S, N, RBD protein.

Serum collected from all participants will be tested for bAb against SARS-CoV-2 nucleocapsid protein at specified timepoints. In addition, serum samples from a selected subset of study participants who received mRNA-1273 or mRNA-1273.222 will be selected for testing of nAb and bAb against the SARS-CoV-2 S protein. Sample aliquots will be designed to ensure that backup samples are available and that vial volumes are likely to be adequate for future testing needs. The actual time and date of each sample collected will be recorded in the eCRF, and unique sample identification will be utilized to maintain the blind at the laboratory at all times and to allow for automated sample tracking and housing. Handling and preparation of the samples for analysis, as well as shipping and storage requirements, will be provided in a separate study manual.

Measurement of bAb and nAb levels will be performed in a laboratory designated by the Sponsor.

According to the ICF (Section 10.2.6), serum from immunogenicity testing may be used for future research, which may be performed at the discretion of the Sponsor to further characterize

the immune response to SARS-CoV-2, additional assay development, and the immune response across CoVs.

The maximum planned volume of blood sampled per participant for immunogenicity assessments in 1 day is 21 mL.

7.3. Exploratory Assessments and Biomarkers

Exploratory assessments may include assessment of biomarkers for safety, reactogenicity, inflammatory and cardiac function. Serologic markers of disease severity, immune response to SARS-CoV-2, RT-PCR of NP or nasal swab or saliva samples, and genetic sequences of SARS-CoV-2 strains isolated from participants' samples may also be measured.

7.4. Efficacy Assessments

7.4.1. Vaccine Effectiveness Assessments

Vaccine effectiveness for adolescents of ages of 12 to < 18 years will be inferred based on serum Ab responses obtained on Day 57 (28 days after the second injection of mRNA-1273) and Day 209 (28 days after the second injection of mRNA1273.222). Inference will be based on assessing the adolescent Ab responses against the following:

1. *If available at the time of analysis*, adolescent Ab responses will be assessed against an accepted serum nAb threshold conferring protection against COVID-19.
2. *If an accepted threshold of protection is not available*, adolescent Ab responses will be assessed by establishing noninferiority of the GM value and SRR of serum nAb from adolescent participants compared with those from young adults enrolled in the ongoing clinical endpoint efficacy trial (Study P301). The statistical parameters to infer effectiveness are described in Section 2.

COVID-19:

To be considered as a case of COVID-19 for the evaluation of the primary efficacy endpoint, the following case definition must be met:

- The participant must have experienced at least TWO of the following systemic symptoms: Fever (≥38ºC/≥100.4ºF), chills, myalgia, headache, sore throat, new olfactory and taste disorder(s), OR
- The participant must have experienced at least ONE of the following respiratory signs/symptoms: cough, shortness of breath or difficulty breathing, OR clinical or radiographical evidence of pneumonia; AND
- The participant must have at least 1 NP or nasal swab or saliva sample (or respiratory sample, if hospitalized) positive for SARS-CoV-2 by RT-PCR.

Severe COVID-19:

To be considered severe COVID-19, the following criteria must be met:

- Confirmed COVID-19 as per the primary efficacy endpoint case definition, plus any of the following:
  - Clinical signs indicative of severe systemic illness, respiratory rates ≥30 per minute, heart rate ≥125 beats per minute, SpO2 ≤93% on room air at sea level or PaO2/FIO2 < 300 mm Hg, OR
  - Respiratory failure or Acute Respiratory Distress Syndrome, (defined as needing high-flow oxygen, noninvasive or mechanical ventilation, or ECMO), evidence of shock (systolic BP < 90 mmHg, diastolic BP < 60 mmHg or requiring vasopressors), OR
  - Significant acute renal, hepatic, or neurologic dysfunction, OR
  - Admission to an ICU or death.

The secondary case definition of COVID-19 is defined as the following systemic symptoms: fever (temperature >38ºC/≥100.4ºF), or chills, cough, shortness of breath or difficulty breathing, fatigue, muscle aches, or body aches, headache, new loss of taste or smell, sore throat, congestion or runny nose, nausea, or vomiting or diarrhea AND a positive NP or nasal swab or saliva sample for SARS-CoV-2 by RT-PCR.

Death attributed to COVID-19 is defined as any participant who dies during the study with a cause directly attributed to a complication of COVID-19.

SARS-CoV-2 Infection:

- SARS-CoV-2 infection is defined in participants with SARS-CoV-2 negative at baseline:
  - bAb level against SARS-CoV-2 nucleocapsid protein negative at Day 1, which becomes positive post baseline, OR
  - Post baseline-Positive RT-PCR.

7.4.2. Surveillance for COVID-19 Symptoms

Surveillance for COVID-19 symptoms will be conducted via biweekly telephone calls or eDiary prompts as specified in Section 7.1.1 and Figure 7; starting after participant enrollment and throughout the study.

If there is no response to an eDiary prompt for 3 days, the study site staff will contact the study participant by phone.

According to the CDC as of 10 Jun 2020 (CDC 2020c), patients with COVID-19 have reported a wide range of symptoms ranging from mild symptoms to severe illness. Throughout the study, to survey for COVID-19, the following prespecified symptoms that meet the criteria for suspicion of COVID-19 will be elicited weekly from the participant and the presence of any one of these symptoms lasting at least 48 hours (except for fever and/or respiratory symptoms) will result in the study site staff arranging an illness visit to collect an NP or nasal swab within 72 hours:

- Fever (temperature ≥38ºC/≥100.4ºF) or chills (of any duration, including ≤48 hours),

- Shortness of breath or difficulty breathing (of any duration, including ≤48 hours)
- Cough (of any duration, including ≤48 hours),
- Fatigue,
- Muscle or body aches,
- Headache,
- New loss of taste or smell,
- Sore throat,
- Congestion or runny nose,
- Nausea or vomiting,
- Diarrhea.

Figure 7: Surveillance for COVID-19 Symptoms and the Corresponding Clinical Data Pathways

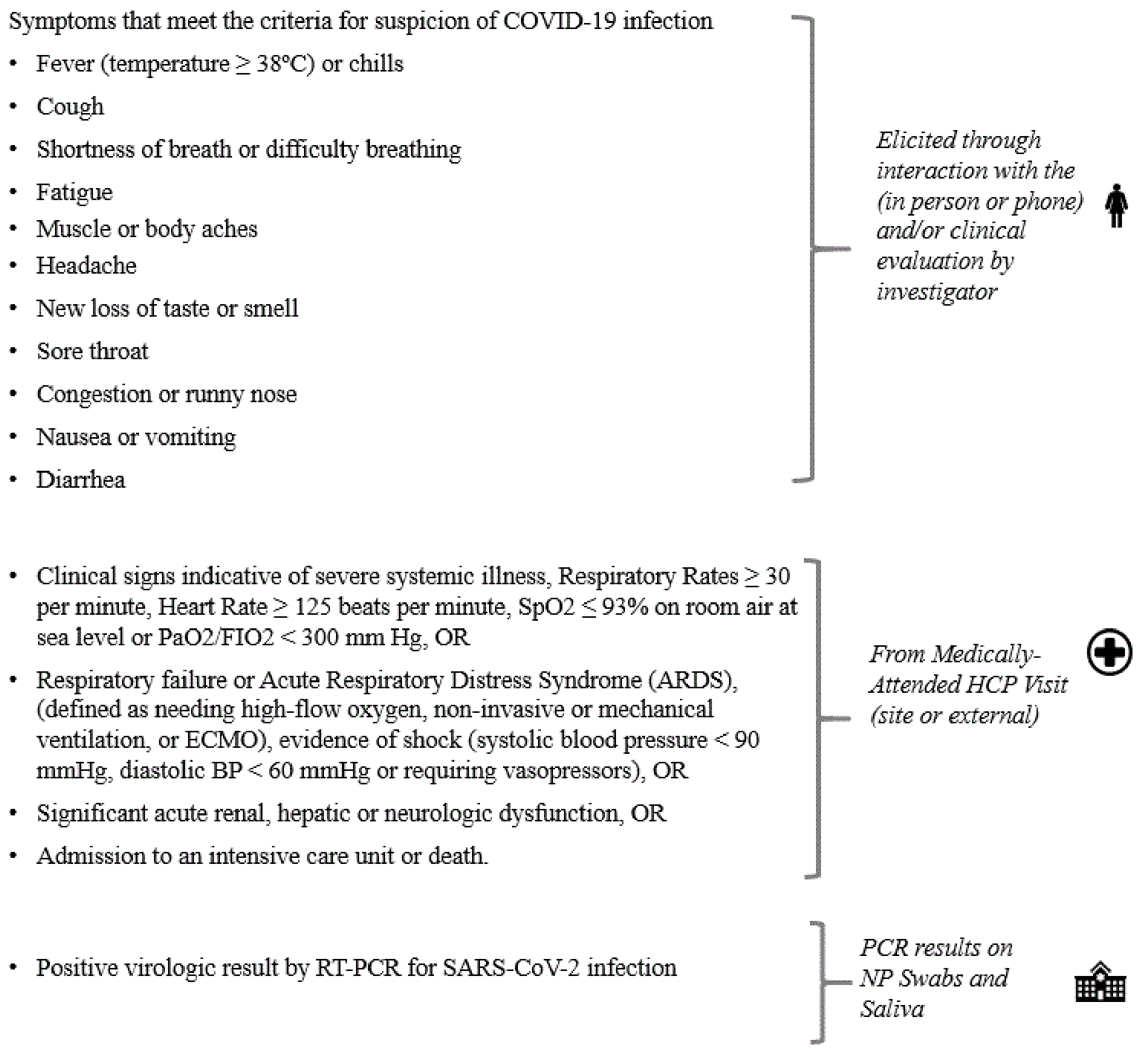

Abbreviations: BP = blood pressure, COVID-19 = coronavirus disease 2019, ECMO, SpO2 = oxygen saturation, PaO2 = partial pressure of oxygen, FIO2 = fraction of inspired oxygen, HCP = healthcare practitioner, RT-PCR = reverse transcriptase polymerase chain reaction, SARS-CoV-2 = Severe Acute Respiratory Syndrome coronavirus 2.

It is important to note that some of the symptoms of COVID-19 overlap with solicited systemic ARs that are expected after vaccination with mRNA-1273 (eg, myalgia, headache, fever, and chills). During the first 7 days after vaccination, when these solicited ARs are common, investigators should use their clinical judgment to decide if an NP or nasal swab should be collected. The collection of an NP or nasal swab prior to each vaccination can help ensure that cases of COVID-19 are not overlooked. Any study participant who reports respiratory symptoms during the 7-day period after vaccination should be evaluated for COVID-19.

During the course of the study, participants with symptoms of COVID-19 will be asked to return within 72 hours or as soon as possible to the study site or medically qualified staff from the study site will conduct a home visit as soon as possible to collect an NP or nasal swab sample (for RT-PCR) for evaluation of COVID-19. Both study site visits and home visits are referred to as illness visits (Section 7.1.6). The NP or nasal swab sample will also be tested for the presence of other respiratory infections. Additionally, a convalescent visit will be scheduled approximately 28 days (+7 days) after diagnosis (for Part 1A and 1B only) (Section 7.4.3). At this visit, an NP or nasal swab sampling for viral PCR and a blood sample will be collected for potential immunologic assessment of SARS-CoV-2 infection. In addition, the study site may collect an additional respiratory sample for SARS-CoV-2 testing to be able to render appropriate medical care for the study participant as determined by local standards of care.

Cases are defined as participants meeting clinical criteria based both on symptoms for COVID-19 and on RT-PCR detection of SARS-CoV-2 from samples collected within 72 hours of the study participant reporting symptoms meeting the definition of COVID-19. Participants who are hospitalized for COVID-19 without the opportunity for a clinic or home visit will also be considered cases, assuming that the symptomology criteria for COVID-19 are met and a respiratory sample is positive for SARS-CoV-2 by PCR at a clinical laboratory improvement amendments-certified laboratory. Investigators are encouraged to try to obtain a respiratory sample during the course of hospitalization for submission to the study central laboratory, if feasible. The investigator should determine if the criteria for severe COVID-19 has been met.

Severe COVID-19 is defined in Section 7.4.1.

All clinical findings will be recorded in the eCRF. All confirmed cases of COVID-19 will be captured as MAAEs, along with relevant concomitant medications and details about severity, seriousness, and outcome, and will be reported immediately to the Sponsor or designee (Section 7.5.4).

7.4.3. Follow-up/Convalescent Period After Diagnosis with COVID-19

Any confirmed symptomatic COVID-19 occurring in a participant will be captured as a MAAE along with relevant concomitant medications and details about severity, seriousness, and outcome. A convalescent visit will be scheduled approximately 28 days (+7 days) after diagnosis (for Part 1A and Part 1B only). At this visit, an NP or nasal swab sampling for viral PCR and a blood sample will be collected for potential immunologic assessment of SARS-CoV-2 infection. The investigator should determine if the criteria for severe COVID-19 has been met. If the

participant is hospitalized, medically qualified study site personnel will try to obtain medical records and SARS-CoV-2 diagnostic results. If the participant is later discharged from the hospital during the 28-day period following diagnosis of COVID-19, the study site personnel will arrange for a resumption of the protocol schedule.

Convalescent visits are not applicable to Part 1C-1, Part 1C-2, Part 2, and Part 3.

7.5. Safety Definitions and Related Procedures

7.5.1. Adverse Event

An AE is defined as any untoward medical occurrence associated with the use of a drug in humans, whether or not considered drug related.

Events Meeting the Adverse Event Definition

- Exacerbation of a chronic or intermittent pre-existing condition including either an increase in frequency and/or intensity of the condition
- New conditions detected or diagnosed after the first dose of IP even though they may have been present before the start of the study

Events NOT Meeting the Adverse Event Definition

- Medical or surgical procedure (eg, endoscopy, appendectomy): the condition that leads to the procedure should be the AE.
- Situations in which an untoward medical occurrence did not occur (social and/or convenience admission to a hospital).

An AR is any AE for which there is a reasonable possibility that the vaccine caused the AE (Section 7.5.9). For the purposes of investigational new drug safety reporting, “reasonable possibility” means that there is evidence to suggest a causal relationship between the vaccine and the AE.

An unsolicited AE is any AE reported by the participant that is not specified as a solicited AR in the protocol or is specified as a solicited AR but starts outside the protocol-defined period for reporting solicited ARs (ie, for the 7 days after each dose of vaccine).

7.5.2. Serious Adverse Events

An AE (including an AR) is considered an SAE if, in the view of either the investigator or Sponsor, it results in any of the following outcomes:

- **Death**
  A death that occurs during the study or that comes to the attention of the investigator during the protocol-defined follow-up period must be reported to the Sponsor, whether or not it is considered related to the IP.
- **Is life-threatening**
  An AE is considered life-threatening if, in the view of either the investigator or the Sponsor, its occurrence places the participant at immediate risk of death. It does not include an AE that, had it occurred in a more severe form, might have caused death.

- **Inpatient hospitalization or prolongation of existing hospitalization**
  In general, inpatient hospitalization indicates the participant was admitted to the hospital or emergency ward for at least one overnight stay for observation and/or treatment that would not have been appropriate in the physician's office or outpatient setting. The hospital or emergency ward admission should be considered an SAE regardless of whether opinions differ as to the necessity of the admission. Complications that occur during inpatient hospitalization will be recorded as an AE; however, if a complication/AE prolongs hospitalization or otherwise fulfills SAE criteria, the complication/AE will be recorded as a separate SAE.
- **Persistent or significant incapacity or substantial disruption of the ability to conduct normal life functions**
  This definition is not intended to include experiences of relatively minor medical significance such as uncomplicated headache, nausea/vomiting, diarrhea, influenza, and accidental trauma (eg, sprained ankle), which may interfere with or prevent everyday life functions but do not constitute a substantial disruption.
- **Congenital anomaly or birth defect**
- **Medically important event**
  Medical judgment should be exercised in deciding whether SAE reporting is appropriate in other situations, such as important medical events that may not be immediately life-threatening or result in death or hospitalization but may jeopardize the participant or require medical or surgical intervention to prevent one of the other outcomes listed in the above definition. These events should usually be considered serious. Examples of such medical events include allergic bronchospasm requiring intensive treatment in an emergency room or at home, blood dyscrasias or convulsions that do not result in inpatient hospitalization, or the development of drug dependency or drug abuse.

7.5.3. Solicited Adverse Reactions

The term "reactogenicity" refers to the occurrence and intensity of selected signs and symptoms (ARs) occurring after IP injection. The eDiary will solicit daily participant reporting of ARs using a structured checklist (Section 7.1.1). Participants will record such occurrences in an eDiary on the day of each dose injection and for the 6 days after the day of dosing (Part 1A, Part 1C, Part 2, and Part 3 only).

Severity grading of reactogenicity will occur automatically based on participant entry into the eDiary according to the grading scales presented in Table 6 modified from the Toxicity Grading Scale for Healthy Adult and Adolescent Volunteers Enrolled in Preventative Vaccine Clinical Trials (DHHS 2007).

If a solicited local or systemic AR continues beyond Day 7 after vaccination, the participant will be prompted to capture details of the solicited local or systemic AR in the eDiary until it resolves or the next IP injection occurs whichever occurs first; capture of details of ARs in the eDiary should not exceed 28 days after each vaccination. ARs recorded in the eDiary beyond Day 7 should be reviewed by the study site staff either during the next scheduled telephone call or at the next study site visit. All solicited ARs (local and systemic) will be considered causally related to dosing.

Table 6: Solicited Adverse Reactions and Grades

Reaction	Grade 0	Grade 1	Grade 2	Grade 3	Grade 4[a]
Injection site pain	None	Does not interfere with activity	Repeated use of over-the-counter pain reliever > 24 hours or interferes with activity	Any use of prescription pain reliever or prevents daily activity	Requires emergency room visit or hospitalization
Injection site erythema (redness)	< 25 mm/ < 2.5 cm	25 – 50 mm/ 2.5 – 5 cm	51 – 100 mm/ 5.1 – 10 cm	> 100 mm/ > 10 cm	Necrosis or exfoliative dermatitis
Injection site swelling/induration (hardness)	< 25 mm/ < 2.5 cm	25 – 50 mm/ 2.5 – 5 cm	51 – 100 mm/ 5.1 – 10 cm	> 100 mm/ > 10 cm	Necrosis
Axillary (underarm) swelling or tenderness ipsilateral to the side of injection	None	No interference with activity	Repeated use of over-the-counter (nonnarcotic) pain reliever > 24 hours or some interference with activity	Any use of prescription (narcotic) pain reliever or prevents daily activity	Emergency room visit or hospitalization
Headache	None	No interference with activity	Repeated use of over-the-counter pain reliever > 24 hours or some interference with activity	Significant; any use of prescription pain reliever or prevents daily activity	Requires emergency room visit or hospitalization
Fatigue	None	No interference with activity	Some interference with activity	Significant; prevents daily activity	Requires emergency room visit or hospitalization
Myalgia (muscle aches all over body)	None	No interference with activity	Some interference with activity	Significant; prevents daily activity	Requires emergency room visit or hospitalization
Arthralgia (joint aches in several joints)	None	No interference with activity	Some interference with activity	Significant; prevents daily activity	Requires emergency room visit or hospitalization
Nausea/vomiting	None	No interference with activity or 1-2 episodes/ 24 hours	Some interference with activity or > 2 episodes/24 hours	Prevents daily activity, requires outpatient intravenous hydration	Requires emergency room visit or hospitalization for hypotensive

Reaction	Grade 0	Grade 1	Grade 2	Grade 3	Grade 4[a]
					shock
Chills	None	No interference with activity	Some interference with activity not requiring medical intervention	Prevents daily activity and requires medical intervention	Requires emergency room visit or hospitalization
Fever (oral)	< 38.0°C<br>< 100.4°F	38.0 – 38.4°C<br>100.4 – 101.1°F	38.5 – 38.9°C<br>101.2 – 102.0°F	39.0 – 40.0°C<br>102.1 – 104.0°F	> 40.0°C<br>> 104.0°F

a. Grading for Grade 4 events per investigator assessment (with exception of fever).
Source: Guidance for industry – Toxicity Grading Scale for Healthy Adult and Adolescent Volunteers Enrolled in Preventative Vaccine Clinical Trials (DHHS 2007).

Any solicited AR that meets any of the following criteria must be entered into the participant's source document and must also be recorded by the study site staff on the solicited AR page of the participant's eCRF:

- Solicited local or systemic AR that results in a visit to an HCP; otherwise meets the definition of a MAAE
- Solicited local or systemic AR leading to the participant withdrawing from the study or the participant being withdrawn from the study by the investigator (AE leading to withdrawal)
- Solicited local or systemic AR lasting beyond 7 days after injection
- Solicited local or systemic AR that leads to participant withdrawal from IP
- Solicited local or systemic AR that otherwise meets the definition of an SAE

7.5.4. Medically Attended Adverse Events

A MAAE is an AE that leads to an unscheduled visit to an HCP. This would include visits to a study site for unscheduled assessments (eg, abnormal laboratory test results follow-up, COVID-19 [Section 7.4.1]) and visits to HCPs external to the study site (eg, urgent care, primary care physician). Investigators will review unsolicited AEs for the occurrence of any MAAE. Unsolicited AEs will be captured on the AE page of the eCRF.

All confirmed symptomatic COVID-19 cases (Section 7.4.1) will be recorded as MAAEs and reported to the Sponsor or designee immediately and in all circumstances within 24 hours, using the SAE Mailbox, the SAE Hotline, or the SAE Fax line (Section 7.5.11). The investigator will submit any updated COVID-19 case data to the Sponsor within 24 hours of it being available.

7.5.4.1. Anaphylaxis

All suspected cases of anaphylaxis should be recorded as MAAEs and reported as SAEs (Section 7.5.2) based on criteria for a medically important event unless the event meets other serious criteria. As an SAE, the event should be reported to the Sponsor or designee immediately and in all circumstances within 24 hours as per Section 7.5.10 (Reporting SAEs). The investigator will submit any updated anaphylaxis case data to the Sponsor within 24 hours of it

being available. For reporting purposes, a participant who displays signs/symptoms consistent with anaphylaxis as shown below should be reported as a potential case of anaphylaxis. This is provided as general guidance for investigators and is based on the Brighton Collaboration case definition (Rüggeberg et al 2007).

Anaphylaxis is an acute hypersensitivity reaction with multi-organ-system involvement that can present as or rapidly progress to a severe life-threatening reaction. It may occur following exposure to allergens from a variety of sources. Anaphylaxis is a clinical syndrome characterized by:

- Sudden onset AND
- Rapid progression of signs and symptoms AND
- Involving 2 or more organ systems, as follows:
  - **Skin/mucosal**: urticaria (hives), generalized erythema, angioedema, generalized pruritus with skin rash, generalized prickle sensation, red and itchy eyes
  - **Cardiovascular**: measured hypotension, clinical diagnosis of uncompensated shock, loss of consciousness or decreased level of consciousness, evidence of reduced peripheral circulation
  - **Respiratory**: bilateral wheeze (bronchospasm), difficulty breathing, stridor, upper airway swelling (lip, tongue, throat, uvula, or larynx), respiratory distress, persistent dry cough, hoarse voice, sensation of throat closure, sneezing, rhinorrhea
  - **Gastrointestinal**: diarrhea, abdominal pain, nausea, vomiting

7.5.5. Adverse Events of Special Interest

An AESI is an AE (serious or nonserious) of scientific and medical concern specific to the Sponsor’s product or program, for which ongoing monitoring and immediate notification by the investigator to the Sponsor is required and documentation is in the form of a case narrative. Such events may require further investigation to characterize and understand them. Refer to Section 10.4, Appendix 4 for a list of AESIs pertinent to this study. All AESIs will be collected throughout the entire study period and must be reported to the Sponsor or designee immediately and in all circumstances within 24 hours of becoming aware of the event via the EDC system. If a site receives a report of a new AESI from a study participant or receives updated data on a previously reported AESI and the eCRF has been taken offline, then the site can report this information on a paper AESI form using the SAE Mailbox, the SAE Hotline, or the SAE Fax line (Section 7.5.11).

7.5.5.1. Acute Myocarditis and/or Pericarditis

These definitions are intended to serve as a guide to help in the reporting of suspected cases of myocarditis and/or pericarditis; however, the diagnosis of suspected cases is left to the investigator’s clinical judgment.

The investigator’s medical judgment must be applied when parent(s)/LAR(s) report that a participant has symptoms concerning for myocarditis and/or pericarditis per the CDC case definition. Diagnostic evaluation (eg, EKG, echocardiogram) and laboratory testing (eg,

troponin) per the CDC definition of myocarditis and/or pericarditis must be promptly obtained for any participant with concerning signs/symptoms. Referral to a pediatric cardiologist should be obtained in those with positive test results or clinically significant symptoms without other identifiable causes. Additional testing and evaluation may be indicated. Once available, updated myocarditis, pericarditis, or myopericarditis case data will be submitted by the investigator to the Sponsor within 24 hours.

All suspected cases of probable and confirmed myocarditis, pericarditis, or myopericarditis should be recorded as an AESI, and reported as an SAE, if the event meets seriousness criteria. As an SAE, the event should be reported to the Sponsor or designee immediately and in all circumstances within 24 hours as per Section 7.5.12. The investigator will submit any updated myocarditis, pericarditis, or myopericarditis case data to the Sponsor within 24 hours of it being available. For reporting purposes, a participant who displays signs/symptoms consistent with the CDC case definitions as described below (Gargano et al 2021), should be reported as a potential case of confirmed or probable myocarditis, pericarditis, or myopericarditis.

7.5.5.2. Acute Myocarditis Case Definition

Presence of ≥1 new or worsening of the following clinical symptoms (persons who lack the listed symptoms but who meet other criteria may be classified as subclinical myocarditis [probable or confirmed]):

- Chest pain/pressure/discomfort
- Dyspnea/shortness of breath/pain with breathing
- Palpitations
- Syncope

OR

Infants and children aged <12 years might instead have ≥2 of the following symptoms:

- Irritability
- Vomiting
- Poor feeding
- Tachypnea
- Lethargy

AND

For PROBABLE CASE:

Presence of ≥1 new finding of the following:

- Troponin level above upper limit of normal (any type of troponin)
- Abnormal ECG or EKG or rhythm monitoring findings consistent with myocarditis
  - To meet the ECG or rhythm monitoring criterion, a probable case must include at least one of the following:

    - ST segment or T-wave abnormalities
    - Paroxysmal or sustained atrial, supraventricular, or ventricular arrhythmias
    - AV nodal conduction delays or intraventricular conduction defects
- Abnormal cardiac function or wall motion abnormalities on echocardiogram
- Cardiac MRI finding consistent with myocarditis (Ferreira et al 2018)

AND

- No other identifiable cause of the symptoms and findings

For CONFIRMED CASE:

- Histopathologic confirmation of myocarditis (using Dallas criteria [Aretz et al 1987])

OR

- Cardiac magnetic resonance imaging findings consistent with myocarditis in the presence of troponin level above upper limit of normal (any type of troponin)

AND

- No other identifiable cause of the symptoms and findings

7.5.5.3. Acute Pericarditis Case Definition

Presence of ≥2 new or worsening of the following clinical features (Adler et al 2015):

- Acute chest pain (Typically described as pain made worse by lying down, deep inspiration, or cough; and relieved by sitting up or leaning forward, although other types of chest pain may occur)
- Pericardial rub on examination
- New ST-elevation or PR-depression on EKG
- New or worsening pericardial effusion on echocardiogram or magnetic resonance imaging

7.5.5.4. Myopericarditis Case Definition

Participants who meet criteria for both myocarditis and pericarditis may be described under myopericarditis.

7.5.5.5. Multisystem Inflammatory Syndrome in Children Case Definition

Investigators will also be asked to report, as AESI, clinical signs/symptoms consistent with the CDC case definition of MIS-C (https://emergency.cdc.gov/han/2020/han00432.asp):

- An individual aged < 21 years presenting with fever, laboratory evidence of inflammation, and evidence of clinically severe illness requiring hospitalization, with multisystem (>2) organ involvement (cardiac, renal, respiratory, hematologic, gastrointestinal, dermatologic or neurological);

AND

- No alternative plausible diagnoses;

AND

- Positive for current or recent SARS-CoV-2 infection by RT-PCR, serology, or antigen test; or COVID-19 exposure within the 4 weeks prior to the onset of symptoms:
    1. Fever ≥ 38.0°C/≥ 100.4ºF for ≥ 24 hours, or report of subjective fever lasting ≥24 hours
    2. Including, but not limited to, one or more of the following: an elevated CRP, ESR, fibrinogen, procalcitonin, d-dimer, ferritin, LDH, or IL-6, elevated neutrophils, reduced lymphocytes, and low albumin

Some individuals may fulfill full or partial criteria for Kawasaki disease but should be reported if they meet the case definition for MIS-C. Consider MIS-C in any pediatric death with evidence of SARS-CoV-2 infection.

All cases of MIS-C will be reported to the Sponsor or designee immediately and in all circumstances within 24 hours, using the SAE Mailbox, the SAE Hotline, or the SAE Fax line (Section 7.5.11).

7.5.6. Recording and Follow-up of Pregnancy

Female individuals who have a positive pregnancy test at Screening should not be enrolled; participants who have a positive pregnancy test any time during the study should receive no further dosing with IP but should be asked to remain in the study and be monitored for safety.

Details of all pregnancies in female participants will be collected after the start of study treatment and until the end of their participation in the study.

- If a pregnancy is reported, the investigator should inform the Sponsor within 24 hours of learning of the pregnancy and should follow the procedures outlined in this section.
- Abnormal pregnancy outcomes (eg, spontaneous abortion, fetal death, stillbirth, congenital anomalies, ectopic pregnancy) are considered SAEs.

Pregnancies occurring in participants after enrollment must be reported to Sponsor or designee within 24 hours of the study site learning of its occurrence, using the SAE Mailbox, the SAE Hotline, or the SAE Fax line (Section 7.5.11). If the participant agrees to submit this information, the pregnancy must be followed to determine the outcome, including spontaneous or voluntary termination, details of the birth, and the presence or absence of any birth defects, congenital abnormalities, or maternal and/or newborn complications. This follow-up should occur even if intended duration of the safety follow-up for the study has ended. Pregnancy report forms will be distributed to the study site to be used for this purpose. The investigator must immediately (within 24 hours of awareness) report to the Sponsor any pregnancy resulting in an abnormal outcome according to the procedures described for SAEs.

7.5.7. Eliciting and Documenting Adverse Events

The investigator is responsible for ensuring that all AEs and SAEs are recorded in the eCRF and reported to the Sponsor.

Solicited ARs will be collected from Day 1 through 7 days after each dose in Part 1A, Part 1C, Part 2, and Part 3. Other (unsolicited) AEs will be collected from Day 1 through 28 days after each dose in Part 1A, Part 1C, Part 2, and Part 3.

Both MAAEs and SAEs will be collected from participants as specified in the SoA until the end of their participation in each of the study parts (ie, Day 394 in Part 1A, OL-Day 178 in Part 1B, BD-Day 361 in Part 1C, Day 394 in Part 2, and Day 361 in Part 3). Any AEs that occur before administration of IP will be analyzed separately from AEs.

At every study site visit or telephone contact, participants will be asked a standard question to elicit any medically related changes in their well-being (including COVID-19 symptoms) according to the scripts provided. Participants will also be asked if they have been hospitalized, had any accidents, used any new medications, changed concomitant medication regimens (both prescription and over-the-counter medications), or had any nonstudy vaccinations.

In addition to participant observations, physical examination findings, or data relevant to participant safety classified as an AE will be documented on the AE page of the eCRF.

After the initial AE/SAE report, the investigator is required to proactively follow each participant at subsequent visits/contacts. All AEs and SAEs will be treated as medically appropriate and followed until resolution, stabilization, the event is otherwise explained, or the participant is LTFU (as defined in Section 6.4).

7.5.8. Assessment of Intensity

An event is defined as “serious” when it meets at least one of the predefined outcomes as described in the definition of an SAE (Section 7.5.2), NOT when it is rated as severe.

The severity (or intensity) of an AR or AE refers to the extent to which it affects the participant’s daily activities. The Toxicity Grading Scale for Healthy Adult and Adolescent Volunteers Enrolled in Preventative Vaccine Clinical Trials (DHHS 2007) will be used to categorize local and systemic reactogenicity events (solicited ARs), and vital sign measurements observed during this study. Specific criteria for local and systemic reactogenicity events are presented in Section 7.5.3.

The determination of severity for all unsolicited AEs should be made by the investigator based upon medical judgment and the definition of severity as follows:

- Mild: These events do not interfere with the participant’s daily activities.
- Moderate: These events cause some interference with the participant’s daily activities and require limited or no medical intervention.
- Severe: These events prevent the participant’s daily activity and require intensive therapeutic intervention.

Study staff should elicit from the participant the impact of AEs on the participant’s activities of daily living to assess severity and document appropriately in the participant’s source documentation. Changes in the severity of an AE should be documented in the participant’s source documentation to allow an assessment of the duration of the event at each level of intensity to be performed. An AE characterized as intermittent requires documentation of onset

and duration of each episode. An AE that fluctuates in severity during the course of the event is reported once in the eCRF at the highest severity observed.

7.5.9. Assessment of Causality

The investigator's assessment of an AE's relationship to IP is part of the documentation process but is not a factor in determining what is or is not reported in the study.

The investigator will assess causality (ie, whether there is a reasonable possibility that the IP caused the event) for all AEs and SAEs. The relationship will be characterized using the following classification:

Not related: There is not a reasonable possibility of a relationship to the IP. Participant did not receive the IP OR temporal sequence of the AE onset relative to administration of the IP is not reasonable OR the AE is more likely explained by another cause than the IP.

Related: There is a reasonable possibility of a relationship to the IP. There is evidence of exposure to the IP. The temporal sequence of the AE onset relative to the administration of the IP is reasonable. The AE is more likely explained by the IP than by another cause.

7.5.10. Reporting Adverse Events

The investigator is responsible for reporting all AEs that are observed or reported during the study, regardless of their relationship to IP or their clinical significance. If there is any doubt as to whether a clinical observation is an AE, the event should be reported.

All unsolicited AEs reported or observed during the study will be recorded on the AE page of the eCRF. Information to be collected includes the type of event, time of onset, investigator-specified assessment of severity (impact on activities of daily living) and relationship to IP, time of resolution of the event, seriousness, as well as any required treatment or evaluations, and outcome. The unsolicited AEs resulting from concurrent illnesses, reactions to concurrent illnesses, reactions to concurrent medications, or progression of disease states must also be reported. All AEs will be followed until they are resolved or stable or judged by the investigator to be not clinically significant. The MedDRA will be used to code all unsolicited AEs.

Any medical condition that is present at the time that the participant is screened but does not deteriorate should not be reported as an unsolicited AE. However, if it deteriorates at any time during the study, it should be recorded as an unsolicited AE.

7.5.11. Reporting SAEs

Prompt notification by the investigator to the Sponsor of an SAE is essential so that legal obligations and ethical responsibilities toward the safety of participants and the safety of a study intervention under clinical investigation are met.

Any AE considered serious by the investigator or that meets SAE criteria (Section 7.5.2) must be reported to the Sponsor immediately (within 24 hours of becoming aware of the SAE) via the EDC system. The investigator will assess whether there is a reasonable possibility that the IP caused the SAE. The Sponsor will be responsible for notifying the relevant regulatory authorities

of any SAE as outlined in the 21 US CFR Part 312 and Part 320. The investigator is responsible for notifying the IRB directly.

If the eCRF is unavailable at the time of the SAE, the following contact information is to be used for SAE reporting:

- SAE Mailbox: drugsafety@modernatx.com
- SAE Fax line: +1-617-649-3910

Regulatory reporting requirements for SAE are described in Section 7.5.15.

The investigator and any qualified designees are responsible for detecting, documenting, and recording events that meet the definition of an AE, including SAEs, and remain responsible for following up AEs that are serious, considered related to IP or study procedures, or that caused the participant to discontinue the study.

7.5.12. Time Period and Frequency for Collecting AE and SAE Information

Medical occurrences that begin before the start of IP dosing but after obtaining informed consent will be recorded in the Medical History/Current Medical Conditions Section of the eCRF and not in the AE Section; however, if the condition worsens at any time during the study, it will be recorded and reported as an AE.

Adverse events may be collected as follows:

- Observing the participant
- Receiving an unsolicited complaint from the participant
- Questioning the participant in an unbiased and nonleading manner

Solicited ARs will be collected from the day of injection through 6 days after each dose. Other (unsolicited) AEs will be collected from the day of injection through 28 days after each dose.

Serious AEs will be collected from the start of IP dosing until the last day of study participation.

All SAEs will be recorded and reported to the Sponsor or designee immediately and in all circumstances within 24 hours. The investigator will submit any updated SAE data to the Sponsor within 24 hours of it being available.

An abnormal value or result from a clinical or laboratory evaluation can also indicate an AE if it is determined by the investigator to be clinically significant (eg, leads to dose modification or study drug discontinuation, or meets any serious criteria). If this is the case, it must be recorded in the source document and as an AE on the appropriate AE form(s). The evaluation that produced the value or result should be repeated until that value or result returns to normal or is stabilized and the participant’s safety is not at risk.

Investigators are not obligated to actively seek AEs or SAEs after EOS participation. However, if the investigator learns of any SAE (including a death) at any time after a participant has withdrawn from or completed the study, and the investigator considers the event to be reasonably related to the IP or study participation, the investigator must promptly notify the Sponsor.

7.5.13. Method of Detecting AEs and SAEs

Electronic diaries have specifically been designed for this study by the Sponsor. The diaries will include prelisted AEs (solicited ARs) and intensity scales; they will also include blank space for the recording of information on other AEs (unsolicited AEs) and concomitant medications/vaccinations.

The investigator is responsible for the documentation of AEs regardless of treatment group or suspected causal relationship to IP. For all AEs, the investigator must pursue and obtain information adequate to determine the outcome of the AE and to assess whether the AE meets the criteria for classification as an SAE requiring immediate notification to the Sponsor or its designated representative.

Care will be taken not to introduce bias when detecting AEs and/or SAEs. Open-ended and nonleading verbal questioning of the participant is the preferred method to inquire about the occurrence of AE.

7.5.14. Follow-up of AEs and SAEs

After the initial AE/SAE report, the investigator is required to proactively follow each participant at subsequent visits and contacts.

All AEs and SAEs will be treated as medically appropriate and followed until resolution, stabilization, the event is otherwise explained, or the participant is LTFU, as defined in Section 6.4.

7.5.15. Regulatory Reporting Requirements for SAEs

- Prompt notification by the investigator to the Sponsor of an SAE is essential so that legal obligations and ethical responsibilities toward the safety of participants and the safety of a study intervention under clinical investigation are met.
- The Sponsor has a legal responsibility to notify both the local regulatory authority and other regulatory agencies about the safety of a study intervention under clinical investigation. The Sponsor will comply with country-specific regulatory requirements relating to safety reporting to the regulatory authority, IRBs, and investigators.
- Investigator safety reports must be prepared for suspected unexpected serious adverse reaction according to local regulatory requirements and Sponsor policy and forwarded to investigators as necessary.
- An investigator who receives an investigator safety report describing an SAE or other specific safety information (eg, summary or listing of SAEs) from the Sponsor will review and then file it along with the IB and will notify the IRB, if appropriate according to local requirements.

7.6. Safety Monitoring

The CRO’s medical monitor, the Sponsor’s medical monitor, and the individual study site investigators will monitor safety throughout the study.

7.6.1. Data Safety Monitoring Board

Safety oversight will be under the direction of a DSMB composed of external independent consultants with relevant expertise. Members of the DSMB will be independent from the study conduct and free of conflict of interest.

For the blinded phase of Part 1 of the study, the DSMB will have separate meetings by teleconference to review unblinded safety data when half of the study population (1500 randomized participants) have reached Day 8 (1 week after Dose 1) and again approximately when 25% (750), 50% (1500), and 75% (2250) of enrolled participants have reached Day 36 (1 week after Dose 2). Recruitment will continue, as applicable, during the DSMB review period. The DSMB will operate under the rules of an approved charter that will be written and reviewed at the organizational meeting of the DSMB. Details regarding the DSMB composition, responsibilities, procedures, and frequency of data review will be defined in its charter.

For Part 2 and Part 3 of the study, the DSMB will convene on an ad hoc basis if any of the pause rules, described in Section 6.4, are met. The DSMB will review all available unblinded study data to adjudicate any potential study pauses and make recommendations on further study conduct, including requesting additional information, recommending stopping the study, recommending changes to study conduct and/or the protocol, or recommending additional operational considerations due to safety issues that arise during the study.

7.6.2. Independent Cardiac Event Adjudication Committee

An independent CEAC of medically qualified personnel, including cardiologists, will review all suspected cases of myocarditis and pericarditis to determine if they meet CDC criteria of "probable" or "confirmed" events which are reported in ongoing interventional clinical trials per the CEAC charter (Gargano et al 2021). Any case that the CEAC assesses as representing probable or confirmed cases of myocarditis or pericarditis will be referred to the Sponsor, who will then determine if additional action is needed.

The CEAC will operate under the rules of an approved charter that will be written and reviewed at the organizational meeting of the CEAC. Details regarding the CEAC composition, responsibilities, procedures, and frequency of data review will be defined in its charter.

7.7. Treatment of Overdose

As the study treatment is to be administered by a healthcare professional, it is unlikely that an overdose will occur. Dose deviations will be tracked as protocol deviations (Section 10.2.8).

7.8. Pharmacokinetics

Pharmacokinetic parameters are not evaluated in this study.

7.9. Pharmacodynamics

Pharmacodynamic parameters are not evaluated in this study.

7.10. Biomarkers

Immunogenicity assessments are presented in Section 7.2. Biomarker plasma and biomarker serum samples will be stored for potential future biomarker assessment.

7.11. Health Economics

Health economics are not evaluated in this study.

8. STATISTICAL ANALYSIS PLAN

This section summarizes the planned statistical analysis strategy and procedures for the study. The details of statistical analysis will be provided in the SAP, which will be finalized before the clinical database lock for the study. If changes are made to primary and/or key secondary objectives and hypotheses or the statistical methods related to those hypotheses after the study has begun but prior to any data unblinding, then the protocol will be amended (consistent with ICH Guideline E9). Changes to other secondary or exploratory analyses made after the protocol has been finalized, along with an explanation as to when and why they occurred, will be listed in the SAP or CSR for the study. Ad hoc exploratory analyses, if any, will be clearly identified in the CSR.

8.1. Blinding and Responsibility for Analyses

See Section 3.1.1.1 regarding the addition of a Participant Decision Visit as part of this protocol amendment.

Part 1A of this study is observer-blind. The investigator, study staff, study participants, study site monitors, and Sponsor personnel (or its designees) will be blinded to the IP administered until study end or initiation of Part 1B or Part 1C or Part 2, with the following exceptions:

- Unblinded pharmacy personnel (of limited number) will be assigned to vaccine accountability procedures and will prepare and administer mRNA-1273 (or placebo) to all participants. These pharmacy personnel will have no study functions other than study vaccine management, documentation, accountability, preparation, and administration. They will not be involved in participant evaluations and will not reveal the identity of IP to either the participant or the blinded study site personnel involved in the conduct of the study unless this information is necessary in the case of an emergency.
- Unblinded study site monitors, not involved in other aspects of monitoring, will be assigned as the IP accountability monitors. They will have responsibilities to ensure that study sites are following all proper IP accountability, preparation, and administration procedures.
- An unblinded statistical and programming team will perform the preplanned IA (Section 8.7.1). Sponsor team members will be prespecified to be unblinded to the IA results and will not communicate the results of IA to the blinded investigators, study site staff, clinical monitors, or participants. This is detailed in the study Data Blinding Plan.

In Part 1A, the dosing assignment will be concealed by having the unblinded pharmacy personnel prepare the IP in a secure location that is not accessible or visible to other study staff. An opaque sleeve over the syringe used for injection will maintain the blind at the time of injection, as the doses containing mRNA-1273 will look different to that of placebo. Only delegated unblinded study site staff will conduct the injection procedure. Once the injection is completed, only the blinded study staff will perform further assessments and interact with the participants. Access to the randomization code will be strictly controlled at the pharmacy.

As documented in the study Data Blinding Plan, the prespecified Sponsor and CRO personnel will be unblinded for the IA of immunogenicity, safety, and efficacy.

The purpose of the unblinding is to enable the group to develop regulatory submission documents and to address questions from regulatory agencies during the regulatory review of the submission. After unblinding, this unblinded team will not participate in the conduct or execution of the subsequent course of the study. The study Data Blinding Plan provides details of the blinding/unblinding process and personnel. The study site staff, investigators, study monitors, and participants will remain blinded until the initiation of Part 1B or Part 1C or Part 2.

8.1.1. Breaking the Blind

A participant or participants may be unblinded in the event of an SAE or other severe event, or if there is a medical emergency requiring the identity of the product to be known to properly treat a participant. If a participant becomes seriously ill or pregnant during the study, the blind will be broken if knowledge of the administered vaccine will affect that participant's dosing options. In the event of a medical emergency requiring identification of the vaccine administered to an individual participant, the investigator will make every attempt to contact the Sponsor medical lead to explain the need for opening the code within 24 hours of opening the code. The investigator will be responsible for documenting the time, date, reason for the code break, and the names of the personnel involved.

In addition to the aforementioned situations where the blind may be broken, the data will also be unblinded to a statistical team at specified timepoints for IA as outlined in Section 8.7.1.

8.2. Statistical Hypothesis

8.2.1. Part 1

8.2.1.1. Part 1A

If an accepted serum Ab threshold of protection against COVID-19 is established for the primary immunogenicity objective, the null hypothesis is that the percentage of participants on mRNA-1273 with serum Ab equal to or above the established threshold at Day 57 is ≤ 70% (ie, H_0: percentage of participants on mRNA-1273 ≤ 70% with serum Ab at Day 57 equal to or above the established threshold).

The study would be considered as meeting the immunogenicity objective if the 95% CI of percentage of participants on mRNA-1273 rules out 70% (lower bound of the 95% CI > 70%).

If an accepted serum Ab threshold of protection against COVID-19 is not available for the primary immunogenicity objective, the immunogenicity analysis of primary vaccine response will be performed using the noninferiority tests of the 2 null hypotheses based on the 2 coprimary endpoints, respectively.

<u>Coprimary endpoint 1: Ab GM value at Day 57</u>

The null hypothesis H^1_0: immunogenicity response to mRNA-1273 as measured by Ab GM value at Day 57 is inferior in adolescents (12 to < 18 years of age) compared with that in young adults (18 to 25 years of age) using mRNA-1273 Study P301 data.

The noninferiority in Ab GM in adolescents compared with that in young adults (18 to 25 years of age) is demonstrated by that the lower bound of the 95% CI of the GMR rules out 0.667 (lower bound >0.667) using a noninferiority margin of 1.5. The GMR is the ratio of the GM

value of adolescents on mRNA-1273 in this study, Study P203, at Day 57 compared with the GM value of young adults (18 to 25 years of age) on mRNA-1273 in Study P301.

Coprimary endpoint 2: Ab SRR at Day 57

A definition of seroresponse will be provided in the SAP based on forthcoming information about assay performance.

The null hypothesis H^2_0: immunogenicity response to mRNA-1273 as measured by SRR at Day 57 is inferior in adolescents (12 to < 18 years of age) compared with that in young adults (18 to 25 years of age) using mRNA-1273 Study P301 data.

The noninferiority in SRR in adolescents compared with that in young adults (18 to 25 years of age) is demonstrated by that the lower bound of the 95% CI of the SRR difference rules out -10% (ie, lower bound >-10%) using the noninferiority margin of 10%. The SRR difference is defined as the rate in adolescents receiving mRNA-1273 minus the rate in young adults (18 to 25 years of age) receiving mRNA-1273 from Study P301.

The study would be considered as meeting the primary immunogenicity objective if noninferiority is demonstrated based on both coprimary endpoints.

Details regarding the assay to be used to assess noninferiority will be provided in the SAP.

8.2.1.2. Part 1C–1 – Homologous Booster Phase

The immunogenicity analysis of BD vaccine response against the ancestral strain will be performed using the noninferiority tests of the 2 null hypotheses based on the 2 coprimary endpoints, respectively.

Coprimary Endpoint 1: Ab GM value at BD-Day 29

Null hypothesis H^1_0: immunogenicity response to mRNA-1273 BD as measured by Ab GM value at BD-Day 29 in adolescents (12-<18 years of age) in Study P203 Part 1C-1 is inferior compared with Ab GM value at Day 57 (28 days after Dose 2) in the primary series of mRNA-1273 in young adults (18 to 25 years of age) in Study P301.

The noninferiority in Ab GM value at BD-Day 29 in Study P203 Part 1C-1 compared with Ab GM value at Day 57 in the primary series in young adults (18 to 25 years of age) in Study P301 will be demonstrated by the GMR 95% CI lower bound >0.667 using a noninferiority margin of 1.5. The GMR is defined as the GM value of Ab at BD-D29 in Study P203 Part 1C-1 compared with Ab GM value at Day 57 (28 days after Dose 2) following the primary series of mRNA-1273 in Study P301.

Coprimary Endpoint 2: Ab Seroresponse Rate (SRR) at BD-Day 29

Null hypothesis H^2_0: immunogenicity response to mRNA-1273 BD as measured by SRR at BD-Day 29 in adolescents (12-<18 years of age) in Study P203 Part 1C-1 is inferior compared with SRR at Day 57 (28 days after Dose 2) following the primary series of mRNA-1273 in young adults (18 to 25 years of age) in Study P301.

The noninferiority in SRR at BD-D29 compared with SRR at Day 57 (28 days after Dose 2) following the primary series of mRNA-1273 will be demonstrated by the SRR difference 95% CI lower bound >-10% using the noninferiority margin of 10%. The SRR difference is defined as

the SRR at BD-Day 29 Study P203 Part 1C-1 minus the rate at Day 57 (28 days after Dose 2) following the primary series of mRNA-1273 in young adults in Study P301. Seroresponse is defined as Ab value change from baseline (preDose 1) below the LLOQ to ≥4 × LLOQ, or at least a 4-fold rise if baseline is ≥ LLOQ.

The primary immunogenicity objective in Part 1C-1 is met if the noninferiority is demonstrated based on both coprimary endpoints.

Key Secondary Endpoint 1: GM value of Ab Against Circulating Strain at BD-Day 29

Null hypothesis H^1_0: immunogenicity response to mRNA-1273 BD as measured by GM value of Ab against circulating strain at BD-Day 29 in adolescents (12-<18 years of age) in Study P203 Part 1C-1 is inferior compared with GM value of Ab against circulating strain at Day 57 (28 days after Dose 2) in the primary series of mRNA-1273 in young adults (18 to 25 years of age) in Study P301.

The noninferiority in Ab GM value at BD-Day 29 in Study P203 Part 1C-1 compared with Ab GM value at Day 57 in the primary series in young adults (18 to 25 years of age) in Study P301 will be demonstrated by the GMR 95% CI lower bound >0.667 using a noninferiority margin of 1.5. The GMR is defined as the ratio of GM value of Ab at BD-Day 29 in Study P203 Part 1C-1 compared with Ab GM value at Day 57 (28 days after Dose 2) following the primary series of mRNA-1273 in Study P301.

Key Secondary Endpoint 2: Seroresponse Rate (SRR) of Ab Against Circulating Strain at BD-Day 29

Null hypothesis H^2_0: immunogenicity response to mRNA-1273 BD as measured by SRR of Ab against circulating strain at BD-Day 29 in adolescents (12-<18 years of age) in Study P203 Part 1C-1 is inferior compared with SRR of Ab against circulating strain at Day 57 (28 days after Dose 2) following the primary series of mRNA-1273 in young adults (18 to 25 years of age) in Study P301.

The noninferiority in SRR at BD-D29 compared with SRR at Day 57 (28 days after Dose 2) following the primary series of mRNA-1273 will be demonstrated by the SRR difference 95% CI lower bound >-10% using the noninferiority margin of 10%. The SRR difference is defined as the SRR at BD-Day 29 Study P203 Part 1C-1 minus the rate at Day 57 (28 days after Dose 2) following the primary series of mRNA-1273 in young adults in Study P301. Seroresponse is defined as Ab value change from baseline (pre Dose 1) below the LLOQ to ≥4 × LLOQ, or at least a 4-fold rise if baseline is ≥ LLOQ.

The key secondary immunogenicity objective in Part 1C-1 is met if the noninferiority is demonstrated based on both key secondary endpoints.

8.2.2. Part 2

There will be no hypothesis testing in Part 2, given that the enrollment in Part 2 was discontinued (Section 8.3.2). All analyses for Part 2 will be descriptive based on available data.

8.2.3. Part 3

Hypothesis testing for the endpoints that infer effectiveness of the 50-µg mRNA-1273.222 in adolescents will be performed on the null hypotheses for the 2 coprimary endpoints as follows:

Coprimary Endpoint 1: Post Dose 1 Ab GM value against Omicron BA.4/BA.5 at Day 29 (superiority testing)

The null hypothesis H0: GM value of Ab against Omicron BA.4/BA.5 at Day 29 post Dose 1 of 50 µg mRNA-1273.222 in adolescents who are baseline SARS-CoV-2 positive is not superior to GM value at Day 57 post Dose 2 of 100 µg mRNA-1273 in baseline SARS-CoV-2 negative young adults (18 to 25 years of age) in Study P301.

The superiority in Ab GM value against Omicron BA.4/BA.5 in adolescents who are baseline SARS-CoV-2 positive compared with that in baseline SARS-CoV-2 negative young adults (18 to 25 years of age) is demonstrated by meeting the following success criterion:

- The lower bound of the 95% CI of the GMR >1.

The GMR is the ratio of the GM value against Omicron BA.4/BA.5 at Day 29 post Dose 1 of 50 µg mRNA-1273.222 in adolescents who are baseline SARS-CoV-2 positive in Study P203 Part 3 over the GM value against Omicron BA.4/BA.5 at Day 57 in baseline SARS-CoV-2 negative young adults (18 to 25 years of age) post Dose 2 of 100 µg mRNA-1273 in Study P301.

Coprimary Endpoint 2: Post Dose 1 Ab GM value against the ancestral strain at Day 29 (noninferiority testing)

The null hypothesis H_0: GM value of Ab against ancestral strain at Day 29 post Dose 1 of 50 µg mRNA-1273.222 in adolescents who are baseline SARS-CoV-2 positive is inferior to GM value at Day 57 post Dose 2 of 100 µg mRNA-1273 in baseline SARS-CoV-2 negative young adults (18 to 25 years of age) in Study P301.

The noninferiority in Ab GM value against ancestral strain adolescents who are baseline SARS-CoV-2 positive compared with that in baseline SARS-CoV-2 negative young adults (18 to 25 years of age) is demonstrated by meeting the following success criterion:

- The lower bound of the 95% CI of the GMR >0.667.

The GMR is the ratio of the GM value against ancestral strain at Day 29 post Dose 1 of 50 µg mRNA-1273.222 in adolescents who are baseline SARS-CoV-2 positive in Study P203 Part 3 over the GM value against ancestral strain at Day 57 in baseline SARS-CoV-2 negative young adults (18 to 25 years of age) post Dose 2 of 100 µg mRNA-1273 in Study P301.

8.3. Power and Sample Size

8.3.1. Part 1

8.3.1.1. Part 1A

The sample size of this study is driven by safety. Approximately 3000 participants will be randomly assigned in a 2:1 ratio to receive mRNA-1273 and placebo (Section 5.2). With 2000 participants exposed to mRNA-1273, the study has at least 90% probability to observe at least 1 participant with an AE at a true 0.25% AE rate.

Serum samples from all participants will be collected and banked, a subset of participants will be selected, and their samples will be processed for immunogenicity testing (the Immunogenicity Subset).

Approximately 362 participants who receive mRNA-1273 will be selected for the Immunogenicity Subset, with a target of 289 participants receiving mRNA-1273 in the PP Immunogenicity Subset (adjusting for approximately 20% of participants who may be excluded from the PP Immunogenicity Subset, as they may not have immunogenicity results due to any reason). The sample size of the Immunogenicity Subset may be updated with data from other mRNA-1273 studies or external data especially regarding a threshold of protection. In such a situation, the final sample size of the Immunogenicity Subset will be documented in the SAP.

For the primary immunogenicity objective, with approximately 289 participants in the PP Immunogenicity Subset, the study will have > 90% power to rule out 70% with a 2-sided 95% CI for the percentage of mRNA-1273 participants exceeding the acceptable threshold if the true rate of participants exceeding the acceptable threshold is 80%.

If an acceptable Ab threshold of protection against COVID-19 is not available at the time of analysis, for the primary immunogenicity objective, noninferiority tests of 2 null hypotheses based on 2 coprimary endpoints, respectively, will be performed. The sample size calculation for each of the 2 noninferiority tests was performed, and the larger sample size was chosen for the study.

- With approximately 289 participants in the PP Immunogenicity Subset in Study P203 and 289 participants in the PP Immunogenicity Subset in young adults (18 to 25 years of age) from Study P301, there will be 90% power to demonstrate noninferiority of the immune response as measured by Ab GM value in adolescents in Study P203 at a 2-sided α of 0.05, compared with that in young adults (18 to 25 years of age) from Study P301 receiving mRNA-1273, assuming an underlying GMR value of 1 and a noninferiority margin of 1.5. The SD of the log-transformed levels is assumed to be 1.5.
- With approximately 289 participants in the PP Immunogenicity Subset in Study P203 and 289 participants in the PP Immunogenicity Subset in young adults (18 to 25 years of age) from Study P301, there will be at least 90% power to demonstrate noninferiority of the immune response as measured by the SRR in adolescents in Study P203 at a 2-sided α of 0.05, compared with that in young adults (18 to 25 years of age) from Study P301 receiving mRNA-1273, assuming a true SRR of 85% in young adults (18 to 25 years of age) from Study P301, and a true SRR of 85% in adolescents in Study P203 (ie, true rate difference is 0 compared to young adults from Study P301), and a noninferiority margin of 10%.

8.3.1.2. Part 1C

8.3.1.2.1. Part 1C-1– Homologous Booster Phase

All participants enrolled in Part 1A or Part 1B who meet the eligibility criteria for BD will be offered a BD of mRNA-1273 50 µg. With more than 1,000 participants expected to receive mRNA-1273 BD, the study Part 1C-1 has a 90% probability to observe at least 1 participant with an AE at a true AE rate of 0.25%.

Serum samples from all participants will be collected and banked, a subset of participants will be selected (as described in the SAP document version 4.0), and their samples will be processed for immunogenicity testing (the Immunogenicity Subset) at specified timepoints.

Approximately 400 participants who receive mRNA-1273 BD will be selected for the Immunogenicity Subset for Part 1C-1, with a target of 289 participants receiving mRNA-1273 BD in the PP Immunogenicity Subset with prebooster negative SARS-CoV-2 for Part 1C-1 (adjusting for approximately 25% of participants who may be excluded as they may have prebooster positive SARS-CoV-2, have no immunogenicity results due to any reason or have protocol deviations impacting critical data).

For the primary immunogenicity objective in Part 1C-1, noninferiority tests of 2 null hypotheses based on 2 coprimary endpoints, respectively, will be performed.

- With approximately 289 participants receiving mRNA-1273 BD in the PP Immunogenicity Subset with prebooster negative SARS-CoV-2 in Study P203 Part 1C-1 and 289 participants in the PP Immunogenicity Subset in young adults (18 to 25 years of age) in Study P301, there will be 90% power to demonstrate noninferiority of the immune response post BD as measured by Ab GM value in adolescents in Study P203 Part 1C-1 compared with Ab GM value in young adults (18 to 25 years of age) following primary series of mRNA-1273 in Study P301, at a 2-sided α of 0.05, assuming an underlying GMR value of 1 and a noninferiority margin of 1.5. The SD of the log-transformed levels is assumed to be 1.5.
- With approximately 289 participants receiving mRNA-1273 BD in the PP Immunogenicity Subset with prebooster negative SARS-CoV-2 in Study P203 Part 1C-1 and 289 participants in the PP Immunogenicity Subset in young adults (18 to 25 years of age) in Study P301, there will be at least 90% power to demonstrate noninferiority of the immune response post BD as measured by SRR in adolescents in Study P203 Part 1C-1 compared with SRR in young adults of (18 to 25 years of age) following primary series of mRNA-1273 in Study P301, at a 2-sided α of 0.05, assuming true SRR of 90% in young adults (18 to 25 years of age) following primary series of mRNA-1273 in Study P301, and a true SRR of 90% post BD in adolescents in Study P203 Part 1C-1 (ie, true rate difference is 0 compared to young adults [18 to 25 years of age] from Study P301), and a noninferiority margin of 10%.

8.3.1.2.2. Part 1C-2 – Heterologous Booster Phase

Approximately 362 participants who received non-Moderna COVID-19 vaccine as primary series vaccination at least 3 months prior were planned to be enrolled to receive mRNA-1273 50 µg as heterologous booster in Part 1C-2. However, the study population in Part 1C-2 will have less than the planned number of participants enrolled, as a result of slow enrollment and discontinuation of recruitment in Part 1C-2 (Section 3.1.1.4).

8.3.2. Part 2

Approximately 362 participants were planned to be enrolled to receive mRNA-1273 50 µg in the open-label Part 2. However, the study population in Part 2 will have less than the planned

number of participants receiving at least one dose of 50 µg mRNA-1273, with a small sample size as a result of slow enrollment and discontinuation of recruitment in Part 2 (Section 3.1.2).

8.3.3. Part 3

With at least 300 adolescents enrolled in Part 3 to receive 50 µg mRNA-1273.222 primary series, the study will have at least a 95% probability to observe at least 1 participant with an AE at a true AE rate of 1%.

Assuming a true serum Ab GMR of 1.6 for GM value against Omicron BA.4/BA.5 at Day 29 after Dose 1 of mRNA-1273.222 in baseline SARS-CoV-2 positive adolescents compared with GM value against Omicron BA.4/BA.5 at Day 57 after Dose 2 of mRNA-1273 primary series in young adults who are baseline SARS-CoV-2 negative in P301, with approximately 168 participants in the PP Immunogenicity Subset who are baseline SARS-CoV-2 positive in P203 Part 3 and 300 young adults in P301 who are baseline SARS-CoV-2 negative, there will be at least >90% power to demonstrate superiority of the serum Ab GM value against Omicron BA.4/BA.5 after Dose 1 of mRNA-1273.222 in adolescents in P203 to that after Dose 2 of mRNA-1273 in young adults in Study P301 at two-sided α of 0.05 using a superiority margin of 1.0. The standard deviation of the natural log transformed levels of Ab is assumed to be 1.5.

Assuming a true serum Ab GMR of 1.1 for GM value against ancestral strain at Day 29 after Dose 1 of mRNA-1273.222 in baseline SARS-CoV-2 positive adolescents compared with GM value against ancestral strain at Day 57 after Dose 2 of mRNA-1273 primary series in young adults who are baseline SARS-CoV-2 negative in P301, this sample size will also provide at least >90% power to demonstrate noninferiority of GM value of Ab against ancestral strain at Day 29 post Dose 1 of mRNA-1273.222 in adolescents in P203 to that after Dose 2 of mRNA-1273 in young adults in Study P301 at two-sided α of 0.05 using a noninferiority margin of 1.5. The standard deviation of the natural log transformed levels of Ab is assumed to be 1.5.

With approximately 30% of participants in Part 3 who may be excluded from the PP Immunogenicity Subset-baseline SARS-CoV-2 positive (PPIS-POS), as they may be baseline SARS-CoV-2 negative or have missing immunogenicity results due to any reason or protocol deviations impacting critical data, approximately 240 participants are required in Part 3 to provide 168 participants in the PP Immunogenicity Subset-baseline SARS-CoV-2 positive.

8.4. Analysis Sets

The analysis sets are defined in Table 7.

Table 7: Analysis Sets

Analysis Set	Description
Randomization Set	All participants who are randomized, regardless of the participants' treatment status in the study.
FAS	All randomized participants who received at least 1 injection of IP.
Immunogenicity Subset	A subset of participants in the FAS will be selected for immunogenicity testing.
PP Immunogenicity Subset	A subset of participants in the FAS will be selected for immunogenicity testing. The PP Immunogenicity Subset includes participants selected for the Immunogenicity Subset who received planned doses of study vaccination per

Analysis Set	Description
	schedule, complied with immunogenicity testing schedule, and have no major protocol deviations that impact key or critical data. Participants who are seropositive at baseline will be excluded from the PP Immunogenicity Subset. The PP Immunogenicity Subset will be used for analyses of immunogenicity unless specified otherwise.
PP Set for Efficacy	All participants in the FAS who received planned doses of study vaccination, had no immunologic or virologic evidence of prior COVID-19, and have no major protocol deviations that impact key or critical efficacy data.
Solicited Safety Set	The Solicited Safety Set consists of FAS participants who contributed any solicited AR data.<br>The Solicited Safety Set will be used for the analyses of solicited ARs.
Safety Set	All randomized participants who receive at least 1 dose of IP. The Safety Set will be used for all analyses of safety except for the solicited ARs.
mITT Set	All participants in the FAS who have no serologic or virologic evidence of prior SARS-CoV-2 infection before the first dose of IP (both negative RT-PCR test for SARS-CoV-2 and negative serology test based on bAb specific to SARS-CoV-2 nucleocapsid) at baseline.
mITT1 Set	All participants in the mITT Set excluding those who received the wrong treatment (ie, at least 1 dose received is not as randomized or planned).
PP Immunogenicity Subset for BD phase	The PP Immunogenicity Subset for BD phase includes participants who received planned BD per schedule, complied with immunogenicity testing schedule, and have no major protocol deviations that impact key or critical data, with prebooster negative or positive SARS-CoV-2. The PP Immunogenicity Subset with prebooster negative SARS-CoV-2 will be used for the primary immunogenicity analysis.
PP Immunogenicity Subset for Part 3	The PP Immunogenicity Subset for Part 3 includes participants who received planned dose per schedule, complied with immunogenicity testing schedule, and have no major protocol deviations that impact key or critical data, with baseline negative or positive SARS-CoV-2. The PP Immunogenicity Subset for Part 3 with baseline-positive SARS-CoV-2 status will serve as the population for the primary and secondary analysis of immunogenicity data at Day 29 in Part 3.<br>The PP Immunogenicity Subset (Part 3, at Day 209) will serve as the population for the primary and secondary analysis of immunogenicity data at Day 209 in Part 3.

Abbreviations: AR = adverse reaction; bAb = binding antibody; COVID-19 = coronavirus disease 2019; FAS = full analysis set; IP = investigational product; mITT = modified intent-to-treat; PP = per-protocol; RT-PCR = reverse transcription polymerase chain reaction; SARS-CoV-2 = Severe Acute Respiratory Syndrome coronavirus 2.

Note: Positive SARS-CoV-2 status at baseline is defined as a positive RT-PCR test for SARS-CoV-2, and/or a positive serology test based on bAb specific to SARS-CoV-2 nucleocapsid (as measured by Roche Elecsys Anti-SARS-CoV-2 assay) on or before Day 1. Negative status is defined as a negative RT-PCR test for SARS-CoV-2, and a negative serology test based on bAb specific to SARS-CoV-2 nucleocapsid (as measured by Roche Elecsys Anti-SARS-CoV-2 assay) on or before Day 1.

8.5. Statistical Methods

Data from Part 1A, Part 1B, and Part 1C will be presented separately as applicable.

8.5.1. Baseline Characteristics and Demographics

Demographic variables (eg, age, race, sex, height, weight, and BMI) and baseline characteristics will be summarized by treatment group. Summary statistics (mean, SD for continuous variables, and number and percentage for categorical variables) will be provided.

8.5.2. Safety Analyses

All safety analyses will be based on the Safety Set, except summaries of solicited ARs, which will be based on the Solicited Safety Set. All safety analyses will be provided by treatment group.

Safety and reactogenicity will be assessed by clinical review of all relevant parameters including solicited ARs (local and systemic events), unsolicited AEs, SAEs, MAAEs, AESI, AEs leading to discontinuation, vital sign measurements, and physical examination findings.

The number and percentage of participants with any solicited local AR, with any solicited systemic AR, and with any solicited AR during the 7-day follow-up period after each injection (except Part 1B) will be summarized.

The number and percentage of participants with any solicited local AR, with any solicited systemic AR, and with any solicited AR during the 7-day follow-up period after each dose (except Part 1B) will be provided. A 2-sided 95% exact CI using the Clopper-Pearson method will also be provided for the percentage of participants with any solicited AR.

The number and percentage of participants with unsolicited AEs, SAEs, MAAEs, Grade 3 or higher ARs and AEs, and AEs leading to discontinuation from IP or withdrawal from the study will be summarized. Unsolicited AEs will be presented by MedDRA preferred term and system organ class.

The number of events of solicited ARs, unsolicited AEs/SAEs, and MAAEs will be reported in summary tables accordingly.

For all other safety parameters, descriptive summary statistics will be provided, and Table 8 summarizes analysis strategy for safety parameters. Further details will be described in the SAP.

Table 8: Analysis Strategy for Safety Parameters

Safety Endpoint	Number and Percentage of Participants, Number of Events	95% CI
Any solicited AR (overall and by local, systemic)	X	X
Any unsolicited AE	X	—
Any SAE	X	—
Any AESI	X	—
Any unsolicited MAAE	X	—
Any unsolicited treatment-related AE	X	—

Safety Endpoint	Number and Percentage of Participants, Number of Events	95% CI
Any treatment-related SAE	X	—
Discontinuation from dosing/withdrawal from study participation due to AE	X	—
Any severe AE	X	—
Any treatment-related severe AE	X	—

Abbreviations: AE = adverse event; AESI = adverse event of special interest; AR = adverse reaction; CI = confidence interval; MAAE = medically attended adverse event; PT = preferred term; SAE = serious adverse event; SOC = system organ class.

Notes: 95% CI using the Clopper-Pearson method, X = results will be provided. Unsolicited AEs will be summarized by SOC and PT coded by MedDRA.

8.5.3. Immunogenicity Analyses in Part 1

The SAP will describe the complete set of immunogenicity analyses, including the approach to sample participants into an Immunogenicity Subset for analysis of immunogenicity. The PP Immunogenicity Subset is the primary analysis set for immunogenicity unless otherwise specified. The primary immunogenicity objective of this study is to use the immunogenicity response to infer efficacy in adolescents (12 to <18 years in this study). The primary immunogenicity analysis is performed in Part 1A.

If an accepted serum Ab threshold of protection against COVID-19 is available based on data from other mRNA-1273 studies or external data, the number and percentage of participants with Ab greater than or equal to the threshold at Day 57 will be provided with a 2-sided 95% CI using the Clopper-Pearson method. If the lower bound of the 95% CI on the mRNA-1273 group is >70%, the primary immunogenicity objective of this study will be considered to be met.

The percentage of participants with serum Ab greater than or equal to the threshold with 95% CI will be provided at each postbaseline timepoint. The CI will be calculated using the Clopper-Pearson method.

If an accepted serum Ab threshold of protection against COVID-19 is not established, the noninferiority of primary vaccine response as measured by Ab GM value and SRR in adolescents compared with those in young adults (18 to 25 years of age) receiving mRNA-1273 will be assessed. The study is considered as meeting the primary immunogenicity objective if the noninferiority of the immune response to mRNA-1273 as measured by both GM value and SRR at Day 57 is demonstrated in adolescents in this study at a 2-sided α of 0.05, compared with that in young adults (18 to 25 years of age) in Study P301 receiving mRNA-1273.

An ANCOVA model will be carried out with Ab value at Day 57 as a dependent variable and a group variable (adolescents in Study P203 and young adults in Study P301) as the fixed variable. The GM values of the adolescents at Day 57 will be estimated by the GLSM from the model. The GMR (ratio of GM values) will be estimated by the ratio of GLSM from the model. A corresponding 2-sided 95% CI will be provided to assess the difference in immune response for the adolescents in Study P203 compared to the young adults (18 to 25 years of age) in Study P301 at Day 57. The noninferiority of immune response to mRNA-1273 as measured by GM value will be considered demonstrated if the lower bound of the 95% CI of the GMR is >0.667 based on the noninferiority margin of 1.5.

The number and percentage (rate) of participants achieving Ab seroresponse at Day 57 will be summarized. The difference of SRRs between adolescents receiving mRNA-1273 in Study P203 and young adults (18 to 25 years of age) receiving mRNA-1273 in Study P301 will be calculated with 95% CI. The noninferiority in SRR of adolescents in Study P203 compared to young adults (18 to 25 years of age) in Study P301 will be considered demonstrated if the lower bound of the 95% CI of the SRR difference is >-10%, based on the noninferiority margin of 10%.

In addition, the GM level of specific nAb and bAb with corresponding 95% CI will be provided at each timepoint. The 95% CIs will be calculated based on the t-distribution of the log transformed values then back transformed to the original scale. The GMFR of nAb and bAb with corresponding 95% CI will be provided at each timepoint with Day 57 as the primary timepoint of interest. Descriptive summary statistics including median, minimum, and maximum will also be provided.

8.5.4. Efficacy Analyses

To evaluate the incidence of COVID-19 after vaccination with mRNA-1273 or placebo, the incidence rate will be provided by vaccination group, calculated as the number of cases divided by the total person-time. The incidence rate ratio of mRNA-1273 versus placebo may be provided with its 95% CI computed using the exact method conditional upon the total number of cases adjusted by the total person-time.

For SARS-CoV-2 infection (serologically confirmed SARS-CoV-2 infection or COVID-19), regardless of symptomatology or severity, infection rate will be provided by vaccination group. The infection rate ratio of mRNA-1273 versus placebo may be provided with its 95% CI using the exact method conditional upon the total number of cases adjusted by the total person-time. The incidence rate of asymptomatic SARS-CoV-2 infection will also be provided.

The secondary efficacy analyses will be performed in the PP set, with sensitivity analyses in the FAS, mITT Set, and mITT1 Set.

8.5.5. Long-term Analysis (Including Part 1B)

Long-term analysis will be performed including data collected in the Open-label Observational Phase (Part 1B), and prior to BD if a BD is received. The analysis will include participants who are randomized to mRNA-1273 in Part 1A and remained in the study with or without unblinding, and participants who are originally randomized to placebo and later crossed over to receive mRNA-1273 in Part 1B after unblinding. Long-term analysis of applicable safety, efficacy, and immunogenicity endpoints will be summarized descriptively by treatment cohort as defined in Table 9 without treatment group comparison.

In the long-term safety analysis, unsolicited AEs (SAE, AESI, MAAE, and AE leading to discontinuation) and deaths will be summarized.

In the long-term immunogenicity analysis, nAb and bAb values will be summarized at specified timepoints.

In the long-term efficacy analysis, the incidence rates of COVID-19 and of SARS-CoV-2 infection cases will be counted starting 14 days after the second dose of IP for participants in treatment cohort of mRNA-1273 or starting 14 days after the second dose of mRNA-1273 for participants in the Placebo-mRNA-1273 cohort. Incidence rate with 95% CI adjusting for

person-time will be provided. The incidence rate of asymptomatic SARS-CoV-2 infection will also be provided.

Table 9: Treatment Cohorts for the Long-term Analysis

Long-term Treatment Cohort	Description
**mRNA-1273**	Participants randomized to mRNA-1273 in the Blinded Phase
**Placebo-mRNA-1273**	Participants randomized to Placebo in the Blinded Phase who crossed over to mRNA-1273 in the Open-label Observational Phase

8.5.6. Booster Phase Analysis

8.5.6.1. Part 1C-1 – Homologous Booster Phase

Booster Phase analysis will be performed in participants who receive a BD in Part 1C-1. Safety, immunogenicity endpoints, and incidence of COVID-19 and SARS-CoV-2 infection will be summarized descriptively, and by prebooster SARS-CoV-2 status if applicable.

In the Booster Phase safety analysis, the number and percentage of participants with unsolicited AEs, severe AEs, treatment-related AEs, SAEs, MAAEs, AESIs, and AEs leading to discontinuation from the study participation in Part 1C-1 will be summarized.

The number and percentage of participants with any solicited local AR, with any solicited systemic AR, and with any solicited AR during the 7-day follow-up period after the BD will be provided.

In the Part 1C-1 Booster Phase, for the coprimary endpoints (Ab GM value and SRR against the ancestral strain) and key secondary endpoints (Ab GM value and SRR against the circulating strain), the following analyses will be performed for the respective hypothesis testing:

- The GM titers with 95% CI will be summarized using t-distribution of the log transferred values and then back transformed to the original scale. The GMR with 95% CI to compare postbooster GM value at BD-Day 29 in adolescents in Study P203 with the primary series GM value at Day 57 (28 days after Dose 2) in young adults (18 to 25 years of age) in Study P301 will be computed based on the t-distribution of mean difference in the log transferred values and then back transformed to the original scale.
- The SRR with 95% CI (using Clopper-Pearson method) will be summarized. The SRR difference with 95% CI (using Miettinen-Nurminen score method) to compare postbooster SRR at BD-Day 29 in adolescents in Study P203 with the primary series SRR at Day 57 (28 days after Dose 2) in young adults in Study P301 will be computed. Seroresponse is defined as Ab value change from baseline (pre Dose 1) below the LLOQ to ≥4 × LLOQ, or at least a 4-fold rise if baseline is ≥LLOQ.

In the Booster Phase immunogenicity analyses, the GM titers or levels for specific nAb and bAb, with corresponding 95% CI will be provided at BD-Day 1, BD-Day 29, BD-Day 181, and BD-Day 361 (Part 1C-1). GMFR relative to pre Dose 1, (Homologous Booster) BD-Day 1 and prior Day 57 in the phase of primary 2-dose series of mRNA-1273 will be summarized with 95% CIs at BD-Day 29, BD-Day 181, and BD-Day 361 (Part 1C-1). The 95% CIs will be calculated based on the t-distribution of the log transformed values then back transformed to the original

scale. Descriptive summary statistics including median, minimum, and maximum will also be provided. The immunogenicity descriptive summaries will be provided in the participants who have available nAb and bAb assay results for the specified timepoints.

In the Booster Phase, the incidence rate of the first occurrence of symptomatic COVID-19 starting 14 days after the BD will be summarized by prebooster SARS-CoV-2 status. The incidence rate of SARS-CoV-2 infection (symptomatic or asymptomatic infection) starting 14 days after the BD, and the incidence rate of asymptomatic SARS-CoV-2 infection starting 14 days after the BD will be summarized. The incidence rates with 95% CI will be computed adjusting for person-years starting from BD-Day 1 in Part 1C-1.

8.5.6.2. Part 1C-2 – Heterologous Booster Phase Analysis

Heterologous Booster Phase analysis will be performed in participants who receive mRNA-1273 50 µg in Part 1C-2. Safety, immunogenicity endpoints, and incidence of COVID-19 and SARS-CoV-2 infection will be summarized descriptively, and by prebooster SARS-CoV-2 status if applicable.

In the Heterologous Booster Phase safety analysis, the number and percentage of participants with unsolicited AEs, severe AEs, treatment-related AEs, SAEs, MAAEs, AESIs, and AEs leading to discontinuation from the study participation in Part 1C-2 will be summarized.

The number and percentage of participants with any solicited local AR, with any solicited systemic AR, and with any solicited AR during the 7-day follow-up period after the BD will be provided.

In the Heterologous Booster Phase immunogenicity analyses, the GM titers or levels for specific nAb and bAb (against the ancestral strain or against specific circulating variants of concern), with corresponding 95% CI will be provided at BD-Day 1, BD-Day 29, BD-Day 181, and BD-Day 361. Geometric mean fold rise relative to BD-Day 1 will be summarized with 95% CIs at BD-Day 29, BD-Day 181, and BD-Day 361. The 95% CIs will be calculated based on the t-distribution of the log transformed values then back transformed to the original scale. Descriptive summary statistics including median, minimum, and maximum will also be provided for GM value and GMFR. The SRR and category of at least 2-fold rise relative to BD-Day 1 will be summarized with numbers and percentages of participants. The immunogenicity descriptive summaries will be provided in the PP Immunogenicity Subset who have available nAb and bAb assay results for the specified timepoints.

In the Heterologous Booster Phase, the incidence rate of the first occurrence of symptomatic COVID-19 starting 14 days after the BD will be summarized by prebooster SARS-CoV-2 status. The incidence rate of SARS-CoV-2 infection (symptomatic or asymptomatic infection) starting 14 days after the BD, and the incidence rate of asymptomatic SARS-CoV-2 infection starting 14 days after the BD will be summarized. The incidence rates with 95% CI will be computed adjusting for person-years starting from BD-Day 1 in Part 1C-2.

8.5.7. Part 2 Analysis

Part 2 safety and immunogenicity endpoints will be summarized descriptively.

In the safety analysis, the number and percentage of participants with unsolicited AEs, severe AEs, treatment-related AEs, SAEs, MAAEs, AESIs, and AEs leading to discontinuation from the study participation in Part 2 will be summarized.

The number and percentage of participants with any solicited local AR, with any solicited systemic AR, and with any solicited AR during the 7-day follow-up period after each injection will be provided.

In the immunogenicity descriptive analyses, the GM value for specific Ab (against the ancestral strain or against specific circulating variants of concern) with corresponding 95% CI, and GMFR from baseline with 95% CI will be provided by timepoint. GM and GMFR 95% CIs will be calculated based on the t-distribution of the log transformed values then back transformed to the original scale. Descriptive summary statistics including median, minimum, and maximum will also be provided for GM and GMFR. The SRR and category of at least 2-fold rise from baseline by timepoint will be summarized with numbers and percentages of participants. The immunogenicity descriptive summaries will be provided in the PP Immunogenicity Subset who have available nAb and bAb assay results for the specified timepoints.

8.5.8. Part 3 Analysis

In the safety analysis, the number and percentage of participants with unsolicited AEs, severe AEs, treatment-related AEs, SAEs, MAAEs, AESIs, and AEs leading to discontinuation from the study participation in Part 3 will be summarized.

The number and percentage of participants with any solicited local AR, with any solicited systemic AR, and with any solicited AR during the 7-day follow-up period after each injection will be provided.

In the Part 3 immunogenicity analysis, for the coprimary endpoints (Ab GM value against Omicron BA.4/BA.5 and the ancestral strain at Day 29 after Dose 1 of 50 µg mRNA-1273.222 in baseline SARS-CoV-2 positive adolescents in Study P203 compared with those at Day 57 after Dose 2 of 100 µg mRNA-1273 in baseline SARS-CoV-2 negative adults 18 to 25 years of age in Study P301), the following analyses will be performed for the respective hypothesis testing:

An ANCOVA model will be carried out with Ab value at Day 29 in P203 and Ab value at Day 57 in P301 as a dependent variable and a group variable (baseline SARS-CoV-2 positive adolescents in Study P203 and baseline negative SARS-CoV-2 young adults in Study P301) as the fixed variable, where a covariate may be included in the model (to be specified in the SAP). The GM value at Day 29 in P203 and the GM value at Day 57 in P301, and respective GMR for P203 compared to P301 will be estimated from the model. A corresponding 2-sided 95% CI of GMR estimated from the ANCOVA model will be provided to assess the difference in immune response after Dose 1 (Day 29) for the adolescents in Study P203 compared to that after Dose 2 (Day 57) in the young adults (18 to 25 years of age) in Study P301.

The first coprimary endpoint, superiority of immune response to mRNA-1273.222 against Omicron BA.4/BA.5 after Dose 1 compared to mRNA-1273 against Omicron BA.4/BA.5 after Dose 2 as measured by GM value will be considered demonstrated if the respective GMR 95% CI lower bound is >1 based on a superiority margin of 1.

The second coprimary endpoint, noninferiority of immune response to mRNA-1273.222 against ancestral strain after Dose 1 compared to mRNA-1273 against the ancestral strain after Dose 2 as

measured by GM value will be considered demonstrated if the respective GMR 95% CI lower bound is >0.667 based on the noninferiority margin of 1.5. The GM value and GMR with specified CIs will also be summarized using t-distribution of the log transferred values and then back transformed to the original scale, and by baseline SARS-CoV-2 status.

For secondary immunogenicity endpoints (Ab GM values after Dose 1 or Dose 2 in adolescents compared with those after Dose 2 in adults in P301), descriptive summaries will be provided in the participants who have available nAb and bAb assay results for the specified timepoints. Secondary endpoints evaluating SRR with 95% CIs (using Clopper-Pearson method) will be summarized by baseline SARS-CoV-2 status at Day 29 and Day 209 in adolescents in Study P203, and at Day 57 in adults in Study P301. Seroresponse at subject level is defined as Ab value change from baseline (preDose 1) below the LLOQ to ≥4 × LLOQ, or at least a 4-fold rise if baseline is ≥LLOQ. The SRR and category of at least 2-fold rise from baseline by timepoint will be summarized with numbers and percentages of participants.

The GM values for specific nAb and bAb, with corresponding 95% CIs, and GMFR from baseline with 95% CIs will be summarized by timepoint. The 95% CIs will be calculated based on the t-distribution of the log transformed values then back transformed to the original scale. Descriptive summary statistics including median, minimum, and maximum will also be provided.

The incidence rate of the first occurrence of symptomatic COVID-19, 14 days after Dose 1, and 14 days after Dose 2, will be summarized. The incidence rates with 95% CIs will be computed adjusting for person-years.

8.5.9. Exploratory Analyses

Exploratory analyses will be described in the SAP before database lock.

8.5.10. Subgroup Analyses

Subgroup analyses will be performed as described in the SAP.

8.6. Multiplicity Adjustment

A hierarchical sequential hypothesis testing (fixed-sequence) method will be used to adjust multiplicity to preserve the family-wise type I error rate (α = 0.05). The hypothesis testing for the 2 coprimary endpoints (GMT and SRR) for the primary series of mRNA-1273 in Part 1A was completed and statistically significant based on data snapshot dated 08 May 2021, and thus the α level of 0.05 can be passed to Part 1C-1 hypothesis testing. In Part 1C-1 Homologous Booster Phase, the hypothesis testing for the 2 coprimary endpoints (GMT and SRR against the ancestral strain) after BD of mRNA-1273 will be tested at α level of 0.05. The testing in Part 1A and 1C-1 will continue through the sequence only until an endpoint is not statistically significant (did not meet specified noninferiority success criteria), in which case the testing will stop. If the hypothesis testing for the 2 coprimary endpoints in Part 1C-1-Homologous Booster Phase is statistically significant (meeting the noninferiority success criteria of the coprimary endpoints), the α level of 0.05 will be passed to the hypothesis testing in Part 3.

For the key secondary objective in Part 1C-1 Homologous Booster Phase to evaluate immune response elicited by the 50 µg prototype booster of mRNA-1273 against variant(s) of interest, the

key secondary endpoints will be analyzed independently, given that prototype vaccine mRNA-1273 does not contain variant specific sequences.

Part 3

Since the hypothesis testing for the coprimary endpoints in Part 1C-1 was statistically significant based on an IA with a data cutoff dated 16 May 2022, the α level of 0.05 (two-sided) was passed to Part 3 hypothesis testing.

8.7. Study Analyses

8.7.1. Interim Analyses

More than one IA may be performed.

- The IA of immunogenicity, safety, and efficacy will be performed after Day 57 immunogenicity data are available for the Immunogenicity Subset and at least 1500 participants (1,000 participants receiving mRNA-1273) have completed Day 57 (1 month after Dose 2, Part 1A). This IA will be considered the primary analysis of immunogenicity for Part 1A.
- An IA of immunogenicity and safety may be performed after all or subset of participants who receive BD have completed BD-Day 29 after the BD in Part 1C-1.
- An IA of immunogenicity and safety may be performed after all or subset of participants who receive heterologous BD have completed BD-Day 29 after the BD in Part 1C-2.
- An IA of immunogenicity and safety may be performed after Day 57 immunogenicity data are available after all or a subset of participants in Part 2 have completed Day 57 (1 month after Dose 2, Part 2).
- An IA of immunogenicity and safety may be performed after all or a subset of participants who receive 50 µg mRNA-1273.222 dose have completed Day 29 (1 month after Dose 1) and an additional IA may be performed in all or a subset of participants who completed Day 209 (1 month after Dose 2) in Part 3.
- At the Sponsor’s discretion, a CSR may be developed for an IA.

8.7.2. Final Analysis

The final analysis of all applicable endpoints will be performed after all participants have completed all planned study procedures. Results of this analysis will be presented in a final CSR, including individual listings.

Additional information about all study analyses may be provided in the SAP.

9. REFERENCES

Abdullah F, Myers J, Basu D, Tintinger G, Ueckermann V, Mathebula M, et al. Decreased severity of disease during the first global omicron variant covid-19 outbreak in a large hospital in tshwane, south africa. Int J Infect Dis. 2022;116:38-42.

Adler Y, Charron P, Imazio M, Badano L, Barón-Esquivias G, Bogaert J, et al. 2015 ESC guidelines for the diagnosis and management of pericardial diseases: the task force for the diagnosis and management of pericardial diseases of the european society of cardiology (ESC) endorsed by: The European Association for Cardio-Thoracic Surgery (EACTS). Eur Heart J. 2015;36(42):2921-64.

Aretz HT. Myocarditis: the Dallas criteria. Hum Pathol. 1987;18(6):619-24.

Atmar RL, Lyke KE, Deming ME, Jackson LA, Branche AR, El Sahly HM et al. Heterologous SARS-CoV-2 Booster Vaccinations – Preliminary Report. medRxiv 2021.

Boehmer TK, DeVies J, Caruso E, van Santen KL, Tang S, Black CL, et al. Changing age distribution of the COVID-19 pandemic — United States, May-August 2020. MMWR Morb Mortal Wkly Rep. 2020;69(39):1404-9.

Bretz F, Maurer W, Brannath W, Posch M. A graphical approach to sequentially rejective multiple test procedures. Stat Med. 2009;28(4):586-604.

Buonsenso D, Munblit D, De Rose C, Sinatti D, Ricchiuto A, Carfi A, et al. Preliminary evidence on long COVID in children. Acta Paediatr. 2021;110(7):2208-11.

Buonsenso D, Di Gennaro L, De Rose C, Morello R, D'Ilario F, Zampino G, et al. Long-term outcomes of pediatric infections: from traditional infectious diseases to long Covid. Future Microbiol. 2022;17:551-71.

Centers for Disease Control and Prevention (CDC). 2022a. COVID Data Tracker [database on the Internet]. Atlanta (GA): US Department of Health and Human Services, CDC. c2022 – [cited 2022 Sep 6]. Available from: https://covid.cdc.gov/covid-data-tracker/#demographicsovertime.

Centers for Disease Control and Prevention (CDC). 2022b. COVID Data Tracker [database on the Internet]. Atlanta (GA): US Department of Health and Human Services, CDC. c2022 – [cited 2022 Sep 6]. Retrieved from: https://covid.cdc.gov/covid-data-tracker/#covidnet-hospitalization-network.

Centers for Disease Control and Prevention (CDC). 2022c. COVID Data Tracker [database on the Internet]. Atlanta (GA): US Department of Health and Human Services, CDC. c2022 – [cited 2022 Sep 6]. Available from: https://covid.cdc.gov/covid-data-tracker/#new-hospital-admissions

Centers for Disease Control and Prevention (CDC). 2022d. COVID Data Tracker [database on the Internet]. Atlanta (GA): US Department of Health and Human Services, CDC. c2022 – [cited 2022 Sep 6]. Available from: https://covid.cdc.gov/covid-data-tracker/#mis-national-surveillance.

Chalkias S, Eder F, Essink B, Khetan S, Nestorova B, Feng J, et al. Safety, immunogenicity and antibody persistence of a bivalent Beta-containing booster vaccine against COVID-19: a phase 2/3 trial. Nat Med. 2022a.

Chalkias S, Harper C, Vrbicky K, Walsh SR, Essink B, Brosz A, et al. A Bivalent Omicron-containing Booster Vaccine Against Covid-19. N Engl J Med. 2022b;387(14):1279-91.

Chalkias S, Whatley J, Eder F, Essink B, Khetan S, Bradley P, et al. Safety and Immunogenicity of Omicron BA.4/BA.5 Bivalent Vaccine Against Covid-19. medRxiv. 2022c.

Chen Y, Lu S, Jia H, Deng Y, Zhou J, Huang B, et al. A novel neutralizing monoclonal antibody targeting the N-terminal domain of the MERS-CoV spike protein. Emerg Microbes Infect. 2017;6(5):e37.

Chin J, Magoffin RL, Shearer LA, Schieble JH, Lennette EH. Field evaluation of a respiratory syncytial virus vaccine and a trivalent parainfluenza virus vaccine in a pediatric population. Am J Epidemiol. 1969;89(4):449-63.

Clarke KE, Jones JM, Deng Y, Nycz E, Lee A, Lachan R, et al. Seroprevalence of Infection-Induced SARS-CoV-2 Antibodies — United States, September 2021–February 2022. MMWR Morb Mortal Wkly Rep 2022;71:606-8.

ClinicalTrials.gov [Internet]. Bethesda (MD): National Library of Medicine (US). - Identifier NCT04283461, Safety and immunologic protocol of SARS-CoV-2 vaccine (mRNA-1273) to treat novel coronavirus; 2020 Feb 22. Retrieved from: https://clinicaltrials.gov/ct2/keydates/NCT04283461. (accessed 2020 Jun 01).

ClinicalTrials.gov [Internet]. Bethesda (MD): National Library of Medicine (US). - Identifier NCT04405076, Dose-Confirmation Study to Evaluate the Safety, Reactogenicity, and Immunogenicity of mRNA-1273 COVID-19 Vaccine in Adults Aged 18 Years and Older; 2020 May 28. Retrieved from: https://clinicaltrials.gov/ct2/keydates/NCT04405076. (accessed 2020 Aug 24).

ClinicalTrials.gov [Internet]. Bethesda (MD): National Library of Medicine (US). - Identifier NCT04470427, A Study to Evaluate Efficacy, Safety, and Immunogenicity of mRNA-1273 Vaccine in Adults Aged 18 Years and Older to Prevent COVID-19; 2020 Jul 14. Retrieved from: https://clinicaltrials.gov/ct2/keydates/NCT04470427. (accessed 2020 Aug 24).

ClinicalTrials.gov [Internet]. Bethesda (MD): National Library of Medicine (US). – Identifier NCT04649151, A Study to Evaluate the Safety, Reactogenicity, and Effectiveness of mRNA-1273 Vaccine in Adolescents 12 to <18 Years Old to Prevent COVID-19 (TeenCove); 2020 Dec 2. Retrieved from: https://clinicaltrials.gov/ct2/show/NCT04649151?term=NCT04649151. (accessed 2022 August 30).

ClinicalTrials.gov [Internet]. Bethesda (MD): National Library of Medicine (US). – Identifier NCT04785144, Safety and Immunogenicity Study of a SARS-CoV-2 (COVID-19) Variant Vaccine (mRNA-1273.351) in Naïve and Previously Vaccinated Adults; 2021 Mar 05. Retrieved from: https://clinicaltrials.gov/ct2/show/NCT04785144. (accessed 2022 Aug 30).

ClinicalTrials.gov [Internet]. Bethesda (MD): National Library of Medicine (US). – Identifier NCT04796896, A Study to Evaluate Safety and Effectiveness of mRNA-1273 COVID-19 Vaccine in Healthy Children Between 6 Months of Age and Less Than 12 Years of Age; 2021 Mar 15. Retrieved from: https://clinicaltrials.gov/ct2/show/NCT04796896. (accessed 2022 August 30).

ClinicalTrials.gov [Internet]. Bethesda (MD): National Library of Medicine (US). – Identifier NCT04860297, A Study to Evaluate Safety and Immunogenicity of mRNA-1273 Vaccine to Prevent COVID-19 in Adult Organ Transplant Recipients and in Healthy Adult Participants; 2021 Apr 26. Retrieved from: https://clinicaltrials.gov/ct2/show/NCT04860297. (accessed 2022 Aug 30).

ClinicalTrials.gov [Internet]. Bethesda (MD): National Library of Medicine (US). – Identifier NCT04860297, A Study to Evaluate the Immunogenicity and Safety of mRNA Vaccine Boosters for SARS-CoV-2 (COVID-19) Variants; 2021 Jun 15. Retrieved from: https://clinicaltrials.gov/ct2/show/NCT04727065. (accessed 2022 11 Oct).

ClinicalTrials.gov [Internet]. Bethesda (MD): National Library of Medicine (US). – Identifier NCT05249829, A Study to Evaluate the Immunogenicity and Safety of Omicron Variant Vaccines in Comparison With mRNA-1273 Booster Vaccine for COVID-19; 2022 Feb 22. Retrieved from: https://clinicaltrials.gov/ct2/show/NCT04727065. (accessed 2022 11 Oct).

ClinicalTrials.gov [Internet]. Bethesda (MD): National Library of Medicine (US). – Identifier NCT05436834, A Study to Evaluate the Safety and Immunogenicity of the mRNA-1273.214 COVID-19 Vaccine in Healthy Children Between 6 Months to Less Than 6 Years of Age; 2022 Jun 29. Retrieved from: https://clinicaltrials.gov/ct2/show/NCT05436834. (accessed 2022 11 Oct).

ClinicalTrials.gov [Internet]. Bethesda (MD): National Library of Medicine (US). – Identifier NCT05584202, Study to Evaluate the Safety, Reactogenicity, and Effectiveness of mRNA-1273.214 SARS-CoV-2 (COVID-19) Vaccine in Infants (BabyCOVE); 2023 May 22. Retrieved from: https://clinicaltrials.gov/ct2/show/NCT05584202. (accessed 2023 09 Jun).

Corbett KS, Edwards DK, Leist SR, Abiona OM, Boyoglu-Barnum S, Gillespie RA, et al. SARS-CoV-2 mRNA vaccine development enabled by prototype pathogen preparedness. Nature. 2020a;586(7830):567-71.

Corbett KS, Flynn B, Foulds KE, Francica JR, Boyoglu-Barnum S, Werner AP, et al. Evaluation of the mRNA-1273 vaccine against SARS-CoV-2 in nonhuman primates. N Engl J Med. 2020b;383(16):1544-55.

Corti D, Zhao J, Pedotti M, Simonelli L, Agnihothram S, Fett C, et al. Prophylactic and postexposure efficacy of a potent human monoclonal antibody against MERS coronavirus. Proc Natl Acad Sci U S A. 2015;112(33):10473-8.

Delahoy MJ, Ujamaa D, Whitaker M, O'Halloran A, Anglin O, Burns E, et al. COVID-NET Surveillance Team; COVID-NET Surveillance Team. Hospitalizations Associated with COVID-19 Among Children and Adolescents - COVID-NET, 14 States, March 1, 2020-August 14, 2021. MMWR Morb Mortal Wkly Rep. 2021;70(36):1255-60.

Dembiński Ł, Vieira Martins M, Huss G, Grossman Z, Barak S, Magendie C, et al. SARS-CoV-2 Vaccination in Children and Adolescents-A Joint Statement of the European Academy of Paediatrics and the European Confederation for Primary Care Paediatricians. Front Pediatr. 2021;9:721257.

Department of Health and Human Services (DHHS), Food and Drug Administration, Center for Biologics Evaluation and Research (US). Guidance for industry: Toxicity grading scale for healthy adult and adolescent volunteers enrolled in preventative vaccine clinical trials.

September 2007 [cited 2019 Apr 10] [10 screens]. Available from: https://www.fda.gov/downloads/BiologicsBloodVaccines/GuidanceComplianceRegulatoryInformation/Guidances/Vaccines/ucm091977.pdf.

Department of Health and Human Services (DHHS), Food and Drug Administration, Center for Biologics Evaluation and Research (US). Guidance for Industry, Investigators, and Institutional Review Boards. FDA Guidance on Conduct of Clinical Trials of Medical Products during COVID-19 Public Health Emergency. September 2020. Updated on April 16, 2020. [cited 2020 Apr 17]. Available from: https://www.fda.gov/media/136238/download.

Department of Health and Human Services (DHHS), Food and Drug Administration, Center for Drug Evaluation and Research, Center for Biologics Evaluation and Research (US). Multiple Endpoints in Clinical Trials Guidance for Industry. January 2017. [cited 202 Oct 4]. Available https://www.fda.gov/files/drugs/published/Multiple-Endpoints-in-Clinical-Trials-Guidance-for-Industry.pdf

Ferdinands JM, Rao S, Dixon BE, Mitchell PK, DeSilva MB, Irving SA, et al. Waning 2-Dose and 3-Dose Effectiveness of mRNA Vaccines Against COVID-19-Associated Emergency Department and Urgent Care Encounters and Hospitalizations Among Adults During Periods of Delta and Omicron Variant Predominance - VISION Network, 10 States, August 2021-January 2022. MMWR Morb Mortal Wkly Rep. 2022;71(7):255-63.

Ferreira VM, Schulz-Menger J, Holmvang G, Kramer CM, Carbone I, Sechtem U, et al. Cardiovascular magnetic resonance in nonischemic myocardial inflammation: expert recommendations. J Am Coll Cardiol. 2018;72(24):3158-76.

Fontanet A, Grant R, Tondeur L, Madec Y, Grzelak L, Cailleau I, et al. SARS-CoV-2 infection in primary schools in northern France: A retrospective cohort study in an area of high transmission. medRxiv. 2020.

Fulginiti VA, Eller JJ, Downie AW, Kempe CH. Altered reactivity to measles virus. Atypical measles in children previously immunized with inactivated measles virus vaccines. JAMA. 1967;202(12):1075-80.

Gargano JW, Wallace M, Hadler SC, Langley G, Su JR, Oster ME, et al. Use of mRNA COVID-19 vaccine after reports of myocarditis among vaccine recipients: update from the advisory committee on immunization practices - United States, June 2021. MMWR Morb Mortal Wkly Rep. 2021;70(27):977-82.

Girard B, JE Tomassini, W Deng, M Maglinao, H Zhou, A Figueroa, et al. mRNA-1273 Vaccine-elicited Neutralization of SARS-CoV-2 Omicron in Adolescents and Children. medRxiv. 2022.

Goga A, Feucht U, Pillay S, Reubenson G, Jeena P, Mahdi S, et al. Parental access to hospitalised children during infectious disease pandemics such as COVID-19. S Afr Med J. 2021;111(2):100-5.

Hardin AP, Hackell JM, Committee on practice and ambulatory medicine. Age limit of pediatrics. Pediatrics. 2017;140(3):e20172151.

Hastie KM, Li H, Bedinger D, Schendel SL, Dennison SM, Li K, et al. Defining variant-resistant epitopes targeted by SARS-CoV-2 antibodies: a global consortium study. Science. 2021;374(6566):472-8.

Jackson LA, Anderson EJ, Rouphael NG, Roberts PC, Makhene M, Coler RN, et al. An mRNA vaccine against SARS-CoV-2 – preliminary report. N Engl J Med. 2020;383(20):1920-31.

Johnson RF, Bagci U, Keith L, Tang X, Mollura DJ, Zeitlin L, et al. 3B11-N, a monoclonal antibody against MERS-CoV, reduces lung pathology in rhesus monkeys following intratracheal inoculation of MERS-CoV Jordan-n3/2012. Virology. 2016;490:49-58.

Ju B, Zhang Q, Ge J, Wang R, Sun J, GE X, et al. Human neutralizing antibodies elicited by SARS-CoV-2 infection. Nature. 2020;584(7819)115-19.

Kim Y, Lee H, Park K, Park S, Lim JH, So MK, et al. Selection and characterization of monoclonal antibodies targeting Middle East Respiratory Syndrome coronavirus through a human synthetic fab phage display library panning. Antibodies (Basel). 2019;8(3):42.

Klein NP, Stockwell MS, Demarco M, Gaglani M, Kharbanda AB, Irving SA, et al. Effectiveness of COVID-19 Pfizer-BioNTech BNT162b2 mRNA Vaccination in Preventing COVID-19-Associated Emergency Department and Urgent Care Encounters and Hospitalizations Among Nonimmunocompromised Children and Adolescents Aged 5-17 Years - VISION Network, 10 States, April 2021-January 2022. MMWR Morb Mortal Wkly Rep. 2022;71(9):352-58.

Marks KJ, Whitaker M, Anglin O, Milucky J, Patel K, Pham H, et al. COVID-NET Surveillance Team. Hospitalizations of Children and Adolescents with Laboratory-Confirmed COVID-19 - COVID-NET, 14 States, July 2021-January 2022. MMWR Morb Mortal Wkly Rep. 2022a;71(7):271-8.

Marks KJ, Whitaker M, Agathis NT, Anglin O, Milucky J, Patel K, et al. COVID-NET Surveillance Team. Hospitalization of Infants and Children Aged 0-4 Years with Laboratory-Confirmed COVID-19 - COVID-NET, 14 States, March 2020-February 2022. MMWR Morb Mortal Wkly Rep. 2022b;71(11):429-36.

Patel T, Kelleman M, West Z, Peter A, Dove M, Butto A, et al. Comparison of Multisystem Inflammatory Syndrome in Children-Related Myocarditis, Classic Viral Myocarditis, and COVID-19 Vaccine-Related Myocarditis in Children. J Am Heart Assoc. 2022;11(9):e02439.

Robbiani DF, Gaebler C, Muecksch F, Lorenzi JC, Wang Z, Cho A, et al. Convergent antibody responses to SARS-CoV-2 in convalescent individuals. Nature. 2020;584(7821):437-42.

Rüggeberg JU, Gold MS, Bayas JM, Blum MD, Bonhoeffer J, Friedlander S, et al. Brighton Collaboration Anaphylaxis Working Group. Anaphylaxis: case definition and guidelines for data collection, analysis, and presentation of immunization safety data. Vaccine. 2007;25(31):5675-84.

Scheaffer SM, Lee D, Whitener B, Ying B, Wu K, Jani H et al. Bivalent SARS-CoV-2 mRNA vaccines increase breadth 1 of neutralization and protect against the BA.5 Omicron variant. bioRxiv 2022.09.12.507614.

Thomas SJ, Yoon IK. A review of Dengvaxia®: development to deployment. Hum Vaccin Immunother. 2019;15(10), 2295-314.

Tseng HF, Ackerson BK, Luo Y, Sy LS, Talarico CA, Tian Y, et al. Effectiveness of mRNA-1273 against SARS-CoV-2 Omicron and Delta variants. Nat Med. 2022;28(5):1063-71.

UK Health Security Agency. COVID-19 vaccine surveillance report – Week 7. 17 February 2022. [Available from: COVID-19 vaccine surveillance report - week 7 (publishing.service.gov.uk)]

Vogel TP, Top KA, Karatzios C, Hilmers DC, Tapia LI, Moceri P, et al. Multisystem inflammatory syndrome in children and adults (MIS-C/A): Case definition & guidelines for data collection, analysis, and presentation of immunization safety data. Vaccine. 2021;39(22):3037-49.

Wang L, Shi W, Joyce MG, Modjarrad K, Zhang Y, Leung K, et al. Evaluation of candidate vaccine approaches for MERS-CoV. Nat Commun. 2015;6:7712.

Wang L, Shi W, Chappell JD, Joyce MG, Zhang Y, Kanekiyo M, et al. Importance of neutralizing monoclonal antibodies targeting multiple antigenic sites on the middle east respiratory syndrome coronavirus spike glycoprotein to avoid neutralization escape. J Virol. 2018;92(10):e02002-17.

Widjaja I, Wang C, van Haperen R, Gutiérrez-Álvarez J, van Dieren B, Okba NMA, et al. Towards a solution to MERS: protective human monoclonal antibodies targeting different domains and functions of the MERS-coronavirus spike glycoprotein. Emerg Microbes Infect. 2019;8(1):516-30.

Wong H-Lea. Elevated Risk of Myocarditis/Pericarditis Following COVID-19 mRNA Vaccination in the United States. Lancet. 2022;Preprint.

World Health Organization (WHO). Dengue vaccine: WHO position paper, September 2018 – recommendations. Vaccine. 2019;37(35):4848-49.

World Health Organization. (2020a, Jun 25). Coronavirus disease 2019 (COVID-19) Situation Report – 157. Retrieved from: https://www.who.int/docs/default-source/coronaviruse/situation-reports/20200625-covid-19-sitrep-157.pdf?sfvrsn=423f4a82_2. (accessed 2020 Jun 26).

World Health Organization. (2020b, Sep 28). WHO coronavirus disease (COVID-19) dashboard. Retrieved from: https://covid19.who.int/. (accessed 2020 Sep 28).

Yu X, Zhang S, Jiang L, Cui Y, Li D, Wang D, et al. Structural basis for the neutralization of MERS-CoV by a human monoclonal antibody MERS-27. Sci Rep. 2015;5:13133.

Zent O, Arras-Reiter C, Broeker M, Hennig R. Immediate allergic reactions after vaccinations - a postmarketing surveillance review. Eur J Pediatr. 2002;161(1):21-5.

10. SUPPORTING DOCUMENTATION AND OPERATIONAL CONSIDERATIONS

10.1. APPENDIX 1: Schedule of Assessments

The schedules of assessments are presented in Table 10 for Part 1A, Table 12 for Part 1B, Table 13 for Part 1C-1, Table 14 for Part 1C-2, Table 15 for Part 2, and Table 16 for Part 3.

If a participant cannot attend a study site visit (scheduled or unscheduled) with the exception of Screening or Day 1, a home visit is acceptable if performed by appropriately delegated study site staff or a home healthcare service provided by the Sponsor (Section 7). If neither a participant visit to the study site nor a home visit to the participant is possible (with the aforementioned exceptions), a safety telephone call should be performed that includes the assessments scheduled for the safety telephone calls.

After the Participant Decision Clinic Visit, participants will continue to follow the Part 1A SoA (Table 10) or Part 1B SoA (Table 12), as described in Section 3.1.1.2 and as shown in Figure 8. All eligible participants will be offered a BD and those who choose to receive BD will follow Part 1C-1 SoA (Table 13). Eligible participants that completed their primary COVID-19 vaccination series under EUA and choose to receive a BD will follow Part 1C-2 SoA (Table 14). Eligible participants enrolled in Part 2 will follow Part 2 SoA (Table 15). Eligible participants enrolled in Part 3 will follow Part 3 SoA (Table 16).

Table 10: Schedule of Assessments Part 1A, the Blinded Phase; Part 1B Open-label Observational Phase for Participants who Received mRNA-1273 in Part 1A

Visit Number	0	1	2	3	4	5	-		6	-		7
**Type of Visit**	C	C	Virtual Call	C	Virtual Call	C	SFU		C	SFU		C
**Month Timepoint**		M0		M1		M2	eDiary	SC	M7	eDiary	SC	M13
**Study Visit Day**	D0[1] (Screening)	D1 (Baseline)	D8[2]	D29[3]	D36[2, 3]	D57[2, 3]	Every 4 weeks D71 – D183[3, 4]	Every 4 weeks D85– D197[3, 5]	D209/ Participant Decision Visit[3,6]	Every 4 weeks D223– D363[3, 4]	Every 4 weeks D237– D377[3, 5]	D394[3]
**Window Allowance (Days)**	- 28		+ 3	+ 7	+ 3	+ 7	±3	± 3	- 56/+ 56	± 3	± 3	± 14
**Days Since Most Recent Injection**	-	0	7	28/0	7	28	-	-	180	-	-	365
Informed consent/assent form, demographics, concomitant medications, medical history	X											
Revised informed consent/assent form									X			
Review of inclusion and exclusion criteria	X	X										
Physical examination including vital signs, height, weight[7]	X	X		X		X			X			X
Pregnancy test[8]	X	X		X								
Randomization		X										
Study injection (including 30-minute post dose observation period)		X		X								

**Visit Number**	**0**	**1**	**2**	**3**	**4**	**5**	**-**		**6**	**-**		**7**
**Type of Visit**	**C**	**C**	**Virtual Call**	**C**	**Virtual Call**	**C**	**SFU**		**C**	**SFU**		**C**
**Month Timepoint**		**M0**		**M1**		**M2**	**eDiary**	**SC**	**M7**	**eDiary**	**SC**	**M13**
**Study Visit Day**	**D0[1] (Screening)**	**D1 (Baseline)**	**D8[2]**	**D29[3]**	**D36[2, 3]**	**D57[2, 3]**	**Every 4 weeks D71 – D183[3,4]**	**Every 4 weeks D85– D197[3, 5]**	**D209/ Participant Decision Visit [3,6]**	**Every 4 weeks D223– D363[3,4]**	**Every 4 weeks D237– D377[3,5]**	**D394[3]**
**Window Allowance (Days)**	**- 28**		**+ 3**	**+ 7**	**+ 3**	**+ 7**	**±3**	**± 3**	**- 56/+ 56**	**± 3**	**± 3**	**± 14**
**Days Since Most Recent Injection**	**-**	**0**	**7**	**28/0**	**7**	**28**	**-**	**-**	**180**	**-**	**-**	**365**
Blood sample for vaccine immunogenicity[9]		X				X			X			X
Nasopharyngeal or nasal swab sample for SARS-CoV-2[10]		X		X		X			X			
Surveillance for COVID-19/ Illness visit[11]/ Unscheduled visit		X	X	X	X	X	X	X	X	X	X	X
Convalescent Visit[12]		X	X	X	X	X	X	X	X	X	X	X
eDiary activation for recording solicited ARs (7 days)[13]		X		X								
Review of eDiary data			X		X							
Follow-up safety telephone calls[14]								X		X		
Recording of unsolicited AEs		X	X	X	X	X						
Recording of MAAEs and concomitant medications relevant to or for the treatment of the MAAE[15]		X	X	X	X	X	X		X	X		X

Visit Number	0	1	2	3	4	5	-		6	-		7
**Type of Visit**	C	C	Virtual Call	C	Virtual Call	C	SFU		C	SFU		C
**Month Timepoint**		M0		M1		M2	eDiary	SC	M7	eDiary	SC	M13
**Study Visit Day**	D0[1] (Screening)	D1 (Baseline)	D8[2]	D29[3]	D36[2, 3]	D57[2, 3]	Every 4 weeks D71 – D183[3, 4]	Every 4 weeks D85– D197[3, 5]	D209/ Participant Decision Visit[3,6]	Every 4 weeks D223– D363[3, 4]	Every 4 weeks D237– D377[3, 5]	D394[3]
**Window Allowance (Days)**	- 28		+ 3	+ 7	+ 3	+ 7	±3	± 3	- 56/+ 56	± 3	± 3	± 14
**Days Since Most Recent Injection**	-	0	7	28/0	7	28	-	-	180	-	-	365
Recording of SAEs and concomitant medications relevant to or for the treatment of the SAE[15]		X	X	X	X	X	X		X	X		X
Recording of AESI		X	X	X	X	X	X		X	X		X
Recording of concomitant medications and nonstudy vaccinations[15]		X	X	X	X	X						
Study completion												X

Abbreviations: AE = adverse event; AESI = adverse event of special interest; AR = adverse reaction; C = clinic visit; COVID-19 = coronavirus disease 2019; D = day; eDiary = electronic diary; FDA = US Food and Drug Administration; IRB = institutional review board; M = month; MAAE = medically attended AE; SC = safety (telephone) call; SFU = safety follow-up; RT-PCR = reverse transcriptase polymerase chain reaction; SAE = serious adverse event; SARS-CoV-2 = Severe Acute Respiratory Syndrome coronavirus 2.

Note: In accordance with FDA Guidance on Conduct of Clinical Trials of Medical Products during COVID-19 Public Health Emergency (DHHS 2020), investigators may convert study site visits to home visits or telemedicine visits with the approval of the Sponsor.

Note: If during the Participant Decision Visit (Table 11) the participant is unblinded and determined to have received 2 doses of mRNA-1273 in Part 1A, due to statistical considerations, they will be considered in the Open-label Observational Phase but will continue to follow the Part 1A SoA.

1. Day 0 and Day 1 may be combined on the same day. Additionally, the Day 0 Visit may be performed over multiple visits if preformed within the 28-day Screening window.
2. All scheduled study visits should be completed within the respective visit windows. If the participant is not able to attend a study site visit due to the COVID-19 pandemic (self-quarantine or disruption of study site activities following business continuity plans and/or local government mandates for “stay at home” or “shelter in place”), a safety telephone call to the participant should be made in place of the study site visit. The safety telephone call should encompass all scheduled visit assessments that can be completed remotely, such as assessment for AEs and concomitant medications (eg, as defined in scheduled safety telephone calls). Home visits will be permitted for all

nondosing visits, except for Screening, if a participant cannot visit the study site due to the COVID-19 pandemic. Home visits must be permitted by the study site IRB and the participant via informed consent/assent and have prior approval from the Sponsor (or its designee).

3. If the visit for the second dose (Day 29) is disrupted and cannot be completed at Day 29 +7 days due to the COVID-19 pandemic (self-quarantine or disruption of study site activities following business continuity plans and/or local government mandates for "stay at home" or "shelter in place"), the visit window may be extended to Day 29 + 21 days. When the extended visit window is used, the remaining study visits should be rescheduled to follow the inter-visit interval from the actual date of the second dose.
4. Safety follow-up via an eDiary questionnaire will be performed every 4 weeks from Day 71 to Day 183 and again from Day 223 to Day 363.
5. Safety follow-up via a safety telephone call will be performed every 4 weeks from Day 85 to Day 197 and again from Day 237 to Day 377.
6. The Participant Decision Visit may be performed over multiple visits. Once the Participant Decision Visit has been initiated, all assessments must be completed within a 7-day period.
7. Physical examination: A full physical examination, including height and weight, will be performed at Day 1, Day 29, Day 57, Day 209, and Day 394. BMI will be calculated only at Screening Visit (Day 0). Symptom-directed physical examinations may be performed at other timepoints at the discretion of the investigator. On each injection day before injection and again 7 days after injection, the arm receiving the injection should be examined and the associated lymph nodes should be evaluated. Any clinically significant finding identified during a study visit should be reported as a MAAE. Vital signs are to be measured predose and postdose on days of injection (Day 1 and Day 29). When applicable, vital signs should be measured before blood collection. Participants who are febrile (body temperature ≥ 38.0°C/100.4°F) before injection on Day 1 or Day 29 must have the visit rescheduled within the relevant visit window to receive the injection. Afebrile participants with minor illnesses can be enrolled at the discretion of the investigator.
8. Pregnancy test at Screening and Day 1 and before the second study injection will be a point-of-care urine test. At the discretion of the investigator, a pregnancy test either via blood or point-of-care urine test can be performed at any time.
9. Sample must be collected prior to dosing of injection on Day 1.
10. The nasopharyngeal or nasal swab sample will be used to ascertain the presence of SARS-CoV-2 via RT-PCR.
11. An unscheduled visit may be prompted by reactogenicity issues, illness visit criteria for COVID-19, or new or ongoing AEs. If a participant meets the prespecified criteria of suspicion for COVID-19 (Section 7.1.6), the participants will be asked to return within 72 hours or as soon as possible to the study site for an unscheduled illness visit, to include an NP or nasal swab sample (for RT-PCR testing) and other clinical evaluations. If a study site visit is not possible, a home visit may be arranged to collect the NP or nasal sample and conduct clinical evaluations. If a home visit is not possible, the participant will be asked to submit a saliva sample to the study site by a Sponsor-approved method. At this illness visit, the NP or nasal swab samples will be collected to evaluate for the presence of SARS-CoV-2 infection. NP or nasal swab samples will also be tested for the presence of other respiratory pathogens. If due to COVID-19 related restrictions in the community, the participant's LAR performed a home test rather than obtaining a test performed by a health care professional, see Section 7.1.6. In addition, the study site may collect an additional NP or nasal sample for SARS-CoV-2 testing to be able to render appropriate medical care for the study participant as determined by local standards of care. Additionally, clinical information will be carefully collected to evaluate the severity of the clinical case.
12. A convalescent visit will be scheduled approximately 28 days (+7 days) after diagnosis. At this visit, an NP or nasal swab sampling for viral PCR and a blood sample will be collected for potential immunologic assessment of SARS-CoV-2 infection.
13. Diary entries will be recorded by the participant at approximately 30 minutes after injection while at the study site with instruction provided by study staff. Study participants will continue to record entries in the eDiary each day after they leave the study site, preferably in the evening, on the day of injection and for 6 days following injection. If a solicited local or systemic AR continues beyond Day 7 after vaccination, the participant will be prompted to capture details of the solicited local or systemic AR in the eDiary until the AR is resolves or the next IP injection occurs, whichever occurs first; capture of details of ARs in the eDiary should not exceed 28 days after each vaccination. ARs recorded in eDiaries beyond Day 7 should be reviewed either via telephone call or at the following study visit.
14. Trained study site personnel will call all participants to collect information relating to any AEs, MAAEs, SAEs, AEs leading to study withdrawal, information on concomitant medications associated with those events, and any nonstudy vaccinations. In addition, study personnel will collect information on known participant exposure to someone with known COVID-19 or SARS-CoV-2 infection and on participant experience of COVID-19 symptoms.
15. All concomitant medications and nonstudy vaccinations will be recorded through 28 days after each injection; all concomitant medications relevant to or for the treatment of an SAE or MAAE will be recorded from Day 1 through the final visit (Day 394).

Table 11: Participant Decision Clinic Visit

	All Participants
Return to clinic for Participant Decision Clinic Visit	X
Sign revised Informed Consent Form	X
Confirm participant's choice to be unblinded or not to be unblinded	X
Confirm participant's choice to receive open-label mRNA-1273	X
Nasopharyngeal or nasal swab	X
Blood for immunologic analysis	X
Counsel about public health measures to limit virus spread[1]	X

1. All participants are counseled about the importance of continuing other public health measures to limit the spread of disease including physical-social distancing, wearing a mask, and hand-washing.

Figure 8: Schedule of Part 1A and Part 1B Participant Visits During the Part 1B Open-label Period

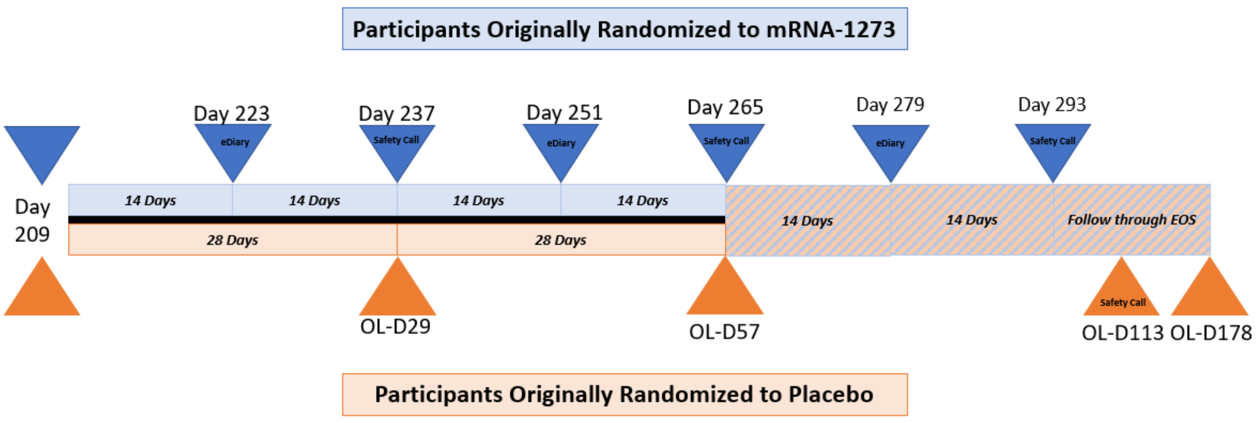

Table 12: Schedule of Assessments Part 1B, Open-label Observational Phase for Participants Who Previously Received Placebo

Visit Number	7	8	9	10	11
**Type of Visit**	**C**	**C**	**C**	**SC[8]**	**C**
**Study Visit Day**	**(~D209/ Participant Decision Visit) OL-D1**	**OL-D29**	**OL-D57[2]**	**OL-D113**	**OL-D178[9]**
**Window Allowance (Days)**	**-56/+56[1]**	**-3/+7**	**±14**	**± 3**	**±14**
**Days Since Most Recent Injection**	**0**	**28**	**28**	**84**	**149**
Informed consent form	These assessments are already performed as part of the regular D209 Visit.				
Blood for vaccine immunogenicity			X		X
Nasopharyngeal or nasal swab sample for SARS-CoV-2[3]		X	X		
Physical examination including vital signs[4]	PE including vitals performed as part of D209 Visit.	X	X		X
	X Vitals obtained post dose.	X			
Pregnancy testing	X	X			
Study injection (including 30-minute postdosing observation period)	X	X			
Informed consent for BD			X[7]		
Recording of MAAEs and concomitant medications relevant to or for the treatment of the MAAE[5]	X	X	X	X	X
Recording of SAEs and concomitant medications relevant to or for the treatment of the SAE[5]	X	X	X	X	X
Recording of AESI	X	X	X	X	X
Recording of concomitant medications and nonstudy vaccinations[5]	X	X	X	X	X
Surveillance for COVID-19/ Illness visit[6]/ Unscheduled visit	X	X	X	X	X
Study completion (for Part 1B)					X

Abbreviations: AE = adverse event; AESI = adverse event of special interest; C = clinic visit; COVID-19 = coronavirus disease 2019; D = Day; M = month; MAAE = medically attended AE; NP = nasopharyngeal; OL = open-label; PCR = polymerase chain reaction; RT-PCR = reverse transcriptase polymerase chain reaction; SAE = serious adverse events; SARS-CoV-2 = Severe Acute Respiratory Syndrome coronavirus 2; SC = safety (telephone) call.

1. The Participant Decision Visit may be performed over multiple visits. Once the Participant Decision Visit has been initiated, all assessments must be completed within a 7-day period.
2. After the OL-D57 visit, Part 1B participants who choose to receive BD will have one additional safety call at OL-D113 prior to entering Part 1C-1.
3. The NP or nasal swab sample, collected prior to vaccination on days of injection, will be used to ascertain the presence of SARS-CoV-2 via PCR.
4. Physical examination: A symptom-directed physical examination will be performed at OL-D1, OL-D29, OL-D57, and OL-D178. Symptom-directed physical examinations may be performed at other timepoints at the discretion of the investigator. Any clinically significant finding identified during a study visit should be reported as a MAAE. Vital signs are to be collected predosing and postdosing on days of injection (OL-D1 and OL-D29). When applicable, vital sign measurements should be performed before blood collection. Participants who are febrile (body temperature ≥ 38.0°C/100.4°F) before injection on OL-D1 or OL-D29 must be rescheduled within the relevant window period to receive the injection. Afebrile participants with minor illnesses can be administered investigational product at the discretion of the investigator.
5. All concomitant medications and nonstudy vaccinations will be recorded through 28 days postinjection; all concomitant medications relevant to or for the treatment of an SAE or MAAE will be recorded from Day 1 through OL-D113 before entering Part 1C-1. If a participant declines a BD, all concomitant medications relevant to or for the treatment of an SAE or MAAE will be recorded from Day 1 through OL-D178.
6. If a participant meets the prespecified criteria of suspicion for COVID-19 (Section 7.1.6), the participants will be asked to return within 72 hours or as soon as possible to the study site for an unscheduled illness visit, to include an NP or nasal swab sample (for RT-PCR testing) and other clinical evaluations. If a study site visit is not possible, a home visit may be arranged to collect the NP or nasal sample and conduct clinical evaluations. If a home visit is not possible, the participant will be asked to submit a saliva sample to the study site by a Sponsor-approved method. At this illness visit, the NP or nasal swab samples will be collected to evaluate for the presence of SARS-CoV-2 infection. NP or nasal swab samples will also be tested for the presence of other respiratory pathogens. If due to COVID-19 related restrictions in the community, the participant's LAR performed a home test rather than obtaining a test performed by a health care professional, see Section 7.1.6. In addition, the study site may collect an additional NP or nasal sample for SARS-CoV-2 testing to be able to render appropriate medical care for the study participant as determined by local standards of care. Additionally, clinical information will be carefully collected to evaluate the severity of the clinical case.
7. During OL-D57, all participants will be given the option to receive a BD. Participants will choose whether to receive the BD or not and sign the consent form. Those who decline BD will continue with Part 1B. Those who choose to receive a BD will have one safety call prior to entering Part 1C-1.
8. All participants in Part 1B who choose to receive BD will have one additional safety call at OL-D113 prior to entering Part 1C-1.
9. OL-D178 is only for those who decline BD. Participants who choose to receive a BD will follow Part 1C-1 SoA after the OL-D113 safety call. The BD-D1 has a window of -1 month, thus, can be scheduled at least 5 months after the second dose, and participants will need to complete the OL-D113 safety call.

Table 13: Schedule of Assessments Part 1C-1 – Homologous Booster Dose Phase

Visit Number	BD-1	BD-1a		BD-2	BD-3		BD-4
Type of Visit	C	C	SC[11]	C	C	SC	C
Study Visit Day	BD-D1[1] (D209: mRNA-1273; D394: placebo-mRNA-1273)	BD-D4	7, 14, and 21 days after BD-D1 (BD-D8, BD-D15, and BD-D22)	BD-D29	BD-D181 (Day 390: mRNA-1273; Day 571: placebo-mRNA-1273)	270 days after BD-1 (BD-D271)	BD-D361 (Day 571 mRNA-1273; Day 751 placebo-mRNA-1273)
Window Allowance (Days)	-28 days	-2	+3	-3/+14	-3/+14	+3	-3/+14
Days Since Most Recent Vaccination (in Part 1C-1)	0	3	7, 14, 21	28	180	270	360
Confirm informed consent form signing	X						
Physical examination[2]	X			X	X		X
Pregnancy testing[3]	X						
**Immunogenicity Assessment**							
Blood for immunologic analysis[4]	X			X	X		X
**Biomarker Assessment**							
Blood sample for potential biomarker analysis[5]		X					
**Dosing**							
Study injection (including 30-minute postdosing observation period[6])	X						
**Efficacy Assessment**							
Surveillance for COVID-19/Unscheduled Visit[7]	X	X	X	X	X	X	X
Nasal swab[8]	X						
**Safety Assessments**							

Visit Number	BD-1	BD-1a		BD-2	BD-3		BD-4
Type of Visit	C	C	SC[11]	C	C	SC	C
Study Visit Day	BD-D1[1] (D209: mRNA-1273; D394: placebo-mRNA-1273)	BD-D4	7, 14, and 21 days after BD-D1 (BD-D8, BD-D15, and BD-D22)	BD-D29	BD-D181 (Day 390: mRNA-1273; Day 571: placebo-mRNA-1273)	270 days after BD-1 (BD-D271)	BD-D361 (Day 571 mRNA-1273; Day 751 placebo-mRNA-1273)
Window Allowance (Days)	-28 days	-2	+3	-3/+14	-3/+14	+3	-3/+14
Days Since Most Recent Vaccination (in Part 1C-1)	0	3	7, 14, 21	28	180	270	360
Follow-up safety[9]			X			X	X
eDiary activation for recording solicited ARs (7 days)[10]	X						
Review of eDiary data[10]			X[10(BD-D8)]				
Recording of unsolicited AEs[11]	X	X	X	X			
Recording of MAAEs and concomitant medications relevant to or for the treatment of the MAAE[12]	X	X	X	X	X	X	X
Recording of AE leading to withdrawal	X	X	X	X	X	X	X
Recording of AESIs	X	X	X	X	X	X	X
Recording of SAEs and concomitant medications relevant to or for the treatment of the SAE[12]	X	X	X	X	X	X	X
Recording of concomitant medications and nonstudy vaccinations	X	X	X	X	X	X	X

Abbreviations: AE = adverse event; AESI = adverse event of special interest; AR = adverse reaction; BD = booster dose; C = clinic visit; D = day; eDiary = electronic diary; MAAE = medically attended AE; NP = nasopharyngeal; SAE = serious adverse event; SC = safety (phone) call.

Note: In accordance with FDA Guidance on Conduct of Clinical Trials of Medical Products during COVID-19 Public Health Emergency (DHHS 2020), investigators may convert study site visits to home visits or telemedicine visits with the approval of the Sponsor.

1. A BD may be administered to all participants who are currently enrolled in Part 1A or Part 1B provided there are no current contraindications for further dosing (Section 6). A participant who is currently in the Convalescent Period may come in for a BD-1 visit and receive a BD as long they are no longer symptomatic, and this includes the possibility for a Convalescent Visit Day 28 to overlap and be combined with a BD-1 visit.
2. Symptom-directed physical examination will be performed at the BD-Day 1. On dosing day before injection, the arm receiving the injection should be examined and the associated lymph nodes should be evaluated. At visits BD-2 (BD-D29), BD-3 (BD-D181) and BD-4 (BD-D361), a symptom-directed physical examination may be performed at the discretion of the investigator. Any clinically significant finding identified during a study visit should be reported as a MAAE. Vital signs are to be collected predosing and postdosing (participant will be seated for at least 5 minutes before all measurements are taken per Section 7.1.4) on the day of injection (BD-D1). Participants who are febrile (body temperature ≥ 38.0°C/100.4°F) before dosing (BD-D1) must be rescheduled to receive the injection. Afebrile participants with minor illnesses can be vaccinated at the discretion of the investigator.
3. The pregnancy test at the BD-1 visit will be a point-of-care urine test. At the discretion of the investigator, a pregnancy test either via blood or point-of-care urine test can be performed.
4. Sample should be collected prior to dosing on BD-D1.
5. All participants who chose to receive a BD. Serum sample from two ~4 mL blood draws. Biomarker plasma and biomarker serum samples will be stored for potential future biomarker assessment.
6. Postdosing, participants will have a 30-minute observation period.
7. An unscheduled visit may be prompted by reactogenicity issues, illness visit criteria for COVID-19, or new or ongoing AEs. If a participant meets the prespecified criteria of suspicion for COVID-19 (Section 7.1.6), the participants will be asked to return within 72 hours or as soon as possible to the study site for an unscheduled illness visit, to include an NP or nasal swab sample (for RT-PCR testing) and other clinical evaluations. If a study site visit is not possible, a home visit may be arranged to collect the nasal swab sample and conduct clinical evaluations. If a home visit is not possible, the participant will be asked to submit a saliva sample to the study site by a Sponsor-approved method. At this illness visit, the NP or nasal swab samples will be collected to evaluate for the presence of SARS-CoV-2 infection. Nasal swab samples will also be tested for the presence of other respiratory pathogens. If due to COVID-19 related restrictions in the community, the participant's LAR performed a home test rather than obtaining a test performed by a health care professional, see Section 7.1.6. In addition, the study site may collect an additional nasal swab sample for SARS-CoV-2 testing to be able to render appropriate medical care for the study participant as determined by local standards of care. Additionally, clinical information will be carefully collected to evaluate the severity of the clinical case.
8. The nasal swab sample must be collected prior to injection at the BD-1 visit.
9. Trained study personnel will call all participants to collect information relating to any unsolicited AEs, MAAEs (including any signs and symptoms of COVID-19), AESIs, AEs leading to withdrawal, SAEs, information on concomitant medications associated with those events, and any nonstudy vaccinations.
10. Diary entries will be recorded by the participant at approximately 30 minutes after injection while at the study site with instruction provided by study staff. Study participants will continue to record entries in the eDiary each day after they leave the study site, preferably in the evening, on the day of injection and for 6 days following injection. If a solicited local or systemic AR continues beyond Day 7 after vaccination, the participant will be prompted to capture details of the solicited local or systemic AR in the eDiary until the AR resolves; capture of details of ARs in the eDiary should not exceed 28 days after vaccination. ARs recorded in eDiaries beyond Day 7 should be reviewed either via telephone call or at the following study visit. Review of eDiary will occur on BD-D8.
11. Only for participants who chose to receive a BD.
12. All concomitant medications relevant to or for the treatment of an SAE or MAAE will be recorded from Screening through the final visit.

Table 14: Schedule of Assessments Part 1C-2 – Heterologous Booster Phase

Visit Number	BD-0	BD-1	BD-1a		BD-2	BD-3		BD-4
Type of Visit	C	C	C	SC[11]	C	C	SC	C
Study Visit Day	BD-D0[1]	BD-D1[1]	BD-D4	7, 14, and 21 days after BD-D1 (BD-D8, BD-D15, BD-D22)	BD-D29	BD-D181	270 days after BD-1 (BD-D271)	BD-D361
Window Allowance (Days)	-7	-	-2	+3	-3/+14	-3/+14	+3	-3/+14
Days Since Most Recent Vaccination (in Part 1C-2)	-	0	3	7, 14, 21	28	180	270	360
Informed consent/assent form, demographics, concomitant medications, medical history	X							
Physical examination including vital signs, height, weight [2]	X	X			X	X		X
Pregnancy testing[3]	X	X						
**Immunogenicity Assessment**								
Blood for immunologic analysis[4]		X			X	X		X
**Biomarker Assessment**								
Blood sample for potential biomarker analysis[5]			X					
**Dosing**								
Study injection (including 30-minute postdosing observation period[6])		X						

Visit Number	BD-0	BD-1	BD-1a		BD-2	BD-3		BD-4
Type of Visit	C	C	C	SC[11]	C	C	SC	C
Study Visit Day	BD-D0[1]	BD-D1[1]	BD-D4	7, 14, and 21 days after BD-D1 (BD-D8, BD-D15, BD-D22)	BD-D29	BD-D181	270 days after BD-1 (BD-D271)	BD-D361
Window Allowance (Days)	-7	-	-2	+3	-3/+14	-3/+14	+3	-3/+14
Days Since Most Recent Vaccination (in Part 1C-2)	-	0	3	7, 14, 21	28	180	270	360
**Efficacy Assessment**								
Surveillance for COVID-19/Unscheduled Visit[7]		X	X	X	X	X	X	X
Nasal swab[8]		X						
**Safety Assessments**								
Follow-up safety[9]				X			X	X
eDiary activation for recording solicited ARs (7 days)[10]		X						
Review of eDiary data[10]				X[10(BD-D8)]				
Recording of unsolicited AEs[11]		X	X	X	X			
Recording of MAAEs and concomitant medications relevant to or for the treatment of the MAAE[12]		X	X	X	X	X	X	X
Recording of AE leading to withdrawal		X	X	X	X	X	X	X
Recording of AESIs		X	X	X	X	X	X	X

Visit Number	BD-0	BD-1	BD-1a		BD-2	BD-3		BD-4
**Type of Visit**	**C**	**C**	**C**	**SC[11]**	**C**	**C**	**SC**	**C**
**Study Visit Day**	**BD-D0[1]**	**BD-D1[1]**	**BD-D4**	**7, 14, and 21 days after BD-D1 (BD-D8, BD-D15, BD-D22)**	**BD-D29**	**BD-D181**	**270 days after BD-1 (BD-D271)**	**BD-D361**
**Window Allowance (Days)**	**-7**	**-**	**-2**	**+3**	**-3/+14**	**-3/+14**	**+3**	**-3/+14**
**Days Since Most Recent Vaccination (in Part 1C-2)**	**-**	**0**	**3**	**7, 14, 21**	**28**	**180**	**270**	**360**
Recording of SAEs and concomitant medications relevant to or for the treatment of the SAE[12]		X	X	X	X	X	X	X
Recording of concomitant medications and nonstudy vaccinations		X	X	X	X	X	X	X

Abbreviations: AE = adverse event; AESI = adverse event of special interest; AR = adverse reaction; BD = booster dose; C = clinic visit; D = day; eDiary = electronic diary; EUA = Emergency Use Authorization; MAAE = medically attended AE; NP = nasopharyngeal; SAE = serious adverse event; SC = safety (phone) call.

Note: In accordance with FDA Guidance on Conduct of Clinical Trials of Medical Products during COVID-19 Public Health Emergency (DHHS 2020), investigators may convert study site visits to home visits or telemedicine visits with the approval of the Sponsor.

1. BD-Day 0 and BD-Day 1 may be combined on the same day. Additionally, the BD-Day 0 Visit may be performed over multiple visits if preformed within the 7-day Screening window. A BD will be administered to all eligible participants who are at least 3 months from completion of primary series under EUA (outside of the study).
2. A full physical examination including height, weight, and vital signs will be performed at the BD-Day 1. On dosing day before injection, the arm receiving the injection should be examined and the associated lymph nodes should be evaluated. At visits BD-2 (BD-D29), BD-3 (BD-D181) and BD-4 (BD-D361), a symptom-directed physical examination may be performed at the discretion of the investigator. Any clinically significant finding identified during a study visit should be reported as a MAAE. Vital signs are to be collected predosing and postdosing (participant will be seated for at least 5 minutes before all measurements are taken per Section 7.1.4 on the day of injection (BD-D1). Participants who are febrile (body temperature ≥38.0°C/100.4°F) before dosing (BD-D1) must be rescheduled to receive the injection. Afebrile participants with minor illnesses can be vaccinated at the discretion of the investigator.
3. The pregnancy test at the BD-1 Visit will be a point-of-care urine test. At the discretion of the investigator, a pregnancy test either via blood or point-of-care urine test can be performed.

4. Sample should be collected prior to dosing on BD-D1.
5. Serum sample from two ~4 mL blood draws. Biomarker plasma and biomarker serum samples will be stored for potential future biomarker assessment.
6. Postdosing, participants will have a 30-minute observation period.
7. An unscheduled visit may be prompted by reactogenicity issues, illness visit criteria for COVID-19, or new or ongoing AEs. If a participant meets the prespecified criteria of suspicion for COVID-19 (Section 7.1.6), the participants will be asked to return within 72 hours or as soon as possible to the study site for an unscheduled illness visit, to include an NP or nasal swab sample (for RT-PCR testing) and other clinical evaluations. If a study site visit is not possible, a home visit may be arranged to collect the nasal swab sample and conduct clinical evaluations. If a home visit is not possible, the participant will be asked to submit a saliva sample to the study site by a Sponsor-approved method. At this illness visit, the NP or nasal swab samples will be collected to evaluate for the presence of SARS-CoV-2 infection. Nasal swab samples will also be tested for the presence of other respiratory pathogens. If due to COVID-19 related restrictions in the community, the participant’s LAR performed a home test rather than obtaining a test performed by a health care professional, see Section 7.1.6. In addition, the study site may collect an additional nasal swab sample for SARS-CoV-2 testing to be able to render appropriate medical care for the study participant as determined by local standards of care. Additionally, clinical information will be carefully collected to evaluate the severity of the clinical case.
8. The nasal swab sample must be collected prior to injection at the BD-1 Visit.
9. Trained study personnel will call all participants to collect information relating to any unsolicited AEs, MAAEs (including any signs and symptoms of COVID-19), AESIs, AEs leading to withdrawal, SAEs, information on concomitant medications associated with those events, and any nonstudy vaccinations.
10. Diary entries will be recorded by the participant at approximately 30 minutes after injection while at the study site with instruction provided by study staff. Study participants will continue to record entries in the eDiary each day after they leave the study site, preferably in the evening, on the day of injection and for 6 days following injection. If a solicited local or systemic AR continues beyond Day 7 after vaccination, the participant will be prompted to capture details of the solicited local or systemic AR in the eDiary until the AR resolves; capture of details of ARs in the eDiary should not exceed 28 days after vaccination. ARs recorded in eDiaries beyond Day 7 should be reviewed either via telephone call or at the following study visit. Review of eDiary will occur on BD-D8.
11. Only for participants who chose to receive a BD.
12. All concomitant medications relevant to or for the treatment of an SAE or MAAE will be recorded from Screening through the final visit.

Table 15: Schedule of Assessments Part 2

**Visit Number**	**0**	**1**	**2**	**3**	**4**	**5**	**6**	**7**	**8**	**-**		**9**	**-**		**10**
**Type of Visit**	**C**	**C**	**Virtual Call**	**C**	**C**	**Virtual Call**	**C**	**Virtual Call**	**C**	**SFU**		**C**	**SFU**		**C**
**Month Timepoint**		**M0**		**M1**			**M2**		**M3**	**eDiary**	**SC**	**M7**	**eDiary**	**SC**	**M13**
**Study Visit Day**	**D0[1] (Screening)**	**D1 (Baseline)**	**D8[2]**	**D29[3]**	**D32**	**D36[2, 3]**	**D57[2, 3]**	**D64**	**D85**	**Every 4 weeks D99-183[3, 4]**	**Every 4 weeks D113-197[3, 5]**	**209[3]**	**Every 4 weeks D223-307[3, 4]**	**Every 4 weeks D237-321[3, 5]**	**394[3]**
**Window Allowance (Days)**	**- 7**		**+ 3**	**+ 7**	**-2**	**+ 3**	**+ 7**	**+3**	**+7**	**±3**	**± 3**	**+7**	**± 3**	**± 3**	**± 14**
**Days Since Most Recent Injection**	**-**	**0**	**7**	**28**	**3**	**7**	**28**	**35**	**65**	**-**	**-**	**180**	**-**	**-**	**365**
Informed consent/assent form, demographics, concomitant medications, medical history	X														
Review of inclusion and exclusion criteria[6]	X	X													
Physical examination including vital signs, height, weight[7]	X	X		X			X					X			X
Pregnancy test[8]	X	X		X											
Study injection (including 30-minute post dose observation period)		X		X											
Blood sample for potential biomarker analysis[9]					X										
Blood sample for vaccine immunogenicity[10]		X		X			X		X			X			X

**Visit Number**	**0**	**1**	**2**	**3**	**4**	**5**	**6**	**7**	**8**	**-**		**9**	**-**		**10**
**Type of Visit**	**C**	**C**	**Virtual Call**	**C**	**C**	**Virtual Call**	**C**	**Virtual Call**	**C**	**SFU**		**C**	**SFU**		**C**
**Month Timepoint**		**M0**		**M1**			**M2**		**M3**	**eDiary**	**SC**	**M7**	**eDiary**	**SC**	**M13**
**Study Visit Day**	**D0[1] (Screening)**	**D1 (Baseline)**	**D8[2]**	**D29[3]**	**D32**	**D36[2, 3]**	**D57[2, 3]**	**D64**	**D85**	**Every 4 weeks D99-183[3, 4]**	**Every 4 weeks D113-197[3, 5]**	**209[3]**	**Every 4 weeks D223-307[3, 4]**	**Every 4 weeks D237-321[3, 5]**	**394[3]**
**Window Allowance (Days)**	**- 7**		**+ 3**	**+ 7**	**-2**	**+ 3**	**+ 7**	**+3**	**+7**	**±3**	**± 3**	**+7**	**± 3**	**± 3**	**± 14**
**Days Since Most Recent Injection**	**-**	**0**	**7**	**28**	**3**	**7**	**28**	**35**	**65**	**-**	**-**	**180**	**-**	**-**	**365**
Nasal swab sample for SARS-CoV-2[11]		X		X			X					X			
Surveillance for COVID-19/ Illness visit[12]/ Unscheduled visit		X	X	X	X	X	X	X	X	X	X	X	X	X	X
eDiary activation for recording solicited ARs (7 days)[13]		X		X											
Review of eDiary data[14]			X			X									
Follow-up safety telephone calls[15]											X			X	
Recording of unsolicited AEs		X	X	X	X	X	X								
Recording of MAAEs and concomitant medications relevant to or for the treatment of the MAAE[16]		X	X	X	X	X	X	X	X	X	X	X	X	X	X

Visit Number	0	1	2	3	4	5	6	7	8	-		9	-		10
Type of Visit	C	C	Virtual Call	C	C	Virtual Call	C	Virtual Call	C	SFU		C	SFU		C
Month Timepoint		M0		M1			M2		M3	eDiary	SC	M7	eDiary	SC	M13
Study Visit Day	D0[1] (Screening)	D1 (Baseline)	D8[2]	D29[3]	D32	D36[2, 3]	D57[2, 3]	D64	D85	Every 4 weeks D99-183[3, 4]	Every 4 weeks D113-197[3, 5]	209 [3]	Every 4 weeks D223-307[3, 4]	Every 4 weeks D237-321[3, 5]	394[3]
Window Allowance (Days)	- 7		+ 3	+ 7	-2	+ 3	+ 7	+3	+7	±3	± 3	+7	± 3	± 3	± 14
Days Since Most Recent Injection	-	0	7	28	3	7	28	35	65	-	-	180	-	-	365
Recording of SAEs and concomitant medications relevant to or for the treatment of the SAE[16]		X	X	X	X	X	X	X	X	X	X	X	X	X	X
Recording of AESI		X	X	X	X	X	X	X	X	X	X	X	X	X	X
Recording of AEs leading to withdrawal		X	X	X	X	X	X	X	X	X	X	X	X	X	X
Recording of concomitant medications and nonstudy vaccinations[15]		X	X	X	X	X	X	X	X	X	X	X	X	X	X
Study completion															X

Abbreviations: AE = adverse event; AESI = adverse event of special interest; AR = adverse reaction; C = clinic visit; COVID-19 = coronavirus disease 2019; D = day; eDiary = electronic diary; FDA = US Food and Drug Administration; IRB = institutional review board; M = month; MAAE = medically attended AE; NP = nasopharyngeal; SC = safety (telephone) call; SFU = safety follow-up; RT-PCR = reverse transcriptase polymerase chain reaction; SAE = serious adverse event; SARS-CoV-2 = Severe Acute Respiratory Syndrome coronavirus 2.

Note: In accordance with FDA Guidance on Conduct of Clinical Trials of Medical Products during COVID-19 Public Health Emergency (DHHS 2020), investigators may convert study site visits to home visits or telemedicine visits with the approval of the Sponsor.

1. Day 0 and Day 1 may be combined on the same day. Additionally, the Day 0 Visit may be performed over multiple visits if they occur within the 7-day Screening window. If eligible, participants will receive 50 µg mRNA-1273 28 days apart.

2. All scheduled study visits should be completed within the respective visit windows. If the participant is not able to attend a study site visit due to the COVID-19 pandemic (self-quarantine or disruption of study site activities following business continuity plans and/or local government mandates for "stay at home" or "shelter in place"), a safety telephone call to the participant should be made in place of the study site visit. The safety telephone call should encompass all scheduled visit assessments that can be completed remotely, such as assessment for AEs and concomitant medications (eg, as defined in scheduled safety telephone calls). Home visits will be permitted for all nondosing visits, except for Screening, if a participant cannot visit the study due to the COVID-19 pandemic. Home visits must be permitted by the study site IRB and the participant via informed consent/assent and have prior approval from the Sponsor (or its designee).
3. If the visit for the second dose (Day 29) is disrupted and cannot be completed at Day 29 +7 due to the COVID-19 pandemic (self-quarantine or disruption of study site activities following business continuity plans and/or local government mandates for "stay at home" or "shelter in place"), the visit window may be extended to Day 29 + 21 days. When the extended visit window is used, the remaining study visits should be rescheduled to follow the inter-visit interval from the actual date of the second dose.
4. Safety follow-up via an eDiary questionnaire will be performed every 4 weeks from Day 99 to Day 183 and again from Day 223 to Day 307.
5. Safety follow-up via a safety telephone call will be performed every 4 weeks from Day 113 to Day 197 and again from Day 237 to Day 321.
6. Review of inclusion and exclusion criteria will be conducted during Screening. However, this review may be conducted on Day 1 if Screening and Baseline procedures are conducted on the same day.
7. Physical examination: A full physical examination, including height, weight, and vital signs will be performed at Screening, Day 1, Day 29, Day 57, Day 209, and Day 394. BMI will be calculated only at Screening Visit (Day 0). Symptom-directed physical examinations may be performed at other timepoints at the discretion of the investigator. On each injection day before injection and again 7 days after injection, the arm receiving the injection should be examined and the associated lymph nodes should be evaluated. Any clinically significant finding identified during a study visit should be reported as a MAAE. Vital signs are to be measured predose and postdose on days of injection (Day 1 and Day 29). When applicable, vital signs should be measured before blood collection. Participants who are febrile (body temperature ≥38.0°C/100.4°F) before injection on Day 1 and Day 29 must have the visit rescheduled within the relevant visit window to receive the injection. Afebrile participants with minor illnesses can be enrolled at the discretion of the investigator.
8. Pregnancy test at Screening and Day 1 and before the second study injection will be a point-of-care urine test. At the discretion of the investigator, a pregnancy test either via blood or point-of-care urine test can be performed at any time.
9. Serum sample from two ~4 mL blood draws. Biomarker plasma and biomarker serum samples will be stored for potential future biomarker assessment.
10. Sample must be collected prior to dosing of injection on Day 1 and Day 29.
11. The nasal swab sample will be used to ascertain the presence of SARS-CoV-2 via RT-PCR.
12. An unscheduled visit may be prompted by reactogenicity issues, illness visit criteria for COVID-19, or new or ongoing AEs. If a participant meets the prespecified criteria of suspicion for COVID-19 (Section 7.1.6), the participants will be asked to return within 72 hours or as soon as possible to the study site for an unscheduled illness visit, to include a nasal swab sample (for RT-PCR testing) and other clinical evaluations. If a study site visit is not possible, a home visit may be arranged to collect the NP or nasal sample and conduct clinical evaluations. If a home visit is not possible, the participant will be asked to submit a saliva sample to the study site by a Sponsor-approved method. At this illness visit, the NP or nasal swab samples will be collected to evaluate for the presence of SARS-CoV-2 infection. NP or nasal swab samples will also be tested for the presence of other respiratory pathogens. In addition, the study site may collect an additional NP or nasal sample for SARS-CoV-2 testing to be able to render appropriate medical care for the study participant as determined by local standards of care. Additionally, clinical information will be carefully collected to evaluate the severity of the clinical case.
13. Diary entries will be recorded by the participant at approximately 30 minutes after injection while at the study site with instruction provided by study staff. Study participants will continue to record entries in the eDiary each day after they leave the study site, preferably in the evening, on the day of injection and for 6 days following injection. If a solicited local or systemic AR continues beyond Day 7 after vaccination, the participant will be prompted to capture details of the solicited local or systemic AR in the eDiary until the AR resolves or the next IP injection occurs, whichever occurs first; capture of details of ARs in the eDiary should not exceed 28 days after each vaccination.

14. ARs recorded in eDiaries beyond Day 7 after vaccination should be reviewed either via telephone call or at the following study visit.
15. Trained study site personnel will call all participants to collect information relating to any AEs, MAAEs, SAEs, AEs leading to study withdrawal, information on concomitant medications associated with those events, and any nonstudy vaccinations. In addition, study personnel will collect information on known participant exposure to someone with known COVID-19 or SARS-CoV-2 infection and on participant experience of COVID-19 symptoms.
16. All concomitant medications and nonstudy vaccinations will be recorded through 28 days after each injection; all concomitant medications relevant to or for the treatment of an SAE or MAAE will be recorded from Day 1 through the final visit (Day 394).

Table 16: Schedule of Assessments Part 3

**Visit Number**	**0**	**1**	**2**	**3**	**4**	**5**	**6**	**-**	**7**	**8**	**9**	**10**	**-**	**11**
**Type of Visit**	**C**	**C**	**C**	**Virtual Call**	**C**	**C**	**C**	**SFU**	**C**	**C**	**Virtual Call**	**C**	**SFU**	**C**
**Month Timepoint**		**M0**			**M1**	**M2**	**M3**	**SC**	**M6**			**M7**	**SC**	**M12**
**Study Visit Day**	**D0[1] (Screening)**	**D1 (Baseline)**	**D3**	**D8[2]**	**D29**	**D57**	**D85**	**Every 6 weeks D99-141[3]**	**D181[4]**	**D184**	**D188**	**D209**	**Every 6 weeks D223-D321[3]**	**D361**
**Window Allowance (Days)**	**- 7**		**-2**	**+ 3**	**+ 7**	**+3**	**+7**	**±3**	**+ 7**	**-2**	**+ 3**	**+ 7**	**±3**	**±14**
**Days Since Most Recent Injection**	**-**	**0**	**3**	**7**	**28**	**56**	**84**	**-**	**180/ 0**	**3**	**7**	**28**	**-**	**180**
Informed consent/assent form, demographics, concomitant medications, medical history	X													
Review of inclusion and exclusion criteria[5]	X	X												
Physical examination including vital signs, height, weight[6]	X	X			X				X			X		X
Pregnancy test[7]	X	X							X					
Study injection (including 30-minute post dose observation period)		X							X					
Blood sample for potential biomarker analysis[8]			X							X				
Blood sample for vaccine immunogenicity[9]		X			X		X		X			X		X
Nasal swab sample for SARS-CoV-2[10]		X			X		X		X			X		

Visit Number	0	1	2	3	4	5	6	-	7	8	9	10	-	11
**Type of Visit**	**C**	**C**	**C**	**Virtual Call**	**C**	**C**	**C**	**SFU**	**C**	**C**	**Virtual Call**	**C**	**SFU**	**C**
**Month Timepoint**		**M0**			**M1**	**M2**	**M3**	**SC**	**M6**			**M7**	**SC**	**M12**
**Study Visit Day**	**D0[1] (Screening)**	**D1 (Baseline)**	**D3**	**D8[2]**	**D29**	**D57**	**D85**	**Every 6 weeks D99-141[3]**	**D181[4]**	**D184**	**D188**	**D209**	**Every 6 weeks D223-D321[3]**	**D361**
**Window Allowance (Days)**	**- 7**		**-2**	**+ 3**	**+ 7**	**+3**	**+7**	**±3**	**+ 7**	**-2**	**+ 3**	**+ 7**	**±3**	**±14**
**Days Since Most Recent Injection**	**-**	**0**	**3**	**7**	**28**	**56**	**84**	**-**	**180/ 0**	**3**	**7**	**28**	**-**	**180**
Surveillance for COVID-19/ Illness visit[11]/ Unscheduled visit		X	X	X	X	X	X	X	X	X	X	X	X	X
eDiary activation for recording solicited ARs (7 days)[12]		X							X					
Review of eDiary data[13]				X							X			
Follow-up safety telephone calls[14]								X					X	
Recording of unsolicited AEs		X	X	X	X				X	X	X	X		
Recording of MAAEs and concomitant medications relevant to or for the treatment of the MAAE[14]		X	X	X	X	X	X	X	X	X	X	X	X	X
Recording of SAEs and concomitant medications relevant to or for the treatment of the SAE[14]		X	X	X	X	X	X	X	X	X	X	X	X	X
Recording of AESI		X	X	X	X	X	X	X	X	X	X	X	X	X
Recording of AEs leading to withdrawal		X	X	X	X	X	X	X	X	X	X	X	X	X
Recording of concomitant medications and nonstudy vaccinations[15]		X	X	X	X	X	X	X	X	X	X	X	X	X
Study completion														X

Abbreviations: AE = adverse event; AESI = adverse event of special interest; AR = adverse reaction; BMI = body mass index; C = clinic visit; COVID-19 = coronavirus disease 2019; D = day; eDiary = electronic diary; FDA = US Food and Drug Administration; IRB = institutional review board; M = month; MAAE = medically attended AE; NP = nasopharyngeal; SC = safety (telephone) call; SFU = safety follow-up; RT-PCR = reverse transcriptase polymerase chain reaction; SAE = serious adverse event; SARS-CoV-2 = Severe Acute Respiratory Syndrome coronavirus 2.

Note: In accordance with FDA Guidance on Conduct of Clinical Trials of Medical Products during COVID-19 Public Health Emergency (DHHS 2020), investigators may convert study site visits to home visits or telemedicine visits with the approval of the Sponsor.

1. Day 0 and Day 1 may be combined on the same day. Additionally, the Day 0 Visit may be performed over multiple visits if they occur within the 7-day Screening window.
2. All scheduled study visits should be completed within the respective visit windows. If the participant is not able to attend a study site visit due to the COVID-19 pandemic (self-quarantine or disruption of study site activities following business continuity plans and/or local government mandates for "stay at home" or "shelter in place"), a safety telephone call to the participant should be made in place of the study site visit. The safety telephone call should encompass all scheduled visit assessments that can be completed remotely, such as assessment for AEs and concomitant medications (eg, as defined in scheduled safety telephone calls). Home visits will be permitted for all nondosing visits, except for Screening, if a participant cannot visit the study site due to the COVID-19 pandemic. Home visits must be permitted by the study site IRB and the participant via informed consent/assent and have prior approval from the Sponsor (or its designee).
3. Safety follow-up via a safety telephone call will be performed every 6 weeks from Day 99 to Day 141 and again from Day 223 to Day 321.
4. If the visit for the second dose (Day 181) is disrupted and cannot be completed at Day 181 +7 due to the COVID-19 pandemic (self-quarantine or disruption of study site activities following business continuity plans and/or local government mandates for "stay at home" or "shelter in place"), the visit window may be extended to Day 181 +21 days. When the extended visit window is used, the remaining study visits should be rescheduled to follow the inter-visit interval from the actual date of the second dose.
5. Review of inclusion and exclusion criteria will be conducted during Screening. However, this review will be conducted on Day 1 if Screening and Baseline procedures are conducted on the same day.
6. Physical examination: A full physical examination, including height, weight, and vital signs will be performed at Screening, Day 1, Day 29, Day 181, Day 209, and Day 361. BMI will be calculated only at Screening Visit (Day 0). Symptom-directed physical examinations may be performed at other timepoints at the discretion of the investigator. On each injection day before injection and again 7 days after injection, the arm receiving the injection should be examined and the associated lymph nodes should be evaluated. Any clinically significant finding identified during a study visit should be reported as a MAAE. Vital signs are to be measured predose and postdose on days of injection (Day 1 and Day 181). When applicable, vital signs should be measured before blood collection. Participants who are febrile (body temperature ≥38.0°C/100.4°F) before injection on Day 1 and Day 181 must have the visit rescheduled within the relevant visit window to receive the injection. Afebrile participants with minor illnesses can be enrolled at the discretion of the investigator.
7. Pregnancy test at Screening and Day 1 and before the second study injection (Day 181) will be a point-of-care urine test. At the discretion of the investigator, a pregnancy test either via blood or point-of-care urine test can be performed at any time.
8. Serum and plasma samples from two ~4 mL blood draws. Biomarker plasma and biomarker serum samples will be stored for potential future biomarker assessment.
9. Sample must be collected prior to dosing of injection on Day 1 and Day 181.
10. The nasal swab sample will be used to ascertain the presence of SARS-CoV-2 via RT-PCR.
11. An unscheduled visit may be prompted by reactogenicity issues, illness visit criteria for COVID-19, or new or ongoing AEs. If a participant meets the prespecified criteria of suspicion for COVID-19 (Section 7.1.6), the participants will be asked to return within 72 hours or as soon as possible to the study site for an unscheduled illness visit, to include a nasal swab sample (for RT-PCR testing) and other clinical evaluations. If a study site visit is not possible, a home visit may be arranged to collect the NP or nasal sample and conduct clinical evaluations. If a home visit is not possible, the participant will be asked to submit a saliva sample to the study site by a Sponsor-approved method. At this illness visit, the NP or nasal swab samples will be collected to evaluate for the presence of SARS-CoV-2 infection. NP or nasal swab samples will also be tested for the presence of other respiratory pathogens. In addition, the study

site may collect an additional NP or nasal sample for SARS-CoV-2 testing to be able to render appropriate medical care for the study participant as determined by local standards of care. Additionally, clinical information will be carefully collected to evaluate the severity of the clinical case.

12. Diary entries will be recorded by the participant at approximately 30 minutes after injection while at the study site with instruction provided by study staff. Study participants will continue to record entries in the eDiary each day after they leave the study site, preferably in the evening, on the day of injection and for 6 days following injection. If a solicited local or systemic AR continues beyond Day 7 after vaccination, the participant will be prompted to capture details of the solicited local or systemic AR in the eDiary until the AR resolves or the next IP injection occurs, whichever occurs first; capture of details of ARs in the eDiary should not exceed 28 days after each vaccination.

13. ARs recorded in eDiaries beyond Day 7 after vaccination should be reviewed either via telephone call or at the following study visit.

14. Trained study site personnel will call all participants to collect information relating to any AEs, MAAEs, SAEs, AEs leading to study withdrawal, information on concomitant medications associated with those events, and any nonstudy vaccinations. In addition, study personnel will collect information on known participant exposure to someone with known COVID-19 or SARS-CoV-2 infection and on participant experience of COVID-19 symptoms.

15. All concomitant medications and nonstudy vaccinations will be recorded through 28 days after each injection; all concomitant medications relevant to or for the treatment of an SAE or MAAE will be recorded from Day 1 through the final visit (Day 361).

10.2. APPENDIX 2: Study Governance Considerations

10.2.1. Regulatory and Ethical Considerations

This study will be conducted in accordance with the protocol and with the following:

- Consensus ethical principles derived from international guidelines including the Declaration of Helsinki and Council for International Organizations of Medical Sciences International Ethical Guidelines.
- Applicable ICH GCP Guidelines.
- Applicable laws and regulatory requirements.
- The protocol, protocol amendments, ICF, IB, and other relevant documents (eg, advertisements) must be submitted to an IRB by the investigator and reviewed and approved by the IRB before the study is initiated.
- Any amendments to the protocol will require IRB approval before implementation of changes made to the study design, except for changes necessary to eliminate an immediate hazard to study participants.
- The investigator will be responsible for the following:
  - Providing written summaries of the status of the study to the IRB annually or more frequently in accordance with the requirements, policies, and procedures established by the IRB
  - Notifying the IRB of SAEs or other significant safety findings as required by IRB procedures
  - Providing oversight of the conduct of the study at the site and adherence to requirements of 21 CFR, ICH guidelines, the IRB, European regulation 536/2014 for clinical studies (if applicable), and all other applicable local regulations.

10.2.2. Study Monitoring

Before an investigational site can enter a participant into the study, a representative of the Sponsor or its representatives will visit the investigational study site to do the following:

- Determine the adequacy of the facilities
- Discuss with the investigator(s) and other personnel their responsibilities with regard to protocol adherence, and the responsibilities of the Sponsor or its representatives. This will be documented in a clinical study agreement between the Sponsor, the designated CRO, and the investigator.

According to ICH GCP guidelines, the Sponsor of the study is responsible for ensuring the proper conduct of the study with regard to protocol adherence and validity of data recorded on the eCRFs. The study monitor’s duties are to aid the investigator and the Sponsor in the maintenance of complete, accurate, legible, well-organized, and easily retrievable data. The study monitor will advise the investigator of the regulatory necessity for study-related monitoring, audits, IRB review, and inspection by providing direct access to the source

data/documents. In addition, the study monitor will explain to and interpret for the investigator all regulations applicable to the clinical evaluation of an IP as documented in ICH guidelines.

It is the study monitor's responsibility to inspect the eCRFs and source documentation throughout the study to protect the rights of the participants; to verify adherence to the protocol; to verify completeness, accuracy, and consistency of the data; and to confirm adherence of study conduct to any local regulations. Details will be outlined in the Clinical Monitoring Plan. During the study, a monitor from the Sponsor or a representative will have regular contacts with the investigational site, for the following:

- Provide information and support to the investigator(s).
- Confirm that facilities remain acceptable.
- Confirm that the investigational team is adhering to the protocol, that the data are being accurately recorded in the eCRFs, and that IP accountability checks are being performed.
- Perform source data verification. This includes a comparison of the data in the eCRFs with the participant's medical records at the hospital or practice, and other records relevant to the study. This will require direct access to all original records for each participant (eg, clinical charts or electronic medical record system).
- Record and report any protocol deviations not previously sent.
- Confirm AEs and SAEs have been properly documented on eCRFs and confirm any SAEs have been forwarded to the SAE Hotline, and those SAEs that met criteria for reporting have been forwarded to the IRB.

The monitor will be available between visits if the investigator(s) or other staff needs information or advice.

10.2.3. Audits and Inspections

The Sponsor, their designee(s), the IRB, or regulatory authorities will be allowed to conduct study site visits to the investigational facilities for the purpose of monitoring or inspecting any aspect of the study. The investigator agrees to allow the Sponsor, their designee(s), the IRB, or regulatory authorities to inspect the IP storage area, IP stocks, IP records, participant charts and study source documents, and other records relative to study conduct.

Authorized representatives of the Sponsor, a regulatory authority, and any IRB may visit the study site to perform audits or inspections, including source data verification. The purpose of a Sponsor audit or inspection is to systematically and independently examine all study-related activities and documents to determine whether these activities were conducted, and data were recorded, analyzed, and accurately reported according to the protocol, ICH E6(R2) GCP, and any applicable regulatory requirements. The investigator should contact the Sponsor immediately if contacted by a regulatory agency about an inspection.

The principal investigator must obtain IRB approval for the investigation. Initial IRB approval, and all materials approved by the IRB for this study including the participant consent/assent form and recruitment materials, must be maintained by the investigator and made available for inspection.

10.2.4. Financial Disclosure

The investigator is required to provide financial disclosure information to allow the Sponsor to submit the complete and accurate certification or disclosure statements required under 21 CFR 54. In addition, the investigator must provide the Sponsor with a commitment to promptly update this information if any relevant changes occur during the course of the investigation and for 1 year following the completion of the study.

The Sponsor, the CRO, and the study site are not financially responsible for further testing or treatment of any medical condition that may be detected during the screening process. In addition, in the absence of specific arrangements, the Sponsor, the CRO, and the study site are not financially responsible for further treatment of the disease under study.

10.2.5. Recruitment Procedures

Advertisements to be used for the recruitment of study participants, and any other written information regarding this study to be provided to the participant should be submitted to the Sponsor for approval. All documents must be approved by the IRB.

10.2.6. Informed Consent/Assent Process

The informed consent/assent document(s) must meet the requirements of 21 CFR 50, local regulations, ICH guidelines, Health Insurance Portability and Accountability Act, where applicable, and the IRB or study site. All consent documents will be approved by the appropriate IRB. The actual ICF used at each study site may differ, depending on local regulations and IRB requirements. However, all versions of the ICF must contain the standard information found in the sample ICF provided by the Sponsor. Any change to the content of the ICF must be approved by the Sponsor and the IRB prior to the ICF being used.

If new information becomes available that may be relevant to the participant’s willingness to continue participation in the study, this will be communicated to them in a timely manner. Such information will be provided via a revised ICF or an addendum to the original ICF.

The investigator or his/her representative will explain the nature of the study to the participant and answer all questions regarding the study.

The investigator is responsible for ensuring that the participant fully understands the nature and purpose of the study. Information should be given in both oral and written form whenever possible.

No participant should be obliged to participate in the study. The participant must be informed that participation is voluntary. Participants, their relatives, guardians, or (if applicable) LARs must be given ample opportunity to inquire about details of the study. The information must make clear that refusal to participate in the study or withdrawal from the study at any stage is without any prejudice to the participant’s subsequent care.

The participant must be allowed sufficient time to decide whether they wish to participate in the study.

The participant must be made aware of, and give consent to, direct access to his/her source medical records by study monitors, auditors, the IRB, and regulatory authorities. The participant should be informed that such access will not violate participant confidentiality or any applicable

regulations. The participant and/or participants' parent(s)/LAR(s) should also be informed that he/she is authorizing such access by signing the ICF.

A copy of the ICF(s) must be provided to the participant and/or participants' parent(s)/LAR(s).

A participant who is rescreened is not required to sign another ICF if the rescreening occurs within 28 days from the previous ICF signature date (within the initial Screening Period).

The ICF will also explain that excess serum from immunogenicity testing may be used for future research, which may be performed at the discretion of the Sponsor to further characterize the immune response to SARS-CoV-2, additional assay development, and the immune response across CoVs.

10.2.7. Protocol Amendments

No change or amendment to this protocol may be made by the investigator or the Sponsor after the protocol has been agreed to and signed by all parties unless such change(s) or amendment(s) has (have) been agreed upon by the investigator or the Sponsor. Any change agreed upon will be recorded in writing, and the written amendment will be signed by the investigator and the Sponsor. IRB approval is required prior to the implementation of an amendment, unless overriding safety reasons warrant immediate action, in which case the IRB(s) will be promptly notified.

Any modifications to the protocol or the ICF, which may impact the conduct of the study, potential benefit of the study, or may affect participant safety, including changes of study objectives, study design, participant population, sample sizes, study procedures, or significant administrative aspects will require a formal amendment to the protocol. Such an amendment will be released by the Sponsor, agreed by the investigator(s), and approved by the relevant IRB(s) prior to implementation. A signed and dated statement that the protocol, any subsequent relevant amended documents, and the ICF have been approved by relevant IRB(s) must be provided to the Sponsor before the study is initiated.

Administrative changes of the protocol are minor corrections and/or clarifications that have no effect on the way the study is to be conducted. These administrative changes will be released by the Sponsor, agreed by the investigators, and notified to the IRB(s).

10.2.8. Protocol Deviations

The noncompliance may be either on the part of the participant, the investigator, or the study site staff. As a result of protocol deviations, corrective actions are to be developed by the study site and implemented promptly.

After a participant proceeds to the Participant Decision Visit (Part 1A) of the study, participants who received mRNA-1273 will continue to follow the open-label Part 1A SoA (Table 10). Participants who received placebo will transition to open-label Part 1B of the study and will follow the Part 1B SoA (Table 12). Participants who transitioned to Part 1C-1, at least 5 months after last dose, will follow the Part 1C-1 SoA (Table 13). Eligible participants that completed their primary COVID-19 vaccination series under EUA and choose to receive a BD will follow Part 1C-2 SoA (Table 14). Participants enrolled in Part 2 will follow Part 2 SoA (Table 15). Participants enrolled in Part 3 will follow Part 3 SoA (Table 16).

It is the responsibility of the study site investigator to use continuous vigilance to identify and report protocol deviations to the Sponsor or its designee. All protocol deviations must be addressed in study source documents, reported to study monitor. Protocol deviations must be sent to the reviewing IRB per their policies. The study site investigator is responsible for knowing and adhering to the reviewing IRB requirements.

10.2.9. Data Protection

Participants will be assigned a unique identifier by the Sponsor. Any participant records or datasets that are transferred to the Sponsor will contain the identifier only; participant names or any information which would make the participant identifiable will not be transferred.

The participant must be informed that his/her personal study-related data will be used by the Sponsor in accordance with local data protection law. The level of disclosure must also be explained to the participant.

The participant must be informed that his/her medical records may be examined by clinical QA auditors or other authorized personnel appointed by the Sponsor, by appropriate IRB members, and by inspectors from regulatory authorities.

Individual participant medical information obtained as a result of this study is considered confidential, and disclosure to third parties is prohibited. Information will be accessible to authorized parties or personnel only. Medical information may be given to the participant's physician or to other appropriate medical personnel responsible for the participant's well-being. Each participant will be asked to complete a form allowing the investigator to notify the participant's primary health care provider of his/her participation in this study.

All laboratory specimens, evaluation forms, reports, and other records will be identified in a manner designed to maintain participant confidentiality. All records will be kept in a secure storage area with limited access. Clinical information will not be released without the written permission of the participant, except as necessary for monitoring and auditing by the Sponsor, its designee, relevant regulatory authority, or the IRB.

The investigator and all employees and coworkers involved with this study may not disclose or use for any purpose other than performance of the study, any data, record, or other unpublished, confidential information disclosed to those individuals for the purpose of the study. Prior written agreement from the Sponsor or its designee must be obtained for the disclosure of any confidential information to other parties.

10.2.10. Sample Retention and Future Biomedical Research

The retention period of laboratory samples will be 20 years, or as permitted by local regulations, to address further scientific questions related to mRNA-1273 or antirespiratory virus immune response. In addition, identifiable samples can be destroyed at any time at the request of the participant. During the study, or during the retention period, in addition to the analysis outlined in the study endpoints, exploratory analysis may be conducted using other Ab-based methodologies on any remaining blood or serum samples, including samples from participants who are screened but are not subsequently enrolled. These analyses will extend the search for other potentially relevant biomarkers to investigate the effect of mRNA-1273, as well as to determine how changes in biomarkers may relate to exposure and clinical outcomes. A decision

to perform such exploratory research may arise from new scientific findings related to the drug class or disease, as well as reagent and assay availability.

10.2.11. Dissemination of Clinical Study Data

The Sponsor shares information about clinical trials and results on publicly accessible websites, based on international and local legal and regulatory requirements, and other clinical trial disclosure commitments established by pharmaceutical industry associations. These websites include clinicaltrials.gov, EU clinical trial register (eu.ctr), as well as some national registries.

In addition, results from clinical trials are required to be submitted to peer-reviewed journals following internal company review for accuracy, fair balance, and intellectual property. For those journals that request sharing of the analyzable data sets that are reported in the publication, interested researchers are directed to submit their request to clinicalstudydatarequest.com.

Individual participant data and supporting clinical documents are available for request at clinicalstudydatarequest.com. While making information available, the privacy of participants in clinical studies sponsored by the Sponsor is assured. Details on data sharing criteria and process for requesting access can be found at this web address: clinicalstudydatarequest.com.

10.2.12. Data Quality Assurance and Quality Control

Data collection is the responsibility of the clinical study staff at the study site under the supervision of the study site investigator. The investigator is responsible for ensuring the accuracy, completeness, legibility, and timeliness of the data reported.

- All participant data relating to the study will be recorded in the eCRF unless transmitted to the Sponsor or designee electronically (eg, laboratory data). The investigator is responsible for verifying that data entries are accurate and correct by physically or electronically signing the eCRF.
- The investigator must maintain accurate documentation (source data) that supports the information entered in the eCRF.
- The investigator must permit study-related monitoring, audits, IRB review, and regulatory agency inspections and provide direct access to source data documents.
- Monitoring details describing strategy (eg, risk-based initiatives in operations and quality such as Risk Management and Mitigation Strategies and Analytical Risk-Based Monitoring), methods, responsibilities and requirements, including handling of noncompliance issues and monitoring techniques (central, remote, or on-site monitoring) are provided in the Clinical Monitoring Plan.
- The Sponsor or designee is responsible for the data management of this study including quality checking of the data.
- The Sponsor assumes accountability for actions delegated to other individuals (eg, CRO).
- Study monitors will perform ongoing source data verification to confirm that data entered into the eCRF by authorized study site personnel are accurate, complete, and verifiable from source documents; that the safety and rights of participants are being protected; and

that the study is being conducted in accordance with the currently approved protocol and any other study agreements, ICH GCP, and all applicable regulatory requirements.

- Records and documents, including signed ICFs, pertaining to the conduct of this study must be retained by the investigator for a period of at least 2 years after the last marketing application approval or, if not approved, 2 years following the discontinuance of the test article for investigation. If this requirement differs from any local regulations, the local regulations will take precedence unless the local retention policy is less than 2 years. No records may be destroyed during the retention period without the written approval of the Sponsor. No records may be transferred to another location or party without written notification to the Sponsor.

Quality assurance includes all the planned and systematic actions that are established to ensure that the clinical study is performed and the data are generated, documented (recorded), and reported according to ICH GCP and local/regional regulatory standards.

A QA representative from the Sponsor or a qualified designee, who is independent of and separated from routine monitoring, may periodically arrange inspections/audits of the clinical study by reviewing the data obtained and procedural aspects. These inspections may include on-site inspections/audits and source data checks. Direct access to source documents is required for the purpose of these periodic inspections/audits.

10.2.13. Data Collection and Management

This study will be conducted in compliance with ICH CGP guidelines. This study will also be conducted in accordance with the most recent version of the Declaration of Helsinki.

This study will use electronic data collection to collect data directly from the study site using eCRFs. The investigator is responsible for ensuring that all sections of each eCRF are completed promptly and correctly and that entries can be verified against any source data.

Study monitors will perform source document verification to identify inconsistencies between the eCRFs and source documents. Discrepancies will be resolved in accordance with the principles of GCP. Detailed study monitoring procedures are provided in the Clinical Monitoring Plan.

Adverse events will be coded with MedDRA. Concomitant medications will be coded using WHO - Drug Dictionary.

10.2.14. Source Documents

Source documents are original documents or certified copies, and include, but are not limited to, eDiaries, medical and hospital records, screening logs, informed consent/assent forms, telephone contact logs, and worksheets. Source documents provide evidence for the existence of the participant and substantiate the integrity of the data collected. Source documents are filed at the investigator’s study site.

Data reported on the CRF or entered in the eCRF that are transcribed from source documents must be consistent with the source documents or the discrepancies must be explained. The investigator may need to request previous medical records or transfer records, depending on the study. Also, current medical records must be available.

The Sponsor or its designee requires that the investigator prepare and maintain adequate and accurate records for each participant treated with the IP. Source documents such as any hospital, clinic, or office charts, and the signed ICFs are to be included in the investigator's files with the participant's study records.

10.2.15. Retention of Records

The principal investigator must maintain all documentation relating to the study for a period of at least 2 years after the last marketing application approval or, if not approved, 2 years following the discontinuance of the test article for investigation. If this requirement differs from any local regulations, the local regulations will take precedence unless the local retention policy is > 2 years.

If it becomes necessary for the Sponsor or the regulatory authority to review any documentation relating to the study, the investigator must permit access to such records. No records will be destroyed without the written consent of the Sponsor, if applicable. It is the responsibility of the Sponsor to inform the investigator when these documents no longer need to be retained.

10.2.16. Study and Site Closure

If the study is prematurely terminated or suspended, the Sponsor shall promptly inform the investigators, the IRBs, the regulatory authorities, and any CRO(s) used in the study of the reason for termination or suspension, as specified by the applicable regulatory requirements. The investigator shall promptly inform the participants and should assure appropriate participant therapy and/or follow-up.

The Sponsor or designee reserves the right to close the study site or terminate the study at any time for any reason at the sole discretion of the Sponsor.

The investigator may initiate study site closure at any time, provided there is reasonable cause and sufficient notice is given in advance of the intended termination.

Reasons for the early closure of a study site by the Sponsor or investigator may include but are not limited to:

- Continuation of the study represents a significant medical risk to participants
- Failure of the investigator to comply with the protocol, the requirements of the IRB or local health authorities, the Sponsor's procedures, or GCP guidelines
- Inadequate recruitment of participants by the investigator
- Discontinuation of further mRNA-1273 development

Study sites will be closed upon study completion. A study site is considered closed when all required documents and study supplies have been collected and a study site closure visit has been performed.

10.2.17. Publication Policy

The results of this study may be published or presented at scientific meetings. If this is foreseen, the investigator agrees to submit all manuscripts or abstracts to the Sponsor before submission. This allows the Sponsor to protect proprietary information and to provide comments.

The Sponsor will comply with the requirements for publication of study results. In accordance with standard editorial and ethical practice, the Sponsor will generally support publication of multicenter studies only in their entirety and not as individual study site data. In this case, a coordinating investigator will be designated by mutual agreement.

Authorship will be determined by mutual agreement and in line with International Committee of Medical Journal Editors authorship requirements.

The clinical study plan and the results of the study will be published on www.ClinicalTrials.gov in accordance with 21 CFR 50.25(c). The results of and data from this study belong to the Sponsor.

10.2.18. BMI Charts for Boys and Girls

<u>For boys aged 5 through 19 years:</u>

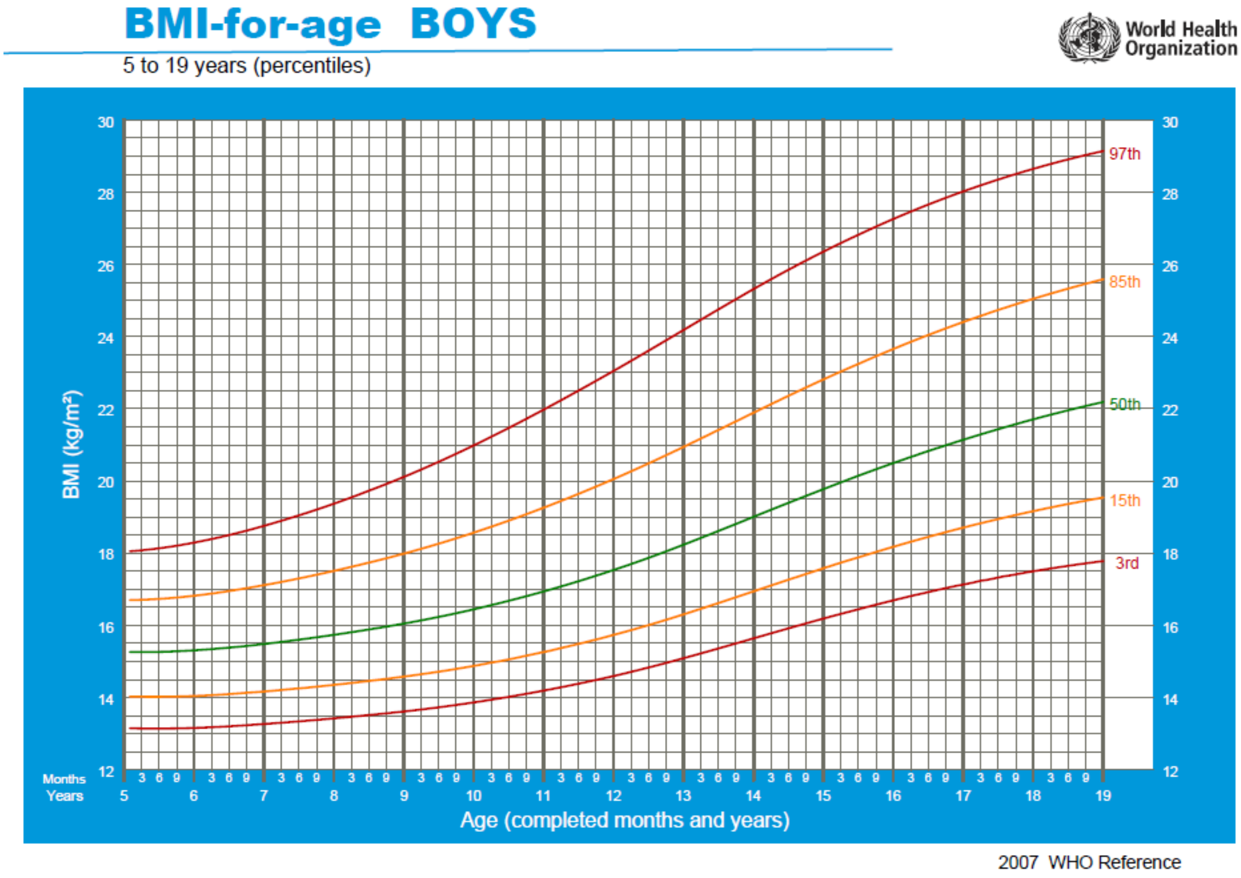

<u>For girls aged 5 through 19 years:</u>

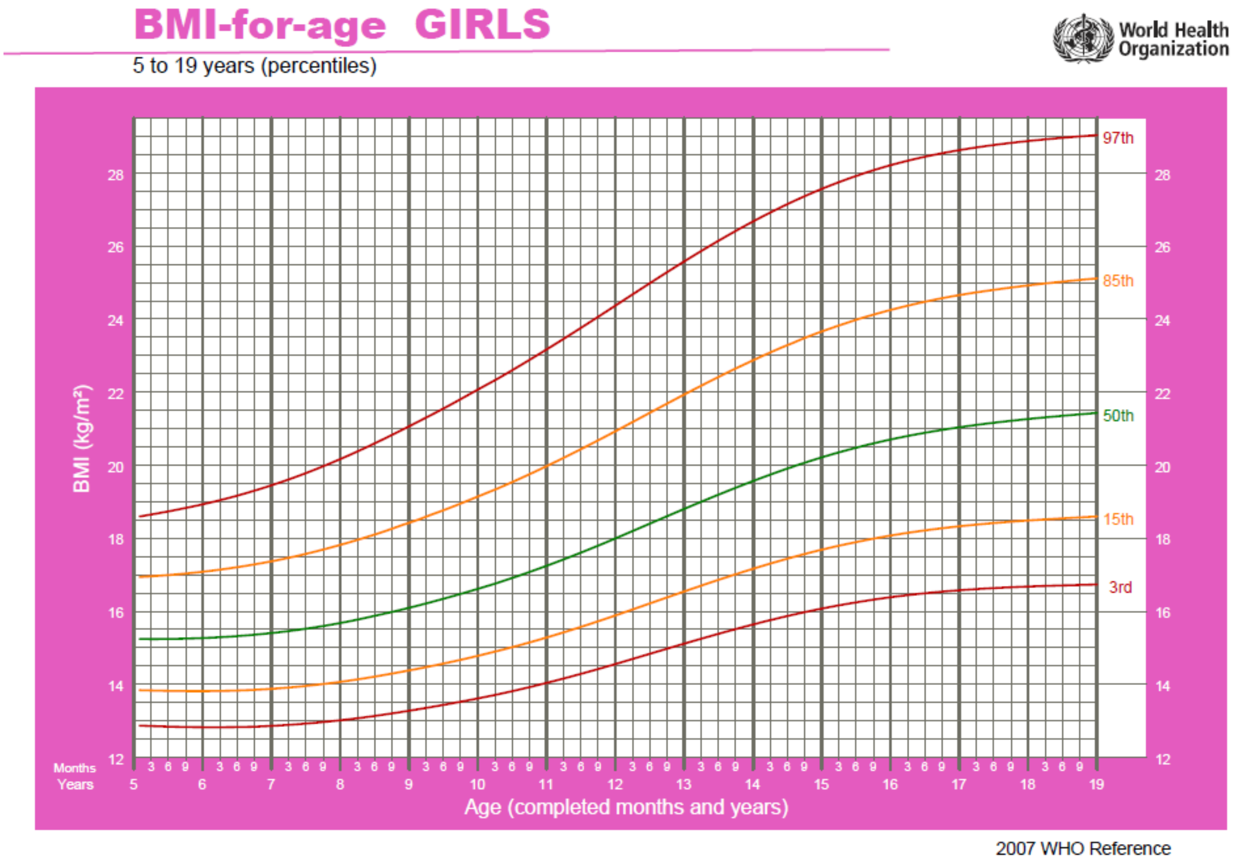

10.3. APPENDIX 3: Contraceptive Guidance

Woman of Childbearing Potential (WOCBP)

Females of childbearing potential are those who are considered fertile following menarche and until becoming postmenopausal unless permanently sterile (see below). If fertility is unclear (eg, amenorrhea in adolescents or athletes) and a menstrual cycle cannot be confirmed before first dose of study treatment, additional evaluation should be considered.

Women in the following categories are not considered WOCBP:

1. Premenarchal
2. Premenopausal, surgically sterile female with 1 of the following:
    - Documented complete hysterectomy
    - Documented surgical sterilization

For individuals with permanent infertility due to an alternate medical cause other than the above, (eg, Mullerian agenesis, androgen insensitivity), investigator discretion should be applied in determining study entry.

Note: Documentation can come from the study site personnel's review of the participant's medical records, medical examination, or medical history interview.

Contraception Guidance:

A highly effective method of birth control is defined as one which results in a low failure rate (eg, less than 1% per year) when used consistently and correctly, such as implants, injectables, combined oral contraceptives, some intrauterine devices, sexual abstinence or vasectomized partner. For participants using a hormonal contraceptive method, information regarding the product under evaluation and its potential effect on the contraceptive should be addressed.

Adequate female contraception is defined as consistent and correct use of an FDA-approved contraceptive method in accordance with the product label. For example:

- Barrier method (such as condoms, diaphragm, or cervical cap) used in conjunction with spermicide
- Intrauterine device
- Prescription hormonal contraceptive taken or administered via oral (pill), transdermal (patch), subdermal, or IM route
- Sterilization of a female participant's monogamous male partner prior to entry into the study

Note that periodic abstinence (eg, calendar, ovulation, symptothermal, postovulation methods) and withdrawal are not acceptable methods of contraception.

10.4. APPENDIX 4: Adverse Event of Special Interest Terms

Investigators should report all events which fall into the following categories as an AESI per the reporting processes specified in Section 7.5.5. The following AESIs are medical concepts that may be related to COVID-19 or are of interest in COVID-19 vaccine safety surveillance. Even if the events below occur in the setting of a COVID infection, the event should still be reported as an AESI if it is one of the medical concepts described in Table 17.

Table 17: Medical Concepts that Qualify as Adverse Events of Special Interest

**Anosmia, Ageusia**	• New onset COVID associated or idiopathic events without other etiology excluding congenital etiologies or trauma
**Subacute thyroiditis**	• Including but not limited to events of atrophic thyroiditis, autoimmune thyroiditis, immune-mediated thyroiditis, silent thyroiditis, thyrotoxicosis and thyroiditis
**Acute pancreatitis**	• Including but not limited to events of autoimmune pancreatitis, immune-mediated pancreatitis, ischemic pancreatitis, edematous pancreatitis, pancreatitis, acute pancreatitis, hemorrhagic pancreatitis, necrotizing pancreatitis, viral pancreatitis, and subacute pancreatitis<br>• Excluding known etiologic causes of pancreatitis (alcohol, gallstones, trauma, recent invasive procedures)
**Appendicitis**	• Include any event of appendicitis
**Rhabdomyolysis**	• New onset rhabdomyolysis without known etiology such as excessive exercise or trauma
**Acute respiratory distress syndrome**	• Including but not limited to new events of ARDS and respiratory failure
**Coagulation disorders**	• Including but not limited to thromboembolic and bleeding disorders, disseminated intravascular coagulation, pulmonary embolism, deep vein thrombosis
**Acute cardiovascular injury**	• Including but not limited to myocarditis, pericarditis, microangiopathy, coronary artery disease, arrhythmia, stress cardiomyopathy, heart failure, or acute myocardial infarction
**Acute kidney injury**	• Include events with idiopathic or autoimmune etiologies<br>• Exclude events with clear alternate etiology (trauma, infection, tumor, or iatrogenic causes such as medications or radiocontrast etc.)<br>• Include all cases that meet the following criteria<br>– Increase in serum creatinine by ≥0.3 mg/dl (≥26.5 µmol/l) within 48 hours;<br>OR<br>– Increase in serum creatinine to ≥1.5 times baseline, known or presumed to have occurred within prior 7 days<br>OR<br>– Urine volume ≤0.5 ml/kg/hour for 6 hours
**Acute liver injury**	• Include events with idiopathic or autoimmune etiologies<br>• Exclude events with clear alternate etiology (trauma, infection, tumor, etc.)

	• Include all cases that meet the following criteria<br>– 3-fold elevation above the upper normal limit for ALT or AST<br>OR<br>– >2-fold elevation above the upper normal limit for total serum bilirubin or GGT or ALP
**Dermatologic findings**	• Chilblain-like lesions<br>• Single organ cutaneous vasculitis<br>• Erythema multiforme<br>• Bullous rashes<br>• Severe cutaneous ARs including but not limited to: SJS, TEN, DRESS, and fixed drug eruptions
**Multisystem inflammatory disorders**	• MIS-A<br>• MIS-C<br>• Kawasaki's disease
**Thrombocytopenia**	• Platelet counts <150 x10^9<br>• Including but not limited to immune thrombocytopenia, platelet production decreased, thrombocytopenia, thrombocytopenic purpura, thrombotic thrombocytopenic purpura, or HELLP syndrome
**Acute aseptic arthritis**	• New onset aseptic arthritis without clear alternate etiology (eg, gout, osteoarthritis, and trauma)
**New onset of or worsening of neurologic disease**	• Including but not limited to:<br>– Guillain-Barre Syndrome<br>– ADEM<br>– Peripheral facial nerve palsy (Bell's palsy)<br>– Transverse myelitis<br>– Encephalitis/encephalomyelitis<br>– Aseptic meningitis<br>– Febrile seizures<br>– Generalized seizures/convulsions<br>– Stroke (Hemorrhagic and nonhemorrhagic)<br>– Narcolepsy
**Anaphylaxis**	• Anaphylaxis as defined PP.<br>• Follow reporting procedures in protocol Section 7.5.5
**Other syndromes**	• Fibromyalgia<br>• Postural orthostatic tachycardia syndrome<br>• Chronic fatigue syndrome (includes myalgic encephalomyelitis and post viral fatigue syndrome)<br>• Myasthenia gravis

Abbreviations: ADEM = acute disseminated encephalomyelitis; ALP = alkaline phosphatase; ALT = alanine transaminase; AST = aspartate aminotransferase; AR = adverse reaction; ARDS = acute respiratory distress syndrome; COVID = coronavirus disease; DRESS = drug reaction with eosinophilia and systemic symptoms; GGT = gamma-glutamyl transpeptidase; HELLP = hemolysis, elevated liver enzymes, and low platelets; MIS-A = multisystem inflammatory syndrome in adults; MIS-C = multisystem inflammatory syndrome in children; SJS = Stevens-Johnson syndrome; TEN = toxic epidermal necrolysis.

10.5. APPENDIX 5: Protocol Amendment History

Amendment 5, 11 Oct 2022

This amendment is considered to be substantial based on the criteria set forth in Article 10(a) of Directive 2001/20/EC of the European Parliament and the Council of the European Union.

Main Rationale for the Amendment:

To add an open-label Part 3 of the study with 50 µg of a bivalent mRNA-1273.222 vaccine given as 2 primary doses, 6 months apart, in COVID-19 vaccine-naïve participants. This amendment is expected to generate safety and immunogenicity data on 50 µg mRNA-1273.222 in the 12 to <18 years old adolescent population.

Summary of Major Changes in Protocol Amendment 5:

The Summary of Changes table provided below describes the major changes made in Protocol Amendment 5 relative to Protocol Amendment 4, including the sections modified and the corresponding rationales. The synopsis of Protocol Amendment 5 has been modified to correspond to changes in the body of the protocol. Formatting edits were introduced throughout the protocol to accommodate the recent update to the Sponsor's style guide. Literature references were updated based on changes in cited content. Minor copy edits and administrative updates were made throughout the protocol to align with new content as well as for clarity, readability, and/or accuracy.

Section Number and Name	Description of Change	Brief Rationale
Section 1.1 Study Rationale	Updated background text with recent epidemiology information, potential complications due to myocarditis, pericarditis, MIS-C, and Long COVID in the pediatric population, information on variants of concern, updates on ongoing studies, updates of findings from Study P301, Granting of EUA by FDA for adolescent population, and objectives of Part 3 of study with mRNA-1273.222. Additional information is described on the development of monovalent and bivalent vaccine candidates mRNA-1273.214 and mRNA-1273.222.	Updated to reflect COVID-19 epidemiology and pathology information in the younger age groups, updated clinical studies, changes in COVID-19 vaccine regulations, and the rationale for the new Part 3 of the study.
Section 1.2 Background and Overview	Added information on the emergence of new Omicron variants, the authorization of mRNA-1273 under emergency use in adolescents, 12 to <18 years of age and on molecular characteristics and approval status of bivalent mRNA-1273.222.	Updated to reflect the Sponsor's activities around modification of mRNA-1273 vaccine against emerging Omicron variants.
Section 1.2.1 Nonclinical Studies	Added information on nonclinical studies in monovalent and bivalent vaccines.	Evidence of protection of mRNA-1273 vaccines against different SARS-CoV-2 variants.

Section Number and Name	Description of Change	Brief Rationale
Section 1.2.2 Clinical Studies	Updated with additional studies in the adult and pediatric population on safety and immunogenicity of mRNA-1273 against new variants. Updates with interim results of current study and from Study mRNA-1273-P205 on the effectiveness of bivalent booster vaccine mRNA-1273.214 as well as safety findings<br>Report of emerging case of myocarditis and/or pericarditis in the study.	Updated to reflect emerging effectiveness and safety data from clinical studies in adult and pediatric populations.
Section 2 Objectives and Endpoints; Section 3 Study Design; Section 8 Statistical Analysis Plan; Section 10.1 Appendix 1: Schedule of Assessments	Modifications to primary objectives and endpoints, secondary endpoints, statistical hypothesis, power and sample size, analysis, and procedures in Part 2 of the study.<br>Clarified that immunogenicity<br>hypothesis testing will not be performed for Part 2. Discontinuation of enrollment in Part 1C-2 and Part 2 of the study.<br>Clarified that Convalescent Visits are applicable to Part 1A and 1B only.	Updated to reflect adjustments made due to small sample size for Part 2 of the study and the implications of adding Part 3 on the conduct of Part 2 of the study.<br>Removed Convalescent visit because data/samples collected from this visit are not analyzed and to minimize the additional visits which could be burdensome for some participants.
Section 1.1 Study Rationale, Section 1.2 Background and Overview, Section 1.3.1 Potential Benefits from Participation, Section 1.3.2 Risks to Study Participants and Risk Mitigation; Section 2 Objectives and Endpoints, Section 3.1.3 Part 3 Study Design, Section 3.2 Scientific Rationale for Study Design; Section 3.4 End-of-Study Definition; Section 4.1 Eligibility Criteria (Part 1A, Part 2, and Part 3), Section 5.4 Study Treatment Compliance, Section 5.5.4; Concomitant Medications and Vaccines that May Lead to the Elimination of a Participant from Per-Protocol Analyses; Section 7 Study Assessments and Procedures, Section 8 Statistical Analysis Plan, Section 8.4 Analysis Sets; Section 8.5.7 Part 2 Analysis; Section 8.5.8 Part 3 Analysis; Section 10.1 Appendix 1: Schedule of	Updated potential benefits to include effectiveness of mRNA-1273.222 against disease caused by variants of concern.<br>Updated risks to study participants with evaluation of risks related to myocarditis and pericarditis.<br>Removed BD from Part 2 and convalescent visits from Part 1C-1, Part 1C-2, and Part 2 of the study.<br>Added Part 3 where participants will get a 50 µg mRNA-1273.222 administered in 2 doses 6 months apart.<br>Generalized language around End-of-Study definition.<br>Updated exclusion criteria to include history of SARS-CoV-2 infection or exposure to someone with COVID-19 within 90 days prior to receipt of the IP for participants in Part 3. Clarified Exclusion criteria in Part 1C2 that	Updates to risks and benefits of the mRNA-1273 program based on development of bivalent vaccine and reports of myocarditis and pericarditis.<br>Addition of a new part to study to test the safety and effectiveness of a bivalent mRNA against ancestral strain and Omicron in the young adolescent population.

Section Number and Name	Description of Change	Brief Rationale
Assessments, Section 10.2.8 Protocol Deviations	monovalent COVID-19 primary series vaccines as well as authorized/approved bivalent vaccine against SARS-CoV-2 as 4th booster are not exclusionary. Added a note to clarify that participants in Part 1C2 and Part 2 planning to receive updated bivalent vaccine outside of study may continue in the study.<br>On Section "exceptions to concomitant medications and vaccines that may lead to the elimination of a participant from per-protocol analyses" it was clarified that exclusion/withdrawal of participants who received COVID-19 vaccine apply only to participants in Part 1A.<br>Updated study assessments and procedures to reflect updates to SoA for Part 1C-1, Part 1C-2, Part 2, and Part 3 of the study.<br>Added PP Immunogenicity Subset for Part 3 in the Analysis Sets summary table.<br>Clarified that confirmed symptomatic COVID-19 cases will be recorded as MAAEs.	
Section 7.5.5.1 Acute Myocarditis and/or Pericarditis	Clarifications on the diagnostic evaluation, management, and reporting of myocarditis and/or pericarditis for the investigator.	To enhance investigator understanding of practice related to mRNA-1273 and mRNA-1273.222-associated AESI.
Section 10.3 Appendix 3: Contraceptive Guidance	Updated definition of highly effective birth control methods using definition containing ICH language.	To ensure appropriate birth control method, accepted by health authorities outside the USA, is used in the study.

Abbreviations: AESI = adverse event of special interest; AR = adverse reaction; bAb = binding antibody; CBER = Center for Biologics Evaluation and Research; D = day; EUA = Emergency Use Authorization; ICH = International Council for Harmonisation; IP = investigational product; MAAE = medically attended adverse event; mITT = modified intent-to-treat; nAb = neutralizing antibody; SAE = serious adverse event; RT-PCR = reverse transcriptase polymerase chain reaction; SARS-CoV2 = Severe Acute Respiratory Syndrome coronavirus 2; SoA = Schedule of Assessments.

Amendment 4, 25 Jan 2022

Main Rationale for the Amendment:

To add 2 additional arms in the study, the 50 µg primary series and BD and heterologous boosting using the 50-µg dose. This amendment is expected to generate immunogenicity data on a lower primary series dose of 50 µg and heterologous boosting in the adolescent population.

Summary of Major Changes in Protocol Amendment 4:

The Summary of Changes table provided below describes the major changes made in Protocol Amendment 4 relative to Protocol Amendment 3, including the sections modified and the corresponding rationales. The synopsis of Protocol Amendment 4 has been modified to correspond to changes in the body of the protocol. Minor copy edits and administrative updates were made throughout the protocol to align with new content as well as for clarity, readability, and/or accuracy.

Section Number and Name	Description of Change	Brief Rationale
Section 1.1. (Study Rationale), Section 1.3.2. (Risks to Study Participants and Risk Mitigation), Section 3.2. (Scientific Rationale for Study Design), Section 3.3. (Justification for Dose, Control Product, and Choice of Study Population)	Added information about 50 µg dose levels and heterologous or mixed series coronavirus disease 2019 (COVID-19) vaccines in adults	Updated to reflect the change in data
Section 1.2.2. (Clinical Studies)	Added information about Study mRNA-1273-P205 which is evaluating boosters for variants	Updated to reflect updated clinical studies
Section 1.3.3. (Overall Benefit/Risk Conclusion), Section 2. (Objectives and Endpoints), Section 3.1. (General Design), Section 3.1.1.4. (Part 1C-2, Heterologous Booster Phase), Section 3.2. (Scientific Rationale for Study Design), Section 3.4. (End-of-Study Definition) Section 4.4 (Study Eligibility Criteria [Part 1C-2 – Heterologous Booster Dose]), Section 5.4. (Study Treatment Compliance), Section 8.3.1.2.2. (Part 1C-2 – Heterologous Booster Phase), Section 8.5.6.2. (Part 1C-2 – Heterologous Booster Phase Analysis), Section 10.1. (APPENDIX 1: Schedule of Assessments; Table 13), Section 10.2.8. (Protocol Deviations)	Addition of Part 1C-2	Added to assess the heterologous or mixed series COVID-19 vaccine in this population
Section 1.3.3. (Overall Benefit/Risk Conclusion), Section 2. (Objectives and Endpoints), Section 3.1. (General Design), Section 3.1.2. (Part 2 Study	Added Part 2 where participants will get a 50 µg primary series and BD	Changes made to test a lower dosing regimen to test safety and efficacy

Section Number and Name	Description of Change	Brief Rationale
Design), Section 3.2. (Scientific Rationale for Study Design), Section 3.3. (Justification for Dose, Control Product, and Choice of Study Population), Section 3.4. (End-of-Study Definition), Section 4.1. (Eligibility Criteria [Part 1A and Part 2]), Section 4.6. (Screen Failures [Part 1A: Blinded Phase and Part 2]), Section 5.1. (Investigational Product Administered), Section 5.3.1 (Administration of Study Vaccine) Section 5.3.2. (Administration of Study Vaccine), Section 5.3.4. (Study Vaccine Packaging and Labeling), Section 5.4. (Study Treatment Compliance), Section 6.4. (Study Pause Rules), Section 7. (Study Assessments and Procedures), Section 7.1. (Safety Assessments and Procedures), Section 7.1.1. (Use of Electronic Diaries), Section 7.1.2. (Safety Telephone Calls), Section 7.1.4. (Vital Sign Measurements), Section 7.1.5. (Physical Examinations), Section 7.1.6. (Assessment for SARS-CoV-2 Infection), Section 7.2. (Immunogenicity Assessments), Section 7.5.7. (Eliciting and Documenting Adverse Events), Section 8.2.2. (Part 2), Section 8.2.2.1. (Justification of Using Immunogenicity Subset of Adults (≥18 Years of Age) from Study P301 as the Comparator for Part 2 Hypothesis testing), Section 8.3.2. (Part 2), Section 8.5.7. (Part 2 Analysis), Section 8.7.1. (Interim Analyses), Section 10.1. (APPENDIX 1: Schedule of Assessments, Table 14), Section 10.2.8. (Protocol Deviations)		
Section 5.3.4. (Study Vaccine Packaging and Labeling)	Updated vial information for investigational product for Part 1C and Part 2	Changes made to reflect current investigational product availability
Section 7.1.6. (Assessment for SARS-CoV-2 Infection)	Added information about restrictions due to Severe Acute Respiratory Syndrome coronavirus 2 related surges	Changes made to allow for flexibility due to COVID-19 related restrictions
Section 7.10. (Biomarkers)	Changed that biomarkers are not evaluated in the study to that they	Changes made to allow for further testing as the landscape changes

Section Number and Name	Description of Change	Brief Rationale
	will be stored for future assessment	
Section 8.6. (Multiplicity Adjustment)	Added section to define the multiplicity adjustment with the multiple parts	Changes made to clarify procedure to preserve the family-wise type I error rate

Amendment 3, 04 Nov 2021

Main Rationale for the Amendment:

To provide a 50 µg BD of mRNA-1273 to participants. This change is prompted by new interim data from an ongoing Moderna Phase 2 study, mRNA-1273-P201 (Study P201). Adult participants in Study mRNA-1273-P201 received 2 doses of either 50 µg or 100 µg of mRNA-1273 and were administered a 50 µg booster of mRNA-1273 6 to 8 months after the second dose. Participants in mRNA-1273-P201 who received the BD, demonstrated enhanced immune responses to SARS-CoV-2 compared to preboost levels and met the noninferiority criteria stipulated in the US FDA Guidance on EUA for Vaccines to Prevent COVID-19. Additionally, no new safety signals emerged upon administration of the BD in Study P201. Based on cumulative evidence, the benefit-risk profile of a BD of mRNA-1273 is favorable, particularly in light of increasing breakthrough disease with the emergence of the Delta variant. Providing the option for a BD to all eligible participants currently enrolled is expected to generate valuable booster data in the adolescent population.

Summary of Major Changes in Protocol Amendment 3:

The Summary of Changes table provided below describes the major changes made in Protocol Amendment 3 relative to Protocol Amendment 2, including the sections modified and the corresponding rationales. The synopsis of Protocol Amendment 3 has been modified to correspond to changes in the body of the protocol. Minor grammar and formatting corrections were made throughout the document to enhance clarity and readability (which did not affect the conduct of the study).

Section Number and Name	Description of Change	Brief Rationale
Title Page, Signature page, Protocol Amendment Summary of Changes, and Header	Updated the protocol version and date.	Updated to reflect the new version and date.
Synopsis, Section 4 (Study Population [Part A: Blinded Phase and Part B: Open-Label Observational Phase])	Updated the estimated date last participant completed and total number of sites.	Changes made to reflect the confirmation of study sites and updated participant timeline.
Synopsis, Section 3.1 (General Design)	Extended the study duration.	Changes made to reflect the addition of a new study part (Part C).
Synopsis, Section 2 (Objectives and Endpoints)	Added exploratory objectives for Part B.	Changes made to reflect the planned analyses.
	Added primary, key secondary, and exploratory objectives for Part C.	Changes made to reflect the addition of a new study part (Part C).

Section Number and Name	Description of Change	Brief Rationale
Synopsis, Section 1.3.2 (Risks to Study Participants and Risk Mitigation), Section 1.3.3 (Overall Benefit/Risk Conclusion), Section 2 (Objectives and Endpoints), Section 3.1 (General Design), Section 3.1.3 (Part C, Booster Dose Phase), Section 3.2 (Scientific Rationale for Study Design), Section 3.3 (Justification for Dose, Control Product, and Choice of Study Population), Section 4.3 (Study Eligibility Criteria [Part C]), Section 5.1 (Investigational Product Administered), Section 5.3.1 (Preparation of Study Vaccine for Injection), Section 5.3.2 (Administration of Study Vaccine), Section 5.3.4 (Study Vaccine Packaging and Labeling), Section 5.4 (Study Treatment Compliance), Section 6.4 (Study Pause Rules), Section 7 (Study Assessments and Procedures), Section 7.1 (Safety Assessments and Procedures), Section 7.1.1 (Use of Electronic Diaries), Section 8.1 (Blinding and Responsibility for Analyses), Section 8.2 (Statistical Hypothesis), Section 8.3 (Power and Sample Size), Section 8.5 (Statistical Methods), Section 10.1 (APPENDIX 1: Schedule of Events; Table 10), Section 10.2.8 (Protocol Deviations)	Part C is being added to Study P203.<br>Part C evaluates the safety and immunogenicity of a third BD of 50 µg of mRNA-1273 in adolescents who have previously received 2 doses of mRNA-1273 as a primary series.	Changes made to reflect the addition of a new study part (Part C) to continue to evaluate the benefit-risk profile of a BD of mRNA-1273.
Synopsis, Section 3.1.1 (Part A, the Blinded Phase), Section 3.1.2 (Part B, the Open-label Observational Phase), Section 3.2 (Scientific Rationale for Study Design), Section 7.1.6 (Assessment for SARS-CoV-2 Infection), Section 7.3 (Exploratory Assessments and Biomarkers), Section 7.4.1 (Vaccine Effectiveness Assessments), Section 7.4.2 (Surveillance for COVID-19 Symptoms), Section 7.4.3 (Follow-up/Convalescent Period After Diagnosis with COVID-19), Section 10.1 (APPENDIX 1: Schedule of Assessments, Table 7, Table 8, Table 9, Table 10)	Add nasal swab to nasopharyngeal at all timepoints.	To align with the original unblinding plan.

Section Number and Name	Description of Change	Brief Rationale
Synopsis, Section 5.3.1 (Preparation of Study Vaccine for Injection), Section 5.3.2 (Administration of Study Vaccine), Section 5.3.4 (Study Vaccine Packaging and Labeling)	Part C language was added to discuss the difference in volume.	Changes made to reflect different vaccine volumes given during the primary series and the boost.
Synopsis, Section 7.3 (Exploratory Assessments and Biomarkers)	Addition of Exploratory Assessments and Biomarkers.	To align language with other clinical studies.
Synopsis, Section 8.5.5 (Long-term Analysis [including Part B]), Section 8.5.6 (Booster Phase Analysis [Part C])	Updated long-term analysis and Booster Phase analysis.	Changes made to reflect the addition of a BD.
Synopsis, Section 8.6.1 (Interim Analyses)	Addition of booster IAs.	Changes made to reflect the addition of a BD.
Section 1.1 (Study Rationale), Section 1.2.2 (Clinical Studies)	Added updated clinical information of mRNA-1273.	Changes made to reflect new and ongoing clinical data in the current and other studies.
Section (5.3.5) Study Vaccine Storage	Updated storage conditions to follow the investigational product label.	Changes made to reflect differences in storage conditions between different vial volumes.
Section 5.5.4 (Concomitant Medications and Vaccines that May Lead to the Elimination of a Participant from Per-Protocol Analyses)	Added receipt or planned receipt of a nonstudy COVID-19 would require withdrawal from the study.	To clarify that participants could not get other COVID-19 vaccines.
Section 6.1.1 (Individual Participant Criteria for Delay of Study Vaccination)	Added investigator judgment to rescheduling of visits.	To allow for flexibility of visits.
Section 7.1.6 (Assessment for SARS-CoV-2 Infection), Section 7.4.2 (Surveillance for COVID-19 Symptoms), Section 7.4.3 (Follow-up/Convalescent Period After Diagnosis with COVID-19), Section 10.1 (APPENDIX 1 Schedule of Events; Table 9)	Added nasal swab for samples for SARS-CoV-2.	To allow more flexibility for participants.
Section 8.5.3 (Immunogenicity Analysis)	Added the primary immunogenicity analysis is performed in Part A and Part C.	To clarify that only Part A and Part C will have primary immunogenicity analyses.

Amendment 2, 27 Jul 2021

Main Rationale for the Amendment:

The main rationale for this amendment is to increase awareness of possible cases and to add a case definition for myocarditis and pericarditis as well as guidance for reporting and assessing suspected cases for this study, given the recent reports in the EUA experience of cases with a temporal association between COVID-19 mRNA vaccine administration and signs and symptoms of myocarditis and pericarditis.

Summary of Major Changes in Protocol Amendment 2:

The Summary of Changes table provided below describes the major changes made in Protocol Amendment 2 relative to Protocol Amendment 1, including the sections modified and the corresponding rationales. The synopsis of Protocol Amendment 2 has been modified to correspond to changes in the body of the protocol. Minor grammar and formatting corrections were made throughout the document to enhance clarity and readability, and new references were added in support of the definition of myocarditis and pericarditis (which did not affect the conduct of the study).

Section Number and Name	Description of Change	Brief Rationale
Title Page, Signature page, Protocol Amendment Summary of Changes, and Header	• Updated the protocol version and date.<br>• Added Protocol Amendment Summary of Changes for Amendment 2.	Updated to reflect the new version and date.
Section 1.3.2 (Risks to Study Participation and Risk Mitigation)	Added paragraph on rare reports of myocarditis and pericarditis occurring after vaccination with Moderna coronavirus disease 2019 (COVID-19) vaccine under EUA in adults 18 years of age and older.	To reflect addendum made to Investigator's Brochure.
Section 7.4.5 (Adverse Events of Special Interest)	Added CDC case definitions for myocarditis and pericarditis.	To provide guidance to the investigators regarding assessing and reporting myocarditis and pericarditis for this study population.
Section 7.5.2 (Independent Cardiac Event Adjudication Committee)	Added new section.	To describe the proposed mechanism to assess risk of myocarditis and pericarditis in the study population (to address CBER request to describe how the risk of myocarditis and pericarditis will be assessed in the study population receiving mRNA-1273).
Section 8.1 (Blinding and Responsibility for Analyses) and Section 8.6.1 (Interim Analysis)	Updated these sections to align with the revised SAP V2.0, which states the IA will be performed for immunogenicity, safety, and efficacy.	To align with the updated SAP.
Section 9 (References)	Updated reference list.	To support the text added to the protocol for myocarditis and pericarditis.
Appendix 1, Schedule of Assessments, Table 7, and Table 9	Increased D209 Participant Decision Visit window to -56/+56.	Minus window expanded to accommodate participants whose D209 Visit is expected to occur after EUA is granted in this age group.
Appendix 1, Schedule of Assessments, Table 9	New footnote was added.	To clarify that D209/Participant Decision Visit may occur over multiple visits but must be completed within 7 days.

Section Number and Name	Description of Change	Brief Rationale
Section 10.4 (Appendix 4: Adverse Event of Special Interest Terms)	Added new appendix to the protocol.	To include AESI list in the protocol instead of as a separate document.
Section 10.5 (Appendix 5: Protocol Amendment History)	Moved the Summary of Changes for Protocol Amendment 1 to Appendix 5: Protocol Amendment History.	To reflect the Summary of Changes for the new version of the amendment.

Amendment 1, 23 Mar 2021

Main Rationale for the Amendment:

The primary rationales for this amendment are as follows:

- To update the primary endpoints and null hypotheses (or success criteria) for establishing immunobridging and assumptions for the sample size calculations, as recommended by health authorities.
- Following the authorization of a COVID-19 vaccine under an EUA, this study amendment is designed to transition to Part B, the Open-Label Observational Phase. Transitioning the study to Part B, the Open-label Observational Phase permits all ongoing study participants (a) to be informed of the availability and eligibility criteria of any COVID-19 vaccine made available under an EUA and (b) for EUA-eligible study participants, the opportunity to schedule a study visit to know their original group assignment (placebo versus mRNA-1273).
- Part B, the Open-label Observational Phase also provides the opportunity for study participants who previously received placebo to request to receive 2 doses of the mRNA-1273 vaccine.

The Summary of Changes table provided here describes the major changes made in Protocol Amendment 1 relative to the original protocol, including the sections modified and the corresponding rationales. The synopsis of Protocol Amendment 1 has been modified to correspond to changes in the body of the protocol.

Summary of Major Changes in Protocol Amendment 1:

Section Number and Name	Description of Change	Brief Rationale
Title Page, Protocol Approval Page, Headers, Protocol Amendment Summary of Changes	Updated the protocol version and date.	To reflect the new version and date of the protocol.
Protocol Synopsis – Objectives and Section 2 (Objectives and Endpoints)	The primary endpoints were redefined. Secondary and exploratory endpoints were refined. An additional secondary objective and corresponding endpoint were added. A new exploratory objective and endpoint were added.	If an accepted threshold of protection is not available, using coprimary endpoints based on nAb geometric mean and SRR (added) in the noninferiority test was recommended by health authorities, as it is considered standard in immunobridging. A secondary objective/endpoint to evaluate asymptomatic SARS-CoV-2 infection was added to be separate

Section Number and Name	Description of Change	Brief Rationale
		from the objective/endpoint of SARS-CoV-2 infection in participants with SARS-CoV-2 negative at baseline. An exploratory objective/endpoint was added to evaluate asymptomatic SARS-CoV-2 infection in participants seropositive at baseline.
Protocol Synopsis – Overall Study Design, Section 3.1 (General Study Design), Section 3.1.1 (Part A, the Blinded Phase), Section 3.1.2 (Part B, the Open-label Observational Phase), and Section 7 (Study Assessments and Procedures)	The study design was updated to describe the updated crossover design of the study, including Part A (the Blinded Phase) and Part B (the Open-label Observational Phase).	The addition of the crossover design provides the opportunity for study participants to be informed regarding the EUA of mRNA-1273 for any persons under the age of 18 years, be unblinded to their original assignment (mRNA-1273 or placebo), and for those who previously received placebo to actively request to receive 2 doses of mRNA-1273 (100 µg) vaccine.
Protocol Synopsis – Study Duration and Section 3.1.1 (Part A, the Blinded Phase)	The study duration was clarified to reflect the differing study duration for participants who were initially randomly assigned to mRNA-1273 versus patients who were initially randomized to placebo but who opt to receive mRNA-1273 after unblinding.	The addition of the crossover design was one of the primary purposes of this amendment.
Protocol Synopsis – Inclusion Criteria and Section 4.1.1 (Inclusion Criteria)	Updated to allow abstinence as a contraception option and remove the criteria that participants not be currently breastfeeding.	Updated to be consistent with the current standard of care in the USA.
Protocol Synopsis – Exclusion Criteria and Section 4.1.2 (Exclusion Criteria)	Updated to remove exclusion of participants with a known history of SARS-CoV-2 infection or contact with a confirmed case of SARS-CoV-2 infection.	Updated to be consistent with the current standard of care in the USA, and to address the increasing prevalence of SARS-CoV-2 infection and make the study more consistent with the "real world" (EUA) setting.
Protocol Synopsis – Study Eligibility Criteria (Part B) and Section 4.2 (Study Eligibility Criteria, Part B)	Section added for Part B of the study to specify that participants participating in Part B must have been previously enrolled in the mRNA-1273-P203 study; and that female participants of childbearing potential may enroll in Part B if they have negative pregnancy tests at OL-Day 1 and OL-Day 29.	To establish separate eligibility criteria for Part B of the study.
Protocol Synopsis – Procedures and Assessments (Safety Assessments) and Section 7.1 (Safety Assessments and Procedures)	Updated to clarify the collection periods for ARs, AEs leading to discontinuation from dosing and/or	To clarify based on the new crossover design of the study.

Section Number and Name	Description of Change	Brief Rationale
	study participation, MAAEs, SAEs, and AESIs.	
Protocol Synopsis – Procedures and Assessments (Immunogenicity Assessments) and Section 7.2 (Immunogenicity Assessments)	Updated to note the assessments for Part A and Part B of the study, to clarify the analytes to be measured, and to add testing of serum for nAb and bAb against the SARS-CoV-2 S protein.	Revised the assessments and tests plan for the unbinding or Participant Decision Visit and subsequent visits to be used for long-term follow-up analyses.
Protocol Synopsis – Procedures and Assessments (Efficacy Assessments) and Section 7.3.1 (Vaccine Effectiveness Assessments)	Updated to refine the Ab response assessment if an accepted threshold of protection is not available.	To define the coprimary endpoints required for the noninferiority test to establish immunobridging.
Protocol Synopsis – Statistical Methods (Hypothesis Testing) and Section 8.2 (Statistical Hypothesis)	Updated to redefine the study hypotheses based on updates to the coprimary endpoints.	To specify the hypotheses for the updated coprimary endpoints using noninferiority test.
Protocol Synopsis – Statistical Methods (Power and Sample Size) and Section 8.3 (Power and Sample Size)	Updated to reflect increases in the size of the Immunogenicity Subset for the purposes of establishing acceptable noninferiority margins.	To detail the sample size and power calculations for the updated primary endpoints and hypotheses.
Protocol Synopsis – Statistical Methods (Analysis Sets) and Section 8.4 (Analysis Sets)	Added mITT and miTT1 analysis sets.	To define mITT and mITT1 to be used in the sensitivity analyses of secondary efficacy endpoints.
Protocol Synopsis – Statistical Methods (Immunogenicity Analyses) and Section 8.5.3 (Immunogenicity Analyses)	Updated the analyses of the coprimary endpoints.	To describe the analysis methods for the updated coprimary endpoints.
Protocol Synopsis – Statistical Methods (Efficacy Analyses) and Section 8.5.4 (Efficacy Analyses)	Updated to clarify the secondary efficacy analyses.	To clarify the secondary efficacy analyses.
Protocol Synopsis – Statistical Methods (Long-term Analysis) and Section 8.5.5 (Long-term Analysis)	Added section to describe long-term analyses of safety, efficacy, and immunogenicity data to include data collected during Part B of the study.	To describe long-term analyses including Part B data.
Protocol Synopsis – Study Analyses (Interim Analyses) and Section 8.6.1 (Interim Analyses)	Updated to indicate that more than one IA may be performed, and to describe the timepoints at which the analyses will be performed.	To support potential EUA in the adolescent age group.
Section 1.2.2 (Clinical Studies)	Revised section to provide data for ongoing mRNA-1273 studies.	To harmonize section content with current status of development in the program.
Section 1.3.2 (Risks from Study Participation and Their Mitigation), 3.1.1 (Part A, the Blinded Phase), 5.3.2 (Administration of Study Vaccine), 7.1.1 (Use of Electronic Diaries), and 7.1.4 (Vital Sign Measurements)	Updated postIP administration observation period from 60 minutes to 30 minutes.	Pursuant to cross-functional discussion and observation period of 15 minutes under the EUA.

Section Number and Name	Description of Change	Brief Rationale
Section 3.3 (Justification for Dose, Control Product, and Choice of Study Population)	Updated section to include justification for the crossover design.	The addition of the crossover design was one of the primary purposes of this amendment.
Section 5.3.2 (Administration of Study Vaccine)	Updated IP administration to include the IP administered in Part A and Part B of the study.	The addition of the crossover design was one of the primary purposes of this amendment.
Section 6.2 (Discontinuing Study Vaccination)	Updated to remove the allowance for study removal for serology or RT-PCR testing positive for SARS-CoV2 for either Day 1 or for an illness visit.	Updated to be consistent with current standard of care in the US.
Section 6.4 (Study Pause Rules)	Updated to clarify that study pause rules are only applicable to Part A of the study but that participants will be monitored for the events leading to study pause during Part B of the study. Updated to clarify actions to be taken if thresholds for any pause rules are met.<br>Updated to clarify that any Grade 3 or higher AEs meet event criteria.	To clarify safety assessments after crossover.
Section 7.1.1. (Use of Electronic Diaries)	Updated to clarify that eDiaries will only be used in Part A of the study.	The solicited AR profile will be sufficiently demonstrated in Part A; therefore, it is no longer necessary to collect additional data on solicited ARs following injection in the open-label phase.
Section 7.3.1 (Vaccine Effectiveness Assessments)	Updated to further define criteria for SARS-CoV-2 infection.	To clarify the definition of SARS-CoV-2 infection in participants who are SARS-CoV-2 negative at baseline.
Section 7.4.4.1 (Anaphylaxis)	Added section to characterize anaphylaxis and provide reporting requirements.	This text is being added to all mRNA-1273 protocols based on recent reports of anaphylaxis in the postEUA setting.
Section 7.4.5 (Adverse Events of Special Interest)	Updated to define an AESI and provide reporting requirements.	To provide more specific guidance to sites.
Section 7.4.7 (Eliciting and Documenting Adverse Events)	Updated to clarify that solicited ARs will be collected only in Part A, and to define the end-of-study participation for the purposes of collection MAAEs and SAEs.	To provide more specific guidance to sites.
Section 8.1 (Blinding and Responsibility for Analyses)	Updated to describe unblinding procedures relevant to the interim analyses.	To clarify the blinding/unblinding plan and process.

Section Number and Name	Description of Change	Brief Rationale
Section 10.1 (Appendix 1: Schedule of Assessments)	Table 7 title updated to reflect Part A of the study.<br>Updated study visit day 209 to "Day 209/Participant Decision Visit"<br>Increased visit window for Day 209 to ±56 days.<br>Increased visit window for the safety follow-ups (Day 223-Day 363) to ±3 days.<br>Row added for revised informed consent/assent form.<br>Added note to clarify the SoA to be followed after unblinding for participants who previously received mRNA-1273.<br>Added footnote for time frame for Participant Decision Clinic Visit.<br>Revised footnote to indicate that eDiary recording will start approximately 30 minutes after injection.	To clarify as related to crossover design.
	Added table (Table 8) for the Participant Decision Clinic Visit.	To allow for participant unblinding and decision to receive mRNA-1273 if previously randomized to placebo.
	Added new SoA (Table 9) for Part B of the study.	To provide instructions for participants previously randomized to placebo who have elected to receive mRNA-1273 in Part B of the study.
	Added flow chart (Figure 4) between Part A and Part B of the study.	To demonstrate participants' movement between Part A and Part B, and back to Part A.
Section 10.2.8 (Protocol Deviations)	Added guidance for participants' movement between Part A and Part B.	The addition of the crossover design was one of the primary purposes of this amendment.

Abbreviations: AESI = adverse event of special interest; AR = adverse reaction; bAb = binding antibody; CBER = Center for Biologics Evaluation and Research; D = day; eDiary = electronic diary; EUA = Emergency Use Authorization; IP = investigational product; MAAE = medically attended adverse event; mITT = modified intent-to-treat; nAb = neutralizing antibody; SAE = serious adverse event; RT-PCR = reverse transcriptase polymerase chain reaction; SARS-CoV2 = Severe Acute Respiratory Syndrome coronavirus 2; SoA = Schedule of Assessments.

Signature Page for VV-CLIN-003517 v10.0

2nd Approval	Amparo Figueroa<br>Clinical<br>23-Jun-2023 16:10:05 GMT+0000

Signature Page for VV-CLIN-003517 v10.0
