# Supplementary material for: Safety and durability of mRNA-1273–induced SARS-CoV-2 immune responses in adolescents: results from the phase 2/3 TeenCOVE trial
Source: eClinicalMedicine. 2024 Jul 18;74:102720. doi: 10.1016/j.eclinm.2024.102720 (PMC11293523; doi:10.1016/j.eclinm.2024.102720)
Supplement: Moderna mRNA-1273-P203 SAP v5.0_Part 1A [file mmc3.pdf]

ModernaTX, Inc.

Protocol mRNA-1273-P203

A Phase 2/3, Randomized, Observer-Blind, Placebo-Controlled Study to Evaluate the Safety, Reactogenicity, and Effectiveness of mRNA-1273 SARS-CoV-2 Vaccine in Healthy Adolescents 12 to < 18 Years of Age

Statistical Analysis Plan
(Part 1A)

SAP Version 5.0
Version Date of SAP: 30 Jun 2023

Prepared by:

PPD
3575 Quakerbridge Road
Suite 201
Hamilton, NJ 08619

TABLE OF CONTENTS

SUMMARY OF MAJOR CHANGES IN SAP VERSION V

LIST OF ABBREVIATIONS VII

1. INTRODUCTION 8

2. STUDY OBJECTIVES 8

2.1. PRIMARY OBJECTIVE 8
2.2. SECONDARY OBJECTIVES 9
2.3. EXPLORATORY OBJECTIVES 9

3. STUDY ENDPOINTS 10

3.1. PRIMARY ENDPOINTS 10
3.2. SECONDARY ENDPOINTS 11
3.3. EXPLORATORY ENDPOINTS 12

4. STUDY DESIGN 13

4.1. OVERALL STUDY DESIGN 13
4.1.1. Part 1 Study Design 13
4.1.1.1. Part 1A, the Blinded Phase 14
4.1.1.2. Part 1B, the Open-label Observational Phase 15
4.1.1.3. Part 1C-1, the Homologous Booster Dose Phase 16
4.1.1.4. Part 1C-2, Heterologous Booster Phase 17
4.1.2. Part 2 Study Design 18
4.1.3. Part 3 Study Design 19
4.2. STATISTICAL HYPOTHESIS 20
4.3. SAMPLE SIZE AND POWER 22
4.4. RANDOMIZATION 23
4.5. BLINDING AND UNBLINDING 23

5. ANALYSIS POPULATIONS 23

5.1. RANDOMIZATION SET 24
5.2. FULL ANALYSIS SET 24
5.3. IMMUNOGENICITY SUBSET 24
5.4. PER-PROTOCOL (PP) IMMUNOGENICITY SUBSET 24
5.5. MODIFIED INTENT-TO-TREAT (MITT) SET 25
5.6. MODIFIED INTENT-TO-TREAT-1 (MITT1) SET 25
5.7. PER-PROTOCOL (PP) SET FOR EFFICACY 25
5.8. SOLICITED SAFETY SET 26
5.9. SAFETY SET 26

6. STATISTICAL ANALYSIS 26

6.1. GENERAL CONSIDERATIONS 26
6.2. BACKGROUND CHARACTERISTICS 31
6.2.1. Subject Disposition 31
6.2.2. Demographics 33
6.2.3. Medical History 33

6.2.4. Prior and Concomitant Medications *33*
6.2.5. Study Exposure *34*
6.2.6. Major Protocol Deviations *34*
6.2.7. COVID-19 Impact *35*
6.3. SAFETY ANALYSIS 35
6.3.1. Adverse Events *35*
6.3.1.1. Incidence of Adverse Events 36
6.3.1.2. TEAEs by System Organ Class and Preferred Term 36
6.3.1.3. TEAEs by Preferred Term 37
6.3.1.4. TEAEs by System Organ Class, Preferred Term and Severity 37
6.3.2. Solicited Adverse Reactions *37*
6.3.3. Pregnancy Tests *39*
6.3.4. Vital Sign Measurements *39*
6.4. IMMUNOGENICITY ANALYSIS 39
6.4.1. Sampling of the Immunogenicity Subset *40*
6.4.2. Immunogenicity Assessments *41*
6.4.3. Primary Analysis of Antibody-Mediated Immunogenicity Endpoints *41*
6.4.4. Secondary Analysis of Antibody-Mediated Immunogenicity Endpoints *42*
6.5. EFFICACY ANALYSIS 43
6.5.1. Endpoint Definition/Derivation *44*
6.5.1.1. Derivation of SARS-CoV-2 Infection 44
6.5.1.2. Derivation of Asymptomatic SARS-CoV-2 Infection 45
6.5.1.3. Derivation of COVID-19 46
6.5.1.4. Derivation of Secondary Case Definition (CDC case definition) of COVID-1947
6.5.2. Analysis Method *48*
6.5.3. Sensitivity Analysis *48*
6.6. LONG-TERM ANALYSIS (INCLUDING PART 1B) 48
6.7. BOOSTER DOSE ANALYSIS 49
6.8. PART 2 ANALYSIS 49
6.9. PART 3 ANALYSIS 49
6.10. EXPLORATORY ANALYSIS 49
6.10.2. SARS-CoV-2 Exposure and Symptoms *51*
6.11. INTERIM ANALYSIS 51
6.12. FINAL ANALYSIS 52

7. CHANGES FROM PLANNED ANALYSES IN PROTOCOL 53

8. REFERENCES 53

9. LIST OF APPENDICES 54

9.1. APPENDIX A STANDARDS FOR SAFETY AND IMMUNOGENICITY VARIABLE DISPLAY IN TFLS 54
9.2. APPENDIX B ANALYSIS VISIT WINDOWS FOR SAFETY AND IMMUNOGENICITY ANALYSIS 54
9.3. APPENDIX C IMPUTATION RULES FOR MISSING PRIOR/CONCOMITANT MEDICATIONS AND NON-STUDY VACCINATIONS 55
9.4. APPENDIX D IMPUTATION RULES FOR MISSING AE DATES 57
9.5. APPENDIX E SCHEDULE OF ASSESSMENTS (PART 1A, THE BLINDED PHASE; PART 1B OPEN-LABEL OBSERVATIONAL PHASE FOR PARTICIPANTS WHO RECEIVED MRNA-1273 IN PART 1A) 58

9.6. Appendix F Schedule of Assessments (Part 1B, Open-label Observational Phase for Subjects Who Previously Received Placebo)........ 63
9.7. Appendix G Schedule of Assessments (Part 1C-1, Homologous Booster Dose Phase)........ 66
9.8. Appendix H Schedule of Assessments (Part 1C-2, Heterologous Booster Dose Phase)........ 70
9.9. Appendix I Schedule of Assessments (Part 2)........ 74
9.10. Appendix J Schedule of Assessments (Part 3)........ 79

Summary of Major Changes in SAP Version

SAP Version	Section # and Name	Description of Change
V2.0 Based on Protocol Amendment 1	4.1 Overall Study Design	Updated to describe the updated crossover design of the study, including Part A (the Blinded Phase) and Part B (the Open-label Observational Phase).
V2.0	4.2 Statistical Hypothesis	An additional noninferiority success criterion on point estimate for both co-primary endpoints has been added
V2.0 Based on Protocol Amendment 1	5 Analysis Populations	Added mITT and miTT1 analysis sets
V2.0 Based on Protocol Amendment 1	6.6 Long-term Analysis	Newly added to reflect the analysis methods for long-term analysis (including Part B)
V2.0	7 Changes from Planned Analyses in Protocol	Updated to explain the discrepancy between SAP and protocol: an additional noninferiority success criterion on point estimate has been added
V2.0 Based on Protocol Amendment 1	9.6 Appendix F: Schedule of Assessments	Added for Part B, the new study part
V3.0	3.1 Primary Endpoints	Seroresponse definition updated to as a change from below the LLOQ to equal or above 4 x LLOQ, or at least a 4-fold rise if baseline is equal to or above the LLOQ.
V3.0 Based on Protocol Amendment 2	3.1 Primary Endpoints<br>6.3 Safety Analysis	AESIs of myocarditis and/or pericarditis were added (in addition to MIS-C).
V3.0 Based on Protocol Amendment 3	4.1 Overall Study Design	Updated to reflect the addition of a new study part (Part C)
V3.0 Based on Protocol Amendment 3	6.1 General Considerations	Analysis Period for blinded phase
V3.0	6.6.1 Assay Specific Definition of Seroresponse	Definition of assay specific seroesponse is included and updated based on a more conservative approach

V3.0 Based on Protocol Amendment 3	6.7 Booster Dose Analysis (Part C)	Added to explain Booster Dose analysis will be included in separate SAP
V3.0 Based on Protocol Amendment 3	6.9 Interim Analyses	Updated to reflect the IA for Part C
V3.0 Based on Protocol Amendment 3	9.6 Appendix F: Schedule of Assessments	Added OL-D113, OL-D178; Added footnote #6-9; Updated Abbreviations and footnote #2-5;
V3.0 Based on Protocol Amendment 3	9.7 Appendix G: Schedule of Assessments	Newly added to reflect the addition of a new study part (Part C)
V3.0 Based on Protocol Amendment 3	6.1 General Considerations	Added At Risk for Severe COVID-19 (Yes, No) in subgroup analysis
V3.0 Based on Protocol Amendment 3		Add separate SAP for Part B and Part C
V4.0		V4.0 for Part A is the same as V3.0 for Part A; V4.0 for Part B and Part C is in a separate SAP document.
V5.0 Based on Protocol Amendment 5		V5.0 for Part A is the same as V4.0 for Part A; V5.0 for Part 1B, Part 1C, Part 2 and Part 3 is in a separate SAP document.
V5.0 Based on Protocol Amendment 5	4.1.Overall Study Design	Updated to reflect the addition of new study parts (Part 1C-2, Part 2 and Part 3)
V5.0 Based on Protocol Amendment 6	4.1.3. Part 3 Study Design	The target number of enrolled participants was also revised from N of 500 to N of at least 300.

List of Abbreviations

Abbreviation	Definition
AE	adverse event
AR	adverse reaction
BD	booster dose
BMI	body mass index
bAb	binding antibody
CDC	Centers for Disease Control and Prevention
CI	confidence interval
CRO	contract research organization
CSP	clinical study protocol
CSR	clinical study report
DHHS	Department of Health and Human Services
eCRF	electronic case report form
eDiary	electronic diary
ELISA	enzyme-linked immunosorbent assay
EUA	Emergency Use Authorization
FAS	full analysis set
GM	geometric mean
GMFR	geometric mean fold rise
GMT	geometric mean titer
GMR	geometric mean ratio
IgG	immunoglobulin G
IP	investigational product
IRT	interactive response technology
LLOQ	lower limit of quantification
MAAEs	medically-attended adverse events
MedDRA	Medical Dictionary for Regulatory Activities
mRNA	messenger ribonucleic acid
nAb	neutralizing antibody
OL	open-label
PP	per-protocol
PT	preferred term
SAE	serious adverse event
SAP	statistical analysis plan
SAS	Statistical Analysis System
SD	standard deviation
SOC	system organ class
SRR	seroresponse rate
TEAE	treatment-emergent adverse event
ULOQ	upper limit of quantification
VOC	variant of concern
WHO	World Health Organization
WHODD	World Health Organization drug dictionary

1. Introduction

This statistical analysis plan (SAP), which describes the planned analyses for Part 1A, the blinded phase of Study mRNA-1273-P203, is based on the approved clinical study protocol (CSP), Version Amendment 6, dated 22-Jun-2023. The most recent approved electronic case report form (eCRF) Version 16, dated 28-Feb-2023. A separate SAP (Part 1B, Part 1C, Part 2 and Part 3) will be provided for the long-term analysis, the booster dose analysis, the Part 2 analysis, and the Part 3 analysis of Study mRNA-1273-P203. Unless specified otherwise, the study endpoints, statistical hypotheses, analysis methods, and other statistical considerations in this SAP pertains to Part 1A, blinded phase.

In addition to the information presented in the statistical analysis plan section of the protocol (Section 8) which provides the principal features of analyses for this study, this SAP provides statistical analysis details/data derivations. It also documents modifications or additions to the analysis plan that are not “principal” in nature and result from information that was not available at the time of protocol finalization.

Study mRNA-1273-P203 is a Phase 2/3, randomized, observer-blind, placebo-controlled study to evaluate the safety, reactogenicity, and effectiveness of messenger ribonucleic acid (mRNA)-1273 SARS-CoV-2 vaccine in healthy adolescents 12 to <18 years of age.

PPD Biostatistics and programming team, designee of Moderna Biostatistics and Programming department, will perform the statistical analysis of the safety, reactogenicity, and effectiveness data; Statistical Analysis System (SAS) Version 9.4 or higher will be used to generate all statistical outputs (tables, figures, listings, and datasets). The SAP will be finalized and approved prior to the primary analysis clinical database lock and treatment unblinding for the study. If the methods in this SAP differ from the methods described in the protocol, the SAP will prevail.

In this document, subject and participant are used interchangeably; injection of IP, injection, and dose are used interchangeably; vaccination group and treatment group are used interchangeably.

2. Study Objectives

2.1. Primary Objective

The primary objectives are the following:

- To evaluate the safety and reactogenicity of 100 µg of mRNA-1273 vaccine administered in 2 doses 28 days apart.
- To infer efficacy of mRNA-1273 (100 µg, 2 doses 28 days apart), serum Ab responses obtained 28 days after the second injection of mRNA-1273 (Day 57) will be either:
    - Evaluated against an accepted Ab threshold of protection against COVID-19 (if established in Study P301)
    - Compared in primary vaccine response as measured by geometric mean (GM) values of serum Ab and seroresponse rate in P203 with those obtained from young adult recipients (18-25 years of age) of mRNA-1273 in the clinical endpoint efficacy trial (Study P301)

2.2. Secondary Objectives

The secondary objectives are the following:

- To evaluate the persistence of the immune response of mRNA-1273 vaccine (100 µg) administered in 2 doses 28 days apart, as assessed by the level of SARS-CoV-2 S2P-specific bAb through 1 year after dose 2.
- To evaluate the persistence of the immune response of mRNA-1273 vaccine (100 µg) administered in 2 doses 28 days apart, as assessed by the level of nAb through 1 year after dose 2.
- To evaluate the effect of mRNA-1273 on the incidence of SARS-CoV-2 infection compared with the incidence among placebo recipients.
- To evaluate the incidence of asymptomatic SARS-CoV-2 infection after vaccination with mRNA-1273 or placebo.
- To evaluate the incidence of COVID-19 after vaccination with mRNA-1273 or placebo. COVID-19 is defined as clinical symptoms consistent with SARS-CoV-2 infection AND positive RT-PCR for SARS-CoV-2.

2.3. Exploratory Objectives

The exploratory objectives are the following:

- To evaluate the genetic and/or phenotypic relationships of isolated SARS-CoV-2 strains to the vaccine sequence.

- To describe the ratio or profile of specific bAb relative to nAb in serum.
- To characterize the clinical profile and immune responses of participants with COVID-19 or with SARS-CoV-2 infection.
- To evaluate the incidence of asymptomatic SARS-CoV-2 infection after vaccination with mRNA-1273 or placebo in participants with serologic evidence of infection at baseline.

3. Study Endpoints

3.1. Primary Endpoints

The primary safety objective will be evaluated by the following safety endpoints:

- Solicited local and systemic adverse reactions (ARs) through 7 days after each injection.
- Unsolicited adverse events (AEs) through 28 days after each injection.
- Medically-attended AEs (MAAEs) through the entire study period.
- Serious AEs (SAEs) through the entire study period.
- AE of special interest (AESI) of multisystem inflammatory syndrome in children (MIS-C) through the entire study period.
- Vital sign measurements.
- Physical examination findings.

The primary immunogenicity objective will be evaluated by either:

- The proportion of participants with a serum Ab level at Day 57 ≥ an Ab threshold of protection. If an accepted serum Ab threshold of vaccine protection against COVID-19 is available, this analysis will form the basis to infer efficacy.
- The primary vaccine response as measured by GM value of serum antibody level and seroresponse rate from Study P203 vaccine recipients at Day 57 compared with those obtained from young adult vaccine recipients (18-25 years of age) at Day 57 in the clinical endpoint efficacy trial (Study P301). If a threshold of protection is not available, efficacy will be inferred based on establishing noninferiority of adolescent (12 to < 18 years; this clinical study) to adult GM values of serum

antibody level and seroresponse rate obtained in Study P301 (GM value 12 to < 18 years / GM value 18-25 years).

Seroresponse due to vaccination at a subject level may be defined as a change from below the LLOQ to equal to or above 4×LLOQ, or a 4-fold rise if baseline is equal to or above LLOQ.

Among the two Pseudovirus tests, PsVNT50 and PsVNT80, PsVNT50 is considered the most appropriate measure of subject response because it falls in the middle of the dynamic range of the dilution response curve while PsVNT80 is close to the plateau and thus subject to restriction.

The GM and seroresponse rate comparisons between adolescents in P203 and young adults (18-25 years of age) in P301 will be compared for the bAb and nAb measures, with pseudovirus nAb PsVNT50 (ID50) considered as the primary assay test for the immunobridging.

3.2. Secondary Endpoints

The secondary objective will be evaluated by the following endpoints:

- The GM values of SARS-CoV-2 S2P-specific bAb on Day 1, Day 57 (1 month after dose 2), Day 209 (6 months after dose 2), and Day 394 (1 year after dose 2).
- The GM values of SARS-CoV-2-specific nAb on Day 1, Day 57 (1 month after dose 2), Day 209 (6 months after dose 2), and Day 394 (1 year after dose 2).
- The incidence of SARS-CoV-2 infection counted starting 14 days after the second dose of IP. SARS-CoV-2 infection will be defined in participants with negative SARS-CoV-2 at baseline:
    - bAb levels against SARS-CoV-2 nucleocapsid protein negative at Day 1 that becomes positive (as measured by Roche Elecsys) starting at Day 57 or later. OR
    - Positive RT-PCR counted starting 14 days after the second dose of IP.
- The incidence of asymptomatic SARS-CoV-2 infection measured by RT-PCR and/or bAb levels against SARS-CoV-2 nucleocapsid protein (by Roche Elecsys) counted starting 14 days after the 2nd dose of IP in participants with negative SARS-CoV-2 at baseline.

- The incidence of the first occurrence of COVID-19 starting 14 days after the second dose of IP, where COVID-19 is defined as symptomatic disease based on the following criteria:
    - The participant must have experienced at least TWO of the following systemic symptoms: Fever (≥ 38° C/≥ 100.4° F), chills, myalgia, headache, sore throat, new olfactory and taste disorder(s), OR
    - The participant must have experienced at least ONE of the following respiratory signs/symptoms: cough, shortness of breath or difficulty breathing, OR clinical or radiographical evidence of pneumonia; AND
    - The participant must have at least 1 NP swab, nasal swab, or saliva sample (or respiratory sample, if hospitalized) positive for SARS-CoV-2 by RT-PCR.
- The incidence of the first occurrence of the secondary COVID-19 case starting 14 days after the first dose of IP, and the secondary COVID-19 case starting 14 days after the second dose of IP.

  The secondary case definition (CDC case definition) of COVID-19 is defined by the following criteria:
    - One of the following systemic or respiratory symptoms: fever (temperature > 38ºC/≥ 100.4ºF), or chills, cough, shortness of breath or difficulty breathing, fatigue, muscle aches, or body aches, headache, new loss of taste or smell, sore throat, congestion or runny nose, nausea, or vomiting or diarrhea, AND
    - At least one positive RT-PCR test for SARS-CoV-2

3.3. Exploratory Endpoints

The exploratory endpoints are the following:

- Alignment of genetic sequence of viral isolates with that of the vaccine sequence.
- Relative amounts or profiles of S protein-specific bAb and specific nAb levels/titers in serum.
- Description of clinical severity and immune responses of participants who are identified as infected by SARS-CoV-2 (COVID-19).

- GM and GMFR of bAb levels against SARS-CoV-2 nucleocapsid protein (quantitative IgG) and % of participants with 2x, 3x and 4x rise of bAb relative to baseline

4. Study Design

This study is split into 3 parts and the overall design schematic can be seen in Figure 1.

Figure 1: Overall Design Schema

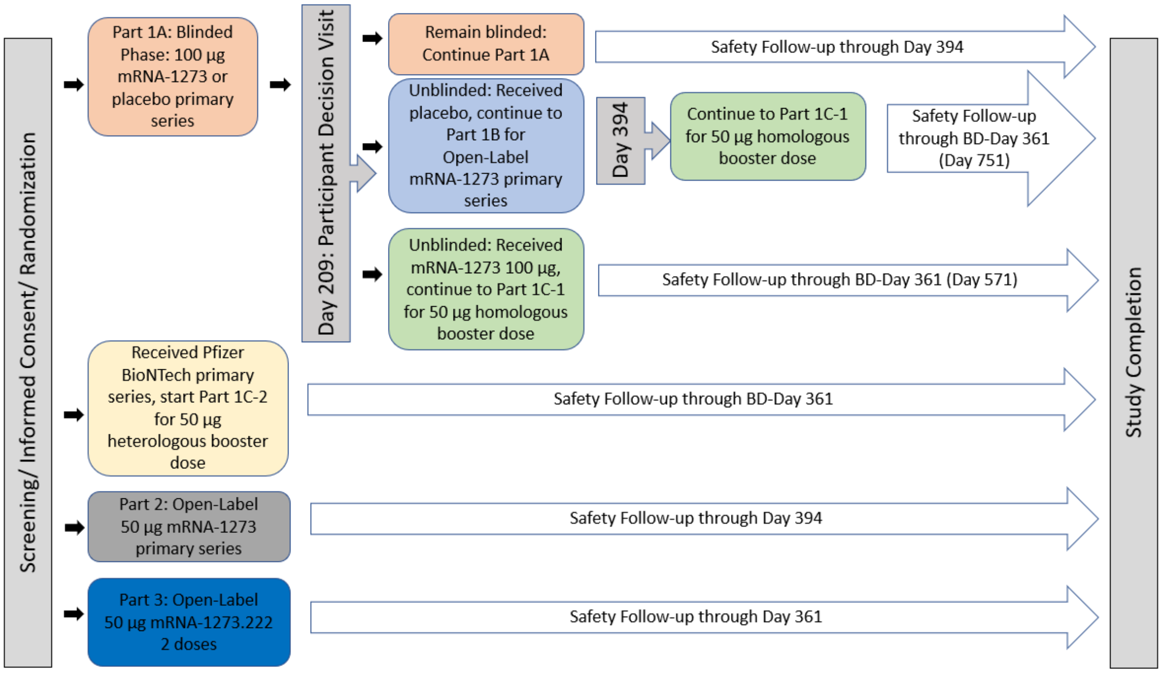

BD = booster dose.

4.1. Overall Study Design

4.1.1. Part 1 Study Design

This is a three-part, Phase 2/3 study: Part 1A, Part 1B, and Part 1C. Participants in Part 1A, the Blinded Phase of the study, are blinded to their treatment assignment.

Part 1B, the Open-label Observational Phase of this study, is designed to offer participants who received placebo in Part 1A of this study and who meet the Emergency Use Authorization (EUA) eligibility criteria an option to receive mRNA-1273 in an open-label fashion (Figure 3). Participants who received mRNA-1273 (100 µg) in Part 1A of this study will proceed to Part 1B after they are unblinded and will continue to follow the Part 1A Schedule of Assessments (SoA). Part 1C-1, the Homologous Booster Dose Phase, is

designed to offer participants in Part 1A and Part 1B, and who are at least 5 months from the last dose, the option to request a BD of 50 µg of mRNA-1273. Part 1C-2, the Heterologous Booster Phase, is designed to provide participants who completed non-Moderna primary COVID-19 vaccination series under EUA (ie, Pfizer) a 50 µg BD of mRNA-1273 at least 3 months from the last dose.

Participants who received mRNA-1273 in Part 1A will be in the study approximately 25 months if a BD is received in Part 1C-1, which includes 1 month for Screening (Day -28 to Day 1), up to 12 months for dosing (on Day 1, Day 29, and Day 209 or BD-D1), and 12 months for follow-up.

Participants who received placebo in Part 1A will be in the study for approximately 25 months total if a BD is received in Part 1C-1, which includes approximately 9 months in Part 1A and approximately 4 months of follow-up following their second dose of mRNA-1273 in Part 1B before entering Part 1C-1 or before BD is received; or approximately 12 months of follow-up.

Participants that decline to unblinding or decline to receive a BD will be in the study approximately 14 months total, which includes 1 month for Screening (Day -28 to Day 1), 1 month for dosing (on Day 1 and Day 29), and 12 months for follow-up.

Participants in Part 1C-2 will be in the study for approximately 12 months total, which includes 7 days for Screening (Day -7 to Day 1), 1 day of dosing (BD-Day 1), and 12 months of follow-up.

4.1.1.1. Part 1A, the Blinded Phase

The Blinded Phase of this study is a randomized, observer-blind, placebo-controlled study intended to infer the effectiveness of mRNA-1273 in an adolescent population aged 12 to < 18 years. The study includes 2 arms: (i) 100 µg of mRNA-1273, and (ii) placebo. Approximately, 3000 participants 12 to < 18 years of age will be randomly assigned in a 2:1 ratio to receive mRNA-1273 (n=2000) or placebo (n=1000).

The schematic of study arms and major study events for Part 1A is illustrated in Figure 2 and the Schedule of Assessments is provided in Appendix E.

The goal of the study is to seek an indication for use of mRNA-1273 (100 µg IM, given as 2 injections, 28 days apart) in the 12 to < 18 years age group. Each participant will receive one injection of mRNA-1273 or placebo on Day 1 and Day 29 and then be followed up for a total of 12 months following the second injection.

Figure 2 Study Design Schematic (Part 1A, Blinded Phase)

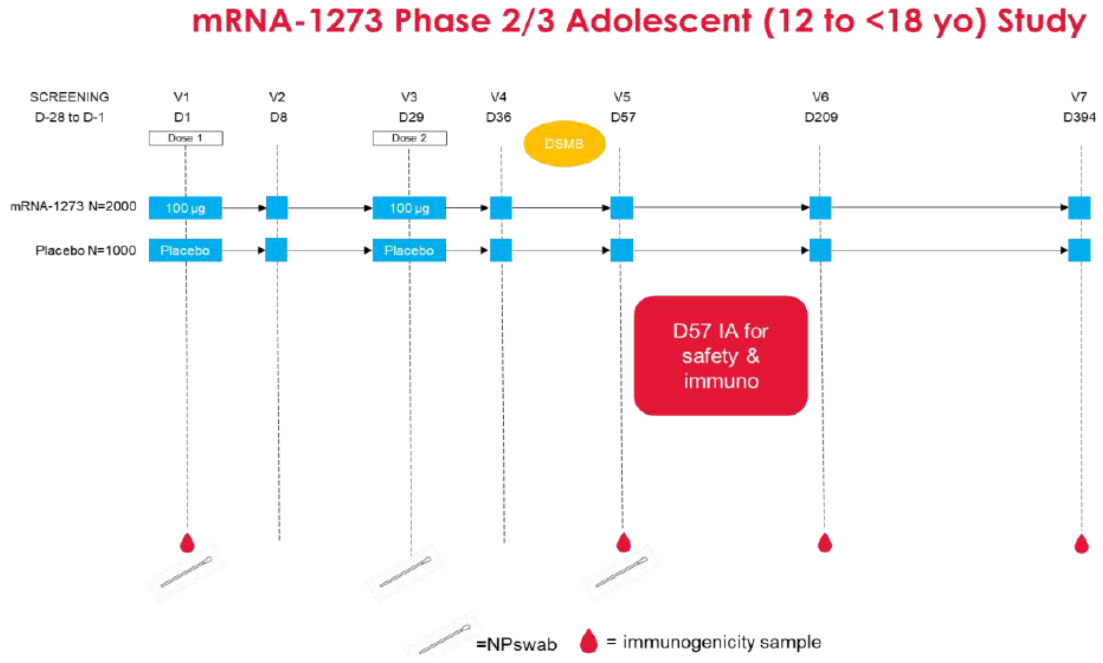

Abbreviation: D = day; DSMB = Data Safety Monitoring Board, IA = interim analysis, immuno = immunogenicity, NP = nasopharyngeal or nasal swab, V = visit, yo = years old.

4.1.1.2.Part 1B, the Open-label Observational Phase

Part 1B, the Open-label Observational Phase of the study, will be prompted by the authorization of a COVID-19 vaccine under an Emergency Use Authorization (EUA) for any persons under the age of 18 years. Participants will be transitioned to Part 1B of the study as their age group becomes EUA-eligible. This transition permits all ongoing study participants to eventually be informed of the availability and eligibility criteria of any COVID-19 vaccine made available under an EUA and the option to offer all ongoing study participants an opportunity to schedule a Participant Decision Visit to know their original treatment assignment (placebo vs. mRNA-1273 100 µg vaccine).

Part 1B provides the opportunity for study participants to be informed regarding the EUA, to be unblinded to their original assignment (mRNA-1273 or placebo), and for those who previously received placebo, to actively request to receive 2 doses of mRNA-1273 (100 µg) vaccine.

Figure 3 Study Schema (Part 1B, Open-label Observational Phase)

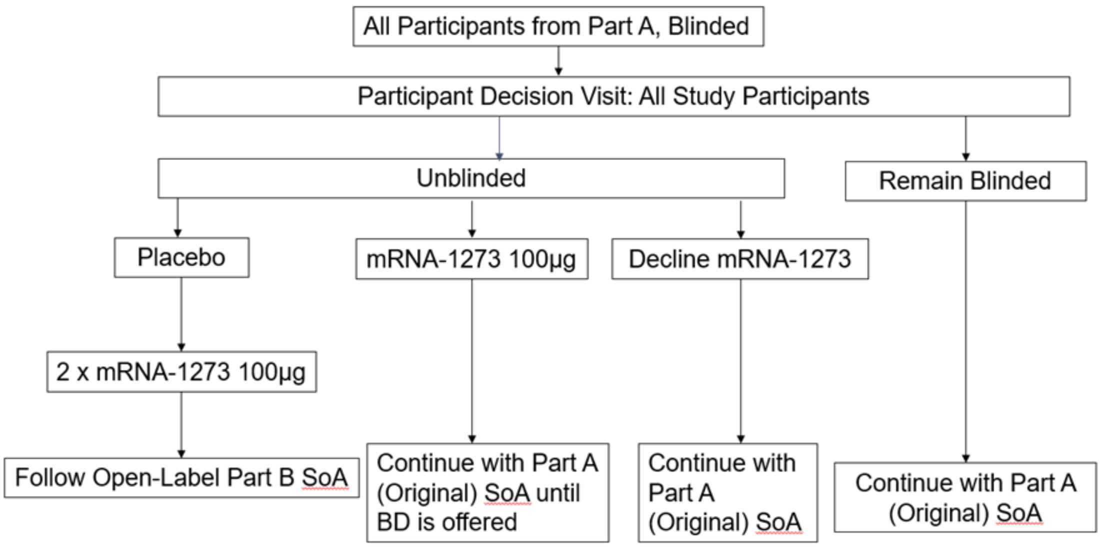

Abbreviation: BD = booster dose; SoA = Schedule of Assessments.

Participants that decline unblinding will remain in Part 1A and follow the Part 1A SoA (Appendix E).

After the Participant Decision Clinic Visit, all participants that consent to unblinding will follow the Part 1A SoA (Appendix E) or Part 1B SoA (Appendix F) as follows:

- Participants received placebo in Part 1A and consent to receiving 2 doses of mRNA-1273 in Part 1B: These participants will proceed to Part 1B and follow the Part 1B SoA in Appendix F until BD is offered.
- Participants received 2 doses of mRNA-1273 in Part 1A: Due to statistical considerations, these participants will be considered in the Open-label Observational Phase, but will continue to follow the Part 1A SoA in Appendix E until BD is offered.

4.1.1.3.Part 1C-1, the Homologous Booster Dose Phase

Part 1C-1 Homologous Booster Dose Phase is designed to offer participants in Part 1A and Part 1B, who are at least 5 months from the last dose, the option to request a BD (50 µg) of mRNA-1273.

Figure 4 Study Schema (Part 1C-1, Open-label Homologous Booster Dose Observational Phase)

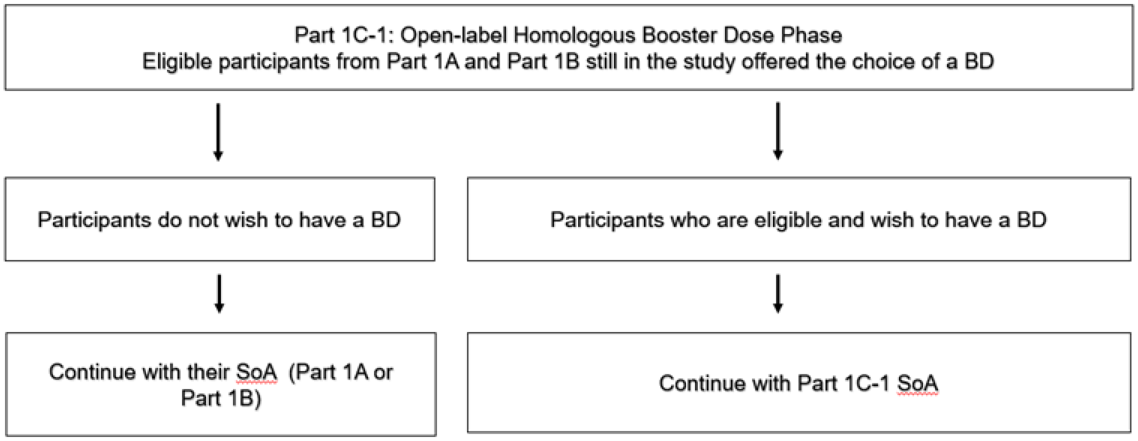

Abbreviations: BD = booster dose; D = day; SoA = Schedule of Assessments.

At the BD-D1 visit, participants who request a BD and are eligible will have the study site visits and complete scheduled activities (subject to investigational vaccine availability) according to the Part 1C-1 Supplemental SoA in Appendix G.

Participants that received mRNA-1273 in Part 1A and decline BD will continue with in Part 1A SoA (Appendix E).

Participants that crossed over from placebo to mRNA-1273 and decline BD will continue with Part 1B SoA (Appendix F).

As this Supplemental SoA is intended to occur in addition to the original SoAs being followed by all participants in Part 1A or Part 1B, there is a possibility for study visits to overlap (ie, Part 1C-1 BD-Day 1 and study visit in Part 1A or Part 1B that coincides with Part 1C-1 BD-Day 1). If visits overlap according to respective visit windows, a single visit may be done with the combined study procedures completed once. Any participant who consented to receive a BD will follow Part 1C-1 SoA. In case a study visit in Part 1A or Part 1B coincides with Part 1C-1 BD-Day1, subject should follow Part 1C-1 SoA.

4.1.1.4.Part 1C-2, Heterologous Booster Phase

Part 1C-2 – Heterologous Booster Phase is designed to provide a BD (50 µg) of mRNA-1273 to eligible participants who completed primary COVID-19 vaccination series with a

non-Moderna vaccine under EUA (ie, Pfizer, Table 1) and are at least 3 months from the last dose. The Supplemental SoA for Part 1C-2 is located in Appendix H.

Table 1: Non-Moderna COVID-19 Vaccine Available Under EUA

COVID-19 Vaccine – Primary Series	Primary Series Number of Doses
Pfizer-BioNTech	2 doses

4.1.2. Part 2 Study Design

Part 2 is an open-label design. The study will evaluate the safety, reactogenicity, and effectiveness of a 50 µg primary series of mRNA-1273 SARS-CoV-2 vaccine in healthy adolescents 12 to < 18 years of age.

The schematic of study arms and major study events for Part 2 is illustrated in Figure 5 and the SoA for Part 2 is located in Appendix I.

Participants who received mRNA-1273 in Part 2 will be in the study approximately 12 months, which includes 1 week for Screening (Day -7 to Day 1), 1 months for dosing (on Day 1, Day 29), and 12 months of follow-up after Dose 2.

Figure 1: Study Schema (Part 2)

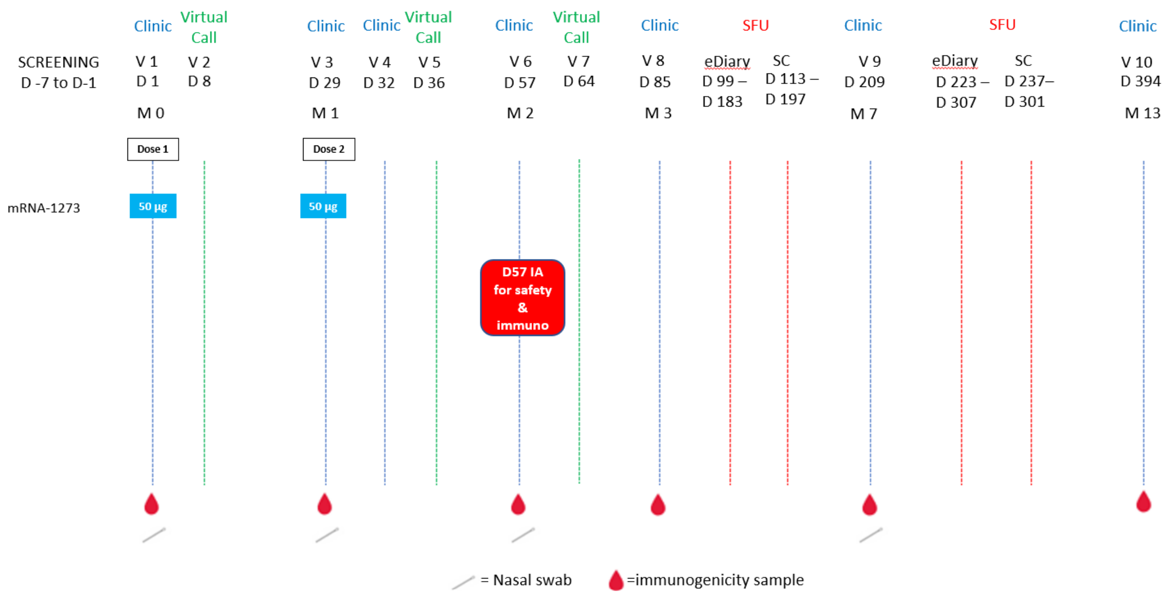

Abbreviation: D = day; IA = interim analysis; immuno = immunogenicity; SC = safety call; SFU = safety follow-up; V = visit.

4.1.3. Part 3 Study Design

Part 3 is an open-label, single-arm design. The study will evaluate the safety, reactogenicity, and effectiveness of a 2-dose 50 µg primary series of mRNA-1273.222 SARS-CoV-2 vaccine, administered 6 months apart, in approximately at least 300 healthy adolescents 12 to <18 years of age.

The schematic of study arms and major study events for Part 3 is illustrated in Figure 6 and the SoA for Part 3 is described in Appendix J.

Participants who receive mRNA-1273.222 in Part 3 will be in the study for approximately 12 months including 1 week for Screening (Day -7 to Day -1), 6 months for dosing (Day 1 to Day 181), and 6 months of follow-up after Dose 2.

Figure 6: Study Schema (Part 3)

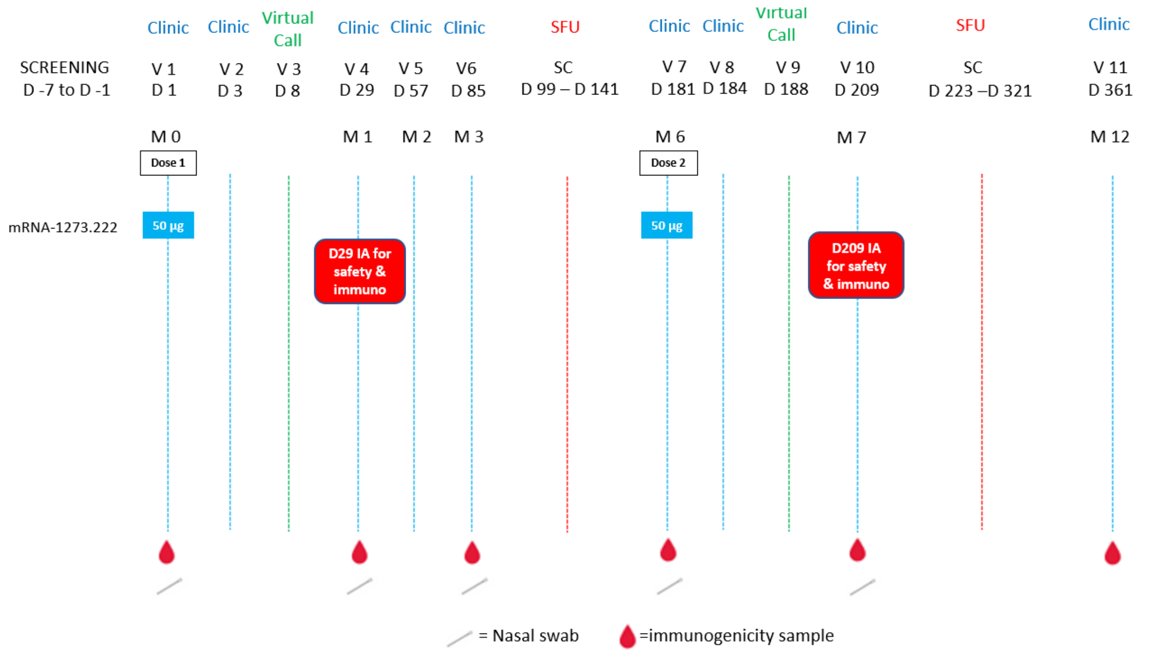

Abbreviation: D = day; IA = interim analysis; immuno = immunogenicity; M = month; SC = safety call; SFU = safety follow-up; V = visit.

4.2. Statistical Hypothesis

- If an accepted serum Ab threshold of protection against COVID-19 is established for the primary immunogenicity objective, the null hypothesis is that the percentage of participants on mRNA-1273 with serum Ab equal to or above the established threshold at Day 57 is ≤ 70% (ie, H_0: percentage of participants on mRNA-1273 ≤ 70% with serum Ab at Day 57 equal to or above the established threshold).

  The study would be considered to meet the immunogenicity objective if the 95% confidence interval (CI) of percentage of participants on mRNA-1273 rules out 70% (lower bound of the 95% CI > 70%).

- If an accepted serum Ab threshold of protection against COVID-19 is not available for the primary immunogenicity objective, the immunogenicity analysis of primary vaccine response will be performed using the noninferiority tests of the 2 null hypotheses based on the 2 coprimary endpoints, respectively.

  - Coprimary endpoint 1: Ab geometric mean (GM) value at Day 57

The null hypothesis:

H^1_0: immunogenicity response to mRNA-1273 as measured by Ab GM value at Day 57 is inferior in adolescents (12 to < 18 years of age) receiving mRNA-1273 compared with that in young adults (18 to 25 years of age) receiving mRNA-1273 using Study P301 data.

The noninferiority in Ab GM in adolescents compared with that in young adults (18 to 25 years of age) is demonstrated by meeting both success criteria:

- The lower bound of the 95% CI of the geometric mean ratio (GMR) rules out 0.667 (lower bound > 0.667) using a noninferiority margin of 1.5.
- The GMR point estimate > 0.8 (minimum threshold).

The GMR is the ratio of the GM value of adolescents on mRNA-1273 in this study, Study P203, at Day 57 compared with the GM value of young adults (18-25 years of age) on mRNA-1273 in Study P301.

- Coprimary endpoint 2: Ab seroresponse rate at Day 57

  The null hypothesis:

  H^2_0: immunogenicity response to mRNA-1273 as measured by seroresponse rate at Day 57 is inferior in adolescents (12 to < 18 years of age) receiving mRNA-1273 compared with that in adults (18 to 25 years of age)using mRNA-1273 Study P301 data

  The noninferiority in seroresponse rate in adolescents compared with that in adults (18 to 25 years) is demonstrated by meeting both success criteria:

  - The lower bound of the 95% CI of the seroresponse rate difference rules out -10% (i.e. lower bound > -10%) using the noninferiority margin of 10%.
  - The seroresponse rate difference point estimate > -5% (minimum threshold)

  The seroresponse rate difference is defined as the rate in adolescents receiving mRNA-1273 minus the rate in young adults (18 to 25 years of age) receiving mRNA-1273 from Study P301.

The study would be considered as meeting the primary immunogenicity objective if noninferiority is demonstrated based on both coprimary endpoints.

4.3. Sample Size and Power

The sample size of this study is driven by safety. Approximately 3,000 participants will be randomly assigned in a 2:1 ratio to receive mRNA-1273 and placebo. With 2,000 participants exposed to mRNA-1273, the study has at least 90% probability to observe at least 1 participant with an AE at a true 0.25% AE rate.

Serum samples from all participants will be collected and banked, a subset of participants will be selected, and their samples will be processed for immunogenicity testing (the Immunogenicity Subset).

Approximately 362 participants who receive mRNA-1273 will be selected for the Immunogenicity Subset, with a target of 289 participants in the PP Immunogenicity Subset (adjusting for approximately 20% of participants who may be excluded from the PP Immunogenicity Subset, as they may not have immunogenicity results due to any reason). The sample size of the Immunogenicity Subset may be updated with data from other mRNA-1273 studies or external data especially regarding a threshold of protection.

For the primary immunogenicity objective, with approximately 289 participants in the PP Immunogenicity Subset, the study will have > 90% power to rule out 70% with a 2-sided 95% CI for the percentage of mRNA-1273 participants exceeding the acceptable threshold if the true rate of participants exceeding the acceptable threshold is 80%.

If an acceptable Ab threshold of protection against COVID-19 is not available at the time of analysis, for the primary immunogenicity objective, noninferiority tests of two null hypotheses based on two coprimary endpoints, respectively, will be performed. The sample size calculation for each of the two noninferiority tests was performed, and the larger sample size was chosen for the study.

- With approximately 289 participants in the PP Immunogenicity Subset in Study P203 and 289 participants in the PP Immunogenicity Subset in young adults (18-25 years of age) from Study P301, there will be 90% power to demonstrate noninferiority of the immune response as measured by Ab GM in adolescents in Study P203 at a 2-sided alpha of 0.05, compared with that in young adults (18-25 years of age) from Study P301 receiving mRNA-1273, assuming an underlying GMR value of 1, a noninferiority margin of 1.5, and a point estimate minimum threshold of 0.8. The standard deviation (SD) of the log-transformed levels is assumed to be 1.5.

- With approximately 289 participants in the PP Immunogenicity Subset in Study P203 and 289 participants in the PP Immunogenicity Subset in young adults (18-25 years of age) from Study P301, there will be at least 90% power to demonstrate noninferiority of the immune response as measured by seroresponse rate in adolescents in Study P203 at a 2 sided alpha of 0.05, compared with that in young adults (18-25 years of age) from Study P301 receiving mRNA 1273, assuming a true seroresponse rate of 85% in young adults (18-25 years of age) from Study P301, and a true seroresponse rate of 85% in adolescents in P203 (i.e., true rate difference is 0 compared to young adults from Study P301), a noninferiority margin of 10%, and a point estimate minimum threshold of -5% in seroresponse rate difference.

4.4. Randomization

Approximately 3,000 participants will be randomly assigned in a 2:1 ratio to receive mRNA-1273 and placebo for Part 1A. The randomization will be in a blinded manner using a centralized Interactive Response Technology (IRT) at the Day 1 visit, in accordance with pre-generated randomization schedules. There will be no strata for randomization in this study.

4.5. Blinding and Unblinding

Part 1A of this study is observer-blind. The investigator, study staff, study participants, site monitors, and Sponsor personnel (or its designees) will be blinded to the investigational product administered until study end or initiation of Part 1B, with certain exceptions, please refer to Section 8.1 of the protocol for details. Planned analyses in this study include two interim analyses (refer to Section 6.8 for details) and a final analysis at the end of study. At the time of interim analysis, only pre-identified Sponsor and unblinded Contract Research Organization (CRO) team members as specified in the study Data Blinding Plan will be unblinded to review treatment level results and individual listings, please also refer to Section 6.8. Study sites will remain blinded to individual treatment assignments until the end of the study or initiation of Part 1B.

5. Analysis Populations

The following analysis sets are defined: Randomization Set, Full Analysis Set (FAS), Immunogenicity Subset, Per-protocol (PP) Immunogenicity Subset, Modified Intent-to-

Treat (mITT) Set, Modified Intent-to-Treat-1 (mITT1) Set, Per-protocol (PP) Set for Efficacy, Solicited Safety Set, and Safety Set.

5.1. Randomization Set

The Randomization Set consists of all participants who are randomized in the study, regardless of the participant's treatment status in the study. Participants will be analyzed according to the treatment group to which they were randomized.

5.2. Full Analysis Set

The Full Analysis Set (FAS) consists of all randomized participants who received at least one dose of IP. Participants will be analyzed according to the treatment group to which they were randomized.

5.3. Immunogenicity Subset

A subset of participants in the FAS will be selected for immunogenicity testing. Immunogenicity Subset consists of

a) a subset of participants in the FAS, and

b) have baseline (Day 1) SARS-CoV-2 status available, and

c) have baseline and at least one post-injection antibody assessment for the analysis endpoint.

Participants will be analyzed according to the treatment group to which they were randomized.

5.4. Per-protocol (PP) Immunogenicity Subset

Per-Protocol (PP) Immunogenicity Subset consists of all participants in Immunogenicity Subset who meet all the following criteria:

a) Received planned doses of study vaccination per schedule

b) Complied with the timing of second dose of injection

c) Had a negative RT-PCR test for SARS-CoV-2 and a negative serology test based on bAb specific to SARS-CoV-2 nucleocapsid (as measured by *Roche Elecsys* Anti-SARS-CoV-2 assay) at baseline

d) Had baseline (Day 1) and Day 57 Ab assessment for the analysis endpoint

e) Had no major protocol deviations that impact key or critical data

The PP Immunogenicity Subset will serve as the primary population for the analysis of immunogenicity data in this study. Participants will be analyzed according to the treatment group to which they were randomized.

5.5. Modified Intent-to-Treat (mITT) Set

The Modified Intent-to-Treat (mITT) Set consists of all participants in the FAS who had no serologic or virologic evidence of prior SARS-CoV-2 infection (both negative RT-PCR test for SARS-CoV-2 and negative serology test based on bAb specific to SARS-CoV-2 nucleocapsid) before the first dose of IP, i.e., all FAS participants excluding those with positive or missing RT-PCR test or serology test at baseline.

Participants will be analyzed according to the treatment group to which they were randomized.

5.6. Modified Intent-to-Treat-1 (mITT1) Set

The mITT1 Set consists of all participants in the mITT Set excluding those who received the wrong treatment (i.e., at least one dose received in Part 1A is not as randomized).

Participants will be analyzed according to the treatment group to which they were randomized.

5.7. Per-protocol (PP) Set for Efficacy

The Per-protocol (PP) Set for Efficacy consists of all participants in the FAS who meet all the following criteria:

a) Received planned doses of study vaccination

b) Complied with the timing of second dose of injection

c) Had a negative RT-PCR test for SARS-CoV-2 and a negative serology test based on bAb specific to SARS-CoV-2 nucleocapsid (as measured by *Roche Elecsys* Anti-SARS-CoV-2 assay) at baseline

d) Had no major protocol deviations that impact key or critical efficacy data.

Participants will be analyzed according to the treatment group to which they were randomized.

5.8. Solicited Safety Set

The Solicited Safety Set consists of all participants who are randomized and received any study injection, and contribute any solicited AR data, i.e., have at least one post-baseline solicited safety assessment. The Solicited Safety Set will be used for the analyses of solicited ARs and participants will be included in the vaccination group corresponding to the study injection they actually received. In addition, the following Solicited Safety Set is defined for each injection separately.

The First (Second) Injection Solicited Safety Set consists of all subjects in the Solicited Safety Set who have received the first (second) study injection and have contributed any solicited AR data from the time of first (second) study injection through the following 6 days.

Participants will be analyzed according to the vaccination group a participant received, rather than the vaccination group to which the subject was randomized. A participant who was randomized to placebo but received any dose of mRNA-1273 at any injection will be included in the mRNA-1273 group in the Solicited Safety Set.

5.9. Safety Set

The Safety Set consists of all randomized participants who received any study injection. The Safety Set will be used for analysis of safety except for the solicited ARs. Participants will be included in the vaccination group corresponding to the vaccination they actually received. For a participant who was randomized to placebo but received any dose of mRNA-1273 at any injection, the participant will be included in the mRNA-1273 group in the Safety Set.

6. Statistical Analysis

6.1. General Considerations

The Schedule of Assessments is provided in Appendix E for Part 1A Blinded Phase, and Appendix F for Part 1B Open-label Observational Phase.

Continuous variables will be summarized using the following descriptive summary statistics: the number of subjects (n), mean, standard deviation (SD), median, minimum (min), and maximum (max).

Categorical variables will be summarized using counts and percentages.

Baseline value, unless specified otherwise, is defined as the most recent non-missing measurement (scheduled or unscheduled) collected before the first dose of IP. For immunogenicity tests, the baseline is defined as the most recent non-missing measurement (scheduled or unscheduled) collected before or on the date of first dose of IP.

For the summary statistics of all numerical variables unless otherwise specified, the display precision will follow programming standards. Please see Appendix A for variable display standards.

When count data are presented, the percentage will be suppressed when the count is zero in order to draw attention to the non-zero counts. A row denoted "Missing" will be included in count tabulations where specified on the shells to account for dropouts and missing values. The denominator for all percentages will be the number of subjects in that vaccination group within the analysis set of interest, unless otherwise specified.

Baseline SARS-CoV-2 status is determined by using virologic and serologic evidence of SARS-CoV-2 infection on or before Day 1.

Positive SARS-CoV-2 status at Baseline is defined as a positive RT-PCR test for SARS-CoV-2, and/or a positive serology test based on bAb specific to SARS-CoV-2 nucleocapsid (as measured by *Roche Elecsys* Anti-SARS-CoV-2 assay) on or before Day 1.

Negative status at Baseline is defined as a negative RT-PCR test for SARS-CoV-2 and a negative serology test based on bAb specific to SARS-CoV-2 nucleocapsid (as measured by *Roche Elecsys* Anti-SARS-CoV-2 assay) on or before Day 1.

Study day relative to the first injection will be calculated as below:

a) study day prior to the first injection will be calculated as: date of assessment/event – date of the first injection;

b) study day on or after the date of the first injection will be calculated as: date of assessment/event – date of the first injection + 1;

Study day relative to the most recent injection will be calculated as below:

a) study day prior to the first injection will be calculated as: date of assessment/event – date of the first injection;

b) study day on or after the date of the first injection but before the second injection (if applicable) will be calculated as: date of assessment/event – date of the first injection + 1;

c) study day on or after the date of the second injection but before the first injection in Part 1B (if applicable) for subjects who received placebo in Part 1A, or before the injection in Part 1C-1 (if applicable) for subjects who received mRNA-1273 in Part 1A will be calculated as: date of assessment/event – date of the second injection + 1; if study day is on the same day as the second injection, date and time will be compared with the second injection date and time.

For calculation regarding antibody levels/titers, antibody values reported as below LLOQ will be replaced by 0.5 × LLOQ. Values that are greater than the upper limit of quantification (ULOQ) and without actual values reported (eg, '>xxx') will be converted to the ULOQ. Values that are greater than the ULOQ and with actual values reported will not be imputed. Missing results will not be imputed (i.e. actual values will be used). Missing results will not be imputed.

The following **analysis periods or stages for safety analyses in the blinded phase** will be used in this study:

- Up to 28 days after any vaccination: this stage starts at the day of each vaccination and continue through the earliest date of (the day of each vaccination and 27 subsequent days, next vaccination [if applicable]). This analysis period will be used as the primary analysis period for safety analyses including unsolicited AE, except for solicited AR, unless specified otherwise.

- Follow-up analysis period:

  For unsolicited AE or assessments that will be collected throughout the study, this analysis period starts from 28 days after the last injection date (i.e. the day of last injection + 28 days, regardless of number of injections received) and continues until the earliest date of (unblinding, non-study COVID-19 vaccine use, first injection in Part 1B, injection in Part 1C-1, study completion, discontinuation from the study, or death).

For assessments that will be collected at study visits (e.g. vital sign), if a subject receives two injections, this stage starts from the day after Day 57 visit and continues until the earliest date of (unblinding, non-study COVID-19 vaccine use, first injection in Part 1B, injection in Part 1C-1, study completion, discontinuation from the study, or death); if a subject receives first injection only, this stage starts from the day after Day 29 visit and continues until the earliest date of (unblinding, non-study COVID-19 vaccine use, first injection in Part 1B, injection in Part 1C-1, study completion, discontinuation from the study, or death).

- Overall period: this analysis period starts at the first injection on Day 1 and continues through the earliest date of (unblinding, non-study COVID-19 vaccine use, first injection in Part 1B, injection in Part 1C-1, study completion, discontinuation from the study, or death).

Unscheduled visits: Unscheduled visit measurements will be included in analysis as follows:

- In scheduled visit windows per specified visit windowing rules.
- In the derivation of baseline/last on-treatment measurements.
- In the derivation of maximum/minimum on-treatment values and maximum/minimum change from baseline values for safety analyses.
- In individual subject data listings as appropriate.

Visit windowing rules: The analysis visit windows for protocol-defined visits are provided in Appendix B.

Incomplete/missing data:

- Imputation rules for missing prior/concomitant medications, non-study vaccinations and procedures are provided in Appendix C.
- Imputation rules for missing AE dates are provided in Appendix D.
- For laboratory assessments, if majority of results are indefinite, imputation of these values will be considered. If the laboratory results are reported as below the LLOQ (e.g., <0.1), the numeric values will be imputed by 0.5 × LLOQ in the summary. If

the laboratory results are reported as greater than the ULOQ (e.g., >3000), the numeric values will be imputed by ULOQ in the summary.

- Other incomplete/missing data will not be imputed, unless specified otherwise.

Treatment groups:

The following vaccination groups will be used for summary purposes:

- Part 1A, Blinded Phase:
    - mRNA-1273
    - Placebo

If a subject received any dose of mRNA-1273 at any injection, regardless of the treatment group the subject was randomized to, the subject will be included to mRNA-1273 100 µg group as the actual treatment group received for safety analyses.

Analysis Periods

The following analysis periods and treatment groups will be used for the Blinded Phase Analysis.

- Blinded Phase:

Cohort	Category	Start Date of Blinded Phase	End Date of Blinded Phase
mRNA-1273	Safety	Date of First Dose	Earlist date of unblinding (non-inclusive), non-study COVID-19 vaccination, first injection in Part 1B, injection in Part 1C-1, study discontinuation, study completion, death, or data cutoff
Placebo			
mRNA-1273	Efficacy/ Immunogenicity	Randomization Date	Earliest date of unblinding (inclusive), non-study COVID-19 vaccination date, first injection in Part 1B, injection in Part 1C-1, study discontinuation, study completion, death, or data cutoff
Placebo			

Subgroup Analysis

Safety, efficacy and immunogenicity endpoints may be analyzed in select subgroups specified below as applicable:

- Baseline SARS-CoV-2 Status (Positive, Negative)
- Age (≥12 and <16 Years, ≥16 and <18 Years)
- Sex (Female, Male)
- Race
- Ethnicity
- At Risk for Severe COVID-19 (Yes, No)
    - Subjects at risk for severe COVID-19, as identified by the CDC (CDC 2022) and literature (Kompaniyets L et al 2021), include those immunocompromised, those with obesity (defined by WHO BMI 95th percentile), chronic lung disease (including asthma), neurologic disorders and other chronic conditions

All analyses and data summaries/displays will be provided by vaccination group using appropriate analysis population unless otherwise specified.

All analyses will be conducted using SAS Version 9.4 or higher.

6.2. Background Characteristics

6.2.1. Subject Disposition

The number and percentage of subjects in the following categories will be summarized by vaccination group as defined in Section 6.1 based on Randomization Set:

- Randomization Set
- Full Analysis Set
- Immunogenicity Subset
- Per-protocol (PP) Immunogenicity Subset
- mITT Set

- mITT1 Set
- Per-protocol (PP) Set for Efficacy
- Solicited Safety Set
- Safety Set

The percentage will be based on subjects in that vaccination group within the Randomization Set (as randomized), except the Solicited Safety Set and Safety Set for which the percentages will be based on the vaccination group in the Safety Set (as treated).

The number of subjects in the following categories will be summarized based on subjects screened:

- Number of subjects screened
- Number and percentage of screen failure subjects and the reason for screen failure

The percentage of subjects who screen failed will be based on the number of subjects screened. The reason for screen failure will be based on the number of subjects who screen failed.

The number and percentage of subjects in each of the following disposition categories will be summarized by vaccination group based on the Randomization Set:

- Randomized by site
- Received each dose of IP
- Prematurely discontinued before receiving the second dose of IP and the reason for discontinuation
- Completed study
- Prematurely discontinued the study and the reason for discontinuation

A subject disposition listing will be provided, including informed consent, subjects who completed the study injection schedule, subjects who completed study, subjects who discontinued from study vaccine or who discontinued from participation in the study, with reasons for discontinuation. A separate listing will be provided for screen failure subjects with reasons for screen failure.

A subject who completed 12 months of follow up after the last injection received is considered to have completed the study.

6.2.2. Demographics

Descriptive statistics will be calculated for the following continuous demographic and baseline characteristics: age (years), weight (kg, z-score), height (cm, z-score), and body mass index (BMI) (kg/m^2, z-score). Number and percentage of subjects will be provided for categorical variables such as gender, race, ethnicity. The summaries will be presented by vaccination group as defined in Section 6.1 based on the FAS, Safety Set, Immunogenicity Subset, mITT Set, mITT1 Set, Per-protocol (PP) Immunogenicity Subset and Per-protocol (PP) Efficacy Set. If the Safety Set differs from the Randomization Set (e.g., subjects randomized but not received any study injection; subjects received study vaccination other than the vaccination group they were randomized to), the analysis will also be conducted using the Randomization Set.

For screened failure subjects, age (years), as well as gender, race, ethnicity will be presented in a listing.

In addition, subjects with any inclusion and exclusion criteria violation will also be provided in a listing.

6.2.3. Medical History

Medical history data will be coded by system organ class (SOC) and preferred term (PT) using the Medical Dictionary for Regulatory Activities (MedDRA).

The number and percentage of participants with any medical history and selected medical history conditions will be summarized by SOC and PT based on the Safety Set. A participant will be counted only once for multiple events within each SOC and PT. SOC will be displayed in internationally agreed order. PT will be displayed in descending order of frequency of mRNA-1273 and then alphabetically within SOC.

Medical history data will be presented in a listing.

6.2.4. Prior and Concomitant Medications

Prior and concomitant medications and non-study vaccination will be coded using the World Health Organization (WHO) drug dictionary (WHODD). The summary of concomitant medications will be based on the Safety Set. Categorization of prior, concomitant, and post medications is summarized in Appendix C Table 7.

The number and percentage of subjects using concomitant medications and non-study vaccination during the 7-day follow-up period (i.e., on the day of injection and the 6

subsequent days) and during the 28-day follow-up period after each injection (i.e., on the day of injection and the 27 subsequent days) will be summarized by vaccination groups as defined in Section 6.1 as follows:

- Any concomitant medications and non-study vaccination within 7 Days Post Injection
- Any concomitant medications and non-study vaccination within 28 Days Post Injection
- Seasonal influenza vaccine within 28 Days Post Injection
- Antipyretic or analgesic medication within 28 Days Post Injection

A summary table of concomitant medications and non-study vaccination that continued or newly received at or after the first injection through 28 days after the last injection will be provided by PT in descending frequency in the mRNA-1273 group.

Medications taken to prevent pain or fever will be collected on eDiary and summaries will be provided based on the Solicited Safety Set by vaccination group as defined in Section 6.1 for each injection (first or second) and any injection, including within 7 days after injection, beyond 7 days after injection and after injection.

Prior, concomitant and post medications and non-study vaccination will be presented in a listing.

Concomitant Procedures will be presented in a listing.

6.2.5. Study Exposure

Study IP administration data will be presented in a listing.

Study duration will be summarized since randomization, since the first injection, and since the second injection.

6.2.6. Major Protocol Deviations

Major protocol deviations are a subset of protocol deviations that may significantly impact the completeness, accuracy, or reliability of the study data or that may significantly affect a subject’s rights, safety, or well-being. Major protocol deviations rules are developed based on the protocol and ongoing data and will be finalized before database lock.

The number and percentage of the subjects with each major protocol deviation type will be provided by vaccination group as defined in Section 6.1 based on the Randomization Set.

Major protocol deviations will be presented in a listing.

6.2.7. COVID-19 Impact

A listing will be provided for COVID-19 impact.

6.3. Safety Analysis

Safety and reactogenicity will be assessed by clinical review of all relevant parameters including solicited ARs (local and systemic), unsolicited AEs, SAEs, MAAEs, AESI, AEs leading to withdrawal from study vaccine and/or study participation, vital signs, and physical examination findings. Unsolicited AEs will be coded by SOC and PT according to the MedDRA. The Toxicity Grading Scale for Healthy Adult and Adolescent Volunteers Enrolled in Preventative Vaccine Clinical Trials (DHHS 2007) is used in this study for solicited ARs as presented in Table 6 from protocol.

All safety analyses will be based on the Safety Set, except summaries of solicited ARs which will be based on the Solicited Safety Set. All safety analyses will be provided by vaccination group unless otherwise specified.

6.3.1. Adverse Events

A treatment-emergent AE (TEAE) is defined as any event occurring during the study not before exposure to study vaccine or any event already present that worsens after exposure to study vaccine. [Note: worsening of a pre-existing condition after vaccination will be reported as a new AE.]

Adverse events will also be evaluated by the investigator for the coexistence of MAAE which is defined as an AE that leads to an unscheduled visit to a healthcare practitioner.

Unsolicited AEs will be coded by PT and SOC using MedDRA and summarized by vaccination group, and stage (up to 28 days after any vaccination, follow-up analysis period and overall stage; see Section 6.1 for definitions of vaccination group and stage).

All summary tables (except for the overall summary of AEs) for unsolicited AEs will be presented by SOC and PT for TEAEs with numbers of subjects included. SOC will be displayed in internationally agreed order. PT will be displayed in descending order of frequency of mRNA-1273 and then alphabetically within SOC. When summarizing the

number and percentage of subjects with an event, subjects with multiple occurrences of the same AE or a continuing AE will be counted once. Subjects will be presented according to the highest severity (the strongest relationship) in the summaries by severity (of related AEs), if subjects reported multiple events under the same SOC and/or PT.

Percentages will be based upon the number of subjects in the Safety Set within each vaccination group.

6.3.1.1. Incidence of Adverse Events

An overall summary of unsolicited TEAEs including the number and percentage of subjects who experience the following will be presented:

- Any unsolicited TEAEs
- Any serious TEAEs
- Any fatal TEAEs
- Any unsolicited medically-attended TEAEs
- Any unsolicited TEAEs leading to discontinuation from study vaccine
- Any unsolicited TEAEs leading to discontinuation from participation in the study
- Any unsolicited severe TEAEs
- Any AESI of MIS-C
- Any AESI other than MIS-C

The table will also include number and percentage of subjects with unsolicited TEAEs that are treatment-related in each of the above categories.

In addition, separate listings containing individual subject adverse event data for unsolicited AEs, unsolicited TEAEs leading to discontinuation from study vaccine, unsolicited TEAEs leading to discontinuation from participation in the study, serious AEs, unsolicited medically-attended AEs, AESI of MIS-C, and AESI other than MIS-C (including myocarditis and/or pericarditis) will be provided separately.

6.3.1.2.TEAEs by System Organ Class and Preferred Term

The following summary tables of TEAEs will be provided by SOC and PT using frequency counts and percentages (i.e., number and percentage of subjects with an event):

- All unsolicited TEAEs
- All unsolicited TEAEs that are treatment-related
- All serious TEAEs
- All serious TEAEs that are treatment-related
- All unsolicited TEAEs leading to discontinuation from study vaccine
- All unsolicited TEAEs leading to discontinuation from participation in the study
- All unsolicited Severe TEAEs
- All unsolicited Severe TEAEs that are treatment-related
- All unsolicited medically-attended TEAEs
- All unsolicited medically-attended TEAEs that are treatment-related
- All AESI of MIS-C
- All AESI other than MIS-C

6.3.1.3.TEAEs by Preferred Term

A summary table of all unsolicited TEAEs will be provided. PTs will be sorted in a descending order according to the frequency in mRNA-1273 group.

6.3.1.4.TEAEs by System Organ Class, Preferred Term and Severity

The following summary tables of TEAEs will be provided by SOC, PT, and maximum severity (mild < moderate < severe) using frequency counts and percentages:

- All unsolicited TEAEs
- All unsolicited TEAEs that are treatment-related

6.3.2. Solicited Adverse Reactions

An AR is any AE for which there is a reasonable possibility that the test product caused the AE. The term “Solicited Adverse Reactions” refers to selected signs and symptoms occurring after injection administration during a specified post-injection follow-up period (day of injection and 6 subsequent days). The solicited ARs are recorded by the subject in eDiary. The occurrence and intensity of selected signs and symptoms is actively solicited

from the participant during a specified post-injection follow-up period (day of injection and 6 subsequent days), using a pre-defined checklist (i.e., solicited ARs).

The following local ARs will be solicited by the eDiary: pain at injection site, erythema (redness) at injection site, swelling (hardness) at injection site, and localized axillary swelling or tenderness ipsilateral to the injection arm.

The following systemic ARs will be solicited by the eDiary: headache, fatigue, myalgia (muscle aches all over the body), arthralgia (aching in several joints), nausea/vomiting, rash, fever, and chills.

The solicited ARs will be graded based on the grading scales presented in Table 6 in the protocol, modified from the Toxicity Grading Scale for Healthy Adult and Adolescent Volunteers Enrolled in Preventative Vaccine Clinical Trials (DHHS 2007). Investigator will assess Grade 4 events (with exception of fever).

If a solicited local or systemic AR continues beyond 7 days post injection, the participant will be prompted to capture the solicited local or systemic AR in the eDiary until resolution.

All solicited ARs (local and systemic) will be considered causally related to injection.

Analyses of solicited ARs will be provided by treatment group for each injection (first or second) based on the associated subset of Solicited Safety Set, i.e. First (Second) Injection Solicited Safety Set; and for any injection based on the Solicited Safety Set, unless otherwise specified.

The number and percentage of subjects who reported each individual solicited local AR (has a severity grade of Grade 1 or greater) and solicited systemic AR (has a severity grade of Grade 1 or greater) during the 7-day follow-up period after each injection will be tabulated by vaccination group, severity grade, and injection. The number and percentage of subjects who reported each individual solicited AR will also be summarized by vaccination group, severity grade, days of reporting and injection.

The number and percentage of subjects experiencing fever (a temperature greater than or equal to 38.0°C/100.4°F by the oral, axillary, or tympanic route) by severity grade will be provided.

A two-sided 95% exact confidence interval (CI) using the Clopper-Pearson method will be provided for the percentage of subjects who reported any solicited local AR, solicited systemic AR, or any solicited AR.

The onset of individual solicited AR is defined as the time point after each injection at which the respective solicited AR first occurred. The number and percentage of subjects with onset of individual solicited AR will be summarized by vaccination group, study day relative to the corresponding injection (Day 1 through Day 7), and injection.

The number of days will be calculated as the days of the solicited AR is reported within the 7 days of injection including the day of injection, no matter it is intermittent or continued. If the solicited AR continues beyond 7 days, the days a solicited AR is reported after 7 days will be included (e.g., an event that lasted 5 days in the first 7 days post injection and 3 days beyond 7 days post injection, the duration will be reported as 8 (5+3) days.)

6.3.3. Pregnancy Tests

A point-of-care urine pregnancy test will be performed at the Screening Visit (Day 0) and before each vaccine dose. At any time, a pregnancy test either via blood or point-of-care urine can be performed, at the discretion of the investigator. A by-subject listing will be provided for pregnancy tests.

6.3.4. Vital Sign Measurements

Vital sign measurements, including systolic and diastolic blood pressures, heart rate, respiratory rate, and body temperature, will be presented in a data listing. The values meeting the toxicity grading criteria (DHHS 2007) will be flagged in the data listing. The abnormalities meeting the toxicity grading criteria (Grade 2 or higher) in any vital sign measurement will be listed separately. If a subject has a vital sign result with Grade 2 or higher abnormality at any post injection visit, then all results of vital sign measurement for that subject will be presented in the listing.

Observed values and changes from baseline for all vital sign measurements will be summarized at each visit by vaccination group as defined in Section 6.1. Shift from baseline in the toxicity grades at each visit and shift from baseline in the toxicity grades to the worst post-baseline result will also be summarized by vaccination group.

6.4. Immunogenicity Analysis

The analyses of immunogenicity will be based on the PP Immunogenicity Subset and Immunogenicity Subset. The PP Immunogenicity Subset is the primary analysis population used in the immunogenicity analyses, unless otherwise specified. Subjects will be included in the treatment group to which they were randomized.

The GMT and geometric mean (GM) level will be calculated using the following formula:

$$10^{\left\{\frac{\sum_{i=1}^{n}\log_{10}(t_i)}{n}\right\}}$$

where $t_1, t_2, \ldots, t_n$ are *n* observed immunogenicity titers or levels.

The geometric mean fold-rise (GMFR) measures the changes in immunogenicity titers or levels within subjects. The GMFR will be calculated using the following formula:

$$10^{\left\{\frac{\sum_{i=1}^{n}\log_{10}\left(v_{ij}/v_{ik}\right)}{n}\right\}} = 10^{\left\{\frac{\sum_{i=1}^{n}\log_{10}(v_{ij})-\log_{10}(v_{ik})}{n}\right\}}$$

where, for *n* subjects, v_{ij} and v_{ik} are observed immunogenicity titers or levels for subject *i* at time points *j* and *k*, $j \neq k$

6.4.1. Sampling of the Immunogenicity Subset

For the primary analysis of immunogenicity, and characterizing immunogenicity of the vaccine, a simple sampling method will be used for measuring bAb and nAb data from a sampled subset of trial participants.

Sampling Plan

The first 550 participants enrolled in Part 1A who meet all the criteria below will be selected.

- The participant is in Full Analysis Set.
- Baseline SARS-CoV-2 status is not missing.

In such case, approximately 362 participants who receive mRNA-1273 will be selected for the Immunogenicity Subset, with a target of 289 participants in the PP Immunogenicity Subset (adjusting for approximately 20% of participants who may be excluded from the PP Immunogenicity Subset, as they may have baseline positive SAR-CoV-2 status, or have no immunogenicity results due to any reason).

For the noninferiority tests of Ab GM and seroresponse rate at Day 57 in adolescents in Study P203 compared with that in young adults (18-25 years of age) from Study P301 receiving mRNA-1273, an immunogenicity subset of 340 young adults from Study P301

will be randomly selected from all participants (18-25 years of age) receiving mRNA-1273, with a target of 289 participants in the PP Immunogenicity Subset (using same definition as in Study P203) after adjusting for approximately 15% of participants not meeting inclusion criteria for PP Immunogenicity Subset.

6.4.2. Immunogenicity Assessments

There will be two types of immunogenicity assessments:

- Serum bAb by a multiplex serology assay specific to the SARS-CoV-2 S protein
- Serum nAb titer against SARS-CoV-2 as measured by pseudovirus and/or live virus neutralization assays

6.4.3. Primary Analysis of Antibody-Mediated Immunogenicity Endpoints

If an accepted serum Ab threshold of protection against COVID-19 is available based on data from other mRNA-1273 studies or external data, the number and percentage of participants with Ab greater than or equal to the threshold at Day 57 will be provided with a 2-sided 95% CI using the Clopper-Pearson method. If the lower bound of the 95% CI on the mRNA-1273 group is > 70%, the primary immunogenicity objective of this study will be considered to be met.

The percentage of participants with serum Ab greater than or equal to the threshold with 95% CI will be provided at each postbaseline time point. The CI will be calculated using the Clopper-Pearson method.

If an accepted serum Ab threshold of protection against COVID-19 is not established, the noninferiority of primary vaccine response as measured by Ab GM and seroresponse rate in adolescents compared with those in young adults (18-25 years of age) receiving mRNA-1273 will be assessed. The study is considered to meet the primary immunogenicity objective if the noninferiority of the immune response to mRNA-1273 as measured by both GM and seroresponse rate at Day 57 is demonstrated in adolescents in this study at a 2-sided alpha of 0.05, compared with those in young adults (18-25 years of age) in Study P301 receiving mRNA-1273.

An ANCOVA model will be carried out with Ab at Day 57 as a dependent variable and a group variable (adolescents in P203 and young adults in P301) as the fixed variable. The GM values of the adolescents at Day 57 will be estimated by the geometric least square mean (GLSM) from the model. The GMR (ratio of GM values) will be estimated by the ratio of GLSM from the model. A corresponding 2-sided 95% CI will be provided to assess the

difference in immune response for the adolescents in P203 compared to the young adults of 18-25 years of age in P301 at Day 57. The noninferiority of immune response to mRNA-1273 as measured by GM will be considered demonstrated if the lower bound of the 95% CI of the GMR is > 0.67 based on the noninferiority margin of 1.5, and GMR point estimate > 0.8 (minimum threshold).

The number and percentage (rate) of participants achieving Ab seroresponse at Day 57 will be summarized. The difference of seroresponse rates between adolescents receiving mRNA-1273 in P203 and young adults of 18-25 years of age receiving mRN-1273 in P301 will be calculated with 95% CI. The noninferiority in seroresponse rate of adolescents in P203 compared to adults of 18-25 years of age in P301 will be considered demonstrated if the lower bound of the 95% of the seroresponse rate difference is > -10% based on the noninferiority margin of 10%, and the seroresponse rate difference point estimate > -5% (minimum threshold).

6.4.4. Secondary Analysis of Antibody-Mediated Immunogenicity Endpoints

For each group, the following evaluations will be performed at each time point at which blood samples are collected for immunogenicity (unless otherwise specified).

- GM level of SARS-CoV-2-specific bAb levels with corresponding 95% CI will be provided at each time point. The 95% CIs will be calculated based on the t-distribution of the log-transformed values then back transformed to the original scale for presentation. GM level and corresponding 95% CI will be plotted at each timepoint. The following descriptive statistics will be also provided at each time point: the number of subjects (n), median, minimum and maximum.
- GM fold-rise of SARS-CoV-2-specific bAb levels with corresponding 95% CI will be provided at each post-baseline timepoint over pre-injection baseline at Day 1. The 95% CIs will be calculated based on the t-distribution of the log-transformed values then back transformed to the original scale for presentation. GM fold-rise and corresponding 95% CI will be plotted at each timepoint. The following descriptive statistics will be also provided at each time point: the number of subjects (n), median, minimum and maximum.

  Proportion of subjects with fold-rise ≥ 2, and fold-rise ≥ 4 of serum SARS-CoV-2 specific bAb levels from Visit Day 1 (baseline) at each post injection time points will be tabulated with 2-sided 95% Clopper Pearson CIs.

- GMT of SARS-CoV-2-specific nAb titers with corresponding 95% CI will be provided at each time point using the same method mentioned above.
- GMFR of SARS-CoV-2-specific nAb titers with corresponding 95% CI will be provided at each post-baseline timepoint over pre-injection baseline at Day 1 using the same method mentioned above.

  Proportion of subjects with fold-rise ≥ 2, and fold-rise ≥ 4 of serum SARS-CoV-2-specific nAb titers from Visit Day 1 (baseline) at each post-injection time points will be provided using the same method mentioned above.
- Proportion of subjects with seroresponse due to vaccination will be tabulated with 2-sided 95% Clopper-Pearson CIs at each post-baseline timepoint.
- Per the study protocol, if the visit for the second dose (Day 29) is disrupted and cannot be completed at Day 29 (+7 days) as a result of the COVID-19 pandemic (self-quarantine or disruption of clinical site activities following business continuity plans and/or local government mandates for “stay at home” or “shelter in place”), the window may be extended to Day 29 + 21 days. More rigid visit window may be used in the Per-protocol Immunogenicity Subset as appropriate.

6.5. Efficacy Analysis

Efficacy analyses will be performed using the FAS, mITT, mITT1 and PP Set for Efficacy. The mITT1 Set is the primary analysis set for efficacy analysis of cases starting from 14 days after first dose, and PP Set for Efficacy is the primary analysis set used in the efficacy analyses for cases starting 14 days after second dose, unless otherwise specified. Subjects will be included in the treatment group which they were randomized.

Baseline SARS-CoV-2 status is described in Section 6.1. Baseline SARS-CoV-2 status, the serology test results based on *Roche Elecsys* assay at baseline, the RT-PCR test results at baseline will be summarized by treatment group.

Participants with baseline positive or missing SARS-CoV-2 status will be excluded from the PP Set for Efficacy.

In this study, the serology test results based on Roche Elecsys assay and the RT-PCR test results will be summarized by visit.

6.5.1. Endpoint Definition/Derivation

6.5.1.1.Derivation of SARS-CoV-2 Infection

This is a secondary efficacy endpoint, which is a combination of COVID-19 and asymptomatic SARS-CoV-2 infection for participants with negative SARS-CoV-2 status at baseline: the incidence of SARS-CoV-2 infection counted starting 14 days after the second dose of IP will be summarized by treatment group. SARS-CoV-2 infection will be defined in participants with negative SARS-CoV-2 at baseline:

- bAb level against SARS-CoV-2 nucleocapsid protein negative (as measured by *Roche Elecsys*) at Day 1 that becomes positive (as measured by *Roche Elecsys*) counted starting at Day 57 or later, OR
- Positive RT-PCR counted starting 14 days after the second dose of IP.

During the analysis, documented infection is counted starting 14 days after the second dose of IP, which requires positive serology test result based on bAb specific to SARS-CoV-2 nucleocapsid at Day 57 visit or later, or a positive RT-PCR result starting 14 days after the second dose of IP. Derivation of this secondary efficacy endpoint is summarized in Table 2 below.

Table 2. Derivation for SARS-CoV-2 Infection

	**Post-baseline assessments**		
**Baseline SARS-CoV-2 Status**	PCR test post baseline	bAb levels against SARS-CoV-2 Nucleocapsid	**Endpoint**: SARS-CoV-2 infection
Negative at Baseline	**Positive** (either at scheduled NP swab test, or at symptom-prompt NP swab test)		Case
Negative at Baseline		**Positive** (at scheduled Day 57 visit or later) as measured by *Roche Elecsys*	Case

The date of documented infection will be the earlier of:

- Date of positive post-baseline RT-PCR result, or
- Date of positive serology test result based on bAb specific to SARS-CoV-2 nucleocapsid

The time to the first SARS-CoV-2 infection will be calculated as:

Time to the 1st SARS-CoV-2 infection = Date of the 1st documented infection – Date of randomization + 1.

Cases will be counted starting 14 days after the second injection, i.e. date of documented infection - Date of the 2nd injection ≥ 14.

SARS-CoV-2 infection cases will also be summarized based on tests performed at least 14 days after first dose of IP.

6.5.1.2.Derivation of Asymptomatic SARS-CoV-2 Infection

This is a secondary efficacy endpoint: the incidence of asymptomatic SARS-CoV-2 infection measured by RT-PCR and/or serology tests obtained at post-baseline visits counted starting 14 days after the second injection in participants with negative SARS-COV-2 status at baseline.

Asymptomatic SARS-CoV-2 infection is identified by absence of symptoms and infections as detected by RT-PCR or serology tests. Specifically:

- Absence of COVID-19 symptoms
- AND at least one from below:
  - bAb level against SARS-CoV-2 nucleocapsid protein negative (as measured by Roche Elecsys) at Day 1 that becomes positive (as measured by Roche Elecsys) counted starting at Day 57 or later, OR
  - Positive RT-PCR test at scheduled or unscheduled/illness visits

The date of documented asymptomatic infection is the earlier date of positive serology test result based on bAb specific to SARS-CoV-2 nucleocapsid due to infection, or positive RT-PCR at scheduled visits, with absence of symptoms.

The time to the asymptomatic SARS-CoV-2 infection will be calculated as:

Time to the asymptomatic SARS-CoV-2 infection = Date of asymptomatic SARS-CoV-2 infection test – Date of randomization + 1.

Asymptomatic SARS-CoV-2 infection cases will also be summarized based on tests performed at least 14 days after first dose of IP.

6.5.1.3.Derivation of COVID-19

This is a secondary efficacy endpoint: the incidence of the first occurrence of COVID-19 starting 14 days after the second dose of IP. COVID-19 is defined as symptomatic disease based on the criteria specified in Section 3.2. Cases are defined as participants meeting clinical criteria based on both symptoms for COVID-19 and positive RT-PCR test results.

Surveillance for COVID-19 symptoms will be conducted via biweekly telephone calls or eDiary. Subjects reporting COVID-19 symptoms, as defined in Section 7.3.2 of the protocol, will be arranged an illness visit to collect an NP swab.

For this efficacy endpoint, a COVID-19 case will be identified as a positive post-baseline RT-PCR test result, together with eligible symptoms, i.e. a positive PCR result of the eligible symptoms summarized below in Table 3.

Table 3. Derivation for COVID-19 (P301 primary case definition)

	**COVID-19**
Post-baseline PCR result	Positive, **AND**
Systemic Symptoms	at least **TWO** of the following **systemic symptoms**: Fever (≥ 38ºC/≥ 100.4º F), chills, myalgia, headache, sore throat, new olfactory and taste disorder(s); **OR**
Respiratory symptoms	at least **ONE** of the following **respiratory** signs/symptoms: cough, shortness of breath or difficulty breathing, OR clinical or radiographical evidence of pneumonia.

The date of documented COVID-19 (case) will be the later date of ([2 systemic symptoms reported, or respiratory symptom reported] and, [date of positive PCR test]). Specifically, the date of documented COVID-19 will be the later date of the following two dates (date of positive PCR test, and the date of eligible symptom(s)), and the two dates should be within 14 days of each other.

- Date of positive PCR test,
- Date of eligible symptom(s), defined as earliest of
    - Respiratory symptom: earliest date of an eligible respiratory symptom is reported
    - Systemic symptoms: earliest date of 2 eligible systemic symptom is reported

The time to the first occurrence of COVID-19 will be calculated as:

Time to the 1st occurrence of COVID-19 = Date of documented COVID-19 – Date of randomization + 1.

Cases will be counted starting 14 days after the second injection, i.e. date of documented COVID-19 - Date of the 2nd injection ≥ 14.

6.5.1.4.Derivation of Secondary Case Definition (CDC case definition) of COVID-19

This is a secondary efficacy endpoint: the incidence of the first occurrence of COVID-19 cases meeting the secondary case definition, starting 14 days after the first dose of IP, and COVID-19 cases starting 14 days after the second dose of IP.

The secondary case definition of COVID-19 is defined by the following criteria:

- One systemic or respiratory symptoms: fever (temperature > 38ºC/≥ 100.4ºF), or chills, cough, shortness of breath or difficulty breathing, fatigue, muscle aches, or body aches, headache, new loss of taste or smell, sore throat, congestion or runny nose, nausea, or vomiting or diarrhea, AND
- At least one positive RT-PCR test for SARS CoV-2

Date of the documented secondary definition of COVID-19 will be later date of:

- Date of the positive RT-PCR test (prompt by symptom)
- Date of eligible symptom for secondary definition of COVID-19, defined as the earliest date of first eligible symptom is reported

and the two dates should be within 14 days of each other.

Secondary case definition of COVID-19 cases will also be summarized based on tests performed after randomization, and based on tests performed at least 14 days after first dose of IP.

6.5.2. Analysis Method

The number and percentage of subjects who had an event will be summarized in the PP Set for Efficacy.

The incidence rate will be provided by vaccination group, calculated as the number of cases divided by the total person-time. The 95% CI of the incidence rate will be calculated using the exact method (Poisson distribution) and adjusted by person-time.

VE is defined as 1 – ratio of incidence rate (mRNA-1273 vs. placebo). The 95% CI of the ratio will be calculated using the exact method conditional upon the total number of cases adjusted by the total person-time.

Person-time is defined as the total time from randomization date to the date of event, last date of study participation, censoring time, or efficacy data cutoff date, whichever is earlier.

Incidence rate and VE will also be analyzed by time period or by calendar month.

6.5.3. Sensitivity Analysis

Sensitivity analysis for these efficacy endpoints will be performed with the same methods described above based on the FAS, mITT Set, and mITT1 Set, and with cases counted starting at different time points.

6.6. Long-term Analysis (including Part 1B)

Long-term analysis will be performed including data collected in the Open-label Observational Phase (Part 1B), and prior to BD if a BD is received. The analysis will include participants who received mRNA-1273 in Part 1A and remained in the study with or without unblinding, and participants who received placebo in Part 1A and received mRNA-1273 in Part 1B after unblinding. Long-term analysis of applicable safety, efficacy, and immunogenicity endpoints will be summarized descriptively by treatment cohort without treatment group comparison.

In the long-term safety analysis, unsolicited AEs (SAE, AESI, MAAE, and AE leading to discontinuation) and deaths will be summarized.

In the long-term immunogenicity analysis, nAb and bAb values will be summarized at specified timepoints.

In the long-term efficacy analysis, the incidence rates of COVID-19 and of SARS-CoV-2 infection cases will be counted starting 14 days after the second dose of IP for participants in treatment cohorts of mRNA-1273 and Placebo, or starting 14 days after the second dose of mRNA-1273 for participants in the Placebo-mRNA-1273 Cohort. Incidence rate with 95% CI adjusting for person-time will be provided. The incidence rate of asymptomatic SARS-CoV-2 infection will also be provided.

Incidence rate will be analyzed by calendar month in the Long-term analysis.

Additional details for the Long-term analysis will be provided in a separate SAP (Part 1B, Part 1C, Part 2 and Part 3).

Table 4. Treatment Cohorts for the Long-term Analysis

Long-term Treatment Cohort	Description
**mRNA-1273**	Participants randomized to mRNA-1273 in the Blinded Phase.
**Placebo-mRNA-1273**	Participants randomized to Placebo in the Blinded phase who crossed over to mRNA-1273 in the Open-label Observational Phase.

6.7. Booster Dose Analysis

Booster Phase Analysis will be described in a separate SAP (Part 1B, Part 1C, Part 2 and Part 3).

6.8. Part 2 Analysis

Part 2 Analysis will be described in a separate SAP (Part 1B, Part 1C, Part 2 and Part 3).

6.9. Part 3 Analysis

Part 3 Analysis will be described in a separate SAP (Part 1B, Part 1C, Part 2 and Part 3).

6.10. Exploratory Analysis

6.10.1. Exploratory Analysis of Immunogenicity

The below exploratory analyses of immunogenicity may be performed:

- The genetic and/or phenotypic relationships of isolated SARS-CoV-2 strains to the vaccine sequence.

- Descriptive summaries of the ratio or profile of specific bAb relative to nAb in serum during the study. The analysis may not be included in the Clinical Study Report (CSR).
- Descriptive summaries of clinical profile and immunologic endpoints to characterize participants with SARS-CoV-2 infection during the study.
- Descriptive summaries of GM and GMFR of bAb levels against SARS-CoV-2 nucleocapsid protein (quantitative IgG) and % of participants with 2x, 3x and 4x rise of bAb relative to baseline)
- The seroresponse rate comparisons between children in P203 and young adults (18-25 years of age) in P301 may be performed using the bAb and nAb measures based on assay-specific seroresponse definitions listed in the table below.

  The definition of assay-specific seroresponse may depend on assay-specific performance characteristics and may defined as a change from below the LLOQ to equal to or above LLOQ, or a z-fold rise if baseline is equal to or above LLOQ. The table 5 below lists the assay-specific definition of seroresponse for select assay/test with established assay-specific seroresponse definition.

Table 5 Assay-specific Definition of Seroresponse

Assay Name	Category	Test Name/ Description	Definition of Seroresponse
Pseudovirus (PsVNT)	nAb	PsVNT50 (ID 50)	baseline <LLOQ: >=LLOQ<br>baseline >=LLOQ: 3.3-foldrise
		PsVNT80 (ID 80)	baseline <LLOQ: >=LLOQ<br>baseline >=LLOQ: 2.3-foldrise
Anti-Spike ELISA	bAb	Anti-Spike VAC65 Spike IgG Antibody	baseline <LLOQ: >=LLOQ<br>baseline >=LLOQ: 4.6-foldrise
MSD multiplex	bAb	Anti-Spike	baseline <LLOQ: >=LLOQ<br>baseline >=LLOQ: 1.9-foldrise<br>established based on MSD multiplex anti-S by VRC

6.10.2. SARS-CoV-2 Exposure and Symptoms

SARS-CoV-2 reported exposure history and symptoms assessment will be assessed during the study.

The number and percentage of subjects who had close contact with a person with SARS-CoV-2 infection, reasons for exposure, subjects with any symptoms of potential COVID-19, and subjects with each symptoms will be presented by visit and vaccination group as defined in Section 6.1. Descriptive statistics will be provided for length of exposure in days by vaccination group.

In addition, the following listings will be provided for subjects infected by SARS-CoV-2:

- Serum bAb level against SARS-CoV-2
- Serum nAb titer against SARS-CoV-2
- Solicited ARs
- Unsolicited AEs

6.11. Interim Analysis

Study Day 57 is the primary time point for assessment of immunogenicity in this study. More than one interim analysis of immunogenicity, safety and efficacy data will be performed.

- The IA of immunogenicity, safety, and efficacy will be performed after Day 57 immunogenicity data are available for the immunogenicity subset and at least 1,500 participants (1,000 participants receiving mRNA-1273) have completed Day 57 (1 month after Dose 2, Part 1A). This IA will be considered the primary analysis of immunogenicity for Part 1A.
- An IA of immunogenicity and safety may be performed after all or subset of participants who receive booster dose have completed BD-Day 29 after the BD in Part 1C-1. More details of this IA will be described in the SAP for Part 1B, Part 1C, Part 2 and Part 3.
- An IA of immunogenicity and safety may be performed after all or subset of participants who receive heterologous BD have completed BD-Day 29 after the BD in Part 1C-2. More details of this IA will be described in the SAP for Part 1B, Part 1C, Part 2 and Part 3.

- An IA of immunogenicity and safety may be performed after Day 57 immunogenicity data are available after all or a subset of participants in Part 2 have completed Day 57 (1 month after Dose 2, Part 2). More details of this IA will be described in the SAP for Part 1B, Part 1C, Part 2 and Part 3.
- An IA of immunogenicity and safety may be performed after all or a subset of participants who receive 50 µg mRNA-1273.222 dose have completed Day 29 (1 month after Dose 1) and an additional IA may be performed in all or a subset of participants who completed Day 209 (1 month after Dose 2) in Part 3. More details of this IA will be described in the SAP for Part 1B, Part 1C, Part 2 and Part 3.
- At the Sponsor's discretion, a CSR may be developed for an IA.

6.12. Final Analysis

The final analysis of all applicable endpoints will be performed after all participants have completed all planned study procedures. Results of this analysis will be presented in a final CSR, including individual listings.

6.13. Data Safety Monitoring Board

Safety oversight will be under the direction of a DSMB composed of external independent consultants with relevant expertise. Members of the DSMB will be independent from the study conduct and free of conflict of interest. The DSMB will have separate meetings by teleconference to review unblinded safety data when half of the study population (1,500 randomized participants) have reached Day 8 (1 week after dose 1) and again approximately when 25% (750), 50% (1,500), and 75% (2,250) of enrolled participants have reached Day 36 (1 week after dose 2). Recruitment will continue, as applicable, during the DSMB review period. The DSMB will operate under the rules of an approved charter that will be written and reviewed at the organizational meeting of the DSMB. Details regarding the DSMB composition, responsibilities, procedures, and frequency of data review will be defined in its charter.

The DSMB will convene on an ad hoc basis if any of the pause rules, described in protocol Section 6.4, are met. The DSMB will review all available unblinded study data to adjudicate any potential study pauses and make recommendations on further study conduct, including requesting additional information, recommending stopping the study, recommending

changes to study conduct and/or the protocol, or recommending additional operational considerations due to safety issues that arise during the study.

7. Changes from Planned Analyses in Protocol

In Section 4.2, for the noninferiority in Ab GM in adolescents in P203 compared with that in young adults (18-25 years of age in P301), an additional success criterion on point estimate has been added in this SAP: the GMR point estimate > 0.8 (minimum threshold). Similarly, for the noninferiority in seroresponse rate in adolescents compared with that in adults (18-25 years of age), an additional success criterion on point estimate has been added in this SAP: the seroresponse rate difference point estimate > -5% (minimum threshold).

8. References

Department of Health and Human Services (DHHS), Food and Drug Administration, Center for Biologics Evaluation and Research (US). Guidance for industry: Toxicity grading scale for healthy adult and adolescent volunteers enrolled in preventative vaccine clinical trials. September 2007 [cited 2019 Apr 10] [10 screens].

Available from: https://www.fda.gov/downloads/BiologicsBloodVaccines/GuidanceComplianceRegulatoryInformation/Guidances/Vaccines/ucm091977.pdf.List of Appendices

Centers for Disease Control and Prevention (CDC). 2022. People with Certain Medical Conditions. Retrieved from: https://www.cdc.gov/coronavirus/2019-ncov/need-extra-precautions/people-with-medical-conditions.html. (accessed 2022 Feb 28).

Kompaniyets L, et al., Underlying Medical Conditions Associated With Severe COVID-19 Illness Among Children. 2021 JAMA Netw Open 4 e2111182.

9. List of Appendices

9.1. Appendix A Standards for Safety and Immunogenicity Variable Display in TFLs

Continuous Variables: The precision for continuous variables will be based on the precision of the data itself. The mean and median will be presented to one decimal place more than the original results; the SD will be presented to two decimal places more than the original results; the minimum and maximum will be presented to the same precision as the original results.

Categorical Variables: Percentages will be presented to 1 decimal place.

9.2. Appendix B Analysis Visit Windows for Safety and Immunogenicity Analysis

Safety and Immunogenicity Analysis will be summarized using the following analysis visit window for post injection assessments:

Step 1: If the safety and immunogenicity assessments are collected at scheduled visit, i.e. nominal scheduled visit, the data collected at scheduled visit will be used.

Step 2: If the safety and immunogenicity assessments are not collected at the scheduled visit, assessments collected at unscheduled visit will be used using the analysis visit windows described in Table 6 below.

If a subject has multiple assessments within the same analysis visit, the following rule will be used:

- If multiple assessments occur within a given analysis visit, the assessment closest to the target study day will be used.
- If there are 2 or more assessments equal distance to the target study day, the last assessment will be used.

Table 6. Visit Window

Visit	Target Study Day	Visit Window in Study Day
**Nasopharyngeal or Nasal Swabs for SARS-CoV-2**		
Day 1	1 (Date of First Injection)	1, Pre-first-dose

Day 29 (Month 1)	29 (Date of Second Injection)	[2, 43]
Day 57 (Month 2)	57	[44, 133]
Day 209	209	≥134 and before earlier date of (unblinding, first injection in Part 1B, injection in Part 1C)
**Vital Signs**		
Day 1	1 (Date of First Injection)	≤1, Pre-first-dose
Day 1	1 (Date of First Injection)	1, Post-first-dose
Day 29 (Month 1)	29 (Date of Second Injection)	[2, 43] Pre-second-dose
Day 29 (Month 1)	29 (Date of Second Injection)	[2, 43] Post-second-dose
Day 57 (Month 2)	57	[44, 133]
Day 209 (Month 7)	209	[134, 301]
Day 394 (Month 13)	394	≥302 and before earlier date of (unblinding, first injection in Part 1B, injection in Part 1C)
**Immunogenicity**		
Day 1	1 (Date of First Injection)	1, Pre-first-dose
Day 57 (Month 2)	57	[44,133]
Day 209 (Month 7)	209	[134, 301]
Day 394 (Month 13)	394	≥302 and before earlier date of (unblinding, first injection in Part 1B, injection in Part 1C)

9.3. Appendix C Imputation Rules for Missing Prior/Concomitant Medications and Non-Study Vaccinations

Imputation rules for missing or partial medication start/stop dates are defined below:

1. Missing or partial medication start date:

    - If only Day is missing, use the first day of the month, unless:

    - The medication end date is after the date of first injection or is missing AND the start month and year of the medication coincide with the start month and year of the first injection. In this case, use the date of first injection

- If Day and Month are both missing, use the first day of the year, unless:
    - The medication end date is after the date of first injection or is missing AND the start year of the medication coincide with the start year of the first injection. In this case, use the date of first injection

- If Day, Month and Year are all missing, the date will not be imputed, but the medication will be treated as though it began prior to the first injection for purposes of determining if status as prior or concomitant.

2. Missing or partial medication stop date:

- If only Day is missing, use the earliest date of (last day of the month, study completion, discontinuation from the study, or death).

- If Day and Month are both missing, use the earliest date of (last day of the year, study completion, discontinuation from the study, or death).

- If Day, Month and Year are all missing, the date will not be imputed, but the medication will be flagged as a continuing medication.

In summary, the prior, concomitant or post categorization of a medication is described in Table 7 below.

Table 7. Prior, Concomitant, and Post Categorization of Medications and Non-study Vaccinations

	**Medication Stop Date**		
**Medication Start Date**	**< First Injection Date of IP**	**≥ First Injection Date and ≤ 28 Days After Last Injection**	**> 28 Days After Last Injection [2]**
< First injection date of IP [1]	P	P, C	P, C, A
≥ First injection date and ≤ 28 days after last injection	-	C	C, A
> 28 days after last injection	-	-	A

A: Post; C: Concomitant; P: Prior
[1] includes medications with completely missing start date
[2] includes medications with completely missing end date

9.4. Appendix D Imputation Rules for Missing AE dates

Imputation rules for missing or partial AE start dates and stop dates are defined below:

1. Missing or partial AE start date:

- If only Day is missing, use the first day of the month, unless:
  - o The AE end date is after the date of first injection or is missing AND the start month and year of the AE coincide with the start month and year of the first injection. In this case, use the date and time of first injection, even if time is collected.
- If Day and Month are both missing, use the first day of the year, unless:
  - o The AE end date is after the date of first injection or is missing AND the start year of the AE coincides with the start year of the first injection. In this case, use the date of first injection

- If Day, Month and Year are all missing, the date will not be imputed. However, if the AE end date is prior to the date of first injection, then the AE will be considered a pre-treatment AE. Otherwise, the AE will be considered treatment-emergent.

2. Missing or partial AE end dates will not be imputed.

9.5. Appendix E Schedule of Assessments (Part 1A, the Blinded Phase; Part 1B Open-label Observational Phase for Participants who Received mRNA-1273 in Part 1A)

**Visit Number**	0	1	2	3	4	5	-		6	-		7
**Type of Visit**	C	C	Virtual Call	C	Virtual Call	C	SFU		C	SFU		C
**Month Timepoint**		M0		M1		M2	eDiary	SC	M7	eDiary	SC	M13
**Study Visit Day**	D0[1] (Screening)	D1 (Baseline)	D8[2]	D29[3]	D36[2, 3]	D57[2, 3]	Every 4 weeks D71 – D183[3, 4]	Every 4 weeks D85– D197[3, 5]	D209/ Participant Decision Visit[3,6]	Every 4 weeks D223– D363[3, 4]	Every 4 weeks D237– D377[3, 5]	D394[3]
**Window Allowance (Days)**	- 28		+ 3	+ 7	+ 3	+ 7	±3	± 3	- 56/+ 56	± 3	± 3	± 14
**Days Since Most Recent Injection**	-	0	7	28/0	7	28	-	-	180	-	-	365
Informed consent/assent form, demographics, concomitant medications, medical history	X											
Revised informed consent/assent form									X			
Review of inclusion and exclusion criteria	X	X										

**Visit Number**	0	1	2	3	4	5	-		6	-		7
**Type of Visit**	C	C	Virtual Call	C	Virtual Call	C	SFU		C	SFU		C
**Month Timepoint**		M0		M1		M2	eDiary	SC	M7	eDiary	SC	M13
**Study Visit Day**	D0[1] (Screening)	D1 (Baseline)	D8[2]	D29[3]	D36[2, 3]	D57[2, 3]	Every 4 weeks D71 – D183[3, 4]	Every 4 weeks D85– D197[3, 5]	D209/ Participant Decision Visit [3,6]	Every 4 weeks D223– D363[3, 4]	Every 4 weeks D237– D377[3, 5]	D394[3]
**Window Allowance (Days)**	- 28		+ 3	+ 7	+ 3	+ 7	±3	± 3	- 56/+ 56	± 3	± 3	± 14
**Days Since Most Recent Injection**	-	0	7	28/0	7	28	-	-	180	-	-	365
Physical examination including vital signs, height, weight[7]	X	X		X		X			X			X
Pregnancy test[8]	X	X		X								
Randomization		X										
Study injection (including 30-minute post-dose observation period)		X		X								
Blood sample for vaccine immunogenicity[9]		X				X			X			X
Nasopharyngeal or nasal swab sample for SARS-CoV-2[10]		X		X		X			X			
Surveillance for COVID-19/ Illness visit[11]/ Unscheduled visit		X	X	X	X	X	X	X	X	X	X	X
Convalescent Visit[12]		X	X	X	X	X	X	X	X	X	X	X

Visit Number	0	1	2	3	4	5	-		6	-		7
Type of Visit	C	C	Virtual Call	C	Virtual Call	C	SFU		C	SFU		C
Month Timepoint		M0		M1		M2	eDiary	SC	M7	eDiary	SC	M13
Study Visit Day	D0[1] (Screening)	D1 (Baseline)	D8[2]	D29[3]	D36[2, 3]	D57[2, 3]	Every 4 weeks D71 – D183[3, 4]	Every 4 weeks D85– D197[3, 5]	D209/ Participant Decision Visit [3,6]	Every 4 weeks D223– D363[3, 4]	Every 4 weeks D237– D377[3, 5]	D394[3]
Window Allowance (Days)	- 28		+ 3	+ 7	+ 3	+ 7	±3	± 3	- 56/+ 56	± 3	± 3	± 14
Days Since Most Recent Injection	-	0	7	28/0	7	28	-	-	180	-	-	365
eDiary activation for recording solicited ARs (7 days)[13]		X		X								
Review of eDiary data			X		X							
Follow-up safety telephone calls[14]								X		X		
Recording of unsolicited AEs		X	X	X	X	X						
Recording of MAAEs and concomitant medications relevant to or for the treatment of the MAAE[15]		X	X	X	X	X	X		X	X		X
Recording of SAEs and concomitant medications relevant to or for the treatment of the SAE[15]		X	X	X	X	X	X		X	X		X
Recording of AESI		X	X	X	X	X	X		X	X		X

**Visit Number**	0	1	2	3	4	5	-		6	-		7
**Type of Visit**	C	C	Virtual Call	C	Virtual Call	C	SFU		C	SFU		C
**Month Timepoint**		M0		M1		M2	eDiary	SC	M7	eDiary	SC	M13
**Study Visit Day**	D0[1] (Screening)	D1 (Baseline)	D8[2]	D29[3]	D36[2, 3]	D57[2, 3]	Every 4 weeks D71 – D183[3, 4]	Every 4 weeks D85– D197[3, 5]	D209/ Participant Decision Visit [3,6]	Every 4 weeks D223– D363[3, 4]	Every 4 weeks D237– D377[3, 5]	D394[3]
**Window Allowance (Days)**	- 28		+ 3	+ 7	+ 3	+ 7	±3	± 3	- 56/+ 56	± 3	± 3	± 14
**Days Since Most Recent Injection**	-	0	7	28/0	7	28	-	-	180	-	-	365
Recording of concomitant medications and nonstudy vaccinations[15]		X	X	X	X	X						
Study completion												X

Abbreviations: AE = adverse event; AESI = adverse event of special interest; AR = adverse reaction; C = clinic visit; COVID-19 = coronavirus disease 2019; D = day; eDiary = electronic diary; FDA = US Food and Drug Administration; IRB = institutional review board; M = month; MAAE = medically attended AE; SC = safety (telephone) call; SFU = safety follow-up; RT-PCR = reverse transcriptase polymerase chain reaction; SAE = serious adverse event; SARS-CoV-2 = Severe Acute Respiratory Syndrome coronavirus 2.

Note: In accordance with FDA Guidance on Conduct of Clinical Trials of Medical Products during COVID-19 Public Health Emergency (DHHS 2020), investigators may convert study site visits to home visits or telemedicine visits with the approval of the Sponsor.

Note: If during the Participant Decision Visit, the participant is unblinded and determined to have received 2 doses of mRNA-1273 in Part 1A, due to statistical considerations, they will be considered in the Open-label Observational Phase but will continue to follow the Part 1A SoA.

1. Day 0 and Day 1 may be combined on the same day. Additionally, the Day 0 visit may be performed over multiple visits if preformed within the 28-day Screening window.
2. All scheduled study visits should be completed within the respective visit windows. If the participant is not able to attend a study site visit as a result of the COVID-19 pandemic (self-quarantine or disruption of study site activities following business continuity plans and/or local government mandates for “stay at home” or “shelter in place”), a safety telephone call to the participant should be made in place of the study site visit. The safety telephone call should encompass all scheduled visit assessments that can be completed remotely, such as assessment for AEs and concomitant medications (eg, as defined in scheduled safety telephone calls). Home visits will be permitted for all nondosing visits, except for Screening, if a participant cannot visit the study site due to the COVID-19 pandemic. Home visits must be permitted by the study site IRB and the participant via informed consent/assent and have prior approval from the Sponsor (or its designee).
3. If the visit for the second dose (Day 29) is disrupted and cannot be completed at Day 29 +7 days due to the COVID-19 pandemic (self-quarantine or disruption of study site activities following business continuity plans and/or local government mandates for “stay at home” or “shelter in place”), the visit window may be

extended to Day 29 + 21 days. When the extended visit window is used, the remaining study visits should be rescheduled to follow the inter-visit interval from the actual date of the second dose.

4. Safety follow-up via an eDiary questionnaire will be performed every 4 weeks from Day 71 to Day 183 and again from Day 223 to Day 363.
5. Safety follow-up via a safety telephone call will be performed every 4 weeks from Day 85 to Day 197 and again from Day 237 to Day 377.
6. The Participant Decision Visit may be performed over multiple visits. Once the Participant Decision Visit has been initiated, all assessments must be completed within a 7-day period.
7. Physical examination: A full physical examination, including height and weight, will be performed at Day 1, Day 29, Day 57, Day 209, and Day 394. BMI will be calculated only at Screening Visit (Day 0). Symptom-directed physical examinations may be performed at other timepoints at the discretion of the investigator. On each injection day before injection and again 7 days after injection, the arm receiving the injection should be examined and the associated lymph nodes should be evaluated. Any clinically significant finding identified during a study visit should be reported as an MAAE. Vital signs are to be measured predose and postdose on days of injection (Day 1 and Day 29). When applicable, vital signs should be measured before blood collection. Participants who are febrile (body temperature ≥ 38.0°C/100.4°F) before injection on Day 1 or Day 29 must have the visit rescheduled within the relevant visit window to receive the injection. Afebrile participants with minor illnesses can be enrolled at the discretion of the investigator.
8. Pregnancy test at Screening and Day 1 and before the second study injection will be a point-of-care urine test. At the discretion of the investigator, a pregnancy test either via blood or point-of-care urine test can be performed at any time.
9. Sample must be collected prior to dosing of injection on Day 1.
10. The nasopharyngeal or nasal swab sample will be used to ascertain the presence of SARS-CoV-2 via RT-PCR.
11. An unscheduled visit may be prompted by reactogenicity issues, illness visit criteria for COVID-19, or new or ongoing AEs. If a participant meets the prespecified criteria of suspicion for COVID-19 (Section 7.1.6), the participants will be asked to return within 72 hours or as soon as possible to the study site for an unscheduled illness visit, to include an NP or nasal swab sample (for RT-PCR testing) and other clinical evaluations. If a study site visit is not possible, a home visit may be arranged to collect the NP or nasal sample and conduct clinical evaluations. If a home visit is not possible, the participant will be asked to submit a saliva sample to the study site by a Sponsor-approved method. At this illness visit, the NP or nasal swab samples will be collected to evaluate for the presence of SARS-CoV-2 infection. NP or nasal swab samples will also be tested for the presence of other respiratory pathogens. If due to COVID-19 related restrictions in the community, the participant's LAR performed a home test rather than obtaining a test performed by a health care professional, see Section 7.1.6. In addition, the study site may collect an additional NP or nasal sample for SARS-CoV-2 testing to be able to render appropriate medical care for the study participant as determined by local standards of care. Additionally, clinical information will be carefully collected to evaluate the severity of the clinical case.
12. A convalescent visit will be scheduled approximately 28 days (+7 days) after diagnosis. At this visit, an NP or nasal swab sampling for viral PCR and a blood sample will be collected for potential immunologic assessment of SARS-CoV-2 infection.
13. Diary entries will be recorded by the participant at approximately 30 minutes after injection while at the study site with instruction provided by study staff. Study participants will continue to record entries in the eDiary each day after they leave the study site, preferably in the evening, on the day of injection and for 6 days following injection. If a solicited local or systemic AR continues beyond Day 7 after vaccination, the participant will be prompted to capture details of the solicited local or systemic AR in the eDiary until the AR is resolves or the next IP injection occurs, whichever occurs first; capture of details of ARs in the eDiary should not exceed 28 days after each vaccination. ARs recorded in eDiaries beyond Day 7 should be reviewed either via telephone call or at the following study visit.
14. Trained study site personnel will call all participants to collect information relating to any AEs, MAAEs, SAEs, AEs leading to study withdrawal, information on concomitant medications associated with those events, and any nonstudy vaccinations. In addition, study personnel will collect information on known participant exposure to someone with known COVID-19 or SARS-CoV-2 infection and on participant experience of COVID-19 symptoms.
15. All concomitant medications and nonstudy vaccinations will be recorded through 28 days after each injection; all concomitant medications relevant to or for the treatment of an SAE or MAAE will be recorded from Day 1 through the final visit (Day 394).

9.6. Appendix F Schedule of Assessments (Part 1B, Open-label Observational Phase for Subjects Who Previously Received Placebo)

**Visit Number**	7	8	9	10	11
**Type of Visit**	C	C	C	SC[8]	C
**Study Visit Day**	(~D209/ Participant Decision Visit) OL-D1	OL-D29	OL-D57[2]	OL-D113	OL-D178[9]
**Window Allowance (Days)**	-56/+56[1]	-3/+7	±14	± 3	±14
**Days Since Most Recent Injection**	0	28	28	84	149
Informed consent form	These assessments are already performed as part of the regular D209 Visit.				
Blood for vaccine immunogenicity			X		X
Nasopharyngeal or nasal swab sample for SARS-CoV-2[3]		X	X		
Physical examination including vital signs[4]	PE including vitals performed as part of D209 Visit.	X	X		X
	X Vitals obtained postdose.	X			
Pregnancy testing	X	X			
Study injection (including 30-minute postdosing observation period)	X	X			
Informed consent for BD			X[7]		
Recording of MAAEs and concomitant medications relevant to or for the treatment of the MAAE[5]	X	X	X	X	X
Recording of SAEs and concomitant medications relevant to or for the treatment of the SAE[5]	X	X	X	X	X

**Visit Number**	7	8	9	10	11
**Type of Visit**	C	C	C	SC[8]	C
**Study Visit Day**	(~D209/ Participant Decision Visit) OL-D1	OL-D29	OL-D57[2]	OL-D113	OL-D178[9]
**Window Allowance (Days)**	-56/+56[1]	-3/+7	±14	± 3	±14
**Days Since Most Recent Injection**	0	28	28	84	149
Recording of AESI	X	X	X	X	X
Recording of concomitant medications and nonstudy vaccinations[5]	X	X	X	X	X
Surveillance for COVID-19/ Illness visit[6]/ Unscheduled visit	X	X	X	X	X
Study completion (for Part 1B)					X

Abbreviations: AE = adverse event; AESI = adverse event of special interest; C = clinic visit; COVID-19 = coronavirus disease 2019; D = Day; M = month; MAAE = medically attended AE; NP = nasopharyngeal; OL = open-label; PCR = polymerase chain reaction; RT-PCR = reverse transcriptase polymerase chain reaction; SAE = serious adverse events; SARS CoV 2 = Severe Acute Respiratory Syndrome coronavirus 2; SC = safety (telephone) call.

1. The Participant Decision Visit may be performed over multiple visits. Once the Participant Decision Visit has been initiated, all assessments must be completed within a 7-day period.
2. After the OL-D57 visit, Part 1B participants who choose to receive BD will have one additional safety call at OL-D113 prior to entering Part 1C-1..
3. The NP or nasal swab sample, collected prior to vaccination on days of injection, will be used to ascertain the presence of SARS-CoV-2 via PCR.
4. Physical examination: A symptom-directed physical examination will be performed at OL-D1, OL-D29, OL-D57, and OL-D178. Symptom-directed physical examinations may be performed at other timepoints at the discretion of the investigator. Any clinically significant finding identified during a study visit should be reported as an MAAE. Vital signs are to be collected predosing and postdosing on days of injection (OL-D1 and OL-D29). When applicable, vital sign measurements should be performed before blood collection. Participants who are febrile (body temperature ≥ 38.0°C/100.4°F) before injection on OL-D1 or OL-D29 must be rescheduled within the relevant window period to receive the injection. Afebrile participants with minor illnesses can be administered investigational product at the discretion of the investigator.
5. All concomitant medications and nonstudy vaccinations will be recorded through 28 days postinjection; all concomitant medications relevant to or for the treatment of an SAE or MAAE will be recorded from Day 1 through OL D113 before entering Part 1C-1. If a participant declines a BD, all concomitant medications relevant to or for the treatment of an SAE or MAAE will be recorded from Day 1 through OL-D178.
6. If a participant meets the prespecified criteria of suspicion for COVID-19 (Section 7.1.6), the participants will be asked to return within 72 hours or as soon as possible to the study site for an unscheduled illness visit, to include an NP or nasal swab sample (for RT-PCR testing) and other clinical evaluations. If a study site visit is not possible, a home visit may be arranged to collect the NP or nasal sample and conduct clinical evaluations. If a home visit is not possible, the participant will be asked to submit a saliva sample to the study site by a Sponsor-approved method. At this illness visit, the NP or nasal swab samples will be collected to evaluate for the presence of SARS-CoV-2 infection. NP or nasal swab samples will also be tested for the presence of other respiratory pathogens. If due to COVID-19 related restrictions in the community, the participant's LAR performed a home test rather than obtaining a test performed by a health care professional, see

Section 7.1.6. In addition, the study site may collect an additional NP or nasal sample for SARS-CoV-2 testing to be able to render appropriate medical care for the study participant as determined by local standards of care. Additionally, clinical information will be carefully collected to evaluate the severity of the clinical case.

7. During OL-D57, all participants will be given the option to receive a BD. Participants will choose whether to receive the BD or not and sign the consent form. Those who decline BD will continue with Part 1B. Those who choose to receive a BD will have one safety call prior to entering Part 1C-1.
8. All participants in Part 1B who choose to receive BD will have one additional safety call at OL-D113 prior to entering Part 1C-1.
9. OL-D178 is only for those who decline BD. Participants who choose to receive a BD will follow Part 1C-1 SoA after the OL D113 safety call. The BD-D1 has a window of 1 month, thus, can be scheduled at least 5 months after the second dose, and participants will need to complete the OL-D113 safety call.

9.7. Appendix G Schedule of Assessments (Part 1C-1, Homologous Booster Dose Phase)

Visit Number	BD-1	BD-1a		BD-2	BD-3		BD-4
**Type of Visit**	C	C	SC[11]	C	C	SC	C
**Study Visit Day**	BD-D1[1] (D209: mRNA-1273; D394:placebo-mRNA-1273)	BD-D4	7, 14, and 21 days after BD-D1 (BD-D8, BD-D15, and BD-D22)	BD-D29	BD-D181 (Day 390: mRNA-1273; Day 571: placebo-mRNA-1273)	270 days after BD-D1 (BD-D271)	BD-D361 (Day 571 mRNA-1273; Day 751 placebo-mRNA-1273)
**Window Allowance (Days)**	-28 days	-2	+3	-3/+14	-3/+14	+3	-3/+14
**Days Since Most Recent Vaccination (in Part 1C-1)**	0	3	7, 14, 21	28	180	270	360
Confirm informed consent form signing	X						
Physical examination[2]	X			X	X		X
Pregnancy testing[3]	X						
**Immunogenicity Assessment**							
Blood for immunologic analysis[4]	X			X	X		X
**Biomarker Assessment**							
Blood sample for potential biomarker analysis[5]		X					
**Dosing**							
Study injection (including 30-minute postdosing observation period[6])	X						
**Efficacy Assessment**							
Surveillance for COVID-19/Unscheduled Visit[7]	X		X	X	X	X	X

**Visit Number**	BD-1	BD-1a		BD-2	BD-3		BD-4
**Type of Visit**	C	C	SC[11]	C	C	SC	C
**Study Visit Day**	BD-D1[1] (D209: mRNA-1273; D394:placebo-mRNA-1273)	BD-D4	7, 14, and 21 days after BD-D1 (BD-D8, BD-D15, and BD-D22)	BD-D29	BD-D181 (Day 390: mRNA-1273; Day 571: placebo-mRNA-1273)	270 days after BD-D1 (BD-D271)	BD-D361 (Day 571 mRNA-1273; Day 751 placebo-mRNA-1273)
**Window Allowance (Days)**	-28 days	-2	+3	-3/+14	-3/+14	+3	-3/+14
**Days Since Most Recent Vaccination (in Part 1C-1)**	0	3	7, 14, 21	28	180	270	360
Nasal swab[8]	X						
**Safety Assessments**							
Follow-up safety[9]			X			X	X
eDiary activation for recording solicited ARs (7 days)[10]	X						
Review of eDiary data[10]			X[10(BD-D8)]				
Recording of unsolicited AEs[11]	X	X	X	X			
Recording of MAAEs and concomitant medications relevant to or for the treatment of the MAAE[12]	X	X	X	X	X	X	X
Recording of AE leading to withdrawal	X	X	X	X	X	X	X
Recording of AESIs	X	X	X	X	X	X	X
Recording of SAEs and concomitant medications relevant to or for the treatment of the SAE[12]	X	X	X	X	X	X	X

Visit Number	BD-1	BD-1a		BD-2	BD-3		BD-4
**Type of Visit**	C	C	SC[11]	C	C	SC	C
**Study Visit Day**	BD-D1[1] (D209: mRNA-1273; D394:placebo-mRNA-1273)	BD-D4	7, 14, and 21 days after BD-D1 (BD-D8, BD-D15, and BD-D22)	BD-D29	BD-D181 (Day 390: mRNA-1273; Day 571: placebo-mRNA-1273)	270 days after BD-D1 (BD-D271)	BD-D361 (Day 571 mRNA-1273; Day 751 placebo-mRNA-1273)
**Window Allowance (Days)**	-28 days	-2	+3	-3/+14	-3/+14	+3	-3/+14
**Days Since Most Recent Vaccination (in Part 1C-1)**	0	3	7, 14, 21	28	180	270	360
Recording of concomitant medications and nonstudy vaccinations	X	X	X	X	X	X	X

Abbreviations: AE = adverse event; AESI = adverse event of special interest; AR = adverse reaction; BD = booster dose; C = clinic visit; D = day; eDiary = electronic diary; MAAE = medically attended AE; NP = nasopharyngeal; SAE = serious adverse event; SC = safety (phone) call.

Note: In accordance with FDA Guidance on Conduct of Clinical Trials of Medical Products during COVID-19 Public Health Emergency (DHHS 2020), investigators may convert study site visits to home visits or telemedicine visits with the approval of the Sponsor.

1. A BD may be administered to all participants who are currently enrolled in Part 1A or Part 1B provided there are no current contraindications for further dosing (Section 6). A participant who is currently in the Convalescent Period may come in for a BD-1 visit and receive a BD as long they are no longer symptomatic, and this includes the possibility for a Convalescent Visit Day 28 to overlap and be combined with a BD-1 visit.
2. Symptom-directed physical examination will be performed at the BD-Day 1. On dosing day before injection, the arm receiving the injection should be examined and the associated lymph nodes should be evaluated. At visits BD-2 (BD-D29), BD-3 (BD-D181) and BD-4 (BD-D361), a symptom-directed physical examination may be performed at the discretion of the investigator. Any clinically significant finding identified during a study visit should be reported as a MAAE. Vital signs are to be collected predosing and postdosing (participant will be seated for at least 5 minutes before all measurements are taken per Section 7.1.4) on the day of injection (BD-D1). Participants who are febrile (body temperature ≥ 38.0°C/100.4°F) before dosing (BD-D1) must be rescheduled to receive the injection. Afebrile participants with minor illnesses can be vaccinated at the discretion of the investigator.
3. The pregnancy test at the BD-1 visit will be a point-of-care urine test. At the discretion of the investigator, a pregnancy test either via blood or point-of-care urine test can be performed.
4. Sample should be collected prior to dosing on BD-D1.
5. All participants who chose to receive a BD. Serum sample from two ~4 mL blood draws. Biomarker plasma and biomarker serum samples will be stored for potential future biomarker assessment.

6. Postdosing, participants will have a 30-minute observation period.
7. An unscheduled visit may be prompted by reactogenicity issues, illness visit criteria for COVID-19, or new or ongoing AEs. If a participant meets the prespecified criteria of suspicion for COVID-19 (Section 7.1.6), the participants will be asked to return within 72 hours or as soon as possible to the study site for an unscheduled illness visit, to include an NP or nasal swab sample (for RT-PCR testing) and other clinical evaluations. If a study site visit is not possible, a home visit may be arranged to collect the nasal swab sample and conduct clinical evaluations. If a home visit is not possible, the participant will be asked to submit a saliva sample to the study site by a Sponsor-approved method. At this illness visit, the NP or nasal swab samples will be collected to evaluate for the presence of SARS-CoV-2 infection. Nasal swab samples will also be tested for the presence of other respiratory pathogens. If due to COVID-19 related restrictions in the community, the participant's LAR performed a home test rather than obtaining a test performed by a health care professional, see Section 7.1.6. In addition, the study site may collect an additional nasal swab sample for SARS-CoV-2 testing to be able to render appropriate medical care for the study participant as determined by local standards of care. Additionally, clinical information will be carefully collected to evaluate the severity of the clinical case.
8. The nasal swab sample must be collected prior to injection at the BD-1 visit.
9. Trained study personnel will call all participants to collect information relating to any unsolicited AEs, MAAEs (including any signs and symptoms of COVID-19), AESIs, AEs leading to withdrawal, SAEs, information on concomitant medications associated with those events, and any nonstudy vaccinations.
10. Diary entries will be recorded by the participant at approximately 30 minutes after injection while at the study site with instruction provided by study staff. Study participants will continue to record entries in the eDiary each day after they leave the study site, preferably in the evening, on the day of injection and for 6 days following injection. If a solicited local or systemic AR continues beyond Day 7 after vaccination, the participant will be prompted to capture details of the solicited local or systemic AR in the eDiary until the AR resolves; capture of details of ARs in the eDiary should not exceed 28 days after vaccination. ARs recorded in eDiaries beyond Day 7 should be reviewed either via telephone call or at the following study visit. Review of eDiary will occur on BD-D8.
11. Only for participants who chose to receive a BD.
12. All concomitant medications relevant to or for the treatment of an SAE or MAAE will be recorded from Screening through the final visit.

9.8. Appendix H Schedule of Assessments (Part 1C-2, Heterologous Booster Dose Phase)

Visit Number	BD-0	BD-1	BD-1a		BD-2	BD-3		BD-4
**Type of Visit**	C	C	C	SC[11]	C	C	SC	C
**Study Visit Day**	BD-D0[1]	BD-D1[1]	BD-D4	7, 14, and 21 days after BD-D1 (BD-D8, BD-D15, BD-D22)	BD-D29	BD-D181	270 days after BD-1 (BD-D271)	BD-D361
**Window Allowance (Days)**	-7	-	-2	+3	-3/+14	-3/+14	+3	-3/+14
**Days Since Most Recent Vaccination (in Part 1C-2)**	-	0	3	7, 14, 21	28	180	270	360
Informed consent/assent form, demographics, concomitant medications, medical history	X							
Physical examination including vital signs, height, weight [2]	X	X			X	X		X
Pregnancy testing[3]	X	X						
**Immunogenicity Assessment**								
Blood for immunologic analysis[4]		X			X	X		X
**Biomarker Assessment**								
Blood sample for potential biomarker analysis[5]			X					
**Dosing**								
Study injection (including 30-minute postdosing observation period[6])		X						
**Efficacy Assessment**								

**Visit Number**	BD-0	BD-1	BD-1a		BD-2	BD-3		BD-4
**Type of Visit**	C	C	C	SC[11]	C	C	SC	C
**Study Visit Day**	BD-D0[1]	BD-D1[1]	BD-D4	7, 14, and 21 days after BD-D1 (BD-D8, BD-D15, BD-D22)	BD-D29	BD-D181	270 days after BD-1 (BD-D271)	BD-D361
**Window Allowance (Days)**	-7	-	-2	+3	-3/+14	-3/+14	+3	-3/+14
**Days Since Most Recent Vaccination (in Part 1C-2)**	-	0	3	7, 14, 21	28	180	270	360
Surveillance for COVID-19/Unscheduled Visit[7]		X	X	X	X	X	X	X
Nasal swab[8]		X						
**Safety Assessments**								
Follow-up safety[9]				X			X	X
eDiary activation for recording solicited ARs (7 days)[10]		X						
Review of eDiary data[10]				X[10(BD-D8)]				
Recording of unsolicited AEs[11]		X	X	X	X			
Recording of MAAEs and concomitant medications relevant to or for the treatment of the MAAE[12]		X	X	X	X	X	X	X
Recording of AE leading to withdrawal		X	X	X	X	X	X	X
Recording of AESIs		X	X	X	X	X	X	X

**Visit Number**	BD-0	BD-1	BD-1a		BD-2	BD-3		BD-4
**Type of Visit**	C	C	C	SC[11]	C	C	SC	C
**Study Visit Day**	BD-D0[1]	BD-D1[1]	BD-D4	7, 14, and 21 days after BD-D1 (BD-D8, BD-D15, BD-D22)	BD-D29	BD-D181	270 days after BD-1 (BD-D271)	BD-D361
**Window Allowance (Days)**	-7	-	-2	+3	-3/+14	-3/+14	+3	-3/+14
**Days Since Most Recent Vaccination (in Part 1C-2)**	-	0	3	7, 14, 21	28	180	270	360
Recording of SAEs and concomitant medications relevant to or for the treatment of the SAE[12]		X	X	X	X	X	X	X
Recording of concomitant medications and nonstudy vaccinations		X	X	X	X	X	X	X

Abbreviations: AE = adverse event; AESI = adverse event of special interest; AR = adverse reaction; BD = booster dose; C = clinic visit; D = day; eDiary = electronic diary; EUA = Emergency Use Authorization; MAAE = medically attended AE; NP = nasopharyngeal; SAE = serious adverse event; SC = safety (phone) call.

Note: In accordance with FDA Guidance on Conduct of Clinical Trials of Medical Products during COVID-19 Public Health Emergency (DHHS 2020), investigators may convert study site visits to home visits or telemedicine visits with the approval of the Sponsor.

1. BD-Day 0 and BD-Day 1 may be combined on the same day. Additionally, the BD-Day 0 Visit may be performed over multiple visits if preformed within the 7-day Screening window. A BD will be administered to all eligible participants who are at least 3 months from completion of primary series under EUA (outside of the study).
2. A full physical examination including height, weight, and vital signs will be performed at the BD-Day 1. On dosing day before injection, the arm receiving the injection should be examined and the associated lymph nodes should be evaluated. At visits BD-2 (BD-D29), BD-3 (BD-D181) and BD-4 (BD-D361), a symptom-directed physical examination may be performed at the discretion of the investigator. Any clinically significant finding identified during a study visit should be reported as a MAAE. Vital signs are to be collected predosing and postdosing (participant will be seated for at least 5 minutes before all measurements are taken per Section 7.1.4 on the day of injection (BD-D1). Participants who are febrile (body temperature ≥ 38.0°C/100.4°F) before dosing (BD-D1) must be rescheduled to receive the injection. Afebrile participants with minor illnesses can be vaccinated at the discretion of the investigator.

3. The pregnancy test at the BD-1 visit will be a point-of-care urine test. At the discretion of the investigator, a pregnancy test either via blood or point-of-care urine test can be performed.
4. Sample should be collected prior to dosing on BD-D1.
5. Serum sample from two ~4 mL blood draws. Biomarker plasma and biomarker serum samples will be stored for potential future biomarker assessment.
6. Postdosing, participants will have a 30-minute observation period.
7. An unscheduled visit may be prompted by reactogenicity issues, illness visit criteria for COVID-19, or new or ongoing AEs. If a participant meets the prespecified criteria of suspicion for COVID-19 (Section 7.1.6), the participants will be asked to return within 72 hours or as soon as possible to the study site for an unscheduled illness visit, to include an NP or nasal swab sample (for RT-PCR testing) and other clinical evaluations. If a study site visit is not possible, a home visit may be arranged to collect the nasal swab sample and conduct clinical evaluations. If a home visit is not possible, the participant will be asked to submit a saliva sample to the study site by a Sponsor-approved method. At this illness visit, the NP or nasal swab samples will be collected to evaluate for the presence of SARS-CoV-2 infection. Nasal swab samples will also be tested for the presence of other respiratory pathogens. If due to COVID-19 related restrictions in the community, the participant's LAR performed a home test rather than obtaining a test performed by a health care professional, see Section 7.1.6. In addition, the study site may collect an additional nasal swab sample for SARS-CoV-2 testing to be able to render appropriate medical care for the study participant as determined by local standards of care. Additionally, clinical information will be carefully collected to evaluate the severity of the clinical case.
8. The nasal swab sample must be collected prior to injection at the BD-1 visit.
9. Trained study personnel will call all participants to collect information relating to any unsolicited AEs, MAAEs (including any signs and symptoms of COVID-19), AESIs, AEs leading to withdrawal, SAEs, information on concomitant medications associated with those events, and any nonstudy vaccinations.
10. Diary entries will be recorded by the participant at approximately 30 minutes after injection while at the study site with instruction provided by study staff. Study participants will continue to record entries in the eDiary each day after they leave the study site, preferably in the evening, on the day of injection and for 6 days following injection. If a solicited local or systemic AR continues beyond Day 7 after vaccination, the participant will be prompted to capture details of the solicited local or systemic AR in the eDiary until the AR resolves; capture of details of ARs in the eDiary should not exceed 28 days after vaccination. ARs recorded in eDiaries beyond Day 7 should be reviewed either via telephone call or at the following study visit. Review of eDiary will occur on BD-D8.
11. Only for participants who chose to receive a BD.
12. All concomitant medications relevant to or for the treatment of an SAE or MAAE will be recorded from Screening through the final visit.

9.9. Appendix I Schedule of Assessments (Part 2)

Visit Number	0	1	2	3	4	5	6	7	8	-		9	-		10
Type of Visit	C	C	Virtual Call	C	C	Virtual Call	C	Virtual Call	C	SFU		C	SFU		C
Month Timepoint		M0		M1			M2		M3	eDiary	SC	M7	eDiary	SC	M13
Study Visit Day	D0[1] (Screening)	D1 (Baseline)	D8[2]	D29[3]	D32	D36[2, 3]	D57[2, 3]	D64	D85	Every 4 weeks D99-183[3, 4]	Every 4 weeks D113-197[3, 5]	209 [3]	Every 4 weeks D223-307[3, 4]	Every 4 weeks D237-321[3, 5]	394[3]
Window Allowance (Days)	- 7		+ 3	+ 7	-2	+ 3	+ 7	+3	+7	±3	± 3	+7	± 3	± 3	± 14
Days Since Most Recent Injection	-	0	7	28	3	7	28	35	65	-	-	180	-	-	365
Informed consent/assent form, demographics, concomitant medications, medical history	X														
Review of inclusion and exclusion criteria[6]	X	X													
Physical examination including vital signs, height, weight[7]	X	X		X			X					X			X
Pregnancy test[8]	X	X		X											
Study injection (including 30-minute post dose observation period)		X		X											
Blood sample for potential biomarker analysis[9]					X										

Visit Number	0	1	2	3	4	5	6	7	8	-		9	-		10
Type of Visit	C	C	Virtual Call	C	C	Virtual Call	C	Virtual Call	C	SFU		C	SFU		C
Month Timepoint		M0		M1			M2		M3	eDiary	SC	M7	eDiary	SC	M13
Study Visit Day	D0[1] (Screening)	D1 (Baseline)	D8[2]	D29[3]	D32	D36[2, 3]	D57[2, 3]	D64	D85	Every 4 weeks D99-183[3, 4]	Every 4 weeks D113-197[3, 5]	209 [3]	Every 4 weeks D223-307[3, 4]	Every 4 weeks D237-321[3, 5]	394[3]
Window Allowance (Days)	- 7		+ 3	+ 7	-2	+ 3	+ 7	+3	+7	±3	± 3	+7	± 3	± 3	± 14
Days Since Most Recent Injection	-	0	7	28	3	7	28	35	65	-	-	180	-	-	365
Blood sample for vaccine immunogenicity[10]		X		X			X		X			X			X
Nasal swab sample for SARS-CoV-2[11]		X		X			X					X			
Surveillance for COVID-19/ Illness visit[12]/ Unscheduled visit		X	X	X	X	X	X	X	X	X	X	X	X	X	X
eDiary activation for recording solicited ARs (7 days)[13]		X		X											
Review of eDiary data[14]			X			X									
Follow-up safety telephone calls[15]											X			X	
Recording of unsolicited AEs		X	X	X	X	X	X								

**Visit Number**	0	1	2	3	4	5	6	7	8	-		9	-		10
**Type of Visit**	C	C	Virtual Call	C	C	Virtual Call	C	Virtual Call	C	SFU		C	SFU		C
**Month Timepoint**		M0		M1			M2		M3	eDiary	SC	M7	eDiary	SC	M13
**Study Visit Day**	D0[1] (Screening)	D1 (Baseline)	D8[2]	D29[3]	D32	D36[2, 3]	D57[2, 3]	D64	D85	Every 4 weeks D99-183[3, 4]	Every 4 weeks D113-197[3, 5]	209 [3]	Every 4 weeks D223-307[3, 4]	Every 4 weeks D237-321[3, 5]	394[3]
**Window Allowance (Days)**	- 7		+ 3	+ 7	-2	+ 3	+ 7	+3	+7	±3	± 3	+7	± 3	± 3	± 14
**Days Since Most Recent Injection**	-	0	7	28	3	7	28	35	65	-	-	180	-	-	365
Recording of MAAEs and concomitant medications relevant to or for the treatment of the MAAE[16]		X	X	X	X	X	X	X	X	X	X	X	X	X	X
Recording of SAEs and concomitant medications relevant to or for the treatment of the SAE[16]		X	X	X	X	X	X	X	X	X	X	X	X	X	X
Recording of AESI		X	X	X	X	X	X	X	X	X	X	X	X	X	X
Recording of AEs leading to withdrawal		X	X	X	X	X	X	X	X	X	X	X	X	X	X
Recording of concomitant medications and nonstudy vaccinations[15]		X	X	X	X	X	X	X	X	X	X	X	X	X	X
Study completion															X

Abbreviations: AE = adverse event; AESI = adverse event of special interest; AR = adverse reaction; C = clinic visit; COVID 19 = coronavirus disease 2019; D = day; eDiary = electronic diary; FDA = US Food and Drug Administration; IRB = institutional review board; M = month;

MAAE = medically attended AE; NP = nasopharyngeal; SC = safety (telephone) call; SFU = safety follow-up; RT-PCR = reverse transcriptase polymerase chain reaction; SAE = serious adverse event; SARS-CoV-2 = Severe Acute Respiratory Syndrome coronavirus 2.

Note: In accordance with FDA Guidance on Conduct of Clinical Trials of Medical Products during COVID-19 Public Health Emergency (DHHS 2020), investigators may convert study site visits to home visits or telemedicine visits with the approval of the Sponsor.

1. Day 0 and Day 1 may be combined on the same day. Additionally, the Day 0 Visit may be performed over multiple visits if they occur within the 7-day Screening window. If eligible, participants will receive 50 µg mRNA-1273 28 days apart.
2. All scheduled study visits should be completed within the respective visit windows. If the participant is not able to attend a study site visit due to the COVID 19 pandemic (self-quarantine or disruption of study site activities following business continuity plans and/or local government mandates for “stay at home” or “shelter in place”), a safety telephone call to the participant should be made in place of the study site visit. The safety telephone call should encompass all scheduled visit assessments that can be completed remotely, such as assessment for AEs and concomitant medications (eg, as defined in scheduled safety telephone calls). Home visits will be permitted for all nondosing visits, except for Screening, if a participant cannot visit the study due to the COVID-19 pandemic. Home visits must be permitted by the study site IRB and the participant via informed consent/assent and have prior approval from the Sponsor (or its designee).
3. If the visit for the second dose (Day 29) is disrupted and cannot be completed at Day 29 +7 due to the COVID-19 pandemic (self-quarantine or disruption of study site activities following business continuity plans and/or local government mandates for “stay at home” or “shelter in place”), the visit window may be extended to Day 29 + 21 days. When the extended visit window is used, the remaining study visits should be rescheduled to follow the inter-visit interval from the actual date of the second dose.
4. Safety follow-up via an eDiary questionnaire will be performed every 4 weeks from Day 99 to Day 183 and again from Day 223 to Day 307.
5. Safety follow-up via a safety telephone call will be performed every 4 weeks from Day 113 to Day 197 and again from Day 237 to Day 321.
6. Review of inclusion and exclusion criteria will be conducted during Screening. However, this review may be conducted on Day 1 if Screening and Baseline procedures are conducted on the same day.
7. Physical examination: A full physical examination, including height, weight, and vital signs will be performed at Screening, Day 1, Day 29, Day 57, Day 209, and Day 394. BMI will be calculated only at Screening Visit (Day 0). Symptom-directed physical examinations may be performed at other timepoints at the discretion of the investigator. On each injection day before injection and again 7 days after injection, the arm receiving the injection should be examined and the associated lymph nodes should be evaluated. Any clinically significant finding identified during a study visit should be reported as a MAAE. Vital signs are to be measured predose and postdose on days of injection (Day 1 and Day 29). When applicable, vital signs should be measured before blood collection. Participants who are febrile (body temperature ≥ 38.0°C/100.4°F) before injection on Day 1 and Day 29 must have the visit rescheduled within the relevant visit window to receive the injection. Afebrile participants with minor illnesses can be enrolled at the discretion of the investigator.
8. Pregnancy test at Screening and Day 1 and before the second study injection will be a point-of-care urine test. At the discretion of the investigator, a pregnancy test either via blood or point-of-care urine test can be performed at any time.
9. Serum sample from two ~4 mL blood draws. Biomarker plasma and biomarker serum samples will be stored for potential future biomarker assessment.
10. Sample must be collected prior to dosing of injection on Day 1 and Day 29.
11. The nasal swab sample will be used to ascertain the presence of SARS-CoV-2 via RT-PCR.
12. An unscheduled visit may be prompted by reactogenicity issues, illness visit criteria for COVID-19, or new or ongoing AEs. If a participant meets the prespecified criteria of suspicion for COVID-19 (Section 7.1.6), the participants will be asked to return within 72 hours or as soon as possible to the study site for an unscheduled illness visit, to include a nasal swab sample (for RT-PCR testing) and other clinical evaluations. If a study site visit

is not possible, a home visit may be arranged to collect the NP or nasal sample and conduct clinical evaluations. If a home visit is not possible, the participant will be asked to submit a saliva sample to the study site by a Sponsor-approved method. At this illness visit, the NP or nasal swab samples will be collected to evaluate for the presence of SARS-CoV-2 infection. NP or nasal swab samples will also be tested for the presence of other respiratory pathogens. In addition, the study site may collect an additional NP or nasal sample for SARS-CoV-2 testing to be able to render appropriate medical care for the study participant as determined by local standards of care. Additionally, clinical information will be carefully collected to evaluate the severity of the clinical case.

13. Diary entries will be recorded by the participant at approximately 30 minutes after injection while at the study site with instruction provided by study staff. Study participants will continue to record entries in the eDiary each day after they leave the study site, preferably in the evening, on the day of injection and for 6 days following injection. If a solicited local or systemic AR continues beyond Day 7 after vaccination, the participant will be prompted to capture details of the solicited local or systemic AR in the eDiary until the AR resolves or the next IP injection occurs, whichever occurs first; capture of details of ARs in the eDiary should not exceed 28 days after each vaccination.

14. ARs recorded in eDiaries beyond Day 7 after vaccination should be reviewed either via telephone call or at the following study visit.

15. Trained study site personnel will call all participants to collect information relating to any AEs, MAAEs, SAEs, AEs leading to study withdrawal, information on concomitant medications associated with those events, and any nonstudy vaccinations. In addition, study personnel will collect information on known participant exposure to someone with known COVID-19 or SARS-CoV-2 infection and on participant experience of COVID-19 symptoms.

16. All concomitant medications and nonstudy vaccinations will be recorded through 28 days after each injection; all concomitant medications relevant to or for the treatment of an SAE or MAAE will be recorded from Day 1 through the final visit (Day 394).

9.10. Appendix J Schedule of Assessments (Part 3)

Visit Number	0	1	2	3	4	5	6	-	7	8	9	10	-	11
Type of Visit	C	C	C	Virtual Call	C	C	C	SFU	C	C	Virtual Call	C	SFU	C
Month Timepoint		M0			M1	M2	M3	SC	M6			M7	SC	M12
Study Visit Day	D0[1] (Screening)	D1 (Baseline)	D3	D8[2]	D29	D57	D85	Every 6 weeks D99-141[3]	D181[4]	D184	D188	D209	Every 6 weeks D223-D321[3]	D361
Window Allowance (Days)	- 7		-2	+ 3	+ 7	+3	+7	±3	+ 7	-2	+ 3	+ 7	±3	±14
Days Since Most Recent Injection	-	0	3	7	28	56	84	-	180 /0	3	7	28	-	180
Informed consent/assent form, demographics, concomitant medications, medical history	X													
Review of inclusion and exclusion criteria[5]	X	X												
Physical examination including vital signs, height, weight[6]	X	X			X				X			X		X
Pregnancy test[7]	X	X							X					
Study injection (including 30-minute post dose observation period)		X							X					
Blood sample for potential biomarker analysis[8]			X							X				
Blood sample for vaccine immunogenicity[9]		X			X		X		X			X		X
Nasal swab sample for SARS-CoV-2[10]		X			X		X		X			X		
Surveillance for COVID-19/ Illness visit[11]/ Unscheduled visit		X	X	X	X	X	X	X	X	X	X	X	X	X
eDiary activation for recording solicited ARs (7 days)[12]		X							X					
Review of eDiary data[13]				X							X			

Visit Number	0	1	2	3	4	5	6	-	7	8	9	10	-	11
**Type of Visit**	C	C	C	Virtual Call	C	C	C	SFU	C	C	Virtual Call	C	SFU	C
**Month Timepoint**		M0			M1	M2	M3	SC	M6			M7	SC	M12
**Study Visit Day**	D0[1] (Screening)	D1 (Baseline)	D3	D8[2]	D29	D57	D85	Every 6 weeks D99-141[3]	D181[4]	D184	D188	D209	Every 6 weeks D223-D321[3]	D361
**Window Allowance (Days)**	- 7		-2	+ 3	+ 7	+3	+7	±3	+ 7	-2	+ 3	+ 7	±3	±14
**Days Since Most Recent Injection**	-	0	3	7	28	56	84	-	180 /0	3	7	28	-	180
Follow-up safety telephone calls[14]								X					X	
Recording of unsolicited AEs		X	X	X	X				X	X	X	X		
Recording of MAAEs and concomitant medications relevant to or for the treatment of the MAAE[14]		X	X	X	X	X	X	X	X	X	X	X	X	X
Recording of SAEs and concomitant medications relevant to or for the treatment of the SAE[14]		X	X	X	X	X	X	X	X	X	X	X	X	X
Recording of AESI		X	X	X	X	X	X	X	X	X	X	X	X	X
Recording of AEs leading to withdrawal		X	X	X	X	X	X	X	X	X	X	X	X	X
Recording of concomitant medications and nonstudy vaccinations[15]		X	X	X	X	X	X	X	X	X	X	X	X	X
Study completion														X

Abbreviations: AE = adverse event; AESI = adverse event of special interest; AR = adverse reaction; BMI = body mass index; C = clinic visit; COVID 19 = coronavirus disease 2019; D = day; eDiary = electronic diary; FDA = US Food and Drug Administration; IRB = institutional review board; M = month; MAAE = medically attended AE; NP = nasopharyngeal; SC = safety (telephone) call; SFU = safety follow-up; RT-PCR = reverse transcriptase polymerase chain reaction; SAE = serious adverse event; SARS-CoV-2 = Severe Acute Respiratory Syndrome coronavirus 2.

Note: In accordance with FDA Guidance on Conduct of Clinical Trials of Medical Products during COVID-19 Public Health Emergency (DHHS 2020), investigators may convert study site visits to home visits or telemedicine visits with the approval of the Sponsor.

1. Day 0 and Day 1 may be combined on the same day. Additionally, the Day 0 Visit may be performed over multiple visits if they occur within the 7-day Screening window.
2. All scheduled study visits should be completed within the respective visit windows. If the participant is not able to attend a study site visit due to the COVID 19 pandemic (self-quarantine or disruption of study site activities following business continuity plans and/or local government mandates for “stay at home” or “shelter in place”), a safety telephone call to the participant should be made in place of the study site visit. The safety telephone call should encompass all scheduled visit assessments that can be completed remotely, such as assessment for AEs and concomitant medications (eg, as defined in scheduled safety telephone calls). Home visits will be permitted for all nondosing visits, except for Screening, if a participant cannot visit the study site due to the COVID-19 pandemic. Home visits must be permitted by the study site IRB and the participant via informed consent/assent and have prior approval from the Sponsor (or its designee).
3. Safety follow-up via a safety telephone call will be performed every 6 weeks from Day 99 to Day 141 and again from Day 223 to Day 321.
4. If the visit for the second dose (Day 181) is disrupted and cannot be completed at Day 181 +7 due to the COVID-19 pandemic (self-quarantine or disruption of study site activities following business continuity plans and/or local government mandates for “stay at home” or “shelter in place”), the visit window may be extended to Day 181 +21 days. When the extended visit window is used, the remaining study visits should be rescheduled to follow the inter-visit interval from the actual date of the second dose.
5. Review of inclusion and exclusion criteria will be conducted during Screening. However, this review will be conducted on Day 1 if Screening and Baseline procedures are conducted on the same day.
6. Physical examination: A full physical examination, including height, weight, and vital signs will be performed at Screening, Day 1, Day 29, Day 181, Day 209, and Day 361. BMI will be calculated only at Screening Visit (Day 0). Symptom-directed physical examinations may be performed at other timepoints at the discretion of the investigator. On each injection day before injection and again 7 days after injection, the arm receiving the injection should be examined and the associated lymph nodes should be evaluated. Any clinically significant finding identified during a study visit should be reported as a MAAE. Vital signs are to be measured predose and postdose on days of injection (Day 1 and Day 181). When applicable, vital signs should be measured before blood collection. Participants who are febrile (body temperature ≥ 38.0°C/100.4°F) before injection on Day 1 and Day 181 must have the visit rescheduled within the relevant visit window to receive the injection. Afebrile participants with minor illnesses can be enrolled at the discretion of the investigator.
7. Pregnancy test at Screening and Day 1 and before the second study injection (Day 181) will be a point-of-care urine test. At the discretion of the investigator, a pregnancy test either via blood or point-of-care urine test can be performed at any time.
8. Serum and plasma samples from two ~4 mL blood draws. Biomarker plasma and biomarker serum samples will be stored for potential future biomarker assessment.
9. Sample must be collected prior to dosing of injection on Day 1 and Day 181.
10. The nasal swab sample will be used to ascertain the presence of SARS-CoV-2 via RT-PCR.
11. An unscheduled visit may be prompted by reactogenicity issues, illness visit criteria for COVID-19, or new or ongoing AEs. If a participant meets the prespecified criteria of suspicion for COVID-19 (Section 7.1.6), the participants will be asked to return within 72 hours or as soon as possible to the study site for an unscheduled illness visit, to include a nasal swab sample (for RT-PCR testing) and other clinical evaluations. If a study site visit is not possible, a home visit may be arranged to collect the NP or nasal sample and conduct clinical evaluations. If a home visit is not possible, the participant will be asked to submit a saliva sample to the study site by a Sponsor-approved method. At this illness visit, the NP or nasal swab samples will be collected to evaluate for the presence of SARS-CoV-2 infection. NP or nasal swab samples will also be tested for the

presence of other respiratory pathogens. In addition, the study site may collect an additional NP or nasal sample for SARS-CoV-2 testing to be able to render appropriate medical care for the study participant as determined by local standards of care. Additionally, clinical information will be carefully collected to evaluate the severity of the clinical case.

12. Diary entries will be recorded by the participant at approximately 30 minutes after injection while at the study site with instruction provided by study staff. Study participants will continue to record entries in the eDiary each day after they leave the study site, preferably in the evening, on the day of injection and for 6 days following injection. If a solicited local or systemic AR continues beyond Day 7 after vaccination, the participant will be prompted to capture details of the solicited local or systemic AR in the eDiary until the AR resolves or the next IP injection occurs, whichever occurs first; capture of details of ARs in the eDiary should not exceed 28 days after each vaccination.

13. ARs recorded in eDiaries beyond Day 7 after vaccination should be reviewed either via telephone call or at the following study visit.

14. Trained study site personnel will call all participants to collect information relating to any AEs, MAAEs, SAEs, AEs leading to study withdrawal, information on concomitant medications associated with those events, and any nonstudy vaccinations. In addition, study personnel will collect information on known participant exposure to someone with known COVID-19 or SARS-CoV-2 infection and on participant experience of COVID-19 symptoms.

15. All concomitant medications and nonstudy vaccinations will be recorded through 28 days after each injection; all concomitant medications relevant to or for the treatment of an SAE or MAAE will be recorded from Day 1 through the final visit (Day 361).
