# Supplementary material for: Safety and durability of mRNA-1273–induced SARS-CoV-2 immune responses in adolescents: results from the phase 2/3 TeenCOVE trial
Source: eClinicalMedicine. 2024 Jul 18;74:102720. doi: 10.1016/j.eclinm.2024.102720 (PMC11293523; doi:10.1016/j.eclinm.2024.102720)
Supplement: Moderna mRNA-1273-P203 SAP v5.0_Part 1B+1C+2+3 [file mmc4.pdf]

ModernaTX, Inc.

Protocol mRNA-1273-P203

A Phase 2/3, Randomized, Observer-Blind, Placebo-Controlled Study to Evaluate the Safety, Reactogenicity, and Effectiveness of mRNA-1273 SARS-CoV-2 Vaccine in Healthy Adolescents 12 to < 18 Years of Age

Statistical Analysis Plan
(Part 1B, Part 1C, Part 2 and Part 3)

SAP Version 5.0
Version Date of SAP: 30 Jun 2023

Prepared by:

PPD
3575 Quakerbridge Road
Suite 201
Hamilton, NJ 08619

Confidential

TABLE OF CONTENTS

SUMMARY OF MAJOR CHANGES IN SAP VERSION V

LIST OF ABBREVIATIONS VII

1. INTRODUCTION 8

2. STUDY OBJECTIVES 8

2.1. PART 1B, OPEN LABEL 8
2.1.1. Exploratory Objectives 8
2.2. PART 1C-1, HOMOLOGOUS BOOSTER DOSE 9
2.2.1. Primary Safety Objective 9
2.2.2. Primary Immunogenicity Objective 9
2.2.3. Key Secondary Objective 9
2.2.4. Exploratory Objectives 9
2.3. PART 1C-2, HETEROLOGOUS BOOSTER DOSE 10
2.3.1. Primary Safety Objective 10
2.3.2. Primary Immunogenicity Objective 10
2.3.3. Key Secondary Objective 10
2.3.4. Exploratory Objectives 10
2.4. PART 2 10
2.4.1. Primary Safety Objective 10
2.4.2. Primary Immunogenicity Objective 11
2.4.3. Exploratory Objectives 11
2.5. PART 3 11
2.5.1. Primary Safety Objective 11
2.5.2. Primary Immunogenicity Objective 11
2.5.3. Secondary Objectives 11
2.5.4. Exploratory Objectives 12

3. STUDY ENDPOINTS 12

3.1. PART 1B, OPEN LABEL 12
3.1.1. Exploratory Endpoints 12
3.2. PART 1C-1, HOMOLOGOUS BOOSTER DOSE 13
3.2.1. Primary Safety Endpoints 13
3.2.2. Primary Immunogenicity Endpoints 13
3.2.3. Key Secondary Endpoints 14
3.2.4. Exploratory Endpoints 14
3.3. PART 1C-2, HETEROLOGOUS BOOSTER DOSE 14
3.3.1. Primary Safety Endpoints 14
3.3.2. Primary Immunogenicity Endpoints 15
3.3.3. Key Secondary Endpoints 15
3.3.4. Exploratory Endpoints 15
3.4. PART 2 15
3.4.1. Primary Safety Endpoints 15
3.4.2. Primary Immunogenicity Endpoints 16
3.4.3. Exploratory Endpoints 16

3.5. Part 3 17
3.5.1. Primary Safety Endpoints 17
3.5.2. Primary Immunogenicity Endpoints 17
3.5.3. Secondary Endpoints 18
3.5.4. Exploratory Endpoints 20

4. STUDY DESIGN 20

4.1. Overall Study Design 20
4.2. Statistical Hypothesis 20
4.2.1. Part 1C-1, Homologous Booster Dose 20
4.2.1.1. Primary Immunogenicity Endpoint 20
4.2.1.2. Key Secondary Immunogenicity Endpoint 21
4.2.2. Part 2 23
4.2.3. Part 3 23
4.3. Sample Size and Power 24
4.3.1. Part 1C-1, Homologous Booster Dose 24
4.3.2. Part 1C-2, Heterologous Booster Dose 25
4.3.3. Part 2 25
4.3.4. Part 3 26
4.4. Multiplicity Adjustment 27
4.5. Blinding and Unblinding 27

5. ANALYSIS POPULATIONS 27

5.1. Full Analysis Set 27
5.2. Immunogenicity Subset 28
5.3. Per-protocol (PP) Immunogenicity Subset 29
5.4. Modified Intent-to-Treat-1 (mITT1) Set 32
5.5. Solicited Safety Set 32
5.6. Safety Set 33

6. STATISTICAL ANALYSIS 33

6.1. General Considerations 33
6.2. Background Characteristics 38
6.2.1. Subject Disposition 38
6.2.2. Demographics 41
6.2.3. Medical History 41
6.2.4. Prior and Concomitant Medications 41
6.2.5. Study Exposure 42
6.2.6. Major Protocol Deviations 42
6.3. Safety Analysis 43
6.3.1. Adverse Events 43
6.3.1.1. Incidence of Adverse Events 44
6.3.1.2. TEAEs by System Organ Class and Preferred Term 44
6.3.2. Solicited Adverse Reactions 45
6.3.3. Pregnancy Tests 46
6.3.4. Vital Sign Measurements 47
6.4. Immunogenicity Analysis 47

6.4.1. Sampling of the Immunogenicity Subset 49
6.4.2. Immunogenicity Assessments 49
6.4.3. Primary Analysis of Antibody-Mediated Immunogenicity Endpoints 50
6.4.4. Key Secondary Analysis of Antibody-Mediated Immunogenicity Endpoints 53
6.4.5. Secondary Analysis of Antibody-Mediated Immunogenicity Endpoints 53
6.4.6. Exploratory Analysis of Antibody-Mediated Immunogenicity Endpoints 55
6.5. Efficacy Analysis 58
6.5.1. Endpoint Definition/Derivation 59
6.5.1.1. Derivation of SARS-CoV-2 Infection 59
6.5.1.2. Derivation of Asymptomatic SARS-CoV-2 Infection 60
6.5.1.3. Derivation of COVID-19 60
6.5.1.4. Derivation of Secondary Case (CDC Case) Definition of COVID-19 62
6.5.2. Analysis Method 62
6.5.3. Sensitivity Analysis 63
6.6. Exploratory Analysis 63
6.6.1. SARS-CoV-2 Exposure and Symptoms 63
6.6.2. Exploratory Analysis of Efficacy Endpoints 63
6.7. Interim Analysis 63
6.8. Final Analysis 64
7. REFERENCES 64
8. LIST OF APPENDICES 66
8.1. Appendix A Standards for Safety and Immunogenicity Variable Display in TFLs 66
8.2. Appendix B Analysis Visit Windows for Safety and Immunogenicity Analysis 66
8.3. Appendix C Imputation Rules for Missing Prior/Concomitant Medications and Non-Study Vaccinations 69
8.4. Appendix D Imputation Rules for Missing AE dates 71

Summary of Major Changes in SAP Version

SAP Version	Section # and Name	Description of Change
V3.0 Based on Protocol Amendment 3		Refer to SAP for Part A
V4.0	Section 5.2	Add Clarification for Immune subset selection;<br>Add Immunogenicity Subset (Long-term Analysis at Day 1, Day 57, and Day 209)
V4.0	Section 5.3	Add Per-protocol (PP) Immunogenicity Subset (Long-term Analysis at Day 1, Day 57 and Day 209)
V4.0	Section 6.2.1	Add Immune subset and PP immune subset for long-term analysis in disposition
V4.0	Section 6.2.2	Add Immune subset and PP immune subset for long-term analysis in demographics
V4.0	Section 6.4.5	1. added clarification for antibody against prototype or variants of interest;<br>2. added paried comparison bwteen visits in P203;<br>3. added ANCOVA moel;<br>4. Added clarification of approach used for comparison of P203 with P301 at BD-Day 29
V4.0	Section 7	Add reference for Adjusted Wald confidence interval
V5.0 Based on Protocol Amendment 4, 5 and 6	Section 2.3, 2.4, 2.5, 3.3, 3.4, 3.5, 4.2.2, 4.2.3, 4.3.3, 4.3.4	Newly added for the new study part Part 1C-2, Part 2 and Part 3 added in Protocol Amendment 4 and Protocol Amendment 5;<br>Revised minorly per Protocol Amendment 6
V5.0 Based on Protocol Amendment 5 and 6	Section 4.4	Newly added based on multiplicity in section 4.2.1.3 and add multiplicity adjustment of new study Part 3
V5.0 Based on Protocol Amendment 5 and 6	Section 5	Updated to reflect the analysis population for new

		studay part Part 1C-2, Part 2 and Part 3 added in Protocol Amendment 4 and Protocol Amendment 5;<br>Revised minorly per Protocol Amendment 6
V5.0	Section 5.3 and 5.4	Moved Per-protocol (PP) Immunogenicity Subset - Pre-booster SARS-CoV-2 Negative (Part C, Booster Dose) from Section 5.4 in SAP v4.0 to Section 5.3
V5.0 Based on Protocol Amendment 4, 5 and 6	Section 6	Updated to reflect the analysis for new study part Part 1C-2, Part 2 and Part 3 added in Protocol Amendment 4 and Protocol Amendment 5;<br>Revised minorly per Protocol Amendment 6;
V5.0 Based on Protocol Amendment 4, 5 and 6	Section 8.2	Updated to add analysis visit window of new study part Part 1C-2, Part 2 and Part 3 added in Protocol Amendment 4 and Protocol Amendment 5;<br>No update in Protocol Amendment 6.

List of Abbreviations

Abbreviation	Definition
AE	adverse event
AR	adverse reaction
BD	booster dose
BMI	body mass index
bAb	binding antibody
CDC	Centers for Disease Control and Prevention
CI	confidence interval
CRO	contract research organization
CSP	clinical study protocol
CSR	clinical study report
DHHS	Department of Health and Human Services
eCRF	electronic case report form
eDiary	electronic diary
ELISA	enzyme-linked immunosorbent assay
EUA	Emergency Use Authorization
FAS	full analysis set
GM	geometric mean
GMFR	geometric mean fold rise
GMT	geometric mean titer
GMR	geometric mean ratio
IgG	immunoglobulin G
IP	investigational product
IRT	interactive response technology
LLOQ	lower limit of quantification
MAAEs	medically-attended adverse events
MedDRA	Medical Dictionary for Regulatory Activities
mRNA	messenger ribonucleic acid
nAb	neutralizing antibody
OL	open-label
PP	per-protocol
PT	preferred term
SAE	serious adverse event
SAP	statistical analysis plan
SAS	Statistical Analysis System
SD	standard deviation
SOC	system organ class
SRR	seroresponse rate
TEAE	treatment-emergent adverse event
ULOQ	upper limit of quantification
VOC	variant of concern
WHO	World Health Organization
WHODD	World Health Organization drug dictionary

1. Introduction

This statistical analysis plan (SAP), which describes the planned analyses for Part 1B and Part 1C, Part 2 and Part 3 of Study mRNA-1273-P203, is based on the approved clinical study protocol (CSP), Version Amendment 6, dated 22-Jun-2023. The most recent approved electronic case report form (eCRF) Version 16, dated 28-FEB-2023. Unless specified otherwise, the language in this SAP pertains to Part 1B, Part 1C, Part 2 and Part 3. SAP for Part 1A is in a separate document.

In addition to the information presented in the statistical analysis plan section of the protocol (Section 8) which provides the principal features of analyses for this study, this SAP provides statistical analysis details/data derivations. It also documents modifications or additions to the analysis plan that are not "principal" in nature and result from information that was not available at the time of protocol finalization.

Study mRNA-1273-P203 is a Phase 2/3, randomized, observer-blind, placebo-controlled study to evaluate the safety, reactogenicity, and effectiveness of messenger ribonucleic acid (mRNA)-1273 SARS-CoV-2 vaccine in healthy adolescents 12 to <18 years of age.

PPD Biostatistics and programming team, designee of Moderna Biostatistics and Programming department, will perform the statistical analysis of the safety, reactogenicity, and effectiveness data; Statistical Analysis System (SAS) Version 9.4 or higher will be used to generate all statistical outputs (tables, figures, listings, and datasets). The SAP will be finalized and approved prior to the primary analysis clinical database lock and treatment unblinding for the study. If the methods in this SAP differ from the methods described in the protocol, the SAP will prevail.

In this document, subject and participant are used interchangeably; injection of IP, injection, and dose are used interchangeably; vaccination group and treatment group are used interchangeably.

2. Study Objectives

2.1. Part 1B, Open Label

2.1.1. Exploratory Objectives

The exploratory objectives are the following:

- To evaluate the safety of 2 doses of mRNA-1273, including the long-term follow-up safety for the mRNA-1273 cohort and the safety after cross-over for the placebo-mRNA-1273 cohort
- To evaluate the incidence of SARS-CoV-2 infection or COVID-19 after vaccination with mRNA-1273, including the incidence for the long-term follow-up period in the mRNA-1273 cohort and the incidence after cross-over for the placebo-mRNA-1273 cohort

2.2. Part 1C-1, Homologous Booster Dose

2.2.1. Primary Safety Objective

The primary safety objective is to evaluate the safety of the 50 µg booster dose (BD) of mRNA-1273.

2.2.2. Primary Immunogenicity Objective

The primary immunogenicity objective is to infer effectiveness of 50 µg of mRNA-1273 booster by establishing noninferiority of Ab response after the BD compared to the primary series of mRNA-1273. GM values of serum Ab and SRR of postbooster in Study P203 compared with primary series from young adult (18 to 25 years of age) recipients of mRNA-1273 in the clinical endpoint efficacy trial (Study P301).

2.2.3. Key Secondary Objective

The key secondary objective is to evaluate immune response elicited by the 50 µg prototype booster of mRNA-1273 against variant(s) of interest.

2.2.4. Exploratory Objectives

The exploratory objectives are the following:

- To evaluate the persistence of the immune response of the BD of mRNA-1273 vaccine (50 µg) as assessed by the level of SARS-CoV-2 S2P specific bAb through 1 year after BD
- To evaluate the persistence of the immune response of the BD of mRNA-1273 vaccine (50 µg) as assessed by the level of nAb through 1 year after BD
- To evaluate the incidence of SARS-CoV-2 infection or COVID-19 after vaccination with mRNA-1273

2.3. Part 1C-2, Heterologous Booster Dose

2.3.1. Primary Safety Objective

The primary safety objective is to evaluate the safety of the 50 µg BD of mRNA-1273 in participants who received non Moderna COVID-19 primary series vaccination.

2.3.2. Primary Immunogenicity Objective

The primary immunogenicity objective is to to evaluate immune response elicited by the 50 µg booster of mRNA-1273 in participants who received non Moderna COVID-19 primary series vaccination.

2.3.3. Key Secondary Objective

The key secondary objective to evaluate immune response elicited by the 50 µg booster of mRNA-1273 against variant(s) of interest in participants who received non Moderna COVID-19 primary series vaccination.

2.3.4. Exploratory Objectives

The exploratory objectives are the following:

- To evaluate the persistence of the immune response of the BD of mRNA-1273 vaccine (50 µg) as assessed by the level of SARS-CoV-2 S2P specific bAb through 1 year after BD in participants who received non Moderna COVID-19 primary series vaccination
- To evaluate the persistence of the immune response of the BD of mRNA-1273 vaccine (50 µg) as assessed by the level of nAb through 1 year after BD in participants who received non Moderna COVID-19 primary series vaccination
- To evaluate the incidence of SARS-CoV-2 infection or COVID-19 after booster vaccination with mRNA-1273 in participants who received non Moderna COVID-19 primary series vaccination

2.4. Part 2

2.4.1. Primary Safety Objective

The primary safety objective is to evaluate the safety and reactogenicity of the 50 µg mRNA-1273 vaccine administered in 2 doses 28 days apart.

2.4.2. Primary Immunogenicity Objective

The primary immunogenicity objective is to evaluate immune response elicited by the 50 µg mRNA-1273 vaccine administered in 2 doses 28 days apart.

2.4.3. Exploratory Objectives

The exploratory objectives are the following:

- To evaluate the persistence of the immune response of 50 µg mRNA-1273 vaccine administered in 2 doses 28 days apart as assessed by the level of SARS-CoV-2 S2P specific bAb through 1 year after Dose 2
- To evaluate the persistence of the immune response of the 50 µg mRNA-1273 vaccine as assessed by the level of nAb through 1 year after Dose 2
- To evaluate the incidence of SARS-CoV-2 infection or COVID-19 after vaccination with mRNA-1273

2.5. Part 3

2.5.1. Primary Safety Objective

The primary safety objective is to evaluate the safety and reactogenicity of the 50 µg mRNA-1273.222 vaccine administered in 2 doses 6 months apart.

2.5.2. Primary Immunogenicity Objective

The primary immunogenicity objective is to infer effectiveness of the 50 µg mRNA-1273.222 vaccine based on immune response against SARS-CoV-2 VOC (Omicron BA.4/BA.5) and ancestral strain obtained 28 days post Dose 1 in the baseline SARS-CoV-2 positive population.

2.5.3. Secondary Objectives

The secondary objectives are the following:

- To evaluate immune response elicited by 50 µg mRNA-1273.222 vaccine administered in 2 doses 6 months apart based on immune responses against Omicron BA.4/BA.5 and ancestral strain obtained 28 days post Dose 1 or 28 days post Dose 2.

- To evaluate immune response elicited by 50 µg mRNA-1273.222 vaccine administered in 2 doses 6 months apart based on immune responses against other variant(s) of interest obtained 28 days post Dose 1 or 28 days post Dose 2.

2.5.4. Exploratory Objectives

The exploratory objectives are the following:

- To evaluate the persistence of the immune response of 50 µg mRNA-1273.222 vaccine, as assessed by the level of SARS-CoV-2 S2P specific bAb through 6 months after Dose 2.
- To evaluate the persistence of the immune response of 50 µg mRNA-1273.222 vaccine, as assessed by the level of nAb through 6 months after Dose 2.
- To evaluate the incidence of COVID-19 after vaccination with mRNA-1273.222.

3. Study Endpoints

3.1. Part 1B, Open Label

3.1.1. Exploratory Endpoints

The exploratory endpoints are the following:

- MAAEs through the last day of study participation or the booster dose if applicable
- SAEs through the last day of study participation or the booster dose if applicable
- AESIs through the last day of study participation or the booster dose if applicable
- AEs leading to discontinuation from study participation through the last day of study participation or the booster dose if applicable
- The incidence of SARS-CoV-2 infection (symptomatic or asymptomatic infection) counted starting 14 days after the second dose and after the first dose of of mRNA-1273, including Part 1A and Part 1B for mRNA-1273 cohort, and Part 1B for placebo-mRNA-1273 cross-over cohort
- To evaluate the incidence of asymptomatic SARS-CoV-2 infection after vaccination with mRNA-1273 measured by RT-PCR and/or bAb levels against SARS-CoV-2 nucleocapsid protein (by Roche Elecsys) counted starting 14 days after the second dose and after the first dose of mRNA-1273 in participants with

negative SARS-CoV-2 at baseline, including Part 1A and Part 1B for mRNA-1273 cohort, and Part 1B for placebo-mRNA-1273 cross-over cohort

- The incidence of the first occurrence of symptomatic COVID-19 starting 14 days after the second dose and after the first dose of of mRNA-1273, including Part 1A and Part 1B for mRNA-1273 cohort, and Part 1B for placebo-mRNA-1273 cross-over cohort

3.2. Part 1C-1, Homologous Booster Dose

3.2.1. Primary Safety Endpoints

The primary safety objective will be evaluated by the following safety endpoints:

- Solicited local and systemic ARs through 7 days after BD
- Unsolicited AEs through 28 days after BD injection
- MAAEs post BD through the last day of study participation
- SAEs post BD through the last day of study participation
- AESIs post BD through the last day of study participation AEs leading to discontinuation from study participation post BD through the last day of study participation

3.2.2. Primary Immunogenicity Endpoints

- Co-Primary endpoint(s):
    - GM value of postbooster (post Dose 3) Ab against ancestral strain in Study P203 as compared to post primary series (post Dose 2) against ancestral strain in the young adults in Study P301
    - Seroresponse rate of postbooster/Dose 3 from baseline (pre Dose 1) as compared to post Dose 2 from baseline (pre Dose 1) against ancestral strain in the young adults in Study P301, using 4-fold rise definition
        - Seroresponse is defined as Ab value change from baseline (pre-Dose 1) below the LLOQ to ≥ 4 × LLOQ, or at least a 4 fold rise if baseline is ≥ LLOQ

3.2.3. Key Secondary Endpoints

The key secondary objective will be evaluated by the following endpoints:

- GM value of postbooster (post Dose 3) Ab against circulating strain as compared to post primary series (post Dose 2) against circulating strain in the young adults in Study P301
- SRR of postbooster/Dose 3 from baseline (pre Dose 1) as compared to post Dose 2 from baseline (pre Dose 1) against circulating strain using 4-foldrise definition in the young adults in Study P301

3.2.4. Exploratory Endpoints

The exploratory endpoints are the following:

- The GM values of SARS-CoV-2 S2P specific bAb on BD-Day 1, BD-Day 29 (1 month after BD), BD-Day 181 (6 months after BD), and BD-Day 361 (1 year after BD)
- The GM values of SARS-CoV-2-specific nAb on BD-Day 1, BD-Day 29 (1 month after BD), BD-Day 181 (6 months after BD), and BD Day 361 (1 year after BD)
- The incidence of SARS-CoV-2 infection (symptomatic or asymptomatic infection) counted starting 14 days after BD of mRNA-1273
- To evaluate the incidence of asymptomatic SARS-CoV-2 infection after vaccination with mRNA-1273 measured by RT-PCR and/or bAb levels against SARS-CoV-2 nucleocapsid protein (by Roche Elecsys) counted starting 14 days after BD in participants with negative SARS-CoV-2 at baseline or prebooster
- The incidence of the first occurrence of symptomatic COVID-19 starting 14 days after BD of mRNA-1273

3.3. Part 1C-2, Heterologous Booster Dose

3.3.1. Primary Safety Endpoints

The primary safety objective will be evaluated by the following safety endpoints:

- Solicited local and systemic ARs through 7 days after BD
- Unsolicited AEs through 28 days after BD injection

- MAAEs post BD through the last day of study participation
- SAEs post BD through the last day of study participation
- AESIs post BD through the last day of study participation AEs leading to discontinuation from study participation post BD through the last day of study participation

3.3.2. Primary Immunogenicity Endpoints

- GM value of postbooster Ab (BD-Day 29) against ancestral strain

3.3.3. Key Secondary Endpoints

- GM value of postbooster Ab (BD-Day 29) against circulating strain

3.3.4. Exploratory Endpoints

The exploratory endpoints are the following:

- The GM values of SARS-CoV-2 S2P specific bAb on BD-Day 1, BD-Day 29 (1 month after BD), BD-Day 181 (6 months after BD), and BD-Day 361 (1 year after BD)
- The GM values of SARS-CoV-2-specific nAb on BD-Day 1, BD-Day 29 (1 month after BD), BD-Day 181 (6 months after BD), and BD Day 361 (1 year after BD)
- The incidence of SARS-CoV-2 infection (symptomatic or asymptomatic infection) counted starting 14 days after BD of mRNA-1273
- To evaluate the incidence of asymptomatic SARS-CoV-2 infection after vaccination with mRNA-1273 measured by RT-PCR and/or bAb levels against SARS-CoV-2 nucleocapsid protein (by Roche Elecsys) counted starting 14 days after BD in participants with negative SARS-CoV-2 at baseline or prebooster
- The incidence of the first occurrence of symptomatic COVID-19 starting 14 days after BD of mRNA-1273

3.4. Part 2

3.4.1. Primary Safety Endpoints

The primary safety objective will be evaluated by the following safety endpoints:

- Solicited local and systemic ARs through 7 days after each injection

- Unsolicited AEs through 28 days after each injection
- MAAEs through the entire study period
- SAEs through the entire study period
- AESIs through the entire study period
- AEs leading to discontinuation from study participation from Dose 1 through the last day of study participation

3.4.2. Primary Immunogenicity Endpoints

The primary immunogenicity objective will be evaluated by the following immunogenicity endpoints:

- GM values of both post Dose 1 (Day 29) and post Dose 2 (Day 57) Ab against ancestral strain in Part 2 of Study P203
- SRR of both post Dose 1 and post Dose 2 from baseline (pre Dose 1) in Part 2 of Study P203 against ancestral strain, using below definition
    - o Seroresponse is defined as Ab value change from baseline (pre Dose 1) below the LLOQ to ≥ 4 × LLOQ, or at least a 4-fold rise if baseline is ≥ LLOQ
    - o Seroresponse for sensitivity analysis is defined as Ab value change from baseline (pre Dose 1) below the LLOQ to ≥ 4 × LLOQ, or at least a 4-fold rise if baseline is ≥ LLOQ and < 4 × LLOQ, or at least 2-fold rise if baseline is ≥ 4 × LLOQ.

3.4.3. Exploratory Endpoints

The exploratory endpoints are the following:

- The GM values of SARS-CoV-2 S2P specific bAb on Day 1, Day 57 (1 month after Dose 2), Day 209 (6 months from Dose 2), Day 394 (1 year from Dose 2)
- The GM values of SARS-CoV-2-specific nAb on Day 1, Day 57 (1 month after Dose 2), Day 209 (6 months from Dose 2), Day 394 (1 year from Dose 2)
- The incidence of SARS-CoV-2 infection (symptomatic or asymptomatic infection) counted starting 14 days after Dose 2 of mRNA-1273

- To evaluate the incidence of asymptomatic SARS-CoV-2 infection after vaccination with mRNA-1273 measured by RT-PCR and/or bAb levels against SARS-CoV-2 nucleocapsid protein (by Roche Elecsys) counted starting 14 days after Dose 2 in participants with negative SARS-CoV-2 at baseline
- The incidence of the first occurrence of symptomatic COVID-19 starting 14 days after Dose 2 of mRNA-1273

3.5. Part 3

3.5.1. Primary Safety Endpoints

The primary safety objective will be evaluated by the following safety endpoints:

- Solicited local and systemic ARs through 7 days after each injection.
- Unsolicited AEs through 28 days after each injection.
- MAAEs through the entire study period.
- SAEs through the entire study period.
- AESIs through the entire study period.
- AEs leading to withdrawal from study participation from Dose 1 through the last day of study participation.
- AEs leading to discontinuation from dosing from Dose 1 through the last day of study participation.

3.5.2. Primary Immunogenicity Endpoints

The primary immunogenicity objective will be evaluated by the following endpoints:

- GM value of post Dose 1 (Day 29) of mRNA-1273.222 Ab against SARS-CoV-2 VOC (Omicron BA.4/BA.5) in adolescents who are baseline SARS-CoV-2 positive in Part 3 of Study P203 compared to that of post primary series of mRNA-1273 (post Dose 2 [Day 57] of 100 µg) in young adults 18 to 25 years of age who are baseline SARS-CoV-2 negative in Study P301 (superiority testing).
- GM value of post Dose 1 (Day 29) of mRNA-1273.222 Ab against ancestral strain in adolescents who are baseline SARS-CoV-2 positive in Part 3 of Study P203 compared to that of post primary series of mRNA-1273 (post Dose 2 [Day 57] of

100 µg) in young adults 18 to 25 years of age who are baseline SARS-CoV-2 negative in Study P301 (noninferiority testing).

3.5.3. Secondary Endpoints

The secondary objectives will be evaluated by the following endpoints:

- SRR of post Dose 1 (Day 29) from baseline (pre Dose 1) against Omicron BA.4/BA.5 in adolescents who are baseline SARS-CoV-2 positive in Part 3 of Study P203 compared to that of post primary series of mRNA-1273 (post Dose 2 [Day 57] of 100 µg) in adults 18 to 25 years of age in Study P301.
    - o Seroresponse at subject level is defined as an Ab value change from baseline (pre Dose 1) below the LLOQ to ≥ 4 × LLOQ, or at least a 4-fold rise if baseline is ≥ LLOQ.
- SRR of post Dose 1 (Day 29) from baseline (pre Dose 1) against ancestral strain in adolescents who are baseline SARS-CoV-2 positive in Part 3 of Study P203 compared to that of post primary series of mRNA-1273 (post Dose 2 [Day 57] of 100 µg) in adults 18 to 25 years of age in Study P301.
    - o Seroresponse at subject level is defined as an Ab value change from baseline (pre Dose 1) below the LLOQ to ≥ 4 × LLOQ, or at least a 4-fold rise if baseline is ≥ LLOQ.
- GM value of post Dose 2 (Day 209) of mRNA-1273.222 Ab against Omicron BA.4/BA.5 in Part 3 of Study P203 compared to that of post primary series of mRNA-1273 (post Dose 2 [Day 57] of 100 µg) in adults 18 to 25 years of age in Study P301.
- GM value of post Dose 2 (Day 209) of mRNA-1273.222 Ab against ancestral strain in Part 3 of Study P203 compared to that of post primary series of mRNA-1273 (post Dose 2 [Day 57] of 100 µg) in adults 18 to 25 years of age in Study P301.
- SRR of post Dose 2 (Day 209) from baseline (pre Dose 1) against Omicron BA.4/BA.5 in Part 3 of Study P203 compared to that of post primary series of mRNA-1273 (post Dose 2 [Day 57] of 100 µg) in adults 18 to 25 years of age in Study P301.

  - Seroresponse at subject level is defined as an Ab value change from baseline (pre Dose 1) below the LLOQ to ≥ 4 × LLOQ, or at least a 4-fold rise if baseline is ≥ LLOQ.

- SRR of post Dose 2 (Day 209) from baseline (pre Dose 1) against ancestral strain in Part 3 of Study P203 compared to that of post primary series of mRNA-1273 (post Dose 2 [Day 57] of 100 µg) in adults 18 to 25 years of age in Study P301.
  - Seroresponse at subject level is defined as an Ab value change from baseline (pre Dose 1) below the LLOQ to ≥ 4 × LLOQ, or at least a 4-fold rise if baseline is ≥ LLOQ.
- GM value of post Dose 1 (Day 29) of mRNA-1273.222 Ab against other variant(s) of interest in Part 3 of Study P203 as compared to that of post primary series of mRNA-1273 (post Dose 2 [Day 57] of 100 µg) in adults 18 to 25 years of age in Study P301.
- GM value of post Dose 2 (Day 209) of mRNA-1273.222 Ab against other variant(s) of interest in Part 3 of Study P203 as compared to that of post primary series of mRNA-1273 (post Dose 2 [Day 57] of 100 µg) in adults 18 to 25 years of age in Study P301.
- As sensitivity anlaysis, SRR of post Dose 1 (Day 29) or post Dose 2 (Day 209) from baseline (pre Dose 1) against Omicron BA.4/BA.5 compared to that of post primary series of mRNA-1273 (post Dose 2 [Day 57] of 100 µg) in adults 18 to 25 years of age in Study P301, using the following definition.
  - Seroresponse at subject level is defined as an Ab value change from baseline (pre Dose 1) below the LLOQ to ≥ 4 × LLOQ, or at least a 4-fold rise if baseline is ≥ LLOQ and < 4 × LLOQ, or at least a 2-fold rise if baseline is ≥ 4 × LLOQ.
- As sensitivity analysis, SRR of post Dose 1 (Day 29) or post Dose 2 (Day 209) from baseline (pre Dose 1) against the ancestral strain compared to that of post primary series of mRNA-1273 (post Dose 2 [Day 57] of 100 µg) in adults 18 to 25 years of age in Study P301, using following definition.
  - Seroresponse at subject level is defined as an Ab value change from baseline (pre Dose 1) below the LLOQ to ≥ 4 × LLOQ, or at least a 4-fold

rise if baseline is ≥ LLOQ and < 4 × LLOQ, or at least a 2-fold rise if baseline is ≥ 4 × LLOQ.

3.5.4. Exploratory Endpoints

The exploratory endpoints are the following:

- The GM values of SARS-CoV-2 S2P specific bAb on Day 1, Day 29 (1 month after Dose 1), Day 85 (3 months after Dose 1), Day 181 (Dose 2), Day 209 (1 month after Dose 2) and Day 361 (6 months after Dose 2).
- The GM values of SARS-CoV-2-specific nAb on Day 1, Day 29 (1 month after Dose 1), Day 85 (3 months after Dose 1), Day 181 (Dose 2), and Day 209 (1 month after Dose 2), and Day 361 (6 months after Dose 2).
- The incidence of the first occurrence of symptomatic COVID-19 starting 14 days after Dose 1 and Dose 2 of mRNA-1273.222.

4. Study Design

4.1. Overall Study Design

Overall study design is described in SAP for Part 1A. Please refer to SAP for Part 1A.

4.2. Statistical Hypothesis

4.2.1. Part 1C-1, Homologous Booster Dose

4.2.1.1.Primary Immunogenicity Endpoint

The immunogenicity analysis of BD vaccine response against the ancestral strain will be performed using the noninferiority tests of the 2 null hypotheses based on the 2 coprimary endpoints, respectively.

Coprimary Endpoint 1: Ab geometric mean (GM) value at BD-Day 29

The null hypothesis:

H^1_0: immunogenicity response to mRNA-1273 BD as measured by Ab GM value at BD-Day 29 in adolescents (12-<18 years of age) in Study P203 Part 1C-1 is inferior compared with Ab GM value at Day 57 (28 days after Dose 2) in the primary series of mRNA-1273 in young adults (18 to 25 years of age) in Study P301.

The noninferiority in Ab GM value at BD-Day 29 in Study P203 Part 1C-1 compared with Ab GM value at Day 57 in the primary series in young adults (18 to 25 years of age) in Study P301 will be demonstrated by meeting both success criteria:

- The lower bound of the 95% CI of the geometric mean ratio (GMR) rules out 0.667 (lower bound > 0.667) using a noninferiority margin of 1.5.
- The GMR point estimate ≥ 0.8 (minimum threshold).

The GMR is defined as the ratio of GM value of Ab at BD-Day 29 in Study P203 Part 1C-1 compared with Ab GM value at Day 57 (28 days after Dose 2) following the primary series of mRNA-1273 in Study P301.

Coprimary Endpoint 2: Ab Seroresponse Rate (SRR) at BD-Day 29

The null hypothesis:

H^2_0: immunogenicity response to mRNA-1273 BD as measured by SRR at BD-Day 29 in adolescents (12-<18 years of age) in Study P203 Part 1C-1 is inferior compared with SRR at Day 57 (28 days after Dose 2) following the primary series of mRNA-1273 in young adults (18 to 25 years of age) in Study P301.

- The noninferiority in SRR at BD-Day 29 compared with SRR at Day 57 (28 days after Dose 2) following the primary series of mRNA-1273 will be demonstrated by the lower bound of the 95% CI of the seroresponse rate difference ≥ -10% (i.e. lower bound > -10%) using the noninferiority margin of 10%.

The SRR difference is defined as the SRR at BD Day 29 in Study P203 Part 1C-1 minus the rate at Day 57 (28 days after Dose 2) following the primary series of mRNA-1273 in young adults in Study P301. The seroresponse is defined as Ab value change from baseline (pre Dose 1) below the LLOQ to ≥ 4 × LLOQ, or at least a 4-fold rise if baseline is ≥ LLOQ.

The primary immunogenicity objective in Part 1C-1 is met if the noninferiority is demonstrated based on both coprimary endpoints.

4.2.1.2.Key Secondary Immunogenicity Endpoint

Key Secondary Endpoint 1: GM value of Ab Against Circulating Strain at BD-Day 29

Null hypothesis:

H^1_0: immunogenicity response to mRNA-1273 BD as measured by GM value of Ab against circulating strain at BD-Day 29 in adolescents (12-<18 years of age) in Study P203 Part 1C-1 is inferior compared with GM value of Ab against circulating strain at Day 57 (28 days after Dose 2) in the primary series of mRNA-1273 in young adults (18 to 25 years of age) in Study P301.

The noninferiority in Ab GM value at BD-Day 29 in Study P203 Part 1C-1 compared with Ab GM value at Day 57 in the primary series in young adults (18 to 25 years of age) in Study P301 will be demonstrated by meeting both success criteria:

- The lower bound of the 95% CI of the geometric mean ratio (GMR) rules out 0.667 (lower bound > 0.667) using a noninferiority margin of 1.5.
- The GMR point estimate ≥ 0.8 (minimum threshold).

The GMR is defined as the ratio of GM value of Ab at BD-Day 29 in Study P203 Part 1C-1 compared with Ab GM value at Day 57 (28 days after Dose 2) following the primary series of mRNA-1273 in Study P301.

Key Secondary Endpoint 2: Seroresponse Rate (SRR) of Ab Against Circulating Strain at BD-Day 29

Null hypothesis:

H^2_0: immunogenicity response to mRNA-1273 BD as measured by SRR of Ab against circulating strain at BD-Day 29 in adolescents (12-<18 years of age) in Study P203 Part 1C-1 is inferior compared with SRR of Ab against circulating strain at Day 57 (28 days after Dose 2) following the primary series of mRNA-1273 in young adults (18 to 25 years of age) in Study P301.

The noninferiority in SRR at BD-Day 29 compared with SRR at Day 57 (28 days after Dose 2) following the primary series of mRNA-1273 will be demonstrated by the lower bound of the 95% CI of the seroresponse rate difference > -10% (i.e. lower bound > -10%) using the noninferiority margin of 10%.

The SRR difference is defined as the SRR at BD-Day 29 in Study P203 Part 1C-1 minus the rate at Day 57 (28 days after Dose 2) following the primary series of mRNA-1273 in young adults in Study P301. The seroresponse is defined as Ab value change from baseline (pre Dose 1) below the LLOQ to ≥ 4 × LLOQ, or at least a 4-fold rise if baseline is ≥ LLOQ.

The key secondary immunogenicity objective in Part 1C-1 is met if the noninferiority is demonstrated based on both key secondary endpoints.

4.2.2. Part 2

There will be no hypothesis testing in Part 2, given that the enrollment in Part 2 was discontinued (protocol Section 3.1.2). All analyses for Part 2 will be descriptive based on available data.

4.2.3. Part 3

Hypothesis testing for the endpoints that infer effectiveness of the 50-µg mRNA-1273.222 in adolescents will be performed on the null hypotheses for the 2 coprimary endpoints as follows:

Coprimary Endpoint 1: Post Dose 1 Ab GM value against Omicron BA.4/BA.5 at Day 29 (superiority testing)

The null hypothesis H^1_0: GM value of Ab against Omicron BA.4/BA.5 at Day 29 post Dose 1 of 50 µg mRNA-1273.222 in adolescents who are baseline SARS-CoV-2 positive is not superior to GM value at Day 57 post Dose 2 of 100 µg mRNA-1273 in baseline SARS-CoV-2 negative young adults (18 to 25 years of age) in Study P301.

The superiority in Ab GM value against Omicron BA.4/BA.5 in adolescents who are baseline SARS-CoV-2 positive compared with that in baseline SARS-CoV-2 negative young adults (18 to 25 years of age) is demonstrated by meeting the following success criterion:

- The lower bound of the 95% CI of the GMR >1.

The GMR is the ratio of the GM value against Omicron BA.4/BA.5 at Day 29 post Dose 1 of 50 µg mRNA-1273.222 in adolescents who are baseline SARS-CoV-2 positive in Study P203 Part 3 over the GM value against Omicron BA.4/BA.5 at Day 57 in baseline SARS-CoV-2 negative young adults (18 to 25 years of age) post Dose 2 of 100 µg mRNA-1273 in Study P301.

Coprimary Endpoint 2: Post Dose 1 Ab GM value against the ancestral strain at Day 29 (noninferiority testing)

The null hypothesis H^2_0: GM value of Ab against ancestral strain at Day 29 post Dose 1 of 50 µg mRNA-1273.222 in adolescents who are baseline SARS-CoV-2 positive is inferior

to GM value at Day 57 post Dose 2 of 100 µg mRNA-1273 in baseline SARS-CoV-2 negative young adults (18 to 25 years of age) in Study P301.

The noninferority in Ab GM value against ancestral strain in adolescents who are baseline SARS-CoV-2 positive compared with that in baseline SARS-CoV-2 negative young adults (18 to 25 years of age) is demonstrated by meeting the following success criterion:

- The lower bound of the 95% CI of the GMR >0.667.

The GMR is the ratio of the GM value against ancestral strain at Day 29 post Dose 1 of 50 µg mRNA-1273.222 in adolescents who are baseline SARS-CoV-2 positive in Study P203 Part 3 over the GM value against ancestral strain at Day 57 in baseline SARS-CoV-2 negative young adults (18 to 25 years of age) post Dose 2 of 100 µg mRNA-1273 in Study P301.

4.3. Sample Size and Power

4.3.1. Part 1C-1, Homologous Booster Dose

All participants enrolled in Part 1A or Part 1B who meet the eligibility criteria for BD will be offered a BD of mRNA-1273 50 µg. With more than 1,000 participants expected to receive mRNA-1273 BD, the study Part 1C-1 (Homologous Booster Dose) has a 90% probability to observe at least 1 participant with an AE at a true AE rate of 0.25%.

Serum samples from all participants will be collected and banked, a subset of participants will be selected, and their samples will be processed for immunogenicity testing (the Immunogenicity Subset) at specified timepoints.

Approximately 362 participants who receive mRNA-1273 BD will be selected for the Immunogenicity Subset for Part 1C-1, with a target of 289 participants receiving mRNA-1273 BD in the PP Immunogenicity Subset for Part 1C-1 (adjusting for approximately 20% of participants who may be excluded from the PP Immunogenicity Subset, as they may not have immunogenicity results due to any reason or may have protocol deviations impacting critical data).

For the primary immunogenicity objective in Part 1C-1, noninferiority tests of two null hypotheses based on two coprimary endpoints, respectively, will be performed. The sample size calculation for each of the two noninferiority tests was performed, and the larger sample size was chosen for the study.

- With approximately 289 participants receiving mRNA-1273 BD in the PP Immunogenicity Subset in Study P203 Part 1C-1 and 289 participants in the PP Immunogenicity Subset in young adults (18-25 years of age) in Study P301, there will be 90% power to demonstrate noninferiority of the immune response post BD as measured by Ab GM in adolescents in Study P203 Part 1C-1 compared with that in young adults (18-25 years of age) following primary series of mRNA-1273 in Study P301, at a 2-sided alpha of 0.05, assuming an underlying GMR value of 1, a noninferiority margin of 1.5, and a point estimate minimum threshold of 0.8. The standard deviation (SD) of the log-transformed levels is assumed to be 1.5.
- With approximately 289 participants receiving mRNA-1273 BD in the PP Immunogenicity Subset in Study P203 Part 1C-1 and 289 participants in the PP Immunogenicity Subset in young adults (18-25 years of age) in Study P301, there will be at least 90% power to demonstrate noninferiority of the immune response post BD as measured by seroresponse rate in adolescents in Study P203 Part 1C-1 compared with that in young adults (18-25 years of age) following primary series of mRNA-1273 in Study P301, at a 2-sided alpha of 0.05, assuming a true seroresponse rate of 90% in young adults (18-25 years of age) following primary series of mRNA-1273 in Study P301, and a true seroresponse rate of 90% post BD in adolescents in P203 Part 1C-1 (i.e., true rate difference is 0 compared to young adults [18-25 years of age] in Study P301), and a noninferiority margin of 10%.

4.3.2. Part 1C-2, Heterologous Booster Dose

Approximately 362 participants who received non-Moderna COVID-19 vaccine as primary series vaccination at least 3 months prior were planned to be enrolled to receive mRNA-1273 50 µg as heterologous booster in Part 1C-2. However, the study population in Part 1C-2 will have less than the planned number participants enrolled, as a result of slow enrollment and discontinuation of recruitment in Part 1C-2 (Protocol Section 3.1.1.4).

4.3.3. Part 2

Approximately 362 participants were planned to be enrolled to receive mRNA-1273 50 µg in the open label Part 2. However, the study population in Part 2 will have less than the planned number of participants receiving at least one dose of 50 µg mRNA-1273, with a small sample size as a result of slow enrollment and discontinuation of recruitment in Part 2 (Protocol Section 3.1.2).

4.3.4. Part 3

With at least 300 adolescents enrolled in Part 3 to receive 50 µg mRNA-1273.222 primary series, the study will have at least a 95% probability to observe at least 1 participant with an AE at a true AE rate of 1%.

Assuming a true serum Ab GMR of 1.6 for GM value against Omicron BA.4/BA.5 at Day 29 after Dose 1 of mRNA 1273.222 in baseline SARS-CoV-2 positive adolescents compared with GM value against Omicron BA.4/BA.5 at Day 57 after Dose 2 of mRNA-1273 primary series in young adults who are baseline SARS-CoV-2 negative in P301, with approximately 168 participants in the PP immunogenicity subset who are baseline SARS-CoV-2 positive in P203 Part 3 and 300 young adults in P301 who are baseline SARS-CoV-2 negative, there will be at least >90% power to demonstrate superiority of the serum Ab GM value against Omicron BA.4/BA.5 after Dose 1 of mRNA-1273.222 in adolescents in P203 to that after Dose 2 of mRNA-1273 in young adults in Study P301 at two-sided α of 0.05 using a superiority margin of 1.0. The standard deviation of the natural log transformed levels of Ab is assumed to be 1.5.

Assuming a true serum Ab GMR of 1.1 for GM value against ancestral strain at Day 29 after Dose 1 of mRNA 1273.222 in baseline SARS-CoV-2 positive adolescents compared with GM value against ancestral strain at Day 57 after Dose 2 of mRNA-1273 primary series in young adults who are baseline SARS-CoV-2 negative in P301, this sample size will also provide at least >90% power to demonstrate noninferiority of GM value of Ab against ancestral strain at Day 29 post Dose 1 of mRNA-1273.222 in adolescents in P203 to that after Dose 2 of mRNA-1273 in young adults in Study P301 at two-sided α of 0.05 using a noninferiority margin of 1.5. The standard deviation of the natural log transformed levels of Ab is assumed to be 1.5.

With approximately 30% of participants in Part 3 who may be excluded from the PP Immunogenicity Subset-baseline SARS-CoV-2 positive (PPIS-POS), as they may be baseline SARS-CoV-2 negative or have missing immunogenicity results due to any reason or protocol deviations impacting critical data, approximately 240 participants are required in Part 3 to provide 168 participants in the PP Immunogenicity Subset-baseline SARS-CoV-2 positive.

4.4. Multiplicity Adjustment

A hierarchical sequential hypothesis testing (fixed-sequence) method will be used to adjust multiplicity to preserve the family-wise Type I error rate (α = 0.05). The hypothesis testing for the 2 coprimary endpoints (geometric mean titer [GMT] and SRR) for the primary series of mRNA-1273 in Part 1A was completed and statistically significant based on data snapshot dated 08 May 2021, and thus the α level of 0.05 can be passed to Part 1C-1 hypothesis testing. In Part 1C-1 Homologous Booster Phase, the hypothesis testing for the 2 coprimary endpoints (GMT and SRR against the ancestral strain) after BD of mRNA-1273 will be tested first at α level of 0.05. The testing in Part 1A and 1C-1 will continue through the sequence only until an endpoint is not statistically significant (did not meet specified noninferiority success criteria), in which case the testing will stop. If the hypothesis testing for the 2 coprimary endpoints in Part 1C-1-Homologous Booster Phase is statistically significant (meeting the noninferiority success criteria of the coprimary endpoints), the α level of 0.05 will be passed to the hypothesis testing in Part 3.

For the key secondary objective in Part 1C-1 Homologous Booster Phase to evaluate immune response elicited by the 50 µg prototype booster of mRNA-1273 against variant(s) of interest, the key secondary endpoints will be analyzed independently, given that prototype vaccine mRNA-1273 does not contain variant specific sequences.

Part 3

Since the hypothesis testing for the coprimary endpoints in Part 1C-1 was statistically significant based on an IA with a data cutoff dated 16 May 2022, the α level of 0.05 (two-sided) was passed to Part 3 hypothesis testing.

4.5. Blinding and Unblinding

Not applicable.

5. Analysis Populations

The following analysis sets are defined: Full Analysis Set (FAS), Immunogenicity Subset , Per-protocol (PP) Immunogenicity Subset, Per-protocol (PP) Immunogenicity Subset - Pre-booster SARS-CoV-2 Negative for Part 1C-1, Modified Intent-to-Treat-1 (mITT1) Set, Solicited Safety Set (not applicable to Part 1B), and Safety Set.

5.1. Full Analysis Set

<u>The Full Analysis Set (FAS) (Long-term Analysis)</u>

The FAS (Long-term Analysis) consists of FAS (Part 1B, Open-Label Phase) for Placebo-mRNA-1273 cohort and FAS for mRNA-1273 cohort. FAS (Part 1B, Open-Label Phase) for Placebo-mRNA-1273 cohort consists of all participants from Part 1A placebo group who cross over and receive mRNA-1273 in Part 1B. FAS for mRNA-1273 cohort is same as the FAS in Part 1A. Participants will be analyzed in the mRNA-1273 group or placebo-mRNA-1273 group if applicable.

The Full Analysis Set (FAS) (Part 1C-1, Part 1C-2)

The FAS for Part 1C-1 and Part 1C-2 consists of all participants who received at least one booster dose in each part. Participants will be analyzed according to the treatment group assigned.

The Full Analysis Set (FAS) (Part 2, Part 3)

The FAS for Part 2 and Part 3 consists of all participants who received at least one dose of IP in each part. Participants will be analyzed according to the treatment group assigned.

5.2. Immunogenicity Subset

Immunogenicity Subset (Long-term Analysis at Day 1, Day 57, and Day 209)

Immunogenicity Subset for long-term analysis at Day 1, Day 57 and Day 209 consists of

a) participants selected for immune testing (Section 6.4.1), and

b) have baseline (pre-dose 1) SARS-CoV-2 status available, and

c) have baseline (pre-dose 1) and at least one post-injection antibody assessment for the analysis endpoint.

Immunogenicity Subset (Part 1C-1, Homologous Booster Dose)

Immunogenicity Subset for Part 1C-1 homologous booster dose consists of

a) participants selected for immune testing (Section 6.4.1), and

b) have baseline (pre-dose 1) SARS-CoV-2 status available, and

c) have at least one post-booster antibody assessment for the analysis endpoint.

Immunogenicity Subset (Part 1C-2, Heterologous Booster Dose)

Immunogenicity Subset for Part 1C-2 Heterologous booster dose consists of all participants who have at least one post-booster antibody assessment for the analysis endpoint.

Immunogenicity Subset (Part 2)

Immunogenicity Subset for Part 2 consists of all participants who have baseline (pre-dose 1) and at least one post-injection antibody assessment for the analysis endpoint.

Immunogenicity Subset (Part 3, at Day 29)

Immunogenicity Subset for Part 3, at Day 29 consists of all participants who have baseline (pre-dose 1) and at least one post-injection antibody assessment at Day 29.

Immunogenicity Subset (Part 3, at Day 209)

Immunogenicity Subset for Part 3, at Day 209 consists of all participants who have baseline (pre-dose 1) and at least one post-injection antibody assessment at Day 209.

5.3. Per-protocol (PP) Immunogenicity Subset

Per-protocol (PP) Immunogenicity Subset (Long-term Analysis at Day 1, Day 57 and Day 209)

Per-Protocol (PP) Immunogenicity Subset (Long-term Analysis) consists of all participants in Immunogenicity Subset (Long-term Analysis) who meet all the following criteria:

a) Received planned doses of study vaccination per schedule

b) Complied with the timing of second dose of injection

c) Had a negative SARS-CoV-2 status at baseline (pre-dose 1)

d) Had no major protocol deviations that impact key or critical data

The PP Immunogenicity Subset (Long-term Analysis) will serve as the population for the long-term analysis of immunogenicity data.

Per-protocol (PP) Immunogenicity Subset (Part 1C-1, Homologous Booster Dose)

Per-Protocol (PP) Immunogenicity Subset (Part 1C-1) consists of all participants in Immunogenicity Subset for Part 1C-1 who meet all the following criteria:

a) Received 2 doses of mRNA-1273 in Part 1A per schedule

b) Received booster dose in Part 1C-1

c) Had a negative SAS-CoV-2 status at baseline (pre-dose 1 of Part 1A)

d) Had BD-Day 29 Ab assessment for the analysis endpoint

e) Had no major protocol deviations that impact key or critical data

Per-protocol (PP) Immunogenicity Subset - Pre-booster SARS-CoV-2 Negative (Part 1C-1, Homologous Booster Dose)

PP Immunogenicity Subset - Pre-booster SARS-CoV-2 Negative for Part 1C-1 consists of participants who are in PP Immunogenicity Subset (Part 1C-1, Homologous Booster Dose), and are pre-booster SARS-CoV-2 negative, defined as no virologic or serologic evidence of SARS-CoV-2 infection on or before BD-Day 1 (pre-booster), i.e. RT-PCR result is not positive if available at BD-Day 1 and a negative bAb specific to SARS-CoV-2 nucleocapsid (as measured by Roche Elecsys Anti-SARS-CoV-2 assay) on or before BD-Day 1 .

PP Immunogenicity Subset - Pre-booster SARS-CoV-2 Negative for Part 1C-1 will serve as the population for the primary and key secondary analysis of immunogenicity data in Part 1C-1. The PP Immunogenicity Subset for Part 1C-1 will serve as the population for the analysis of immunogenicity in Part 1C-1 by pre-booster SARS-CoV-2 status (negative vs. positive).

Per-protocol (PP) Immunogenicity Subset (Part 1C-2, Heterologous Booster Dose)

Per-Protocol (PP) Immunogenicity Subset (Part 1C-2) consists of all participants in Immunogenicity Subset for Part 1C-2 who meet all the following criteria:

a) Received booster dose in Part 1C-2

b) Had BD-Day 29 Ab assessment for the analysis endpoint

c) Had no major protocol deviations that impact key or critical data

The PP Immunogenicity Subset for Part 1C-2 will serve as the population for the analysis of immunogenicity in Part 1C-2 by pre-booster SARS-CoV-2 status (negative vs. positive).

Per-protocol (PP) Immunogenicity Subset (Part 2)

Per-Protocol (PP) Immunogenicity Subset (Part 2) consists of all participants in Immunogenicity Subset (Part 2) who meet all the following criteria:

a) Received at least one dose of planned injection

b) Had Day 29 Ab assessment for the analysis endpoint

c) Had no major protocol deviations that impact key or critical data

Per-protocol (PP) Immunogenicity Subset - Baseline SARS-CoV-2 Positive (Part 2)

Per-Protocol (PP) Immunogenicity Subset - Baseline SARS-CoV-2 Positive (Part 2), abbreviated as PPIS-POS (Part2), consists of all participants in Per-protocoal Immunogenicity Subset (Part 2) who had serologic or virologic evidence of SARS-CoV-2 infection at baseline.

The PP Immunogenicity Subset – Baseline SARS-CoV-2 Positive (Part 2) will serve as the population for the analysis of immunogenicity data in Part 2.

Per-protocol (PP) Immunogenicity Subset (Part 3, at Day 29)

Per-Protocol (PP) Immunogenicity Subset (Part 3, at Day 29) consists of all participants in Immunogenicity Subset (Part 3, at Day 29) who meet all the following criteria:

a) Received planned doses of study vaccination per schedule

b) Had Day 29 Ab assessment for the analysis endpoint

c) Had no major protocol deviations that impact key or critical data

PP Immunogenicity Subset - Baseline SARS-CoV-2 Postive (Part 3, at Day 29)

PP Immunogenicity Subset - Baseline SARS-CoV-2 Postive (Part 3, at Day 29) consisting of all participants in PP Immunogenicity Subset (Part 3, at Day 29) and who have serologic and/or virologic evidence of SARS-CoV-2 infection at baseline, will serve as the population for the primary and secondary analysis of immunogenicity data at Day 29 in Part 3. Per-protocol (PP) Immunogenicity Subset (Part 3, at Day 29) will serve as the population for the analysis of immunogenicity at Day 29 by baseline SARS-CoV-2 status (negative vs. positive).

Per-protocol (PP) Immunogenicity Subset (Part 3, at Day 209)

Per-Protocol (PP) Immunogenicity Subset (Part 3, at Day 209) consists of all participants in Immunogenicity Subset (Part 3, at Day 209) who meet all the following criteria:

a) Received planned doses of study vaccination per schedule

b) Had Day 209 Ab assessment for the analysis endpoint

c) Had no major protocol deviations that impact key or critical data

The PP Immunogenicity Subset (Part 3, at Day 209) will serve as the population for the primary and secondary analysis of immunogenicity data at Day 209 in Part 3.

5.4. Modified Intent-to-Treat-1 (mITT1) Set

mITT1 Set (Long-term Analysis)

The mITT1 Set (Long-term Analysis) consists of mITT1 Set (Part 1B, Open-Label Phase) for Placebo-mRNA-1273 cohort and mITT1 Set for mRNA-1273 cohort. mITT1 Set (Part 1B, Open-Label Phase) for placebo-mRNA-1273 cohort consists of all cross-over participants in the FAS (Part 1B, Open-Label Phase) who had no serologic or virologic evidence of prior SARS-CoV-2 infection (both negative RT-PCR test for SARS-CoV-2 and negative serology test based on bAb specific to SARS-CoV-2 nucleocapsid) before the first dose of mRNA-1273 in Part 1B and received at least one dose of mRNA-1273 without wrong treatment, i.e., all FAS cross-over participants excluding those with positive or missing RT-PCR test or serology test prior to the first dose of mRNA-1273 and those who received the wrong treatment in Part 1B (i.e., at least one dose received in Part 1B is not as assigned). The mITT1 Set for mRNA-1273 cohort is same as the mITT1 Set in Part 1A.

mITT1 Set (Part 1C-1, Homologous Booster Dose)

The mITT1 Set for Part 1C-1 consists of all participants in the FAS for Part 1C-1 who had no serologic or virologic evidence of prior SARS-CoV-2 infection (both negative RT-PCR test for SARS-CoV-2 and negative serology test based on bAb specific to SARS-CoV-2 nucleocapsid) pre-booster dose and received one booster dose without wrong treatment, i.e., all FAS participants excluding those with pre-booster positive or missing RT-PCR test or serology test and those who received the wrong booster dose (i.e., dose received in Part 1C-1 is not as assigned).

Participants will be analyzed according to their treatment group (mRNA-1273-Booster, or Placebo-mRNA-1273-Booster).

5.5. Solicited Safety Set

Solicited Safety Set (Part 1C-1, Part 1C-2, Part 2 and Part 3)

The Solicited Safety Set consists of all participants who received any injection of IP in each part, and contribute any solicited AR data post injection. The Solicited Safety Set will be used for the analyses of solicited ARs in each part. Participants will be analyzed according to the treatment groups received. In addition, the following Solicited Safety Set is defined for each injection separately for Part 2 and Part 3. The First (Second) Injection Solicited Safety Set consists of all subjects in the Solicited Safety Set who have received

the first (second) study injection and have contributed any solicited AR data from the time of first (second) study injection through the following 6 days.

5.6. Safety Set

Safety Set (Long-term Analysis)

The Safety Set (Long-term Analysis) consists of Safety Set (Part 1B, Open-Label Phase) for Placebo-mRNA-1273 cohort and Safety Set for mRNA-1273 cohort. Safety Set (Part 1B, Open-Label Phase) consists of all participants from Part 1A placebo group who cross over and receive mRNA-1273 in Part 1B. The Safety Set for Long-term Analysis for mRNA-1273 cohort is same as the Safety Set in Part 1A. The Safety Set will be used for analysis of safety except for the solicited ARs. Participants will be analyzed in the mRNA-1273 group or Placebo-mRNA-1273 group.

Safety Set (Part 1C-1, Part 1C-2, Part 2 and Part 3)

The Safety Set for Part 1C-1, Part 1C-2, Part 2 and Part 3 consists of all participants who received any study injection of IP in each part. The Safety Set will be used for analysis of safety except for the solicited ARs. Participants will be analyzed according to the treatment group received.

6. Statistical Analysis

6.1. General Considerations

The Schedule of Assessments for each part is provided in SAP for Part 1A.

Continuous variables will be summarized using the following descriptive summary statistics: the number of subjects (n), mean, standard deviation (SD), median, minimum (min), and maximum (max).

Categorical variables will be summarized using counts and percentages.

Value of pre-first dose of mRNA-1273 for Part 1B, unless specified otherwise, is defined as the most recent non-missing measurement (scheduled or unscheduled) collected before the first dose of mRNA-1273 in Part 1B.

Pre-booster value for Part 1C-1, and Part 1C-2, unless specified otherwise, is defined as the most recent non-missing measurement (scheduled or unscheduled) collected before the booster dose. For immunogenicity tests, the pre-booster is defined as the most recent

non-missing measurement (scheduled or unscheduled) collected before or on the date of booster dose.

Baseline value for Part 2 and Part 3 is defined using the same approch as Part 1A.

For the summary statistics of all numerical variables unless otherwise specified, the display precision will follow programming standards. Please see Appendix A for variable display standards.

When count data are presented, the percentage will be suppressed when the count is zero in order to draw attention to the non-zero counts. A row denoted “Missing” will be included in count tabulations where specified on the shells to account for dropouts and missing values. The denominator for all percentages will be the number of subjects in that vaccination group within the analysis set of interest, unless otherwise specified.

Pre-booster SARS-CoV-2 Status

Pre-booster SARS-CoV-2 status for Part 1C-1 and Part 1C-2 is determined by using virologic and serologic evidence of SARS-CoV-2 infection on or before the date of booster dose (BD-Day 1).

Pre-booster positive SARS-CoV-2 status is defined as a positive RT-PCR test for SARS-CoV-2, and/or a positive serology test based on bAb specific to SARS-CoV-2 nucleocapsid (as measured by *Roche Elecsys* Anti-SARS-CoV-2 assay) on or before BD-Day 1.

Pre-booster negative status is defined as a negative RT-PCR test for SARS-CoV-2 and a negative serology test based on bAb specific to SARS-CoV-2 nucleocapsid (as measured by *Roche Elecsys* Anti-SARS-CoV-2 assay) on or before BD-Day 1.

Baseline SARS-CoV-2 status for Part 2 and Part 3 is defined using the same approch as Part 1A.

Study day relative to the first injection in Part 1B or Part 2 or Part 3 will be calculated as below:

a) study day prior to the first injection will be calculated as: date of assessment/event – date of the first injection in Part 1B or Part 2 or Part 3;

b) study day on or after the date of the first injection will be calculated as: date of assessment/event – date of the first injection in Part 1B or Part 2 or Part 3 + 1;

Study day relative to the booster injection in Part 1C-1 orPart 1C-2 will be calculated as below:

a) study day prior to the booster dose will be calculated as: date of assessment/event – date of the booster injection;

b) study day on or after the date of the booster dose will be calculated as: date of assessment/event – date of the booster injection + 1;

Study day relative to the most recent injection will be calculated as below:

a) study day on or after the date of the first injection (if applicable) but before the second injection (if applicable) will be calculated as: date of assessment/event – date of the first injection + 1;

b) study day on or after the date of the second injection (if applicable) but before the booster injection (if applicable) will be calculated as: date of assessment/event – date of the second injection + 1;

c) study day on or after the date of booster dose in Part 1C-1 (if applicable), or Part 1C-2 will be calculated as: date of assessment/event – date of the booster injection + 1;

if study day is on the same day as the injection, date and time will be compared with the injection date and time.

For calculation regarding antibody levels/titers, antibody values reported as below LLOQ will be replaced by 0.5 × LLOQ. Values that are greater than the upper limit of quantification (ULOQ) and without actual values reported (eg, '>xxx') will be converted to the ULOQ. Values that are greater than the ULOQ and with actual values reported will not be imputed (i.e. actual values will be used). Missing results will not be imputed.

The following **analysis periods for safety analyses** will be used as applicable for specific subjects cohort in this study:

- Up to 28 days after any vaccination: this stage starts at the day of each vaccination and continue through the earliest date of (the day of each vaccination and 27 subsequent days, next vaccination [if applicable]). This analysis period will be used as the primary analysis period for safety analyses including unsolicited AE, except for solicited AR, unless specified otherwise.

- Overall period or throughout the study: this analysis period starts at the first injection on Day 1 and continues through the earliest date of (study completion, discontinuation from the study, or death).

Unscheduled visits: Unscheduled visit measurements will be included in analysis as follows:

- In scheduled visit windows per specified visit windowing rules.
- In the derivation of baseline/last on-treatment measurements.
- In the derivation of maximum/minimum on-treatment values and maximum/minimum change from baseline values for safety analyses.
- In individual subject data listings as appropriate.

Visit windowing rules: The analysis visit windows for protocol-defined visits are provided in Appendix B.

Incomplete/missing data:

- Imputation rules for missing prior/concomitant medications, non-study vaccinations and procedures are provided in Appendix C.
- Imputation rules for missing AE dates are provided in Appendix D.
- For laboratory assessments, if majority of results are indefinite, imputation of these values will be considered. If the laboratory results are reported as below the LLOQ (e.g., <0.1), the numeric values will be imputed by 0.5 × LLOQ in the summary. If the laboratory results are reported as greater than the ULOQ (e.g., ">3000"), the numeric values will be imputed by ULOQ in the summary.
- Other incomplete/missing data will not be imputed, unless specified otherwise.

Treatment groups:

The following vaccination groups will be used for summary purposes:

- Long-term Analysis (Part 1A plus Part 1B):
    - mRNA-1273: Participants from Part 1A mRNA-1273 group
    - Placebo - mRNA-1273: Participants from Part 1A placebo group who cross over and receive mRNA-1273 in Part 1B

- Part 1C-1 Homologous Booster Dose Analysis:
    - mRNA-1273 - Booster: Participants from Part 1A mRNA-1273 group who received booster dose in Part 1C-1
    - Placebo - mRNA-1273 - Booster: Participants from Part 1A placebo group who crossed over to receive mRNA-1273 in Part 1B, and received booster dose in Part 1C-1.
- Part 1C-2 Heterologous Booster Dose Analysis: Heterologous Booster
- Part 2: mRNA-1273 50 µg
- Part 3: mRNA-1273.222 50 µg

Analysis periods

The following analysis periods and treatment groups will be used for Long-term Analysis (including Part 1B) and Part 1C-1 Homologous Booster Dose Analysis.

- Long-term Analysis (Part 1A plus Part 1B)

Cohort	Category	Start Date	End Date
mRNA-1273	Safety	Date of First Dose of mRNA-1273	Earliest date of booster dose, study discontinuation, study completion, death, or data cutoff
Placebo - mRNA-1273			
mRNA-1273	Efficacy/ Immunogenicity	Date of First Dose of mRNA-1273	Earliest date of booster dose (inclusive), study discontinuation, study completion, death, or data cutoff
Placebo - mRNA-1273			

Long-term analysis of incidence rate will also be performed for the entire study period after the first dose of mRNA-1273 regardlesss of booster dose.

- Part 1C-1 Homologous Booster Dose Analysis

Cohort	Category	Start	End
mRNA-1273 - Booster	Safety	Date of Booster Dose	Earliest date of study discontinuation, study completion, death, or data cutoff
Placebo - mRNA-1273 - Booster			

mRNA-1273 - Booster	Efficacy/ Immunogenicity	Date of Booster Dose (considered as pre-booster data)	Earliest date of study discontinuation, study completion, death, or data cutoff
Placebo - mRNA-1273 - Booster			

Subgroup Analysis

Safety, efficacy and immunogenicity endpoints may be analyzed in select subgroups specified below as applicable:

- Baseline SARS-CoV-2 Status (Positive, Negative)
- Pre-booster SARS-CoV-2 Status (Positive, Negative)
- Age (≥12 and <16 Years, ≥16 and <18 Years)
- Sex (Female, Male)
- Race
- Ethnicity
- At Risk for Severe COVID-19 (Yes, No), as defined in SAP for Part 1A

Analyses Approach

There are multiple parts in P203, all analyses and data summaries/displays will be provided by vaccination groups for each study part using appropriate analysis population unless otherwise specified.

All analyses will be conducted using SAS Version 9.4 or higher.

6.2. Background Characteristics

6.2.1. Subject Disposition

The number and percentage of subjects in the following categories will be summarized by vaccination group as defined in Section 6.1 based on specific analysis datasets for each part separately:

- Full Analysis Set

- Immunogenicity Subset
- Per-protocol (PP) Immunogenicity Subset
- Per-protocol (PP) Immunogenicity Subset - Pre-booster SARS-CoV-2 Negative (Part 1C-1 only)
- Per-protocol (PP) Immunogenicity Subset - Baseline SARS-CoV-2 Positive (Part 2 and Part 3)
- mITT1 Set (Long-term Analysis, Part 1C-1)
- Solicited Safety Set (except long-term analysis)
- Safety Set

The percentage will be based on subjects in that vaccination group within the Full Analysis Set for each part separately, except the Solicited Safety Set and Safety Set for which the percentages will be based on the vaccination group in the Safety Set (as treated) for each part separately.

For Part 1C-2, Part 2 and Part 3, The number of subjects in the following categories will be summarized based on subjects screened:

- Number of subjects screened
- Number and percentage of screen failure subjects and the reason for screen failure

The percentage of subjects who screen failed will be based on the number of subjects screened. The reason for screen failure will be based on the number of subjects who screen failed.

For long-term analysis, the number and percentage of subjects in each of the following disposition categories will be summarized by vaccination group based on the Randomization Set in Part 1A:

- Received first injection in Part 1A
- Received second injection in Part 1A
- Continuing and unblinded in open-label Part 1B
- Received cross-over first injection in Part 1B
- Received cross-over second injection in Part 1B

- Prematurely discontinued study vaccine during Part 1B and the reason for discontinuation
- Received booster dose in Part 1C-1
- Completed study
- Prematurely discontinued the study and the reason for discontinuation (for the entire study period, and for study parts in applicable subjects cohort)

For Part 1C-1 and Part 1C-2 analyses, the number and percentage of subjects in each of the following disposition categories will be summarized by vaccination group based on the Full Analysis Set for Part 1C-1 or Part 1C-2, respectively:

- Received booster dose
- Completed study
- Prematurely discontinued the study and the reason for discontinuation

For Part 2 and Part 3 analysis, the number and percentage of subjects in each of the following disposition categories will be summarized by vaccination group based on the Full Analysis Set for each part separately:

- Received each dose of IP
- Prematurely discontinued before receiving the second dose of IP and the reason for discontinuation
- Completed study
- Prematurely discontinued the study and the reason for discontinuation (for the entire study period, and for study parts in applicable subjects cohort)

A subject disposition listing will be provided, including informed consent, subjects who completed the study injection schedule, subjects who completed study, subjects who discontinued from study vaccine or who discontinued from participation in the study, with reasons for discontinuation for each part separately. A separate listing will be provided for screen failure subjects with reasons for screen failure in Part 1C-2, Part 2 and Part 3.

A subject who completed 12 months (except Part 3) or 6 months in Part 3 of follow up after the last injection received is considered to have completed the study.

6.2.2. Demographics

Descriptive statistics will be calculated for the following continuous demographic and baseline characteristics: age (years), weight (kg, z-score), height (cm, z-score), and body mass index (BMI) (kg/m^2, z-score). Number and percentage of subjects will be provided for categorical variables such as gender, race, ethnicity. The summaries will be presented by vaccination group as defined in Section 6.1 if applicable, based on the Safety Set, FAS, mITT1 Set, Immunogenicity Subset, and Per-protocol (PP) Immunogenicity Subset (by pre-booster SARS-CoV-2 status and overall for Part 1C-1, by baseline SARS-CoV-2 status and overall for Part 2 and Part 3).

6.2.3. Medical History

Medical history data will be coded by system organ class (SOC) and preferred term (PT) using the Medical Dictionary for Regulatory Activities (MedDRA).

The number and percentage of participants with any medical history will be summarized by SOC and PT based on the Safety Set. A participant will be counted only once for multiple events within each SOC and PT. SOC will be displayed in internationally agreed order. PT will be displayed in descending order of frequency and then alphabetically within SOC.

Medical history data will be presented in a listing.

Medical history data for participants who entered study in Part 1A will be provided in Part 1A analysis.

6.2.4. Prior and Concomitant Medications

Prior and concomitant medications and non-study vaccination will be coded using the World Health Organization (WHO) drug dictionary (WHODD). The summary of concomitant medications will be based on the Safety Set. Categorization of prior, concomitant, and post medications is summarized in Appendix C Table 4.

The number and percentage of subjects using concomitant medications and non-study vaccination during the 7-day follow-up period (i.e., on the day of injection and the 6 subsequent days) and during the 28-day follow-up period after each injection (i.e., on the day of injection and the 27 subsequent days) will be summarized by vaccination groups as defined in Section 6.1 as follows:

- Any concomitant medications and non-study vaccination within 7 Days Post Injection
- Any concomitant medications and non-study vaccination within 28 Days Post Injection
- Seasonal influenza vaccine within 28 Days Post Injection
- Antipyretic or analgesic medication within 28 Days Post Injection

A summary table of concomitant medications and non-study vaccination that continued or newly received at or after the first injection through 28 days after the booster injection will be provided by PT in descending frequency in the mRNA-1273 group (except in Part 3) or mRNA-1273.222 group in Part 3.

Medications taken to prevent pain or fever will be collected on eDiary and summaries will be provided based on the Solicited Safety Set by vaccination group as defined in Section 6.1 for booster injection, including within 7 days after injection, beyond 7 days after injection.

Concomitant and post medications and non-study vaccination will be presented in a listing.

6.2.5. Study Exposure

Study IP administration data will be presented in a listing for each Part separately.

Study duration will be summarized since the first injection, since the second injection in long-term analysis, in Part 2 and Part 3, and since booster injection in Part 1C-1, Part 1C-2.

6.2.6. Major Protocol Deviations

Major protocol deviations are a subset of protocol deviations that may significantly impact the completeness, accuracy, or reliability of the study data or that may significantly affect a subject's rights, safety, or well-being. Major protocol deviations rules will be developed and finalized before database lock.

The number and percentage of the subjects with each major protocol deviation type will be provided by vaccination group as defined in Section 6.1 based on the Full Analysis Set (FAS).

Major protocol deviations will be presented in a listing.

6.3. Safety Analysis

Safety and reactogenicity will be assessed by clinical review of all relevant parameters including solicited ARs (local and systemic), unsolicited AEs, SAEs, MAAEs, AESI, AEs leading to withdrawal from study vaccine and/or study participation, vital signs, and physical examination findings. Unsolicited AEs will be coded by SOC and PT according to the MedDRA. The Toxicity Grading Scale for Healthy Adult and Adolescent Volunteers Enrolled in Preventative Vaccine Clinical Trials (DHHS 2007) is used in this study for solicited ARs as presented in Table 6 from protocol.

All safety analyses will be based on the Safety Set, except summaries of solicited ARs which will be based on the Solicited Safety Set. All safety analyses will be provided by vaccination group unless otherwise specified.

6.3.1. Adverse Events

A treatment-emergent AE (TEAE) is defined as any event occurring during the study not before exposure to study vaccine or any event already present that worsens after exposure to study vaccine. [Note: worsening of a pre-existing condition after vaccination will be reported as a new AE.]

Adverse events will also be evaluated by the investigator for the coexistence of MAAE which is defined as an AE that leads to an unscheduled visit to a healthcare practitioner.

Unsolicited AEs will be coded by PT and SOC using MedDRA and summarized by vaccination group, and stage (up to 28 days after any vaccination, follow-up analysis period and overall stage; see Section 6.1 for definitions of vaccination group and stage).

All summary tables (except for the overall summary of AEs) for unsolicited AEs will be presented by SOC and PT for TEAEs with counts of subjects included. SOC will be displayed in internationally agreed order. PT will be displayed in descending order of frequency of mRNA-1273 (except in Part 3) or mRNA-1273.222 group in Part 3 and then alphabetically within SOC. When summarizing the number and percentage of subjects with an event, subjects with multiple occurrences of the same AE or a continuing AE will be counted once. Subjects will be presented according to the highest severity (the strongest relationship) in the summaries by severity (of related AEs), if subjects reported multiple events under the same SOC and/or PT.

Percentages will be based upon the number of subjects in the Safety Set for specified analysis within each vaccination group.

6.3.1.1. Incidence of Adverse Events

An overall summary of unsolicited TEAEs including the number and percentage of subjects who experience the following will be presented:

- Any unsolicited TEAEs
- Any serious TEAEs
- Any fatal TEAEs
- Any unsolicited medically-attended TEAEs
- Any unsolicited TEAEs leading to discontinuation from study vaccine
- Any unsolicited TEAEs leading to discontinuation from participation in the study
- Any unsolicited severe TEAEs
- Any AESI of MIS-C
- Any AESI other than MIS-C

The table will also include number and percentage of subjects with unsolicited TEAEs that are treatment-related in each of the above categories. Safety summary tables for Part 1C-1 will be provided separately from Part 1A and Part 1B.

In addition, listings containing individual subject adverse event data for unsolicited AEs, unsolicited TEAEs leading to discontinuation from study vaccine, unsolicited TEAEs leading to discontinuation from participation in the study, serious AEs, unsolicited medically-attended AEs, AESI of MIS-C and AESI other than MIS-C (including myocarditis and/or pericarditis) will be provided separately for placebo-mRNA-1273 group in Part 1B, and each group receiving the injection in Part 1C-1, Part 1C-2, Part 2 and Part 3. The safety data for the original mRNA-1273 group in open label will be included in Part 1A listings with a flag for open label phase.

6.3.1.2.TEAEs by System Organ Class and Preferred Term

The following summary tables of TEAEs will be provided by SOC and PT using frequency counts and percentages (i.e., number and percentage of subjects with an event):

- All unsolicited TEAEs
- All unsolicited TEAEs that are treatment-related

- All serious TEAEs
- All serious TEAEs that are treatment-related
- All unsolicited TEAEs leading to discontinuation from study vaccine
- All unsolicited TEAEs leading to discontinuation from participation in the study
- All unsolicited Severe TEAEs
- All unsolicited Severe TEAEs that are treatment-related
- All unsolicited medically-attended TEAEs
- All unsolicited medically-attended TEAEs that are treatment-related
- All AESI of MIS-C
- All AESI other than MIS-C

6.3.2. Solicited Adverse Reactions

An AR is any AE for which there is a reasonable possibility that the test product caused the AE. The term "Solicited Adverse Reactions" refers to selected signs and symptoms occurring after injection administration during a specified post-injection follow-up period (day of injection and 6 subsequent days). The solicited ARs are recorded by the subject in eDiary. The occurrence and intensity of selected signs and symptoms is actively solicited from the participant during a specified post-injection follow-up period (day of injection and 6 subsequent days), using a pre-defined checklist (i.e., solicited ARs).

The following local ARs will be solicited by the eDiary in each part except Part 1B: pain at injection site, erythema (redness) at injection site, swelling (hardness) at injection site, and localized axillary swelling or tenderness ipsilateral to the injection arm.

The following systemic ARs will be solicited by the eDiary in each part except Part 1B: headache, fatigue, myalgia (muscle aches all over the body), arthralgia (aching in several joints), nausea/vomiting, rash, fever, and chills.

The solicited ARs will be graded based on the grading scales presented in Table 6 in the protocol, modified from the Toxicity Grading Scale for Healthy Adult and Adolescent Volunteers Enrolled in Preventative Vaccine Clinical Trials (DHHS 2007). Investigator will assess Grade 4 events (with exception of fever).

If a solicited local or systemic AR continues beyond 7 days post injection, the participant will be prompted to capture solicited local or systemic AR in the eDiary until resolution.

All solicited ARs (local and systemic) will be considered causally related to injection.

Analyses of solicited ARs will be provided by treatment group based on the Solicited Safety Set, unless otherwise specified.

The number and percentage of subjects who reported each individual solicited local AR (has a severity grade of Grade 1 or greater) and solicited systemic AR (has a severity grade of Grade 1 or greater) during the 7-day follow-up period after each injection will be tabulated by vaccination group, severity grade, and injection. The number and percentage of subjects who reported each individual solicited AR will also be summarized by vaccination group, severity grade, days of reporting and injection.

The number and percentage of subjects experiencing fever (a temperature greater than or equal to 38.0°C/100.4°F by the oral, axillary, or tympanic route) by severity grade will be provided.

A two-sided 95% exact confidence interval (CI) using the Clopper-Pearson method will be provided for the percentage of subjects who reported any solicited local AR, solicited systemic AR, or any solicited AR.

The onset of individual solicited AR is defined as the time point after each injection at which the respective solicited AR first occurred. The number and percentage of subjects with onset of individual solicited AR will be summarized by vaccination group, study day relative to the corresponding injection (Day 1 through Day 7), and injection.

The number of days will be calculated as the last day – the first day + 1 when the solicited adverse reaction was reported starting within the 7 days of injection. If the solicited AR continues beyond 7 days, the days a solicited AR is reported after 7 days will be included (e.g., an event that lasted 5 days in the first 7 days post injection and 3 days beyond 7 days post injection, the duration will be reported as 8 (5+3) days.)

6.3.3. Pregnancy Tests

A point-of-care urine pregnancy test will be performed at the Screening Visit (Day 0) and before each vaccine dose. At any time, a pregnancy test either via blood or point-of-care urine can be performed, at the discretion of the investigator.

A by-subject listing will be provided for pregnancy tests for each Part separately.

6.3.4. Vital Sign Measurements

Vital sign measurements, including systolic and diastolic blood pressures, heart rate, respiratory rate, and body temperature, will be presented in a data listing. The values meeting the toxicity grading criteria (DHHS 2007) will be flagged in the data listing. The abnormalities meeting the toxicity grading criteria (Grade 2 or higher) in any vital sign measurement will be listed separately. If a subject has a vital sign result with Grade 2 or higher abnormality at any post injection visit, then all results of vital sign measurement for that subject will be presented in the listing.

Observed values and changes from baseline for all vital sign measurements will be summarized at each visit by vaccination group as defined in Section 6.1. Shift from baseline in the toxicity grades at each visit and shift from baseline in the toxicity grades to the worst post-baseline result will also be summarized by vaccination group.

6.4. Immunogenicity Analysis

Analysis Population for Long-term analysis

The analyses of immunogenicity in long-term analysis at Day 1, Day 57, Day 209 and Day 394 will be based on the PP Immunogenicity Subset (Long-term Analysis) and Immunogenicity Subset (Long-term Analysis).

Analysis Population for Part 1C-1

The analyses of immunogenicity in Part 1C-1 will be based on

- PP Immunogenicity Subset with Pre-booster SARS-CoV-2 Negative
- PP Immunogenicity Subset (including Pre-booster SARS-CoV-2 Negative and Positive)

The PP Immunogenicity Subset with pre-booster SARS-CoV-2 negative will be used in the primary and key secondary immunogenicity analyses in Part 1C-1, unless otherwise specified. The PP Immunogenicity Subset will be used in the immunogenicity analyses by pre-booster SARS-CoV-2 status (negative vs. positive).

Analysis Population for Part 1C-2

The analyses of immunogenicity in Part 1C-2 will be based on the PP Immunogenicity Set.

Analysis Population for Part 2

The analyses of immunogenicity in Part 2 will be based on the PP Immunogenicity Set and PP Immunogenicity Set - baseline SARS-CoV-2 Positive. The PP Immunogenicity Subset

- baseline SARS-CoV-2 Positive is the primary analysis population used in the immunogenicity analyses in Part 2, unless otherwise specified. PP Immunogenicity Set will be used in the immunogenicity analyses by baseline SARS-CoV-2 status (negative vs. positive).

Analysis Population for Part 3

The analyses of immunogenicity at Day 29 in Part 3 will be based on PP Immunogenicity Subset (Day 29) and PP Immunogenicity Subset - baseline SARS-CoV-2 Positive (Day 29). The PP Immunogenicity Subset - baseline SARS-CoV-2 Positive (Day 29) is the primary analysis population used in the immunogenicity analyses at Day 29 in Part 3, unless otherwise specified. PP Immunogenicity Set will be used in the immunogenicity analyses at Day 29 by baseline SARS-CoV-2 status (negative vs. positive).

The analyses of immunogenicity at Day 209 in Part 3 will be based on PP Immunogenicity Subset (Day 209).

Calculation of GM and GMFR

The geometric mean (GM) titer or level will be calculated using the following formula:

$$10^{\left\{\frac{\sum_{i=1}^{n} \log_{10}(t_i)}{n}\right\}}$$

where $t_1, t_2, \ldots, t_n$ are n observed immunogenicity titers or levels.

The geometric mean fold-rise (GMFR) measures the changes in immunogenicity titers or levels within subjects. The GMFR will be calculated using the following formula:

$$10^{\left\{\frac{\sum_{i=1}^{n} \log_{10}\left(v_{ij}/v_{ik}\right)}{n}\right\}} = 10^{\left\{\frac{\sum_{i=1}^{n} \log_{10}(v_{ij}) - \log_{10}(v_{ik})}{n}\right\}}$$

where, for n subjects, v_{ij} and v_{ik} are observed immunogenicity titers or levels for subject i at time points j and k, $j \neq k$

6.4.1. Sampling of the Immunogenicity Subset

In long-term and Part 1C-1, for the primary analysis of immunogenicity, and charactering immunogenicity of the vaccine, a simple sampling method will be used for measuring bAb and nAb data from a sampled subset of trial participants in Part 1C-1.

Sampling Plan

The first ~400 participants in the mRNA-1273 group who received booster dose (third dose, starting from 27Dec2021) in Part 1C-1 and meet all the criteria below will be selected for the immunogenicity subset.

- The participant is in Full Analysis Set (Part 1C-1, Homologous Booster Dose).
- SARS-CoV-2 status is not missing for baseline (pre-dose 1).

The blood samples of these subjects collected at the long-term timepoints (Day 1, Day 57, and Day 209) and subsequent booster dose phase timepoints (BD-Day 1 and BD-Day 29) will have immunogenicity testing, to support both the long-term immunogenicity analysis and booster dose immunogenicity analysis.

The target is to obtain 289 participants in the PP Immunogenicity Subset with pre-booster negative SAR-CoV-2 status (adjusting for approximately 25% of participants who may be excluded as they may have pre-booster positive SAR-CoV-2 status, or have no immunogenicity results due to any reason).

For the noninferiority tests of Ab GM and seroresponse rate at BD-Day 29 in adolescents in Study P203 compared with the primary series GM at Day 57 in young adults (18-25 years of age) in Study P301 receiving mRNA-1273, an immunogenicity subset of 340 young adults in Study P301 randomly selected from all participants (18-25 years of age) receiving mRNA-1273 will be used, with a target of 289 participants in the PP Immunogenicity Subset (using same definition as in Study P203) after adjusting for approximately 15% of participants not meeting inclusion criteria for PP Immunogenicity Subset.

6.4.2. Immunogenicity Assessments

There will be two types of immunogenicity assessments in each part except Part 1B:

- Serum bAb by a multiplex serology assay specific to the SARS-CoV-2 S protein

- Serum nAb titer against SARS-CoV-2 as measured by pseudovirus and/or live virus neutralization assays

6.4.3. Primary Analysis of Antibody-Mediated Immunogenicity Endpoints

Part 1C-1

In the Part 1C-1 homologous booster phase, the noninferiority of the coprimary endpoints (Ab GM and SRR against the ancestral strain) in adolescents compared with those at Day 57 (28 days after Dose 2) in young adults (18-25 years of age) receiving mRNA-1273 in Study P301 will be assessed. The study is considered to meet the primary immunogenicity objective if the noninferiority of the immune response to mRNA-1273 as measured by both GM and seroresponse rate at BD-Day 29 is demonstrated in adolescents in this study at a 2-sided alpha of 0.05, compared with those at Day 57 (28 days after Dose 2) in young adults (18-25 years of age) in Study P301 receiving mRNA-1273.

The GM with 95% CI will be summarized using t-distribution of the log transferred values and then back transformed to the original scale. The GMR with 95% CI to compare post-booster GM at BD-Day 29 in adolescents in Study P203 with the primary series GM at Day 57 (28 days after Dose 2) in young adults (18-25 years of age) in Study P301 will be computed based on the t-distribution of mean difference in the log transferred values and then back transformed to the original scale. The noninferiority of immune response to mRNA-1273 as measured by GM will be considered demonstrated if the lower bound of the 95% CI of the GMR is > 0.667 based on the noninferiority margin of 1.5, and GMR point estimate ≥ 0.8 (minimum threshold).

The number and percentage (rate) of participants achieving Ab seroresponse at BD-Day 29 will be summarized. The SRR difference with 95% CI (using Miettinen-Nurminen score method) to compare post-booster SRR at BD-Day 29 in adolescents in Study P203 with the primary series SRR at Day 57 (28 days after Dose 2) in young adults in Study P301 will be calculated. The noninferiority in seroresponse rate of adolescents in P203 compared to adults of 18-25 years of age in P301 will be considered demonstrated if the lower bound of the 95% of the seroresponse rate difference is > -10% based on the noninferiority margin of 10%.

Part 1C-2

GM value of postbooster Ab (BD-Day 29) against ancestral strain with 95% CI will be summarized using t-distribution of the log transferred values and then back transformed to the original scale.

Part 2

GM value of both post Dose 1 (Day 29) and post Dose 2 (Day 57) against ancestral strain with 95% CI will be summarized using t-distribution of the log transferred values and then back transformed to the original scale.

SRR of post Dose 1 (Day 29) and post Dose 2 (Day 57) from baseline (pre Dose 1) against ancestral strain with 95% CI (using Clopper-Pearson method) will be summarized. Seroresponse at subject level is defined as an Ab value change from baseline (pre Dose 1) below the LLOQ to ≥ 4 × LLOQ, or at least a 4-fold rise if baseline is ≥ LLOQ.

Descriptive immunogenicity analysis will be preformed to compare to P203 antibody responses (both GM and SRR) at post Dose 1 (Day 29) and post Dose 2 (Day 57) with that from young adults (18-25 years of age) in P301 at Day 57.

For the immunogenicity descriptive analysis comparison of P203 Day 29 with P301 Day 57, the analysis population for P203 will be based on Per-Protocol (PP) Immunogenicity Subset - Baseline SARS-CoV-2 Positive (Part 2, at Day 29) and the analysis population for P301 will be based on baseline SAR-CoV-2 negative participants.

Part 3

Superiority in Ab GM value against Omicron BA.4/BA.5 at Day 29

In Part 3 analysis, the superiority of the first coprimary endpoint (Ab GM against Omicron BA.4/BA.5 at Day 29 after Dose 1 of 50 µg mRNA-1273.222) in adolescents in Study P203 compared with those at Day 57 (28 days after Dose 2 of 100 µg mRNA-1273) in young adults 18 to 25 years of age in Study P301 will be assessed. The analysis population for P203 will be based on Per-Protocol (PP) Immunogenicity Subset - Baseline SARS-CoV-2 Positive (Part 3, at Day 29) and the analysis population for P301 will be based on baseline SARS-CoV-2 negative participants.

An ANCOVA model will be carried out with Ab value at Day 29 in P203 and Ab value at Day 57 in P301 as a dependent variable and a group variable (baseline SARS-CoV-2 positive adolescents in Study P203 and baseline SARS-CoV-2 negative young adults in Study P301) as the fixed variable, where a covariate may be included in the model. The

GM value at Day 29 in P203 and the GM value at Day 57 in P301, and respective GMR for P203 compared to P301 will be estimated from the model. A corresponding 2-sided 95% CI of GMR estimated from the ANCOVA model will be provided to assess the difference in immune response after Dose 1 (Day 29) for the adolescents in Study P203 compared to that after Dose 2 (Day 57) in the young adults (18 to 25 years of age) in Study P301.

The first coprimary immunogenicity endpoint, the superiority of the immune response to mRNA-1273.222 against Omicron BA.4/BA.5 at Day 29 after Dose 1 compared to mRNA-1273 against Omicron BA.4/BA.5 after Dose 2 as measured by GM value will be considered demonstrated if the respective GMR 95% CI lower bound is >1 based on a superiority margin of 1.

Noninferiority in Ab GM value against ancestral strain at Day 29

The noninferiority of the second coprimary endpoint (Ab GM against ancestral strain at Day 29 after Dose 1 of 50 µg mRNA-1273.222) in adolescents in Study P203 compared with those at Day 57 (28 days after Dose 2 of 100 µg mRNA-1273) in young adults 18 to 25 years of age in Study P301 will be assessed. The analysis population for P203 will be based on Per-Protocol (PP) Immunogenicity Subset - Baseline SARS-CoV-2 Positive (Part 3, at Day 29) and the analysis population for P301 will be based on baseline SAR-CoV-2 negative participants.

An ANCOVA model will be carried out with Ab value at Day 29 in P203 and Ab value at Day 57 in P301 as a dependent variable and a group variable (baseline SARS-CoV-2 positive adolescents in Study P203 and baseline SARS-CoV-2 negative young adults in Study P301) as the fixed variable, where a covariate may be included in the model. The GM value at Day 29 in P203 and the GM value at Day 57 in P301, and respective GMR for P203 compared to P301 will be estimated from the model. A corresponding 2-sided 95% CI of GMR estimated from the ANCOVA model will be provided to assess the difference in immune response after Dose 1 (Day 29) for the adolescents in Study P203 compared to that after Dose 2 (Day 57) in the young adults (18 to 25 years of age) in Study P301.

The second coprimary endpoint, noninferiority of immune response to mRNA-1273.222 against ancestral strain after Dose 1 compared to mRNA-1273 against the ancestral strain after Dose 2 as measured by GM value will be considered demonstrated if the respective GMR 95% CI lower bound is >0.667 based on the noninferiority margin of 1.5.

6.4.4. Key Secondary Analysis of Antibody-Mediated Immunogenicity Endpoints

Part 1C-1

In the Part 1C-1 homologous booster phase, the noninferiority of key secondary endpoints (Ab GM and SRR against the circulating strain) will be assessed and tested using the same method as primary analysis.

Part 1C-2

GM value of postbooster Ab (BD-Day 29) against circulating strain with 95% CI will be summarized using t-distribution of the log transferred values and then back transformed to the original scale.

6.4.5. Secondary Analysis of Antibody-Mediated Immunogenicity Endpoints

Part 3

SRR against Omicron BA.4/BA.5 at Day 29

Descriptive analysis of SRR against Omicron BA.4/BA.5 at Day 29 after Dose 1 of 50 µg mRNA-1273.222 in adolescents in Study P203 will be compared with those at Day 57 (28 days after Dose 2 of 100 µg mRNA-1273) in young adults 18 to 25 years of age in Study P301.

The number and percentage (rate) of participants achieving Ab seroresponse at Day 29 with specified (95%) CI (using Clopper-Pearson method) will be summarized. The SRR difference with 95% CI (using Miettinen-Nurminen score method) to compare SRR at Day 29 after Dose 1 of mRNA-1273.222 in adolescents in Study P203 with the SRR at Day 57 after Dose 2 of mRNA-1273 in young adults in Study P301 will be calculated. Seroresponse at subject level is defined as an Ab value change from baseline (pre Dose 1) below the LLOQ to ≥ 4 × LLOQ, or at least a 4-fold rise if baseline is ≥ LLOQ.

As sensitivity analysis, SRR against Omicron BA.4/BA.5 at Day 29 defined using the second definition below will be analyzed using the same approach as above.

- Seroresponse at subject level is defined as an Ab value change from baseline (pre Dose 1) below the LLOQ to ≥ 4 × LLOQ, or at least a 4-fold rise if baseline is ≥ LLOQ and < 4 × LLOQ, or at least a 2-fold rise if baseline is ≥ 4 × LLOQ

SRR against ancestral strain at Day 29

The secondary endpoint of SRR against ancestral strain at Day 29 will be assessed using the same approach as SRR against Omicron BA.4/BA.5 at Day 29.

Ab GM value against Omicron BA.4/BA.5 at Day 209

The secondary endpoint of Ab GM against Omicron BA.4/BA.5 at Day 209 after Dose 2 of 50 µg mRNA-1273.222 with 95% CI will be summarized using t-distribution of the log transferred values and then back transformed to the original scale. The GMR with 95% CI to compare Ab GM at Day 29 in adolescents in Study P203 with the primary series GM at Day 57 (28 days after Dose 2) in young adults (18-25 years of age) in Study P301 will be computed based on the t-distribution of mean difference in the log transferred values and then back transformed to the original scale.

Ab GM value against ancestral strain at Day 209

The secondary endpoint of Ab GM against ancestral strain at Day 209 will be assessed using the same approach as the secondary endpoint of Ab GM against Omicron BA.4/BA.5 at Day 209 after Dose 2 of 50 µg mRNA-1273.222.

SRR against Omicron BA.4/BA.5 at Day 209

The secondary endpoint of SRR against ancestral strain at Day 209 after Dose 2 of 50 µg mRNA-1273.222 will be assessed using the same approach as the secondary endpoint of SRR against Omicron BA.4/BA.5 at Day 29 after Dose 1 of 50 µg mRNA-1273.222.

SRR against ancestral strain at Day 209

The secondary endpoint of SRR against ancestral strain at Day 209 after Dose 2 of 50 µg mRNA-1273.222 will be assessed using the same approach as the secondary endpoint of SRR against Omicron BA.4/BA.5 at Day 29 after Dose 1 of 50 µg mRNA-1273.222.

GM value of post Dose 1 (Day 29) or post Dose 2 (Day 209) of mRNA-1273.222 Ab against other variant(s) of interest with 95% CI will be assessed using the same approach as the secondary endpoint of Ab GM against Omicron BA.4/BA.5 at Day 209 after Dose 2 of 50 µg mRNA-1273.222.

6.4.6. Exploratory Analysis of Antibody-Mediated Immunogenicity Endpoints

For each group applicable, the following evaluations will be performed at each time point at which blood samples are collected for immunogenicity antibody tests against SAS-CoV-2 prototype or variants of interest as applicable.

- GM level of SARS-CoV-2-specific Ab levels with corresponding 95% CI will be provided at each time point (e.g., Baseline [Pre-dose 1], Day 29, Day 57, Day 209, Pre-booster, and BD-Day 29). The 95% CIs will be calculated based on the t-distribution of the log-transformed values then back transformed to the original scale for presentation. GM level will be plotted at each timepoint using boxplot. The following descriptive statistics will be also provided at each time point: the number of subjects (n), median, minimum and maximum.
- GM fold-rise of SARS-CoV-2-specific Ab levels with corresponding 95% CI will be provided at each timepoint over baseline (pre-dose 1) level, and post-booster timepoint over pre-dose 1 level and pre-booster level (Part 1C only). The 95% CIs will be calculated based on the t-distribution of the log-transformed values then back transformed to the original scale for presentation. The following descriptive statistics will be also provided at each time point: the number of subjects (n), median, minimum and maximum.

  Proportion of subjects with fold-rise ≥ 2 of serum SARS-CoV-2 specific Ab levels from pre-dose 1 and pre-booster (Part 1C only) if applicable at each time point will be tabulated with 2-sided 95% Clopper Pearson CIs.
- Proportion of subjects with seroresponse due to vaccination relative to pre-dose 1 will be tabulated with 2-sided 95% Clopper-Pearson CIs at each timepoint.
- For Part 1C-1, An analysis of covariance (ANCOVA) model may be performed to assess the difference in the post-booster GMT or GM values at BD-Day 29 in adolescents in Study P203 with the primary series GM at Day 57 (28 days after Dose 2) in young adults (18 to 25 years of age) in Study P301. In the ANCOVA model, antibody titer/level at BD-Day 29 will be a dependent variable, and a group variable (adolescents in P203 and adults in P301) will be the fixed effect. The GMT will be estimated by the geometric least square mean (GLSM) from the model and its corresponding 95% will be provided for each group. The GMR (ratio of GMTs) for adolescents in P203 with respect to adults in P301 will be estimated by the ratio

of GLSM from the model and the corresponding 95% CIs will be provided. The 95% CI for GMR will be used to assess the between group difference in immune response at BD-Day 29 in adolescents in P203 compared to that at Day 57 in young adults in P301.

- For Part 1C-1, the seroresponse rate comparisons between post-booster SRR at BD-Day 29 in adolescents in Study P203 with the primary series SRR at Day 57 (28 days after Dose 2) in young adults in Study P301 may be performed using the Ab measures change from pre-booster below the LLOQ to ≥ 4 × LLOQ, or at least a 4-fold rise if pre-booster is ≥ LLOQ for BD-Day 29 in P203.

- For Part 1C-1, an exploratory analysis of the primary immunogenicity endponts or key secondary immunogenicity endpoints after booster dose may be performed to compare the post-booster dose GMT or GM level, and SRR in P203 with those in P301 adult immunugneicity subset in the booster dose phase.

  An analysis of covariance (ANCOVA) model may be performed to assess the difference in the GMT or GM values at BD-Day 29 between adolescents receiving mRNA-1273 booster dose (third dose) in P203 and adults (≥18 years of age) receiving mRNA-1273 booster dose (third dose) in P301. In the ANCOVA model, antibody titer/level at BD-Day 29 will be a dependent variable, and a group variable (adolescents in P203 and adults in P301) will be the fixed effect, adjusting for pre-booster antibody titer/level, if applicable. The GMT will be estimated by the geometric least square mean (GLSM) from the model and its corresponding 95% will be provided for each group. The GMR (ratio of GMTs) for adolescents in P203 with respect to adults in P301 will be estimated by the ratio of GLSM from the model and the corresponding 95% CIs will be provided. The 95% CI for GMR will be used to assess the between group difference in immune response at BD-Day 29 in adolescents in P203 compared to adults in P301.

  The SRR difference with 95% CI (using Miettinen-Nurminen score method) to compare post-booster SRR at BD-Day 29 in adolescents in Study P203 with the post-booster SRR at BD-Day 29 in adults in Study P301 will be calculated. The analysis of seroresponse relative to pre-dose 1 and pre-booster dose will be conducted respectively.

- For Part 1C-1, Paired comparisons between visits (e.g. Day 209 vs. Day 57, BD-Day 29 vs. Day 57) will be performed in P203 if same assay tests are available at

both visits within subjects. GMT ratios may be calculated by back transforming the mean of paired differences of antibody titer data on the logarithmic scale between visits (e.g. Day 209 vs. Day 57, BD-Day 29 vs. Day 57). CIs for the GMT ratio will be based on t-distribution of the log-transformed values (paired differences) then back transformed to the original scale for presentation. The SRR difference between visits (e.g. Day 209 vs. Day 57, BD-Day 29 vs. Day 57) in P203 may be provided with 95% CI using the Adjusted Wald method (Bonett D et al 2012) for difference of paired proportions within subjects.

- Durability of immune response in Part 1C-1 or long-term: Mixed Model for Repeated Measures (MMRM)

To characterize the immunogenicity of mRNA-1273 in SARS-CoV-2 Ab including durability of immune-response (e.g. in the long-term analysis at Day 57 and Day 209), an exploratory analysis using a mixed model for repeated measures (MMRM) may be conducted. If performed, this analysis will be based on the participants receiving mRNA-1273 in the Per-protocol (PP) Immunogenicity Subset with negative baseline SARS-CoV-2 status.

For each select SARS-CoV-2-specific nAb and S protein-specific bAb of interest, the model will include all available log-transformed antibody titers at each post-Dose 1 of mRNA-1273 timepoints as the dependent variable. No treatment group variable in the model as all subjects in the model have received primary series of mRNA-1273. The model will include Visit (as a class variable, e.g. Day 57, Day 209) as fixed effects, and subject as a random effect. The model may also adjust for other covariates such as age groups and sex. An unstructured covariance structure will be used to model the within-subject errors. A Kenward-Roger approximation will be used for the denominator degrees of freedom. If there is a convergence issue due to the unstructured covariance matrix, a compound symmetry covariance structure will be used to model the within-subject errors. No imputation of missing data will be done.

GMT at each post-Dose 1 timepoint will be estimated by the geometric least squares mean (GLSM) and corresponding 2-sided 95% CI estimated from the model. Ratio of GMT (fold change) between specified two timepoints will be estimated by the ratio of GLSM. For example, fold change from Day 57 to Day 209 could be estimated by the ratio of GLSM from the model. The corresponding 95% CI results in log-transformed scale estimated from the model will be back-transformed to obtain these estimates in the original scale.

- In Part 3, for the primary endpoints (Ab GM value against Omicron BA.4/BA.5 and ancestral strain at Day 29 after Dose 1 of 50 µg mRNA-1273.222 in adolescents in Study P203 compared with those at Day 57 after Dose 2 of 100 µg mRNA-1273 in adults 18 to 25 years of age in Study P301), the following analyses will be performed. The GM with 95% CIs will be summarized using t-distribution of the log transferred values and then back transformed to the original scale. The GMR with 95% CIs to compare Ab GM value at Day 29 or Day 209 in adolescents in Study P203 with those at Day 57 (28 days after Dose 2 of 100 µg mRNA-1273) in young adults (18 to 25 years of age) in Study P301 will be computed based on the t-distribution of mean difference in the log transferred values and then back transformed to the original scale.

6.5. Efficacy Analysis

Analyses of the exploratory efficacy endpoints (incidence rates in COVID-19, SARS-CoV-2 infection, and asymptomatic infection) will be performed using the PP Set for Efficacy and mITT1 Set for long-term and Part 1C-1, and using the FAS for Part 1C-2, Part 2 and Part 3. The PP Set for Efficacy used in blinded phase for mRNA-1273 arm and the mITT1 Set for the long-term analysis will be used for the efficacy analysis in the long term including both the blinded and open label phases; the mITT1 Set for the booster-dose Part 1C-1will be used for the efficacy analysis in the Part 1C-1 booster dose phase; the FAS will be used for the efficacy analysis in Part 1C-2, Part 2 and Part 3, unless otherwise specified. Subjects will be included in the vaccination group as defined in Section 6.1.

Baseline SARS-CoV-2 status and Pre-booster SARS-CoV-2 status is described in Section 6.1. Baseline SARS-CoV-2 status in Part 2 and Part 3, Pre-booster SARS-CoV-2 status in Part 1C-1 and Part 1C-2, the serology test results based on *Roche Elecsys* assay and the RT-PCR test results will be summarized by treatment group.

For mRNA-1273 arm, PP Set for Efficacy and mITT1 set is same as those used in blinded phase. For Placebo-mRNA-1273 cross-over arm, participants with positive or missing SARS-CoV-2 status before the first dose of mRNA-1273 will be excluded from the mITT1 Set for long-term efficacy analysis.

Participants with pre-booster positive or missing SARS-CoV-2 status will be excluded from the mITT1 Set for Part 1C-1 booster dose efficacy analysis.

The serology test results based on Roche Elecsys assay and the RT-PCR test results will be summarized by visit.

6.5.1. Endpoint Definition/Derivation

6.5.1.1.Derivation of SARS-CoV-2 Infection

This is an exploratory efficacy endpoint, which is a combination of COVID-19 and asymptomatic SARS-CoV-2 infection for participants with negative SARS-CoV-2 status at baseline (for mRNA-1273 group in long-term analysis), pre-dose 1 of mRNA-1273 (for Placebo-mRNA-1273 group in long-term analysis), or pre-booster dose (for Part 1C-1 booster dose analysis), the incidence of SARS-CoV-2 infection counted starting 14 days after the second dose of mRNA-1273 in long-term (including Part 1B), and cases counted starting 14 days after the booster dose of mRNA-1273 in Part 1C-1. SARS-CoV-2 infection will be defined in participants with negative SARS-CoV-2 at baseline of each part (pre-dose 1 of mRNA-1273 or pre-booster dose):

- bAb level against SARS-CoV-2 nucleocapsid protein negative (as measured by *Roche Elecsys*) at baseline of each part that becomes positive (as measured by *Roche Elecsys*) post-baseline, OR
- Positive RT-PCR post-baseline.

Derivation of this secondary efficacy endpoint is summarized in Table 1 below.

Table 1. Derivation for SARS-CoV-2 Infection

	Post-baseline assessments		
**Baseline SARS-CoV-2 Status**	PCR test post baseline	bAb levels against SARS-CoV-2 Nucleocapsid	**Endpoint**: SARS-CoV-2 infection
Negative at Baseline of each part	**Positive** (either at scheduled NP swab test, or at exposure or symptom-prompt NP swab test)		Case
Negative at Baseline of each part		**Positive** (at scheduled post-baseline visits) as measured by *Roche Elecsys*	Case

The date of documented infection will be the earlier of:

- Date of positive post-baseline RT-PCR result, or
- Date of positive serology test result based on bAb specific to SARS-CoV-2 nucleocapsid

In long-term analysis (including Part 1B), SARS-CoV-2 infection cases will be counted starting 14 days after the second injection of mRNA-1273, i.e. date of documented infection ‐ Date of the 2nd injection ≥ 14. SARS-CoV-2 infection cases will also be summarized based on tests performed at least 14 days after first dose of IP.

In Part 1C-1, SARS-CoV-2 infection cases will be counted starting 14 days after the booster dose of mRNA-1273, i.e. date of documented infection ‐ Date of the booster injection ≥ 14.

6.5.1.2.Derivation of Asymptomatic SARS-CoV-2 Infection

This is an exploratory efficacy endpoint: the incidence of asymptomatic SARS-CoV-2 infection measured by RT-PCR and/or serology tests obtained at post-baseline visits counted starting 14 days after the second injection of mRNA-1273 in long-term (including Part 1B), and cases counted starting 14 days after the booster dose of mRNA-1273 in Part 1C-1, in participants with negative SARS-COV-2 status at baseline of each part.

Asymptomatic SARS-CoV-2 infection is identified by absence of symptoms and infections as detected by RT-PCR or serology tests. Specifically:

- Absent of COVID-19 symptoms
- AND at least one from below:
  - bAb level against SARS-CoV-2 nucleocapsid protein negative (as measured by Roche Elecsys) at baseline of each part that becomes positive (as measured by Roche Elecsys) post-baseline, OR
  - Positive RT-PCR test post-baseline (at scheduled or unscheduled/illness visits)

The date of documented asymptomatic infection is the earlier date of positive serology test result based on bAb specific to SARS-CoV-2 nucleocapsid due to infection, or positive RT-PCR at scheduled visits, with absence of symptoms.

6.5.1.3.Derivation of COVID-19

This is a exploratory efficacy endpoint: the incidence of the first occurrence of COVID-19 starting 14 days after the second dose of IP in long-term (including Part 1B), Part 2 and Part 3, cases counted starting 14 days after the first dose of IP in Part 3, and cases counted starting 14 days after the booster dose of mRNA-1273 in Part 1C-1 and Part 1C-2.

COVID-19 is defined as symptomatic disease based on the criteria specified in Section 3.2. Cases are defined as participants meeting clinical criteria based on both symptoms for COVID-19 and positive RT-PCR test results.

Surveillance for COVID-19 symptoms will be conducted via biweekly telephone calls or eDiary. Subjects reporting COVID-19 symptoms, as defined in Section 7.3.2 of the protocol, will be arranged an illness visit to collect an NP swab.

For this efficacy endpoint, a COVID-19 case will be identified as a positive post-baseline RT-PCR test result, together with eligible symptoms, i.e. a positive PCR result of the eligible symptoms summarized below in Table 2.

Table 2. Derivation for COVID-19

	**COVID-19**
Post-baseline PCR result	Positive, **AND**
Systemic Symptoms	at least **TWO** of the following **systemic symptoms**: Fever (≥ 38ºC/≥ 100.4º F), chills, myalgia, headache, sore throat, new olfactory and taste disorder(s); **OR**
Respiratory symptoms	at least **ONE** of the following **respiratory** signs/symptoms: cough, shortness of breath or difficulty breathing, OR clinical or radiographical evidence of pneumonia.

The date of documented COVID-19 (case) will be the later date of ([2 systemic symptoms reported, or respiratory symptom reported] and, [date of positive PCR test]). Specifically, the date of documented COVID-19 will be the later date of the following two dates (date of positive PCR test, and the date of eligible symptom(s)), and the two dates should be within 14 days of each other.

- Date of positive PCR test,
- Date of eligible symptom(s), defined as earliest of

- Respiratory symptom: earliest date of an eligible respiratory symptom is reported
- Systemic symptoms: earliest date of 2 eligible systemic symptom is reported

6.5.1.4.Derivation of Secondary Case (CDC Case) Definition of COVID-19

This is a exploratory efficacy endpoint: the incidence of the first occurrence of COVID-19 cases meeting the secondary case (CDC case) definition, starting 14 days after the second dose of IP in Part 1B, Part 2 and Part 3, starting 14 days after the first dose of IP in Part 3, COVID-19 cases counted starting 14 days after the booster dose of mRNA-1273 in Part 1C-1 and Part 1C-2.

The secondary case definition of COVID-19 is defined by the following criteria:

- One systemic or respiratory symptoms: fever (temperature > 38ºC/≥ 100.4ºF), or chills, cough, shortness of breath or difficulty breathing, fatigue, muscle aches, or body aches, headache, new loss of taste or smell, sore throat, congestion or runny nose, nausea, or vomiting or diarrhea, AND
- At least one positive RT-PCR test for SARS-CoV-2

Date of the documented secondary definition of COVID-19 will be later date of:

- Date of the positive RT-PCR test (prompt by symptom)
- Date of eligible symptom for secondary definition of COVID-19, defined as the earliest date of first eligible symptom is reported

and the two dates should be within 14 days of each other.

6.5.2. Analysis Method

The number and percentage of subjects who had an event will be summarized in the mITT1 Set for the long-term analysis and Part 1C-1 booster dose phase anlaysis, and FAS for Part 1C-2, Part 2 and Part 3 analysis.

The incidence rate will be provided by vaccination group if applicable, calculated as the number of cases divided by the total person-time. The 95% CI of the incidence rate will be calculated using the exact method (Poisson distribution) and adjusted by person-time.

Person-time is defined as the total time from date of the 1st dose of each part to the date of event, dose date in next part, last date of study participation, censoring time, or efficacy data cutoff date, whichever is earlier.

Incidence rate will also be analyzed by time period or by calendar month as applicable.

6.5.3. Sensitivity Analysis

Sensitivity analysis for these efficacy endpoints may be performed with the same methods described above based on the FAS in long-term, or Part 1C-1, and with cases counted starting at different time points as applicable.

6.6. Exploratory Analysis

6.6.1. SARS-CoV-2 Exposure and Symptoms

SARS-CoV-2 reported exposure history and symptoms assessment will be assessed during the study.

SARS-CoV-2 reported exposure history and symptoms assessment will be provided in a listing for each Part separately as applicable.

6.6.2. Exploratory Analysis of Efficacy Endpoints

For the exploratory analysis of efficacy endpoints, in addition to the efficacy endpoint COVID-19 and secondary case (CDC definition) COVID-19 based on eligible symptoms and confirmed positive RT-PCR results (central lab or local diagnostic test) originally defined in SAP, a sensitivity analysis using efficacy endpoint COVID-19 and secondary case (CDC definition) COVID-19based on both RT-PCR results and other COVID-19 test results including home antigen tests will also be derived. Specifically, each COVID-19 case will be based on eligible symptom(s) and all positive COVID-19 test results including RT-PCR (central lab or local diagnostic test) and (home) antigen test results.

6.7. Interim Analysis

More than one IA may be performed.

- The IA of immunogenicity, safety, and efficacy will be performed after Day 57 immunogenicity data are available for the immunogenicity subset and at least 1,500 participants (1,000 participants receiving mRNA-1273) have completed Day 57 (1 month after Dose 2, Part 1A). This IA will be considered the primary

analysis of immunogenicity for Part 1A. Details of this IA is decribed in SAP for Part 1A.

- An IA of immunogenicity and safety may be performed after all or subset of participants who receive BD have completed BD-Day 29 after the BD in Part 1C-1.
- An IA of immunogenicity and safety may be performed after all or subset of participants who receive heterologous BD have completed BD-Day 29 after the BD in Part 1C-2.
- An IA of immunogenicity and safety may be performed after Day 57 immunogenicity data are available after all or a subset of participants in Part 2 have completed Day 57 (1 month after Dose 2, Part 2).
- An IA of immunogenicity and safety may be performed after all or a subset of participants who receive 50 µg mRNA-1273.222 dose have completed Day 29 (1 month after Dose 1) and an additional IA may be performed in all or a subset of participants who completed Day 209 (1 month after Dose 2) in Part 3.
- At the Sponsor's discretion, a CSR may be developed for the IA.

6.8. Final Analysis

The final analysis of all applicable endpoints will be performed after all participants have completed all planned study procedures. Results of this analysis will be presented in a final CSR, including individual listings.

7. References

Department of Health and Human Services (DHHS), Food and Drug Administration, Center for Biologics Evaluation and Research (US). Guidance for industry: Toxicity grading scale for healthy adult and adolescent volunteers enrolled in preventative vaccine clinical trials. September 2007 [cited 2019 Apr 10] [10 screens].

Available from:
https://www.fda.gov/downloads/BiologicsBloodVaccines/GuidanceComplianceRegulatoryInformation/Guidances/Vaccines/ucm091977.pdf.List of Appendices

Bonett D., Price R., Adjusted Wald Confidence Interval for a Difference of Binomial Proportions Based on Paired Data, Journal of Educational and Behavioral Statistics, Aug 2012.

8. List of Appendices

8.1. Appendix A Standards for Safety and Immunogenicity Variable Display in TFLs

Continuous Variables: The precision for continuous variables will be based on the precision of the data itself. The mean and median will be presented to one decimal place more than the original results; the SD will be presented to two decimal places more than the original results; the minimum and maximum will be presented to the same precision as the original results.

Categorical Variables: Percentages will be presented to 1 decimal place.

8.2. Appendix B Analysis Visit Windows for Safety and Immunogenicity Analysis

Safety and Immunogenicity Analysis will be summarized using the following analysis visit window for post injection assessments:

Step 1: If the safety and immunogenicity assessments are collected at scheduled visit, i.e. nominal scheduled visit, the data collected at scheduled visit will be used.

Step 2: If the safety and immunogenicity assessments are not collected at the scheduled visit, assessments collected at unscheduled visit will be used using the analysis visit windows described in Table 3 below.

If a subject has multiple assessments within the same analysis visit, the following rule will be used:

- If multiple assessments occur within a given analysis visit, the assessment closest to the target study day will be used.
- If there are 2 or more assessments equal distance to the target study day, the last assessment will be used.

Table 3. Visit Window

Visit	Target Study Day	Visit Window in Study Day
**Nasopharyngeal or Nasal Swabs for SARS-CoV-2**		
Part 1B, Part 1C-1 and Part 1C-2		

OL-Day 1	1 (Date of First Injection in Part 1B) relative to OL-Day 1	≤1 and VISIT is OL-Day 1
OL-Day 29 (Month 1)	29 (Date of Second Injection in Part 1B) relative to OL-Day 1	[2, 43]
OL-Day 57 (Month 2)	57 relative to OL-Day 1	≥44 and before BD-Day 1
BD-Day 1	1 (Date of Injection in Part 1C-1, Part 1C-2) relative to BD-Day 1	≤1 and VISIT is BD-Day 1
Post BD-Day 1	≥2 relative to BD-Day 1	≥2
Part 2		
Day 1	1 (Date of First Injection)	1, Pre-first-dose
Day 29 (Month 1)	29 (Date of Second Injection)	[2, 43]
Day 57 (Month 2)	57	[44, 133]
Day 209 (Month 7)	209	≥134
Part 3		
Day 1	1 (Date of First Injection)	1, Pre-first-dose
Day 29 (Month 1)	29	[2, 57]
Day 85 (Month 3)	85	[58, 133]
Day 181 (Month 6)	181 (Date of Second Injection	[134, 195]
Day 209 (Month 7)	209	≥196
**Vital Signs**		
Part 1B, Part 1C-1 and Part 1C-2		
OL-Day 1	1 (Date of First Injection in Part 1B) relative to OL-Day 1	≤1 and VISIT is OL-Day 1, Pre-first-dose
OL-Day 1	1 (Date of First Injection in Part 1B) relative to OL-Day 1	≤1 and VISIT is OL-Day 1, Post-first-dose
OL-Day 29 (Month 1)	29 (Date of Second Injection in Part 1B) relative to OL-Day 1	[2, 43] Pre-second-dose
OL-Day 29 (Month 1)	29 (Date of Second Injection in Part 1B) relative to OL-Day 1	[2, 43] Post-second-dose

OL-Day 57 (Month 2)	57 relative to OL-Day 1	≥44 and before BD-Day 1 for subject receiving BD
OL-Day 57 (Month 2)	57 relative to OL-Day 1	[44, 117] for subject declining BD
OL-Day 178 (Month 6)	178 relative to OL-Day 1	≥118 for subject declining BD
BD-Day 1	1 (Date of Injection in Part 1C-1, Part 1C-2) relative to BD-Day 1	≤1 and VISIT is BD-Day 1, Pre-dose
BD-Day 1	1 (Date of Injection in Part 1C-1, Part 1C-2) relative to BD-Day 1	≤1 and VISIT is BD-Day 1, Post-dose
BD-Day 29 (Month 1)	29 relative to BD-Day 1	[2, 105]
BD-Day 181 (Month 6)	181 relative to BD-Day 1	[106, 271]
BD-Day 361 (Month 12)	361 relative to BD-Day 1	≥272
Part 2		
Day 1	1 (Date of First Injection)	≤1, Pre-first-dose
Day 1	1 (Date of First Injection)	1, Post-first-dose
Day 29 (Month 1)	29 (Date of Second Injection)	[2, 43] Pre-second-dose
Day 29 (Month 1)	29 (Date of Second Injection)	[2, 43] Post-second-dose
Day 57 (Month 2)	57	[44, 133]
Day 209 (Month 7)	209	[134, 301]
Day 394 (Month 13)	394	≥302
Part 3		
Day 1	1 (Date of First Injection)	≤1, Pre-first-dose
Day 1	1 (Date of First Injection)	1, Post-first-dose
Day 29 (Month 1)	29	[2, 105]
Day 181 (Month 6)	181 (Date of Second Injection	[106, 195] Pre-second-dose
Day 181 (Month 6)	181 (Date of Second Injection	[106, 195] Post-second-dose
Day 209 (Month 7)	209	[196, 285]
Day 361 (Month 12)	361	≥286
**Immunogenicity**		
Part 1B, Part 1C-1 and Part 1C-2		

OL-Day 1	1 (Date of First Injection in Part 1B) relative to OL-Day 1	1, Pre-first-dose
OL-Day 57 (Month 2)	57 relative to OL-Day 1	≥44 and before BD-Day 1 for subject receiving BD
OL-Day 57 (Month 2)	57 relative to OL-Day 1	[44, 117] for subject declining BD
OL-Day 178 (Month 6)	178 relative to OL-Day 1	≥18 for subject declining BD
BD-Day 1	1 (Date of Injection in Part 1C-1, Part 1C-2) relative to BD-Day 1	≤1 and VISIT is BD-Day 1
BD-Day 29 (Month 1)	29 relative to BD-Day 1	[2, 105]
BD-Day 181 (Month 6)	181 relative to BD-Day 1	[106, 271]
BD-Day 361 (Month 12)	361 relative to BD-Day 1	≥272
Part 2		
Day 1	1 (Date of First Injection)	1, Pre-first-dose
Day 29 (Month 1)	29 (Date of Second Injection)	[2, 43]
Day 57 (Month 2)	57	[44, 71]
Day 85 (Month 3)	85	[72, 147]
Day 209 (Month 7)	209	[148, 301]
Day 394 (Month 13)	394	≥302
Part 3		
Day 1	1 (Date of First Injection)	1, Pre-first-dose
Day 29 (Month 1)	29	[2, 57]
Day 85 (Month 3)	85	[58, 133]
Day 181 (Month 6)	181 (Date of Second Injection	[134, 195]
Day 209 (Month 7)	209	[196, 285]
Day 361 (Month 12)	361	≥286

8.3. Appendix A Imputation Rules for Missing Prior/Concomitant Medications and Non-Study Vaccinations

Imputation rules for missing or partial medication start/stop dates are defined below:

1. Missing or partial medication start date:
   - If only Day is missing, use the first day of the month, unless:
     - The medication end date is after the date of first injection or is missing AND the start month and year of the medication coincide with the start month and year of the first injection. In this case, use the date of first injection
   - If Day and Month are both missing, use the first day of the year, unless:
     - The medication end date is after the date of first injection or is missing AND the start year of the medication coincide with the start year of the first injection. In this case, use the date of first injection
   - If Day, Month and Year are all missing, the date will not be imputed, but the medication will be treated as though it began prior to the first injection for purposes of determining if status as prior or concomitant.
2. Missing or partial medication stop date:
   - If only Day is missing, use the earliest date of (last day of the month, study completion, discontinuation from the study, or death).
   - If Day and Month are both missing, use the earliest date of (last day of the year, study completion, discontinuation from the study, or death).
   - If Day, Month and Year are all missing, the date will not be imputed, but the medication will be flagged as a continuing medication.

In summary, the prior, concomitant or post categorization of a medication is described in Table 4 below.

Table 4. Prior, Concomitant, and Post Categorization of Medications and Non-study Vaccinations

	**Medication Stop Date**		
**Medication Start Date**	**< First Injection Date of IP**	**≥ First Injection Date and ≤ 28 Days After Last Injection**	**≥ 28 Days After Last Injection [2]**
< First injection date of IP [1]	P	P, C	P, C, A
≥ First injection date and ≤ 28 days after last injection	-	C	C, A
> 28 days after last injection	-	-	A

A: Post; C: Concomitant; P: Prior
[1] includes medications with completely missing start date
[2] includes medications with completely missing end date

8.4. Appendix B Imputation Rules for Missing AE dates

Imputation rules for missing or partial AE start dates and stop dates are defined below:

1. Missing or partial AE start date:

- If only Day is missing, use the first day of the month, unless:
  - The AE end date is after the date of first injection or is missing AND the start month and year of the AE coincide with the start month and year of the first injection. In this case, use the date and time of first injection, even if time is collected.
- If Day and Month are both missing, use the first day of the year, unless:
  - The AE end date is after the date of first injection or is missing AND the start year of the AE coincides with the start year of the first injection. In this case, use the date of first injection

- If Day, Month and Year are all missing, the date will not be imputed. However, if the AE end date is prior to the date of first injection, then the AE will be considered a pre-treatment AE. Otherwise, the AE will be considered treatment-emergent.

2. Missing or partial AE end dates will not be imputed.
